# Anle138b: a novel oligomer modulator for disease-modifying therapy of neurodegenerative diseases such as prion and Parkinson’s disease

**DOI:** 10.1007/s00401-013-1114-9

**Published:** 2013-04-19

**Authors:** Jens Wagner, Sergey Ryazanov, Andrei Leonov, Johannes Levin, Song Shi, Felix Schmidt, Catharina Prix, Francisco Pan-Montojo, Uwe Bertsch, Gerda Mitteregger-Kretzschmar, Markus Geissen, Martin Eiden, Fabienne Leidel, Thomas Hirschberger, Andreas A. Deeg, Julian J. Krauth, Wolfgang Zinth, Paul Tavan, Jens Pilger, Markus Zweckstetter, Tobias Frank, Mathias Bähr, Jochen H. Weishaupt, Manfred Uhr, Henning Urlaub, Ulrike Teichmann, Matthias Samwer, Kai Bötzel, Martin Groschup, Hans Kretzschmar, Christian Griesinger, Armin Giese

**Affiliations:** 1 Zentrum für Neuropathologie und Prionforschung, Ludwig-Maximilians-Universität München, Feodor-Lynen-Str. 23, 81377 Munich, Germany; 2 NMR based structural Biology, Max Planck Institute for Biophysical Chemistry, Am Fassberg 11, 37077 Göttingen, Germany; 3 DFG Center for Nanoscale Microscopy and Molecular Physiology of the Brain (CNMPB), Göttingen, Germany; 4 Neurologische Klinik, Klinikum der Ludwig-Maximilians-Universität München, Marchioninistr. 15, 81377 Munich, Germany; 5 Medical Faculty, Institute for Anatomy, TU-Dresden, Dresden, Germany; 6 Friedrich-Loeffler-Institut, Bundesforschungsinstitut für Tiergesundheit, Greifswald-Insel Riems, Germany; 7 BioMolekulare Optik, Ludwig-Maximilians-Universität, Munich, Germany; 8 German Center for Neurodegenerative Diseases (DZNE), Göttingen, Germany; 9 Neurologie, Universitätsmedizin Göttingen, Göttingen, Germany; 10 Labor für Pharmakokinetik, Max-Planck-Institut für Psychiatrie, Munich, Germany; 11 Bioanalytische Massenspektrometrie, Max-Planck-Institut für biophysikalische Chemie, Göttingen, Germany; 12 Bioanalytics, Department of Clinical Chemistry, University Medical Center, Göttingen, Germany; 13 Tierhaltung, Max-Planck-Institut für biophysikalische Chemie, Göttingen, Germany; 14 Zelluläre Logistik, Max-Planck-Institut für biophysikalische Chemie, Göttingen, Germany; 15 Present Address: Institut für Immunologie, Universitätsklinikum Schleswig-Holstein, Kiel, Germany; 16 Present Address: Department of Vascular Medicine, UKE, Hamburg, Germany

## Abstract

In neurodegenerative diseases such as Alzheimer’s disease (AD), Parkinson’s disease (PD) and prion diseases, deposits of aggregated disease-specific proteins are found. Oligomeric aggregates are presumed to be the key neurotoxic agent. Here we describe the novel oligomer modulator anle138b [3-(1,3-benzodioxol-5-yl)-5-(3-bromophenyl)-1*H*-pyrazole], an aggregation inhibitor we developed based on a systematic high-throughput screening campaign combined with medicinal chemistry optimization. In vitro, anle138b blocked the formation of pathological aggregates of prion protein (PrP^Sc^) and of α-synuclein (α-syn), which is deposited in PD and other synucleinopathies such as dementia with Lewy bodies (DLB) and multiple system atrophy (MSA). Notably, anle138b strongly inhibited all prion strains tested including BSE-derived and human prions. Anle138b showed structure-dependent binding to pathological aggregates and strongly inhibited formation of pathological oligomers in vitro and in vivo both for prion protein and α-synuclein. Both in mouse models of prion disease and in three different PD mouse models, anle138b strongly inhibited oligomer accumulation, neuronal degeneration, and disease progression in vivo. Anle138b had no detectable toxicity at therapeutic doses and an excellent oral bioavailability and blood–brain-barrier penetration. Our findings indicate that oligomer modulators provide a new approach for disease-modifying therapy in these diseases, for which only symptomatic treatment is available so far. Moreover, our findings suggest that pathological oligomers in neurodegenerative diseases share structural features, although the main protein component is disease-specific, indicating that compounds such as anle138b that modulate oligomer formation by targeting structure-dependent epitopes can have a broad spectrum of activity in the treatment of different protein aggregation diseases.

**Electronic supplementary material:**

The online version of this article (doi:10.1007/s00401-013-1114-9) contains supplementary material, which is available to authorized users.

## Introduction

As life expectancy continues to rise, the increasing prevalence of age-related neurodegenerative diseases poses major problems to public health. For example, PD affects about 1 % of people beyond 65 years of age [19]. Pathologically, PD is characterized by deposition of α-synuclein (α-syn) aggregates and degeneration of dopaminergic neurons in the substantia nigra resulting in impaired motor functions. A crucial role of α-syn aggregates in disease pathogenesis is suggested by abundant evidence including (1) the consistent detection of α-syn deposits in affected brain areas, (2) pathogenic mutations affecting the α-syn gene in familial PD and association of the α-syn locus with idiopathic PD in genome-wide association studies, and (3) experimental evidence in vitro, in cell culture, and in animal models [34, 57].

Several studies indicate that α-synucleinopathies as well as other protein aggregation diseases share key molecular features [29, 58] including aggregate toxicity and spread by seeded aggregation with prion disorders [1, 31]. Prion diseases are invariably fatal neurodegenerative diseases that include Creutzfeldt-Jakob disease (CJD) and bovine spongiform encephalopathy (BSE). They are caused by an unconventional infectious agent which consists primarily of the misfolded, aggregated, beta-sheet rich PrP^Sc^ isoform of the membrane glycoprotein PrP^C^ [51]. The available evidence suggests that PrP^Sc^ acts both as a template for this conversion and as a neurotoxic agent causing neuronal dysfunction and cell death [13, 51].

Recent evidence indicates that pathological oligomers [7] rather than large fibrillar deposits constitute the key neurotoxic species [4, 22, 35]. Thus, we set out to discover small molecules targeting pathological oligomers to provide a therapeutic strategy for causal disease-modifying treatment of these devastating diseases and other protein aggregation disorders for which only symptomatic treatment is available so far [20, 22, 59].

## Materials and methods

### Screening for anti-prion compounds

#### Compound libraries

**Fig. 1:**
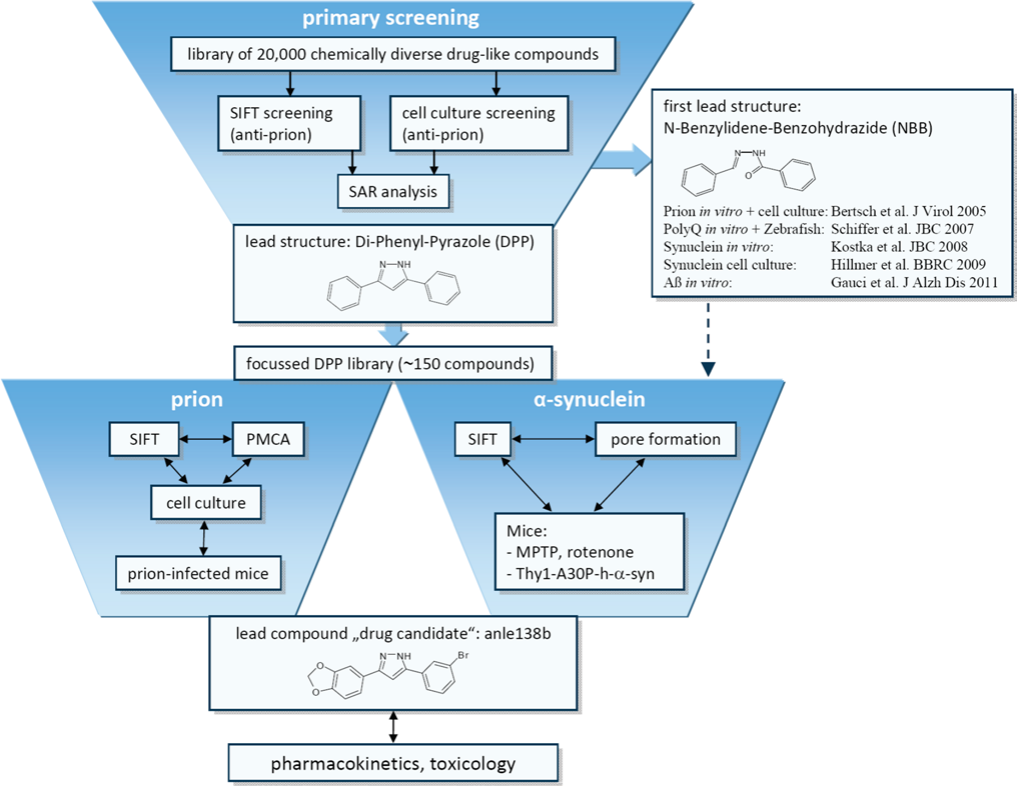
Summary of experimental strategy. In a first project phase, we tested a library of 10,000 chemically diverse drug-like compounds in regard to inhibition of prion protein aggregation in a molecular SIFT screening assay. Based on this data in combination with testing of primary hits in a cellular anti-prion assay, we identified *N*-benzylidene-benzohydrazide (NBB) derivatives as a new lead structure with anti-prion activity providing a proof of principle of the experimental strategy. Notably, the same compounds also inhibit Poly-Q and α-syn aggregation both in vitro and in vivo. However, NBB’s contain a Schiff’s base like =N–NH–CO– structure that can result in rapid metabolism in vivo. In order to identify further lead structures with favourable medicinal chemical properties, we continued by screening of 10,000 additional compounds and performed a parallel analysis of all these 20,000 compounds in a microtiter plate based high-throughput anti-prion cell culture assay followed by structure–activity and cluster analysis of the data obtained with these two independent approaches (Suppl. Fig. 1). This analysis identified a cluster of highly active compounds belonging to the chemical compound class of 3,5-diphenyl-pyrazole (DPP) derivatives. A pilot experiment with one DPP compound from the initial screening library showed a significant effect on survival in prion-infected mice (Suppl. Fig. 2). Therefore, we synthesized a focused library of ~150 DPP-related compounds (Suppl. “Compound Synthesis”) for further testing in vitro, in cell culture, and in vivo in regard to therapeutic effects on prion and α-syn aggregation and disease progression in animal models. Based on results from the SIFT and cell culture anti-prion assays, 38 compounds were selected for in vivo testing in prion-infected mice (Suppl. Fig. 3). In these experiments, the compound anle138b [3-(1,3-benzodioxol-5-yl)-5-(3-bromophenyl)-1*H*-pyrazole] showed the highest anti-prion activity. In addition, anle138b showed efficacy in in vitro and in vivo models for synucleinopathies such as PD

The two libraries screened contain 10,000 compounds each and are called DIVERSet1 and DIVERSet2, because they cover only a part of the larger DIVERSet library (ChemBridge Corp., San Diego, CA). DIVERSet is a collection of rationally selected, diverse, drug-like small molecules. The compounds were supplied in dimethyl sulfoxide (DMSO) solution on 96-well microtiter plates. A database containing molecular structures and some physico-chemical data for each of the compounds is available at www.chembridge.com. Based on the discovery of the new di-phenyl-pyrazole (DPP) lead structure (Fig. 1), a focussed compound library was generated. Details regarding synthesis and quality control of >150 newly synthesized DPP-related compounds are provided in the Supplement “Compound Synthesis”. Unless otherwise noted, materials and solvents were purchased from commercial sources (Sigma-Aldrich, Germany; Alfa Aesar, Germany) and used without purification.

#### Assay for PrP^C^–PrP^Sc^ association by scanning for intensely fluorescent targets (SIFT)

Recombinant PrP 23-231 (rPrP) was produced and purified essentially as described [2]. L42 monoclonal antibody (r-biopharm, Darmstadt, Germany) was labelled with Alexa-Fluor-647 (Invitrogen, Eugene) according to the manufacturer’s manual. Recombinant mouse PrP 23-231 was labelled with Alexa-Fluor-488 (Invitrogen, Eugene) in 20 mM potassium phosphate buffer, pH 6, 0.1 % Nonidet P40, 40 mM sodium bicarbonate buffer, pH 8.3. Unbound fluorophores were separated by gel filtration on PD10 columns (GE Healthcare, Freiburg, Germany) equilibrated with 20 mM potassium phosphate buffer, pH 6, 0.1 % Nonidet P40. PrP^Sc^ was prepared from brain of CJD patients according to Safar et al. [54] and aliquots of the final pellet resuspended in 1× PBS + 0.1 % sarcosyl solution were diluted 1:5 into buffer A (20 mM potassium phosphate buffer at pH 6.0, 0.1 % Nonidet P40) and sonicated in a water-bath sonicator for 60 s. After centrifugation at 1,000 rpm for 1 min the supernatant was diluted 1:100 in buffer A for the assay. A mixture of labelled mouse rPrP and labelled L42 monoclonal antibody was prepared in buffer A so that the labelled molecules were approximately equally abundant at 2–6 nM. In a 20-μl assay volume 8 μl of the rPrP/antibody mixture, 2 μl compound (final concentration in primary screening: 10 μM), and 10 μl of the diluted PrP^Sc^ preparation were mixed. The samples were loaded onto 96-well plates with cover-glass bottom (Evotec-Technologies, Hamburg, Germany) and measured using dual-colour SIFT on an Insight Reader as described [2].

#### Inhibition of PrP^Sc^ accumulation in a cell-based dot blot assay

Mouse neuroblastoma (ScN2a) and scrapie mouse brain (SMB) cells (TSE Resource Centre) [6], infected with the Rocky Mountain Laboratory (RML) scrapie strain, were seeded in a concentration of 20,000 cells per well in 100 μl of medium at a Costar 3599 flat-bottom 96-well plate (Corning Inc., Corning, NY) prior to compound addition. Test compounds were added to the cell medium in a final dilution of 20 and 2 μM, respectively. After an incubation period of 3 days at 37 °C (ScN2a cells) or at 35 °C (SMB cells) in a CO_2_ incubator the cultures were lysed and analyzed for PrP^Sc^ formation.

After cell medium removal, 100 μl of lysis buffer (50 mM Tris/HCl, ph 8.0, 150 mM NaCl; 0.5 % (w/v) DOC; 0.5 % (v/v) Triton X-100) was added to each well for 5 min at room temperature. Using a dot blot apparatus (Sigma-Aldrich) cell lysate was transferred under vacuum to an activated polyvinylidene difluoride (PVDF) membrane (Immobilon-P; Milipore) and fixed to the membrane by incubation for 1 h at 37 °C. The membrane was incubated in lysis buffer and treated with proteinase K solution (final dilution 25 μg/ml) for 90 min at 37 °C. Subsequently, the membrane was washed twice with pure water, the denaturation solution (3 M Guanidiniumthiocyanat, 0.1 M TrisHCl pH 8.0) was added for 15 min at room temperature and membranes were washed five times with pure water. After the denaturation step the membrane was blocked with PBST-milk [5 % (w/v) non-fat milk, 0.1 % (v/v) tween-20 (Sigma) in phosphate buffered saline (PBS)] for 60 min. An appropriate dilution of polyclonal antibody “rabbit-10” [30] in PBST-milk was incubated with the membrane for 60 min. After PBST rinsing, the membrane was incubated in a 0.2-μg/ml concentration of Horseradish Peroxidase (HRP) conjugated, anti-rabbit IgG antibody (Promega) in PBST-milk for 1 h at room temperature. After additional PBST rinsing the bound antibodies were detected using a chemiluminescence reagent system (ECL, Amersham) and were visualized directly in an image analysis system (Versa Doc, Quantity One, Bio-Rad, Munich, Germany). Inhibition was calculated as relative signal volume compared to the untreated control (100 %).

### Experiments in prion-infected mice

#### Inoculation with RML prions and therapeutic treatment

For screening and structure–activity analysis, compounds were tested in regard to their inhibitory effect on PrP^Sc^ accumulation in vivo by three experimental protocols:

- Seven-week-old female C57BL/6 mice were inoculated intracerebrally (i.c.) with 30 μl of 1 % (w/v) brain homogenate (RML scrapie). Treatment was started at 80 days post infection (dpi) with 1 mg compound per day mixed with 10 μl DMSO + 200 μl peanut butter applied orally. PrP^Sc^ level in brain was measured at 120 dpi by immunoblot analysis.
- Seven-week-old female C57BL/6 mice were inoculated intraperitoneally (i.p.) with 100 μl of 1 % (w/v) brain homogenate (RML scrapie). PrP^Sc^ level in the spleen was determined at 35 dpi following 34 days of treatment with 1 mg compound mixed with 10 μl DMSO + 200 μl peanut butter per day.
- Seven-week-old female C57BL/6 mice were inoculated i.c. with 30 μl of 1 % (w/v) brain homogenate (RML scrapie). Treatment was started at 80 dpi with 0.84 mg compound (in 25 μl DMSO) per day applied by intraperitoneal injection for 14 days followed by 2 × 5 days (with 2 days without treatment in between) of 1 mg compound (in 10 μl DMSO + 40 μl vegetable oil) applied orally by gavage. PrP^Sc^ level in brain was measured at 106 dpi.

For long-term survival experiments, anle138b was administered orally in DMSO/peanut butter as described above. In a first set of experiments, 5 mg anle138b were given once daily starting either at day 80 or day 120 post i.c. infection. Animals of each treatment group were monitored daily for signs of disease by trained animal caretakers from day 80 post infection. The animals were sacrificed, when they had reached the terminal stage of the disease based on clinical signs (ataxia, tremor, difficulty in righting up from a position lying on its back and tail stiffness). Typically the disease progress through the terminal stage will lead to the death of the animal within 1 or 2 days. In addition, groups of four mice per experimental group were sacrificed at predefined time points as indicated in Fig. 3c–e. From all animals, one brain hemisphere and one half of the spleen were freshly frozen at −80 °C for biochemical analysis. The other hemisphere and the remaining half of the spleen as well as all inner organs were fixed in 4 % formaldehyde solution for histological analysis. In a further experiment, treatment with anle138b was started on the day of i.c. infection with a dose of 5 mg anle138b twice daily.

#### Histology and immunohistochemistry

Prion infectivity was inactivated by immersion in 100 % formic acid [8]. Paraffin-fixed sections (2.5 μm) of brain tissue were stained with H&E. For PrP^Sc^ detection using antibody CDC1 [50], sections were immunostained on a Ventana automated staining apparatus. To assess neuronal degeneration, neurons with pyknotic nuclei were counted in blinded slides in the cerebellar granule cell layer. This approach has been validated previously as an efficient measure for apoptotic neuronal cell death [28]. For each animal, 30 randomly chosen high power visual fields were analyzed.

#### Quantification of PrP^Sc^

For quantification of PrP^Sc^, brain homogenates were homogenized 10 % (w/v) in lysis buffer (100 mM NaCl, 10 mM EDTA, 0.5 % (v/v) NP-40, 0.5 % (w/v) deoxycholate, 10 mM Tris/HCl pH 7.4) and subjected to dot blot analysis using 6H4 antibody (Prionics, Switzerland) at a dilution of 1:5,000. For PrP^Sc^ immunoblotting, infected brain homogenates were treated with proteinase K (100 μg/ml, 1 h, 37 °C) prior to dot blot analysis. Spleens were homogenized in Dulbecco’s PBS 10 % (w/v) and PrP^Sc^ was precipitated from 500 μl of homogenates using sodium phosphotungstic acid (NaPTA) as previously described [65]. Pellets were resuspended in 20 μl of 0.1 % sarkosyl buffer and treated with proteinase K (50 μg/ml, 1 h, 37 °C). PrP was visualized with CDP-Star detection reagent (Roche, Mannheim, Germany). Quantification was performed using a Diana III luminescence imaging system along with the AIDA software package (Raytest, Straubenhardt, Germany). The relative inhibition of PrP^Sc^ accumulation compared to DMSO-treated groups was calculated as follows:

$$ \% {\text{ Inhibition}} = \, \left( { 1- \, \left( {x - s} \right)/\left( {c - s} \right)} \right) \times 100 $$

where *x* amount of PrP^Sc^ in compound-treated group at end of treatment period, *s* amount of PrP^Sc^ in control group at start of treatment period, and *c* amount of PrP^Sc^ in control group at end of treatment period.

### Protein misfolding cyclic amplification (PMCA) [5, 53]

Frozen BSE- and scrapie-infected mouse brains and human sporadic CJD (sCJD) and variant CJD (vCJD) brain samples were homogenized in cold PMCA conversion buffer, containing 1-fold PBS, 1 % Triton X-100, 5 mM EDTA, 150 mM NaCl and protease inhibitor cocktail tablets (Roche, Basel, Switzerland). Crude 10 % (w/v) homogenates were centrifuged for 10 s at 2,000×*g*. Aliquots of the supernatant were immediately frozen at −80 °C for subsequent experiments. Whole normal mouse brains were obtained from 10-week-old C57BL/6 mice. After homogenization in cold PMCA conversion buffer, 10 % (w/v) normal brain homogenates were centrifuged for 10 s at 2,000×*g*, and aliquots of the supernatant were frozen at −80 °C. Frozen cortex from non-CJD human brain was homogenized as above.

PMCA was performed in a water-bath sonicator (Misonix sonicator 3000, Misonix, Farmingdale, NY, USA), which had a microplate horn for PCR tubes. Normal brain homogenate was mixed with infected brain homogenate (100:1 v/v) and 99 μl of this mixture was transferred into 0.2-ml PCR tubes which contained 1 μl of DMSO or compound solved in DMSO. For mouse substrates 18 PMCA cycles and for human substrates 40 cycles were performed. Each cycle consisted of sonication at 60 % potency (~209 W) for 20 s followed by incubation at 37 °C for 59 min 40 s.

PMCA product was digested with 50 μg/ml proteinase K at 37 °C for 1 h. After adding an equal volume of SDS loading buffer and boiling for 10 min, samples were separated by SDS-PAGE and transferred to PVDF membranes (Immobilon-P, Millipore, MA, USA) at 12 V for 2 h. For immunoblotting, the membrane was blocked with 5 % non-fat milk in PBST and incubated with 1:5,000 diluted PrP monoclonal antibody 3F4 (Dako, Glostrup, Denmark) in PBST at room temperature for 2 h. After three washes in PBST, the membrane was immerged in a 1:5,000 diluted alkaline phosphatase conjugated anti-mouse IgG (Dako) in PBST and incubated at room temperature for 2 h. Detection was performed with CDP-Star solution (Roche, Mannheim, Germany). Immunoblots were scanned and quantified by a Diana III luminescence imaging system along with the AIDA software package (Raytest, Straubenhardt, Germany). In the Western blots shown in Fig. 4c, d “start” indicates the sample taken from the PMCA reaction mixture at time point 0 after mixing normal brain homogenate with a minute amount of PrP^Sc^ that acts as a seed for the subsequent PMCA reaction. Thus, this sample provides the reference for samples “anle234b”, “anle138b” and “DMSO”, which following PMCA incubation contain different amounts of newly formed PK-resistant PrP^Sc^ depending on the different efficacy of prion amplification during PMCA. Thus, this assay design (comparison of time points before and after PMCA amplification) does not require a “loading control”.

### α-Synuclein in vitro oligomer formation assay

Expression and purification of recombinant wild-type α-synuclein (α-syn) was performed according to established protocols [38]. Fluorescent labelling of α-syn and quality control were performed as described [27]. For α-syn aggregation experiments, a 5-fold stock solution of fluorescently labelled α-syn was prepared by mixing α-syn labelled with Alexa-488 and α-syn labelled with Alexa-647. The concentrations of α-syn-Alexa-488 and α-syn-Alexa-647 were adjusted to ~10 molecules/focal volume and ~15 molecules/focal volume, respectively.

The α-syn aggregation assay was conducted with minor modifications as published previously [38]. All experiments were performed in 50 mM Tris-buffer (pH 7.0) in a total volume of 20 μl in 384- or 96-well plates with a cover slide bottom (Evotec-Technologies, Germany). The concentration of α-syn was approximately 20 nM. In all experiments, α-syn aggregation was induced by final concentrations of 1 % DMSO and 10 μM FeCl_3_. Compounds were diluted into the assay mixture from 10-fold stock solutions containing 10 % DMSO (v/v), resulting in a final concentration of 1 % DMSO in all samples. “Low control” measurements for threshold setting were obtained without FeCl_3_ and DMSO. Experiments were started by diluting the 5-fold stock solution of fluorescently labelled α-syn in 50 mM Tris-buffer, pH 7.0, containing 1 % DMSO, 10 μM FeCl_3_ and compounds in concentrations ranging from 1–10 μM. Aggregation was monitored at room temperature for at least 2.5 h in 3–4 independent samples for each experimental group.

Aggregation of α-syn was measured by scanning for intensely fluorescent targets (SIFT) [3]. SIFT measurements were carried out on an Insight Reader (Evotec-Technologies) with dual-colour excitation at 488 and 633 nm, using a 40 × 1.2 numerical aperture microscope objective (Olympus, Japan) and a pinhole diameter of 70 μm at FIDA setting. Excitation power was 200 μW at 488 nm and 300 μW at 633 nm. Measurement time was 10 s. Scanning parameters were set to 100 μm scan path length, 50 Hz beam scanner frequency, and 2,000 μm positioning table movement. The frequency of specific combinations of “green” and “red” photon counts was recorded in a two-dimensional intensity distribution histogram, as described previously [27]. Evaluation of SIFT data in two-dimensional intensity distribution histograms was performed by summing up the numbers of photons from high intensity bins using a two-dimensional SIFT software module (Evotec-Technologies). For threshold setting, non-aggregated reference samples were used. The photon count in bins above the threshold was considered aggregate signal.

### α-syn oligomer pore formation in planar lipid bilayers

Planar lipid bilayers were generated and analyzed in a Ionovation Compact (Ionovation, Osnabrück, Germany) by the painting technique [46]. Two bath chambers (1.2 ml) separated by a Teflon-septum were filled with 250 mM KCl, 10 mM MOPS, pH = 7.2 (Merck, Darmstadt, Germany and Roth, Karlsruhe, Germany). Then, 2 μl of a 100 mg/ml-solution of purified azolectin in *n*-Decan (Ionovation, Osnabrück, Germany) were applied to a 120-μm pinhole in the septum. After 30 min incubation at room temperature, lipid was thinned out by repetitive lowering and re-raising of the buffer level until a bilayer was formed. This formation was monitored optically and by capacitance measurements. The resulting bilayers had a typical capacitance of 60–80 pF and a resistance of >100 GΩ. Measurements were performed using Ag/AgCl-electrodes connected to an EPC10-amplifier and Patchmaster software (HEKA, Lambrecht/Pfalz, Germany). The noise was ~0.5 pA at 3 kHz bandwidth. α-syn was incubated in 50 mM Tris–HCl, pH 7.0 with 1 % DMSO (Sigma-Aldrich, Taufkirchen, Germany) and 20 μM FeCl_3_ (Merck, Darmstadt, Germany) for 72 h at room temperature in a total volume of 200 μl. Concentration of alpha-synuclein was 2.1 μM. Compounds were added at the start of incubation in concentrations of 50 μM (baicalein) or 25 μM (anle138b). After bilayer formation, α-syn was added in 20 μl aliquots to the cis chamber close to the membrane and the electrophysiological properties were monitored. Threshold for pore detection was set to 72 pS. If no pore formation was detected for 5 min, the next aliquot of the same sample was added as described previously [55].

### Oral rotenone in vivo mouse model of Parkinson’s disease

One-year old C57Bl/6J mice were maintained in a constant light/dark cycle (12 h/12 h). Water and food were given ad libitum. Mice were divided into 4 groups (“no rotenone”, “rotenone/normal feed”, “rotenone/placebo feed”, “rotenone/anle138b”). A further group of mice (“rotenone/hemivagotomy”) that was used for comparison was hemivagotomized previous to rotenone treatment as previously described [49]. This procedure partially prevents the spread of pathology from the enteric nervous system to the CNS resulting in a decreased dopaminergic cell death. Oral rotenone treatment was performed as previously described [48]. Briefly, mice were treated with 0.01 ml/g body weight of solutions containing either only vehicle [4 % carboxymethylcellulose (Sigma-Aldrich, Germany) and 1.25 % chloroform (Carl Roth, Germany)] or rotenone (0.625 mg/ml rotenone (Sigma-Aldrich, Germany), 4 % carboxymethylcellulose and 1.25 % chloroform) once a day, 6 days/week for 4 months. Treatment was administered orally with the help of a 1.2 × 60-mm gavage (Unimed, Switzerland). During treatment, two groups of rotenone-treated mice were supplied with the same batch of food pellets (Ssniff, Soest, Germany) that either contained the compound (“rotenone/anle138b” group) or were prepared without anle138b (“rotenone/placebo feed” group). The other two groups (“no rotenone” and “rotenone/normal feed”) were fed with standard food pellets. The locomotor abilities of the mice were tested using an acceleration protocol of the rotarod test, as previously described [48]. The test was repeated once a month, four times a day on each animal over three consecutive days. In addition, a 1-h stool collection test was performed on 4 consecutive days once a month as previously described [49] to assess gut motility and function of the enteric nervous system. All animal experiments were carried out in accordance with the National Institutes of Health Guide for the Care and Use of Laboratory Animals that had been approved by the Saxonian Committee for Animal Research, Dresden, Germany.

### Transgenic α-synuclein mouse model

The in vivo effect of anle138b was tested in a well-established murine α-synucleinopathy model {(Thy1)-h[A30P]α-syn} on a genetic background of C57/Bl6 mice [47]. Anle138b treatment was tested against placebo treatment with the vehicle (DMSO/peanut butter). Furthermore, untreated non-transgenic C57/Bl6 mice were examined as age-matched controls. The animals were evaluated clinically regarding survival time, motor performance and body weight. In addition, we conducted histological post mortem evaluation of α-syn deposition. Treatment with anle138b and placebo, respectively, was initiated at the age of 8 weeks. Transgenic animals in the treatment and placebo group were matched both in regard to litter and sex. Anle138b was solved in DMSO and mixed with peanut butter. Five days prior to the first dose of anle138b, initial doses of 200 μl peanut butter (Barney′s best creamy peanut butter, Dockhorn & Co, Hamburg, Germany) were given to the mice once daily. During the first 2 weeks of treatment, 2 mg of anle138b dissolved in 10 μl DMSO mixed with 200 μl peanut butter were given. After 2 weeks of treatment, the dose was increased to 5 mg in 10 μl DMSO/200 μl peanut butter. At the age of 33 weeks, the dose was increased to 2 × 5 mg per day. All mice were monitored daily for signs of disease. Detailed clinical assessment including behaviour and movement was performed once a week. Every 2 weeks, rotarod performance and body weight was monitored.

For assessment of motor performance, a Rotarod advanced for five mice V4.1.1 (TSE Systems, Chesterfield, MO, USA) was used. All mice were tested regarding their rotarod performance every 2 weeks beginning with an age of 250–260 days. On the 2 days prior to the rotarod performance test, mice were trained in three trial runs each during which the rotarod accelerated from 0 to 30 rpm over a period of 180 s. On the third day, the test consisted of three runs on a rod that accelerated continuously during 300 s from 0 to 50 rpm. The latency between each trial run was at least 40 s. This paradigm was designed in a way that all mice fell from the rod at some time point [45]. The average time on the rotarod and the best performance out of three runs were evaluated. If in a run an animal fell of the rod immediately (i.e., did not stay on the accelerating rod for more than 10 s), this run was repeated. Up to a maximum of three repeats per test day were conducted.

For all mice, rotarod performance was normalized to their mean performance between 42 and 51 weeks of age. As cut-off to assess the start of neurological symptoms, we used the normalized mean rotarod performance of age-matched non-tg mice at the age of 42–51 weeks minus three sigma (=55.28). Mice with the best run below this value were considered symptomatic. Animals that were not able to stay at least once for more than 20 s on the accelerating rotarod on the test day were considered terminally ill and sacrificed. In addition, four animals matched in regard to sex and litter were sacrificed per experimental group at the age of 69 weeks for analysis of pre-terminal histopathological changes. Fluctuations in rotarod performance in the prodromal stage were determined by assessing the standard deviation of the best rotarod performance in the measurements between 300 and 450 days of life for individual animals.

All animals were submitted to pathological examination. For histopathological and immunohistochemical investigation, formalin-fixed brain tissue was used. Pathological deposits of human α-syn were detected by the anti-human-α-syn antibody 15G7 [47]. In detail, 1-μm tissue slices were deparaffinized, treated with 0.2 M boric acid, pH 9 at 63 °C for 25 min and afterwards stored in the same buffer for 30 min at room temperature. Then the slices were washed with Millipore water and stained with haemalum. For PK digestion 50 mg/ml PK stock solution was diluted 1/500 with digestion-buffer (10 mM Tris–HCl, pH 7.8 + 100 mM NaCl + 0.1 % BRIJ35). PK digestion was performed for 10 min at 37 °C. After washing, the slices were treated for 32 min with 15G7 in 1/4,000 dilution and then with a biotinylated rabbit anti-rat secondary antibody (Dako, Hamburg, Germany) at 1/300 dilution for 20 min. DAB was used for detection.

### Continuous sucrose-gradient assay

Continuous sucrose-gradient assay was performed as described by Tzaban et al. [63] with minor modifications. Briefly, 10 % brain homogenates (w/v) were prepared using 1× PBS (pH 7.2) containing 0.1 % NP-40 with protease inhibitor cocktail EDTA-free (Roche, Switzerland) and were stored at −80 °C without centrifugation. Before further processing, samples were thawed on ice. To obtain a 1 % (w/v) brain homogenate 1× PBS containing 0.5 % sodium deoxycholate and 1 % sarcosyl was added. Then samples were agitated with 1,200 rpm at 4 °C for 30 min (Thermomixer Comfort, Eppendorf, Germany). To discard debris, samples were centrifuged with 20,000*g* at 4 °C for 1 min. To prepare a sucrose-gradient solutions containing 10 mM Tris (pH 7.2), 1 % sarcosyl and sucrose (10, 20, 30, 40, 50 and 60 %, respectively) were filled into a 4-ml 11 × 60 mm polyallomer tube (Beckman coulter, USA) beginning with 200 μl of 60 % sucrose solution loaded to the bottom, then followed by 400 μl of 50–10 % sucrose. Finally, 200-μl brain homogenate was loaded on the top of the sucrose-gradient. Ultracentrifugation with 100,000*g* at 4 °C for 1 h was performed using a Sw60Ti rotor (Beckman coulter, USA). Samples for western blotting were harvested after centrifugation from the top to the bottom of the sucrose-gradient in 200 μl fractions (i.e. fraction 1 represents the top of the gradient and fraction 12 the bottom fraction). For RML samples, fractions from the sucrose-gradient were digested with 50 μg/ml PK at 37 °C for 1 h. The reaction was stopped by adding 2× loading buffer and boiling at 100 °C for 15 min. Synuclein fractions were boiled in 2× loading buffer for western blotting.

### Anle138b fluorecence measurement

Fluorescence measurements of anle138b in combination with α-syn monomers and fibrils were performed in fused silica cells (Hellma, 10 × 4 mm, Typ 117.104F-QS) with a spectrofluorometer (Horiba, Fluorolog3) in front-face geometry. A solution of 250 nM anle138b in buffer containing 0.0025 % (v/v) DMSO was prepared by adding 2.5 μl of a 10-mM anle138b solution in DMSO to 100 ml of 50 mM Tris-buffer (pH 7.0) under ultra sound treatment at about 30 °C. Two identical cells, Cell1 and Cell2, were filled with this 250-nM anle138b solution and fluorescence spectra (excitation at 300 nm, 2 μW) were recorded. At the excitation wavelength, the absorption of the sample is dominated by anle138b. Subsequently, about every 30-min small amounts of 50-μM monomeric α-syn and 13-μM aggregated α-syn were added to Cell 1 and Cell 2, respectively, and fluorescence spectra were recorded. Both cells were stirred during the fluorescence measurements. For aggregation, 50 μM α-syn in 50 mM Tris–HCl, pH = 7.0 containing 100 mM NaCl and 0.02 % of NaN_3_ was incubated for 96 h at 37 °C under constant agitation (1,400 rpm). Directly prior to the fluorescence measurements this solution was centrifuged (15 min, 10,000 rpm) to remove residual monomeric α-syn. The pellet was resuspended in Tris–HCl, pH = 7.0 and a solution of 13 μM of α-syn in the aggregated form (concentration determined by Tyrosin absorbance at 280 nm) was obtained. At 300 nm absorption and corresponding emission from the tyrosines in α-syn is small. In order to consider this contribution we performed reference measurements by adding the monomers and fibrils to cells containing the same buffer (Tris-buffer (pH 7.0), 0.0025 % (v/v) DMSO) but no anle138b. The emission recorded from these samples was subtracted from the emission recorded in the original experiment.

## Results

### Discovery of anle138b and related DPP compounds by screening for anti-prion compounds

We tested a compound collection of 20,000 chemically diverse drug-like compounds both for inhibition of prion protein aggregation using a molecular SIFT assay [2, 3] and in an anti-prion cell culture assay [25, 41]. Structure–activity and cluster analysis of the data obtained with these two independent high-throughput screening approaches identified a cluster of highly active compounds belonging to the chemical class of 3,5-diphenyl-pyrazole (DPP) derivatives (Suppl. Fig. 1). Therefore, we synthesized a focussed library of ~150 DPP-related compounds and tested these compounds in vitro for inhibition of pathological prion and α-syn oligomer formation, in cell culture, and in vivo for therapeutic effects on disease progression and oligomer formation in a range of animal models. A detailed account of our experimental drug discovery strategy is provided in Fig. 1.

**Fig. 2:**
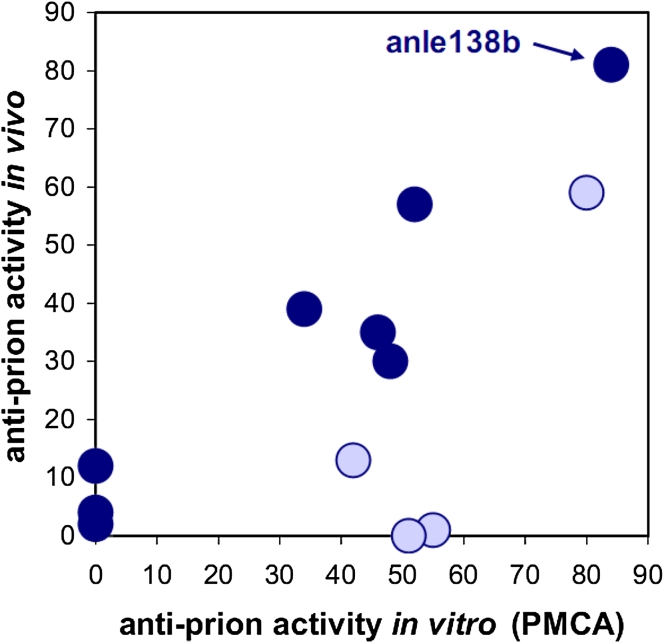
Correlation between anti-prion activity in vitro and in vivo. For compounds shown in Table 1 that reach brain levels ≥15 nmol/g (indicated by *dark blue dots*), there is a strong linear correlation (*R* = 0.951) between anti-prion activity in vitro and in vivo. Those compounds that reach lower brain levels (*light blue dots*) result in lower in vivo activities. No compounds with low anti-prion activity in the PMCA assay in vitro are active in vivo. Anle138b is the most active compound in both assays. Anti-prion activity is provided as % inhibition

Evaluation of the DPP lead structure according to medicinal chemistry criteria [12, 42, 64] indicated good metabolic stability and oral bioavailability required for long-term therapy. First in vivo experiments were done in the prion model, as this provides an authentic neurodegenerative disease model with a well-defined and relatively rapid time course. Compounds were given orally and, in addition to therapeutic efficacy, brain levels were investigated for a number of compounds (Table 1). In these experiments, anle138b [3-(1,3-benzodioxol-5-yl)-5-(3-bromophenyl)-1*H*-pyrazole] showed the highest anti-prion activity both in vivo and in vitro (Fig. 2). Comparison of anle138b with systematic variations of this structure revealed a well-defined structure–activity relationship (SAR). Regarding the 5-phenyl (R3), bromine in *meta*-position led to the highest inhibitory activity, whereas the change of bromine location to *para*- or *ortho*-position in the 5-phenyl ring (R3) reduced or abolished the activity, respectively. Interestingly, substitution in the *ortho*-position for steric reasons tilts this phenyl ring so that the molecule is no longer planar (Suppl. Fig. 4). Testing different halogen atoms in the *meta*-position revealed a bell-shaped correlation with the size of the substituent. Regarding the central five-membered ring (R2), pyrazole was associated with highest activity. For the substituent at the 3-phenyl (R1), SAR was more complex, as modifications in this position strongly affected bioavailability in the brain. 1,3-Benzodioxole as in anle138b was associated with best bioavailability and good inhibitory activity in vitro, thus resulting in highest activity in vivo. Notably, when brain levels are taken into account, there is a strong correlation between anti-prion activity in vitro and in vivo (Fig. 2) indicating that the therapeutic effect in vivo is based on direct targeting of PrP^Sc^.

**Table 1:**
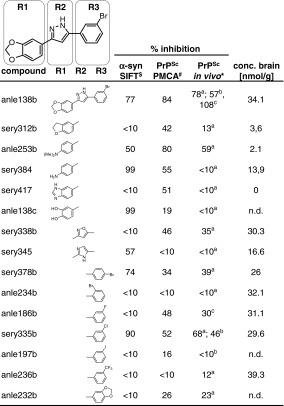
Structure–activity relationship for various DPP-derivatives

The table summarizes the effect of various compounds in regard to α-syn oligomer formation and prion propagation in vitro (SIFT, PMCA) and in vivo in prion-infected mice, as well as compound concentrations achieved in the brain 4 h after oral application of 1 mg compound in DMSO/peanut butter. For all compounds other than anle138b, only the part of the molecule (R1, R2, or R3) that differs from this lead structure is displayed

^$^Inhibition relative to high control, 10 μM compound, 1 % DMSO, 10 μM FeCl_3_

^#^Inhibition relative to high control, 10 μM compound, RML prion strain

* Relative inhibition of PrP^Sc^ accumulation normalized to DMSO-treated group (0 % inhibition) and PrP^Sc^ level at start of treatment (100 % inhibition)

^a^PrP^Sc^ level in brain 120 days after i.c. infection and treatment for 40 days with 1 mg compound (oral, in peanut butter)

^b^PrP^Sc^ level in spleen determined 35 days after i.p. infection followed by 34 days of treatment with 1 mg compound (oral, in peanut butter)

^c^PrP^Sc^ level in brain at 106 days after i.c. infection and treatment for 24 days [14 days i.p. (0.84 mg compound); 2 × 5 days oral by gavage (1 mg)]

### Efficacy of treatment started after onset of disease

**Fig. 3:**
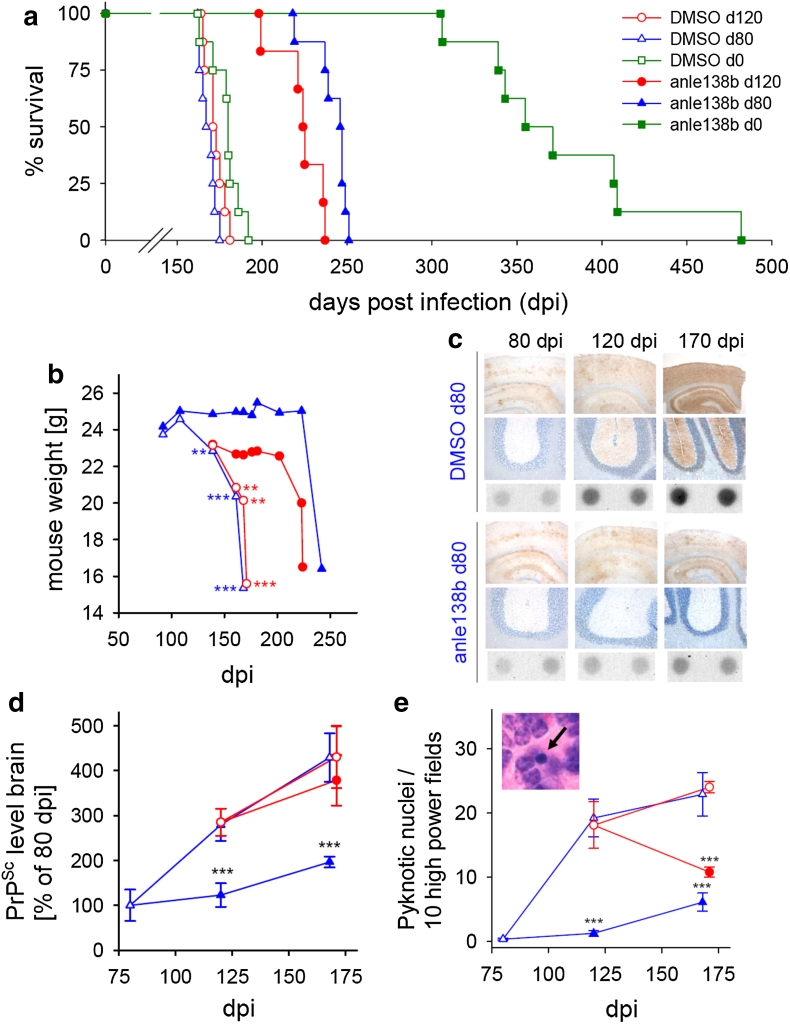
Influence of daily anle138b treatment on PrP^Sc^ accumulation and prion pathology of mice infected with RML scrapie. **a** Survival curves of mice treated orally with anle138b beginning at 0, 80 or 120 days post i.c. prion infection. Treatment resulted in prolonged survival, even when started at an advanced disease stage at 120 dpi. For detailed statistics see Suppl. Fig. 6, *n* = 7–9. **b** Control mice showed progressive weight loss starting after 100 dpi. Treatment with anle138b from 80 dpi onwards prevents weight loss for ~100 days. Treatment from 120 dpi inhibits further weight loss for ~70 days. **c** Immunohistochemistry (*upper row* cortex/hippocampus, *middle row* cerebellum) and dot blot analysis (*lower row*) showed that anle138b treatment inhibits PrP^Sc^ accumulation in comparison to DMSO-treated control animals. **d** Quantification of brain PrP^Sc^ levels at different time points shows highly significant inhibition of PrP^Sc^ accumulation in mice treated from 80 dpi onwards. PrP^Sc^ accumulation is also reduced in animals treated from 120 dpi. **e** Histological analysis reveals a significantly reduced number of pyknotic nuclei both in mice treated from 80 and 120 dpi onwards. *Inset* shows an example of a pyknotic granule cell nucleus (*arrow*). *Error bars* in (**d**) and (**e**) indicate standard error (*n* = 4), **p < 0.01, ***p < 0.001. The legend shown in (**a**) also applies to (**b**–**e**)

SAR was primarily investigated through the effect of treatment for 40 days [80–120 days post infection (dpi)] on PrP^Sc^ accumulation, which provides a rapid and reliable biochemical quantification and thus allowed testing of a large number of compounds in vivo. To further investigate the mode of action and therapeutic potency, we chose anle138b, which in our SAR study had the highest therapeutic efficacy in vivo. To be useful for treatment in humans, a compound should also be effective when given after signs and symptoms of disease are detectable. Thus, we analyzed the effect on clinical outcome (i.e. motor performance, weight loss and survival) for treatment started at different time points after intracerebral infection (Fig. 3; Suppl. Fig. 5). Without treatment, at about 80 dpi subtle clinical signs can be observed [44] and PrP^Sc^ is detectable in the brain (Fig. 3c), at 120 dpi obvious signs of disease are present (Fig. 3b, c). Even start of treatment with anle138b after onset of disease at 120 dpi resulted in a substantially prolonged survival and preservation of body weight (Fig. 3a, b; Suppl. Fig. 6). To our knowledge, the prolongation of survival obtained with anle138b is the largest that has been found for any drug-like compound tested so far in late-stage treatment experiments [15, 17, 36, 62]. The medicinal chemistry optimization of DPP compounds thus was essential for high in vivo efficacy compared to the efficacy observed for the first hit compounds derived from the compound collection used for primary screening [25, 41].

Analysis of mice at different time points during treatment revealed that anle138b strongly inhibited accumulation of PrP^Sc^ (Fig. 3c, d) and neuronal cell death (Fig. 3e) even when start of treatment was in the symptomatic phase. Interestingly, the number of pyknotic nuclei indicating apoptotic neuronal cell death seemed not to depend on the absolute amount of PrP^Sc^ but correlated with the rate of PrP^Sc^ amplification. This is in line with published data [13] and provides an explanation for the strong therapeutic effect found also in the late-treatment group.

### Targeting of pathological protein aggregation by anle138b

**Fig. 4:**
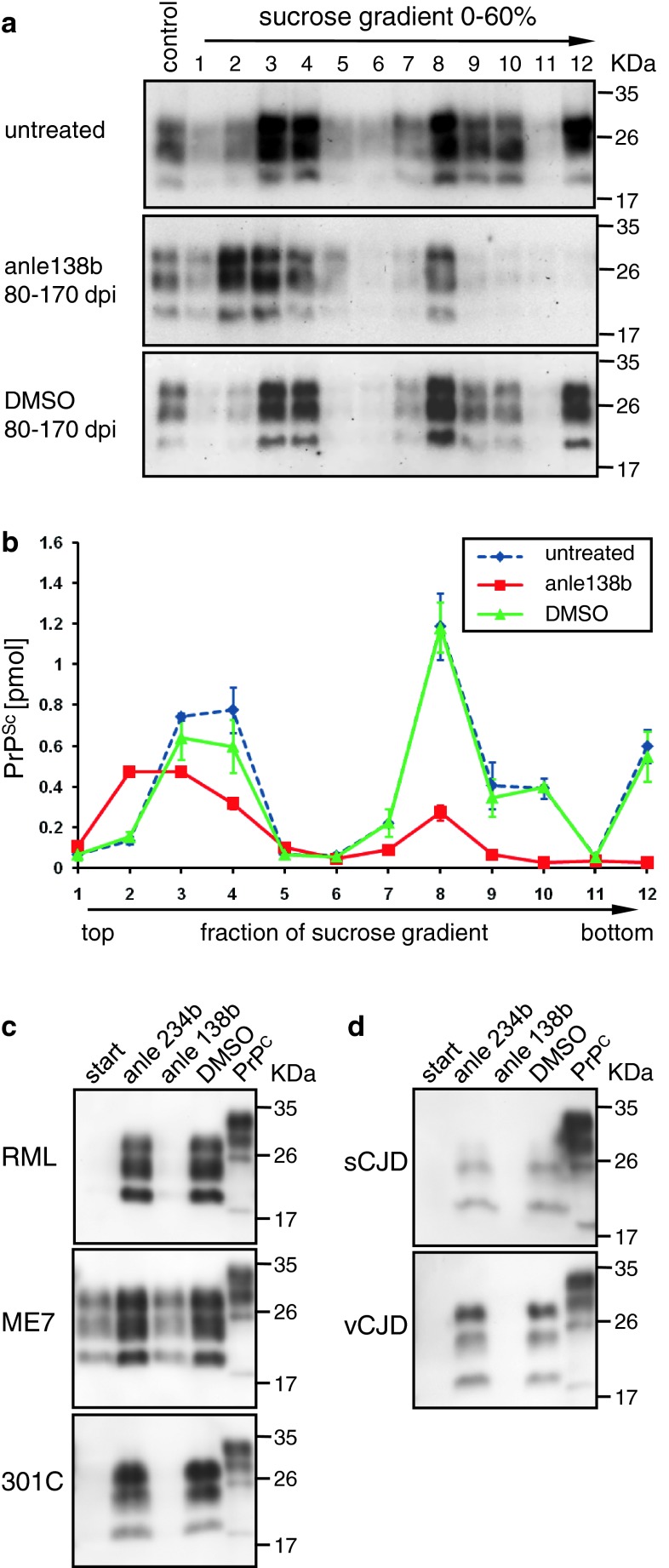
Size modulation of PrP^Sc^ oligomers by anle138b and inhibition of various human and non-human prion strains in PMCA. (**a**, **b**) Size distribution of PrP^Sc^ aggregates was analyzed by sucrose-gradient centrifugation. Mice treated with anle138b show a strong reduction of high molecular weight species (fractions 7–12). Also small molecular weight oligomers (fractions 3–4) are reduced and show a shift towards smaller size (fraction 2) indicating that anle138b blocks aggregation at the level of small oligomers. DMSO-treated mice are indistinguishable from terminally ill untreated mice. (**c**, **d**) For PMCA assay, infected brain homogenates were diluted 100-fold by appropriate normal brain homogenates containing compounds (1 μl of 10 mM solutions in DMSO) or 1 μl of DMSO as control. **c** Mouse-adapted scrapie strains (RML, ME7) and Mouse-adapted BSE (301C) were used as seed in C57/BL6 mouse normal brain homogenates. **d** sCJD and vCJD samples were propagated in non-CJD human brain homogenates. In all gels, 0.5 % (w/v) normal brain homogenates from C57/BL6 and human brain, respectively, were loaded directly in the last lane without proteolysis as a reference (indicated as PrP^C^ on the top). All other samples were treated with 50 μg/ml PK. “Start” (lane 1 of each gel) indicates samples containing infected brain homogenate before PMCA. Molecular weight markers are indicated on the *right*. PrP migrates in three different bands due to the presence of non-glycosylated, mono-glycosylated, and di-glycosylated forms, and digestion with Proteinase K results in a shift to lower molecular weights with the non-glycosylated band migrating at ~20 kD. Anle138b shows a strong inhibitory activity in all prion strains tested. As an additional control, the inactive isomer anle234b (see Table 1, Suppl. Fig. 4) was used. Quantitative PMCA results are provided in Table 1 and Suppl. Fig. 8

Regarding mode of action, these findings in combination with the finding that PrP^C^ expression is unaffected by anle138b (Suppl. Fig. 7) indicate that anle138b directly blocks PrP^Sc^ amplification and reduces neurotoxicity in vivo. Notably, we observed a significant change in size distribution of aggregates indicating a modulation of the abundance of different oligomeric species. To characterize oligomer formation in vivo, we used sucrose-gradient centrifugation, as this method, which is well established in the prion field [52], has several advantages compared to other methods for analysis of size distribution of aggregates: (i) it allows direct analysis of brain homogenates, which would be difficult by gel filtration, (ii) there is no purification step, which might modify the aggregation state, required prior to analysis, and (iii) it allows to detect and quantify all potential particle sizes (i.e. monomers, oligomers, fibrils) simultaneously. Analysis of brain homogenates by sucrose-gradient centrifugation assay reveals a strong reduction of high molecular weight species and also a shift towards smaller oligomer size for low molecular weight oligomers (Fig. 4a, b). That oligomer modulation—in addition to reduced toxicity—also interferes with PrP^Sc^ amplification is further corroborated by the fact that PrP^Sc^ amplification is also blocked in a cell-free in vitro protein misfolding cyclic amplification (PMCA) assay (Fig. 4c, d; Suppl. Fig. 8). This method provides several key advantages for compound testing. It has been shown that PMCA provides an experimental tool for rapid propagation of authentic infectious prions in vitro [67] and preserves strain properties [10]. Importantly, this approach constitutes the only experimental approach to test the efficacy of compounds in a purely human system (human prions + human brain tissue). Moreover, we found that the results obtained in the PMCA assay are predictive for the effects observed in vivo (Table 1; Fig. 2). We tested anle138b using a range of murine (RML, ME7 and 301C i.e. mouse-adapted BSE) and human strains (sCJD and vCJD). Anle138b strongly inhibited all prion strains tested including BSE-derived and human prions in a purely human system. Dose–response curves for the RML prion strain used in our animal experiments and for human vCJD were similar (EC_50_RML = 7.3 μM, EC_50_vCJD = 7.1 μM), indicating that this compound may also be useful for treatment of human prion disease (Suppl. Fig. 8).

### Pharmacokinetic analysis

**Fig. 5:**
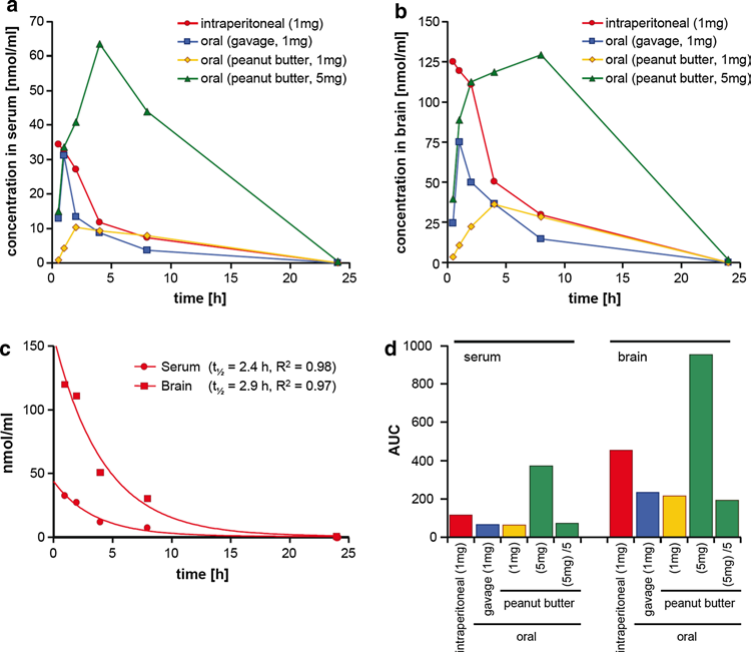
Pharmacokinetic analysis of anle138b. (**a**, **b**) A single dose of anle138b was applied to non-infected C57/BL6 mice as indicated in the figure legends. In detail, intraperitoneal application was performed using 1 mg compound in 25 μl DMSO, oral application by gavage was done with 1 mg compound in 10 μl DMSO + 40 μl vegetable oil, and oral application of 1 mg as well as 5 mg compound in peanut butter was done by providing the compound dissolved in 10 μl DMSO mixed with 200 μl peanut butter. At different time points after application (0.5, 1, 2, 4, 8, and 24 h) two animals per group and time point were sacrificed and the amount of compound in the serum (**a**) and brain (**b**) was measured by LC-MSMS. **c** From the results obtained following i.p. application, half-life of anle138b in serum and brain was calculated. **d** Calculation of AUC values. To facilitate comparison with the groups receiving a dose of 1 mg, for the group receiving 5 mg anle138b (*green columns*) we also provide the calculated AUC values per 1 mg (column: “(5 mg)/5”)

Pharmacokinetic (PK) analysis of the different application protocols used in mice modelling CJD as well as Parkinson’s disease (vide infra) revealed that anle138b has an excellent oral bioavailability and excellent blood–brain-barrier penetration, and that concentrations shown to be active in the PMCA assay were obtained in the brain (Fig. 5). Key findings in regard to PK are that (i) oral application of anle138b consistently results in approximately 50 % of the AUC (area under the curve) values compared to i.p. application, which indicates very good oral bioavailability, (ii) application in peanut butter results in slower resorption and prolonged availability of anle138b, (iii) a higher dose results in a proportional increase in serum and brain levels indicating linear PK without saturation of clearance, and (iv) at all time points in all experimental groups anle138b reaches ~3-fold higher levels in the brain than in the serum, which corroborates excellent blood–brain-barrier penetration. Thus, anle138b has a better bioavailability compared to several potential anti-aggregation drugs investigated previously, for which concentrations in the brain were found to be low [56]. Dose–response analysis for the effect of anle138b in vivo (Suppl. Fig. 9) is compatible with pharmacokinetic data and the dose–response curves obtained in vitro. In some experiments, even a reduction of PrP^Sc^ levels could be obtained with anle138b (Suppl. Fig. 10). At the same time, investigation of the brain extracts with HPLC in combination with high resolution mass spectroscopy did not identify metabolites of anle138b (Suppl. Fig. 11) in the brain, indicating that anle138b is most probably the active oligomer-modulating molecule.

### Efficacy of anle138b in synucleinopathies

**Fig. 6:**
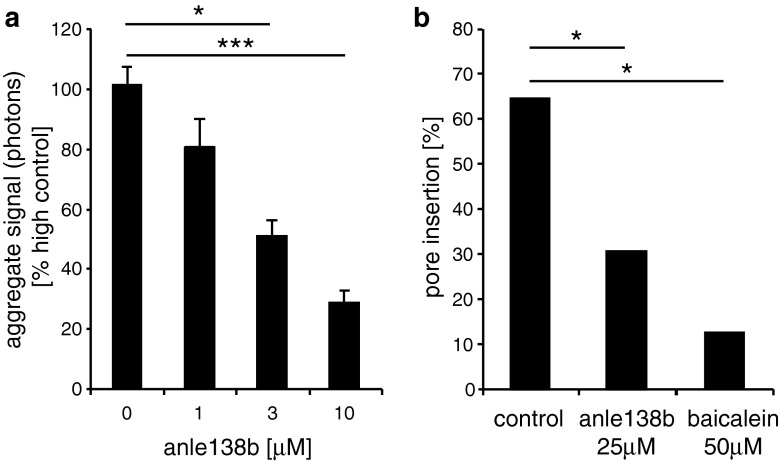
Effect of anle138b on α-syn oligomer formation. **a** Iron-induced oligomer formation of α-syn analyzed by SIFT assay [38] is blocked by anle138b in a dose-dependent manner, the apparent EC_50_ is 2.8 μM. *Error bars* indicate standard error. **b** Pore formation in lipid bilayers by α-syn oligomers is significantly reduced in presence of 25 μM anle138b. Similar results are obtained for 50 μM baicalein. Raw data for panels a and b are shown in Suppl. Fig. 13. For statistical analysis, we used student’s *t* test (**a**) and Fisher exact test (**b**). Statistical significance is indicated by *p < 0.05; **p < 0.01; ***p < 0.001

α-Synucleinopathies share molecular features in regard to aggregate structure and seeding with prion diseases [1, 29, 43] and are of high clinical importance due to their high prevalence [19]. Thus, we tested our library of DPP-derivatives with respect to effects on α-syn oligomer formation and found several active compounds (Suppl. Fig. 12), as well as several parallels with the anti-prion effect regarding SAR (Table 1). Again, at R3 the type and position of the halogen substituent was crucial with a complete block of activity with bromine in the *ortho*-position, and again pyrazol was the most active variant at R2. Anle138b turned out to inhibit α-syn oligomer formation at the same concentration range that was active in the PMCA anti-prion assay (Fig. 6a, Suppl. Fig. 13) and that was reached in brain tissue (Fig. 5). Moreover, we could show in vitro that anle138b also inhibited generation of pathological α-syn oligomers that formed ion-permeable pores in lipid membranes, a potentially neurotoxic effector species in α-syn-related neurodegenerative diseases [11, 38, 40, 55] (Fig. 6b).

**Fig. 7:**
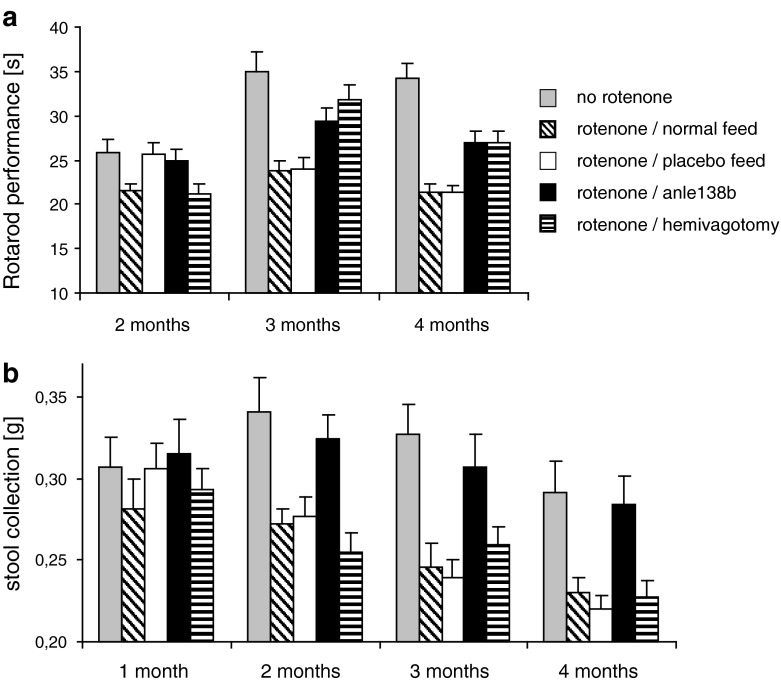
Effect of anle138b in animal models of Parkinson’s disease: low-dose intragastric rotenone model. **a** A significant decrease in motor performance (Rotarod) can be observed in rotenone-treated mice (normal feed and placebo feed) compared to the non rotenone-treated control group both after 3 (*p* < 0.01) and 4 (*p* < 0.001) months of rotenone treatment. This effect is ameliorated by treatment with anle138b. Both anle138b and hemivagotomized mice present a significantly improved motor performance after 3 (*p* < 0.01) and 4 months (*p* < 0.01) when compared to rotenone-treated (normal feed and placebo feed) mice. Interestingly, no significant differences (*p* > 0.20) could be observed between hemivagotomized or anle138b rotenone-treated mice at any time point, indicating an effect size of anle138b similar to the hemivagotomy which has recently been shown to partially block the spread of α-syn pathology from the enteric nervous system to the CNS [49]. **b** 1-h stool collection gut motility test. Decreases in stool deposition have been previously shown to correlate with the presence of α-syn pathology in the enteric nervous system as a result of rotenone treatment. Co-treatment with anle138b results in a complete rescue of enteric function. Whereas all rotenone-treated mice (including hemivagotomized mice) present significant alterations (*p* < 0.05) in stool deposition, anle138b-rotenone-treated mice show no significant difference in stool deposition when compared to controls. *Error bars* in (**a**, **b**) indicate standard error. For statistical analysis 2-way ANOVA with Bonferroni post hoc test was used

Recent evidence suggests that α-syn aggregation also plays a role in mediating toxicity of complex-I inhibitors [9, 21, 48]. In order to test the neuroprotective effect of anle138b in vivo, we used a recently established low-dose oral rotenone model that recapitulates essential features of PD pathology in humans such as α-syn accumulation and progressive spread of pathology [18, 48]. Due to the low dose and intragastric application of rotenone, the direct toxic effect of rotenone is confined to the gut without systemic complex-I inhibition in the brain and muscles, thus differing from classical rotenone models. In oral rotenone-treated mice, α-syn pathology propagates from the enteric nervous system to the CNS through synaptically connected structures, indicating that this propagation is due to transsynaptic spread and that α-syn aggregation induces dopaminergic cell death and the resulting motor phenotype. Interestingly, anle138b prevented the development of motor dysfunction in this synucleinopathy/PD model [48] to the same extent as hemivagotomy. This surgical procedure has recently been shown to partially block the spread of α-syn pathology from the enteric nervous system to the CNS [49] (Fig. 7a). Moreover, treatment with anle138b almost completely rescued the phenotype in the 1-h stool collection gut motility test (Fig. 7b), which has previously been shown to correlate with the presence of α-syn pathology in the enteric nervous system in this model [49].

**Fig. 8:**
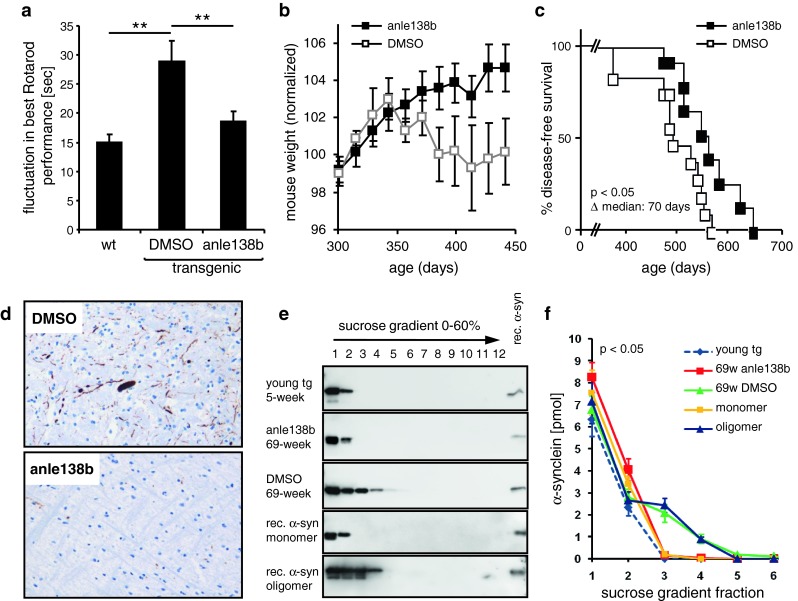
Effect of anle138b in animal models of Parkinson’s disease: A30P-hum-α-syn transgenic mice. Effect of treatment with anle138b in a transgenic mouse Parkinson model based on neuronal overexpression of A30P-hum-α-syn [47]. **a** In the prodromal phase of the disease (300–450 days of age), placebo-treated transgenic animals exhibit significantly increased fluctuations in rotarod performance. This phenotype can be rescued by treatment with anle138b. **b** In this time span, nearly normal development of body weight can be observed in the anle138b treated group, whereas the placebo-treated group shows reduced gain of weight. Mouse weights were normalized to the mean weights obtained at measurements between 250 and 315 days of age. **c** Kaplan–Meier evaluation of treatment with anle138b shows a significant increase in disease-free survival. The median disease-free survival is prolonged by 10 weeks. **d** Representative sections from the brain stem show a reduction of pathological α-syn deposits in 69-week-old anle138-treated mice in comparison to placebo-treated age- and sex-matched littermates (see also Suppl. Fig. 15). (**e**, **f**) Sucrose-gradient centrifugation shows that in young transgenic mice α-syn is found in the same fractions (1–2) as monomeric recombinant α-syn. In 69-week-old placebo-treated mice, α-syn oligomers can be found that show the same size distribution as oligomers derived from recombinant α-syn by treatment with DMSO/Fe^3+^. Oligomer formation in transgenic mice is inhibited by treatment with anle138b. For the corresponding graph shown in (**f**), four mice per group were analyzed (Suppl. Fig. 16). *Error bars* in (**a**, **b**, **f**) indicate standard error. For statistical analysis, we used student’s *t* test, Statistical significance is indicated by **p < 0.01 (**a**), log-rank test (Mantel-Cox) and Gehan-Breslow-Wilcoxon test (**c**), and ANOVA and multiple comparison post-test (**f**)

In a further set of in vivo experiments, treatment with anle138b also blocked dopaminergic cell death in the substantia nigra in a sub-acute MPTP mouse model for PD (Suppl. Fig. 14). However, the details of the interaction between mitochondrial toxins, progression of pathology, and α-syn aggregation are not fully established. Thus, we also tested the effect of anle138b in a well-established long-term transgenic α-synucleinopathy mouse model based on neuronal expression of human A30P-α-syn [47] (Fig. 8). In placebo-treated transgenic mice, pathological deposition of α-syn in the brain was first detected in the brain stem at the end of the first year of life, increased with time and spread to supratentorial areas. Signs of disease developed in parallel to α-syn deposition. During a prodromal phase (approximately 300–450 days of age), rotarod measurements revealed increased fluctuations in motor performance (Fig. 8a), which are a typical feature of human synucleinopathies [61]. In addition, reduced gain of weight was observed (Fig. 8b). This was followed by a clinical phase characterized by a rapid decline in rotarod performance. Anle138b significantly reduced fluctuations in motor performance approaching the level of non-transgenic control mice (Fig. 8a) and lead to an almost normal increase in body weight as observed for wt mice (Fig. 8b). Disease-free survival was prolonged significantly (*p* < 0.05) by ~10 weeks (Fig. 8c). In anle138b-treated mice, we observed that pathological deposition of α-syn in the brain was reduced (Fig. 8d; Suppl. Fig. 15). Similar to the prion mouse model, we found a strong reduction of pathological α-syn oligomers in the anle138b-treated mice (Fig. 8e, f; Suppl. Fig. 16). The level of total α-syn was unchanged in anle138b-treated mice (Suppl. Fig. 17), indicating that anle138b does not interfere with expression and degradation of α-syn in these mice but acts as an aggregation inhibitor. Notably, no toxic effect of anle138b was observed in our in vivo experiments even after long-term high-dose treatment.

In preparation for a first clinical trial, preclinical assays for acute toxicity and mutagenicity yielded promising results (Suppl. Fig. 18). However, further preclinical safety studies will be required prior to clinical studies in humans. Anle138b does not interfere with the formation of physiological fibrillar protein assemblies that are structurally different from amyloid such as actin as shown by in vitro experiments (Suppl. Fig. 19). Such an interaction was not expected given the treatment of mice for more than a year without any sign of toxicity.

### Binding studies using intrinsic fluorescence of anle138b

**Fig. 9:**
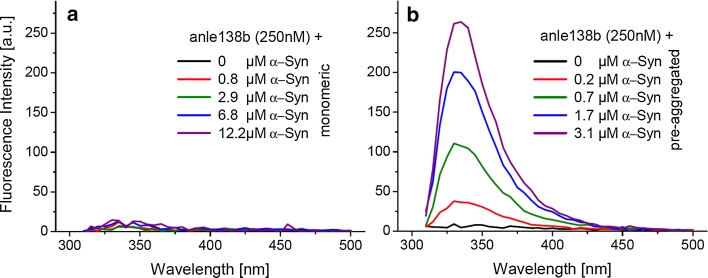
Structure-dependent binding of anle138b. Intrinsic Fluorescence of 250nM anle138b was measured with excitation at 300 nm. **a** Addition of α-syn monomers does not significantly change the weak fluorescence of anle138b. **b** Addition of pre-aggregated α-syn results in a dramatic change in the fluorescence properties with a strong increase of fluorescence at ~340 nm by more than a factor of 30. This points to specific binding of anle138b to a structure-dependent binding site

Based on the molecular structure of anle138b, we considered the possibility that this compound has an intrinsic fluorescence that might be employed for direct molecular binding studies. We found that in aqueous solution with excitation at 300 nm, anle138b has an extinction coefficient of 10,000 (Mcm)^−1^ but exhibits a very weak intrinsic fluorescence (Fig. 9). The fluorescence properties of anle138b do not change upon addition of monomeric α-syn. However, one observes a strong increase in the fluorescence intensity of anle138b by more than a factor of 30 when pre-aggregated α-syn was added, indicating strong structure-dependent binding of anle138b to aggregated α-syn even at nanomolar concentrations (Fig. 9b).

## Discussion

Regarding treatment of neurodegenerative diseases in humans, DPP-derivatives and specifically anle138b represent a new lead structure with several favourable features. They are small molecules that have an excellent oral bioavailability and blood–brain-barrier penetration without signs of toxicity at therapeutic doses. To the best of our knowledge, the prolongation of survival even in late-stage treatment experiments obtained in prion-infected mice is by far the largest that has been found for any drug-like compound tested so far (Suppl. Fig. 20). We found that anle138b has several beneficial properties compared to previously identified molecules such as compound B by Kawasaki et al. [36]: (i) the effect on survival is larger, (ii) anle138b has better pharmacokinetic properties (e.g. a longer half-life), and (iii) compound B was reported to lack activity for certain prion strains and—at the biochemical level—to be less efficient for double glycosylated PrP^Sc^ species, whereas we show that anle138b is active in all strains tested (including human CJD and BSE-derived strains) and that there is no lack of activity for double glycosylated PrP^Sc^. We did not observe a selection and over-representation of this PrP^Sc^ species in treated animals (Fig. 4a). Several studies aiming at the development of new therapeutic anti-prion compounds showed anti-prion activity only in cell culture but either lacked data from animal models or were found to be inefficient in vivo [23, 26, 60]. For example, quinacrine was reported to exhibit a high anti-prion activity in cell culture [37]. However, in vivo studies in mice and humans were unsuccessful [14] as this compound does not reach sufficient concentrations in the brain [24], which underlines the importance of systematic and timely in vivo testing to optimize therapeutic efficacy of synthesized compounds not only in vitro but in vivo.

Regarding the mode of action of anle138b, we present comprehensive evidence, that it is based on direct modulation of aggregation at the oligomer level. At the molecular level, we demonstrate that anle138b blocks the formation of toxic pore-forming α-syn oligomers (Fig. 6) for which we previously provided a detailed morphological and structural characterization based on single-particle spectroscopy and atomic force microscopy [38], as well as single-channel electrophysiology [55]. It has been shown that these globular oligomers are able to interact with lipid membranes, form pores, modulate electrophysiological properties of neuronal cells and cause toxicity in cell culture [32, 33, 38, 55]. In vivo, we see a diversion of formation of disease-associated oligomers to smaller oligomers and monomers by anle138b treatment for both PrP^Sc^ and α-syn (Figs. 4a, b; 8e, f). Importantly, anle138b does not lower the level of normal PrP^C^ and α-syn (Suppl. Fig. 7; Suppl. Fig. 17), which indicates that this compound directly interferes with pathological aggregation. This is further corroborated by the anti-aggregative activity of anle138b in vitro both in regard to α-syn aggregation and in regard to prion propagation in cell-free assays. Notably, the anti-aggregative activity in vitro and the reduction of aggregated species in vivo correlate well with the therapeutic effect in vivo for all compounds tested (Fig. 2). In addition, we show a direct and structure-dependent binding, i.e. a direct targeting of pathological aggregates by anle138b, based on a dramatic change in intrinsic fluorescence, whereas there is no indication for binding to monomers (Fig. 9).

Thus, our findings indicate that the therapeutic effect in vivo is due to direct targeting of pathological aggregation at the level of oligomers and not to indirect effects such as reduced protein expression or increased clearance. However, the identification of residues with which anle138b interacts on α-syn and prion protein oligomers is so far unsolved. This is due to fundamental experimental challenges which are:

1. Unlike some other compounds, anle138b does not bind to the monomer (Fig. 9). From a therapeutic view this is a major advantage, as anle138b is therefore unlikely to interfere with the physiological functions of the non-aggregated protein. For monomer binders such as PcTS, NMR was successful in localizing the residues where the interaction occurs [39]. Since oligomers are a very mobile and dynamic assembly of monomers which are partially unstructured, NMR-based approaches failed so far to identify the residues which interact with small molecule inhibitors such as anle138b.
2. Anle138b is soluble in water only in concentrations below 1 μM precluding high-resolution NMR spectroscopy of complexes of anle138b with soluble oligomers.
3. As anle138b binds to a structure-dependent epitope, it may well be that the compound interacts with several residues, which would require a full structure determination of the oligomer to characterize the binding epitope. However, atomic resolution oligomer structures are not available neither in solution nor in the membrane. Fibrils are much more structured and this allowed for the only work available so far, namely the residue-specific atomic resolution structure of congo-red with het-S fibrils. Unlike oligomers, these fibrils are very well structured and stable [66].

The fact that the atomic resolution structure of prions, pathological oligomers, and amyloid fibrils remains to be elucidated constitutes a major challenge for drug development for neurodegenerative diseases and may be one key reason that there are no drugs available for treatment of human patients so far. Therefore, some groups work on compounds that bind to and stabilize the conformation of the physiological conformation of aggregation-prone proteins. This approach has been successful in the case of the target transthyretin [16] which is a folded protein with known atomic resolution structure. The mode of action of these compounds is fundamentally different from our approach, which targets the aggregated state. Importantly, inhibition of the aggregation of intrinsically unfolded proteins like in the case of α-syn cannot use this strategy. Thus, we employed an innovative approach to drug development. We performed molecular screening using SIFT, a novel method based on single-particle spectroscopy that allows targeting oligomer formation. Due to the low concentration of α-syn used in this assay, solubility requirements for the compounds did not bias dramatically against lipophilic compounds. This method was a key success factor for finding anle138b, since it represents the first described way to target intrinsically disordered proteins. Moreover, the use of prion-infected mice allowed efficient testing of a large number of compounds in vivo in an authentic disease model and, thus, to optimize compounds in regard to in vivo efficacy while balancing the in vitro efficacy in an unprecedented way.

In summary, our experiments show that targeting protein oligomers provides a potent disease-modifying therapy in vivo for both prion diseases and PD, and corroborate the key role of protein oligomers in disease pathogenesis. In addition, anle138b provides a new tool to further dissect the role of protein aggregates during disease pathogenesis in vivo. The finding that DPP compounds modulate the formation of pathological oligomers of both prion and α-syn and inhibit disease progression with obvious parallels regarding SAR fits well with recent evidence that pathological aggregates in these diseases share common structural features and have similar properties with respect to binding of specific structure-sensitive antibodies and certain chemical dyes such as congo-red and thioflavinT [29, 58]. This suggests that common basic principles regarding the structure of toxic aggregates are involved in these diseases and amenable to pharmacological inhibition in vivo.

So far, no drug using this molecular mode of action has entered clinical practice. Current therapies for neurodegenerative diseases such as Alzheimer’s disease, Parkinson’s disease and prion diseases target disease symptoms [20]. Here, we show that compounds targeting oligomer formation can have a broad spectrum of activity in the treatment of different protein aggregation diseases. Therefore, we currently extend our experiments with anle138b to Abeta, tau, SOD1 and TDP43 aggregation important for AD, frontotemporal dementia (FTD), and amyotrophic lateral sclerosis (ALS).

## Electronic supplementary material

Below is the link to the electronic supplementary material.

Supplementary material 1 (PDF 3337 kb)

Supplementary material 2 (PDF 4030 kb)
