# Supplementary material for: Anle138b: a novel oligomer modulator for disease-modifying therapy of neurodegenerative diseases such as prion and Parkinson’s disease
Source: Acta Neuropathol. 2013 Apr 19;125(6):795–813. doi: 10.1007/s00401-013-1114-9 (PMC3661926; doi:10.1007/s00401-013-1114-9)
Supplement: Supplementary file 1 — Supplementary material 1 (PDF 3337 kb) [file 401_2013_1114_MOESM1_ESM.pdf]

Supplement Figures 1-20

Anle138b: a novel oligomer modulator for disease-modifying therapy of neurodegenerative diseases such as prion and Parkinson´s disease.

Jens Wagner[1,#], Sergey Ryazanov[2,3,#], Andrei Leonov[2,3,#], Johannes Levin[4,#], Song Shi[1,#], Felix Schmidt[1,4], Catharina Prix[1], Francisco Pan-Montojo[5], Uwe Bertsch[1,14], Gerda Mitteregger-Kretzschmar[1], Markus Geissen[6,15], Martin Eiden[6], Fabienne Leidel[6], Thomas Hirschberger[7], Andreas A. Deeg[7], Julian J. Krauth[7], Wolfgang Zinth[7], Paul Tavan[7], Jens Pilger[2,3], Markus Zweckstetter[2,3,8], Tobias Frank[3,9], Mathias Bähr[3,9], Jochen Weishaupt[3,9], Manfred Uhr[10], Henning Urlaub[11], Ulrike Teichmann[12], Matthias Samwer[13], Kai Bötzel[4], Martin Groschup[6], Hans Kretzschmar[1], Christian Griesinger[2,3,*], Armin Giese[1,*]

Supplement Figure 1:

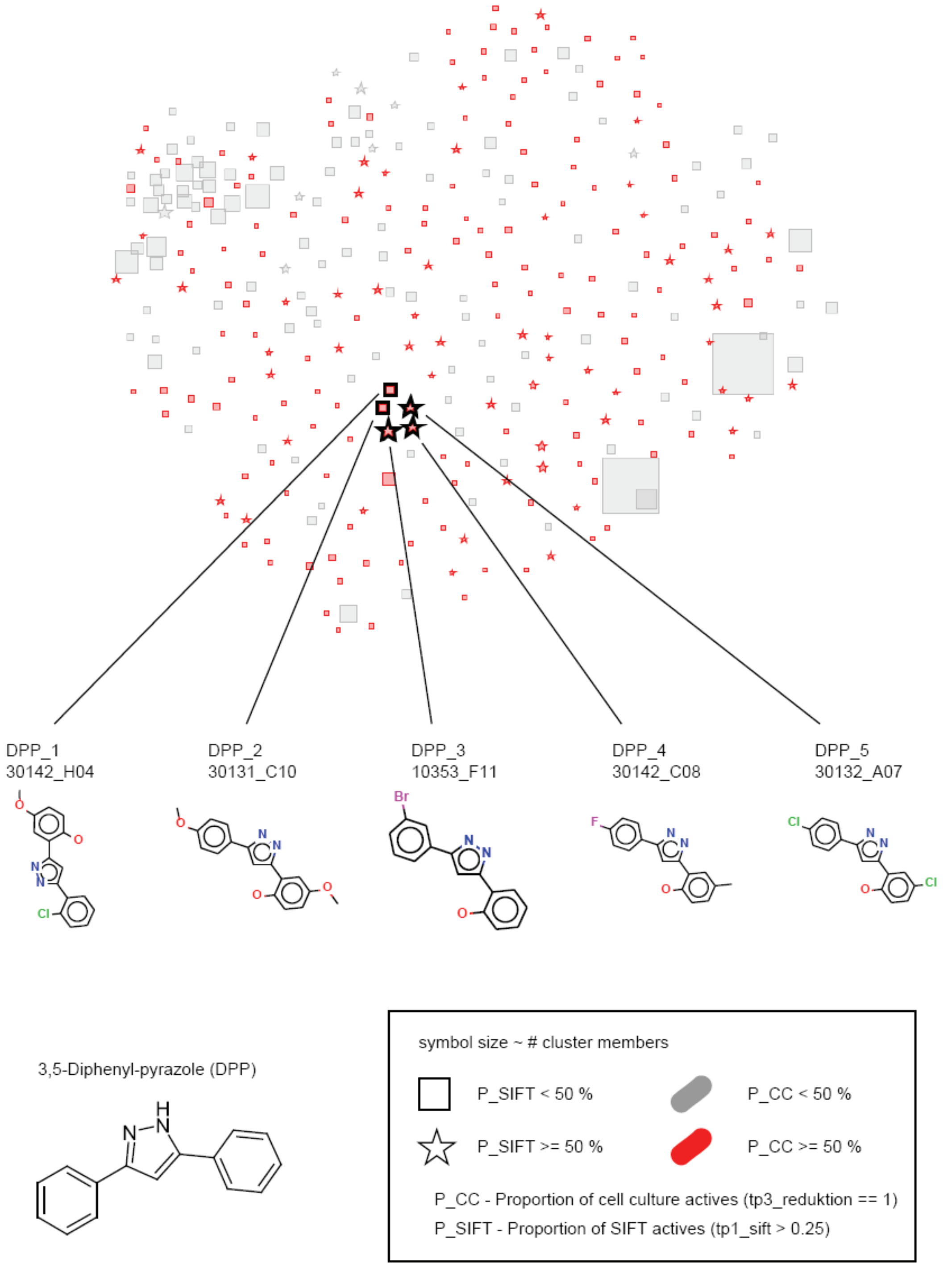

Structure-Actvity-Relationships (SAR)-Map generated for substances of the libraries screened for anti-prion activity using the SIFT and cell culture assays.

The SAR-Map shows clusters of structurally similar compounds (represented by stars or boxes) built from the 837 hit compounds of the primary cell culture screening campaign of the substance libraries DIVERSet 1 and 2. The symbols representing the clusters are arranged such that similar clusters are close to each other, and the symbols are scaled, shaped and coloured according to the sizes of the clusters and the proportions of SIFT and cell culture actives, respectively, as explained in detail below. Thus large clusters, containing large proportions of SIFT and cell culture actives are symbolized by large red stars. Five clusters, termed DPP_1 through DPP_5 are selected and prototypical compounds representing these clusters are displayed.

For the generation of the SAR-map substances from the DIVERSet libraries were subjected to a cluster analysis using the software package Benchware HTS DataMiner (DM; Tripos Inc., St. Louis, MO, USA). Since the set of all (20,000) compounds from the libraries would have been too large as a starting set for cluster formation using DM, the initial set of compounds was restricted to the set of primary hits (837 compounds) from the cell culture screening. Thus, clusters were built based on the active compounds only. Here, the DM program grouped structurally similar compounds into clusters thereby enabling the identification of potentially relevant new lead structures. In a second step, the thus established classification was applied to the rest of the library covering the compounds inactive in cell culture. Here, the DM program added the remaining (inactive) compounds to the generated clusters, if the employed measure indicated a high structural similarity.

The result of the cluster analysis is displayed by DataMiner as a SAR-map, in which the substance clusters S are represented by symbols arranged in proximity according to structural similarity. The sizes, forms and colours of the symbols were allocated based on cluster-specific properties. The sizes of the symbols were selected proportional to the sizes $|S|$ of the clusters, i.e. to the number of compounds C contained. The forms of the symbols were determined based on the cluster-local proportions

$$P_{\mathrm{SIFT}}(S) = |\{\mathrm{C} \in S \mid a(C) \geq a_{\min}\}| \,/\, |S|$$

of those compounds C in the respective clusters S, whose primary activity $a(C)$ determined in the SIFT screening was above a selected threshold of $a_{\min} = 0.25$. Based on this, clusters whose proportion $P_{\mathrm{SIFT}}(S)$ is above 50% are shown as stars, whereas the remaining clusters are shown as boxes. Analogously, the colours of the symbols encode the cluster-local proportions

$$P_{\mathrm{CC}}(S) = |\{\mathrm{C} \in S \mid \textit{is primary hit}\}| \,/\, |S|$$

of primary hits from the cell culture (CC) screening, whereupon the symbols of clusters with more than 50% active substances are coloured red and those of the remaining clusters are coloured grey.

Hence, as pointed out, in the resulting SAR-map, large red stars symbolise clusters with a high proportions of SIFT and cell culture positive substances. Such clusters represent potential lead structures. Using DataMiner, clusters of interest were further analysed and a group of five neighbouring clusters was identified and termed DPP_1 through DPP_5 (shown in bold). The fact that these clusters are located close to each other indicates that they contain structurally similar compounds. In fact, all of them belong to the chemical compound class of 3,5-Di-Phenyl-Pyrazole (DPP) derivatives.

Supplement Figure 2:

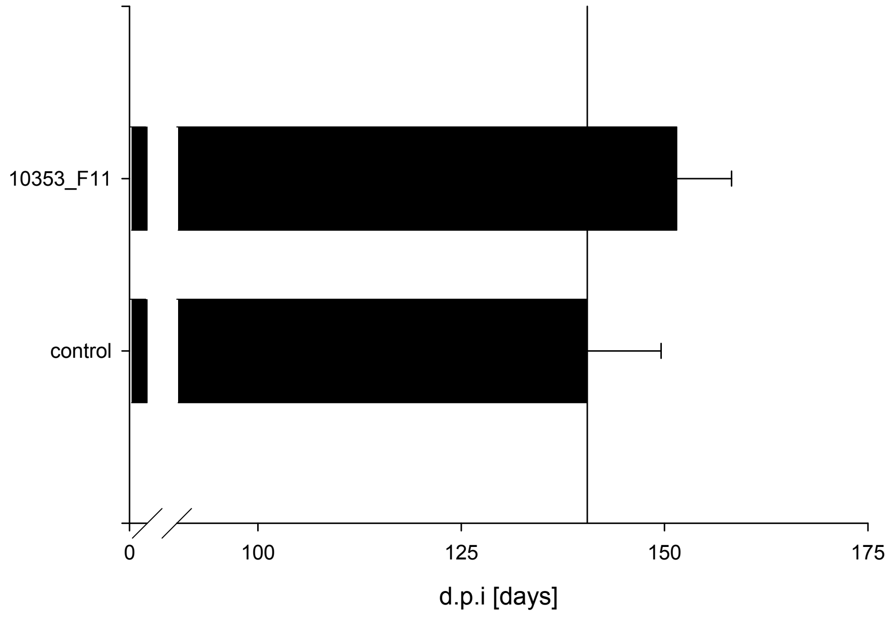

Effects of treatment with DPP compound 10353_F11 on survival time of mice after intracerebral infection with RML scrapie.

Treatment with compound 10353_F11 (chemical structure: see Suppl.-Fig.1) prolonged mean survival of mice infected intracerebrally with prion strain RML by eleven days (n=8, $p < 0.05$). The compound was administrated daily for 14 days from day 80 post infection (50 µl i.p., 10 mM compound in DMSO). Mean survival times are expressed in days + standard deviation.

Supplement Figure 3:

DPP-derivatives tested for anti-prion activity *in vivo*

No.	compound	structure	% inhibition[#]	Δ survival[+]
1	Anle138b	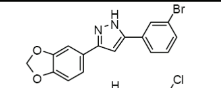	78[a], 57[b], 108[c], 70[d]	15/14[f], 22/24[g], 51/53[i], 74/88[j], 76/82[k], 198/179[l]
2	Sery335b	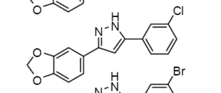	68[a], 46[b]	
3	Anle253b	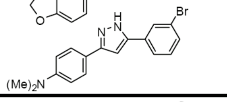	59[a]	
4	Sery149	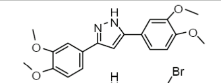	<10[b]	12/14[f]
5	10353F11	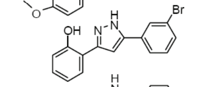		11/14[f]
6	Sery378b	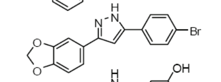	39[a]	
7	Sery339b	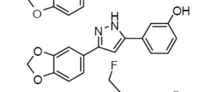	37[b]	
8	Sery363a	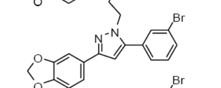	35[a]	
9	Sery338b	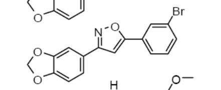	35[b]	
10	Anle233b	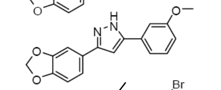	33[a]	
11	Sery392b	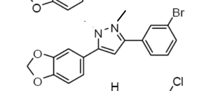	33[a]	
12	Sery383	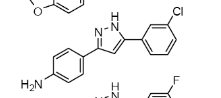	31[a]	
13	Anle186b	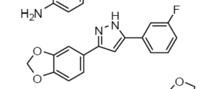	30[c]	4/24[g]
14	Anle237	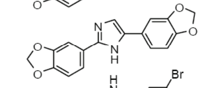	26[a]	
15	Sery255b	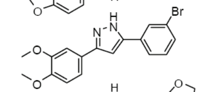	25[a], <10[d]	
16	Anle232b	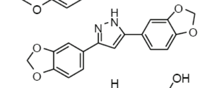	23[a]	
17	Sery344	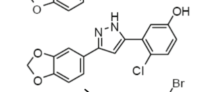	21[b]	
18	Sery392a	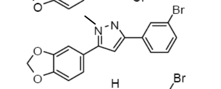	14[a]	
19	Sery312b	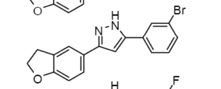	13[a]	
20	Sery85	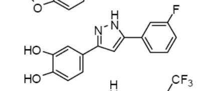	13[a]	
21	Anle236b	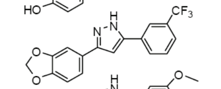	12[a]	
22	Anle143b	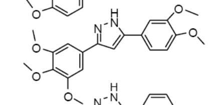		3/14[f]
23	Sery106	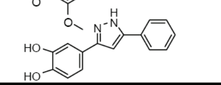		3/14[f]

No.	compound	structure	% inhibition#	Δ survival+
24	Sery166a	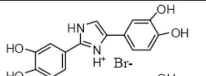	<10[e]	2/85[h]
25	Sery158b	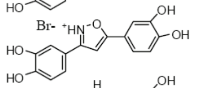	<10[e]	
26	Sery159a	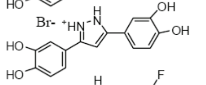	<10[e]	
27	Sery294b	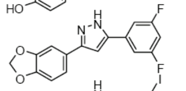	<10[b]	12/14[f]
28	Anle197b	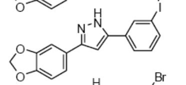	<10[b]	11/14[f]
29	Anle138c	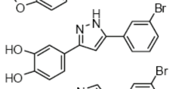	<10[a]	
30	Sery345	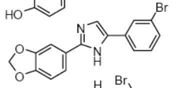	<10[a]	
31	Anle234b	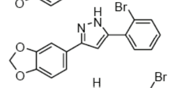	<10[a]	
32	Sery384	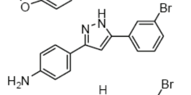	<10[a]	
33	Sery401b	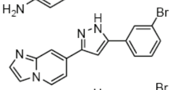	<10[a]	
34	Anle270	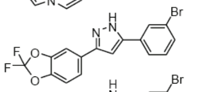	<10[a]	
35	Sery417	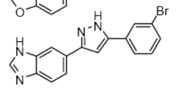	<10[a]	
36	Sery363b	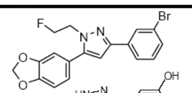	n/a*	
37	Anle143c	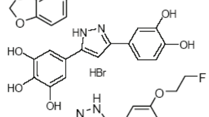		toxic
38	Sery369	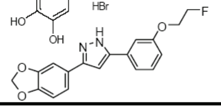	toxic	

The table summarizes the effect of various compounds in regard to inhibition of prion propagation and prolongation of survival time *in vivo*.

# relative inhibition of PrP^{Sc} accumulation normalized to DMSO-treated group (0% inhibition) and PrP^{Sc} level at start of treatment (100% inhibition).

+ Δ survival (prolongation of survival in days/treatment days up to mean survival of controls)

* mice refused to eat peanut butter pellets

[a] PrP^{Sc} level in brain 120 days after i.c. infection and treatment for 40 days with 1 mg compound (oral, in peanut butter).

[b] PrP^{Sc} level in spleen determined 35 days after i.p. infection followed by 34 days of treatment with 1 mg compound (oral, in peanut butter).

[c] PrP^{Sc} level in brain at 106 days after i.c. infection and treatment for 24 days (14 days i.p. (0.84 mg compound); 2 x 5 days oral by gavage (1 mg)).

[d] PrP^{Sc} level in spleen at 35 days after i.p. infection and treatment for 24 days (14 days i.p. (0.84 mg compound); 2 x 5 days oral by gavage (1 mg)).

[e] PrP^{Sc} level in spleen at 35 days after i.p. infection and treatment for 14 days (14 days i.p. (50 µl 100 µM compound).

[f] Prolongation of survival time (days) after i.p. infection and treatment for 14 days starting at 80 dpi (14 days i.p. (50 µl 100 µM compound).

[g] Prolongation of survival time (days) after i.c. infection and treatment for 24 days starting at 80 dpi (14 days i.p. (0.84 mg compound); 2 x 5 days oral by gavage (1 mg)).

[h] Prolongation of survival time (days) after i.c. infection and application of the compounds via an osmotic pump that was implanted at 80 dpi (60 nmol compound/d).

[i] Prolongation of survival time (days) after i.c. infection and daily treatment starting at 120 dpi with 5 mg compound (oral, in peanut butter) until terminal disease.

[j] Prolongation of survival time (days) after i.c. infection and daily treatment starting at 80 dpi with 5 mg compound (oral, in peanut butter) until terminal disease.

[k] Prolongation of survival time (days) after i.c. infection and 2 x daily treatment starting at 80 dpi with 5 mg compound (oral, in peanut butter) until terminal disease.

[l] Prolongation of survival time (days) after i.c. infection and 2 x daily treatment starting at 0 dpi with 5 mg compound (oral, in peanut butter) until terminal disease.

Supplement Figure 4:

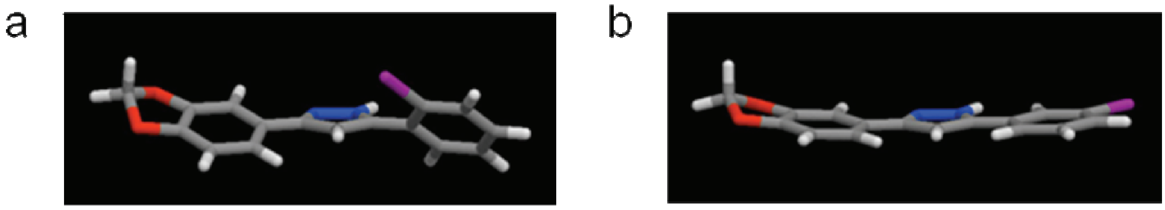

Models of likely 3-dimensional structures of compounds anle234b (**a**) and anle138b (**b**). Substitution of the bromine in the *ortho*-position (anle234b) abolished the inhibitory activity of anle138b which is substituted in the *meta*-position. The substitution tilts the phenyl ring so that the molecule is no longer planar indicating that a planar conformation is necessary for activity of inhibitory compounds. The 3-D structures were generated with the Molinspiration Galaxy 3D Structure Generator (www.molinspiration.com).

Supplement Figure 5:

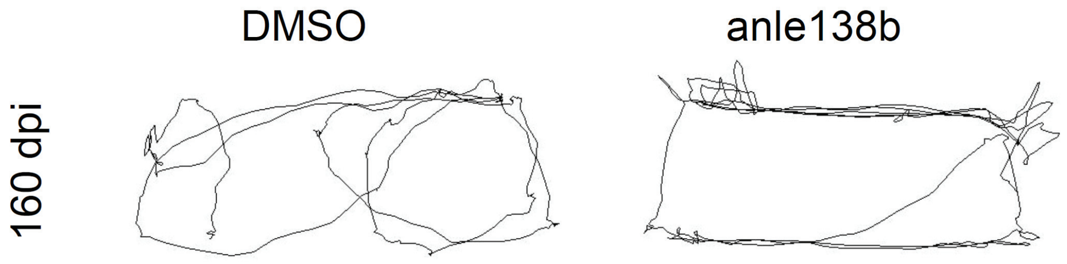

Motor performance in prion infected mice at 160 d.p.i. is unimpaired in mice treated with anle138b. Mice were placed in an arena (265 x 150 x 420 mm). Movements were recorded with a monitoring system. Movements of the animals were tracked and analyzed with ImageJ 1.39. Representative paths for a DMSO-treated (left) or anle138b-treated (right) mouse at 160 days after intracerebral infection are shown.

Supplement Figure 6:

Statistical evaluation of survival data shown in Fig. 3a

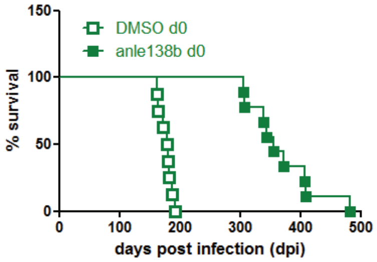

Comparison of Survival Curves

**Log-rank (Mantel-Cox) Test**	
Chi square	18,83
P value	< 0.0001
**Gehan-Breslow-Wilcoxon Test**	
Chi square	16,00
P value	< 0.0001
**Median survival**	
DMSO d0	179,5
anle138b d0	355,0
**Hazard Ratio**	
Ratio	30,75
95% CI of ratio	6.542 to 144.5

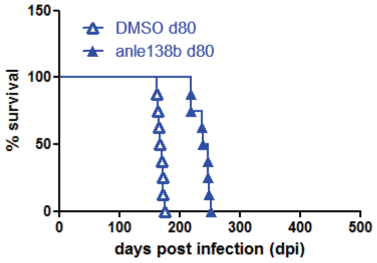

Comparison of Survival Curves

**Log-rank (Mantel-Cox) Test**	
Chi square	16,94
P value	< 0.0001
**Gehan-Breslow-Wilcoxon Test**	
Chi square	14,22
P value	0,0002
**Median survival**	
DMSO d80	168,5
anle138b d80	242,5
**Hazard Ratio**	
Ratio	24,41
95% CI of ratio	5.331 to 111.8

Supplement Figure 6 (cont.):

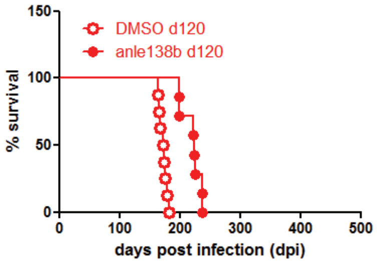

Comparison of Survival Curves

Log-rank (Mantel-Cox) Test

Chi square	15,04
P value	0,0001

Gehan-Breslow-Wilcoxon Test

Chi square	12,44
P value	0,0004

Median survival

DMSO d120	172,0
anle138b d120	224,0

Hazard Ratio

Ratio	19,34
95% CI of ratio	4.328 to 86.43

Supplement Figure 7:

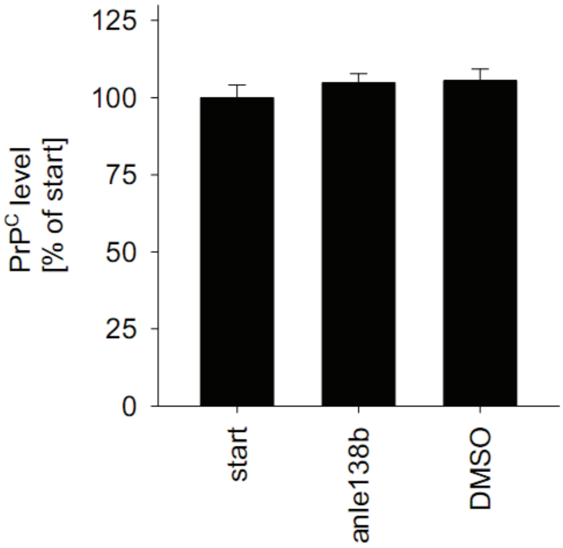

Quantification of PrP^C by immunoblotting of brain tissue from non-infected mice treated with anle138b (1 mg per day in DMSO/peanut butter) for 1 week.
No reduction in PrP^C level was observed in mice treated with anle138b when compared to control mice that received DMSO/peanut butter without anle138b. Error bars indicate standard error (n = 4 mice).

Supplement Figure 8:

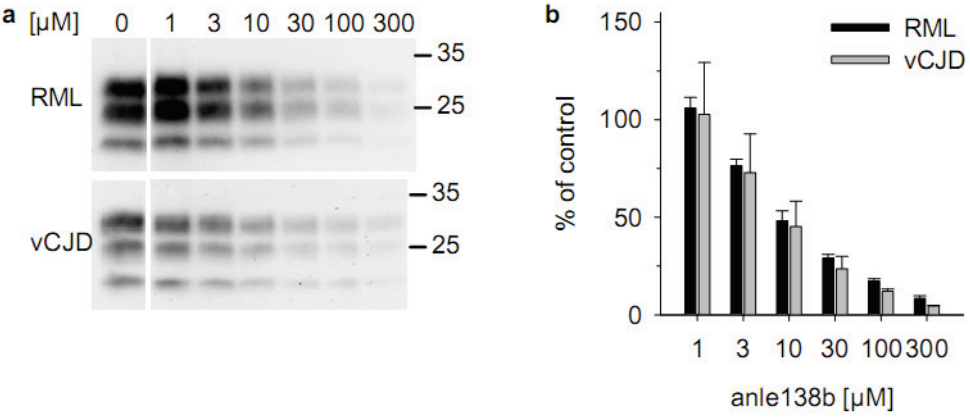

Inhibition of *in vitro* propagation of different prion strains by anle138b.

(**A**) Normal brain homogenates of C57BL/6 mouse and human seeded with a 100-fold dilution of infected brain homogenates respectively were mixed with different concentration of anle138b (0, 1, 3, 10, 30, 100 and 300 µM, final concentration). PMCA reactions were conducted 18 cycles for mouse substrate and 40 cycles for human substrate. The effect of anle138b was dose-dependent. Molecular weight markers are indicated on the right in kD. (**B**) The amount of PrP^{Sc} was quantified densitometrically and normalized to the control reaction without compound. Similar dose-response curves were obtained for human prions (vCJD) and for the murine prion strain RML that was used in animal experiments. The EC_{50} values for anle138b in the PMCA assay are 7.3 µM for RML prions and 7.1 µM for vCJD prions, respectively. Three independent experiments were performed. Results are presented as mean ± standard error.

Supplement Figure 9:

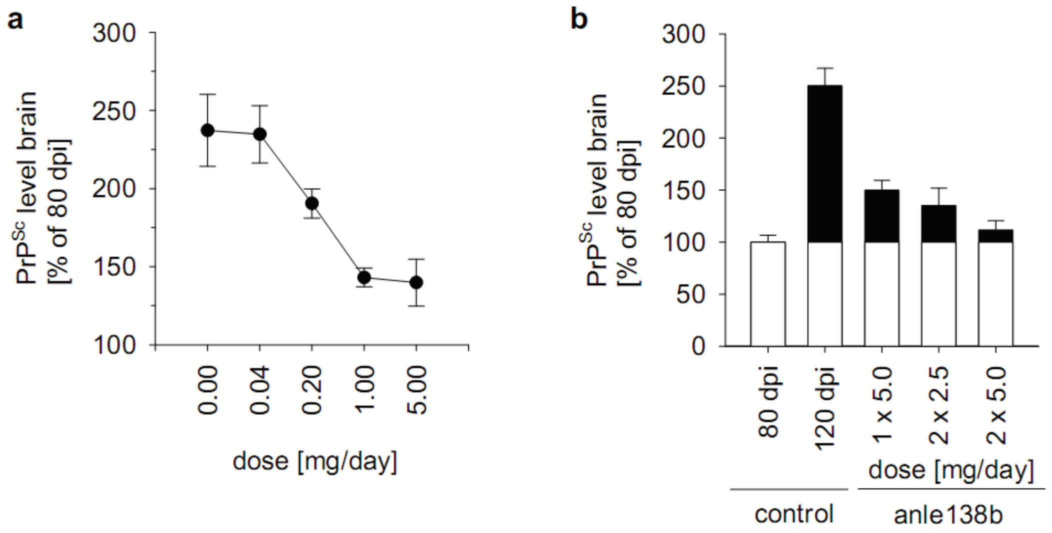

Dose-dependent effect of anle138b administration on PrP^{Sc} levels in brain.
(**A**) C57/BL6 mice were inoculated intracerebrally with 30 µl of 1% brain homogenate (RML scrapie). Treatment was started at 80 days post infection with different amounts of anle138b applied orally mixed with DMSO/peanut butter. At 120 days post infection, animals were sacrificed and the amount of PrP^{Sc} in the brain was quantified in comparison to animals sacrificed at day 80 post infection. Treatment with anle138b reduced PrP^{Sc} accumulation in brain in a dose-dependent manner with an EC_{50} of 0.21 mg/day. (**B**) In an independent experiment, different treatment schedules were compared using the same experimental approach as described in (**A**). Application of anle138b twice daily appears to be more efficient than one single dose/day. Error bars indicate standard error (n = 4 mice).

Supplement Figure 10:

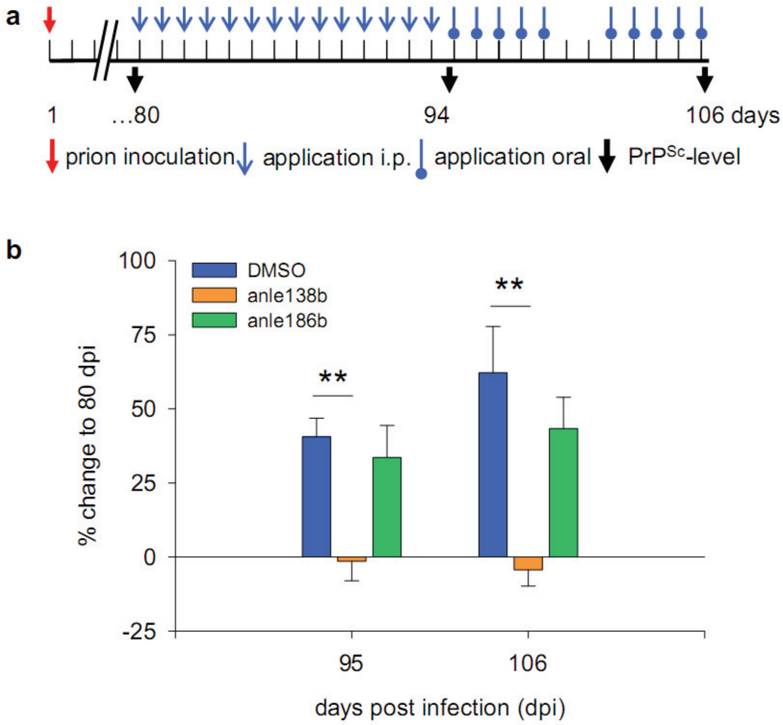

Mixed application scheme resulting in a decrease of PrP^{Sc} levels in anle138b-treated mice.
(**A**) Experimental protocol: Seven-week-old female C57/BL6 mice were inoculated intracerebrally with 30 µl of 1% brain homogenate (RML scrapie). Treatment was started at 80 days post infection with 0.84 mg compound (in 25 µl DMSO) per day applied by intraperitoneal injection for 14 days followed by 2 x 5 days of 1 mg compound (in 10 µl DMSO + 40 µl vegetable oil) applied orally by gavage. PrP^{Sc} level in brain was measured before treatment and at 95 and 106 days post infection. (**B**) Change of PrP^{Sc} levels after treatment with compounds anle138b and anle186b in comparison to controls. Treatment with anle186b leads to a slight reduction of PrP^{Sc} accumulation. In the anle138b-treated group, a decrease in PrP^{Sc} levels can be observed indicating a virtually complete block of prion amplification. Error bars indicate standard error ($n = 4$; ** = $p < 0.01$).

Supplement Figure 11:

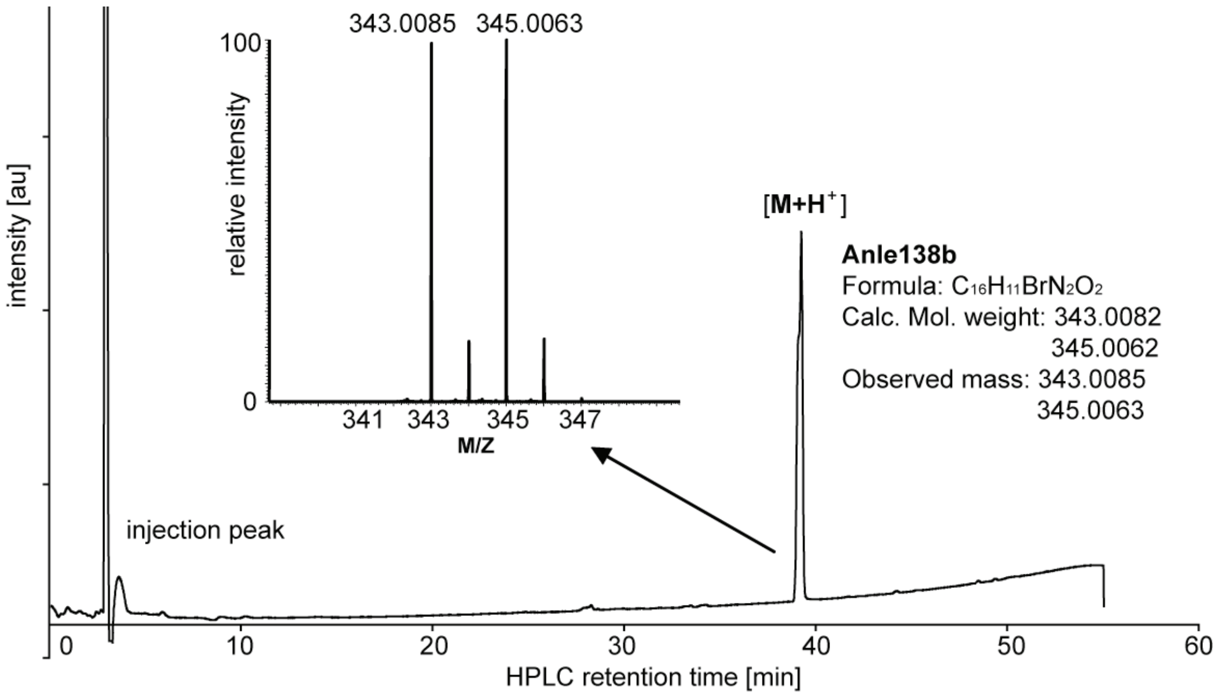

HPLC chromatogram of mouse brain homogenate for quantification of anle138b. The high resolution mass spectrum (ESI$^+$ mode) identifies the present compound clearly as anle138b from the mass and the two equally populated isotopes ^{79}Br and ^{81}Br and the two additional small peaks shifted by one mass unit originating from the ^{13}C isotope for 16 carbons in the molecule.

The tissues were defreezed at 4°C prior to use. It was homogenized twice in 5 ml of acetonitrile at maximum speed for 3 minutes using a homogenizer (IKA ULTRA-TURRAX Tube drive workstation, Germany). The homogenate was ultrasonicated at 30°C for 5 minutes and centrifuged at 5000g for 10 minutes. An aliquot (100 ul) of supernatant was injected into HPLC system. Briefly, analytical high performance liquid chromatography (HPLC) was performed using a Waters HPLC system with a Waters 996 Photodiode Array Detector. All separations involved a mobile phase of 0.1% trifluoroacetic acid (TFA) (v/v) in water (solvent A) and 0.1% TFA in acetonitrile (solvent B). HPLC was performed using reversed-phase (RP) column Eurospher RP 18, 100 Å, 5µm, 250 × 4.6 mm at flow rates of 1 mL/min with a gradient of solvent B from 0% to 100% in 50 minutes. The effluent was monitored for UV absorption at 260 nm. Samples were quantified using peak area ratio of compounds to external standard."

The reason for the background free detection of anle138b in the HPLC chromatogram is the following:

1) A level of anle138b in the brain is high and anle138b has a high solubility in acetonitrile (solvent that we use for extraction).

2) Many other small organic molecules (e.g. amino acids, lipids, dopamine) that are present in the brain have a lower concentration and/or a lower solubility in acetonitrile and/or a low extinction coefficient at 260 nm. Therefore several small peaks observed in the chromatogram could reflect the presence of other UV-active endogenous compounds from the brain.

3) Proteins and other macromolecules are insoluble in acetonitrile.

Supplement Figure 12:

compound	% inh.	structure	compound	% inh.	structure	compound	% inh.	structure
anle126	32	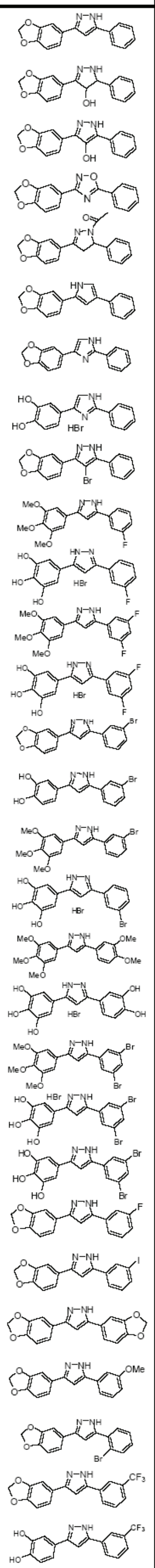	anle237	24	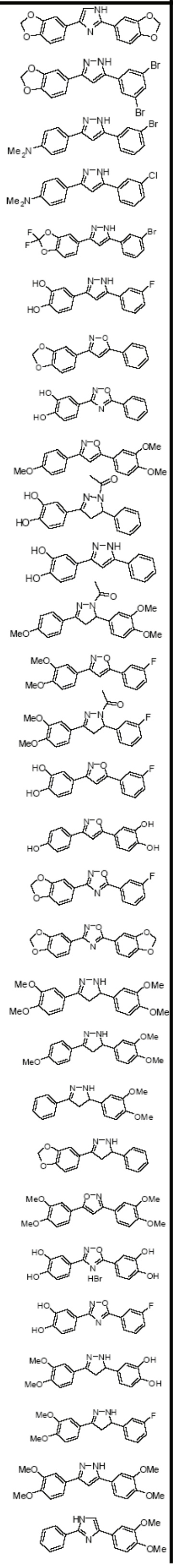	sery153	< 10	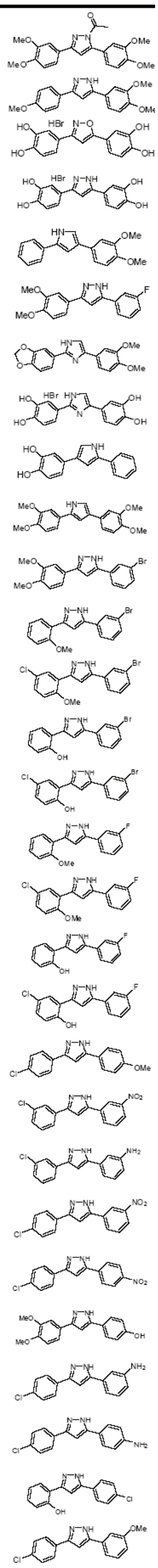
anle127b	40		anle246b	20		sery156	< 10	
anle127c	< 10		anle253b	50		sery158b	98	
anle128b	< 10		anle254b	42		sery159a	100	
anle129	94		anle270	< 10		sery160	< 10	
anle130b	63		sery85	95		sery161	42	
anle131b	< 10		sery93b	50		sery165	< 10	
anle132	96		sery95	69		sery166a	100	
anle134	< 10		sery103	< 10		sery166b	99	
anle136b	< 10		sery105	95		sery167	< 10	
anle136c	99		sery106	97		sery255b	< 10	
anle137b	< 10		sery108	< 10		sery256b	55	
anle137c	100		sery109	95		sery257b	< 10	
**anle138b**	77		sery115	< 10		sery260a	< 10	
anle138c	100		sery117	100		sery260b	< 10	
anle142b	< 10		sery118	76		sery261a	14	
anle142c	95		sery128	< 10		sery261b	65	
anle143b	< 10		sery129	< 10		sery263a	50	
anle143c	n.d.		sery132	23		sery263b	< 10	
anle145b	< 10		sery133	< 10		sery269b	47	
anle145c	96		sery135	< 10		sery275a	< 10	
anle145d	95		sery136	51		sery275b	85	
anle186b	< 10		sery137	< 10		sery278a	< 10	
anle197b	< 10		sery139	100		sery278b	< 10	
anle232b	< 10		sery140	84		sery279b	72	
anle233b	13		sery144	91		sery280a	93	
anle234b	< 10		sery145	72		sery280b	35	
anle236b	< 10		sery149	< 10		sery283	62	
anle236c	100		sery152	< 10		sery289	88	

compound	% inh.	structure	compound	% inh.	structure	compound	% inh.	structure
sery290b	71	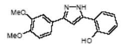	sery369	83	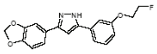	293G02	83	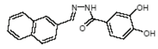
sery292b	30	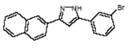	sery378b	74	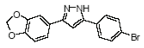	Baicalein	72	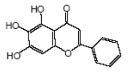
sery294b	< 10	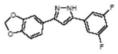	sery383	99	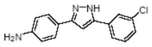			
sery297b	< 10	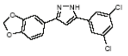	sery384	99	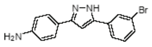			
sery300a	< 10	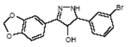	sery392a	< 10	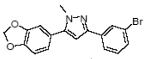			
sery301	13	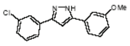	sery392b	< 10	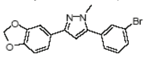			
sery302c	< 10	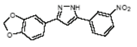	sery417	< 10	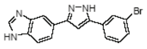			
sery308	88	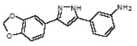						
sery309	n.d.	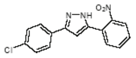						
sery310b	89	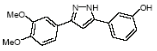						
sery312b	< 10	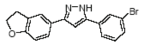						
sery315b	36	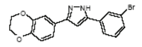						
sery316b	31	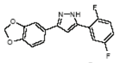						
sery319	75	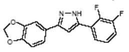						
sery320a	< 10	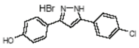						
sery320b	85	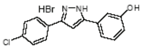						
sery320c	95	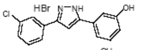						
sery329	26	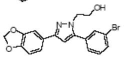 mixture of isomers 1:1 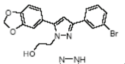						
sery330	< 10	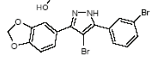						
sery335b	90	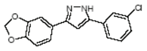						
sery338b	< 10	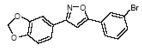						
sery339b	< 10	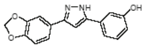						
sery342c	13	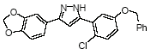						
sery344	98	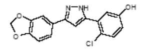						
sery345	57	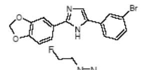						
sery363a	76	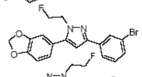						
sery363b	n.d.	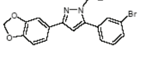						

Results for compounds tested in regard to inhibition of formation of Fe^{3+}/DMSO-induced α-syn oligomers by SIFT assay (see Materials and Methods). Shown are the results for all newly synthesized compounds that are related to the DPP lead structure as they contain two phenyl rings linked by a central five-membered ring containing nitrogen. In addition, the structures of the two control compounds 293G02 and baicalein are shown.

Supplement Figure 13:

“Raw data” for figure 6a:

	% von high control			
	high control	1µM anle138b	3µM anle138b	10µM anle138b
experiment 1	122,33	117,92		48,76
	90,02	103,78		5,55
	87,66	38,70		23,27
experiment 2	103,00			19,10
	97,00			28,20
experiment 3	209,79		19,58	14,39
	68,33		59,54	54,35
	71,13		53,15	31,57
	50,75		64,74	13,19
experiment 4	109,71		54,00	8,76
	105,53		45,20	33,06
	101,72		52,06	18,80
	83,04		69,86	6,90
experiment 5	115,37	46,09	1,19	0,85
	68,98	130,13	-	0,77
	115,64	56,03	20,39	0,06
experiment 6	116,22	65,73	85,74	65,12
	119,84	106,10	78,16	49,83
	84,62	64,30	42,34	31,66
	79,32	71,35	71,67	49,28
experiment 7	96,74	138,71	41,77	35,64
	83,23	66,37	71,37	65,36
	99,31	30,29	41,96	14,60
	120,71	81,70	30,62	57,11
mean	**100,00**	**79,80**	**50,19**	**28,17**
standard dev	**30,21**	**34,26**	**22,37**	**20,99**
SEM	**6,17**	**9,16**	**5,27**	**4,28**

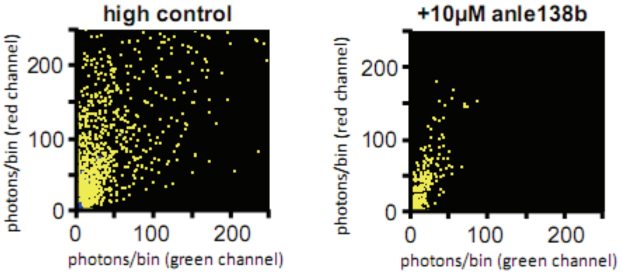

“Raw data” for figure 6b:

	N_pore	N_no pore	total	pore formation [%]	p_Fisher Exact (SigmaStat)
**αSyn (2,1µM)**	40	22	62	64.5%	
**+ anle138b (25µM)**	4	9	13	30.8%	0.046
**+ baicalein (50µM)**	1	7	8	12.5%	0.013

Supplement Figure 14:

Effect of compounds in an MPTP *in vivo* mouse model of Parkinson´s disease
Mice were treated with MPTP (1-methyl-4-phenyl-1,2,3,6-tetrahydropyridine, 30 mg/kg bodyweight daily) by i.p. injection on days 1-5 to induce degeneration of dopaminergic neurons in the substantia nigra pars compacta (SNpc). Animals (8-15 per experimental group) were treated with anle138b (5 mg daily) or vehicle by oral application (gavage, compound in 12.5 µl DMSO mixed with 487.5 µl olive oil, 6 h before the MPTP injections) on days 0-12. Loss of neurons compared to control mice and MPTP-treated mice that were treated with vehicle only (DMSO/olive oil) was quantified on day 12 (i.e. seven days after last MPTP application). For quantification of tyrosine hydroxylase (TH)-positive neurons in the SNpc, 50 µm sections were immunostained with an anti-TH-antibody (1:1000; Zymed). Every second section through the SNpc was analyzed using Stereo investigator software (MicroBrightfield, Colchester, VT, USA). Immunostained cells were counted by the optical fractionator method using a 20x objective. Stereological counts were performed blindly by two independent investigators.

A

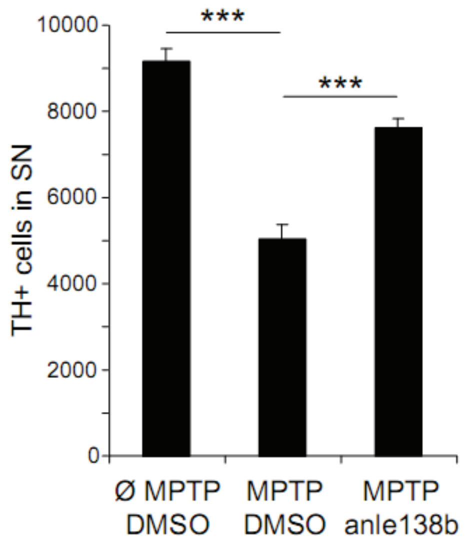

B

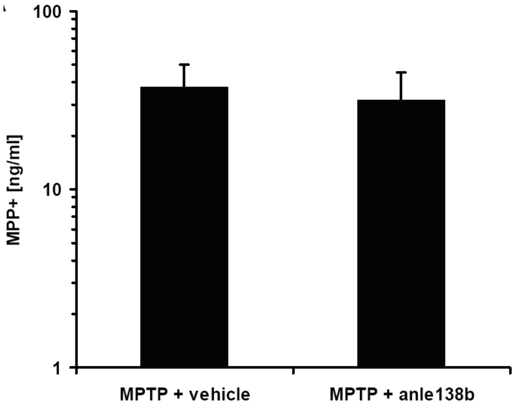

A) A significant loss of dopaminergic neurons in the substantia nigra can be observed in a sub-acute MPTP mouse Parkinson model compared to non-MPTP treated control animals. This effect is significantly ameliorated by treatment with anle138b. Shown is the mean and SEM.
B) MPP+ levels were measured in striatal brain lysates by HPLC. Anle138b does not affect the level of MPP+ found in the brain (p=0.58). Shown is the mean and SD for n=3-4 animals.

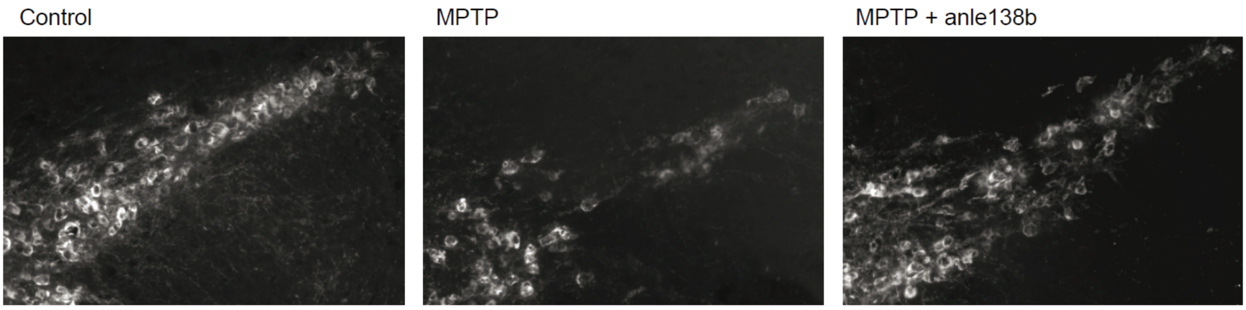

Representative examples of immunohistochemistry for TH-positive cells in substantia nigra.

Supplement Figure 15:

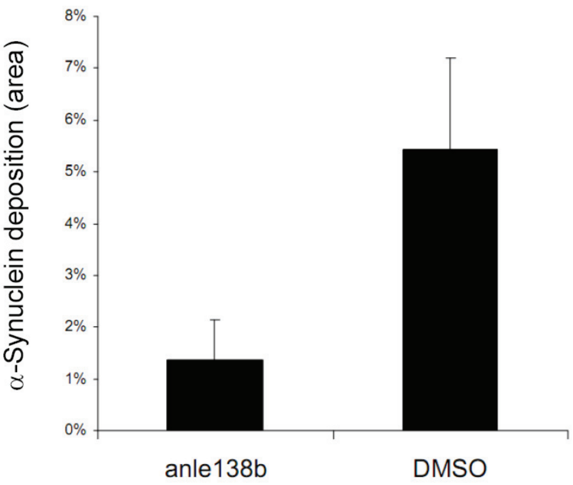

Quantitative analysis of α-synuclein deposition in 69-week old transgenic mice.
As described in the Materials and Methods section, anle138b treatment was tested against placebo treatment with the vehicle (DMSO/peanut butter) in {(Thy1)-h[A30P]α-syn} mice on a genetic background of C57/Bl6. Treatment with anle138b and placebo, respectively, was initiated at the age of eight weeks. During the first two weeks of treatment, 2 mg of anle138b dissolved in 10 µl DMSO mixed with 200 µl peanut butter were given. After two weeks of treatment, the dose was increased to 5 mg in 10 µl DMSO/200 µl peanut butter. At the age of 33 weeks, the dose was increased to 2x5 mg per day. Four animals matched in regard to sex and litter were sacrificed per experimental group at the age of 69 weeks for analysis of pre-terminal histopathological changes. For histopathological and immunohistochemical investigation, formalin-fixed brain tissue was used. Pathological deposits of human α-syn were detected by the anti-human-α-syn antibody 15G7 (see also Fig. 8d). For quantitative analysis, the stained area was quantified in blinded sections at the level of the brainstem by image analysis software cell^{D} 2.5 (Olympus/Soft Imaging Systems GmbH, Münster, Germany). The observed difference was statistically significant (t-test, $p < 0.05$).

Supplement Figure 16:

“Raw data” for figure 4b (all blots are from different mice):

RML untreated

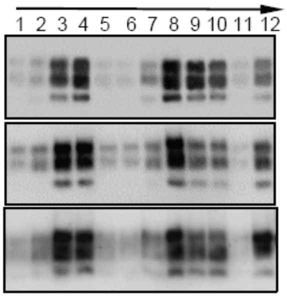

tm ref \ fraction	1	2	3	4	5	6	7	8	9	10	11	12
T-1	0.07	0.17	0.74	1.19	0.08	0.06	0.25	1.66	0.81	0.55	0.07	0.45
T-2	0.08	0.15	0.82	0.63	0.06	0.06	0.19	0.90	0.35	0.33	0.04	0.42
T-3	0.05	0.11	0.69	0.57	0.06	0.06	0.23	0.83	0.24	0.31	0.07	0.88
**mean**	**0.07**	**0.14**	**0.75**	**0.80**	**0.07**	**0.06**	**0.22**	**1.13**	**0.47**	**0.40**	**0.06**	**0.59**
stdev	0.01	0.03	0.06	0.34	0.01	0.003	0.03	0.46	0.30	0.14	0.02	0.26
sterr	0.003	0.01	0.02	0.11	0.003	0.001	0.01	0.15	0.10	0.05	0.007	0.09

unit: pmol

RML anle 80-170 dpi

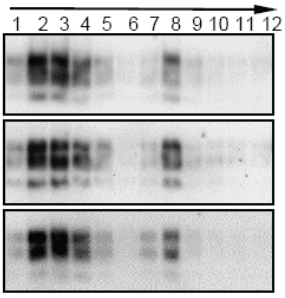

anle 80-170dpi \ fraction	1	2	3	4	5	6	7	8	9	10	11	12
A1-1	0.07	0.42	0.45	0.31	0.09	0.04	0.08	0.19	0.06	0.03	0.04	0.02
A1-2	0.14	0.51	0.50	0.29	0.12	0.04	0.07	0.37	0.04	0.03	0.02	0.03
A1-3	0.09	0.48	0.47	0.35	0.10	0.06	0.12	0.25	0.09	0.02	0.04	0.03
**mean**	**0.10**	**0.47**	**0.47**	**0.32**	**0.10**	**0.05**	**0.09**	**0.27**	**0.06**	**0.03**	**0.03**	**0.03**
stdev	0.03	0.05	0.02	0.03	0.02	0.015	0.02	0.09	0.03	0.006	0.01	0.01
sterr	0.01	0.02	0.007	0.01	0.007	0.005	0.008	0.03	0.01	0.002	0.003	0.003

unit: pmol

RML DMSO 80-170 dpi

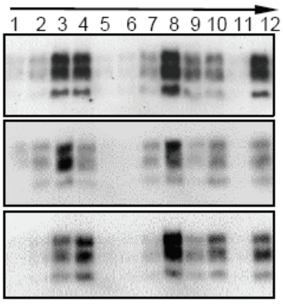

DMSO 80-170dpi \ fraction	1	2	3	4	5	6	7	8	9	10	11	12
D-1	0.07	0.15	0.96	0.92	0.09	0.06	0.37	1.52	0.65	0.41	0.05	0.89
D-2	0.07	0.21	0.62	0.30	0.05	0.06	0.20	0.80	0.21	0.39	0.06	0.30
D-3	0.06	0.10	0.37	0.58	0.06	0.06	0.10	1.22	0.18	0.40	0.05	0.45
**mean**	**0.07**	**0.15**	**0.65**	**0.60**	**0.07**	**0.06**	**0.22**	**1.18**	**0.35**	**0.40**	**0.05**	**0.55**
stdev	0.003	0.06	0.29	0.31	0.018	0.002	0.13	0.36	0.26	0.015	0.003	0.31
sterr	0.001	0.02	0.10	0.10	0.006	0.000	0.04	0.12	0.09	0.005	0.001	0.10

unit: pmol

“Raw data” for figure 8f (all blots are from different mice):

<30-week a-syn mice

sucrose gradient 0-60%

No.25
No.171
No.172

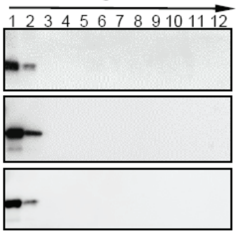

syn-ref \ fraction	1	2	3	4	5	6	7	8	9	10	11	12
No.25	6.09	1.87	0	0	0	0	0	0	0	0	0	0
No.171	8.78	3.06	0	0	0	0	0	0	0	0	0	0
No.172	6.14	2.08	0	0	0	0	0	0	0	0	0	0
**mean**	**6.34**	**2.34**										
stdev	2.35	0.64										
sterr	0.78	0.21										

unit: pmol

69-week anle138b-treated a-syn mice

sucrose gradient 0-60%

No.43
No.46
No.66
No.64

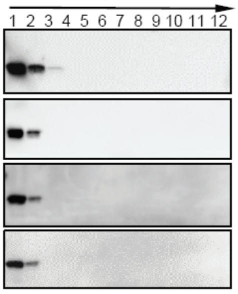

syn-anle \ fraction	1	2	3	4	5	6	7	8	9	10	11	12
no.43	10.18	5.87	0.66	0	0	0	0	0	0	0	0	0
no.46	7.16	3.12	0	0	0	0	0	0	0	0	0	0
no.66	9.26	4.03	0	0	0	0	0	0	0	0	0	0
no.64	6.77	3.26	0	0	0	0	0	0	0	0	0	0
**mean**	**8.34**	**4.07**	**0.17**									
stdev	1.64	1.26	0.33									
sterr	0.55	0.42	0.11									

unit: pmol

69-week DMSO-treated a-syn mice

sucrose gradient 0-60%

No.40
No.47
No.68
No.65

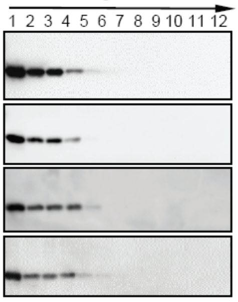

syn-DMSO \ fraction	1	2	3	4	5	6	7	8	9	10	11	12
no.40	7.92	3.51	2.53	0.43	0.09	0.02	0	0	0	0	0	0
no.47	6.32	2.39	2.41	0.29	0.07	0	0	0	0	0	0	0
no.68	6.39	2.82	2.02	1.68	0.22	0.05	0	0	0	0	0	0
no.65	6.45	2.56	1.87	1.63	0.36	0.19	0	0	0	0	0	0
**mean**	**6.77**	**2.82**	**2.21**	**1.01**	**0.19**	**0.07**						
stdev	2.43	0.49	0.31	0.75	0.13	0.09						
sterr	0.61	0.16	0.10	0.19	0.03	0.02						

unit: pmol

α-syn monomer in sucrose-gradient

sucrose gradient 0-60%

m-1
m-2
m-3

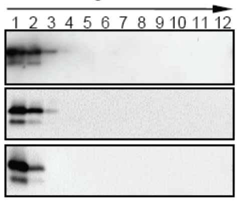

syn-monomer \ fraction	1	2	3	4	5	6	7	8	9	10	11	12
m-1	6.55	3.34	0.45	0	0	0	0	0	0	0	0	0
m-2	6.29	3.97	0.15	0	0	0	0	0	0	0	0	0
m-3	9.98	2.43	0	0	0	0	0	0	0	0	0	0
**mean**	**7.61**	**3.25**	**0.2**									
stdev	2.06	0.77	0.23									
sterr	0.69	0.26	0.07									

unit: pmol

α-syn oligomer in sucrose-gradient

sucrose gradient 0-60%

o-1
o-2
o-3

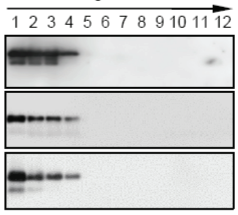

syn-oligomer \ fraction	1	2	3	4	5	6	7	8	9	10	11	12
o-1	5.15	3.55	3.19	0.89	0	0	0	0	0	0	0	0
o-2	7.25	2.28	2.34	1.19	0	0	0	0	0	0	0	0
o-3	9.15	2.16	1.83	0.69	0	0	0	0	0	0	0	0
**mean**	**7.18**	**2.66**	**2.45**	**0.92**								
stdev	2.00	0.77	0.69	0.25								
sterr	0.67	0.26	0.23	0.08								

unit: pmol

Supplement Figure 17:

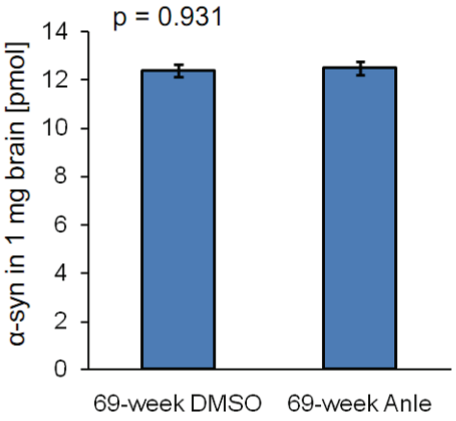

The amount of total alpha-synuclein was quantified by western blot analysis of brain homogenates from 69-week old mice treated with anle138b and DMSO-treated control mice. For every mouse, 10 µl of 10% brain homogenates (1 mg of brain) per lane were prepared for western blotting. Each group contained three mice. Samples were boiled with 2 x loading buffer at 100°C for 10 min followed by separating on 15% SDS-PAGE gel. Proteins were transferred to PVDF membrane and detected by monoclonal antibody 15G7 (1:1000). No difference in level of total synuclein was found.

Supplement Figure 18:

Preclinical studies regarding acute toxicity and mutagenicity of anle138b.
These experiments were outsourced to LPT Laboratory of Pharmacology and Toxicology, Hamburg, Germany. Studies were carried out according to the 'Good Laboratory Practice' Regulations.

a) acute toxicity study in mice (NMRI, Charles River)

Symptoms/<br>Criteria	2000 mg/kg b.w.<br>(n = 5)<br>males	 <br>females
clinical signs	none	none
mortality<br>within 6 h<br>within 24 h<br>within 7 d<br>within 14 d	 0<br>0<br>0<br>0	 0<br>0<br>0<br>0
mean body weight (in g)<br>start<br>after 7 days<br><br>after 14 days	 31.6<br>36.8<br>(+16.5)<br>38.0<br>(+20.3)	 25.8<br>29.6<br>(+14.7)<br>31.0<br>(+20.2)
inhibition of body weight gain	none	none
necropsy findings	none	none

in brackets: body weight gain (%), compared with the start value
d = days
h = hours

2000 mg anle138b were mixed with 2 mL DMSO and allowed to stand 5 minutes in a 40°C water bath, followed by addition of olive oil to a total volume of 40 mL, followed by 5 minutes in the water bath and vortexing for 20 seconds. The administration volume was 40 mL/kg b.w., per oral.
Observations were performed before and immediately, 5, 15, 30 and 60 min, as well as 3, 6 and 24 hours after the administration. All animals were observed. During the 14-day follow-up period changes of skin and fur, eyes and mucous membranes, respiratory and the circulatory functions, autonomic and central nervous system and somatomotor activity, as well as behavioural pattern were observed at least once a day until all symptoms had subsided, thereafter each working day. Attention was also paid to possible tremors, convulsions, salivation, diarrhoea, lethargy, sleep and coma. Observations on mortality were made at least once daily to minimize loss of animals during the study.
Individual body weights were recorded before administration of the test item and thereafter in weekly intervals up to the end of the study. Changes in weight were calculated when survival exceeded one day.
At the end of the experiment all animals were sacrificed, dissected and inspected macroscopically. All gross pathological changes were recorded. No microscopic examination was performed as no pathological findings were noted at necropsy of the animals at the end of the 14-day observation period.

Under the present test conditions, a single oral administration of 2000 mg anle138b/kg b.w. to mice did not reveal any signs of toxicity and no mortality. All animals gained the expected body weight throughout the whole study period. No macroscopical changes were noted at necropsy.

b) acute toxicity study in rats (CD, Charles River)

Symptoms/ Criteria	2000 mg/kg b.w. (n = 5) males	females
clinical signs	none	none
mortality		
within 6 h	0	0
within 24 h	0	0
within 7 d	0	0
within 14 d	0	0
mean body weight (in g)		
start	215.4	185.0
after 7 days	281.6 (+30.7)	218.0 (+17.8)
after 14 days	330.6 (+53.5)	227.8 (+23.1)
inhibition of body weight gain	none	none
necropsy findings	none	none

in brackets: body weight gain (%), compared with the start value
d = days
h = hours

2000 mg anle138b were mixed with 2 mL DMSO and allowed to stand 5 minutes in a 40°C water bath, followed by addition of olive oil to a total volume of 40 mL, followed by 5 minutes in the water bath and vortexing for 20 seconds. The administration volume was 40 mL/kg b.w., per oral.
Observations were performed before and immediately, 5, 15, 30 and 60 min, as well as 3, 6 and 24 hours after the administration. All animals were observed. During the 14-day follow-up period changes of skin and fur, eyes and mucous membranes, respiratory and the circulatory functions, autonomic and central nervous system and somatomotor activity, as well as behavioural pattern were observed at least once a day until all symptoms had subsided, thereafter each working day. Attention was also paid to possible tremors, convulsions, salivation, diarrhoea, lethargy, sleep and coma. Observations on mortality were made at least once daily to minimize loss of animals during the study.
Individual body weights were recorded before administration of the test item and thereafter in weekly intervals up to the end of the study. Changes in weight were calculated when survival exceeded one day.
At the end of the experiment all animals were sacrificed, dissected and inspected macroscopically. All gross pathological changes were recorded. No microscopic examination was performed as no pathological findings were noted at necropsy of the animals at the end of the 14-day observation period.

Under the present test conditions, a single oral administration of 2000 mg anle138b/kg b.w. to rats did not reveal any signs of toxicity and no mortality. All animals gained the expected body weight throughout the whole study period. No macroscopical changes were noted at necropsy.

c) Mutagenicity Study in the *Salmonella typhimurium* Reverse Mutation Assay (AMES test)

Metabolic Activation	Test Article	Dose Level (µg/plate)	1st Experiment: Plate Incorporation Test				
			Revertants per Plate (Mean ± SD)				
			TA 98	TA 100	TA 102	TA 1535	TA 1537
Without Activation	Solvent Control: DMSO	100 µL/plate	33.7 ± 3.1	155.0 ± 23.3	273.3 ± 28.7	18.3 ± 4.6	8.3 ± 1.5
	ANLE138B	100	33.0 ± 6.1#	148.3 ± 17.5#	265.3 ± 19.1#	15.7 ± 1.5#	7.3 ± 0.6#
		31.6	32.0 ± 7.0	153.7 ±15.0	268.7 ± 12.7	19.0 ± 1.7	7.0 ± 1.0
		10.0	34.0 ± 9.5	144.3 ± 5.9	270.0 ± 21.3	18.0 ± 1.0	8.0 ± 2.6
		3.16	34.0 ± 6.2	133.0 ± 3.6	254.7 ± 7.2	17.0 ± 1.7	8.0 ± 2.0
		1.0	31.7 ± 2.3	142.7 ± 14.0	283.0 ± 11.5	14.7 ± 3.1	9.0 ± 1.0
	Positive Controls:						
	2-nitro-fluorene	10	797.3 ± 10.0				
	Sodium azide	10		982.3 ± 5.7		141.3 ± 4.9	
	Methylmethane sulfonate	1300			1053.0 ± 42.7		
	9-amino-acridine	100					106.3 ± 4.2
With Activation	Solvent Control: DMSO	100 µL/plate	35.3 ± 4.2	169.3 ± 12.7	276.7 ± 24.2	16.3 ± 2.3	7.0 ± 3.0
	ANLE138B	3160	31.7 ± 3.1#	146.3 ± 2.3#	256.0 ± 14.5#	17.3 ± 2.5#	8.3 ± 0.6#
		1000	34.3 ± 2.3	168.0 ± 11.3	277.0 ± 11.1	15.3 ± 1.5	7.3 ± 2.1
		316	33.0 ± 3.6	146.0 ± 1.7	275.7 ± 18.9	14.3 ± 2.3	4.3 ± 1.5
		100	33.0 ± 4.6	167.7 ± 17.6	260.0 ± 12.8	17.7 ± 2.9	5.7 ± 1.5
		31.6	35.3 ± 5.1	155.0 ± 3.0	271.0 ± 6.9	14.7 ± 0.6	6.7 ± 2.5
	Positive Controls:						
	2-amino-anthracene	2	1045.3 ± 10.3		1072.3 ± 41.6		89.7 ± 2.1
	Cyclophosphamide	1500		973.0 ± 19.9		112.3 ± 2.1	

Metabolic Activation	Test Article	Dose Level (µg/plate)	2nd Experiment: Preincubation Test				
			Revertants per Plate (Mean ± SD)				
			TA 98	TA 100	TA 102	TA 1535	TA 1537
Without Activation	Solvent Control: DMSO	100 µL/plate	22.7 ± 3.1	129.7 ± 12.3	274.0 ± 12.8	30.7 ± 2.1	6.0 ± 1.7
	ANLE138B	100	29.7 ± 11.6#	113.0 ± 5.3#	260.7 ± 7.4#	16.0 ± 5.6#	5.0 ± 1.0#
		31.6	29.3 ± 2.1	118.7 ± 7.1	262.0 ± 4.6	14.3 ± 2.1	7.0 ± 1.0
		10.0	30.7 ± 4.0	119.0 ± 19.0	263.3 ± 4.5	18.3 ± 2.1	7.0 ± 1.7
		3.16	42.7 ± 7.6	126.0 ± 20.0	259.3 ± 4.0	16.3 ± 4.2	7.3 ± 1.5
		1.0	42.3 ± 11.6	131.0 ± 4.6	264.0 ± 7.0	18.3 ± 5.9	7.0 ± 1.7
	Positive Controls:						
	2-nitro-fluorene	10	461.3 ± 21.8				
	Sodium azide	10		870.3 ± 16.7		559.0 ± 28.9	
	Methylmethane sulfonate	1300			1054.3 ± 24.4		
	9-Amino-acridine	100					365.0 ± 27.0
With Activation	Solvent Control: DMSO	100 µL/plate	36.3 ± 7.5	124.3 ± 11.7	264.0 ± 5.6	24.3 ± 1.5	6.7 ± 1.2
	ANLE138B	100	34.3 ± 11.0#	92.3 ± 10.0#	254.0 ± 2.6#	15.7 ± 4.5#	6.3 ± 0.6#
		31.6	31.7 ± 4.9	112.0 ± 8.2	267.7 ± 5.7	19.0 ± 2.6	7.3 ± 2.1
		10.0	39.3 ± 5.8	117.0 ± 1.0	262.3 ± 4.0	21.3 ± 3.2	6.3 ± 2.3
		3.16	25.0 ± 4.4	109.0 ± 5.0	265.7 ± 6.0	22.7 ± 4.5	6.3 ± 0.6
		1.0	25.7 ± 0.6	119.3 ± 4.0	267.0 ± 7.9	23.7 ± 1.5	7.3 ± 0.6
	Positive Controls:						
	2-amino-anthracene	2	466.3 ± 14.5		1023.3 ± 31.2		330.7 ± 18.6
	Cyclophosphamide	1500		872.7 ± 12.1		555.0 ± 16.5	

\# test item precipitation
SD standard deviation

Anle138b was examined in the 5 Salmonella typhimurium strains TA 98, TA 100, TA 102, TA 1535 and TA 1537 in two independent experiments, each carried out without and with metabolic activation (a microsomal preparation derived from Aroclor 1254-induced rat liver). The first experiment was carried out as a plate incorporation test and the second as a preincubation test. Anle138b was dissolved in dimethyl sulfoxide (DMSO).

ANLE138B was examined in a preliminary cytotoxicity test without metabolic activation in test strain TA 100 employing a plate incorporation test. Ten concentrations ranging from 0.316 to 5000 µg/plate were tested. No signs of cytotoxicity were noted up to the top concentration of 5000 µg/plate. Test item precipitation was noted from a concentration of 100 µg/plate onwards. Hence, 100 µg/plate were

chosen as the top concentration for the main study. In the main study, five concentrations ranging from 1.0 to 100 µg/plate were employed in independent experiments, each carried out without and with metabolic activation. No signs of cytotoxicity were noted up to the top concentration of 100 µg/plate in the plate incorporation test and the preincubation test, each carried out without and with metabolic activation in any test strain. No mutagenic effect (no increase in revertant colony numbers as compared with control counts) was observed for anle138b tested up to a concentration of 100 µg/plate, that led to test item precipitation in any of the 5 test strains in two independent experiments without and with metabolic activation (plate incorporation and preincubation test, respectively). In conclusion, under the present test conditions anle138b tested up to a concentration of 100 µg/plate, that led to test item precipitation caused no mutagenic effect in the Salmonella typhimurium strains TA 98, TA 100, TA 102, TA 1535 and TA 1537 neither in the plate incorporation test nor in the preincubation test each carried out without and with metabolic activation.

d) *in vitro* assessment of the clastogenic activity in cultured human peripheral lymphocytes

Test samples of anle138b were assayed in an *in vitro* cytogenetic study using human lymphocyte cultures both in the presence and absence of metabolic activation by a rat liver post-mitochondrial fraction (S9 mix) from Aroclor 1254 induced animals. Anle138b was dissolved in dimethyl sulfoxide (DMSO). The test was carried out employing 2 exposure times without S9 mix: 4 and 24 hours, and 1 exposure time with S9 mix: 4 hours. The experiment with S9 mix was carried out twice. The harvesting time was 24 hours after starting of exposure. The incubation procedure took place in the dark. The study was conducted in duplicate. Mitomycin C and cyclophosphamide were employed as positive controls in the absence and presence of metabolic activation, respectively.

Tests without metabolic activation (4- and 24-hour exposure)
The mean incidence of chromosomal aberrations (excluding gaps) of the cells treated with anle138b at concentrations from 312.5 to 2500 or 78.13 to 312.5 µg/mL medium (4-h or 24-h exposure) in the absence of metabolic activation ranged from 1.5% to 4.0%. The results obtained are considered to be within the normal range of the solvent control where a mean incidence of chromosomal aberrations (excluding gaps) of 1.5% or 1.0% was observed after a 4-hour and 24-hour exposure, respectively. Only at the pronounced cytotoxic concentration of 625 µg/mL medium (24-hour exposure, only 92 of 200 metaphases could be evaluated) a marginal, though not significant increase to 4.3% was noted in the number of aberrations. It is known that high cytotoxicity causes artefacts in the form of aberrations in *in vitro* chromosomal tests. Hence, the increase at the concentration of 625 µg anle138b/mL medium (24-hour exposure) is considered as artefact and not test item-related.

Test with metabolic activation (4-hour exposure)
The mean incidence of chromosomal aberrations (excluding gaps) of the cells treated with ANLE138B at concentrations from 312.5 to 2500 or 312.5 to 1250 µg/mL medium in the presence of metabolic activation in the first and second experiment, respectively, ranged from 1.5% to 3.5%. The results obtained are considered to be within the normal range of the solvent control where a mean incidence of chromosomal aberrations (excluding gaps) of 1.5% was observed after a 4-hour exposure. Only at the pronounced cytotoxic concentration of 2500 µg/mL medium (the second experiment, only 130 of 200 metaphases could be evaluated) an increase to 4.6% (significant at $p \leq 0.05$) was noted in the number of aberrations. It is known that high cytotoxicity causes artefacts in the form of aberrations in *in vitro* chromosomal tests. Hence, the increase at the concentration of 2500 µg anle138b/mL medium in the second experiment is considered as artefact and not test item-related. No item-related polyploidy or endoreduplication was noted in the experiments with or without metabolic activation.
Thus, under the present test conditions, anle138b tested up to cytotoxic concentrations in the absence and in the presence of metabolic activation employing two exposure times (without S9) and one exposure time (with S9) revealed no indications of mutagenic properties with respect to chromosomal or chromatid damage. In the same test, Mitomycin C and cyclophosphamide induced significant damage.

Supplement Figure 19:

Anle138b and related compounds do not affect actin polymerization

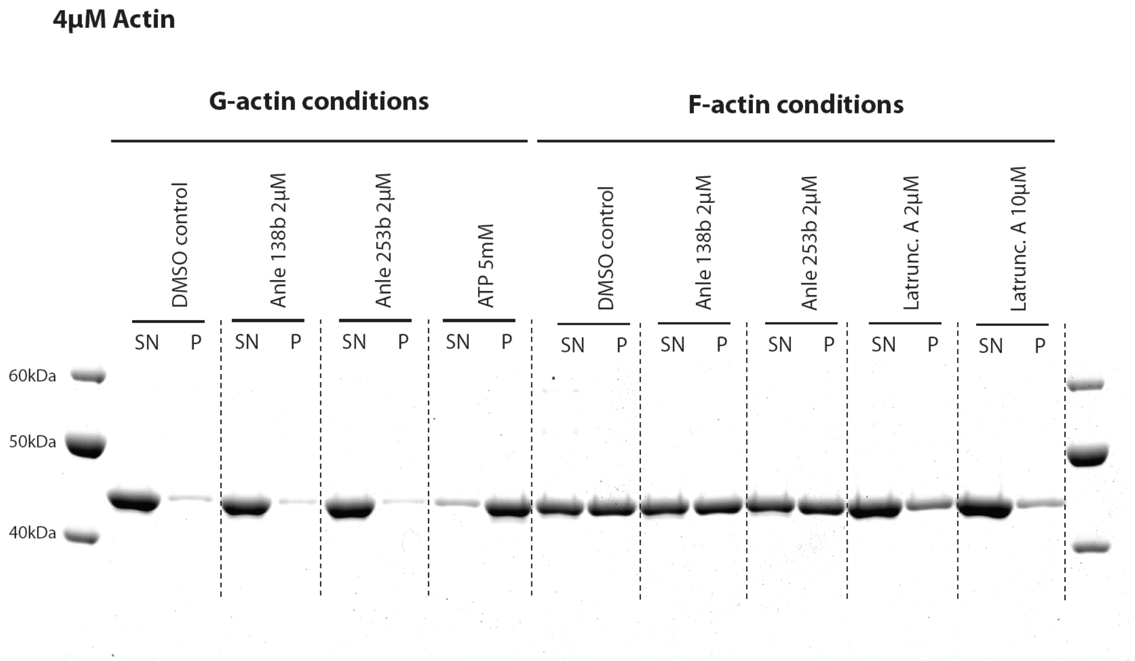

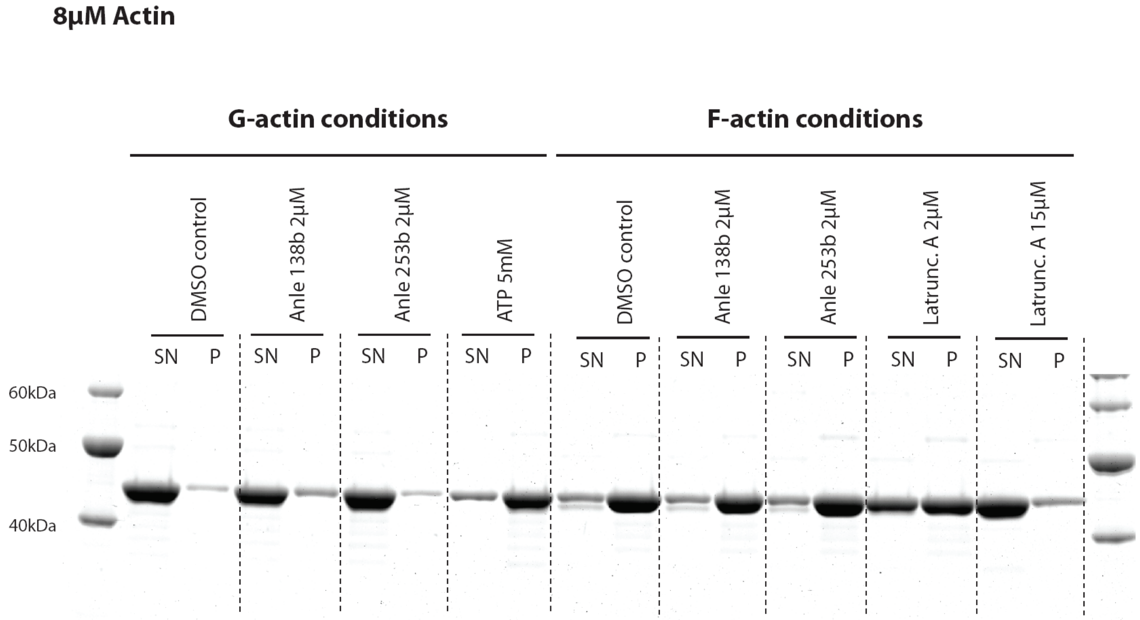

Supplement Figure 19(cont.):

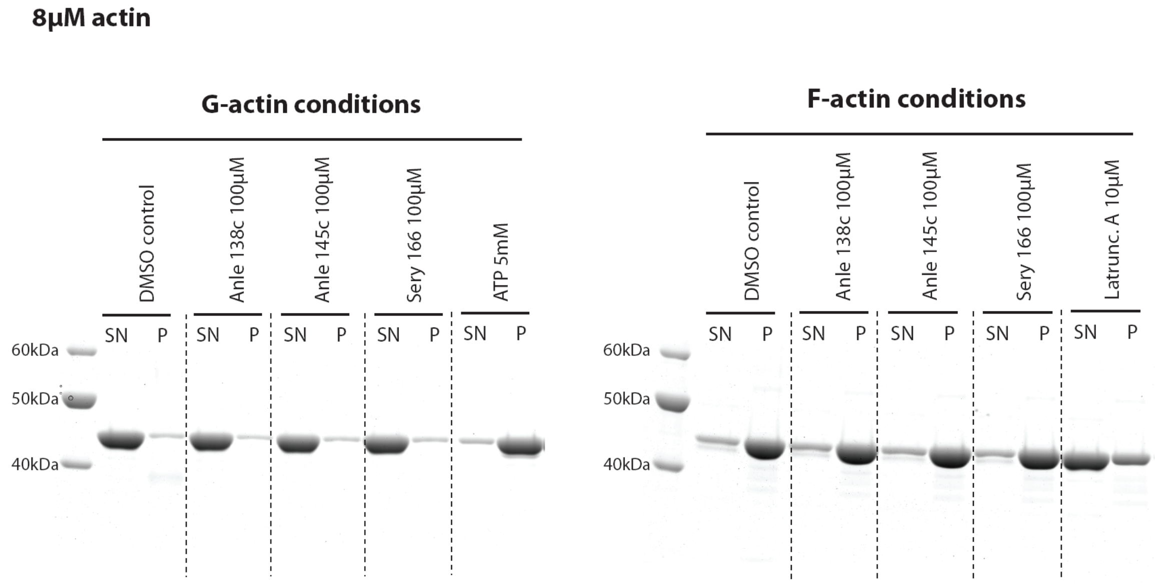

Anle138b and related compounds do not affect actin polymerization

The G-actin condition (10 mM Tris pH 7.5, 0.2 mMCaCl$_2$) does not support polymerization of actin, thus actin is expected to stay in the supernatant after incubation and high speed centrifugation. If a compound interferes with actin protein integrity, aggegates should form and more actin will be found in the pellet. The F-actin condition (10 mM Tris pH 7.5, 0.2 mM CaCl$_2$, 2 mM MgCl$_2$, 100 mM KCl, 0.5 mM ATP) supports actin polymerization, thus actin is expected to be found in the pellet after incubation and high speed centrifugation. ATP, which induces aggregation in G-actin conditions, and Lactrunculin A, which inhibits actin polymerization in F-actin conditions, were used as controls (Coué M, Brenner SL, Spector I, Korn ED. Inhibition of actin polymerization by latrunculin A. FEBS Lett (1987) 213: 316-8). G-actin in G-buffer was cleared of potential preformed aggregates by high-speed centrifugation (30 min, 130,000g). 100x stock solutions of the compounds were prepared in DMSO and premixed with the respective buffer prior to addition of G-actin solution. Then samples were incubated for 30 min at RT and subsequently centrifuged at high speed (30 min, 130,000g). Highly soluble compounds (anle138c, anle145c, sery166) were used at 100 µM, compounds with lower solubility in water (anle138b, anle253b) were used at 2µM. None of the DPP compounds tested had a detectable effect on actin polymerization.

Supplement Figure 20:

compound	Mouse	Prion strain	Treatment	Dose, mg/kg	Median/Mean* Survival Control, days	Median/Mean* Survival Therapy, days	Δ, days	Reference
Anle138b	C57BL/6	RML, i.c.	0 dpi, oral	250	180	355	175	
Anle138b	C57BL/6	RML, i.c.	80 dpi, oral	250	168	242	74	
Anle138b	C57BL/6	RML, i.c.	120 dpi, oral	250	172	224	52	
CompoundB	Tga20	RML, i.c.	0 dpi, oral	300	63	174	111	1
CompoundB	ICR	RML, i.c.	0-187 dpi, oral	300	156	270	120	1
Amphoterin B	C57BL/6	C506M3, i.c.	0 dpi, i.p.	2.5	171	223	52	2
Amphoterin B	C57BL/6	C506M3, i.c.	80 dpi, i.p.	2.5	158	196	38	3
MS-8209	C57BL/6	C506M3, i.c.	0 dpi, i.p.	2.5	171	215	44	2
MS-8209	C57BL/6	C506M3, i.c.	80 dpi, i.p.	25	158	221	63	3
Curcumin	C57BL/6	139A, i.c.	100 dpi, oral	50	196*	208*	12	4
PcTS	RML	RML, i.c.	0-28 dpi, ip	25	174.7*	171.1*	-3.6	5
PcTS	Tg7	263K, i.c.	0-28 dpi, ip	25	46.3*	49.6*	3.3	5
PPS	Tg7	263K, i.c.	10-38 dpi, ip	20			No effect	6
Comp 59	CD-1	139A, i.p.	0 dpi, ip	10	170	215	45	7
Trimipramine	CD-1	139A, i.p.	0 dpi, ip	10	170	210	40	7
Fluphenazine	CD-1	139A, i.p.	0 dpi, ip	10	170	205	35	7
Pravastatin	C57BL/6	139A, i.c.	0 dpi, oral	200	177*	194*	17	8
Simvastatin	C57BL/6	RML, i.c.	41 dpi, oral	20	148*	167*	19	9
Na2CaCDTA	Balb/c	M1000	7 dpi, ip	200 ul 0.2 M	169*	189*	20	10
Tacrolimus	C57BL/6	RML, i.p.	Simptoms, ip	5	26*	35*	9	11

References:

1. Kawasaki, Y. et al. (2007). Orally administered amyloidophilic compound is effective in prolonging the incubation periods of animals cerebrally infected with prion diseases in a prion strain-dependent manner. J. Virol. **81**, 12889-12898.
2. Demaimay, R. et al. (1994). Pharmacological studies of a new derivative of amphotericin B, MS-8209, in mouse and hamster scrapie. J. Gen. Virol. **75**, 2499-2503.
3. Demaimay, R. et al. (1997). Late treatment with polyene antibiotics can prolong the survival time of scrapie-infected animals. J. Virol. **71**, 9685-9689.
4. Riemer, C. et al. (2008). Evaluation of drugs for treatment of prion infections of the central nervous system. J. Gen. Virol. **89**, 594-597.
5. Priola, S. A., Raines, A. & Caughey, W. (2003). Prophylactic and therapeutic effects of phthalocyanine tetrasulfonate in scrapie-infected mice. J. Infect. Dis. **188**, 699-705.
6. Doh-ura, K. et al. (2004). Treatment of transmissible spongiform encephalopathy by intraventricular drug infusion in animal models. J. Virol. **78**, 4999-5006.
7. Chung, E. et al. (2011). Styryl-based and tricyclic compounds as potential anti-prion agents. PLoS One **6**, e24844.
8. Vetrugno, V. et al. (2009). Oral pravastatin prolongs survival time of scrapie-infected mice. J. Gen. Virol. **90**, 1775-1780.
9. Haviv, Y. et al. (2008). Induced neuroprotection independently from PrPSc accumulation in a mouse model for prion disease treated with simvastatin. Arch. Neurol. **65**, 762-775.
10. Brazier, M. W. et al. (2010). Manganese chelation therapy extends survival in a mouse model of M1000 prion disease. J. Neurochem. **114**, 440-451.
11. Mukherjee, A. et al. (2010). Calcineurin inhibition at the clinical phase of prion disease reduces neurodegeneration, improves behavioral alterations and increases animal survival. PLoS Pathog. **6**, e1001138.
