# Supplementary material for: Anle138b: a novel oligomer modulator for disease-modifying therapy of neurodegenerative diseases such as prion and Parkinson’s disease
Source: Acta Neuropathol. 2013 Apr 19;125(6):795–813. doi: 10.1007/s00401-013-1114-9 (PMC3661926; doi:10.1007/s00401-013-1114-9)
Supplement: Supplementary file 2 — Supplementary material 2 (PDF 4030 kb) [file 401_2013_1114_MOESM2_ESM.pdf]

1 Materials and methods

All starting materials and solvents were purchased from ABCR, Acros, Alfa Aesar, Fluorochem, Sigma-Aldrich or Merck and used as such unless noted otherwise. Melting points were determined on a Stuart Scientific (BIBBY, UK) melting point apparatus using open glass capillares and are uncorrected. Thin layer chromatography (TLC): Macherey-Nagel precoated sheets, 0.25 mm ALUGRAM® SIL G/UV254 plates, detection with UV and/or by charring with 10 wt% ethanolic phosphomolybdic acid reagent followed by heating at 200 °C. Flash column chromatography was performed by using Merck silica gel 60 (0.063–0.100 mm). Analytical and preparative high performance liquid chromatography (HPLC) were performed by using a Waters HPLC system with a Waters 996 Photodiode Array Detector. All separations involved a mobile phase of 0.1% trifluoroacetic acid (TFA) (v/v) in water (solvent A) and 0.1% TFA in acetonitrile (solvent B). HPLC was performed by using a reversed-phase (RP) column Eurospher RP 18, 100 Å, 5 µm, 250×4.6 mm (analytical) and 250×16 mm (preparative) at flow rates of 1 $mL\cdot min^{-1}$ (analytical) and 7 $mL\cdot min^{-1}$ (preparative). Electrospray ionization mass spectrometry (ESI-MS) and liquid chromatography/ mass spectrometry (LC/MS) analyses were obtained by using a Waters Micromass ZQ 4000 mass spectrometer in conjunction with the Waters HPLC apparatus described above. High-resolution mass spectra (HRMS) were recorded by using an Thermo Scientific LTQ Orbitrap XL hybrid FTMS mass spectrometer and are reported in *m*/*z*. NMR spectra were recorded at a temperature of 298 K by using a 400 MHz Bruker Avance spectrometer (Bruker AG, Rheinstetten, Germany) equipped with a TXI HCN z-gradient probe. All spectra were processed by using TOPSPIN2 (Bruker AG, Karlsruhe, Germany). ^{1}H NMR chemical shifts (δ) are reported in parts per million (ppm) relative to $CHCl_3$ and $[D_5]$DMSO as internal standards. Data are reported as follows: chemical shift, multiplicity (s = singlet, d = doublet, t = triplet, b = broadened, m = multiplet), coupling constants (*J*, given in Hz), integration. ^{13}C NMR chemical shifts (δ) are reported in parts per million (ppm) relative to $CDCl_3$ and $[D_6]$DMSO as internal standards. The following experiments were used to record the resonances of the compounds: ^{1}H-1D, ^{13}C-1D NMR spectra and ^{13}C-APT (attached proton test with a single J-evolution time of 1/145 s, spectra are processed such that quaternary and methylene groups have positive sign and methyl and methine groups negative sign). To resolve overlap of resonances and recover undetectable resonances in ^{1}H and APT spectra, 2D-[^{13}C,^{1}H]-HSQC (heteronuclear single quantum coherence), 2D-[^{13}C,^{1}H]-HMBC (heteronuclear multiple bond correlation) and 2D-NOESY were recorded for some compounds. 1,3-Diarylpropane-1,3-diones are presented in the figures as diketones despite the fact that the enol form dominates the spectra.

2 Experimental Procedures and characterisation data of compounds

2.1 Synthesis of pyrazoles

General Experimental Procedure for the synthesis of 1,3-diarylpropane-1,3-diones (GEP 1)[1]

1,3-Bis(3,4-dimethoxyphenyl)propane-1,3-dione (sery148)

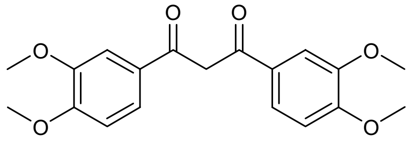

This compound has been reported previously[2] and is commercially available (CAS 52751-63-0). A 60 % suspension of sodium hydride in mineral oil (0.4 g, 10 mmol) was washed with petroleum benzin (20 ml) two times, anhydrous DMSO (10 ml) was added. After being stirred for 30 min at room temperature under argon, THF (5 ml) was added, the flask was cooled down to 15°C and ethyl 3,4-dimethoxybenzoate (2.1 g, 10 mmol) was added. The temperature was allowed to drop to 10 °C and a solution of 3,4-dimethoxyacetophenone (1.08 g, 6 mmol) in DMSO (4 ml) was added at such a rate that the temperature didn't rise above 15 °C. Upon completion of addition the reaction mixture was stirred 15 h at room temperature, then poured slowly into crushed ice (250 g) containing 85 % phosphoric acid (1 ml). Resulting precipitate was collected by filtration, washed with water (50 ml) and dried to provide **sery148** (2.1 g, 99 %) as a yellow powder. m.p.: 128-130 °C (lit[2] 100.5-101 °C). ^{1}H NMR (400 MHz, DMSO$[D_6]$) δ = 7.85 (dd, *J* = 8.5, 1.9 Hz, 2H), 7.61 (d, *J* = 1.9 Hz, 2H), 7.21 (s, 1H), 7.11 (d, *J* = 8.5 Hz, 2H), 3.88 (m, 12H) (in ^{1}H NMR spectrum 15% of diketone form is present). ^{13}C NMR (100.6 MHz, DMSO$[D_6]$) δ = 184.2, 152.9, 148.7, 127.2, 121.7, 111.2, 110.0, 91.8, 55.7 (2C).

General Experimental Procedure for the synthesis of 3,5-diaryl-1*H*-pyrazoles (GEP 2)[3]

1,3-Bis(3,4-dimethoxyphenyl)-1*H*-pyrazole (sery149)

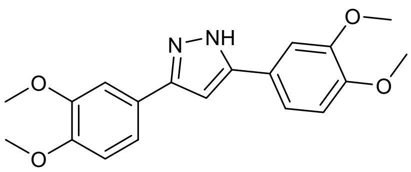

This compound is commercially available (CAS 1159937-09-3). A solution of **sery148** (1.0 g, 2.9 mmol) and hydrazine hydrate (218 mg, 4.4 mmol) in ethanol (15 ml) was heated under reflux 3 h with stirring. The clear yellow solution was evaporated under reduced pressure, water was added and resulting precipitate was collected by filtration, washed with water and dried to provide **sery149** (960 mg, 97 %) as a beige powder. m.p.: 165-167 °C. ^{1}H NMR (400 MHz, DMSO[D$_6$]) δ = 13.07 (s, 1H), 7.41 (s, 2H), 7.36 (d, J = 8.2 Hz, 2H), 7.06 (s, 1H), 7.01 (d, J = 8.2 Hz, 2H), 3.84 (s, 6H), 3.79 (s, 6H). ^{13}C NMR (100.6 MHz, DMSO[D$_6$]) δ = 148.9, 148.6, 143.6, 122.1, 117.6, 112.1, 108.9, 98.6, 55.5 (2C). HRMS (m/z) [M+H]$^+$ calcd for M = $C_{19}H_{20}N_2O_4$, 341.1501; found, 341.1505.

General Experimental Procedure for the *O*-deprotection of phenyl alkyl ethers (GEP 3)[4]

3,5-Bis(3,4-dihydroxyphenyl)-1*H*-pyrazole hydrobromide (sery159a)

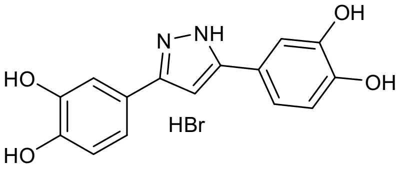

This compound has been reported previously[5] (CAS 1147315-28-3). A solution of **sery149** (120 mg, 0.35 mmol) in dichloromethane (5 ml) was cooled down to −78 °C, treated with boron tribromide (0.34 ml, 3.5 mmol), stirred at −78 °C for 3 h and then overnight at room temperature. The mixture was cooled down to −78 °C and quenched with methanol (5 ml). After stirring for 3 h at room temperature solvents were evaporated under reduced pressure, the residue was co-evaporated four times with methanol (10 ml). The resulting precipitate was reflux in 5 ml chloroform, after cooling the product was collected by filtration and dried to provide **sery159a** (108 mg, 85 %) as a yellow powder. m.p.: 285-290 °C (dec). ^{1}H NMR (400 MHz, DMSO[D$_6$]) δ = 7.19 (d, J = 1.9 Hz, 2H), 7.08 (dd, J = 8.2, 1.9 Hz, 2H), 6.80 (d, J = 8.2 Hz, 2H), 6.74 (s, 1H). ^{13}C NMR (100.6 MHz, DMSO[D$_6$]) δ = 147.6, 147.0, 145.7, 120.0, 117.9, 115.9, 113.6, 99.1.

General Experimental Procedure for the *O*-deprotection of phenyl alkyl ethers with $AlBr_3$ (GEP 4)[6]

3-(3-Bromophenyl)-5-(2-hydroxyphenyl)-1*H*-pyrazole (sery260a)

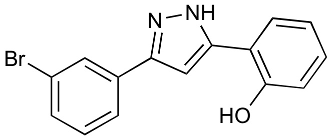

To a solution of $AlBr_3$ (4.00 g, 15 mmol) in ethanethiol (8 ml) **sery256b** (670 mg, 2.04 mmol) was added in one portion. After stirring for 12 h at room temperature the reaction mixture was slowly poured in 200 ml of 0.1N HCl, extracted with ethyl acetate (2×50 ml), the combined extracts were washed with brine (15 ml), dried (Na_2SO_4) and evaporated. The resulting precipitate was purified by recrystallisation from petroleum benzine (40-60 °C)/chloroform to provide **sery260a** as a white solid. Yield 97%. m.p.: 147-149 °C. ^{1}H NMR (400 MHz, DMSO-d$_6$ + 1-2% conc. DCl) δ = 10.50 (s, 1H), 8.06 (s, 1H), 7.85 (d, J = 7.8 Hz, 1H), 7.73 (d, J = 7.2 Hz, 1H), 7.54 (d, J = 7.2 Hz, 1H), 7.42 (t, J = 7.8 Hz, 1H), 7.31 (s, 1H), 7.20 (t, J = 7.8 Hz, 1H), 6.97 (d, J = 8.0 Hz, 1H), 6.91 (t, J = 7.8 Hz, 1H). ^{13}C NMR (100.6 MHz, DMSO-d$_6$ + 1-2 % conc. DCl) δ = 154.7, 147.0-145.0 (2C), 134.0, 131.1, 130.8, 130.7, 129.3, 127.8, 127.3, 124.3, 122.4, 119.4, 116.6, 101.3. HRMS (m/z) [M+H]$^+$ calcd for M = $C_{15}H_{11}{}^{79}BrN_2O$, 315.0133; found, 315.0138.

General Experimental Procedure for the synthesis of 1,3-diaryl-2-propene-1-ones (GEP 5)[7]

(*E*)-3-(3-Chlorophenyl)-1-(3-nitrophenyl)-2-propene-1-one (sery273a)

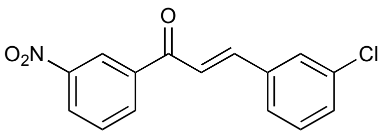

A solution of 1-(3-nitrophenyl)ethanone (1.8 g, 10 mmol), 3-chlorobenzaldehyde (1.4 g, 10 mmol), NaOH (50 mg, 1.25 mmol) and $Ba(OH)_2$•$8H_2O$ (100 mg, 0.32 mmol) in methanol (10 ml) was stirred at room temperature for 24h. The reaction mixture was cooled to +4 °C, a resulting precipitate was collected by filtration and purified by recrystallisation from ethanol/EtOAc to provide **sery273a** (2.22 g) as a yellow powder. Yield 77%. m.p.: 164-165 °C. ^{1}H NMR (400 MHz, DMSO-d$_6$) δ = 8.84 (t, J = 1.9 Hz, 1H), 8.62 (dt, J = 7.9, 1.4 Hz, 1H), 8.50 (ddd, J = 8.1, 2.3, 0.9 Hz, 1H), 8.15-8.07 (m, 2H), 7.91-7.84 (m, 2H), 7.80 (d, J = 15.6 Hz, 1H), 7.53-7.46 (m, 2H). ^{13}C NMR (100.6 MHz, DMSO-d$_6$) δ = 187.4, 148.2, 143.8, 138.5, 136.7, 134.8, 133.8, 130.6, 130.4, 128.3, 128.2, 128.1, 127.5, 123.0, 122.9. HRMS (m/z) [M+H]$^+$ calcd for M = $C_{15}H_{10}ClNO_3$, 288.0427; found, 288.0422.

General Experimental Procedure for the synthesis of 1,3-diaryl-2,3-dibromopropane-1-ones (GEP 6)[8]

3-(3-Chlorophenyl)-2,3-dibromo-1-(3-nitrophenyl)propane-1-one (sery273b)

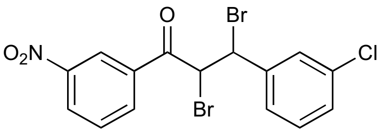

To a stirred solution of **sery273a** (1000 mg, 3.5 mmol) in chloroform (25 ml) was added dropwise a solution of bromine (560 mg, 3.5 mmol) in chloroform (5 ml) at 0°C. After being stirred for 2 h at 0°C the reaction mixture was diluted with petroleum benzin (50 ml) and the mixture was refrigerated (-24°C) for 10 hours, a resulting precipitate was collected by filtration, washed with *n*-hexane (10 ml) and dried to provide **sery273b** (1275 mg) as a white powder. Yield 82%. m.p.: 167-170 °C. ^{1}H NMR (400 MHz, DMSO-d_6) δ = 9.00 (t, *J* = 2.0 Hz, 1H), 8.72 (d, *J* = 8.0 Hz, 1H), 8.60 (dd, *J* = 8.1, 2.2 Hz, 1H), 8.05 (s, 1H), 7.97 (t, *J* = 8.0 Hz, 1H), 7.82 (dt, *J* = 6.7, 2.1 Hz, 1H), 7.51-7.45 (m, 2H), 6.91 (d, *J* = 11.2 Hz, 1H), 5.88 (d, *J* = 11.2 Hz, 1H). ^{13}C NMR (100.6 MHz, DMSO-d_6) δ = 190.0, 148.5, 140.8, 135.1, 134.7, 133.3, 131.1, 130.5, 129.1, 128.9, 128.6, 127.7, 123.4, 48.7, 46.0.

General Experimental Procedure for the synthesis of 3,5-diaryl-1*H*-pyrazoles from 1,3-diaryl-2,3-dibromopropane-1-ones (GEP 7)[9]

3-(3-Chlorophenyl)-5-(3-nitrophenyl)-1*H*-pyrazole (sery275a)

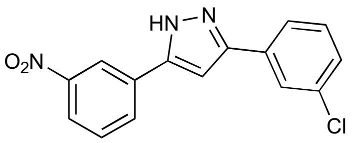

A solution of **sery273a** (1.0 g, 2.2 mmol) and hydrazine hydrate (773 mg, 15.0 mmol) in ethanol (20 ml) was heated under reflux 24 h with stirring. The clear solution was evaporated under reduced pressure, water was added and resulting precipitate was collected by filtration, washed with water and purified by recrystallisation from MeOH to provide **sery275a** (450 mg) as a white powder. Yield 67%. m.p.: 255 °C. ^{1}H NMR (400 MHz, DMSO[D_6]) δ = 13.72 (s, 1H), 8.66 (s, 1H), 8.28 (d, *J* = 7.5 Hz, 1H), 8.18 (d, *J* = 8.1 Hz, 1H), 7.93 (s, 1H), 7.82 (d, *J* = 7.8 Hz, 1H), 7.75 (t, *J* = 8.0 Hz, 1H), 7.54 (s, 1H), 7.50 (t, *J* = 7.8 Hz, 1H), 7.41 (d, *J* = 7.8 Hz, 1H). ^{13}C NMR (100.6 MHz, DMSO[D_6]) δ= 149.9, 148.8, 143.0, 135.7, 135.6, 134.2, 131.7, 131.3, 130.9, 128.3, 125.2, 124.1, 122.7, 119.8, 101.8. HRMS (*m*/*z*) $[M+H]^+$ calcd for M = $C_{15}H_{10}ClN_3O_2$, 300.0540; found, 300.0542.

General Experimental Procedure for the synthesis of 3,5-diaryl-1*H*-pyrazoles containing amino group (GEP 8)[10]

3-(3-Aminophenyl)-5-(3-chlorophenyl)-1*H*-pyrazole (sery275b)

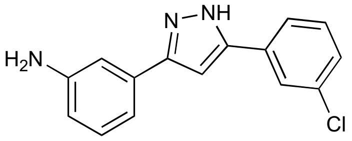

The flask was charged with ethanol (5 mL) and iron powder (186 mg, 3.3 mmol) was added in portions under efficient stirring, followed by concentrated HCl (28 ul, 0.3 mmol). The suspension was stirred at 60 °C for 2 h, 25% aqueous ammonium chloride solution (0.5 mL) was added followed by **sery275a**. The reaction mixture was stirred at 60 °C for an additional 3 h and was cooled down. Ethanol (20 mL) was added, the reaction mixture was filtered over a pad of Celite (15 g) with suction. The filter cake was washed with EtOH (10 mL) and the filtrate was concentrated under reduced pressure. A crude product was purified by recrystallization from EtOH/ petroleum benzine (40-60 °C) to provide **sery275b** (96 mg) as a white powder. Yield 53%. m.p.: 191-192 °C. ^{1}H NMR (400 MHz, DMSO[D_6]) δ= 13.3 (bs, 1H), 7.89 (s, 1H), 7.80 (d, *J* = 7.0 Hz, 1H), 7.46 (t, *J* = 7.7 Hz, 1H), 7.37 (d, *J* = 7.2 Hz, 1H), 7.16-6.86 (m, 4H), 6.56 (d, *J* = 7.2 Hz, 1H), 5.16 (s, 2H). ^{13}C NMR (100.6 MHz, DMSO[D_6]) δ= 149.6, 148.7, 144.5, 135.9, 133.7, 130.8, 129.7, 129.5, 127.4, 124.7, 123.7, 114.2, 113.4, 110.9, 99.9. HRMS (*m*/*z*) $[M+H]^+$ calcd for M = $C_{15}H_{12}ClN_3$, 270.0798; found, 270.0800.

1-(1,3-Benzodioxol-5-yl)-3-phenylpropane-1,3-dione (anle125)

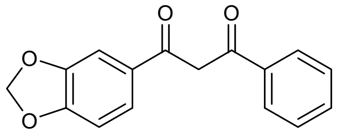

The title compound has been reported previously[11] and is commercially available (CAS 26036-40-8). It was prepared according to the GEP 1 using 1-(1,3-benzodioxol-5-yl)ethanone and methyl benzoate and purified by recrystallisation from ethanol/water to provide **anle125** as a yellow solid. Yield 62%. m.p.: 84-85 °C. ^{1}H NMR (400 MHz, DMSO[D_6]) δ= 8.15 (d, *J* = 7.5, Hz, 2H), 7.82 (dd, *J* = 8.1, 1.7 Hz, 1H), 7.73 (d, *J* = 1.3 Hz, 1H), 7.66-7.59 (m, 1H), 7.55 (t, *J* = 7.5 Hz, 2H), 7.26 (s, 1H), 7.09 (d, *J* = 8.2 Hz, 1 H), 6.16 (s, 2H) (in ^{1}H NMR spectrum 9% of diketone form is present).

3-(1,3-Benzodioxol-5-yl)-5-phenyl-1*H*-pyrazole (anle126)

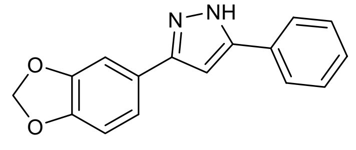

The title compound has been reported previously[12, 13] (CAS 37060-27-8). It was prepared according to the GEP 2 starting from **anle125**. White solid. Yield 70%. m.p.: 195-196 °C (lit[13] 192 °C). ^{1}H NMR (400 MHz, DMSO[D_6]) δ = 7.80 (d, J = 7.6, Hz, 2H), 7.43 (t, J = 7.5 Hz, 2H), 7.37 (d, J = 1.3 Hz, 1H), 7.32 (t, J = 7.5 Hz, 2H), 7.08 (s, 1H), 6.98 (d, J = 8.2 Hz, 1 H), 6.05 (s, 2H).

1-(3-Flurophenyl)-3-(3,4,5-trimethoxyphenyl)propane-1,3-dione (anle136a)

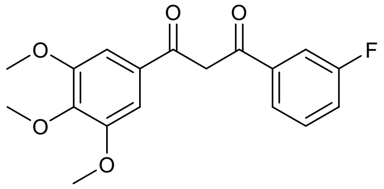

The title compound was prepared according to the GEP 1 using 1-(3,4,5-trimethoxyphenyl)ethanone and ethyl 3-fluorobenzoate and purified by recrystallisation from ethanol/water to provide **anle136a** as a yellow solid. Yield 95%. m.p.: 95-96 °C. ^{1}H NMR (400 MHz, DMSO[D_6]) δ = 8.05-7.95 (m, 2H), 7.66-7.58 (m, 1H), 7.49 (td, J = 8.6, 2.4 Hz, 1H), 7.43 (s, 2H), 7.31 (s, 1H), 3.89 (s, 6H), 3.76 (s, 3H) (in ^{1}H NMR spectrum 7% of diketone form is present). ^{13}C NMR (100.6 MHz, DMSO[D_6]) δ = 186.4, 182.5, 162.5 (d, J = 244.3 Hz), 153.2, 142.2, 137.7 (d, J = 7.2 Hz), 131.1 (d, J = 8.2 Hz), 130.0, 123.7, 119.8 (d, J = 21.4 Hz), 114.2 (d, J = 23.0 Hz), 105.4, 93.7, 60.4, 56.3. HRMS (m/z) [M+H]$^+$ calcd for M = $C_{18}H_{17}FO_5$, 333.1138; found, 333.1132.

3-(3-Fluorophenyl)-5-(3,4,5-trimethoxyphenyl)-1*H*-pyrazole (anle136b)

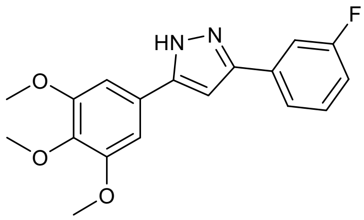

The title compound was prepared according to the GEP 2 starting from **anle136a**. White solid. Yield 88%. m.p.: 110-113 °C. ^{1}H NMR (400 MHz, DMSO[D_6]) δ = 7.68 (d, J = 7.8, Hz, 1H), 7.63 (dt, J = 10.6, 2.0 Hz, 1H), 7.48 (m, 1H), 7.29 (s, 1H), 7.18-7.10 (m, 3H), 3.85 (s, 6H), 3.68 (s, 3 H). ^{13}C NMR (100.6 MHz, DMSO[D_6]) δ = 162.8 (d, J = 242.6 Hz), 153.5, 147.6, 146.9, 137.6, 134.9, 131.1 (d, J = 8.5 Hz), 126.5, 121.3, 114.5 (d, J = 20.9 Hz), 111.9 (d, J = 22.8 Hz), 102.9, 100.6, 60.4, 56.3. HRMS (m/z) [M+H]$^+$ calcd for M = $C_{18}H_{17}FN_2O_3$, 329.1301; found, 329.1295.

3-(3-Fluorophenyl)-5-(3,4,5-trihydroxyphenyl)-1*H*-pyrazole (anle136c)

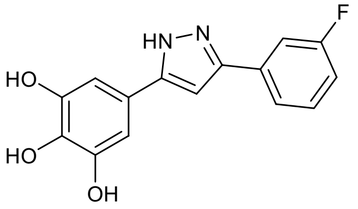

The title compound was prepared according to the GEP 3 starting from **anle136b**. White solid. Yield 100%. m.p.: 224-227 °C. ^{1}H NMR (400 MHz, DMSO[D_6]) δ = 7.68 (d, J = 7.9 Hz, 1H), 7,64 (dt, J = 10.5, 2.1 Hz, 1H), 7.50-7.42 (m, 1H), 7.14 (td, = 8.4, 2.3 Hz, 1H), 7.00 (s, 1H), 6.72 (s, 2H). ^{13}C NMR (100.6 MHz, DMSO[D_6]) δ = 162.8 (d, J = 242.9 Hz), 147.4, 147.0, 146.5, 134.6 (d, J = 9.0 Hz), 134.0, 131.0 (d, J = 8.5 Hz), 121.4, 120.9, 114.6 (d, J = 20.9 Hz), 111.9 (d, J = 22.7 Hz), 105.1, 99.5. HRMS (m/z) [M+H]$^+$ calcd for M = $C_{15}H_{11}FN_2O_3$, 287.0832; found, 287.0830.

1-(3,5-Diflurophenyl)-3-(3,4,5-trimethoxyphenyl)propane-1,3-dione (anle137a)

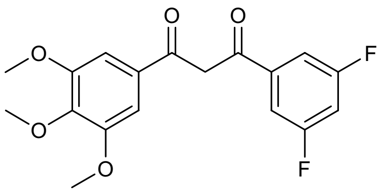

The title compound was prepared according to the GEP 1 using 1-(3,4,5-trimethoxyphenyl)ethanone and methyl 3,5-difluorobenzoate and purified by recrystallisation from ethanol/water to provide **anle137a** as a yellow solid. Yield 70%. m.p.: 125-126 °C. ^{1}H NMR (400 MHz, DMSO[D_6]) δ = 7.91-7.83 (m, 2H), 7.49 (tt, J = 9.0, 2.3 Hz, 1H), 7.43 (s, 2 H), 7.32 (s, 1 H), 3.89 (s, 6H), 3.76 (s, 3H) (in ^{1}H NMR spectrum 5% of diketone form is present). ^{13}C NMR (100.6 MHz, DMSO[D_6]) δ = 186.7, 181.0, 162.8 (dd, J = 246.8, 13.1 Hz), 153.2, 142.4, 138.3, 129.7, 110.7 (d, J = 26.6 Hz), 108.2 (t, J = 26.7 Hz), 105.6, 93.9, 60.4, 56.5. HRMS (m/z) [M+H]$^+$ calcd for M = $C_{18}H_{16}F_2O_5$, 351.1044; found, 351.1039.

3-(3,5-Diflurophenyl)-5-(3,4,5-trimethoxyphenyl)-1*H*-pyrazole (anle137b)

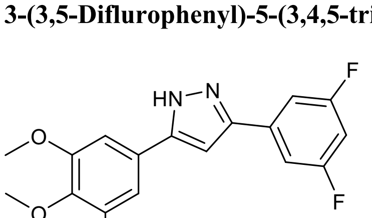

The title compound was prepared according to the GEP 2 starting from **anle137a**. White solid. Yield 100%. m.p.: 189-190 °C. ^{1}H NMR (400 MHz, DMSO[D_6]) δ = 7.59-7.49 (m, 2H), 7.36 (s, 1H), 7.17 (tt, J = 9.3, 2.3 Hz, 1H), 7.12 (s, 2 H), 3.85 (s, 6H), 3.68 (s, 3H). ^{13}C NMR (100.6 MHz, DMSO[D_6]) δ = 163.1 (dd, J = 245.2, 13.6 Hz), 153.5, 147.4, 146.2, 137.7, 132.9, 125.9, 108.2 (d, J = 26.4 Hz), 102.99 (t, J = 26.0 Hz), 102.96, 101.0, 60.4, 56.3. HRMS (m/z) [M+H]$^+$ calcd for M = $C_{18}H_{16}F_2N_2O_3$, 347.1207; found,

347.1203.

3-(3,5-Difluorophenyl)-5-(3,4,5-trihydroxyphenyl)-1*H*-pyrazole (anle137c)

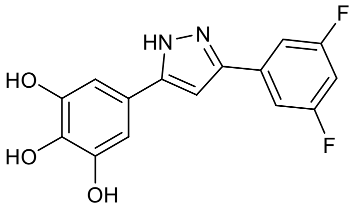

The title compound was prepared according to the GEP 3 starting from **anle137b**. White solid. Yield 100%. m.p.: 265-270 °C (dec). ^{1}H NMR (400 MHz, DMSO[D$_6$]) δ = 7.58-7.50 (m, 2H), 7.14 (tt, J = 9.3, 2.3 Hz, 1H), 7.00 (s, 1H), 6.70 (s, 2 H). ^{13}C NMR (100.6 MHz, DMSO[D$_6$]) δ = 163.0 (dd, J = 244.9, 13.5 Hz), 147.3, 146.5 (2C), 136.6, 134.0, 120.7, 108.2 (d, J = 26.3 Hz), 105.0, 102.8 (t, J = 26.0 Hz), 99.7. HRMS (m/z) [M+H]$^+$ calcd for M = $C_{15}H_{10}F_2N_2O_3$, 305.0738; found, 305.0736.

1-(1,3-Benzodioxol-5-yl)-3-(3-bromophenyl)propane-1,3-dione (anle138a)

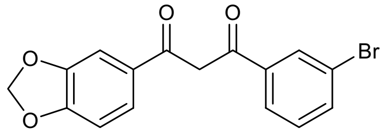

The title compound has been reported previously[12] (CAS 882696-95-9). It was prepared according to the GEP 1 using 1-(1,3-benzodioxol-5-yl)ethanone and methyl 3-bromobenzoate and purified by recrystallisation from ethanol/water to provide **anle138a** as a yellow solid. Yield 87%. m.p.: 136-137 °C. ^{1}H NMR (400 MHz, DMSO[D$_6$]) δ = 8.32 (s, 1H), 8.1 (d, J = 8.1 Hz, 1H), 7.82 (dd, J = 8.1, 1.6 Hz, 1H), 7.78 (d, J = 8.1 Hz, 1H), 7.73 (d, J = 1.6 Hz, 1H), 7.50 (t, J = 8.1 Hz, 1H), 7.27 (s, 1H), 7.05 (d, J = 8.1 Hz, 1H), 6.15 (s, 2H) (in ^{1}H NMR spectrum 6% of diketone form is present).

3-(1,3-Benzodioxol-5-yl)-5-(3-bromophenyl)-1*H*-pyrazole (anle138b)

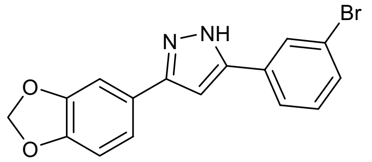

The title compound has been reported previously[12] (CAS 882697-00-9). It was prepared according to the GEP 2 starting from **anle138a**. White solid. Yield 89%. m.p.: 192-193 °C (lit[12] 128-130 °C). ^{1}H NMR (400 MHz, DMSO[D$_6$]) δ = 8.00 (s, 1H), 7.81 (d, J = 8.1 Hz, 1H), 7.50 (d, J = 8.0 Hz, 1H), 7.40 (d, J = 7.9 Hz, 1H), 7.37 (d, J = 1.5 Hz, 1H), 7.32 (d, J = 8.0 Hz, 1H), 7.19 (s, 1H), 7.00 (d, J = 8.0 Hz, 1H), 6.06 (s, 2H).

3-(3-Bromophenyl)-5-(3,4-dihydroxyphenyl)-1*H*-pyrazole (anle138c)

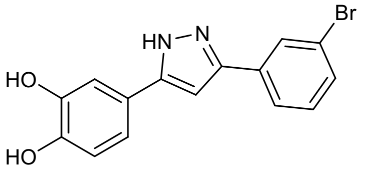

This compound was prepared according to the GEP 3 starting from **sery255b**. White solid. Yield 87%. m.p.: 232-237 °C. ^{1}H NMR (400 MHz, DMSO[D$_6$]) δ = 8.02 (s, 1H), 7.83 (d, J = 7.8 Hz, 1H), 7.50 (d, J = 7.8 Hz, 1H), 7.38 (t, J = 7.8 Hz, 1H), 7.19 (d, J = 1.9 Hz, 1H), 7.08 (dd, J = 8.2, 1.9 Hz, 1H), 7.02 (s, 1H), 6.80 (d, J = 8.2 Hz, 1H). ^{13}C NMR (100.6 MHz, DMSO[D$_6$]) δ = 147.1, 146.6, 145.9, 145.6, 134.4, 130.9, 103.5, 127.7, 124.2, 122.2, 121.5, 117.0, 115.9, 113.1, 99.3. HRMS (m/z) [M+H]$^+$ calcd for M = $C_{15}H_{11}{}^{79}BrN_2O_2$, 331.0082; found, 331.0088.

1-(3-Bromophenyl)-3-(3,4,5-trimethoxyphenyl)propane-1,3-dione (anle142a)

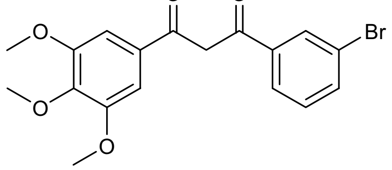

The title compound was prepared according to the GEP 1 using 1-(3,4,5-trimethoxyphenyl)ethanone and methyl 3-bromobenzoate and purified by recrystallisation from ethanol/water to provide **anle142a** as a yellow solid. Yield 99%. m.p.: 109-111 °C. ^{1}H NMR (400 MHz, DMSO[D$_6$]) δ = 8.29 (t, J = 1.8 Hz, 1H), 8.16 (d, J = 7.9 Hz, 1H), 7.83 (d, J = 7.9 Hz, 1H), 7.53 (t, J = 7.9 Hz, 1H), 7.43 (s, 2H), 7.32 (s, 1H), 3.90 (s, 6H), 3.76 (s, 3H) (in ^{1}H NMR spectrum 7% of diketone form is present). ^{13}C NMR (100.6 MHz, DMSO[D$_6$]) δ = 186.8, 182.7, 153.5, 142.8, 137.3, 135.9, 131.5, 130.3, 130.2, 126.9, 122.8, 105.9, 94.1, 60.7, 56.8. HRMS (m/z) [M+H]$^+$ calcd for M = $C_{18}H_{17}{}^{79}BrO_5$, 393.0338; found, 393.0337.

3-(3-Bromophenyl)-5-(3,4,5-trimethoxyphenyl)-1*H*-pyrazole (anle142b)

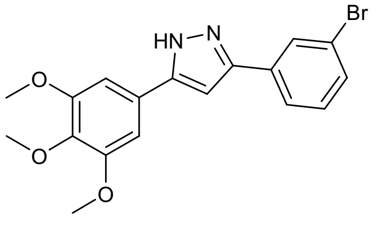

The title compound was prepared according to the GEP 2 starting from **anle142a**. White solid. Yield 92%. m.p.: 114-117 °C. ^{1}H NMR (400 MHz, DMSO[D$_6$] + 1-2% conc. DCl) δ = 8.02 (s, 1H), 7.84 (d, J = 7.8 Hz, 1H), 7.51 (d, J = 8.0 Hz, 1H), 7.40 (t, J = 7.8 Hz, 1H), 7.32 (s, 1H), 7.14 (s, 2H), 3.85 (s, 6H), 3.68 (s, 3H). ^{13}C NMR (100.6 MHz, DMSO[D$_6$] + 1-2% conc. DCl) δ = 153.5, 147.3, 146.6, 137.6, 134.8, 131.2, 130.5, 127.8, 126.4, 124.2,

122.5, 102.9, 100.6, 60.4, 56.5. HRMS (*m*/*z*) $[M+H]^+$ calcd for M = $C_{18}H_{17}{}^{79}BrN_2O_3$, 389.0501; found, 389.0503.

3-(3-Bromophenyl)-5-(3,4,5-trihydroxyphenyl)-1*H*-pyrazole hydrobromide (anle142c)

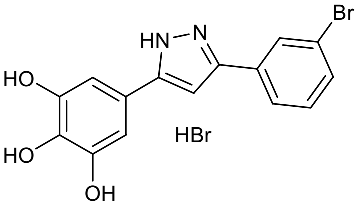

The title compound was prepared according to the GEP 3 starting from **anle142b**. Brown solid. Yield 68%. m.p.: 221-224 °C (dec). ^{1}H NMR (400 MHz, DMSO[D$_6$]) δ = 8.02 (t, *J* = 1.7 Hz, 1H), 7.83 (d, *J* = 7.8 Hz, 1H), 7,49 (d, *J* = 8.0 Hz, 1H), 7.36 (t, *J* = 7.8 Hz, 1H), 6.95 (s, 1H), 6.72 (s, 2H). ^{13}C NMR (100.6 MHz, DMSO[D$_6$]) δ = 147.2, 146.8, 135.3, 134.2, 131.4, 130.7, 128.0, 125.8, 124.6, 122.7, 121.4, 105.3, 99.6. HRMS (*m*/*z*) $[M+H]^+$ calcd for M = $C_{15}H_{11}{}^{79}BrN_2O_3$, 347.0031; found, 347.0035.

1-(3,4-Dimethoxyphenyl)-3-(3,4,5-trimethoxyphenyl)propane-1,3-dione (anle143a)

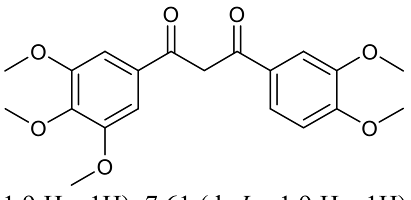

The title compound was prepared according to the GEP 1 using 1-(3,4,5-trimethoxyphenyl)ethanone and ethyl 3,4-dimethoxybenzoate, and purified by column chromatography (hexane:EtOAc, 1:1 v/v) followed by recrystallisation from methanol/water to provide **anle143a** as a yellow solid. Yield 29%. TLC (hexane:EtOAc, 1:1 v/v): RF = 0.57. m.p.: 48-51 °C. ^{1}H NMR (400 MHz, DMSO[D$_6$]) δ = 7.88 (dd, *J* = 8.4, 1.9 Hz, 1H), 7.61 (d, *J* = 1.9 Hz, 1H), 7.39 (s, 2H), 7.21 (s, 1H), 7.09 (d, *J* = 8.6 Hz, 1H), 3.89 (s, 6H), 3.86 (s, 3H), 3.85 (s, 3H), 3.75 (s, 3H) (in ^{1}H NMR spectrum 11% of diketone form is present). ^{13}C NMR (100.6 MHz, DMSO[D$_6$]) δ = 185.5, 183.6, 153.3, 153.1, 148,9, 141.7, 130.2, 127.4, 122.2, 111.4, 110.3, 105.1, 92.7, 60.4, 56.4, 56.0, 55.9. HRMS (*m*/*z*) $[M+H]^+$ calcd for M = $C_{20}H_{22}O_7$, 375.1444; found, 375.1442.

3-(3,4-Dimethoxyphenyl)-5-(3,4,5-trimethoxyphenyl)-1*H*-pyrazole (anle143b)

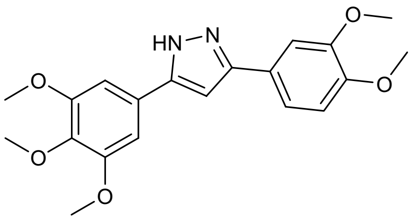

The title compound has been reported previously[14] (CAS 861881-81-4). It was prepared according to the GEP 2 starting from **anle143a**. White solid. Yield 69%. m.p.: 91-93 °C (lit. 68 °C). ^{1}H NMR (400 MHz, DMSO[D$_6$] + 1-2% conc. DCl) δ = 7.40 (d, *J* = 1.9 Hz, 1H), 7.36 (dd, *J* = 8.2, 1.9 Hz, 1H), 7.12 (s, 3H), 7.02 (d, *J* = 8.4 Hz, 1H), 3.85 (s, 6H), 3.84 (s, 3H), 3.78 (s, 3H), 3.68 (s, 3H).

3-(3,4-Dihydroxyphenyl)-5-(3,4,5-trihydroxyphenyl)-1*H*-pyrazole hydrobromide (anle143c)

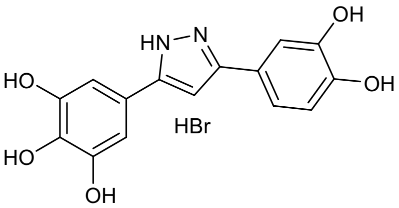

The title compound was prepared according to the GEP 3 starting from **anle143b**. Brown solid. Yield 81%. m.p.: 225-230 °C (dec). ^{1}H NMR (400 MHz, DMSO[D$_6$] + 1-2% conc. DCl) δ = 9.57-8.57 (m, 5H), 8.53-7.97 (bs, 1H), 7.17 (s, 1H), 7.05 (dd, *J* = 8.1, 1.7 Hz, 1H), 6.76 (d, *J* = 8.1 Hz, 1H), 6.70 (s, 2H), 6.57 (s, 1H). ^{13}C NMR (100.6 MHz, DMSO[D$_6$] + 1-2% conc. DCl) δ = 148.1, 146.4, 145.5, 145.4, 142.4, 133.4, 123.3, 122.0, 116.8, 115.9, 113.0, 104.8, 97.7. HRMS (*m*/*z*) $[M+H]^+$ calcd for M = $C_{15}H_{12}N_2O_5$, 301.0824; found, 301.0823.

1-(3,5-Dibromophenyl)-3-(3,4,5-trimethoxyphenyl)propane-1,3-dione (anle145a)

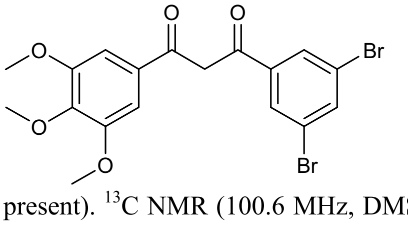

The title compound was prepared according to the GEP 1 using 1-(3,4,5-trimethoxyphenyl)ethanone and methyl 3,5-dibromobenzoate and purified by recrystallisation from ethanol/water to provide **anle145a** as a yellow solid. Yield 90%. m.p.: 126-129 °C. ^{1}H NMR (400 MHz, DMSO[D$_6$]) δ = 8.28 (d, *J* = 1.7 Hz, 2H), 8.10 (t, *J* = 1.7 Hz, 1H), 7.44 (s, 2H), 7.33 (s, 1H), 3.90 (s, 6H), 3.77 (s, 3H) (in ^{1}H NMR spectrum 6% of diketone form is present). ^{13}C NMR (100.6 MHz, DMSO[D$_6$]) δ = 186.8, 180.7, 153.2, 142.5, 138.4, 137.3, 129.8, 129.1, 123.3, 105.8, 94.2, 60.4, 56.6. HRMS (*m*/*z*) $[M+H]^+$ calcd for M = $C_{18}H_{16}{}^{79}Br^{81}BrO_5$, 472.9422; found, 472.9423.

3-(3,5-Dibromophenyl)-5-(3,4,5-trimethoxyphenyl)-1*H*-pyrazole (anle145b)

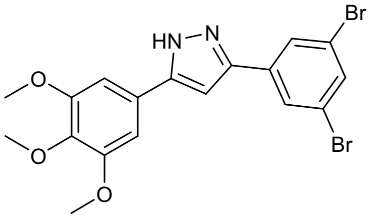

The title compound was prepared according to the GEP 2 starting from **anle145a**. White solid. Yield 99%. m.p.: 138-141 °C. ^{1}H NMR (400 MHz,

DMSO[D_6] + 1-2% conc. DCl) δ = 8.04 (d, J = 1.7 Hz, 2H), 7.77 (t, J = 1.7 Hz, 1H), 7.42 (s, 1H), 7.14 (s, 2H), 3.85 (s, 6H), 3.68 (s, 3H). ^{13}C NMR (100.6 MHz, DMSO[D_6] + 1-2% conc. DCl) δ = 153.8, 147.3, 137.9, 133.4, 132.6, 131.6, 131.0, 127.0, 123.5, 103.1, 101.3, 60.6, 56.5. HRMS (*m*/*z*) [M+H]$^+$ calcd for M = $C_{18}H_{16}{}^{79}Br^{81}BrN_2O_3$, 468.9585; found, 468.9586.

3-(3,5-Dibromophenyl)-5-(3,4,5-trihydroxyphenyl)-1*H*-pyrazole hydrobromide (anle145c)

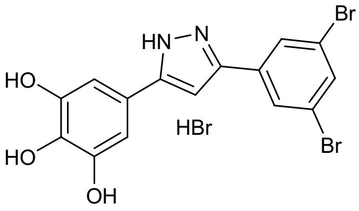

The title compound was prepared according to the GEP 3 starting from **anle145b**. Beige solid. Yield 100%. m.p.: 285-290 °C (dec). ^{1}H NMR (400 MHz, DMSO[D_6]) δ = 8.04 (d, J = 1.8 Hz, 2H), 7.74 (t, J = 1.8 Hz, 1H), 7.06 (s, 1H), 6.71 (s, 2H). ^{13}C NMR (100.6 MHz, DMSO[D_6]) δ = 146.7-146.5 (3C), 136.9, 134.1, 132.3, 126.9, 123.2, 120.7, 105.1, 100.0. HRMS (*m*/*z*) [M+H]$^+$ calcd for M = $C_{15}H_{11}{}^{79}Br^{81}BrN_2O_3$, 426.9116; found, 426.9119.

3-(3,5-Dibromophenyl)-5-(3,4,5-trihydroxyphenyl)-1*H*-pyrazole (anle145d)

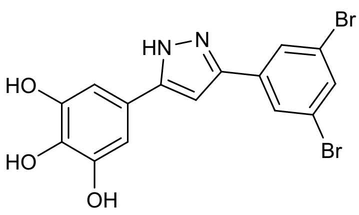

Anle145c (171 mg, 337 µmol) was partioned between ethyl acetate (5 ml) and 0.2 M phosphate buffer pH = 7.5 (10 ml). The organic layer was separated and the aqueous layer was extracted with ethyl acetate (2 × 5 ml). The combined organic layers were dried over Na_2SO_4 and concentrated in vacuo. The residue was stirred 30 min with water (5 ml), the resulting precipitate was collected by filtration, washed with water (2 × 3 ml) and dried in vacuo to provide **anle145d** (106 mg, 73 %) as a brown solid. m.p.: 290-295 °C (dec). The observed characterisation data (^{1}H-, ^{13}C-NMR in DMSO[D_6] + 1-2% conc. DCl; HRMS) was consistent with that of compound **anle145c**.

1-(1,3-Benzodioxol-5-yl)-3-(3-fluorophenyl)propane-1,3-dione (anle186a)

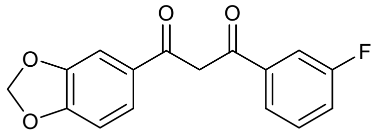

The title compound was prepared according to the GEP 1 using 1-(1,3-benzodioxol-5-yl)ethanone and ethyl 3-fluorobenzoate and purified by recrystallisation from ethanol/water to provide **anle186a** as a yellow solid. Yield 89%. m.p.: 120-122 °C. ^{1}H NMR (400 MHz, DMSO[D_6]) δ = 8.05-7.93 (m, 2H), 7.83 (dd, J = 8.2, 1.8 Hz, 1H), 7.75 (d, J = 1.7 Hz, 1H), 7.64-7.55 (m, 1H), 7.50-7.42 (m, 1H), 7.29 (s, 1H), 7.08 (d, J = 8.2 Hz, 1H), 6.16 (s, 2H) (in ^{1}H NMR spectrum 3% of diketone form is present). ^{13}C NMR (100.6 MHz, DMSO[D_6]) δ = 186.3, 181.6, 162.5 (d, J = 244.2 Hz), 151.9, 148.2, 137.1 (d, J = 7.3 Hz), 131.1 (d, J = 8.2 Hz), 129.0, 124.0, 123.5, 119.6 (d, J = 21.2 Hz), 114.0 (d, J = 23.1 Hz), 108.6, 107.4, 102.4, 93.3. HRMS (*m*/*z*) [M+H]$^+$ calcd for M = $C_{16}H_{11}FO_4$, 287.0720; found, 287.0718.

3-(1,3-Benzodioxol-5-yl)-5-(3-fluorophenyl)-1*H*-pyrazole (anle186b)

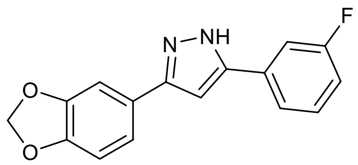

The title compound was prepared according to the GEP 2 starting from **anle186a**. White solid. Yield 86%. m.p.: 191-192 °C. ^{1}H NMR (400 MHz, DMSO[D_6] + 1-2% conc. DCl) δ = 7.64 (d, J = 7.8 Hz, 1H), 7.63-7.58 (m, 1H), 7.51-7.44 (m, 1H), 7.36 (d, J = 1.6 Hz, 1H), 7.31 (dd, J = 8.1, 1.6 Hz, 1H), 7.19-7.10 (m, 1H), 7.16 (s, 1H), 6.99 (d, J = 8.0 Hz, 1H), 6.05 (s, 2H). ^{13}C NMR (100.6 MHz, DMSO[D_6] + 1-2% conc. DCl) δ = 162.8 (d, J = 242.7 Hz), 148.0, 147.3, 135.0, 131.0 (d, J = 8.6 Hz), 129.7, 126.3, 124.4, 121.3, 119.1, 114.5 (d, J = 21.2 Hz), 111.8 (d, J = 22.8 Hz), 108.9, 105.8, 101.4, 100.0. HRMS (*m*/*z*) [M+H]$^+$ calcd for M = $C_{16}H_{11}FN_2O_2$, 283.0883; found, 283.0883.

1-(1,3-Benzodioxol-5-yl)-3-(3-iodophenyl)propane-1,3-dione (anle197a)

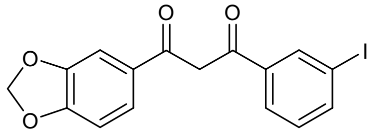

The title was prepared according to the GEP 1 using 1-(1,3-benzodioxol-5-yl)ethanone and methyl 3-iodobenzoate and purified by recrystallisation from ethanol/water to provide **anle197a** as a yellow solid. Yield 22%. m.p.: 143-144 °C. ^{1}H NMR (400 MHz, DMSO[D_6]) δ = 8.49 (t, J = 1.6 Hz, 1H), 8.14 (d, J = 7.9 Hz, 1H), 7.97 (d, J = 7.9 Hz, 1H), 7.84 (dd, J = 8.2, 1.8 Hz, 1H), 7.76 (d, J = 1.7 Hz, 1H), 7.34 (t, J = 7.8 Hz, 1H), 7.28 (s, 1H), 7.08 (d, J = 8.2 Hz, 1H), 6.16 (s, 2H) (in ^{1}H NMR spectrum 7% of diketone form is present). ^{13}C NMR (100.6 MHz, DMSO[D_6]) δ = 186.2, 181.6, 151.9,

148.2, 141.3, 136.7, 135.5, 131.0, 129.0, 126.7, 124.1, 108.6, 107.5, 102.3, 95.4, 93.2. HRMS (*m*/*z*) $[M+H]^+$ calcd for M = $C_{16}H_{11}IO_4$, 394.9780; found, 394.9788.

3-(1,3-Benzodioxol-5-yl)-5-(3-iodophenyl)-1*H*-pyrazole (anle197b)

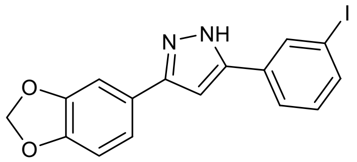

The title compound was prepared according to the GEP 2 starting from **anle197a**. White solid. Yield 75%. m.p.: 205-207 °C. ^{1}H NMR (400 MHz, DMSO[D_6] + 1-2% conc. DCl) δ = 8.18 (t, *J* = 1.6 Hz, 1H), 7.82 (d, *J* = 8.0 Hz, 1H), 7.67 (d, *J* = 8.2 Hz, 1H), 7.36 (d, *J* = 1.6 Hz, 1H), 7.32 (dd, *J* = 8.0, 1.7 Hz, 1H), 7.23 (t, *J* = 7.8 Hz, 1H), 7.16 (s, 1H), 6.99 (d, *J* = 8.0 Hz, 1H), 6.05 (s, 2H). ^{13}C NMR (100.6 MHz, DMSO[D_6] + 1-2% conc. DCl) δ = 148.0, 147.3, 146.8 (2C), 136.4, 134.4, 133.5, 131.1, 125.2, 124.5, 119.1, 108.9, 105.8, 101.4, 99.9, 95.5. HRMS (*m*/*z*) $[M+H]^+$ calcd for M = $C_{16}H_{11}IN_2O_2$, 390.9944; found, 390.9948.

1,3-Bis(1,3-benzodioxol-5-yl)-propane-1,3-dione (anle232a)

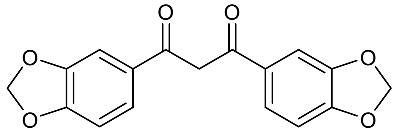

The title compound has been reported previously[15] (CAS 204397-06-8). It was prepared according to the GEP 1 using 1-(1,3-benzodioxol-5-yl)ethanone and methyl 1,3-benzodioxole-5-carboxylate and purified by recrystallisation from ethanol/water to provide **anle232a** as a yellow solid. Yield 71%. m.p.: 179-180 °C (lit[15] 185-186 °C). ^{1}H NMR (400 MHz, DMSO[D_6]) δ = 7.78 (dd, *J* = 8.2, 1.7 Hz, 2H), 7.73 (d, *J* = 1.7 Hz, 2H), 7.17 (s, 1H), 7.07 (d, *J* = 8.2 Hz, 2H), 6.15 (s, 4H) (in ^{1}H NMR spectrum 11% of diketone form is present).

3,5-Bis(1,3-benzodioxol-5-yl)-1*H*-pyrazole (anle232b)

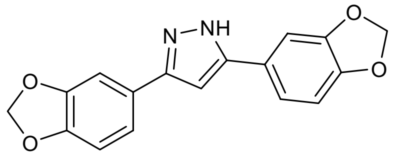

The title compound has been reported previously[16] (CAS 1147315-26-1). It was prepared according to the GEP 2 starting from **anle232a**. Beige solid. Yield 71%. m.p.: 228-229 °C. ^{1}H NMR (400 MHz, DMSO[D_6]) δ = 7.34 (s, 2H), 7.30 (d, *J* = 8.0 Hz, 2H), 7.00 (s, 1H), 6.98 (d, *J* = 8.0 Hz, 2H), 6.04 (s, 4H).

1-(1,3-Benzodioxol-5-yl)-3-(3-methoxyphenyl)propane-1,3-dione (anle233a)

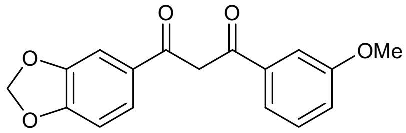

The title compound has been reported previously[12] (CAS 882696-96-0). It was prepared according to the GEP 1 using 1-(1,3-benzodioxol-5-yl)ethanone and ethyl 3-methoxybenzoate and purified by recrystallisation from ethanol/water to provide **anle233a** as a yellow solid. Yield 68%. m.p.: 86-88 °C. ^{1}H NMR (400 MHz, DMSO[D_6]) δ = 7.82 (dd, *J* = 8.2, 1.7 Hz, 1H), 7.78-7.71 (m, 2H), 7.63 (t, *J* = 2.0 Hz, 1H), 7.46 (t, *J* = 8.0 Hz, 1H), 7.24 (s, 1H), 7.19 (dd, *J* = 8.0, 2.4 Hz, 1H), 7.09 (d, J = 8.2 Hz, 1H), 6.16 (s, 2H), 3.85 (s, 3H).

3-(1,3-Benzodioxol-5-yl)-5-(3-methoxyphenyl)-1*H*-pyrazole (anle233b)

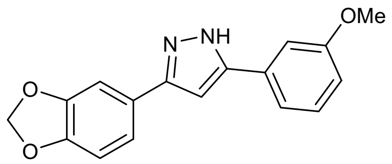

The title compound has been reported previously[12] (CAS 882697-01-0). It was prepared according to the GEP 2 starting from **anle233a**. White solid. Yield 79%. m.p.: 160-161 °C (lit[12] 149 °C) . ^{1}H NMR (400 MHz, DMSO[D_6] + 1-2% conc. DCl) δ = 7.46-7.29 (m, 5H), 7.11 (s, 1H), 6.98 (d, *J* = 8.0 Hz, 1H), 6.89 (br d, *J* = 7.3Hz, 1H), 6.05 (s, 2H), 3.81 (s, 3H).

1-(1,3-Benzodioxol-5-yl)-3-(2-bromophenyl)propane-1,3-dione (anle234a)

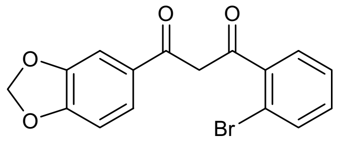

The title was prepared according to the GEP 1 using 1-(1,3-benzodioxol-5-yl)ethanone and methyl 2-bromobenzoate and purified by recrystallisation from ethanol/water to provide **anle234a** as a yellow solid. Yield 82%. m.p.: 73-76 °C. ^{1}H NMR (400 MHz, DMSO[D_6]) δ = 7.76 (dd, *J* = 7.9, 1.0 Hz, 1H), 7.71 (dd, *J* = 8.2, 1.7 Hz, 1H), 7.67 (dd, *J* = 7.6, 1.7 Hz, 1H), 7.59 (d, *J* = 1.7 Hz, 1H), 7.52 (dt, *J* = 7.4, 1.0 Hz, 1H), 7.45 (dt, *J* = 7.6, 1.7 Hz, 1H), 7.07 (d, *J* = 8.2 Hz, 1H), 6.81 (s, 1H), 6.15 (s, 2H) (in ^{1}H NMR spectrum 4% of diketone form is present). ^{13}C NMR (100.6 MHz, DMSO[D_6]) δ = 186.7, 184.6, 151.9, 148.3, 137.6, 133.8, 132.5, 130.4, 128.4, 128.1, 123.9, 119.7, 108.7, 107.1, 102.4, 97.6. HRMS (*m*/*z*) $[M+H]^+$ calcd for M = $C_{16}H_{11}{}^{79}BrO_4$, 346.9919; found, 346.9921.

3-(1,3-Benzodioxol-5-yl)-5-(2-bromophenyl)-1*H*-pyrazole (anle234b)

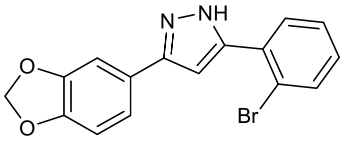

The title compound was prepared according to the GEP 2 starting from **anle234a**. White solid. Yield 60%. m.p.: 170 °C. ^{1}H NMR (400 MHz, DMSO[D$_6$] + 1-2% conc. DCl) δ = 7.73 (d, J = 7.9 Hz, 1H), 7.63 (d, J = 7.4 Hz, 1H), 7.46 (t, J = 7.4 Hz, 1H), 7.38 (s, 1H), 7.35-7.25 (m, 2H), 7.05-6.90 (m, 2H), 6.05 (s, 2H). ^{13}C NMR (100.6 MHz, DMSO[D$_6$] + 1-2% conc. DCl) δ = 148.0, 147.2, 146.0 (2C), 133.6, 133.1, 131.3, 130.0, 128.0, 125.4, 121.6, 119.2, 108.9, 105.9, 103.0, 101.4. HRMS (*m*/*z*) [M+H]$^+$ calcd for M = $C_{16}H_{11}{}^{79}BrN_2O_2$, 343.0082; found, 343.0087.

1-(1,3-Benzodioxol-5-yl)-3-[3-(trifluoromethyl)phenyl]propane-1,3-dione (anle236a)

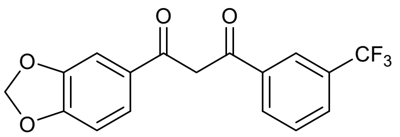

The title was prepared according to the GEP 1 using 1-(1,3-benzodioxol-5-yl)ethanone and methyl 3-(trifluoromethyl)benzoate and purified by recrystallisation from ethanol/water to provide **anle236a** as a yellow solid. Yield 83%. m.p.: 97-99 °C. ^{1}H NMR (400 MHz, DMSO[D$_6$]) δ = 8.50-8.39 (m, 2H), 7.98 (d, J = 7.7 Hz, 1H), 7.86 (dd, J = 8.2, 1.7 Hz, 1H), 7.83-7.74 (m, 2H), 7.39 (s, 1H), 7.10 (d, J = 8.2 Hz, 1H), 6.16 (s, 2H) (in ^{1}H NMR spectrum 6% of diketone form is present). ^{13}C NMR (100.6 MHz, DMSO[D$_6$]) δ = 186.6, 181.2, 152.0, 148.2, 135.7. 131.3, 130.2, 129.9 (q, J = 32.3 Hz), 129.1 (q, J = 3.6 Hz), 128.9, 124.2, 124.1 (q, J =273.3 Hz), 123.8 (q, J = 3.6 Hz), 108.6, 107.5, 102.4, 93.5. HRMS (*m*/*z*) [M+H]$^+$ calcd for M = $C_{17}H_{11}F_3O_4$, 337.0688; found, 337.0688.

3-(1,3-Benzodioxol-5-yl)-5-[3-(trifluoromethyl)phenyl]-1*H*-pyrazole (anle236b)

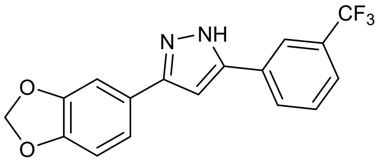

The title compound was prepared according to the GEP 2 starting from **anle236a**. White solid. Yield 90%. m.p.: 220-222 °C. ^{1}H NMR (400 MHz, DMSO[D$_6$] + 1-2% conc. DCl) δ = 8.14 (s, 1H), 8.11 (t, J = 3.6 Hz, 1H), 7.75-7.60 (m, 2H), 7.39 (d, J = 1.6 Hz, 1H), 7.34 (dd, J = 8.0, 1.6 Hz, 1H), 7.27 (s, 1H), 7.00 (d, J = 8.0 Hz, 1H), 6.06 (s, 2H). ^{13}C NMR (100.6 MHz, DMSO[D$_6$] + 1-2% conc. DCl) δ = 148.9, 148.8, 148.0, 147.4, 133.6, 130.2, 129.9 (q, J = 31.6 Hz), 129.1, 124.9, 124.5 (q, J = 272.4 Hz), 124.3 (q, J = 3.6 Hz), 121.5 (q, J = 3.7 Hz), 119.2, 109.0, 105.8, 101.5, 100.2. HRMS (*m*/*z*) [M+H]$^+$ calcd for M = $C_{17}H_{11}F_3N_2O_2$, 333.0851; found, 333.0851.

3-(3,4-dihydroxyphenyl)-5-[3-(trifluoromethyl)phenyl]-1*H*-pyrazole (anle236c)

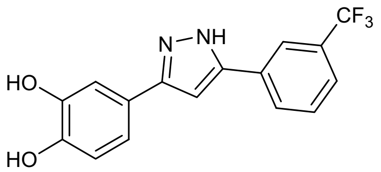

The title compound was prepared according to the GEP 3 starting from **anle236b**. Beige solid. Yield 100%. m.p.: 270-280 °C (dec). ^{1}H NMR (400 MHz, DMSO[D$_6$] + 1-2% conc. DCl) δ = 8.15 (s, 1H), 8.12 (t, J = 4.3 Hz, 1H), 7.71-7.57 (m, 2H), 7.20 (d, J = 2.0 Hz, 1H), 7.14-7.05 (m, 2H), 6.81 (d, J = 8.1 Hz, 1H). ^{13}C NMR (100.6 MHz, DMSO[D$_6$] + 1-2% conc. DCl) δ = 147.8, 146.5, 146.0, 145.8, 134.0, 130.1, 129.8 (q, J = 31.6 Hz), 129.1, 124.5 (J = 272.6 Hz), 124.1, 122.0, 121.5, 117.1, 116.1, 113.2, 99.4. HRMS (*m*/*z*) [M+H]$^+$ calcd for M = $C_{16}H_{11}F_3N_2O_2$, 321.0851; found, 321.0850.

1-(1,3-Benzodioxol-5-yl)-3-(3,5-dibromophenyl)propane-1,3-dione (anle246a)

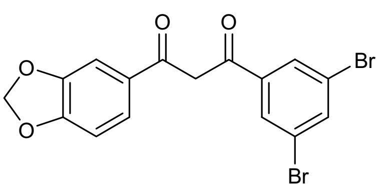

The title compound was prepared according to the GEP 1 using 1-(1,3-benzodioxol-5-yl)ethanone and methyl 3,5-dibromobenzoate and purified by recrystallisation from ethanol/water to provide **anle246a** as a yellow solid. Yield 80%. m.p.: 181-182 °C. ^{1}H NMR (400 MHz, DMSO[D$_6$]) δ = 8.31 (d, J = 1.7 Hz, 2H), 8.08 (t, J = 1.6 Hz, 1H), 7.85 (dd, J = 8.2, 1.7 Hz, 1H), 7.77 (d, J = 1.6 Hz, 1H), 7.33 (s, J = 8.2 Hz, 1H), 7.09 (d, J = Hz, 1H), 6.17 (s, 2H) (in ^{1}H NMR spectrum 5% of diketone form is present). ^{13}C NMR (100.6 MHz, DMSO[D$_6$]) δ = 186.8, 180.1, 152.4, 148.5, 138.7, 137.5, 129.3, 129.1, 124.7, 123.6, 108.9, 107.9, 102.7, 94.0. HRMS (*m*/*z*) [M+H]$^+$ calcd for M = $C_{16}H_{10}{}^{79}Br^{81}BrO_4$, 426.9004; found, 426.8998.

3-(1,3-Benzodioxol-5-yl)-5-(3,5-dibromophenyl)-1*H*-pyrazole (anle246b)

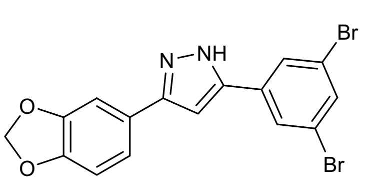

The title compound was prepared according to the GEP 2 starting from **anle246a**. White solid. Yield 95%. m.p.: 264-265 °C. ^{1}H NMR (400 MHz,

DMSO[D_6] + 1-2% conc. DCl) δ = 8.01 (d, J = 1.7 Hz, 2H), 7.75 (t, J = 1.7 Hz, 1H), 7.35 (d, J = 1.6 Hz, 1H), 7.31 (dd, J = 8.0, 1.6 Hz, 1H), 7.28 (s, 1H), 7.00 (d, J = 8.0 Hz, 1H), 6.05 (s, 2H). ^{13}C NMR (100.6 MHz, DMSO[D_6] + 1-2% conc. DCl) δ = 148.1, 147.4, 146.8-145.8 (2C), 136.5, 132.3, 126.8, 124.6, 123.3, 119.2, 109.0, 105.8, 101.5, 100.6. HRMS (*m*/*z*) [M+H]$^+$ calcd for M = $C_{16}H_{10}{}^{79}Br^{81}BrN_2O_2$, 422.9167; found, 422.9159.

1-(3-Bromophenyl)-3-[4-(dimethylamino)phenyl]propane-1,3-dione (anle253a)

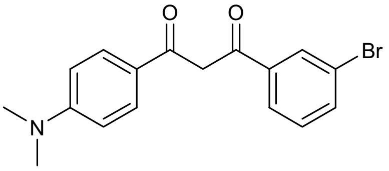

The title compound was prepared according to the GEP 1 using 1-[4-(dimethylamino)phenyl]ethanone and methyl 3-bromobenzoate and purified by recrystallisation from ethanol/water to provide **anle253a** as a yellow solid. Yield 94%. m.p.: 123-124 °C. ^{1}H NMR (400 MHz, DMSO[D_6]) δ = 8.28 (t, J = 1.7 Hz, 1H), 8.09 (d, J = 8.0 Hz, 1H), 8.05 (d, J = 9.1 Hz, 2H), 7.77 (d, J = 8.0 Hz, 1H), 7.48 (t, J = 7.9 Hz, 1H), 7.20 (s, 1H), 6.76 (d, J = 9.1 Hz, 2H), 3.05 (s, 6H) (in ^{1}H NMR spectrum 6% of diketone form is present). ^{13}C NMR (100.6 MHz, DMSO[D_6]) δ = 189.9, 179.5, 153.8, 137.5, 134.8, 131.0, 130.0, 129.5, 126.0, 122.4, 121.2, 111.2, 92.3, 39.8. HRMS (*m*/*z*) [M+H]$^+$ calcd for M = $C_{17}H_{16}{}^{79}BrNO_2$, 346.0443; found, 346.0437.

3-(3-Bromophenyl)-5-[4-(dimethylamino)phenyl]-1*H*-pyrazole (anle253b)

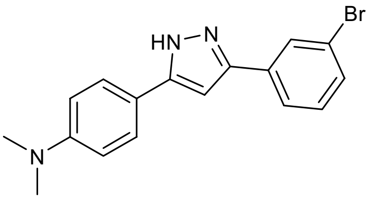

The title compound was prepared according to the GEP 2 starting from **anle253a**. White solid. Yield 85%. m.p.: 185-187 °C. ^{1}H NMR (400 MHz, DMSO[D_6]) δ = 8.01 (s, 1H), 7.83 (d, J = 7.2 Hz, 1H), 7.61 (d, J = 7.4 Hz, 2H), 7.48 (d, J = 7.2 Hz, 1H), 7.37 (t, J = 7.2 Hz, 1H), 7.04 (s, 1H), 6.76 (d, J = 7.4 Hz, 2H), 2.92 (s, 6H). ^{13}C NMR (100.6 MHz, DMSO[D_6]) δ = 150.4, 149.8, 144.6, 136.6, 131.1, 130.1, 127.7, 126.3, 124.2, 122.4, 117.1, 112.5, 98.3, 40.1. HRMS (*m*/*z*) [M+H]$^+$ calcd for M = $C_{17}H_{16}{}^{79}BrN_3$, 342.0606; found, 342.0611.

1-(3-Chlorophenyl)-3-[4-(dimethylamino)phenyl]propane-1,3-dione (anle254a)

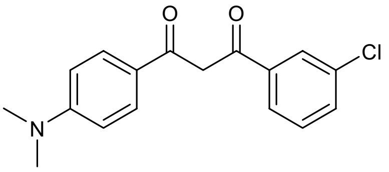

The title compound was prepared according to the GEP 1 using 1-[4-(dimethylamino)phenyl]ethanone and methyl 3-chlorobenzoate and purified by recrystallisation from ethanol/water to provide **anle254a** as a yellow solid. Yield 85%. m.p.: 120-122 °C. ^{1}H NMR (400 MHz, DMSO[D_6]) δ = 8.15 (t, J = 1.6 Hz, 1H), 8.05 (d, J = 9.0 Hz, 3H), 7.64 (d, J = 7.9 Hz, 1H), 7.55 (t, J = 7.9 Hz, 1H), 7.20 (s, 1H), 6.77 (d, J = 9.1 Hz, 2H), 3.05 (s, 6H) (in ^{1}H NMR spectrum 6% of diketone form is present). ^{13}C NMR (100.6 MHz, DMSO[D_6]) δ = 187.0, 179.5, 153.8, 133.9, 131.9, 130.8, 130.0, 126.7, 125.6, 121.2 (2C), 111.2, 92.3, 39.8. HRMS (*m*/*z*) [M+Na]$^+$ calcd for M = $C_{17}H_{16}ClNO_2$, 324.0767; found, 324.0774.

3-(3-Chlorophenyl)-5-[4-(dimethylamino)phenyl]-1*H*-pyrazole (anle254b)

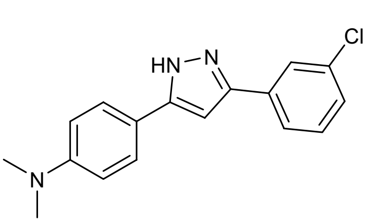

The title compound was prepared according to the GEP 2 starting from **anle254a**. White solid. Yield 85%. m.p.: 184-186 °C. ^{1}H NMR (400 MHz, DMSO[D_6] + 1-2% conc. DCl) δ = 7.87 (t, J = 1.6 Hz, 1H), 7.79 (d, J = 7.8 Hz, 1H), 7.63 (br d, J = 8.8 Hz, 2H), 7.44 (t, J = 7.8 Hz, 1H), 7.35 (d, J = 7.8 Hz, 1H), 7.06 (s, 1H), 6.80 (d, J = 8.8 Hz, 2H), 2.94 (s, 6H). ^{13}C NMR (100.6 MHz, DMSO[D_6] + 1-2% conc. DCl) δ = 150.1, 148.1, 146.5, 135.5, 135.3, 133.8, 130.8, 127.4, 126.3, 124.8, 123.8, 112.8, 98.7, 40.4. HRMS (*m*/*z*) [M+H]$^+$ calcd for M = $C_{17}H_{16}ClN_3$, 298.1111; found, 298.1111.

1-(3,4-Dimethoxyphenyl)-3-(3-fluorophenyl)propane-1,3-dione (sery157)

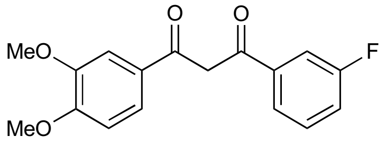

The title compound was prepared according to the GEP 1 using 1-(3,4-dimethoxyphenyl)ethanone and ethyl 3-fluorobenzoate and purified by recrystallisation from methanol to provide **sery157** as a yellow solid. Yield 92%. m.p.: 91-92 °C. ^{1}H NMR (400 MHz, DMSO[D_6]) δ = 8.03-7.95 (m, 2H), 7.90 (dd, J = 8.5, 2.0 Hz, 1H), 7.66-7.57 (m, 2H), 7.48 (td, J = 8.5, 2.4 Hz, 1H), 7.31 (s, 1H), 7.12 (d, J = 8.6 Hz, 1H), 3.87 (s, 6H) (in ^{1}H NMR spectrum 7% of diketone form is present). ^{13}C NMR (100.6 MHz, DMSO[D_6]) δ = 186.7, 181.2, 162.3 (d, J = 244.3 Hz), 153.4, 148.8, 137.0 (d, J

= 7.1 Hz), 130.9 (d, *J* = 8.1 Hz), 127.1, 123.3, 122.3, 119.4 (d, *J* = 21.3 Hz), 113.8 (d, *J* = 23.0 Hz), 111.3, 110.2, 93.0, 55.8, 55.75. HRMS (*m*/*z*) $[M+H]^+$ calcd for M = $C_{17}H_{15}FO_4$, 303.1033; found, 303.1032.

3-(3,4-Dimethoxyphenyl)-5-(3-fluorophenyl)-1*H*-pyrazole (sery161)

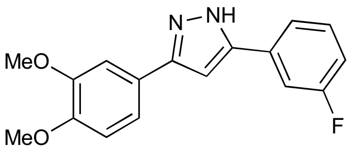

The title compound was prepared according to the GEP 2 starting from **sery157**. White solid. Yield 95%. m.p.: 172-173 °C. ^{1}H NMR (400 MHz, DMSO[D_6] + 1-2% conc. DCl) δ = 13.30 (bs, 1H), 7.69 (d, *J* = 7.8 Hz, 1H), 7.64 (d, *J* = 10.2 Hz, 1H), 7.51-7.44 (m, 1H), 7.41 (d, *J* = 1.7 Hz, 1H), 7.36 (dd, *J* = 8.3, 1.7 Hz, 1H), 7.20 (s, 1H), 7.14 (td, *J* = 8.6, 2.3 Hz, 1H), 7.03 (d, *J* = 8.3 Hz, 1H), 3.84 (s, 3H), 3.79 (s, 3H). ^{13}C NMR (100.6 MHz, DMSO[D_6] + 1-2% conc. DCl) δ = 162.7 (d, *J* = 242.7 Hz), 149.1, 148.9, 147.8, 146.6, 134.9, 130.9 (d, *J* = 8.5 Hz), 123.4, 121.2, 117.8, 114.3 (d, *J* = 21.2 Hz), 112.2, 111.7 (d, *J* = 22.7 Hz), 109.1, 99.8, 55.7, 55.67. HRMS (*m*/*z*) $[M+H]^+$ calcd for M = $C_{17}H_{15}FN_2O_2$, 299.1196; found, 299.1197.

3-(3,4-Dihydroxyphenyl)-5-(3-fluorophenyl)-1*H*-pyrazole (sery85)

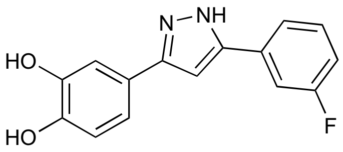

The title compound was prepared according to the GEP 3 starting from **sery161**. White solid. Yield 85%. m.p.: 231-233 °C. ^{1}H NMR (400 MHz, DMSO[D_6] + 1-2% conc. DCl) δ = 7.68 (d, *J* = 7.8 Hz, 1H), 7.66-7.60 (m, 1H), 7.47 (td, *J* = 8.2, 6.2 Hz, 1H), 7.20 (d, *J* = 2.1 Hz, 1H), 7.15 (td, *J* = 8.2, 2.3 Hz, 1H), 7.09 (dd, *J* = 8.2, 2.1 Hz, 1H), 7.04 (s, 1H), 6.81 (d, *J* = 8.2 Hz, 1H). ^{13}C NMR (100.6 MHz, DMSO[D_6] + 1-2% conc. DCl) δ = 162.7 (d, *J* = 242.9 Hz), 147.5, 146.7, 145.9, 145.6, 134.6 (d, *J* = 8.0 Hz), 130.9 (d, *J* = 7.4 Hz), 121.7, 121.4, 117.0, 116.0, 114.5 (d, *J* = 21.3 Hz), 113.1, 111.8 (d, *J* = 23.7 Hz), 99.4. HRMS (*m*/*z*) $[M+H]^+$ calcd for M = $C_{15}H_{11}FN_2O_2$, 271.0883; found, 271.0882.

3-(3,4-Dihydroxyphenyl)-5-phenyl-1*H*-pyrazole (sery106)

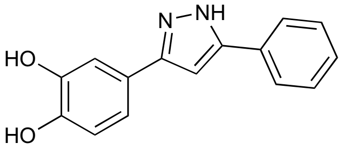

The title compound was prepared according to the GEP 3 starting from **anle126**. White solid. Yield 90%. m.p.: 224-229 °C. ^{1}H NMR (400 MHz, DMSO[D_6]) δ = 7.81 (d, *J* = 7.5 Hz, 2H), 7.43 (t, *J* = 7.5 Hz, 2H), 7.32 (t, *J* = 7.5 Hz, 1H), 7.20 (d, *J* = 1.4 Hz, 1H), 7.09 (dd, *J* = 8.1, 1.4 Hz, 1H), 6.95 (s, 1H), 6.79 (d, *J* = 8.1 Hz, 1H). ^{13}C NMR (100.6 MHz, DMSO[D_6]) δ = 147.7, 147.0, 145.7, 145.5, 131.7, 128.7, 127.8, 125.2, 122.3, 116.8, 115.8, 112.9, 98.7. HRMS (*m*/*z*) $[M+H]^+$ calcd for M = $C_{15}H_{12}N_2O_2$, 253.0977; found, 253.0975.

1-(3,4-Dimethoxyphenyl)-3-(4-methoxyphenyl)propane-1,3-dione (sery150)

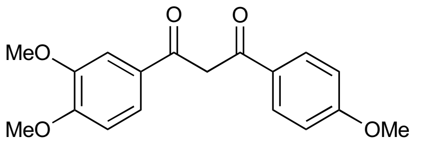

The title compound has been reported previously[17] (CAS 80370-28-1). It was prepared according to the GEP 1 using 1-(4-methoxyphenyl)ethanone and ethyl 3,4-dimethoxybenzoate and purified by recrystallisation from acetonitrile/methanol to provide **sery150** as a yellow solid. Yield 67%. m.p.: 95-96 °C. (lit[17] 97 °C). ^{1}H NMR (400 MHz, DMSO[D_6]) δ = 8.14 (d, *J* = 8.9 Hz, 2H), 7.83 (dd, *J* = 8.5, 1.9 Hz, 1H), 7.62 (d, *J* = 1.9 Hz, 1H), 7.19 (s, 1H), 7.14-7.05 (m, 3H), 3.87 (s, 3H), 3.86 (s, 6H) (in ^{1}H NMR spectrum 15% of diketone form is present).

3-(3,4-Dimethoxyphenyl)-5-(4-methoxyphenyl)-1*H*-pyrazole (sery156)

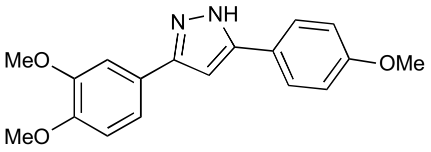

The title compound was prepared according to the GEP 2 starting from **sery150**. White solid. Yield 90%. m.p.: 82-85 °C. ^{1}H NMR (400 MHz, DMSO[D_6] + 1-2% conc. DCl) δ = 7.74 (d, *J* = 8.7 Hz, 2H), 7.41 (d, *J* = 1.7 Hz, 1H), 7.36 (dd, *J* = 8.3, 1.7 Hz, 1H), 7.09-6.94 (m, 4H), 3.84 (s, 3H), 3.79 (s, 3H), 3.78 (s, 3H). ^{13}C NMR (100.6 MHz, DMSO[D_6]) δ = 158.9, 151.1, 149.0, 148.6, 143.6, 126.4, 125.7, 122.6, 117.6, 114.2, 112.1, 109.0, 98.4, 55.6, 55.5, 55.1. HRMS (*m*/*z*) $[M+H]^+$ calcd for M = $C_{18}H_{18}N_2O_3$, 311.1396; found, 311.1397.

1-(3-Bromophenyl)-3-(3,4-dimethoxyphenyl)propane-1,3-dione (sery255a)

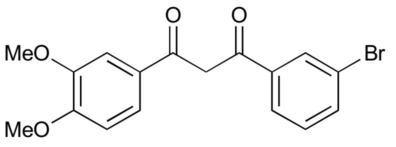

The title compound was prepared according to the GEP 1 using 1-(3,4-dimethoxyphenyl)ethanone and methyl 3-bromobenzoate and purified by recrystallisation from acetonitrile/methanol to provide **sery255a** as a yellow solid. Yield 74%. m.p.: 131-132 °C. ^{1}H NMR (400 MHz, DMSO[D$_6$]) δ = 8.31 (t, J = 1.6 Hz, 1H), 8.14 (d, J = 8.0 Hz, 1H), 7.91 (dd, J = 8.6, 2.0 Hz, 1H), 7.81 (dd, J = 8.0, 1.0 Hz, 1H), 7.64 (d, J = 2.0 Hz, 1H), 7.51 (t, J = 8.0 Hz, 1H), 7.32 (s, 1H), 7.11 (d, J = 8.6 Hz, 1H), 3.87 (s, 6H) (in ^{1}H NMR spectrum 7% of diketone form is present). ^{13}C NMR (100.6 MHz, DMSO[D$_6$]) δ = 186.8, 181.0, 153.4, 148.8, 136.8, 135.2, 130.9, 129.6, 127.1, 126.2, 122.4, 122.3, 111.2, 110.2, 93.1, 55.8 (2C). HRMS (m/z) [M+H]$^+$ calcd for M = $C_{17}H_{15}{}^{79}BrO_4$, 363.0232; found, 363.0237.

3-(3-Bromophenyl)-5-(3,4-dimethoxyphenyl)-1*H*-pyrazole (sery255b)

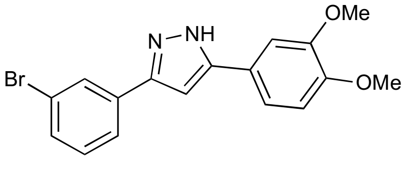

The title compound was prepared according to the GEP 2 starting from **sery255a**. White solid. Yield 98%. m.p.: 164-165 °C. ^{1}H NMR (400 MHz, DMSO[D$_6$] + 1-2% conc. DCl) δ = 8.03 (t, J = 1.7 Hz, 1H), 7.85 (dd, J = 7.9, 0.9 Hz, 1H), 7.51 (ddd, J = 7.9, 1.7, 0.9 Hz, 1H), 7.44-7.33 (m, 3H), 7.22 (s, 1H), 7.03 (d, J = 8.4 Hz, 1H), 3.84 (s, 3H), 3.79 (s, 3H). ^{13}C NMR (100.6 MHz, DMSO[D$_6$] + 1-2% conc. DCl) δ = 149.0, 148.8, 147.6, 146.4, 135.0, 131.0, 130.3, 127.5, 124.0, 123.4, 122.3, 117.7, 112.1, 109.0, 99.7, 55.7, 55.6. HRMS (m/z) [M+H]$^+$ calcd for M = $C_{17}H_{15}{}^{79}BrN_2O_2$, 359.0395; found, 359.0402.

1-(3-Bromophenyl)-3-(2-methoxyphenyl)propane-1,3-dione (sery256a)

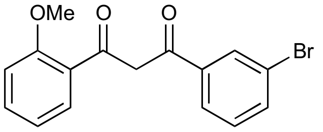

The title compound was prepared according to the GEP 1 using 1-(3-bromophenyl)ethanone and methyl 2-methoxybenzoate and purified by column chromatography (hexane:CH_2Cl_2, 2:1 v/v) to provide **sery256a** as a white solid. Yield 60%. TLC (hexane:EtOAc, 5:1 v/v): RF = 0.52. m.p.: 84 °C. ^{1}H NMR (400 MHz, $CDCl_3$) δ = 8.09 (t, J = 1.7 Hz, 1H), 7.94 (dd, J = 7.8, 1.8 Hz, 1H), 7.87 (dt, J = 7.8, 1.3 Hz, 1H), 7.65 (ddd, J = 8.0, 1.9, 1.0 Hz, 1H), 7.52-7.45 (m, 1H), 7.35 (t, J = 7.9 Hz, 1H), 7.11-7.05 (m, 2H), 7.01 (d, J = 8.4 Hz, 1H), 3.97 (s, 3H). ^{13}C NMR (100.6 MHz, $CDCl_3$) δ = 184.8, 184.1, 158.7, 138.2, 135.1, 133.5, 130.5, 130.4, 130.3, 125.9, 124.8, 123.0, 121.0, 111.9, 98.8, 56.0. HRMS (m/z) [M+H]$^+$ calcd for M = $C_{16}H_{13}{}^{79}BrO_3$, 333.0126; found, 333.0126.

3-(3-Bromophenyl)-5-(2-methoxyphenyl)-1*H*-pyrazole (sery256b)

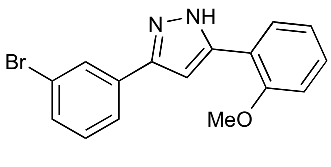

The title compound was prepared according to the GEP 2 starting from **sery256a** and purified by recrystallisation from ethanol to provide **sery256b** as a white solid. Yield 63%. m.p.: 113-114 °C. ^{1}H NMR (400 MHz, DMSO[D$_6$] + 1-2% conc. DCl) δ = 8.04 (t, J = 1.7 Hz, 1H), 7.87 (d, J = 7.8 Hz, 1H), 7.78 (d, J = 7.0 Hz, 1H), 7.50 (ddd, J = 8.1, 1.7, 0.8 Hz, 1H), 7.43-7.32 (m, 2H), 7.23 (s, 1H), 7.15 (d, J = 8.2 Hz, 1H), 7.05 (td, J = 7.4, 0.8 Hz, 1H), 3.92 (s, 3H). ^{13}C NMR (100.6 MHz, DMSO[D$_6$] + 1-2% conc. DCl) δ = 155.8, 147.6, 141.9, 135.6, 130.9, 130.0, 129.4, 127.7, 127.4, 124.0, 122.2, 120.7, 118.3, 111.9, 102.2, 55.5. HRMS (m/z) [M+H]$^+$ calcd for M = $C_{16}H_{13}{}^{79}BrN_2O$, 329.0290; found, 329.0294.

1-(3-Bromophenyl)-3-(5-chloro-2-methoxyphenyl)propane-1,3-dione (sery257a)

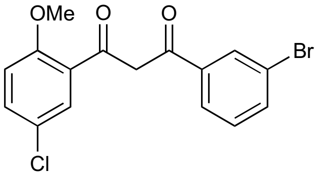

The title compound was prepared according to the GEP 1 using 1-(3-bromophenyl)ethanone and methyl 5-chloro-2-methoxybenzoate and purified by recrystallisation from methanol (twice) to provide **sery257a** as a yellow solid. Yield 75%. m.p.: 115-116 °C. ^{1}H NMR (400 MHz, $CDCl_3$) δ = 8.08 (t, J = 1.7 Hz, 1H), 7.90 (d, J = 2.7 Hz, 1H), 7.86 (d, J = 7.8 Hz, 1H), 7.66 (m, 1H), 7.42 (dd, J = 8.9, 2.7 Hz, 1H), 7.35 (t, J = 7.9 Hz, 1H), 7.07 (s, 1H), 6.94 (d, J = 8.9 Hz, 1H), 3.96 (s, 3H). ^{13}C NMR (100.6 MHz, $CDCl_3$) δ = 184.5, 183.0, 157.2, 137.9, 135.3, 132.9, 130.5, 130.3, 130.1, 126.4, 126.0, 125.9, 123.1, 113.3, 98.8, 56.4. HRMS (m/z) [M+H]$^+$ calcd for M = $C_{16}H_{12}{}^{79}BrClO_3$, 366.9737; found, 366.9738.

3-(3-Bromophenyl)-5-(5-chloro-2-methoxyphenyl)-1*H*-pyrazole (sery257b)

The title compound was prepared according to the GEP 2 starting from **sery257a** and purified by recrystallisation from ethanol to provide **sery257b** as a white solid. Yield 85%. m.p.: 163-164 °C. ^{1}H NMR (400

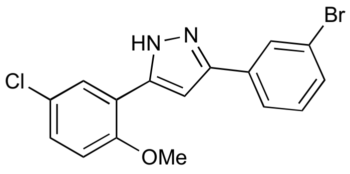

MHz, DMSO[D_6] + 1-2% conc. DCl) δ = 8.05 (t, J = 1.5 Hz, 1H), 7.91-7.81 (m, 2H), 7.51 (d, J = 8.3 Hz, 1H), 7.44-7.36 (m, 2H), 7.33 (s, 1H), 7.17 (d, J = 8.9 Hz, 1H), 3.93 (s, 3H). ^{13}C NMR (100.6 MHz, DMSO[D_6] + 1-2% conc. DCl) δ = 154.6, 147.2, 141.4, 135.1, 130.9, 130.2, 128.6, 127.5, 126.8, 124.5, 124.0, 122.2, 120.3, 113.9, 103.1, 55.9. HRMS (m/z) [M+H]$^+$ calcd for M = $C_{16}H_{12}{}^{79}BrClN_2O$, 362.9900; found, 362.9903.

3-(3-Bromophenyl)-5-(5-chloro-2-hydroxyphenyl)-1*H*-pyrazole (sery260b)

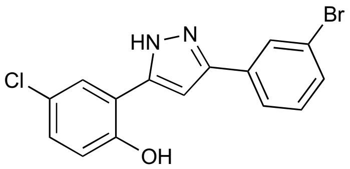

The title compound was prepared according to the GEP 4 starting from **sery257b** and purified by recrystallisation from petroleum benzine (40-60 °C)/chloroform and second recrystallisation from hexane/EtOAc to provide **sery260b** as a white solid. Yield 51%. m.p.: 217-220 (decomp.) °C. ^{1}H NMR (400 MHz, DMSO[D_6] + 1-2% conc. DCl) δ = 10.74 (bs, 1H), 8.04 (s, 1H), 7.84 (d, J = 7.8 Hz, 1H), 7.80 (d, J = 2.6 Hz, 1H), 7.54 (d, J = 7.8 Hz, 1H), 7.47-7.37 (m, 2H), 7.22 (dd, J = 8.7, 2.6 Hz, 1H), 6.99 (d, J = 8.7 Hz, 1H). ^{13}C NMR (100.6 MHz, DMSO[D_6] + 1-2% conc. DCl) δ = 153.6, 145.2, 144.5, 133.7, 131.2, 130.8, 128.6, 127.8, 126.5, 124.3, 123.1, 122.4, 118.5, 118.2, 102.0. HRMS (m/z) [M+H]$^+$ calcd for M = $C_{15}H_{10}{}^{79}BrClN_2O$, 348.9743; found, 348.9748.

1-(3-Fluorophenyl)-3-(2-methoxyphenyl)propane-1,3-dione (sery258)

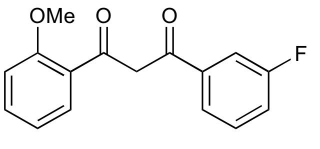

The title compound was prepared according to the GEP 1 using 1-(3-fluorophenyl)ethanone and methyl 2-methoxybenzoate and purified by recrystallisation (twice) from methanol to provide **sery258** as a yellow solid. Yield 73%. m.p.: 95-96 °C. ^{1}H NMR (400 MHz, $CDCl_3$) δ = 7.95 (dd, J = 7.8, 1.6 Hz, 1H), 7.74 (d, J = 7.8 Hz, 1H), 7.65 (d, J = 9.7 Hz, 1H), 7.53-7.40 (m, 2H), 7.23 (td, J = 8.4, 2.3 Hz, 1H), 7.12 (s, 1H), 7.07 (t, J = 7.4 Hz, 1H), 7.01 (d, J = 8.4 Hz, 1H), 3.97 (s, 3H). ^{13}C NMR (100.6 MHz, $CDCl_3$) δ = 184.7, 184.2, 163.0 (d, J = 246.5 Hz), 158.7, 138.5 (d, J = 7.1 Hz), 133.5, 130.5, 130.3, 124.7, 123.0, 121.0, 119.1 (d, J = 21.4 Hz), 114.2 (d, J = 18.2 Hz), 111.8, 98.8, 96.0. HRMS (m/z) [M+H]$^+$ calcd for M = $C_{16}H_{13}FO_3$, 273.0927; found, 273.0926.

3-(3-Fluorophenyl)-5-(2-methoxyphenyl)-1*H*-pyrazole (sery261a)

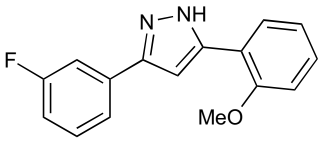

The title compound has been reported previously[18] (CAS 939051-95-3). It was prepared according to the GEP 2 starting from **sery258** and purified by column chromatography (hexane:EtOAc, 4:1 v/v) to provide **sery261a** as a white solid. Yield 90%. TLC (hexane:EtOAc, 3:1 v/v): RF = 0.51. m.p.: 109 °C. ^{1}H NMR (400 MHz, DMSO[D_6] + 1-2% conc. DCl) δ = 7.78 (d, J = 7.4 Hz, 1H), 7.71 (d, J = 7.7 Hz, 1H), 7.65 (d, J = 10.4 Hz, 1H), 7.47 (q, J = 7.7 Hz, 1H), 7.35 (t, J = 7.4 Hz, 1H), 7.21 (s, 1H), 7.18-7.09 (m. 2H), 7.04 (t, J = 7.4 Hz, 1H), 3.91 (s, 3H). HRMS (m/z) [M+H]$^+$ calcd for M = $C_{16}H_{13}FN_2O$, 269.1090; found, 269.1090.

3-(3-Fluorophenyl)-5-(2-hydroxyphenyl)-1*H*-pyrazole (sery263a)

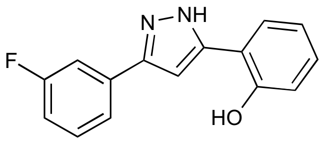

The title compound has been reported previously[19] (CAS 312314-31-1). It was prepared according to the GEP 4 starting from **sery261a** and purified by column chromatography (hexane:EtOAc, 5:1 v/v) to provide **sery263a** as a white solid. Yield 59%. TLC (hexane:EtOAc, 6:1 v/v): RF = 0.28. m.p.: 146 °C. ^{1}H NMR (400 MHz, DMSO[D_6] + 1-2% conc. DCl) δ = 10.56 (s, 1H), 7.78-7.61 (m, 3H), 7.50 (m, 1H), 7.30 (s, 1H), 7.23-7.13 (m, 2H), 6.97 (dd, J = 8.2, 0.9 Hz, 1H), 6.91 (td, J = 7.6, 1.0 Hz, 1H). HRMS (m/z) [M+H]$^+$ calcd for M = $C_{15}H_{11}FN_2O$, 255.0934; found, 255.0932.

1-(4-Chlorophenyl)-3-(4-methoxyphenyl)propane-1,3-dione (sery269a)

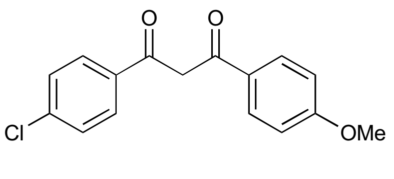

The title compound has been reported previously[20] (CAS 37975-19-2). It was prepared according to the GEP 1 using 1-(4-methoxyphenyl)ethanone and ethyl 4-chlorobenzoate and purified by recrystallisation from methanol to provide **sery269a** as a white solid. Yield 84%. m.p.: 116-117 °C. ^{1}H NMR (400 MHz, DMSO[D_6]) δ = 8.15 (d, J = 8.3 Hz, 4H), 7.60 (d, J = 8.6 Hz, 2H), 7.28 (s, 1H), 7.08 (d, J = 8.9 Hz, 2H), 3.86 (s,

3H) (in ^{1}H NMR spectrum 7.5% of diketone form is present).

3-(4-Chlorophenyl)-5-(4-methoxyphenyl)-1*H*-pyrazole (sery269b)

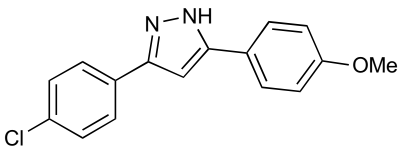

The title compound has been reported previously[21] (CAS 1204141-84-3). It was prepared according to the GEP 2 starting from **sery269a** and purified by recrystallisation from ethanol to provide **sery269b** as a white solid. Yield 83%. m.p.: 201-202 °C. (lit[21] 221 °C). ^{1}H NMR (400 MHz, DMSO[D$_6$] + 1-2% conc. DCl) δ= 7.84 (d, J = 8.6 Hz, 2H), 7.74 (d, J = 8.8 Hz, 2H), 7.49 (d, J = 8.6 Hz, 2H), 7.08 (s, 1H), 7.02 (d, J = 8.8 Hz, 2H), 3.78 (s, 3H).

3-(4-Chlorophenyl)-5-(4-hydroxyphenyl)-1*H*-pyrazole hydrobromide (sery320a)

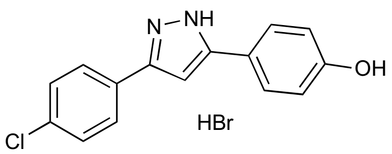

This compound was prepared according to the GEP 3 starting from **sery269b**. White solid. Yield 92%. m.p.: 288-293 °C. ^{1}H NMR (400 MHz, DMSO[D$_6$]) δ= 13.00-11.00 (bs, 2.6H), 7.87 (d, J = 8.5 Hz, 2H), 7.67 (d, J = 8.6 Hz, 2H), 7.51 (d, J = 8.5 Hz, 2H), 7.16 (s, 1H), 6.87 (d, J = 8.6 Hz, 2H). ^{13}C NMR (100.6 MHz, DMSO[D$_6$]) δ= 158.0, 147.3, 146.5, 132.7, 130.2, 129.0, 128.9, 127.2, 127.0, 120.6, 115.8, 115.7, 99.3. HRMS (m/z) [M+H]$^+$ calcd for M = $C_{15}H_{11}ClN_2O$, 271.0638; found, 271.0643.

1-(4-Chlorophenyl)-3-(3-methoxyphenyl)propane-1,3-dione (sery287a)

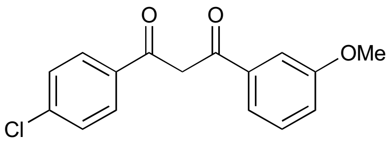

The title compound was prepared according to the GEP 1 using 1-(4-chlorophenyl)ethanone and ethyl 3-methoxybenzoate and purified by recrystallisation (twice) from methanol to provide **sery287a** as a yellow solid. Yield 34%. m.p.: 77-79 °C. ^{1}H NMR (400 MHz, DMSO[D$_6$]) δ = 8.19 (d, J = 8.5 Hz, 2H), 7.76 (d, J = 7.6 Hz, 1H), 7.70-7.57 (m, 3H), 7.48 (t, J = 8.0 Hz, 1H), 7.34 (s, 1H), 7.22 (dd, J = 8.0, 1.9 Hz, 1H), 3.86 (s, 3H). ^{13}C NMR (100.6 MHz, DMSO[D$_6$]) δ= 185.3, 184.0, 159.6, 137.9, 135.9, 133.4, 130.0, 129.3, 128.9, 119.9, 119.0, 112.3, 93.6, 55.4. HRMS (m/z) [M+H]$^+$ calcd for M = $C_{16}H_{13}ClO_3$, 289.0631; found, 289.0630.

3-(4-Chlorophenyl)-5-(3-methoxyphenyl)-1*H*-pyrazole (sery289)

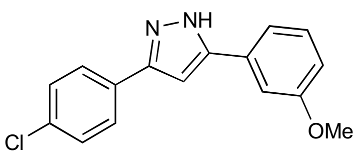

The title compound was prepared according to the GEP 2 starting from **sery287a** and purified by column chromatography (hexane:EtOAc, 3.5:1 v/v) to provide **sery289** as a white solid. Yield 65%. TLC (hexane:EtOAc, 3:1 v/v): RF = 0.40. m.p.: 176-177 °C. ^{1}H NMR (400 MHz, DMSO[D$_6$] + 1-2% conc. DCl) δ= 7.86 (d, J = 8.6 Hz, 2H), 7.51 (d, J = 8.6 Hz, 2H), 7.43-7.33 (m, 3H), 7.23 (s, 1H), 6.95-6.89 (m, 1H), 3.82 (s, 3H). ^{13}C NMR (100.6 MHz, DMSO[D$_6$] + 1-2% conc. DCl) δ = 159.7, 148.0-147.0 (2C), 132.2, 131.9, 130.4, 130.1, 128.9, 126.9, 117.5, 113.8, 110.5, 100.2, 55.4. HRMS (m/z) [M+H]$^+$ calcd for M = $C_{16}H_{13}ClN_2O$, 285.0795; found, 285.0797.

3-(4-Chlorophenyl)-5-(3-hydroxyphenyl)-1*H*-pyrazole hydrobromide (sery320b)

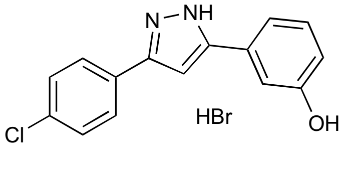

This compound was prepared according to the GEP 3 starting from **sery289**. White solid. Yield 82%. m.p.: 246-251 °C (dec). ^{1}H NMR (400 MHz, DMSO[D$_6$]) δ= 9.0-8.0 (bs, 4H), 7.87 (d, J = 8.6 Hz, 2H), 7.50 (d, J = 8.6 Hz, 2H), 7.30-7.18 (m, 3H), 7.15 (s, 1H), 6.78 (m, 1H). ^{13}C NMR (100.6 MHz, DMSO[D$_6$]) δ= 157.8, 147.0, 146.7, 132.3, 131.8, 130.7, 129.9, 128.8, 126.9, 116.2, 115.2, 112.1, 100.0. HRMS (m/z) [M+H]$^+$ calcd for M = $C_{15}H_{11}ClN_2O$, 271.0638; found, 271.0642.

1-(5-Chloro-2-methoxyphenyl)-3-(3-fluorophenyl)propane-1,3-dione (sery259)

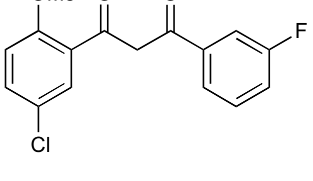

The title compound was prepared according to the GEP 1 using 1-(3-fluorophenyl)ethanone and methyl 5-chloro-2-methoxybenzoate and purified by recrystallisation (twice) from methanol to provide **sery259** as a yellow solid. Yield 90%. m.p.: 98-99 °C. ^{1}H NMR (400 MHz, CDCl$_3$) δ= 7.92 (d, J = 2.8 Hz, 1H), 7.73 (d, J = 7.9 Hz, 1H), 7.64 (ddd, J = 9.7, 2.3, 1.7 Hz, 1H), 7.49-7.40 (m,

2H), 7.24 (tdd, J = 8.3, 2.6, 0.8 Hz, 1H), 7.09 (s, 1H), 6.95 (d, J = 8.9 Hz, 1H), 3.96 (s, 3H). ^{13}C NMR (100.6 MHz, $CDCl_3$) δ = 184.7, 183.0, 163.0 (d, J = 247.2 Hz), 157.2, 138.2 (d, J = 7.0 Hz), 132.9, 130.4 (d, J = 7.9 Hz), 130.1, 126.4, 126.0, 123.0, 119.4 (d, J = 21.4 Hz), 114.3 (d, J = 23.0 Hz), 113.3, 98.8, 56.4. HRMS (*m*/*z*) $[M+H]^+$ calcd for M = $C_{16}H_{12}ClFO_3$, 307.0537; found, 307.0537.

3-(5-Chloro-2-methoxyphenyl)-5-(3-fluorophenyl)-1*H*-pyrazole (sery261b)

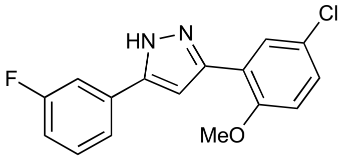

The title compound was prepared according to the GEP 2 starting from **sery259** and purified by column chromatography (hexane:EtOAc, 4:1 v/v) to provide **sery261b** as a white solid. Yield 98%. TLC (hexane:EtOAc, 3:1 v/v): RF = 0.45. m.p.: 133 °C. ^{1}H NMR (400 MHz, DMSO[D_6] + 1-2% conc. DCl) δ = 7.85 (s, 1H), 7.76-7.61 (m, 2H), 7.48 (q, J = 6.8 Hz, 1H), 7.38 (d, J = 7.7 Hz, 1H), 7.31 (s, 1H), 7.21-7.10 (m, 2H), 3.92 (s, 3H). ^{13}C NMR (100.6 MHz, DMSO[D_6] + 1-2% conc. DCl) δ = 162.7 (d, J = 242.6 Hz), 154.7, 147.2, 141.2, 134.7, 130.9 (d, J = 8.4 Hz), 128.7, 126.9, 124.6, 121.2, 120.6, 114.4 (d, J = 21.0 Hz), 113.9, 111.7 (d, J = 22.6 Hz), 103.3, 56.0. HRMS (*m*/*z*) $[M+H]^+$ calcd for M = $C_{16}H_{12}ClFN_2O$, 303.0700; found, 303.0704.

3-(5-Chloro-2-hydroxyphenyl)-5-(3-fluorophenyl)-1*H*-pyrazole (sery263b)

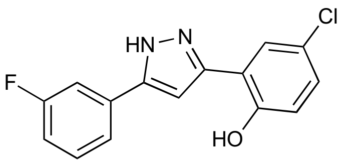

The title compound was prepared according to the GEP 4 starting from **sery261b** and purified by column chromatography (hexane:EtOAc, 6:1 v/v) to provide **sery263b** as a white solid. Yield 60%. TLC (hexane:EtOAc, 6:1 v/v): RF = 0.38. m.p.: 202 °C. ^{1}H NMR (400 MHz, DMSO[D_6] + 1-2% conc. DCl) δ = 10.74 (bs, 1H), 7.80 (d, J = 2.6 Hz, 1H), 7.73-7.61 (m, 2H), 7.51 (m, 1H), 7.40 (s, 1H), 7.26-7.14 (m, 2H), 6.98 (d, J = 8.7 Hz, 1H). ^{13}C NMR (100.6 MHz, DMSO[D_6] + 1-2% conc. DCl) δ = 162.6 (d, J = 243.0 Hz), 153.6, 146.0-144.8 (2C), 133.2, 131.1 (d, J = 8.5 Hz), 128.6, 126.4, 123.0, 121.4, 118.5, 118.2, 114.8 (d, J = 19.9 Hz), 111.9 (d, J = 23.0 Hz), 102.0. HRMS (*m*/*z*) $[M+H]^+$ calcd for M = $C_{15}H_{10}ClFN_2O$, 289.0544 found, 289.0546.

1-(3-Chlorophenyl)-3-(3-methoxyphenyl)propane-1,3-dione (sery287b)

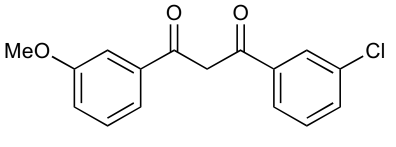

The title compound was prepared according to the GEP 1 using 1-(3-chlorophenyl)ethanone and ethyl 3-methoxybenzoate and purified by recrystallisation from methanol followed by column chromatography (hexane:$CHCl_3$, 3:1 v/v) to provide **sery287b** as a white solid. Yield 30%. TLC (hexane:EtOAc, 5:1 v/v): RF = 0.45. m.p.: 56-57 °C. ^{1}H NMR (400 MHz, $CDCl_3$) δ = 7.95 (t, J = 1.9 Hz, 1H), 7.86 (dt, J = 7.8, 1.3 Hz, 1H), 7.56 (d, J = 7.8 Hz, 1H), 7.54-7.50 (m, 2H), 7.46-7.38 (m, 2H), 7.11 (ddd, J = 8.2, 2.6, 0.7 Hz, 1H), 6.79 (s, 1H), 3.89 (s, 3H). ^{13}C NMR (100.6 MHz, $CDCl_3$) δ = 186.4, 183.9, 160.1, 137.4, 136.9, 135.1, 132.4, 130.1, 129.9, 127.4, 125.4, 119.8, 119.0, 112.2, 93.7, 55.6. HRMS (*m*/*z*) $[M+H]^+$ calcd for M = $C_{16}H_{13}ClO_3$, 289.0631; found, 289.0633.

3-(3-Chlorophenyl)-5-(3-methoxyphenyl)-1*H*-pyrazole (sery301)

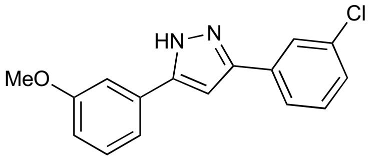

The title compound was prepared according to the GEP 2 starting from **sery287b** and purified by recrystallisation from methanol to provide **sery301** as a white solid. Yield 76%. m.p.: 197-198 °C. ^{1}H NMR (400 MHz, DMSO[D_6] + 1-2% conc. DCl) δ = 7.90 (s, 1H), 7.81 (d, J = 7.6 Hz, 1H), 7.53-7.33 (m, 5H), 7.31 (s, 1H), 6.92 (d, J = 7.5 Hz, 1H), 3.82 (s, 3H). ^{13}C NMR (100.6 MHz, DMSO[D_6] + 1-2% conc. DCl) δ = 159.7, 147.2, 146.3, 134.2, 133.7, 132.0, 130.8, 130.1, 127.5, 124.7, 123.7, 117.5, 113.8, 110.5, 100.5, 55.2. HRMS (*m*/*z*) $[M+H]^+$ calcd for M = $C_{16}H_{13}ClN_2O$, 285.0795; found, 285.0797.

3-(3-Chlorophenyl)-5-(3-hydroxyphenyl)-1*H*-pyrazole hydrobromide (sery320c)

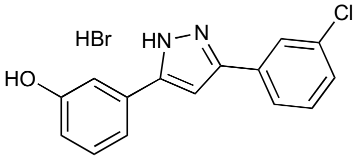

This compound was prepared according to the GEP 3 starting from **sery301**. White solid. Yield 67%. m.p.: 244-250 °C. ^{1}H NMR (400 MHz, DMSO[D_6]) δ = 11.06 bs, 1H), 7.91 (t, J = 1.7 Hz, 1H), 7.81 (d, J = 7.8 Hz, 1H), 7.47 (t, J = 7.8 Hz, 1H), 7.38 (m, 1H), 7.26-7.20 (m, 4H), 6.78 (m, 1H). ^{13}C NMR (100.6 MHz, DMSO[D_6]) δ = 157.8, 146.8, 146.7, 134.0, 133.7, 131.8, 130.7, 129.9, 127.5, 124.8, 123.8, 116.2, 115.2, 112.1, 100.3. HRMS (*m*/*z*) $[M+H]^+$ calcd for M = $C_{15}H_{11}ClN_2O$, 271.0638; found, 271.0640.

1-(3-Bromophenyl)-3-(naphthalen-2-yl)propane-1,3-dione (sery292a)

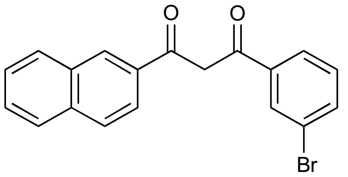

The title compound was prepared according to the GEP 1 using 1-(naphthalen-2-yl)ethanone and ethyl 3-bromobenzoate and purified by recrystallisation from methanol to provide **sery292a** as a white solid. Yield 73%. m.p.: 121-122 °C. ^{1}H NMR (400 MHz, DMSO[D$_6$]) δ = 8.88 (s, 1H), 8.40 (t, J = 1.6 Hz, 1H), 8.24 (dd, J = 8.6, 1.6 Hz, 1H), 8.20 (d, J = 7.9 Hz, 1H), 8.13 (d, J = 7.9 Hz, 1H), 8.07 (d, J = 8.6 Hz, 1H), 8.02 (d, J = 7.7 Hz, 1H), 7.88-7.82 (m, 1H), 7.72-7.61 (m, 2H), 7.58-7.51 (m, 2H), (in ^{1}H NMR spectrum 5.5% of diketone form is present). ^{13}C NMR (100.6 MHz, DMSO[D$_6$]) δ = 185.7, 183.3, 136.9, 135.6, 135.1, 132.3, 131.7, 131.0, 129.8, 129.4, 129.0, 128.6, 128.4, 127.7, 127.0, 126.4, 123.4, 122.3, 94.0. HRMS (m/z) [M+H]$^+$ calcd for M = $C_{19}H_{13}{}^{79}BrO_2$, 353.0177; found, 353.0178.

3-(3-Bromophenyl)-5-(naphthalen-2-yl)-1*H*-pyrazole (sery292b)

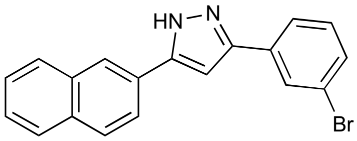

The title compound was prepared according to the GEP 2 starting from **sery292a** and purified by recrystallisation from ethanol to provide **sery292b** as a white solid. Yield 88%. m.p.: 189-190 °C. ^{1}H NMR (400 MHz, DMSO[D$_6$] + 1-2% conc. DCl) δ = 8.37 (s, 1H), 8.09 (t, J = 1.6 Hz, 1H), 8.00 (s, 2H), 7.97-7.91 (m, 2H), 7.89 (d, J = 7.8 Hz, 1H), 7.59-7.48 (m, 3H), 7.46-7.40 (m, 2H). ^{13}C NMR (100.6 MHz, DMSO[D$_6$] + 1-2% conc. DCl) δ = 147.5, 146.9, 130.5, 134.1, 133.2, 132.6, 131.1, 130.5, 128.6, 128.0, 127.8, 127.6, 126.8, 126.3 (2C), 124.1, 123.6, 122.4, 100.8. HRMS (m/z) [M+H]$^+$ calcd for M = $C_{19}H_{13}{}^{79}BrN_2$, 349.0340; found, 349.0346.

1-(1,3-Benzodioxol-5-yl)-3-(3,5-difluorophenyl)propane-1,3-dione (sery294a)

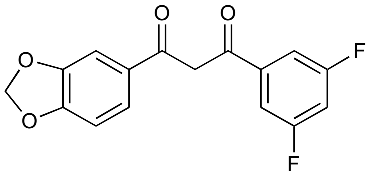

The title compound was prepared according to the GEP 1 using 1-(1,3-benzodioxol-5-yl)ethanone and methyl 3,5-difluorobenzoate and purified by recrystallisation from methanol to provide **sery294a** as a yellow solid. Yield 85%. m.p.: 150-151 °C. ^{1}H NMR (400 MHz, DMSO[D$_6$]) δ = 7.91 (m, 3H), 7.76 (d, J = 1.7 Hz, 1H), 7.53 (tt, J = 9.1, 2.3 Hz, 1H), 7.32 (s, 1H), 7.10 (d, J = 8.3 Hz, 1H), 6.17 (s, 2H), (in ^{1}H NMR spectrum 4.5% of diketone form is present). ^{13}C NMR (100.6 MHz, DMSO[D$_6$]) δ = 186.4, 179.9, 162.6 (dd, J = 246.8, 12.7 Hz), 151.9, 148.1, 138.1, 128.6, 124.1, 110.4 (d, J = 23.0 Hz), 108.4, 107.9 (t, J = 26.1 Hz), 107.3, 102.2, 93.4. HRMS (m/z) [M+H]$^+$ calcd for M = $C_{16}H_{10}F_2O_4$, 305.0625; found, 305.0626.

3-(1,3-Benzodioxol-5-yl)-5-(3,5-difluorophenyl)-1*H*-pyrazole (sery294b)

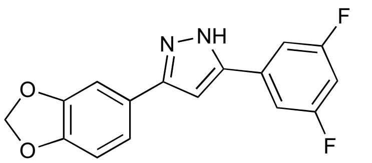

The title compound was prepared according to the GEP 2 starting from **sery294a** and purified by recrystallisation from ethanol to provide **sery294b** as a white solid. Yield 81%. m.p.: 222-223 °C. ^{1}H NMR (400 MHz, DMSO[D$_6$] + 1-2% conc. DCl) δ = 7.56-7.48 (m, 2H), 7.35 (d, J = 1.5 Hz, 1H), 7.31 (dd, J = 8.1, 1.5 Hz, 1H), 7.23 (s, 1H), 7.16 (tt, J = 9.3, 2.2 Hz, 1H), 7.01 (d, J = 8.1 Hz, 1H), 6.07 (s, 2H). ^{13}C NMR (100.6 MHz, DMSO[D$_6$] + 1-2% conc. DCl) δ = 162.8 (dd, J = 245.1, 13.4 Hz), 148.0, 147.3, 147.0, 146.1, 136.1, 124.5, 119.1, 108.9, 108.1 (d, J = 26.1 Hz), 105.7, 102.9 (t, J = 26.0 Hz), 101.4, 100.4. HRMS (m/z) [M+H]$^+$ calcd for M = $C_{16}H_{10}F_2N_2O_2$, 301.0789; found, 301.0789.

1-(1,3-Benzodioxol-5-yl)-3-(3,5-dichlorophenyl)propane-1,3-dione (sery297a)

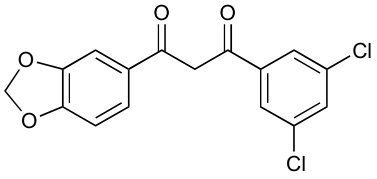

The title compound was prepared according to the GEP 1 using 1-(1,3-benzodioxol-5-yl)ethanone and methyl 3,5-dichlorobenzoate and purified by recrystallisation from methanol to provide **sery297a** as a yellow solid. Yield 98%. m.p.: 160-161 °C. ^{1}H NMR (400 MHz, $CDCl_3$) δ = 7.80 (d, J = 1.9 Hz, 2H), 7.60 (dd, J = 8.2, 1.8 Hz, 1H), 7.50 (t, J = 1.9 Hz, 1H), 7.46 (d, J = 1.8 Hz, 1H), 6.90 (d, J = 8.2 Hz, 1H), 6.65 (s, 1H), 6.08 (s, 2H). ^{13}C NMR (100.6 MHz, $CDCl_3$) δ = 186.7, 180.7, 152.1, 148.5, 138.5, 135.7, 131.9, 129.7, 125.5, 123.5, 108.4, 107.4, 102.2, 93.0. HRMS (m/z) [M+H]$^+$ calcd for M = $C_{16}H_{10}Cl_2O_4$, 337.0034; found, 337.0033.

3-(1,3-Benzodioxol-5-yl)-5-(3,5-dichlorophenyl)-1*H*-pyrazole (sery297b)

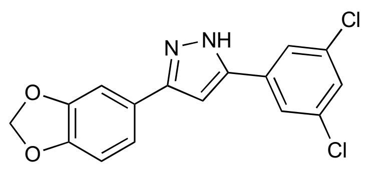

The title compound was prepared according to the GEP 2 starting from **sery297a** and purified by recrystallisation from methanol to provide **sery297b** as a white solid. Yield 81%. m.p.: 257-259 °C. ^{1}H NMR (400 MHz, DMSO[D$_6$] + 1-2% conc. DCl) δ = 7.86 (s, 2H), 7.52 (s, 1H), 7.41-7.24 (m, 3H), 7.01 (d, J = 8.0 Hz, 1H), 6.06 (s, 2H). ^{13}C NMR (100.6 MHz, DMSO[D$_6$]) δ = 148.6, 147.9, 147.3, 143.7, 137.2, 134.5, 126.6, 123.4, 123.1, 119.0, 108.8, 105.6, 101.3, 100.1. HRMS (*m*/*z*) [M+H]$^+$ calcd for M = $C_{16}H_{10}Cl_2N_2O_2$, 333.0198; found, 333.0201.

1-(3-Bromophenyl)-3-(2,3-dihydrobenzofuran-5-yl)propane-1,3-dione (sery312a)

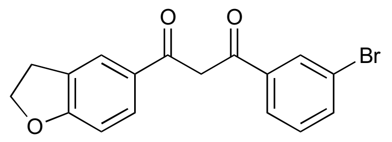

The title compound was prepared according to the GEP 1 using 1-(2,3-dihydrobenzofuran-5-yl)ethanone and ethyl 3-bromobenzoate and purified by recrystallisation from methanol to provide **sery312a** as a yellow solid. Yield 88%. m.p.: 117-118 °C. ^{1}H NMR (400 MHz, $CDCl_3$) δ= 8.08 (t, J = 1.8 Hz, 1H), 7.92-7.86 (m, 2H), 7.84 (dd, J = 8.5, 1.9 Hz, 1H), 7.65 (ddd, J = 8.0, 1.8, 1.0 Hz, 1H), 7.35 (t, J = 8.0 Hz, 1H), 6.86 (d, J = 8.5 Hz, 1H), 6.72 (s, 1H), 4.69 (t, J = 8.8 Hz, 2H), 3.29 (t, J = 8.8 Hz, 2H). ^{13}C NMR (100.6 MHz, $CDCl_3$) δ= 187.0, 181.9, 164.6, 137.8, 135.0, 130.3, 130.1, 129.2, 128.3, 128.1, 125.6, 124.7, 123.0, 109.6, 92.7, 72.4, 29.3. HRMS (*m*/*z*) [M+H]$^+$ calcd for M = $C_{17}H_{13}{}^{79}BrO_3$, 345.0126; found, 345.0128.

3-(3-Bromophenyl)-5-(2,3-dihydrobenzofuran-5-yl)-1*H*-pyrazole (sery312b)

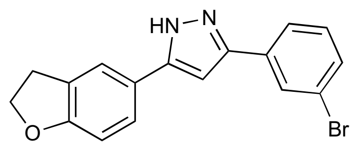

The title compound was prepared according to the GEP 2 starting from **sery312a** and purified by recrystallisation from ethanol/water to provide **sery312b** as a white solid. Yield 82%. m.p.: 169-170 °C. ^{1}H NMR (400 MHz, DMSO[D$_6$] + 1-2% conc. DCl) δ = 8.02 (s, 1H), 7.83 (d, J = 7.5 Hz, 1H), 7.68 (s, 1H), 7.55 (d, J = 8.1 Hz, 1H), 7.50 (d, J = 7.5 Hz, 1H), 7.39 (t, J = 7.8 Hz, 1H), 7.12 (s, 1H), 6.84 (d, J = 8.2 Hz, 1H), 4.56 (t, J = 8.5 Hz, 2H), 3.22 (t, J = 8.5 Hz, 2H). ^{13}C NMR (100.6 MHz, DMSO[D$_6$] + 1-2% conc. DCl) δ= 159.8, 147.0, 146.2, 134.8, 131.0, 130.3, 128.2, 127.6, 125.3, 124.1, 123.2, 122.3, 122.2, 109.3, 99.3, 71.3, 29.0. HRMS (*m*/*z*) [M+H]$^+$ calcd for M = $C_{17}H_{13}{}^{79}BrN_2O$, 341.0290; found, 341.0294.

1-(3-Bromophenyl)-3-(2,3-dihydro-1,4-benzodioxin-6-yl)propane-1,3-dione (sery315a)

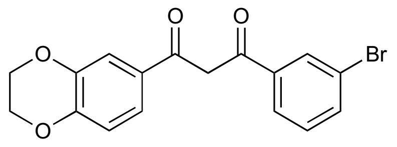

The title compound was prepared according to the GEP 1 using 1-(2,3-dihydro-1,4-benzodioxin-6-yl)ethanone and ethyl 3-bromobenzoate and purified by recrystallisation from methanol to provide **sery315a** as a yellow solid. Yield 89%. m.p.: 108-110 °C. ^{1}H NMR (400 MHz, DMSO[D$_6$]) δ= 8.35 (s, 1H), 8.13 (d, J = 8.0 Hz, 1H), 7.80 (d, J = 8.0 Hz, 1H), 7.77-7.70 (m, 2H), 7.50 (t, J = 8.0 Hz, 1H), 7.30 (s, 1H), 7.01 (d, J = 8.3 Hz, 1H), 4.40-4.25 (m, 4H). ^{13}C NMR (100.6 MHz, DMSO[D$_6$]) δ= 186.1, 181.5, 148.1, 143.6, 136.8, 135.2, 130.9, 129.7, 127.7, 126.2, 122.3, 121.7, 117.3, 116.7, 93.1, 64.6, 63.9. HRMS (*m*/*z*) [M+H]$^+$ calcd for M = $C_{17}H_{13}{}^{79}BrO_4$, 361.0075; found, 361.0077.

3-(3-Bromophenyl)-5-(2,3-dihydro-1,4-benzodioxin-6-yl)-1*H*-pyrazole (sery315b)

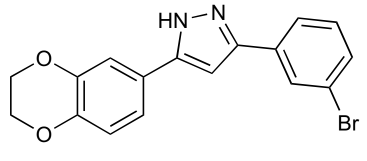

The title compound was prepared according to the GEP 2 starting from **sery315a** and purified by recrystallisation from methanol to provide **sery315b** as a white solid. Yield 69%. m.p.: 167-169 °C. ^{1}H NMR (400 MHz, DMSO[D$_6$] + 1-2% conc. DCl) δ= 8.02 (s, 1H), 7.83 (d, J = 7.8 Hz, 1H), 7.50 (d, J = 8.1 Hz, 1H), 7.39 (t, J = 7.8 Hz, 1H), 7.33 (d, J = 1.8 Hz, 1H), 7.28 (dd, J = 8.4, 1.8 Hz, 1H), 7.17 (s, 1H), 6.93 (d, J = 8.4 Hz, 1H), 4.27 (s, 4H). ^{13}C NMR (100.6 MHz, DMSO[D$_6$] + 1-2% conc. DCl) δ= 147.8, 146.3, 143.7, 143.4, 134.7, 131.0, 130.3, 127.6, 124.1 (2C), 122.3, 118.4, 117.6, 113.9, 99.7, 64.3, 64.2. HRMS (*m*/*z*) [M+H]$^+$ calcd for M = $C_{17}H_{13}{}^{79}BrN_2O_2$, 357.0239; found, 357.0242.

1-(1,3-Benzodioxol-5-yl)-3-(2,5-difluorophenyl)propane-1,3-dione (sery316a)

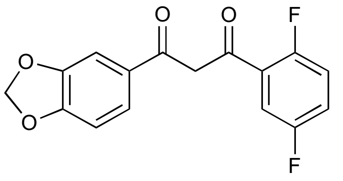

The title compound was prepared according to the GEP 1 using 1-(1,3-benzodioxol-5-yl)ethanone and methyl 2,5-difluorobenzoate and purified by recrystallisation from methanol to provide **sery316a** as a yellow solid. Yield 89%. m.p.: 131-133 °C. ^{1}H NMR (400 MHz, DMSO[D$_6$]) δ = 7.79-7.68 (m, 2H), 7.58 (d, J = 1.5 Hz, 1H), 7.54-7.40 (m, 2H), 7.08 (d, J = 8.2 Hz, 1H), 6.98 (s, 1H), 6.17 (s, 2H). ^{13}C NMR (100.6 MHz, DMSO[D$_6$]) δ = 186.2, 178.7, 158.0 (d, J = 241.6 Hz), 156.4 (d, J = 249.6 Hz), 151.9, 148.1, 128.5, 124.4 (m), 123.9, 120.7 (dd, J = 23.5, 9.7 Hz), 118.8 (dd, J = 25.5, 7.4 Hz), 115.9 (dd, J = 25.5, 7.4 Hz), 108.5, 106.9, 102.3, 96.8 (dd, J = 8.7, 3.7 Hz). HRMS (m/z) [M+H]$^+$ calcd for M = $C_{16}H_{10}F_2O_4$, 305.0625; found, 305.0625.

3-(1,3-Benzodioxol-5-yl)-5-(2,5-difluorophenyl)-1*H*-pyrazole (sery316b)

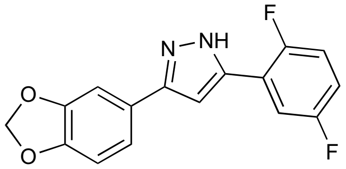

The title compound was prepared according to the GEP 2 starting from **sery316a** and purified by recrystallisation from ethanol/EtOAc to provide **sery316b** as a white solid. Yield 83%. m.p.: 225-226 °C. ^{1}H NMR (400 MHz, DMSO[D$_6$] + 1-2% conc. DCl) δ = 7.76-7.67 (m, 1H), 7.42-7.30 (m, 3H), 7.26-7.17 (m, 1H), 7.03 (d, J = 3.4 Hz, 1H), 6.99 (d, J = 8.1 Hz, 1H), 6.06 (s, 2H). ^{13}C NMR (100.6 MHz, DMSO[D$_6$]) δ = 158.3 (d, J = 239.8 Hz), 155.2 (d, J = 244.6 Hz), 147.9, 147.3, 146.4, 142.4, 124.6, 121.6, 119.2, 118.0 (dd, J = 25.3, 8.9 Hz), 115.9 (dd, J = 24.2, 8.8 Hz), 113.8 (m), 108.8, 105.8, 102.3 (d, J = 8.4 Hz), 101.3. HRMS (m/z) [M+H]$^+$ calcd for M = $C_{16}H_{10}F_2N_2O_2$, 301.0789; found, 301.0789.

5-(1,3-Benzodioxol-5-yl)-3-(3-bromophenyl)-1-(2-hydroxyethyl)-1*H*-pyrazole and 3-(1,3-Benzodioxol-5-yl)-5-(3-bromophenyl)-1-(2-hydroxyethyl)-1*H*-pyrazole (sery329)

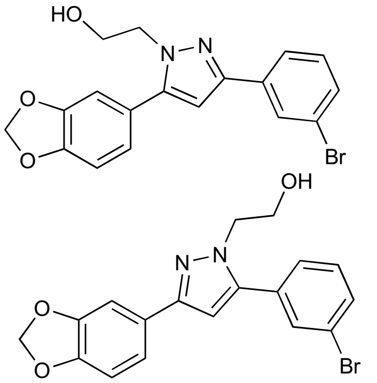

The title compounds was prepared according to the GEP 2 starting from **anle138b** and 2-hydroxyethylhydrazine and purified by column chromatography (hexane:EtOAc, 2.5:1 v/v) to provide **sery329** as a white solid (mixture of isomers 1:1). Yield 50%. TLC (hexane:EtOAc, 2.5:1 v/v): RF = 0.28. m.p.: 90-120 °C. ^{1}H NMR (400 MHz, DMSO[D$_6$]) δ = 8.08 (t, J = 1.6 Hz, 1H), 7.86 (t, J = 1.6 Hz, 1H), 7.83 (d, J = 7.8 Hz, 1H), 7.69-7.63 (m, 2H), 7.52-7.44 (m, 2H), 7.41-7.32 (m, 3H), 7.23 (d, J = 1.5 Hz, 1H), 7.11 (dd, J = 8.0, 1.5 Hz, 1H), 7.05 (d, J = 8.0 Hz, 1H), 6.95 (d, J = 8.0 Hz, 1H), 6.85 (s, 1H), 6.83 (s, 1H), 6.10 (s, 2H), 6.04 (s, 2H), 5.04-4.97 (m, 2H), 4.18-4.09 (m, 4H), 3.88-3.80 (m, 4H). ^{13}C NMR (100.6 MHz, DMSO[D$_6$]) δ = 149.3, 147.7, 147.6 (2C), 147.5, 146.8, 145.2, 143.6, 135.7, 132.5, 131.4, 131.3, 130.85, 130.8, 130.1, 127.9, 127.5, 127.4, 124.0, 123.6, 123.0, 122.1, 121.9, 118.7, 109.3, 108.5, 108.45, 105.5, 103.35, 103.3, 101.4, 101.0, 60.0, 59.9, 51.5, 51.4. HRMS (m/z) [M+H]$^+$ calcd for M = $C_{18}H_{15}{}^{79}BrN_2O_3$, 387.0344; found, 387.0345.

1-(1,3-Benzodioxol-5-yl)-3-(3-chlorophenyl)propane-1,3-dione (sery335a)

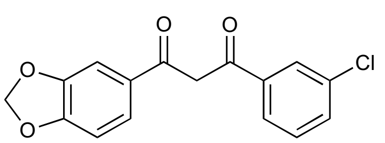

The title compound has been reported previously[12] (CAS 749927-14-8). It was prepared according to the GEP 1 using 1-(1,3-benzodioxol-5-yl)ethanone and methyl 3-chlorobenzoate and purified by recrystallisation from methanol to provide **sery335a** as a yellow solid. Yield 87%. m.p.: 127-128 °C. ^{1}H NMR (400 MHz, DMSO[D$_6$]) δ = 8.20 (s, 1H), 8.07 (d, J = 7.8 Hz, 1H), 7.82 (d, J = 8.2 Hz, 1H), 7.74 (s, 1H), 7.66 (d, J = 7.8 Hz, 1H), 7.56 (t, J = 7.8 Hz, 1H), 7.27 (s, 1H), 7.07 (d, J = 8.2 Hz, 1H), 6.16 (s, 2H) (in ^{1}H NMR spectrum 5.0% of diketone form is present). ^{13}C NMR (100.6 MHz, DMSO[D$_6$]) δ = 186.1, 181.3, 151.7, 148.0, 136.5, 133.8, 132.3, 130.6, 128.8, 126.8, 125.7, 123.9, 108.4, 107.3, 102.2, 93.1. HRMS (m/z) [M+H]$^+$ calcd for M = $C_{16}H_{11}ClO_4$, 303.0424; found, 303.0426.

3-(1,3-Benzodioxol-5-yl)-5-(3-chlorophenyl)-1*H*-pyrazole (sery335b)

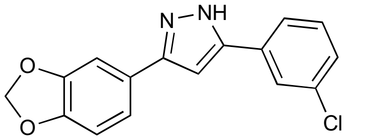

The title compound has been reported previously[12] (CAS 749927-20-6). It was prepared according to the GEP 2 starting from **sery335a** and purified by recrystallisation from methanol to provide **sery335b** as a white solid. Yield 83%. m.p.: 188-189 °C. ^{1}H NMR (400 MHz, DMSO[D$_6$] + 1-2% conc. DCl) δ = 7.87 (s, 1H), 7.78 (d, J = 7.2 Hz, 1H), 7.45 (t, J = 7.2 Hz, 1H), 7.4-7.25 (m, 3H), 7.18 (s, 1H), 7.00 (d, J = 6.9 Hz, 1H), 6.05 (s, 2H). HRMS (m/z) [M+H]$^+$ calcd for M = $C_{16}H_{11}ClN_2O_2$, 299.0587; found, 299.0587.

1-(4-Benzyloxyphenyl)-3-(3,4-dimethoxyphenyl)propane-1,3-dione (sery455a)

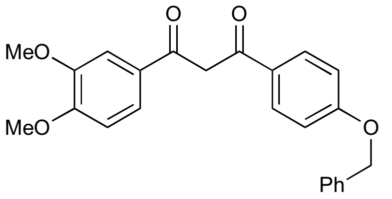

The title compound was prepared according to the GEP 1 using 1-(4-benzyloxyphenyl)ethanone and ethyl 3,4-dimethoxybenzoate and purified by recrystallisation from methanol/EtOAc to provide **sery455a** as a yellow solid. Yield 56%. m.p.: 112-113 °C. ^{1}H NMR (400 MHz, DMSO[D$_6$]) δ = 8.15 (d, J = 8.9 Hz, 2H), 7.83 (dd, J = 8.5, 2.0 Hz, 1H), 7.61 (d, J = 2.0 Hz, 1H), 7.55-7.52 (m, 2H), 7.44-7.34 (m, 3H), 7.22-7.14 (m, 3H), 7.10 (d, J = 8.6 Hz, 1H), 5.23 (s, 2H), 3.87 (s, 3H), 3.86 (s, 3H) (in ^{1}H NMR spectrum 12% of diketone form is present). ^{13}C NMR (100.6 MHz, DMSO[D$_6$]) δ = 184.6, 183.7, 162.1, 152.9, 148.7, 136.5, 129.5, 128.5, 128.0, 127.8, 127.3 (2C), 121.6, 114.9, 111.2, 110.0, 91.7, 69.5, 55.7 (2C). HRMS (m/z) [M+H]$^+$ calcd for M = $C_{24}H_{22}O_5$, 391.1545; found, 391.1542.

3-(4-Benzyloxyphenyl)-5-(3,4-dimethoxyphenyl)-1*H*-pyrazole (sery455b)

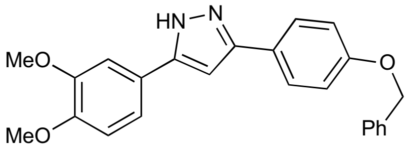

The title compound was prepared according to the GEP 2 starting from **sery455a** and purified by recrystallisation from ethanol/water to provide **sery455b** as a white solid. Yield 76%. m.p.: 131-134 °C. ^{1}H NMR (400 MHz, DMSO[D$_6$] + 1-2% conc. DCl) δ = 7.75 (d, J = 8.7 Hz, 2H), 7.50-7.31 (m, 7H), 7.09 (d, J = 8.7 Hz, 2H), 7.04-6.99 (m, 2H), 5.14 (s, 2H), 3.84 (s, 3H), 3.79 (s, 3H). ^{13}C NMR (100.6 MHz, DMSO[D$_6$] + 1-2% conc. DCl) δ = 158.0, 148.9, 148.6, 147.7-146.8 (2C), 137.0, 128.4, 127.9, 127.7, 126.4, 124.3, 124.1, 117.6, 115.1, 112.0, 109.0, 98.5, 69.3, 55.6 (2C). HRMS (m/z) [M+H]$^+$ calcd for M = $C_{24}H_{22}N_2O_3$, 387.1709; found, 387.1710.

3-(3,4-Dimethoxyphenyl)-5-(4-hydroxyphenyl)-1*H*-pyrazole (sery279b)

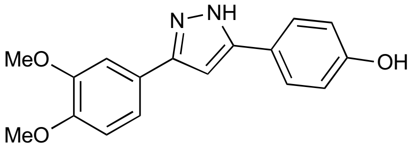

To a stirred suspension of palladium on activated carbon (10 wt %, 40 mg) in acetic acid (2 ml) under a hydrogen atmosphere a solution of **sery455b** (386 mg, 1 mmol) in acetic acid (3 ml) was added. After stirring for 3 hours under the hydrogen atmosphere at room temperature, the reaction mixture was filtered through Celite and concentrated under reduced pressure. A resulting precipitate was purified by recrystallisation from methanol/water to provide **sery279b** (210 mg, 0.71 mmol) as a white solid. Yield 71%. m.p.: 232-233 °C. ^{1}H NMR (400 MHz, DMSO[D$_6$] + 1-2% conc. DCl) δ = 9.63 (bs, 1H), 7.63 (d, J = 8.6 Hz, 2H), 7.41 (d, J = 1.9 Hz, 1H), 7.34 (dd, J = 8.4, 1.9 Hz, 1H), 6.99 (d, J = 8.1 Hz, 1H), 6.95 (s, 1H), 6.83 (d, J = 8.6 Hz, 2H), 3.83 (s, 3H), 3.78 (s, 3H). ^{13}C NMR (100.6 MHz, DMSO[D$_6$] + 1-2% conc. DCl) δ = 157.3, 149.0, 148.6, 148.4-147.4 (2C), 126.6, 125.0, 123.0, 117.6, 115.6, 112.1, 109.0, 98.2, 55.6 (2C). HRMS (m/z) [M+H]$^+$ calcd for M = $C_{17}H_{16}N_2O_3$, 297.1239; found, 297.1234.

1-(3-Benzyloxyphenyl)-3-(3,4-dimethoxyphenyl)propane-1,3-dione (sery456a)

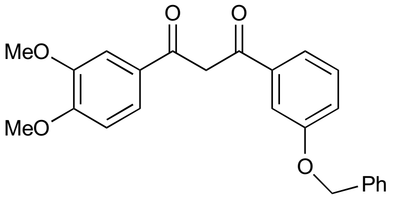

The title compound was prepared according to the GEP 1 using methyl 4-benzyloxybenzoate and 1-(3,4-dimethoxyphenyl)ethanone and purified by recrystallisation from methanol to provide **sery456a** as a yellow solid. Yield 63%. m.p.: 109-111 °C. ^{1}H NMR (400 MHz, $CDCl_3$) δ = 7.63-7.59 (m, 2H), 7.59-7.54 (m, 2H), 7.50-7.44 (m, 2H), 7.44-7.32 (m, 4H), 7.15 (dd, J = 8.2, 2.6 Hz, 1H), 6.93 (d, J = 8.5 Hz, 1H), 6.76 (s, 1H), 5.14 (s, 2H), 3.98 (s, 3H), 3.96 (s, 3H) (in ^{1}H NMR spectrum 6% of diketone form is present). ^{13}C NMR (100.6 MHz, $CDCl_3$) δ = 186.7, 183.3, 159.2, 153.1, 149.3, 137.1, 136.7, 129.8, 128.8, 128.7, 128.3, 127.7, 121.5, 119.8, 119.0, 113.3, 110.6, 109.9, 92.8, 70.4, 56.2 (2C). HRMS (m/z) [M+H]$^+$ calcd for M = $C_{24}H_{22}O_5$, 391.1545; found, 391.1541.

3-(4-Benzyloxyphenyl)-5-(3,4-dimethoxyphenyl)-1*H*-pyrazole (sery456b)

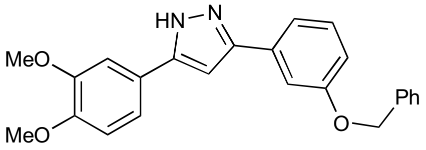

The title compound was prepared according to the GEP 2 starting from **sery456a** and purified by washing with water to provide **sery456b** as a white solid. Yield 86%. m.p.: 74-75 °C. ^{1}H NMR (400 MHz, DMSO[D$_6$] + 1-2% conc. DCl) δ = 7.53-7.47 (m, 3H), 7.45-7.31 (m, 7H), 7.15 (s, 1H), 7.03 (d, J = 8.4 Hz, 1H), 6.98 (dd, J = 8.1, 1.8 Hz, 1H), 5.17 (s, 2H), 3.84 (s, 3H), 3.79 (s, 3H). ^{13}C NMR (100.6 MHz, DMSO[D$_6$] + 1-2% conc. DCl) δ = 158.8,

149.0, 148.7, 147.5, 146.8, 137.1, 132.9, 129.9, 128.4, 127.9, 127.8, 123.8, 117.7, 117.6, 114.0, 112.0, 111.5, 109.0, 99.4, 69.3, 55.6, 55.55. HRMS (*m*/*z*) $[M+H]^+$ calcd for M = $C_{24}H_{22}N_2O_3$, 387.1709; found, 387.1708.

3-(3,4-Dimethoxyphenyl)-5-(3-hydroxyphenyl)-1*H*-pyrazole (sery310b)

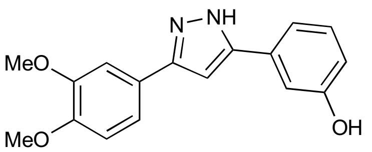

The title compound was prepared using an analogous procedure to that described for **sery279b** starting from **sery456b** and purified by recrystallisation from methanol/water to provide **sery310b** as a white solid. Yield 90%. m.p.: 115 °C (dec). ^{1}H NMR (400 MHz, DMSO[D$_6$] + 1-2% conc. DCl) δ= 9.52 (bs, 1H), 7.41 (d, *J* = 1.6 Hz, 1H), 7.36 (dd, *J* = 8.3, 1.6 Hz, 1H), 7.27-7.19 (m, 3H), 7.03 (s, 1H), 7.01 (d, *J* = 8.4 Hz, 1H), 6.74 (dt, *J* = 6.9, 2.2 Hz, 1H), 3.84 (s, 3H), 3.78 (s, 3H). ^{13}C NMR (100.6 MHz, DMSO[D$_6$] + 1-2% conc. DCl) δ= 157.8, 149.1, 148.8, 147.9, 146.9, 133.2, 129.9, 124.2, 117.8, 116.3, 114.9, 112.15, 112.1, 109.1, 99.3, 55.7 (2C). HRMS (*m*/*z*) $[M+H]^+$ calcd for M = $C_{17}H_{16}N_2O_3$, 297.1239; found, 297.1241.

1-(1,3-Benzodioxol-5-yl)-3-(3-benzyloxyphenyl)propane-1,3-dione (sery336b)

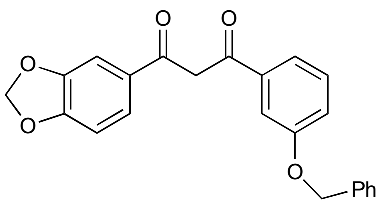

The title compound was prepared according to the GEP 1 using 1-(3-benzyloxyphenyl)ethanone and methyl ester of 1,3-benzodioxole-5-carboxylic acid and purified by column chromatography (CH_2Cl_2) followed by recrystallisation from methanol to provide **sery336b** as a yellow solid. Yield 46%. TLC (hexane:EtOAc, 9:1 v/v): RF = 0.33. m.p.: 102-103 °C. ^{1}H NMR (400 MHz, DMSO[D$_6$]) δ = 7.83 (dd, *J* = 8.2, 1.4 Hz, 1H), 7.79-7.72 (m, 3H), 7.54-7.31 (m, 6H), 7.28 (dd, *J* = 7.8, 1.7 Hz, 1H), 7.25 (s, 1H), 7.09 (d, *J* = 8.2 Hz, 1H), 6.17 (s, 2H), 5.21 (s, 2H). ^{13}C NMR (100.6 MHz, DMSO[D$_6$]) δ = 185.7, 183.0, 158.6, 151.5, 148.0, 136.8, 135.9, 129.9, 128.9, 128.4, 127.9, 127.8, 123.7, 119.8, 119.2, 113.2, 108.4, 107.2, 102.1, 92.9, 69.5. HRMS (*m*/*z*) $[M+H]^+$ calcd for M = $C_{23}H_{18}O_5$, 375.1232; found, 375.1234.

3-(1,3-Benzodioxol-5-yl)-5-(3-benzyloxyphenyl)-1*H*-pyrazole (sery339a)

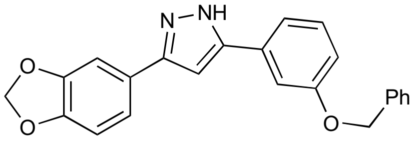

The title compound was prepared according to the GEP 2 starting from **sery336b** and purified by recrystallisation from methanol (twice) to provide **sery339a** as a white solid. Yield 74%. m.p.: 158-159 °C. ^{1}H NMR (400 MHz, DMSO[D$_6$] + 1-2% conc. DCl) δ = 7.52 (m, 3H), 7.43-7.31 (m, 7H), 7.12 (s, 1H), 7.02-6.95 (m, 2H), 6.05 (s, 2H), 5.16 (s, 2H). ^{13}C NMR (100.6 MHz, DMSO[D$_6$] + 1-2% conc. DCl) δ= 158.8, 147.8, 147.7-147.0 (2C), 147.0, 137.1, 132.9, 130.0, 128.5, 127.9, 127.8, 125.8, 118.9, 117.8, 114.2, 111.5, 108.7, 105.6, 101.2, 99.6, 69.3. HRMS (*m*/*z*) $[M+H]^+$ calcd for M = $C_{23}H_{18}N_2O_3$, 371.1396; found, 371.1391.

3-(1,3-Benzodioxol-5-yl)-5-(3-hydroxyphenyl)-1*H*-pyrazole (sery339b)

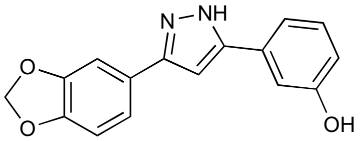

The title compound has been reported previously[12] (CAS 882697-02-1). It was prepared using an analogous procedure to that described for **sery279b** starting from **sery339a** and purified by recrystallisation from methanol/water to provide **sery339b** as a white solid. Yield 78%. m.p.: 218-220 °C. ^{1}H NMR (400 MHz, DMSO[D$_6$] + 1-2% conc. DCl) δ= 9.51 (s, 1H), 7.39 (d, *J* = 1.6 Hz, 1H), 7.33 (dd, *J* = 8.1, 1.6 Hz, 1H), 7.24-7.19 (m, 3H), 7.01 (s, 1H), 6.98 (d, *J* = 8.1 Hz, 1H), 6.77-6.70 (m, 1H), 6.05 (s, 2H). HRMS (*m*/*z*) $[M+H]^+$ calcd for M = $C_{16}H_{12}N_2O_3$, 281.0926; found, 281.0927.

Methyl 2-chloro-5-hydroxybenzoate (sery341)

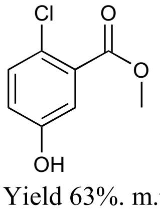

The title compound has been reported previously[22] (CAS 247092-10-0). It was prepared by a modified method[23]. A mixture of 2-chloro-5-hydroxybenzoic acid (790 mg, 4.6 mmol), concentrated sulphuric acid (0.3 ml) in methanol (15 ml) was refluxed for 16 h. The reaction mixture was then poured into cold water (20 ml) and extracted with ether (3×20 ml). The combined ethereal extracts were washed with water (20 ml), 10% aqueous $NaHCO_3$ (20 ml), dried with Na_2SO_4, and concentrated to provide **sery341** (535 mg, 2.9 mmol) as a white solid. Yield 63%. m.p.: 95-96 °C. ^{1}H NMR (400 MHz, DMSO[D$_6$]) δ= 10.06 (s, 1H), 7.34 (d, *J* = 8.7 Hz, 1H), 7.16 (d, *J* = 3.0 Hz, 1H), 6.95 (dd, *J* = 8.7, 3.0 Hz, 1H), 3.83 (s, 3H).

Methyl 5-benzyloxy-2-chlorobenzoate (sery342a)

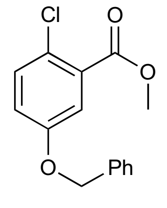

The title compound was prepared from **sery341** according to the published protocol[24] and purified by column chromatography (hexane:CH_2Cl_2, 3:2 v/v) to provide **sery342a** as a white solid. Yield 98%. TLC (hexane:EtOAc, 9:1 v/v): RF = 0.44. m.p.: 68-70 °C. ^{1}H NMR (400 MHz, DMSO[D_6]) δ = 7.48 (d, J = 8.8 Hz, 1H), 7.46-7.31 (m, 6H), 7.22 (dd, J = 8.8, 3.1 Hz, 1H), 5.15 (s, 2H), 3.85 (s, 3H). ^{13}C NMR (100.6 MHz, DMSO[D_6]) δ = 165.3, 156.9, 136.4, 131.6, 131.0, 128.5, 128.0, 127.8, 122.9, 119.7, 116.7, 69.8, 52.6. HRMS (m/z) $[M+Na]^+$ calcd for M = $C_{15}H_{13}ClO_3$, 299.0451; found, 299.0448.

1-(1,3-Benzodioxol-5-yl)-3-(5-benzyloxy-2-chlorophenyl)propane-1,3-dione (sery342b)

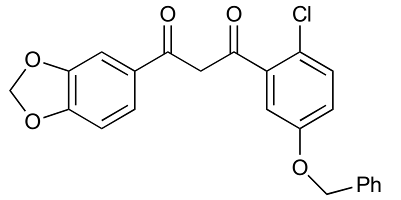

The title compound was prepared according to the GEP 1 using 1-(1,3-benzodioxol-5-yl)ethanone and **sery342a** and purified by recrystallisation from methanol to provide **sery342b** as a yellow solid. Yield 87%. m.p.: 94-96 °C. ^{1}H NMR (400 MHz, DMSO[D_6]) δ = 7.71 (dd, J = 8.2, 1.5 Hz, 1H), 7.60 (d, J = 1.5 Hz, 1H), 7.53-7.31 (m, 7H), 7.20 (dd, J = 8.8, 3.0 Hz, 1H), 7.08 (d, J = 8.2 Hz, 1H), 6.83 (s, 1H), 6.16 (s, 2H), 5.19 (s, 2H) (in ^{1}H NMR spectrum 8% of diketone form is present). ^{13}C NMR (100.6 MHz, DMSO[D_6]) δ = 184.9, 184.7, 157.1, 151.8, 148.1, 136.5, 136.0, 131.5, 128.5, 128.3, 128.0, 127.8, 123.7, 122.0, 118.8, 116.3, 108.5, 106.9, 102.2, 97.4, 69.8. HRMS (m/z) $[M+H]^+$ calcd for M = $C_{23}H_{17}ClO_5$, 409.0843; found, 409.0845.

3-(1,3-Benzodioxol-5-yl)-5-(5-benzyloxy-2-chlorophenyl)-1*H*-pyrazole (sery342c)

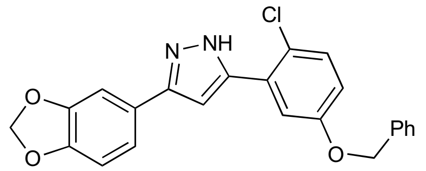

The title compound was prepared according to the GEP 2 starting from **sery342b** and purified by recrystallisation from methanol to provide **sery342c** as a white solid. Yield 84%. m.p.: 122-123 °C. ^{1}H NMR (400 MHz, DMSO[D_6] + 1-2% conc. DCl) δ = 7.49-7.29 (m, 9H), 7.09-7.02 (m, 2H), 6.99 (d, J = 8.1 Hz, 1H), 6.06 (s, 2H), 5.16 (s, 2H). ^{13}C NMR (100.6 MHz, DMSO[D_6] + 1-2% conc. DCl) δ = 157.2, 147.9, 147.1, 146.0, 145.3, 136.7, 131.6, 131.3, 128.5, 128.0, 127.8, 125.3, 122.6, 119.1, 116.2 (2C), 108.8, 105.7, 102.9, 101.3, 69.7. HRMS (m/z) $[M+H]^+$ calcd for M = $C_{23}H_{17}ClN_2O_3$, 405.1006; found, 405.1013.

3-(1,3-Benzodioxol-5-yl)-5-(2-chloro-5-hydroxyphenyl)-1*H*-pyrazole (sery344)

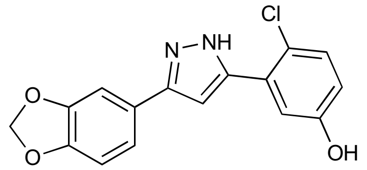

The title compound was prepared using an analogous procedure to that described for **sery279b** starting from **sery342c** and purified by recrystallisation from methanol/water to provide **sery344** as a white solid. Yield 93%. m.p.: 193-195 °C. ^{1}H NMR (400 MHz, DMSO[D_6] + 1-2% conc. DCl) δ = 9.80 (bs, 1H), 7.39 (d, J = 1.6 Hz, 1H), 7.35-7.28 (m, 2H), 7.15 (d, J = 2.9 Hz, 1H), 7.02-6.96 (m, 2H), 6.80 (dd, J = 8.6, 2.9 Hz, 1H), 6.06 (s, 2H). ^{13}C NMR (100.6 MHz, DMSO[D_6] + 1-2% conc. DCl) δ = 156.3, 147.7, 146.9, 145.8, 145.2, 131.8, 131.0, 125.6, 120.6, 119.0, 116.7, 116.6, 108.7, 105.6, 102.7, 101.2. HRMS (m/z) $[M+H]^+$ calcd for M = $C_{16}H_{11}ClN_2O_3$, 315.0536; found, 315.0539.

1-(1,3-Benzodioxol-5-yl)-3-(4-bromophenyl)propane-1,3-dione (sery378a)

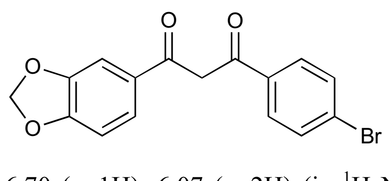

The title compound has been reported previously[25] (CAS 87724-01-4). It was prepared according to the GEP 1 using 1-(1,3-benzodioxol-5-yl)ethanone and methyl 4-bromobenzoate and purified by recrystallisation from ethanol/EtOAc to provide **sery378a** as a yellow solid. Yield 76%. m.p.: 158-160 °C. ^{1}H NMR (400 MHz, $CDCl_3$) δ = 7.82 (d, J = 8.6 Hz, 2H), 7.66-7.55 (m, 3H), 7.45 (d, J = 1.5 Hz, 1H), 6.89 (d, J = 8.2 Hz, 1H), 6.70 (s, 1H), 6.07 (s, 2H) (in ^{1}H NMR spectrum 2.5% of diketone form is present). ^{13}C NMR (100.6 MHz, $CDCl_3$) δ = 186.3, 182.7, 151.8, 148.4, 134.4, 132.1, 130.1, 128.6, 127.2, 123.2, 108.4, 107.4, 102.1, 92.6.

3-(1,3-Benzodioxol-5-yl)-5-(4-bromophenyl)-1*H*-pyrazole (sery378b)

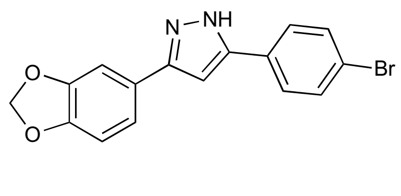

The title compound has been reported previously[25] (CAS 651717-21-4). It was prepared according to the GEP 2 starting from **sery378a** and purified by recrystallisation from ethanol/EtOAc to provide **sery378b** as

a white solid. Yield 75%. m.p.: 231-233 °C. ^{1}H NMR (400 MHz, DMSO[D$_6$]) δ = 13.29 (bs, 1H), 7.77 (d, J = 7.8 Hz, 2H), 7.63 (d, J = 7.7 Hz, 2H), 7.37 (s, 1H), 7.32 (d, J = 7.8 Hz, 1H), 7.12 (s, 1H), 7.00 (d, J = 7.9 Hz, 1H), 6.06 (s, 2H). ^{13}C NMR (100.6 MHz, DMSO[D$_6$] + 1-2% conc. DCl) δ = 148.0, 147.4, 147.3, 146.7, 131.9, 131.4, 127.3, 125.1, 120.9, 119.2, 108.9, 105.8, 101.4, 99.7.

Methyl 3-(2-fluoroethoxy)benzoate (sery387)

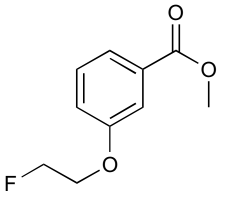

The title compound has been reported previously[26] (CAS 132838-27-8). To a solution of methyl 3-hydroxybenzoate (1520 mg, 10 mmol) in DMF (15 ml) 60 % suspension of sodium hydride in mineral oil (420 mg, 10.5 mmol) was added in small portions, resulting mixture was stirred for 15 min at room temperature under argon atmosphere and treated with 2-fluoroethyl ester of 4-methylbenzenesulfonic acid (2.4 g, 11 mmol). After stirring overnight at room temperature solvent was evaporated under reduced pressure and a remaining oil was passed though a small pad of silica gel eluting with CH_2Cl_2 to provide **sery387** (1550 mg, 78%) as a colourless oil. ^{1}H NMR (400 MHz, DMSO[D$_6$]) δ = 7.57 (d, J = 7.7 Hz, 1H), 7.49-7.41 (m, 2H), 7.27 (dd, J = 8.2, 2.2 Hz, 1H), 4.75 (m, 2H), 4.30 (m, 2H), 3.85 (s, 3H).

1-(1,3-Benzodioxol-5-yl)-3-[3-(2-fluoroethoxy)phenyl]propane-1,3-dione (sery388a)

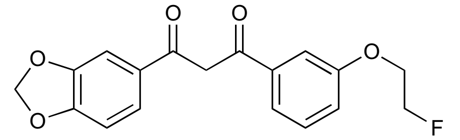

The title compound was prepared according to the GEP 1 using 1-(1,3-benzodioxol-5-yl)ethanone and **sery387** and purified by recrystallisation from methanol to provide **sery388a** as a yellow solid. Yield 54%. m.p.: 117-119 °C. ^{1}H NMR (400 MHz, DMSO[D$_6$]) δ = 7.84 (dd, J = 8.2, 1.6 Hz, 1H), 7.79-7.73 (m, 2H), 7.69 (s, 1H), 7.48 (t, J = 8.0 Hz, 1H), 7.27 (s, 1H), 7.24 (dd, J = 8.2, 2.4 Hz, 1H), 7.09 (d, J = 8.2 Hz, 1H), 6.17 (s, 2H), 4.79 (ddd, J = 47.7, 4.2, 3.5 Hz, 2H), 4.36 (ddd, J = 30.2, 4.5, 3.2 Hz, 2H) (in ^{1}H NMR spectrum 4% of diketone form is present). ^{13}C NMR (100.6 MHz, DMSO[D$_6$]) δ = 185.8, 182.9, 158.5, 151.6, 148.0, 136.0, 130.0, 129.0, 123.7, 120.0, 119.0, 112.8, 108.4, 107.2, 102.1, 92.9, 82.1 (d, J = 167.8 Hz), 67.3 (d, J = 19.4 Hz). HRMS (m/z) [M+H]$^+$ calcd for M = $C_{18}H_{15}FO_5$, 331.0982; found, 331.0976.

3-(1,3-Benzodioxol-5-yl)-5-[3-(2-fluoroethoxy)phenyl]-1*H*-pyrazole (sery369)

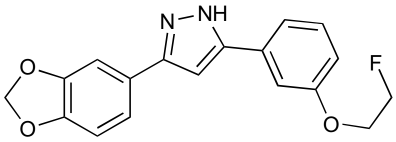

The title compound was prepared according to the GEP 2 starting from **sery388a** and purified by recrystallisation from ethanol to provide **sery369** as a white solid. Yield 82%. m.p.: 167-168 °C. ^{1}H NMR (400 MHz, DMSO[D$_6$] + 1-2% conc. DCl) δ = 13.22 (bs, 1H), 7.47-7.30 (m, 5H), 7.13 (s, 1H), 7.69 (d, J = 8.0 Hz, 1H), 6.93 (d, J = 7.9 Hz, 1H), 6.05 (s, 2H), 4.77 (ddd, J = 30.2, 4.0, 2.7 Hz, 2H), 4.30 (ddd, J = 47.3, 4.2, 2.5 Hz, 2H). ^{13}C NMR (100.6 MHz, DMSO[D$_6$] + 1-2% conc. DCl) δ = 158.7, 147.9, 147.4, 147.2, 147.0, 133.0, 130.2, 125.8, 119.0, 118.0, 114.0, 111.3, 108.8, 105.7, 101.3, 99.7, 82.3 (d, J = 166.4 Hz), 67.2 (d, J = 18.4 Hz). HRMS (m/z) [M+H]$^+$ calcd for M = $C_{18}H_{15}FN_2O_3$, 327.1145; found, 327.1144.

Methyl ester of imidazo[1,2-a]pyridine-7-carboxylic acid (sery391)

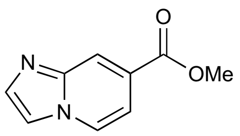

The title compound has been reported previously[27] (CAS 86718-01-6). It was prepared according to the published protocol[27] and purified by column chromatography ($CHCl_3$:MeOH, 100:1 v/v) followed by recrystallisation from hexane/EtOAc to provide **sery391** as a yellow solid. Yield 94%. TLC ($CHCl_3$:MeOH, 100:1 v/v): RF = 0.22. m.p.: 143 °C. ^{1}H NMR (400 MHz, DMSO[D$_6$]) δ = 8.37 (s, 1H), 8.17 (d, J = 7.1 Hz, 1H), 7.80 (s, 1H), 7.69 (s, 1H), 7.40 (d, J = 7.1, 1.4 Hz, 1H), 3.96 (s, 3H).

1-(3-Bromophenyl)-3-(imidazo[1,2-a]pyridin-7-yl)propane-1,3-dione (sery401a)

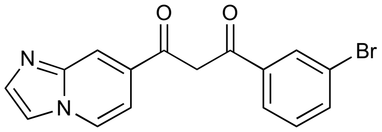

The title compound was prepared according to the GEP 1 using 1-(3-bromophenyl)ethanone and **sery391** and purified by recrystallisation from methanol to provide **sery388a** as a yellow solid. Yield 49%. m.p.: 188-189 °C. ^{1}H NMR (400 MHz, DMSO[D$_6$]) δ = 8.73-8.65 (m, 2H), 8.44 (s, 1H), 8.23-8.12 (m, 2H), 7.88-7.79 (m, 2H), 7.61-7.49 (m, 3H) (in ^{1}H NMR

spectrum 5% of diketone form is present). ^{13}C NMR (100.6 MHz, DMSO[D$_6$]) δ = 184.8, 182.1, 143.8, 136.5, 136.1, 135.5, 130.9, 130.0, 129.9, 127.0, 126.3, 122.3, 117.9, 115.1, 109.1, 93.8. HRMS (*m/z*) [M+H]$^+$ calcd for M = $C_{16}H_{11}{}^{79}BrN_2O_2$, 343.0082; found, 343.0088.

7-[3-(3-bromophenyl)-1*H*-pyrazol-5-yl]imidazo[1,2-a]pyridine (sery401b)

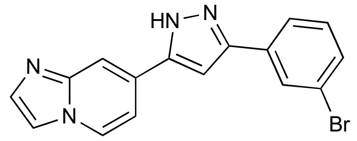

The title compound was prepared according to the GEP 2 starting from **sery401a** and purified by refluxing in EtOAc/ethanol to provide **sery401b** as a white solid. Yield 78%. m.p.: 282-283 °C. ^{1}H NMR (400 MHz, DMSO[D$_6$] + 1-2% conc. DCl) δ = 13.62 (bs, 1H), 8.62 (d, J = 6.5 Hz, 1H), 8.06 (s, 1H), 8.02 (s, 1H), 7.96 (s, 1H), 7.86 (d, J = 7.4 Hz, 1H), 7.61 (s, 1H), 7.54 (d, J = 7.8 Hz, 1H), 7.48 (s, 1H), 7.46-7.34 (m, 2H). ^{13}C NMR (100.6 MHz, DMSO[D$_6$] + 1-2% conc. DCl) δ = 149.9, 144.6, 142.3, 138.1, 134.0, 132.8, 131.1, 130.6, 127.6, 127.2, 124.1, 122.3, 113.4, 111.6, 109.8, 101.1. HRMS (*m/z*) [M+H]$^+$ calcd for M = $C_{16}H_{11}{}^{79}BrN_4$, 339.0245; found, 339.0249.

3-(3-Bromophenyl)-5-(2,2-difluoro-1,3-benzodioxol-5-yl)-1*H*-pyrazole (anle270)

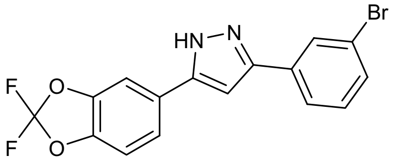

The title compound was prepared by the modified published protocol[28]. To a mixture of 2,2-difluoro-1,3-benzodioxole-5-carboxylic acid (606 mg, 3.0 mmol) and 2,3,4,5,6-pentafluorophenol (570 mg, 3.1 mmol) in dry dioxane (15 mL) 1,3-dicyclohexylcarbodiimide (640 mg, 3.1 mmol) was added. The mixture was stirred for 15 h at room temperature. Dicyclohexylurea formed was filtered off, the solvent was evaporated under reduced pressure, the residue was mixed with dry toluene (15 ml), the small amount of dicyclohexylurea was filtered off. The residue was concentrated under reduced pressure and redissolved in dry toluene (8 ml) to provide a solution of 2,2-difluoro-1,3-benzodioxole-5-carboxylic acid 2,3,4,5,6-pentafluorophenyl ester. In a separate flask, hexamethyldisilazane (1.251 ml, 968 mg, 6.0 mmol) was dissolved in 15 mL of dry cyclohexane in a screw cap vial (with septum), and then the solution was cooled to 0 °C under nitrogen. *n*BuLi (2.4 ml, 2.5 M in hexanes, 6.0 mmol) was added via syringe, the resulting mixture was stirred for 30 min. 1-(3-Bromophenyl)ethanone (797 µl, 1.194 g, 6.0 mmol) was added quickly via syringe, the resulting mixture was stirred at 0 °C for 15 min, followed by the addition of 2,2-difluoro-1,3-benzodioxole-5-carboxylic acid 2,3,4,5,6-pentafluorophenyl ester solution in toluene in one portion with stirring then allowed to stir at room temperature for 15 min. AcOH (6 ml) and EtOH (30 ml) were added to form a homogeneous mixture, then hydrazine hydrate (4.5 ml, 4.64 g, 92.7 mmol) was added, and the resulting mixture was heated under reflux for 30 min with stirring. After cooling the solvent was evaporated under reduced pressure, the residue was stirred 30 min with saturated sodium hydrogen carbonate solution (60 ml), the resulting precipitate was collected by filtration, washed with water, dried and recrystallized from $CHCl_3$/cyclohexane to provide **anle270** (733 mg, 64%) as a white solid. m.p.: 202-203 °C. ^{1}H NMR (400 MHz, DMSO[D$_6$] + 1-2% conc. DCl) δ = 8.00 (t, J = 1.7 Hz, 1H), 7.85-7.76 (m, 2H), 7.67 (dd, J = 8.4, 1.6 Hz, 1H), 7.52 (d, J = 7.8 Hz, 1H), 7.47 (d, J = 8.4 Hz, 1H), 7.41 (t, J = 7.8 Hz, 1H), 7.30 (s, 1H). ^{13}C NMR (100.6 MHz, DMSO[D$_6$] + 1-2% conc. DCl) δ = 147.3, 146.9, 143.6, 142.5, 133.6, 131.5 (t, J = 253.1 Hz), 131.4, 130.9, 128.7, 127.8, 124.3, 122.6, 121.6, 110.8, 107.1, 100.9. HRMS (*m/z*) [M+H]$^+$ calcd for M = $C_{16}H_9{}^{79}BrF_2N_2O_2$, 378.9894; found, 378.9896.

3-(4-Chlorophenyl)-5-(2-nitrophenyl)-1*H*-pyrazole (sery309)

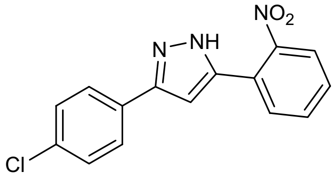

The title compound was prepared analogously to the procedure for **anle270** using 2-nitrobenzoic acid and 1-(4-chlorophenyl)ethanone. The reaction mixture was stirred at reflux for 2 h after addition of hydrazine hydrate. The crude product after aqueous workup was purified by column chromatography on silica gel (hexane:EtOAc, 2:1 v/v) followed by recrystallisation from methanol/water to provide **sery309** as a gray solid. Yield 48%. TLC (hexane:EtOAc, 2:1 v/v): RF = 0.35. m.p.: 135-137 °C. ^{1}H NMR (400 MHz, DMSO[D$_6$] + 1-2% conc. DCl) δ = 7.85 (d, J = 8.0 Hz, 1H), 7.83-7.76 (m, 3H), 7.72 (dd, J = 7.5, 1.0 Hz, 1H), 7.58 (dd, J = 8.0, 1.3 Hz, 1H), 7.52 (d, J = 8.6 Hz, 2H), 7.01 (s, 1H). ^{13}C NMR (100.6 MHz, DMSO[D$_6$] + 1-2% conc. DCl) δ = 148.8, 145.9, 144.1, 133.0, 132.5, 130.6, 130.2, 129.3, 128.9, 127.1, 125.9, 123.9, 102.2. HRMS (*m/z*) [M+H]$^+$ calcd for M = $C_{15}H_{10}ClN_3O_2$, 300.0540; found, 300.0542.

1-Methoxymethyl-1*H*-benzimidazole-5-carboxylic acid methyl ester and 1-methoxymethyl-1*H*-benzimidazole-6-carboxylic acid methyl ester (sery406)

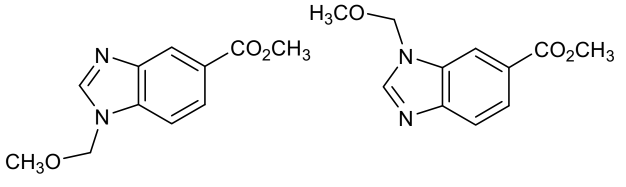

To a solution of methyl 1*H*-benzimidazole-6-carboxylate (2.64 g, 15 mmol) in THF (45 ml) a dispersion of sodium hydride (60% in oil, 700 mg, 17.5 mmol) was added in portions over a period of 15 min at 0 °C and stirred for 15 min. Methoxymethyl chloride (1.52 ml, 1.61 g, 20 mmol) was added, the mixture was stirred 1.5 h at room temperature and poured into cold saturated solution of $NaHCO_3$ (150 ml). The solution was extracted with Et_2O (2 × 60 ml), the combined organic fractions were washed with brine (50 ml), dried over Na_2SO_4 and evaporated under reduced pressure. The resulting oil was purified by column chromatography on silica gel ($CHCl_3$:MeOH, 100:5 v/v) to provide **sery406** (2.64 g, 80%) as a inseparable mixture of 5- and 6-isomers in ratio 1:1 (^{1}H NMR). Viscous red oil. TLC ($CHCl_3$:MeOH, 100:5 v/v): RF = 0.39. ^{1}H NMR (400 MHz, DMSO[D_6]) δ = 8.57 (s, 1H), 8.53 (s, 1H), 8.28 (s, 2H), 7.92 (dd, *J* = 8.4, 1.5 Hz, 1H), 7.87 (dd, *J* = 8.4, 1.6 Hz, 1H), 7.77 (d, *J* = 5.6 Hz, 1H), 7.76 (d, *J* = 5.6 Hz, 1H), 5.69 (s, 2H), 5.64 (s, 2H), 3.87 (s, 3H), 3.86 (s, 3H), 3.21 (s, 3H), 3.20 (s, 3H). ^{13}C NMR (100.6 MHz, DMSO[D_6]) δ = 166.8, 166.7, 147.7, 147.3, 146.8, 143.5, 137.2, 133.4, 124.4, 124.09, 124.05, 123.4, 121.4, 119.7, 113.0, 111.2, 75.53, 75.49, 55.9, 55.8, 52.26, 52.21. HRMS (*m/z*) [M+H]$^+$ calcd for M = $C_{11}H_{12}N_2O_3$, 221.0926; found, 221.0921.

1-Methoxymethyl-1*H*-benzimidazole-5-carboxylic acid and 1-methoxymethyl-1*H*-benzimidazole-6-carboxylic acid (sery407)

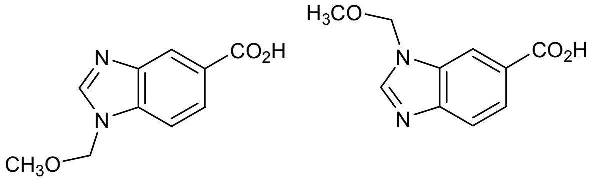

To a solution of **sery406** (1.20 g, 5.45 mmol) in MeOH (2.5 ml) an aqueous 2 M LiOH solution (2.73 ml, 5.46 mmol) was added and stirred 15 h at room temperature. The solvent was evaporated under reduced pressure, the residue was acidified with 1 M HCl (5.5 ml, 5.5 mmol) at 0 °C and stirred for 30 min. The product was collected by filtration and dried to provide **sery407** (960 mg, 86%) as a inseparable mixture of 5- and 6-isomers in ratio 1:1 (^{1}H NMR). Brown solid. m.p.: 153-157 °C. ^{1}H NMR (400 MHz, DMSO[D_6]) δ = 12.83 (br s, 2H), 8.54 (s, 1H), 8.50 (s, 1H), 8.26 (s, 2H), 7.92 (dd, *J* = 8.4, 1.3 Hz, 1H), 7.86 (dd, *J* = 8.4, 1.4 Hz, 1H), 7.75 (d, *J* = 5.1 Hz, 1H), 7.73 (d, *J* = 5.1 Hz, 1H), 5.68 (s, 2H), 5.64 (s, 2H), 3.21 (s, 3H), 3.20 (s, 3H). ^{13}C NMR (100.6 MHz, DMSO[D_6]) δ = 167.9, 167.8, 147.4, 147.1, 146.6, 143.4, 136.9, 133.4, 125.5, 125.2, 124.3, 123.6, 121.5, 119.5, 113.1, 110.9, 75.49, 75.46, 66.9, 55.8. HRMS (*m/z*) [M+H]$^+$ calcd for M = $C_{10}H_{10}N_2O_3$, 207.0770; found, 207.0760.

6-[5-(3-Bromophenyl)-1*H*-pyrazol-3-yl]-1-methoxymethyl-1*H*-benzimidazole and 5-[5-(3-bromophenyl)-1*H*-pyrazol-3-yl]-1-methoxymethyl-1*H*-benzimidazole (sery415)

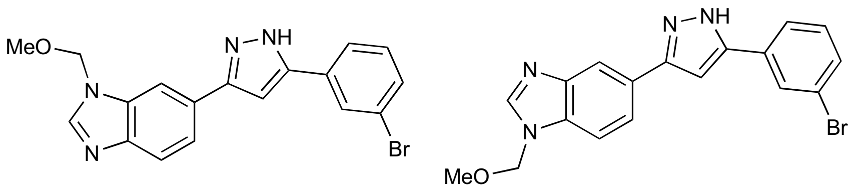

The title compound was prepared analogously to the procedure for **anle270** using **sery407** and 1-(3-bromophenyl)ethanone. The crude product after aqueous work up was purified by column chromatography on silica gel ($CHCl_3$:MeOH, 10:1 v/v) to provide **sery415** as a inseparable mixture of 5- and 6-isomers in ratio 7:3 (^{1}H NMR). White solid. Yield 49%. m.p. 189-198 °C. TLC ($CHCl_3$:MeOH, 10:1 v/v): RF = 0.45. ^{1}H NMR (400 MHz, DMSO[D_6] + 1-2% conc. DCl) δ = 8.45 (s, 1H), 8.16 and 8.13 (2 × s, 1H), 8.05 (s, 1H), 7.86 (d, *J* = 7.6 Hz, 1H), 7.83-7.69 (m, 2H), 7.50 (d, *J* = 7.7 Hz, 1H), 7.40 (t, *J* = 7.7 Hz, 1H), 7.33 and 7.31 (2 × s, 1H), 5.65 and 5.63 (2 × s, 2H), 3.25 and 3.23 (2 × s, 3H). ^{13}C NMR (100.6 MHz, DMSO[D_6] + 1-2% conc. DCl) δ = 146.5, 145.5, 144.5, 143.7, 143.1, 134.8, 134.0, 133.5, 131.1, 130.3, 127.6, 124.1, 122.3, 121.0, 120.2, 119.8, 116.0, 111.5, 107.6, 100.4, 100.1, 75.3, 55.8. HRMS (*m/z*) [M+H]$^+$ calcd for M = $C_{18}H_{15}{}^{79}BrN_4O$, 383.0507; found, 383.0507.

5-[5-(3-Bromophenyl)-1*H*-pyrazol-3-yl]-1*H*-benzimidazole (sery417)

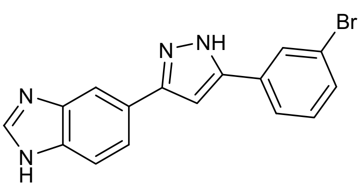

The title compound was prepared according to the GEP 3 starting from **sery415** and purified by column chromatography on silica gel ($CHCl_3$:MeOH, 10:1 v/v). Yiellow solid. Yield 54%. TLC ($CHCl_3$:MeOH, 10:1 v/v): RF = 0.24. m.p.: 164-166 °C. ^{1}H NMR (400 MHz, DMSO[D_6] + 1-2% conc. DCl) δ = 13.43 (bs, 1H), 8.30 (s, 1H), 8.11-8.03 (m, 2H), 7.87 (d, *J* = 7.8 Hz, 1H), 7.81 (d, *J* = 7.8 Hz, 1H), 7.64-7.56 (m, 2H), 7.40 (t, *J* = 7.8

Hz, 1H), 7.28 (s, 1H). ^{13}C NMR (100.6 MHz, DMSO[D$_6$] + 1-2% conc. DCl) δ = 150.0, 144.9, 143.1, 138.5, 138.0, 136.2, 131.0, 130.3, 127.6, 124.1, 123.4, 122.3, 120.0, 115.8, 112.0, 100.0. HRMS (*m*/*z*) [M+H]$^+$ calcd for M = $C_{16}H_{11}{}^{79}BrN_4$, 339.0245; found, 339.0244.

(*E*)-3-(4-Chlorophenyl)-1-(3-nitrophenyl)-2-propene-1-one (sery276a)

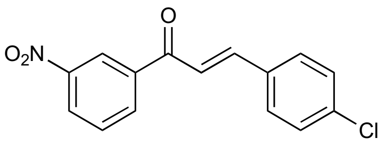

The title compound has been reported previously[29] (CAS 131497-20-6). It was prepared according to the GEP 5 using 1-(3-nitrophenyl)ethanone, 4-chlorobenzaldehyde and purified by refluxing in ethanol to provide **sery276a** as a yellow solid. Yield 98%. m.p.: 197-198 °C (lit[29] 197.5 °C). ^{1}H NMR (400 MHz, DMSO[D$_6$]) δ = 8.85 (t, *J* = 2.0 Hz, 1H), 8.61 (dt, *J* = 7.8, 1.1 Hz, 1H), 8.50 (ddd, *J* = 8.1, 2.2, 1.0 Hz, 1H), 8.07 (d, *J* = 15.6 Hz, 1H), 7.99 (d, *J* = 8.6 Hz, 2H), 7.89 (t, *J* = 8.0 Hz, 1H), 7.83 (d, *J* = 15.6 Hz, 1H), 7.56 (d, *J* = 8.6 Hz, 2H).

2,3-Dibromo-3-(4-chlorophenyl)-1-(3-nitrophenyl)propane-1-one (sery277a)

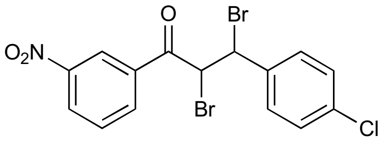

The title compound has been reported previously[30] (CAS 1032418-75-9). It was prepared according to the GEP 6 starting from **sery276a** to provide **sery277a** as a white solid. Yield 81%. m.p.: 173-175 °C. ^{1}H NMR (400 MHz, DMSO[D$_6$]) δ = 9.01 (t, *J* = 2.0 Hz, 1H), 8.71 (d, *J* = 8.0 Hz, 1H), 8.59 (ddd, *J* = 8.2, 2.0, 0.8 Hz, 1H), 7.96 (t, *J* = 8.0 Hz, 1H), 7.90 (d, *J* = 8.5 Hz, 2H), 7.53 (d, *J* = 8.5 Hz, 2H), 6.90 (d, *J* = 11.3 Hz, 1H), 5.89 (d, *J* = 11.3 Hz, 1H).

3-(4-Chlorophenyl)-5-(3-nitrophenyl)-1*H*-pyrazole (sery278a)

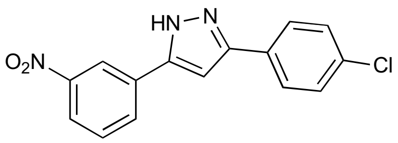

The title compound was prepared according to the GEP 7 starting from **sery277a** and purified by recrystallisation from methanol to provide **sery278a** as a white solid. Yield 60%. m.p.: 240-241 °C. ^{1}H NMR (400 MHz, DMSO[D$_6$] + 1-2% conc. DCl) δ = 8.64 (s, 1H), 8.26 (d, *J* = 7.9 Hz, 1H), 8.16 (d, *J* = 7.9 Hz, 1H), 7.85 (d, *J* = 8.5 Hz, 2H), 7.74 (t, *J* = 7.9 Hz, 1H), 7.52 (d, *J* = 8.5 Hz, 2H), 7.42 (s, 1H). ^{13}C NMR (100.6 MHz, DMSO[D$_6$] + 1-2% conc. DCl) δ = 148.5, 146.8, 145.9, 133.7, 132.8, 131.5, 130.6, 129.6, 129.1, 127.0, 122.5, 119.4, 101.1. HRMS (*m*/*z*) [M+H]$^+$ calcd for M = $C_{15}H_{10}ClN_3O_2$, 300.0540; found, 300.0544.

3-(3-Aminophenyl)-5-(4-chlorophenyl)-1*H*-pyrazole (sery280a)

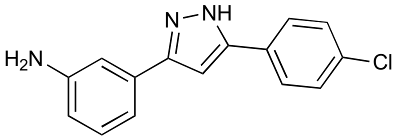

The title compound was prepared according to the GEP 8 starting from **sery278a** and purified by column chromatography ($CHCl_3$:MeOH, 19:1 v/v) to provide **sery280a** as a white solid. Yield 28%. m.p.: 183 °C. ^{1}H NMR (400 MHz, DMSO[D$_6$]) δ = 13.25 (bs, 1H), 7.85 (d, *J* = 8.2 Hz, 2H), 7.49 (d, *J* = 8.2 Hz, 2H), 7.09 (t, *J* = 7.7 Hz, 1H), 7.04-6.86 (m, 3H), 6.55 (d, *J* = 7.7 Hz, 1H), 5.15 (s, 2H). ^{13}C NMR (100.6 MHz, DMSO[D$_6$] + 1-2% conc. DCl) δ = 147.3, 145.3, 133.4, 132.5, 132.4, 130.3, 129.5, 128.9, 126.9, 124.8, 122.6, 119.8, 100.4. HRMS (*m*/*z*) [M+H]$^+$ calcd for M = $C_{15}H_{12}ClN_3$, 270.0798; found, 270.0799.

(*E*)-3-(4-Chlorophenyl)-1-(4-nitrophenyl)-2-propene-1-one (sery276b)

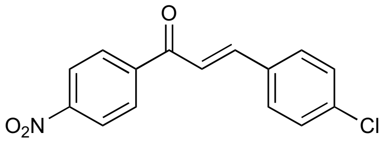

The title compound has been reported previously[31] (CAS 315180-19-9). It was prepared according to the GEP 5 using 1-(4-nitrophenyl)ethanone, 4-chlorobenzaldehyde and purified by refluxing in ethanol to provide **sery276b** as a yellow solid. Yield 87%. m.p.: 163-165 °C (lit[31] 154-156 °C). ^{1}H NMR (400 MHz, DMSO[D$_6$]) δ = 8.37 (bs, 4H), 8.03-7.93 (m, 3H), 7.8 (d, *J* = 15.6 Hz, 1H), 7.55 (d, *J* = 8.4 Hz, 2H).

2,3-Dibromo-3-(4-chlorophenyl)-1-(4-nitrophenyl)propane-1-one (sery277b)

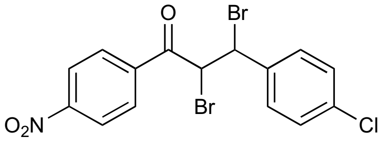

The title compound has been reported previously[32] (CAS 24213-20-5). It was prepared according to the GEP 6 starting from **sery276b** to provide **sery277b** as a white solid. Yield 78%. m.p.: 154-156 °C (lit[32] 150-153 °C). ^{1}H NMR (400 MHz, DMSO[D$_6$]) δ = 8.53 (d, *J* = 8.9 Hz, 2H), 8.45 (d, *J* =

8.9 Hz, 2H), 7.89 (d, J = 8.5 Hz, 2H), 7.53 (d, J = 8.5 Hz, 2H), 6.83 (d, J = 11.3 Hz, 1H), 5.87 (d, J = 11.3 Hz, 1H).

3-(4-Chlorophenyl)-5-(4-nitrophenyl)-1*H*-pyrazole (sery278b)

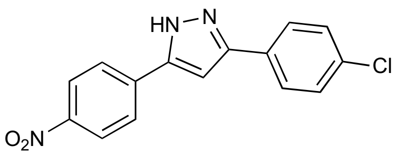

The title compound has been reported previously[21] (CAS 106961-02-8). It was prepared according to the GEP 7 starting from **sery277b** and purified by recrystallisation from methanol (twice) to provide **sery278b** as a white solid. Yield 50%. m.p.: 271-272 °C (lit[21] 280 °C). ^{1}H NMR (400 MHz, DMSO[D$_6$]) δ = 13.77 (s, 1H), 8.31 (d, J = 8.6 Hz, 2H), 8.10 (d, J = 8.6 Hz, 2H), 7.85 (d, J = 8.2 Hz, 2H), 7.55 (d, J = 8.2 Hz, 2H), 7.45 (s, 1H).

3-(4-Aminophenyl)-5-(4-chlorophenyl)-1*H*-pyrazole (sery280b)

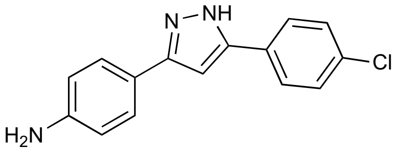

The title compound was prepared according to the GEP 8 starting from **sery278b** and purified by column chromatography (CHCl$_3$:MeOH, 100:1 v/v) to provide **sery280b** as a white solid. Yield 22%. m.p.: 213-214 °C. ^{1}H NMR (400 MHz, DMSO[D$_6$]) δ = 13.01 (s, 1H), 7.84 (d, J = 8.2 Hz, 2H), 7.46 (d, J = 5.1 Hz, 4H), 6.92 (s, 1H), 6.62 (d, J = 8.5 Hz, 2H), 5.32 (s, 2H). ^{13}C NMR (100.6 MHz, DMSO[D$_6$]) δ = 149.8, 148.8, 144.8, 133.0, 131.8, 128.6, 126.7, 126.2, 116.9, 113.8, 97.6. HRMS (*m*/*z*) [M+H]$^+$ calcd for M = $C_{15}H_{12}ClN_3$, 270.0798; found, 270.0798.

(*E*)-3-(3-Chlorophenyl)-1-(4-nitrophenyl)-2-propene-1-one (sery379a)

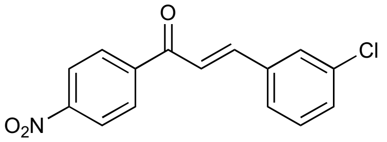

The title compound has been reported previously[33] (CAS 63831-41-4). It was prepared according to the GEP 5 using 1-(4-nitrophenyl)ethanone, 3-chlorobenzaldehyde and purified by recrystallisation from EtOAc/ethanol to provide **sery379a** as a yellow solid. Yield 81%. m.p.: 144-146 °C. ^{1}H NMR (400 MHz, DMSO[D$_6$]) δ = 8.42-8.34 (m, 4H), 8.10 (s, 1H), 8.05 (d, J = 15.7 Hz, 1H), 7.85 (d, J = 7.1 Hz, 1H), 7.77 (d, J = 15.7 Hz, 1H), 7.55-7.46 (m, 2H).

3-(3-Chlorophenyl)-2,3-dibromo-1-(4-nitrophenyl)-propane-1-one (sery380a)

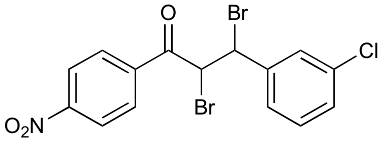

The title compound has been reported previously[34] (CAS 649739-74-2). It was prepared according to the GEP 6 starting from **sery379a** to provide **sery380a** as a yellow solid. Yield 88%. m.p.: 139-141 °C. ^{1}H NMR (400 MHz, DMSO[D$_6$]) δ = 8.54 (d, J = 8.9 Hz, 2H), 8.46 (d, J = 8.9 Hz, 2H), 8.04 (s, 1H), 7.80 (dt, J = 6.5, 2.0 Hz, 1H), 7.52-7.42 (m, 2H), 6.84 (d, J = 11.3 Hz, 1H), 5.87 (d, J = 11.3 Hz, 1H).

3-(3-Chlorophenyl)-5-(4-nitrophenyl)-1*H*-pyrazole (sery381a)

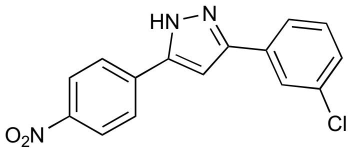

The title compound was prepared according to the GEP 7 starting from **sery380a** and purified by recrystallisation from methanol to provide **sery381a** as a yellow solid. Yield 25%. m.p.: 275-276 °C. ^{1}H NMR (400 MHz, DMSO[D$_6$] + 1-2% conc. DCl) δ = 8.30 (d, J = 8.6 Hz, 2H), 8.09 (d, J = 8.6 Hz, 2H), 7.92 (s, 1H), 7.81 (d, J = 7.7 Hz, 1H), 7.57-7.46 (m, 2H), 7.42 (d, J = 7.8 Hz, 1H). ^{13}C NMR (100.6 MHz, DMSO[D$_6$] + 1-2% conc. DCl) δ = 149.4, 146.9, 146.5, 138.1, 133.7, 132.1, 130.9, 127.9, 125.9, 124.8, 124.3, 123.7, 102.0. HRMS (*m*/*z*) [M+H]$^+$ calcd for M = $C_{15}H_{10}ClN_3O_2$, 300.0540; found, 300.0543.

3-(4-Aminophenyl)-5-(3-chlorophenyl)-1*H*-pyrazole hydrochloride (sery383)

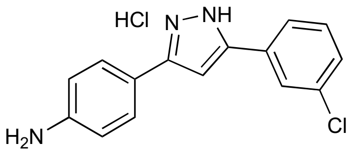

To a suspension of **sery381a** (650 mg, 2.2 mmol) in acetic acid (35 ml) iron powder (186 mg, 3.3 mmol) was added in small portions under efficient stirring at 100 °C. After addition the suspension was stirred at 100 °C for 30 min and poured in ice (200 g). A precipitate was filtered and aqueous filtrate was extracted with chloroform (40 ml × 2). A resulting crystals were

dissolved in methanol (40 ml), solutions in chloroform and methanol were combined, filtered and concentrated under reduced pressure. A crude product was dissolved in THF (25 ml) and conc. hydrochloric acid (0.3 ml) was added. The mixture was cooled for 1 h at 4 °C and a white precipitate was collected to provide **sery383** (500 mg) as a white powder. Yield 75%. m.p.: 265-268 °C (dec). ^{1}H NMR (400 MHz, DMSO[D$_6$]) δ = 10.14 (bs, 4H), 7.99-7.90 (m, 3H), 7.82 (d, J = 7.8 Hz, 1H), 7.53 (m, 3H), 7.39 (d, J = 7.8 Hz, 1H), 7.33 (s, 1H). ^{13}C NMR (100.6 MHz, DMSO[D$_6$]) δ = 146.5, 146.1, 133.7, 133.3, 131.6, 130.9, 130.8, 127.7, 126.3, 124.8, 123.77, 123.75, 100.7. HRMS (m/z) [M+H]$^+$ calcd for M = $C_{15}H_{12}ClN_3$, 270.0798; found, 270.0802.

(*E*)-3-(3-Bromophenyl)-1-(4-nitrophenyl)-2-propene-1-one (sery379b)

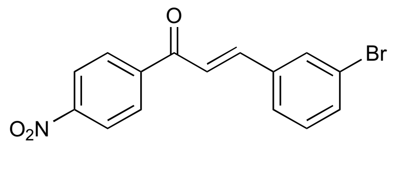

The title compound has been reported previously[35] (CAS 196082-55-0). It was prepared according to the GEP 5 using 1-(4-nitrophenyl)ethanone, 3-bromobenzaldehyde and purified by recrystallisation from EtOAc/ethanol to provide **sery379b** as a yellow solid. Yield 80%. m.p.: 152-153 °C (lit[35] 130 °C). ^{1}H NMR (400 MHz, DMSO[D$_6$]) δ = 8.38 (s, 4H), 8.24 (s, 1H), 8.04 (d, J = 15.7 Hz, 1H), 7.89 (d, J = 7.9 Hz, 1H), 7.77 (d, J = 15.7 Hz, 1H), 7.66 (d, J = 7.9 Hz, 1H), 7.43 (t, J = 7.9 Hz, 1H).

3-(3-Bromophenyl)-2,3-dibromo-1-(4-nitrophenyl)propane-1-one (sery380b)

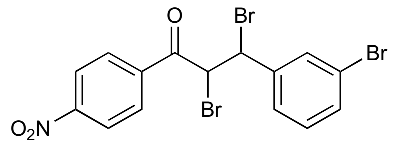

The title compound has been reported previously[35] (CAS 577970-80-0). It was prepared according to the GEP 6 starting from **sery379b** to provide **sery380b** as a white solid. Yield 85%. m.p.: 123-124 °C (lit[35] 180 °C). ^{1}H NMR (400 MHz, DMSO[D$_6$]) δ = 8.53 (d, J = 8.8 Hz, 2H), 8.45 (d, J = 8.8 Hz, 2H), 8.18 (s, 1H), 7.84 (d, J = 7.8 Hz, 1H), 7.59 (d, J = 7.9 Hz, 1H), 7.41 (t, J = 7.9 Hz, 1H), 6.84 (d, J = 11.2 Hz, 1H), 5.85 (d, J = 11.2 Hz, 1H).

3-(3-Bromophenyl)-5-(4-nitrophenyl)-1*H*-pyrazole (sery381b)

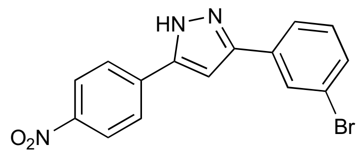

The title compound was prepared according to the GEP 7 starting from **sery380b** and purified by recrystallisation from methanol to provide **sery381b** as a yellow solid. Yield 27%. m.p.: 272 °C. ^{1}H NMR (400 MHz, DMSO[D$_6$] + 1-2% conc. DCl) δ = 8.30 (d, J = 8.7 Hz, 2H), 8.09 (d, J = 8.7 Hz, 2H), 8.05 (s, 1H), 7.84 (d, J = 7.7 Hz, 1H), 7.56 (d, J = 7.8 Hz, 1H), 7.49 (s, 1H), 7.43 (t, J = 7.8 Hz, 1H). ^{13}C NMR (100.6 MHz, DMSO[D$_6$] + 1-2% conc. DCl) δ = 146.8, 146.6, 145.4, 138.2, 132.7, 131.1, 130.8, 127.7, 125.9, 124.3, 124.1, 122.4, 102.0. HRMS (m/z) [M+H]$^+$ calcd for M = $C_{15}H_{10}{}^{79}BrN_3O_2$, 344.0035; found, 344.0039.

3-(4-Aminophenyl)-5-(3-bromophenyl)-1*H*-pyrazole hydrochloride (sery384)

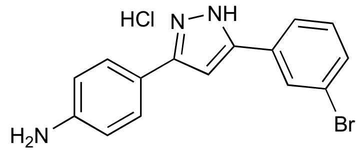

The title compound was prepared using an analogous procedure to that described for **sery383** starting from **sery381b** to provide **sery384** as a white solid. Yield 70%. m.p.: 267-272 °C (dec). ^{1}H NMR (400 MHz, DMSO[D$_6$]) δ = 8.05 (s, 1H), 7.89 (d, J = 8.5 Hz, 2H), 7.85 (d, J = 7.8 Hz, 1H), 7.54 (d, J = 7.8 Hz, 1H), 7.42 (t, J = 7.8 Hz, 1H), 7.37 (d, J = 8.5 Hz, 2H), 7.28 (s, 1H). ^{13}C NMR (100.6 MHz, DMSO[D$_6$]) δ = 146.5, 146.0, 133.5, 131.5, 131.1, 131.0, 130.7, 127.7, 126.4, 124.2, 124.0, 122.4, 100.8. HRMS (m/z) [M+H]$^+$ calcd for M = $C_{15}H_{12}{}^{79}BrN_3$, 314.0293; found, 314.0297.

(*E*)-1-(1,3-Benzodioxol-5-yl)-3-(3-nitrophenyl)-2-propene-1-one (sery302a)

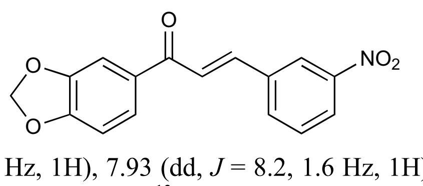

The title compound was prepared according to the GEP 5 using 1-(1,3-benzodioxol-5-yl)ethanone, 3-nitrobenzaldehyde and purified by refluxing in ethanol to provide **sery302a** as a yellow solid. Yield 97%. m.p.: 173-177 °C. ^{1}H NMR (400 MHz, DMSO[D$_6$]) δ = 8.79 (s, 1H), 8.33 (d, J = 7.8 Hz, 1H), 8.26 (dd, J = 8.2, 1.6 Hz, 1H), 8.15 (d, J = 15.6 Hz, 1H), 7.93 (dd, J = 8.2, 1.6 Hz, 1H), 7.81 (d, J = 15.6 Hz, 1H), 7.77-7.70 (m, 2H), 7.11 (d, J = 8.2 Hz, 1H), 6.18 (s, 2H). ^{13}C NMR (100.6 MHz, DMSO[D$_6$]) δ = 186.8, 151.8, 148.4, 148.1, 140.9, 136.7, 135.1, 131.9, 130.3, 125.5, 124.6, 124.5, 122.9, 108.2, 108.0, 102.2. HRMS (m/z) [M+H]$^+$ calcd for M = $C_{16}H_{11}N_3O_5$, 298.0715; found, 298.0715.

***trans*-1-(1,3-Benzodioxol-5-yl)-2,3-epoxy-3-(3-nitrophenyl)propane-1-one (sery302b)**

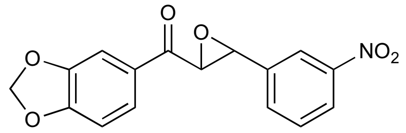

The title compound was prepared according to the published protocol[13] starting from **sery302a** and purified by recrystallisation from ethanol/EtOAc to provide **sery302b** as a white solid. Yield 70%. m.p.: 132-133 °C. ^{1}H NMR (400 MHz, DMSO[D$_6$]) δ= 8.28 (s, 1H), 8.24 (d, J = 8.2 Hz, 1H), 7.90 (d, J = 7.7 Hz, 1H), 7.78-7.68 (m, 2H), 7.53 (d, J = 1.6 Hz, 1H), 7.10 (d, J = 8.2 Hz, 1H), 6.17 (s, 1H), 6.16 (s, 1H), 4.91 (d, J = 1.8 Hz, 1H), 4.34 (d, J = 1.8 Hz, 1H). ^{13}C NMR (100.6 MHz, DMSO[D$_6$]) δ= 190.4, 152.4, 148.1, 148.0, 138.3, 133.1, 130.1, 129.9, 125.5, 123.6, 121.2, 108.4, 107.4, 102.3, 59.2, 57.3. HRMS (*m*/*z*) [M+H]$^+$ calcd for M = $C_{16}H_{11}NO_6$, 314.0665; found, 314.0664.

3-(1,3-Benzodioxol-5-yl)-5-(3-nitrophenyl)-1*H*-pyrazole (sery302c)

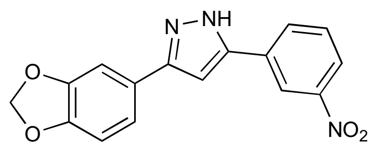

The title compound was prepared by a modified method[14]. A mixture of **sery302b** (313 mg, 1 mmol), hydrazine hydrate (150 mg, 3 mmol) and *p*-toluenesulfonic acid monohydrate (20 mg) in toluene (5 ml) was heated under reflux 4 h with stirring. A reaction mixture was evaporated under reduced pressure and a resulting solid was purified by recrystallisation from ethanol to provide **sery302c** as a yellow solid. Yield 72%. m.p.: 226-227 °C (dec). ^{1}H NMR (400 MHz, DMSO[D$_6$] + 1-2% conc. DCl) δ= 8.63 (s, 1H), 8.26 (d, J = 7.6 Hz, 1H), 8.16 (d, J = 7.6 Hz, 1H), 7.73 (t, J = 7.9 Hz, 1H), 7.40 (s, 1H), 7.38-7.26 (m, 2H), 7.02 (d, J = 7.9 Hz, 1H), 6.07 (s, 2H). ^{13}C NMR (100.6 MHz, DMSO[D$_6$]) δ= 149.1, 148.3, 147.9, 147.3, 143.8, 135.4, 131.2, 130.3, 123.1, 122.0, 119.2, 119.1, 108.8, 105.6, 101.4, 99.9. HRMS (*m*/*z*) [M+H]$^+$ calcd for M = $C_{16}H_{11}N_3O_4$, 310.0828; found, 310.0831.

3-(3-Aminophenyl)-5-(1,3-benzodioxol-5-yl)-1*H*-pyrazole (sery308)

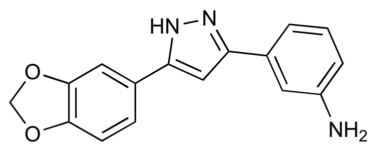

The title compound was prepared according to the GEP 8 starting from **sery302c** and was purified by column chromatography ($CHCl_3$:MeOH, 20:1 v/v) to provide **sery308** as a grey solid. Yield 31%. TLC ($CHCl_3$:MeOH, 10:1 v/v): RF = 0.47. m.p.: 200 °C. ^{1}H NMR (400 MHz, DMSO[D$_6$]) δ = 7.39 (s, 1H), 7.34 (d, J = 8.1 Hz, 1H), 7.07 (t, J = 7.5 Hz, 1H), 7.03-6.91 (m, 3H), 6.89 (s, 1H), 6.54 (d, J = 7.5 Hz, 1H), 6.05 (s, 2H), 5.16 (s, 2H). ^{13}C NMR (100.6 MHz, DMSO[D$_6$]) δ= 151.3, 148.9, 147.7, 146.8, 144.2, 130.0, 129.2, 124.1, 118.8, 113.7, 113.1, 110.6, 108.6, 105.6, 101.1, 98.9. HRMS (*m*/*z*) [M+H]$^+$ calcd for M = $C_{16}H_{13}N_3O_2$, 280.1086; found, 280.1086.

(*E*)-1-(1,3-Benzodioxol-5-yl)-3-(2,3-difluorophenyl)-2-propene-1-one (sery318a)

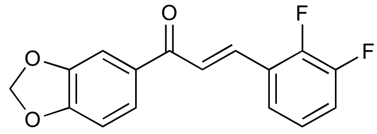

The title compound was prepared according to the GEP 5 using 1-(1,3-benzodioxol-5-yl)ethanone, 2,3-difluorobenzaldehyde and purified by recrystallisation from methanol to provide **sery318a** as a yellow solid. Yield 87%. m.p.: 121-122 °C. ^{1}H NMR (400 MHz, DMSO[D$_6$]) δ = 8.05-7.91 (m, 2H), 7.86 (dd, J = 8.2, 1.5 Hz, 1H), 7.76 (d, J = 15.7 Hz, 1H), 7.64 (d, J = 1.5 Hz, 1H), 7.56-7.26 (m. 2H), 7.10 (d, J = 8.2 Hz, 1H), 6.17 (s, 2H). ^{13}C NMR (100.6 MHz, DMSO[D$_6$]) δ= 186.6, 151.9, 150.0 (dd, J = 245.2, 12.7 Hz), 148.5 (dd, J = 252.2, 13.0 Hz), 148.1, 133.1 (t, J = 3.6 Hz), 131.8, 125.4, 125.3 (d, J = 3.9 Hz), 125.0 (dd, J = 7.0, 4.8 Hz), 124.7 (d, J = 2.9 Hz), 124.1, 119.0 (d, J = 17.0 Hz), 108.2, 107.9, 102.2. HRMS (*m*/*z*) [M+H]$^+$ calcd for M = $C_{16}H_{10}F_2O_3$, 289.0676; found, 289.0677.

***trans*-1-(1,3-Benzodioxol-5-yl)-3-(2,3-difluorophenyl)-2,3-epoxypropane-1-one (sery318b)**

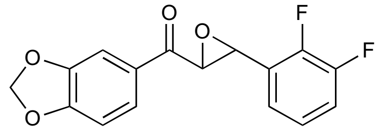

The title compound was prepared according to the published protocol[13] starting from **sery318a** to provide **sery318b** as a white solid. Yield 82%. m.p.: 92-93 °C. ^{1}H NMR (400 MHz, DMSO[D$_6$]) δ= 7.78 (dd, J = 8.2, 1.7 Hz, 1H), 7.54 (d, J = 1.7 Hz, 1H), 7.51-7.42 (m, 1H), 7.32-7.18 (m, 2H), 7.10 (d, J = 8.2 Hz, 1H), 6.17 (s, 2H), 4.84 (d, J = 1.9 Hz, 1H), 4.28 (d, J = 1.8 Hz, 1H). ^{13}C NMR (100.6 MHz, DMSO[D$_6$]) δ= 190.4, 152.5, 149.5 (dd, J = 246.1, 12.2 Hz), 148.8 (dd, J = 248.1, 13.8 Hz), 148.1, 129.8, 125.6, 125.5 (dd, J = 6.9, 4.3 Hz), 125.1, 122.4, 117.6 (d, J = 16.9 Hz), 108.4, 107.4, 102.3, 57.8, 52.9 (t, J = 4.6 Hz). HRMS (*m*/*z*) [M+Na]$^+$ calcd for M = $C_{16}H_{10}F_2O_4$, 327.0045; found, 327.0447.

3-(1,3-Benzodioxol-5-yl)-5-(2,3-difluorophenyl)-1*H*-pyrazole (sery319)

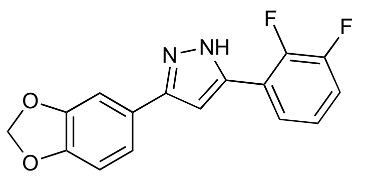

The title compound was prepared using an analogous procedure to that described for **sery302c** starting from **sery318b** and purified by recrystallisation from methanol to provide **sery319** as a brown solid. Yield 50%. m.p.: 211-212 °C (dec). ^{1}H NMR (400 MHz, DMSO[D$_6$] + 1-2% conc. DCl) δ = 13.47 (bs, 1H), 7.74 (t, *J* = 7.4 Hz, 1H), 7.44-7.23 (m, 4H), 7.03 (d, *J* = 3.0 Hz, 1H), 6.99 (d, *J* = 8.1 Hz, 1H), 6.07 (s, 2H). ^{13}C NMR (100.6 MHz, DMSO[D$_6$] + 1-2% conc. DCl) δ = 150.4 (d, *J* = 244.6 Hz), 150.2 (d, *J* = 244.9 Hz), 147.8, 147.2, 145.7, 142.3, 124.9, 124.4, 123.1, 122.2, 119.1, 116.3 (dd, *J* = 15.2, 3.6 Hz), 108.7, 105.7, 102.2 (d, *J* = 7.7 Hz), 101.2. HRMS (*m*/*z*) [M+H]$^+$ calcd for M = $C_{16}H_{10}F_2N0_2O_2$, 301.0789; found, 301.0792.

2-Acetylphenyl-4-chlorobenzoate (sery281b)

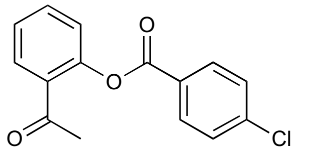

The title compound has been reported previously[36] (CAS 84634-62-8). It was prepared according to the published protocol[36] starting from 4-chlorobenzoyl chloride and 1-(2-hydroxyphenyl)ethanone to provide **sery281b** as a white solid. Yield 75%. m.p.: 93-94 °C (lit[36] 94 °C). ^{1}H NMR (400 MHz, DMSO[D$_6$]) δ = 8.12 (d, *J* = 8.7 Hz, 2H), 7.99 (dd, *J* = 7.7, 1.6 Hz, 1H), 7.73-7.66 (m, 3H), 7.48 (td, *J* = 7.7, 1.1 Hz, 1H), 7.39 (dd, *J* = 8.1, 1.1 Hz, 1H), 2.51 (s, 3H).

1-(4-Chlorophenyl)-3-(2-hydroxyphenyl)propane-1,3-dione (sery282)

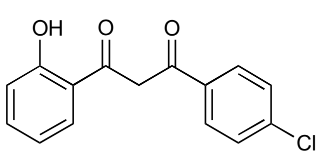

The title compound has been reported previously[36] (CAS 84634-62-8). It was prepared according to the published protocol[37] starting from **sery281b** and purified by recrystallisation from methanol to provide **sery282** as a yellow solid. Yield 42%. m.p.: 123-124 °C (lit[36] 120 °C). ^{1}H NMR (400 MHz, $CDCl_3$) δ = 12.01 (s, 1H), 7.88 (d, *J* = 8.6 Hz, 2H), 7.77 (dd, *J* = 8.1, 1.4 Hz, 1H), 7.51-7.44 (m, 3H), 7.01 (d, *J* = 8.6 Hz, 1H), 6.93 (t, *J* = 7.4 Hz, 1H), 6.81 (s, 1H).

3-(4-Chlorophenyl)-5-(2-hydroxyphenyl)-1*H*-pyrazole (sery283)

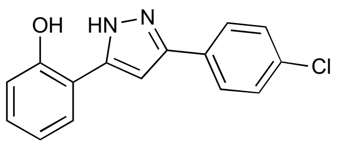

The title compound has been reported previously[38] (CAS 38371-80-1). It was prepared according to the GEP 2 starting from **sery282** and purified by recrystallisation from petroleum benzine (40-60 °C)/methanol to provide **sery283** as a white solid. Yield 46%. m.p.: 192-194 °C (lit[38] 191 °C). ^{1}H NMR (400 MHz, DMSO[D$_6$] + 1-2% conc. DCl) δ = 10.58 (bs, 1H), 7.86 (d, *J* = 8.6 Hz, 2H), 7.72 (dd, *J* = 7.7, 1.4 Hz, 1H), 7.52 (d, *J* = 8.6 Hz, 2H), 7.26 (s, 1H), 7.22-7.15 (m, 1H), 6.97 (d, *J* = 8.2 Hz, 1H), 6.90 (td, *J* = 7.7, 1.0 Hz, 1H).

2-Acetylphenyl 3,4-dimethoxybenzoate (sery288b)

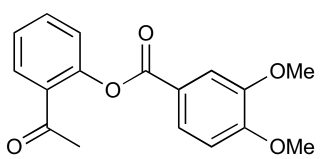

The title compound has been reported previously[36] (CAS 67858-33-7). It was prepared according to the published protocol[36] starting from 3,4-dimethoxybenzoyl chloride and 1-(2-hydroxyphenyl)ethanone and purified by recrystallisation from hexane/EtOAc to provide **sery288b** as a white solid. Yield 74%. m.p.: 125-127 °C (lit[36] 126-127 °C). ^{1}H NMR (400 MHz, DMSO[D$_6$]) δ = 7.93 (dd, *J* = 7.8, 1.6 Hz, 1H), 7.78 (dd, *J* = 8.4, 2.0 Hz, 1H), 7.67 (td, *J* = 7.8, 1.6 Hz, 1H), 7.58 (d, *J* = 2.0 Hz, 1H), 7.45 (td, *J* = 7.6, 1.0 Hz, 1H), 7.34 (dd, *J* = 8.1, 1.0 Hz, 1H), 7.16 (d, *J* = 8.4 Hz, 1H), 3.88 (s, 3H), 3.84 (s, 3H), 2.50 (s, 3H).

1-(3,4-Dimethoxyphenyl)-3-(2-hydroxyphenyl)propane-1,3-dione (sery290a)

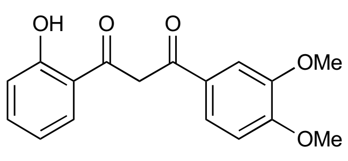

The title compound has been reported previously[36] (CAS 4233-35-6). It was prepared according to the published protocol[37] starting from **sery288b** and purified by recrystallisation from methanol to provide **sery290a** as a yellow solid. Yield 73%. m.p.: 129-130 °C (lit[36] 115 °C). ^{1}H NMR (400 MHz, $CDCl_3$) δ = 12.09 (s, 1H), 7.75 (dd, *J* = 8.1, 1.4 Hz, 1H), 7.57 (dd, *J* = 8.4, 2.0 Hz, 1H), 7.48-7.41 (m, 2H), 6.99 (dd, *J* = 8.4, 0.9 Hz, 1H), 6.95-6.87 (m, 2H), 6.75 (s, 1H), 3.97 (s, 3H), 3.95 (s, 3H).

3-(3,4-Dimethoxyphenyl)-5-(2-hydroxyphenyl)-1*H*-pyrazole (sery290b)

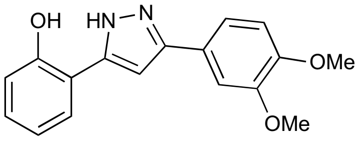

The title compound has been reported previously[39] (CAS 38371-74-3). It was prepared according to the GEP 2 starting from **sery290a** and purified by recrystallisation from methanol to provide **sery290b** as a white solid. Yield 86%. m.p.: 216-217 °C (lit[39] 214 °C). ^{1}H NMR (400 MHz, DMSO[D_6] + 1-2% conc. DCl) δ = 10.84 (bs, 1H), 7.75 (d, J = 7.3 Hz, 1H), 7.44 (s, 1H), 7.39 (d, J = 8.3 Hz, 1H), 7.23 (s, 1H), 7.19 (t, J = 8.1 Hz, 1H), 7.05 (d, J = 8.3 Hz, 1H), 6.99-6.87 (m, 2H), 3.85 (s, 3H), 3.80 (s, 3H).

1-Acetyl-3,5-bis(3,4-dimethoxyphenyl)-1*H*-pyrazole (sery153)

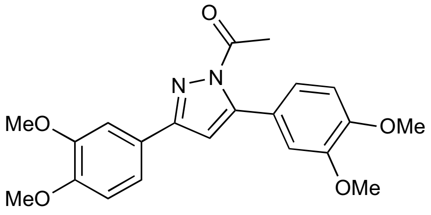

The title compound was prepared according to the published protocol[40]. A mixture of **sery149** (340 mg, 1 mmol), TEA (152 mg, 1.5 mmol) and acetyl chloride (118 mg, 1.5 mmol) in benzene (5 ml) was heated under reflux 5 h with stirring. The reaction mixture was cooled down, quenched with water (15 ml) and benzene (10 ml). The organic layer was washed with 0.1M HCl (10 ml), 0.1 NaOH (10 ml), brine (15 ml), dried (Na_2SO_4) and concentrated. The crude product was purified by column chromatography (hexane:EtOAc, 1:1 v/v) followed by recrystallisation from hexane to provide **sery153** (105 mg) as a white solid. Yield 28%. m.p.: 113-115 °C. ^{1}H NMR (400 MHz, DMSO[D_6]) δ = 7.55-7.48 (m, 2H), 7.13 (d, J = 1.9 Hz, 1H), 7.11 (s, 1H), 7.08-7.03 (m, 2H), 6.99 (d, J = 8.4 Hz, 1H), 3.85 (s, 3H), 3.81 (s, 3H), 3.80 (s, 3H), 3.76 (s, 3H), 2.73 (s, 3H). ^{13}C NMR (100.6 MHz, DMSO[D_6]) δ = 170.0, 152.5, 149.8, 149.2, 149.0, 147.9, 146.5, 124.1, 123.3, 121.6, 118.9, 113.1, 111.9, 110.9, 109.7, 109.4, 55.7, 55.6, 55.5 (2C), 23.7. HRMS (m/z) [M+H]$^+$ calcd for M = $C_{21}H_{22}N_2O_5$, 383.1607; found, 383.1608.

5-(1,3-Benzodioxol-5-yl)-3-(3-bromophenyl)-1-(2-fluoroethyl)-1*H*-pyrazole (sery363a) and 3-(1,3-benzodioxol-5-yl)-5-(3-bromophenyl)-1-(2-fluoroethyl)-1*H*-pyrazole (sery363b)

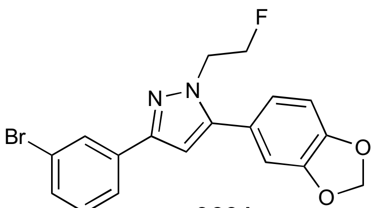

sery363A

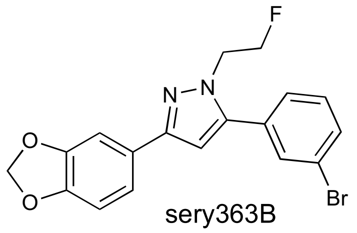

sery363B

A mixture of **anle138b** (1.5 g, 4.4 mmol), 1N NaOH (4.4 ml) and DMF (40 ml) was heated for 10 min at 100 °C and a solution of 2-fluoroethyl ester of 4-methylbenzenesulfonic acid (1.05 g, 4.8 mmol) in DMF (5 ml) was added in one portion. After stirring for 5 min at 100 °C the reaction mixture was cooled down, the solvent was evaporated under reduced pressure. A remaining oil was diluted with water (50 ml), extracted with EtOAc (3 × 50 ml), combined organic fractions were washed with brine (20 ml), dried (Na_2SO_4) and evaporated. A resulting oil was purified by column chromatography (hexane:EtOAc, 8:1 v/v) on fine silica gel (5-40 mesh) to provide **sery363a** (775 mg, 46%) as a white solid. The second product was purified by additional column chromatography (gradient hexane:CH_2Cl_2, 3:1 v/v to hexane:CH_2Cl_2, 1:4 v/v) to provide **sery363b** (400 mg, 24%) as a white solid.

sery363a: TLC (hexane:EtOAc, 5:1 v/v): RF = 0.40. m.p.: 94-95 °C. ^{1}H NMR (400 MHz, DMSO[D_6]) δ = 8.02 (t, J = 1.7 Hz, 1H), 7.84 (d, J = 7.9 Hz, 1H), 7.51 (d, J = 7.9 Hz, 1H), 7.39 (t, J = 7.9 Hz, 1H), 7.11 (d, J = 1.6 Hz, 1H), 7.07 (d, J = 8.0 Hz, 1H), 7.00 (dd, J = 8.0, 1.7 Hz, 1H), 6.90 (s, 1H), 6.10 (s, 2H), 4.84 (dt, J = 47.0, 4.7 Hz, 2H), 4.40 (dt, J = 26.7, 4.7 Hz, 2H). ^{13}C NMR (100.6 MHz, DMSO[D_6]) δ = 148.2, 147.8, 147.6, 145.4, 135.4, 130.9, 130.3, 127.5, 124.1, 123.3, 122.9, 122.1, 109.2, 108.6, 103.8, 101.5, 82.0 (d, J = 167.4 Hz), 49.5 (d, J = 20.4 Hz). HRMS (m/z) [M+H]$^+$ calcd for M = $C_{18}H_{14}{}^{79}BrFN_2O_2$, 389.0301; found, 389.0304.

sery363b: TLC (hexane:EtOAc, 5:1 v/v): RF = 0.36. m.p.: 98-99 °C. ^{1}H NMR (400 MHz, DMSO[D_6]) δ = 7.74 (t, J = 1.6 Hz, 1H), 7.69 (d, J = 7.9 Hz, 1H), 7.54 (d, J = 7.9 Hz, 1H), 7.49 (t, J = 7.9 Hz, 1H), 7.4-7.34 (m, 2H), 6.97 (d, J = 7.9 Hz, 1H), 6.88 (s, 1H), 6.05 (s, 2H), 4.83 (dt, J = 47.2, 4.6 Hz, 2H), 4.40 (dt, J = 26.7, 4.6 Hz, 2H). ^{13}C NMR (100.6 MHz, DMSO[D_6]) δ = 149.8, 147.65, 146.9, 143.8, 132.2, 131.5, 131.3, 130.9, 127.8, 127.2, 122.0, 118.8, 108.5, 105.5, 103.8, 101.0, 82.2 (d, J = 169 Hz), 49.6 (d, J = 20 Hz). HRMS (m/z) [M+H]$^+$ calcd for M = $C_{18}H_{14}{}^{79}BrFN_2O_2$, 389.0301; found, 389.0303. A discrimination between two isomers was done based on NOESY spectrum.

5-(1,3-Benzodioxol-5-yl)-3-(3-bromophenyl)-1-methyl-1*H*-pyrazole (sery392a) and 3-(1,3-benzodioxol-5-yl)-5-(3-bromophenyl)-1-methyl-1*H*-pyrazole (sery392b)

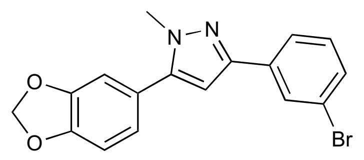

sery392A

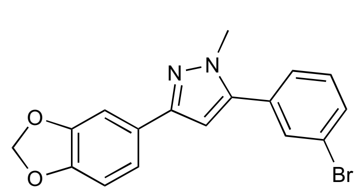

sery392B

To a solution of **anle138b** (2.0 g, 5.8 mmol) in anhydrous DMF (20 ml) 60 % suspension of sodium hydride in mineral oil (236 mg, 5.9 mmol) was added in small portions, a resulting mixture was stirred for 1 h at room temperature under argon atmosphere and treated with iodomethane (1.66 g, 11.7 mmol). After stirring for overnight at room temperature solvent was evaporated under reduced pressure and a remaining oil was diluted with water (50 ml), extracted with EtOAc (3 × 50 ml), combined organic fractions were washed with brine (20 ml), dried (Na_2SO_4) and evaporated. A resulting mixture was purified by column chromatography (hexane:EtOAc, 10:1 v/v) on fine silica gel (5-40 mesh) followed by recrystallisation from ethanol to provide two products: **sery392a** (1.02 g, 49%) as a white solid and **sery392b** (625 mg, 30%) as a white solid.

sery392a: TLC (hexane:EtOAc, 9:1 v/v): RF = 0.14. m.p.: 96-97 °C. ^{1}H NMR (400 MHz, DMSO[D_6]) δ= 7.99 (t, J = 1.6 Hz, 1H), 7.82 (d, J = 7.8 Hz, 1H), 7.49 (ddd, J = 7.9, 1.6, 0.8 Hz, 1H), 7.37 (t, J = 7.8 Hz, 1H), 7.17 (s, 1H), 7.05 (s, 2H), 6.90 (s, 1H), 6.11 (s, 2H), 3.87 (s, 3H). ^{13}C NMR (100.6 MHz, DMSO[D_6]) δ= 147.6, 147.6, 147.2, 144.4, 135.6, 130.9, 130.0, 127.4, 123.9, 123.5, 122.5, 122.1, 108.9, 108.6, 103.3, 101.4, 37.7. HRMS (*m/z*) $[M+H]^+$ calcd for M = $C_{17}H_{13}{}^{79}BrN_2O_2$, 357.0239; found, 357.0237.

sery392b: TLC (hexane:EtOAc, 9:1 v/v): RF = 0.17. m.p.: 114-115 °C. ^{1}H NMR (400 MHz, DMSO[D_6]) δ= 7.79 (t, J = 1.6 Hz, 1H), 7.68-7.57 (m, 2H), 7.48 (t, J = 7.9 Hz, 1H), 7.39-7.30 (m, 2H), 6.95 (d, J = 8.0 Hz, 1H), 6.89 (s, 1H), 6.04 (s, 2H), 3.88 (s, 3H). ^{13}C NMR (100.6 MHz, DMSO[D_6]) δ= 148.9, 147.6, 146.8, 142.7, 132.3, 131.3, 130.9, 130.9, 127.4, 123.3, 122.0, 118.6, 108.5, 105.4, 103.3, 101.0, 37.3. HRMS (*m/z*) $[M+H]^+$ calcd for M = $C_{17}H_{13}{}^{79}BrN_2O_2$, 357.0239; found, 357.0241. A discrimination between two isomers was done based on NOESY spectrum.

3-(1,3-Benzodioxol-5-yl)-4-bromo-5-phenyl-1*H*-pyrazole (anle134)

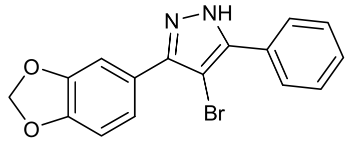

The title compound was prepared by the published protocol[41] with minor modifications. A mixture of **anle126** (264 mg, 1.00 mmol) and *N*-bromosuccinimide (180 mg, 1.01 mmol) in $CHCl_3$ (6 ml) was stirred 2 h at room temperature. The solvent was evaporated under reduced pressure, the residue was co-evaporated two times with MeOH (20 ml). The resulting precipitate was stirred 2 h with water (40 ml), the product was collected by filtration and dried to provide **anle134** (286 mg, 83%) as a white solid. m.p.: 183-185 °C. ^{1}H NMR (400 MHz, DMSO[D_6] + 1-2% conc. DCl) δ= 7.77 (d, J = 7.2 Hz, 2H), 7.50 (t, J = 7.2 Hz, 2H), 7.43 (t, J = 7.2 Hz, 1H), 7.33-7.23 (m, 2H), 7.05 (d, J = 8.6 Hz, 1H), 6.08 (s, 2H). ^{13}C NMR (100.6 MHz, DMSO[D_6] + 1-2% conc. DCl) δ= 147.8, 147.7, 145.1 (2C), 130.7, 128.9, 128.8, 127.8, 124.3, 121.9, 108.8, 108.1, 101.7, 89.6. HRMS (*m/z*) $[M+H]^+$ calcd for M = $C_{16}H_{11}{}^{79}BrN_2O_2$, 343.0082; found, 343.0084.

3-(1,3-Benzodioxol-5-yl)-4-bromo-5-(3-bromophenyl)-1*H*-pyrazole (sery330)

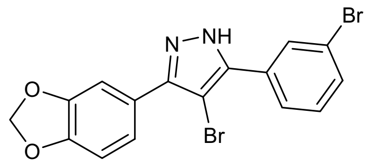

The title compound was prepared analogously to the procedure for **anle134** starting from **anle138b**. White solid. Yield 93%. m.p.: 204-205 °C. ^{1}H NMR (400 MHz, DMSO[D_6] + 1-2% conc. DCl) δ= 13.75 (bs, 1H), 7.97 (s, 1H), 7.83 (d, J = 6.8 Hz, 1H), 7.63 (d, J = 7.2 Hz, 1H), 7.46 (t, J = 7.6 Hz, 1H), 7.27 (s, 2H), 7.06 (d, J = 8.0 Hz, 1H), 6.09 (s, 2H). ^{13}C NMR (100.6 MHz, DMSO[D_6] + 1-2% conc. DCl) δ= 147.8, 147.6, 144.2 (2C), 133.3, 131.2, 130.9, 129.9, 126.5, 123.3, 121.8, 121.8, 108.6, 107.9, 101.5, 89.6. HRMS (*m/z*) $[M+H]^+$ calcd for M = $C_{16}H_{10}{}^{79}Br^{81}BrN_2O_2$, 422.9167; found, 422.9168.

2.2 Synthesis of isoxazoles

(*E*)-1-(1,3-Benzodioxol-5-yl)-3-phenyl-2-propene-1-one (sery89)

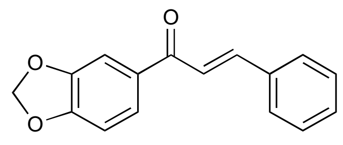

The title compound has been reported previously[42] (CAS 204450-46-4). It was prepared according to the GEP 5 using 1-(1,3-benzodioxol-5-yl)ethanone, benzaldehyde and purified by recrystallisation from methanol to provide **sery89** as a yellow solid. Yield 87%. m.p.: 99-100 °C (lit[42] 97 °C). ^{1}H NMR (400 MHz, DMSO[D_6]) δ= 7.97-7.83 (m, 4H), 7.70 (d, J = 15.6 Hz, 1H), 7.66 (d, J = 1.4 Hz, 1H), 7.50-7.40 (m, 3H), 7.08 (d, J = 8.2 Hz, 1H), 6.16 (s, 2H).

1-(1,3-Benzodioxol-5-yl)-2,3-dibromo-3-phenylpropane-1-one (sery93a)

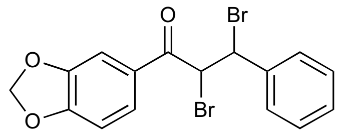

The title compound has been reported previously[43] (CAS 25856-08-0). It was prepared according to the GEP 6 starting from **sery89** to provide **sery93a** as a red solid. Yield 60%. m.p.: 159-160 °C (lit[43] 160-162 °C). ^{1}H NMR (400 MHz, DMSO[D_6]) δ = 7.99 (dd, J = 8.3, 1.4 Hz, 1H), 7.85-7.75 (m, 3H), 7.48-7.33 (m, 3H), 7.16 (d, J = 8.3 Hz, 1H), 6.62 (d, J = 11.3 Hz, 1H), 6.20 (s, 2H), 5.73 (d, J = 11.3 Hz, 1H).

General Experimental Procedure for the synthesis of 3,5-diarylisoxazoles (GEP 9)[8]

3-(1,3-Benzodioxol-5-yl)-5-phenylisoxazole (sery93b)

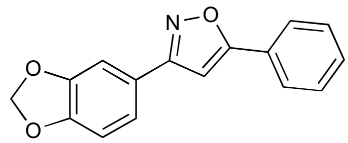

The title compound has been reported previously[43] (CAS 25856-13-7). A mixture of **sery93a** (150 mg, 0.36 mmol), hydroxylamine hydrochloride (110 mg, 1.6 mmol) and water (500 mg) in ethanol (2 ml) was heated under reflux 2 h with stirring. The reaction mixture was treated with water (0.5 ml) and cooled in ice bath for 2h. A resulting precipitate was collected by filtration and dried to provide **sery93b** as a white solid. Yield 53%. m.p.: 134-135 °C (lit[43] 130 °C). ^{1}H NMR (400 MHz, DMSO[D_6]) δ = 7.89 (d, J = 7.2 Hz, 2H), 7.63-7.40 (m, 6H), 7.08 (d, J = 8.3 Hz, 1H), 6.12 (s, 2H). ^{13}C NMR (100.6 MHz, DMSO[D_6]) δ = 169.5, 162.2, 148.9, 148.0, 130.4, 129.3, 126.9, 125.5, 122.4, 121.1, 108.9, 106.4, 101.6, 98.5.

(*E*)-3-(3,4-Dimethoxyphenyl)-1-(4-methoxyphenyl)-2-propene-1-one (sery96)

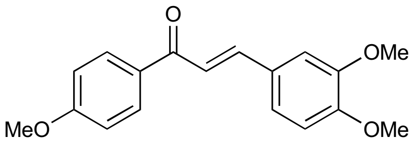

The title compound has been reported previously[44] (CAS 144309-49-9). It was prepared according to the GEP 5 using 1-(4-methoxyphenyl)ethanone, 3,4-dimethoxybenzaldehyde and purified by recrystallisation from methanol to provide **sery96** as a yellow solid. Yield 77%. m.p.: 92-93 °C (lit[44] 93 °C). ^{1}H NMR (400 MHz, DMSO[D_6]) δ = 8.16 (d, J = 8.9 Hz, 2H), 7.82 (d, J = 15.5 Hz, 1H), 7.67 (d, J = 15.5 Hz, 1H), 7.57 (d, J = 2.0 Hz, 1H), 7.37 (dd, J = 8.2, 2.0 Hz, 1H), 7.08 (d, J = 8.9 Hz, 2H), 7.01 (d, J = 8.2 Hz, 1H), 3.86 (s, 6H), 3.81 (s, 3H).

2,3-Dibromo-3-(3,4-dimethoxyphenyl)-1-(4-methoxyphenyl)propane-1-one (sery102)

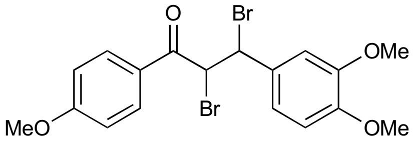

The title compound was prepared according to the GEP 6 starting from **sery96** to provide **sery102** as a white solid. Yield 79%. m.p.: 136 °C. ^{1}H NMR (400 MHz, $CDCl_3$) δ = 8.09 (d, J = 8.9 Hz, 2H), 7.11 (dd, J = 8.3, 2.0 Hz, 1H), 7.05-6.98 (m, 3H), 6.89 (d, J = 8.3 Hz, 1H), 5.76 (d, J = 11.3 Hz, 1H), 5.65 (d, J = 11.3 Hz, 1H), 3.95 (s, 3H), 3.91 (s, 3H), 3.90 (s, 3H). ^{13}C NMR (100.6 MHz, DMSO[D_6]) δ = 189.8, 164.6, 149.9, 149.2, 131.5, 130.9, 127.4, 121.3, 114.4, 111.4, 111.1, 56.2, 56.1, 55.8, 51.0, 47.2. HRMS (*m/z*) $[M\text{-}2HBr+H]^+$ calcd for M = $C_{18}H_{18}{}^{79}Br_2O_4$, 297.1127; found, 297.1122.

5-(3,4-Dimethoxyphenyl)-3-(4-methoxyphenyl)isoxazole (sery103)

The title compound was prepared according to the GEP 9 starting from **sery102** to provide **sery103** as a white solid. Yield 43%. m.p.: 115-118 °C. ^{1}H NMR (400 MHz, DMSO[D_6]) δ = 7.84 (d, J = 8.8 Hz, 2H), 7.50-7.42 (m, 3H), 7.13 (d, J = 8.4 Hz, 1H), 7.10 (d, J = 8.8 Hz, 2H), 3.87 (s, 3H), 3.83 (s, 6H). ^{13}C NMR (100.6 MHz, DMSO[D_6]) δ = 169.6, 162.1, 160.7, 150.6, 149.1, 128.0, 121.1, 119.7, 118.6, 114.5, 112.0, 108.9, 97.1, 55.7, 55.6, 55.3. HRMS (*m/z*) $[M+Na]^+$ calcd for M = $C_{18}H_{17}NO_4$, 334.1055; found, 334.1053.

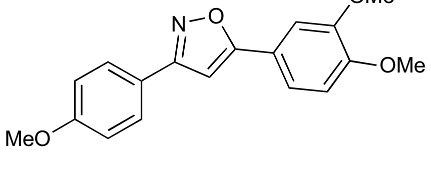

5-(3,4-Dihydroxyphenyl)-3-(4-hydroxyphenyl)isoxazole (sery118)

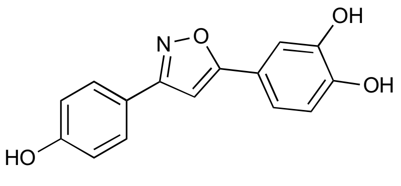

The title compound was prepared according to the GEP 3 starting from **sery103** to provide **sery118** as a white solid. Yield 68%. m.p.: >300 °C. ^{1}H NMR (400 MHz, DMSO[D$_6$]) δ = 9.89 (s, 1H), 9.58 (s, 1H), 9.35 (s, 1H), 7.72 (d, *J* = 8.6 Hz, 2H), 7.25 (d, *J* = 2.0 Hz, 1H), 7.20 (dd, *J* = 8.2, 2.0 Hz, 1H), 7.15 (s, 1H), 6.92-6.84 (m, 3H). ^{13}C NMR (100.6 MHz, DMSO[D$_6$]) δ = 169.8, 162.2, 159.1, 147.8, 145.7, 128.1, 119.6, 118.5, 117.6, 116.1, 115.8, 112.7, 96.0. HRMS (*m*/*z*) [M+H]$^+$ calcd for M = $C_{15}H_{11}NO_4$, 270.0766; found, 270.0761.

(*E*)-1-(3,4-Dimethoxyphenyl)-3-(3-fluorophenyl)-2-propene-1-one (sery97)

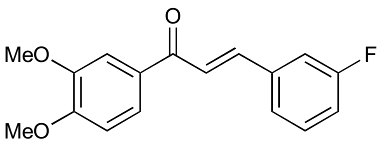

The title compound prepared according to the GEP 5 using 1-(3,4-dimethoxyphenyl)ethanone, 3-fluorobenzaldehyde and purified by recrystallisation from methanol to provide **sery97** as a white solid. Yield 58%. m.p.: 105-107 °C. ^{1}H NMR (400 MHz, DMSO[D$_6$]) δ = 8.01 (d, *J* = 15.6 Hz, 1H), 7.94 (dd, *J* = 8.4, 1.8 Hz, 1H), 7.85 (d, *J* = 10.3 Hz, 1H), 7.74-7.65 (m, 2H), 7.61 (d, *J* = 1.8 Hz, 1H), 7.53-7.45 (m, 1H), 7.27 (td, *J* = 8.5, 2.1 Hz, 1H), 7.11 (d, *J* = 8.5 Hz, 1H), 3.88 (s, 3H), 3.86 (s, 3H). ^{13}C NMR (100.6 MHz, DMSO[D$_6$]) δ = 187.2, 162.5 (d, *J* = 243.3 Hz), 153.4, 148.8, 141.6, 137.4 (d, *J* = 8.0 Hz), 130.8 (d, *J* = 8.0 Hz), 130.3, 125.5, 123.6, 123.4, 117.0 (d, *J* = 21.4 Hz), 114.5 (d, *J* = 21.8 Hz), 110.9, 110.8, 55.8, 55.6. HRMS (*m*/*z*) [M+H]$^+$ calcd for M = $C_{17}H_{15}FO_3$, 287.1083; found, 287.1083.

2,3-Dibromo-1-(3,4-dimethoxyphenyl)-3-(3-fluorophenyl)propane-1-one (sery107)

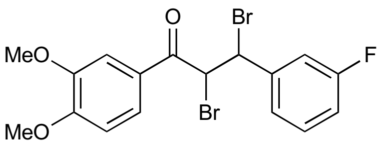

The title compound was prepared according to the GEP 6 starting from **sery97** to provide **sery107** as a white solid. Yield 70%. m.p.: 174 °C. ^{1}H NMR (400 MHz, DMSO[D$_6$]) δ = 8.09 (dd, *J* = 8.5, 2.0 Hz, 1H). 7.83 (d, *J* = 10.2 Hz, 1H), 7.70-7.60 (m, 2H), 7.53-7.43 (m, 1H), 7.22 (td, *J* = 8.5, 2.3 Hz, 1H), 7.18 (d, *J* = 8.5 Hz, 1H), 6.67 (d, *J* = 11.3 Hz, 1H), 5.81 (d, *J* = 11.3 Hz, 1H), 3.91 (s, 3H), 3.88 (s, 3H). ^{13}C NMR (100.6 MHz, DMSO[D$_6$]) δ = 189.7, 162.2 (d, *J* = 245.5 Hz), 154.4, 149.1, 141.4 (d, *J* = 7.9 Hz), 130.6 (d, *J* = 8.5 Hz), 126.4, 125.2, 124.5, 116.0 (d, *J* = 21.0 Hz), 115.6 (d, *J* = 23.0 Hz), 111.1, 111.0, 56.0, 55.7, 49.7, 45.8. HRMS (*m*/*z*) [M-2HBr+H]$^+$ calcd for M = $C_{17}H_{15}{}^{79}Br^{81}Br$ FO_3, 285.0927; found, 285.0924.

3-(3,4-Dimethoxyphenyl)-5-(3-fluorophenyl)isoxazole (sery109)

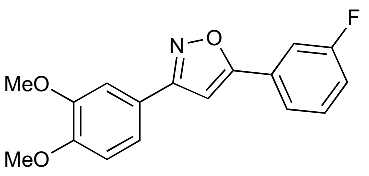

The title compound was prepared according to the GEP 9 starting from **sery107** to provide **sery109** as a white solid. Yield 60%. m.p.: 116 °C. ^{1}H NMR (400 MHz, DMSO[D$_6$]) δ = 7.80-7.71 (m, 2H), 7.70 (s, 1H), 7.66-7.58 (m, 1H), 7.50-7.42 (m, 2H), 7.37 (td, *J* = 8.6, 2.1 Hz, 1H), 7.11 (d, *J* = 8.1 Hz, 1H), 3.86 (s, 3H), 3.83 (s, 3H). ^{13}C NMR (100.6 MHz, DMSO[D$_6$]) δ = 168.0, 162.5, 162.4 (d, *J* = 244.5 Hz), 150.6, 149.1, 131.6 (d, *J* = 8.5 Hz), 129.0 (d, *J* = 8.7 Hz), 121.6, 120.8, 119.6, 117.3 (d, *J* = 20.9 Hz), 112.4 (d, *J* = 23.7 Hz), 112.0, 109.6, 99.6, 55.6 (2C). HRMS (*m*/*z*) [M+Na]$^+$ calcd for M = $C_{17}H_{14}FNO_3$, 300.1036; found, 300.1034.

3-(3,4-Dihydroxyphenyl)-5-(3-fluorophenyl)isoxazole (sery117)

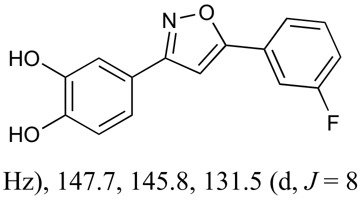

The title compound was prepared according to the GEP 3 starting from **sery109** to provide **sery117** as a white solid. Yield 65%. m.p.: 190-200 °C (dec). ^{1}H NMR (400 MHz, DMSO[D$_6$]) δ = 9.46 (s, 1H), 9.29 (s, 1H), 7.78-7.70 (m, 2H), 7.65-7.56 (m, 1H), 7.52 (s, 1H), 7.31 (td, *J* = 8.6, 2.5 Hz, 1H), 7.32 (d, *J* = 2.0 Hz, 1H), 7.18 (dd, *J* = 8.2, 2.0 Hz, 1H), 6.87 (d, *J* = 8.2 Hz, 1H). ^{13}C NMR (100.6 MHz, DMSO[D$_6$]) δ = 167.8, 162.7, 162.4 (d, *J* = 244.4 Hz), 147.7, 145.8, 131.5 (d, *J* = 8.5 Hz), 129.0 (d, *J* = 8.6 Hz), 121.7, 119.4, 118.6, 117.2 (d, *J* = 20.5 Hz), 115.9, 113.5, 112.4 (d, *J* = 21.2 Hz), 99.4. HRMS (*m*/*z*) [M+H]$^+$ calcd for M = $C_{15}H_{10}FNO_3$, 272.0723; found, 272.0719.

3,5-Bis(3,4-Dimethoxyphenyl)isoxazole (sery137)

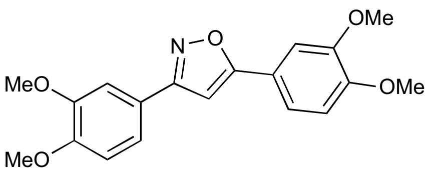

The title compound has been reported previously[45] (CAS 52789-39-6). A mixture of **sery149** (772 mg, 2.25 mmol), hydroxylamine hydrochloride (165 mg, 2.36 mmol) and pyridine (2 ml) in ethanol (8 ml) was heated under reflux 2 h with stirring. The reaction mixture

was evaporated under reduced pressure, treated with water (20 ml). A resulting precipitate was collected by filtration and purified by recrystallisation from ethanol to provide **sery137** as a white solid. Yield 89%. m.p.: 134 °C. ^{1}H NMR (400 MHz, DMSO[D$_6$]) δ = 7.50-7.41 (m, 5H), 7.15-7.07 (m, 2H), 3.88 (s, 3H), 3.86 (s, 3H), 3.85-3.80 (m, 6H). ^{13}C NMR (100.6 MHz, DMSO[D$_6$]) δ = 169.6, 162.3, 150.6, 150.4, 149.1, 149.0, 121.2, 119.7, 119.5, 118.6, 112.0, 111.9, 109.6, 108.9, 97.3, 55.6 (4C).

3,5-Bis(3,4-Dihydroxyphenyl)isoxazole hydrobromide (sery158b)

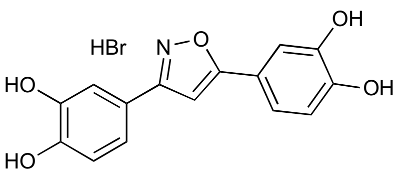

The title compound was prepared according to the GEP 3 starting from **sery137** to provide **sery158b** as a grey solid. Yield 93%. m.p.: 277 °C (dec). ^{1}H NMR (400 MHz, DMSO[D$_6$]) δ = 7.29 (d, J = 2.1 Hz, 1H), 7.24 (d, J = 2.1 Hz, 1H), 7.22-7.14 (m, 2H), 7.09 (s, 1H), 6.87 (d, J = 8.2 Hz, 1H), 6.84 (d, J = 8.2 Hz, 1H). ^{13}C NMR (100.6 MHz, DMSO[D$_6$]) δ = 169.7, 162.4, 147.7, 147.4, 145.7, 145.6, 120.0, 118.6, 118.5, 117.6, 116.1, 115.8, 113.5, 112.8, 96.1. HRMS (*m*/*z*) [M+H]$^+$ calcd for M = $C_{15}H_{11}NO_5$, 286.0715; found, 286.0716.

(*E*)-1-(1,3-Benzodioxol-5-yl)-3-(3-bromophenyl)-2-propene-1-one (sery298a)

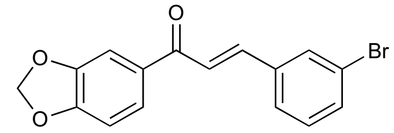

The title compound was prepared according to the GEP 5 using 1-(1,3-benzodioxol-5-yl)ethanone, 3-bromobenzaldehyde and purified by washing on filter with methanol to provide **sery298a** as a white solid. Yield 91%. m.p.: 121-122 °C. ^{1}H NMR (400 MHz, DMSO[D$_6$]) δ = 8.21 (t, J = 1.6 Hz, 1H), 8.00 (d, J = 15.6 Hz, 1H), 7.90 (dd, J = 8.2, 1.8 Hz, 1H), 7.84 (d, J = 7.8 Hz, 1H), 7.70-7.60 (m, 3H), 7.40 (d, J = 7.8 Hz, 1H), 7.09 (d, J = 8.2 Hz, 1H), 6.17 (s, 2H). ^{13}C NMR (100.6 MHz, DMSO[D$_6$]) δ = 186.8, 151.7, 148.0, 141.6, 137.3, 132.9, 132.0, 130.9, 130.7, 128.3, 125.3, 123.4, 122.4, 108.2, 108.0, 102.1. HRMS (*m*/*z*) [M+H]$^+$ calcd for M = $C_{16}H_{11}{}^{79}BrO_3$, 330.9970; found, 330.9972.

1-(1,3-Benzodioxol-5-yl)-3-(3-bromophenyl)-2,3-dibromopropane-1-one (sery338a)

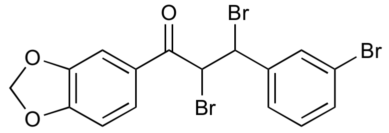

The title compound was prepared according to the GEP 6 starting from **sery298a** to provide **sery338a** as a red solid. Yield 88%. m.p.: 122 °C. ^{1}H NMR (400 MHz, CDCl$_3$) δ = 7.71 (dd, J = 8.2, 1.8 Hz, 1H), 7.68 (d, J = 1.8 Hz, 1H), 7.56 (d, J = 1.8 Hz, 1H), 7.51 (ddd, J = 7.9, 1.8, 1.1 Hz, 1H), 7.44 (dt, J = 7.9, 1.2 Hz, 1H), 7.31 (t, J = 7.9 Hz, 1H), 6.94 (d, J = 8.2 Hz, 1H), 6.11 (s, 2H), 5.68 (d, J = 11.3 Hz, 1H), 5.56 (d, J = 11.3 Hz, 1H). ^{13}C NMR (100.6 MHz, CDCl$_3$) δ = 189.1, 153.1, 148.8, 140.7, 132.5, 131.5, 130.5, 129.0, 127.3, 125.6, 122.9, 108.8, 108.4, 102.4, 48.8, 46.6. HRMS (*m*/*z*) [M+H]$^+$ calcd for M = $C_{16}H_{11}{}^{79}Br_2{}^{81}BrO_3$, 490.8316; found, 490.8321.

3-(1,3-Benzodioxol-5-yl)-5-(3-bromophenyl)isoxazole (sery338b)

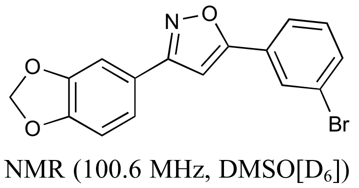

The title compound was prepared according to the GEP 9 starting from **sery338a** to provide **sery338b** as a white solid. Yield 62%. m.p.: 158-160 °C. ^{1}H NMR (400 MHz, DMSO[D$_6$]) δ = 8.08 (t, J = 1.7 Hz, 1H), 7.89 (dt, J = 7.9, 1.1 Hz, 1H), 7.73 (ddd, J = 7.9, 1.7, 1.1 Hz, 1H), 7.67 (s, 1H), 7.53 (t, J = 7.9 Hz, 1H), 7.46-7.39 (m, 2H), 7.09 (d, J = 8.2 Hz, 1H), 6.12 (s, 2H). ^{13}C NMR (100.6 MHz, DMSO[D$_6$]) δ = 167.8, 162.3, 149.0, 148.0, 133.1, 131.5, 128.9, 128.0, 124.4, 122.5, 122.1, 121.1, 108.9, 106.4, 101.6, 99.7. HRMS (*m*/*z*) [M+H]$^+$ calcd for M = $C_{16}H_{10}{}^{79}BrNO_3$, 343.9922; found, 343.9926.

2.3 Synthesis of 3,5-diaryl-1,2,4-oxadiazoles

General Experimental Procedure for the synthesis of 3,5-diaryl-1,2,4-oxadiazoles (GEP 10)[46]

3-(1,3-Benzodioxol-5-yl)-5-phenyl-1,2,4-oxadiazole (anle128b)

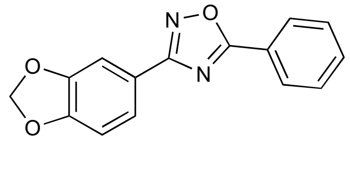

The title compound was prepared according to the published protocol[46]. To a solution of 1,3-benzodioxole-5-carboximidamide (360 mg, 2.0 mmol) and methyl benzoate (300 mg, 2.2 mmol) in ethanol (5 ml) potassium *tert*-butoxide (230 mg, 3.79 mmol) was added and the reaction mixture was heated under reflux for 12 h. After cooling a precipitate was collected by filtration, washed with hot ethanol followed by cold water and dried to provide

anle128b (266 mg) as a white powder. Yield 50%. m.p.: 144-145 °C. ^{1}H NMR (400 MHz, DMSO[D$_6$]) δ = 8.16 (d, J = 7.4 Hz, 2H), 7.73 (t, J = 7.4 Hz, 1H), 7.69-7.62 (m, 3H), 7.52 (d, J = 1.5 Hz, 1H), 7.11 (d, J = 8.1 Hz, 1H), 6.14 (s, 2H). ^{13}C NMR (100.6 MHz, DMSO[D$_6$]) δ = 175.3, 168.1, 150.3, 148.2, 133.5, 129.7, 1278.1, 123.5, 122.4, 120.0, 109.1, 106.8, 102.1. HRMS (*m*/*z*) [M+H]$^+$ calcd for M = $C_{15}H_{10}N_2O_3$, 267.0770; found, 267.0765.

3-(3,4-Dihydroxyphenyl)-5-phenyl-1,2,4-oxadiazole (sery95)

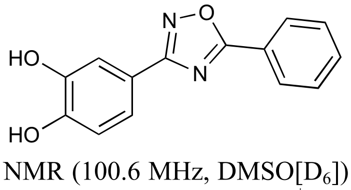

The title compound was prepared according to the GEP 3 starting from **anle128b** and purified by recrystallisation from chloroform/methanol to provide **sery95** as a white solid. Yield 54%. m.p.: 194-196 °C. ^{1}H NMR (400 MHz, DMSO[D$_6$]) δ = 8.15 (d, J = 7.9 Hz, 2H), 7.75-7.61 (m, 3H), 7.50 (d, J = 2.0 Hz, 1H), 7.42 (dd, J = 8.2, 2.0 Hz, 1H), 6.91 (d, J = 8.2 Hz, 1H). ^{13}C NMR (100.6 MHz, DMSO[D$_6$]) δ = 174.8, 168.3, 148.8, 145.8, 133.2, 129.6, 127.8, 123.6, 119.2, 117.0, 116.1, 114.1. HRMS (*m*/*z*) [M+H]$^+$ calcd for M = $C_{14}H_{10}N_2O_3$, 255.0770; found, 255.0765.

3-(1,3-Benzodioxol-5-yl)-5-(3-fluorophenyl)-1,2,4-oxadiazole (sery128)

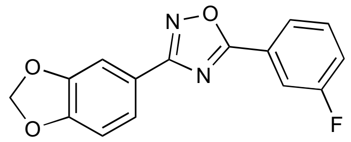

The title compound was prepared according to the GEP 10 starting from 1,3-benzodioxole-5-carboximidamide and ethyl 3-fluorobenzoate and purified by recrystallisation from ethanol (twice) to provide **sery128** as a white solid. Yield 25%. m.p.: 161 °C. ^{1}H NMR (400 MHz, DMSO[D$_6$]) δ = 8.03 (d, J = 7.7 Hz, 1H), 7.97 (dt, J = 9.3, 2.0 Hz, 1H), 7.78-7.69 (m, 1H), 7.67 (dd, J =8.1, 1.6 Hz, 1H), 7.61 (td, J = 8.5, 2.0 Hz, 1H), 7.54 (d, J = 1.6 Hz, 1H), 7.14 (d, J = 8.1 Hz, 1H), 6.16 (s, 2H). ^{13}C NMR (100.6 MHz, DMSO[D$_6$]) δ = 174.1, 168.0, 162.2 (d, J = 244.4 Hz), 150.2, 148.1, 132.0 (d, J = 8.2 Hz), 125.3 (d, J = 8.7 Hz), 124.2 (d, J = 2.8 Hz), 122.3, 120.3 (d, J = 21.0 Hz), 119.6, 114.6 (d, J = 24.1 Hz), 109.0, 106.6, 102.0. HRMS (*m*/*z*) [M+H]$^+$ calcd for M = $C_{15}H_9FN_2O_3$, 285.0675; found, 285.0672.

3-(3,4-Dihydroxyphenyl)-5-(3-fluorophenyl)-1,2,4-oxadiazole (sery140)

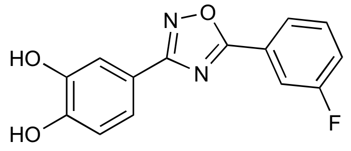

The title compound was prepared according to the GEP 3 starting from **sery128** to provide **sery140** as a grey solid. Yield 60 %. m.p.: 206-207 °C. ^{1}H NMR (400 MHz, DMSO[D$_6$]) δ = 9.64 (s, 1H), 9.45 (s, 1H), 8.00 (dt, J = 7.8, 1.1 Hz, 1H), 7.92 (ddd, J = 9.3, 2.4, 1.4 Hz, 1H), 7.75-7.67 (m, 1H), 7.59 (td, J = 8.4, 2.2 Hz, 1H), 7.49 (d, J = 2.0 Hz, 1H), 7.42 (dd, J = 8.2, 2.0 Hz, 1H), 6.91 (d, J = 8.2 Hz, 1H). ^{13}C NMR (100.6 MHz, DMSO[D$_6$]) δ = 173.8, 168.4, 162.2 (d, J = 245.7 Hz), 148.9, 145.8, 132.0 (d, J = 8.3 Hz), 125.5 (d, J = 8.8 Hz), 124.2, 120.2 (d, J = 21.1 Hz), 119.3, 116.8, 116.1, 114.5 (d, J = 24.1 Hz), 114.1. HRMS (*m*/*z*) [M+H]$^+$ calcd for M = $C_{14}H_9FN_2O_3$, 273.0675; found, 273.0669.

3,5-Bis(1,3-benzodioxol-5-yl)-1,2,4-oxadiazole (sery129)

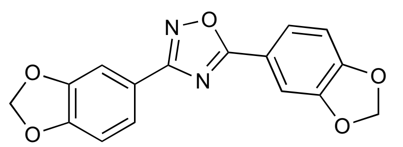

The title compound has been reported previously[47] (CAS 861350-99-4). It was prepared according to the GEP 10 starting from 1,3-benzodioxole-5-carboximidamide and methyl 1,3-benzodioxole-5-carboxylate and purified by washing with hot ethanol followed by hot water to provide **sery129** as a white solid. Yield 38%. m.p.: 208-209 °C. ^{1}H NMR (400 MHz, DMSO[D$_6$]) δ = 7.76 (dd, J = 8.1, 2.1 Hz, 1H), 7.67-7.60 (m, 2H), 7.5 (d, J = 1.6 Hz, 1H), 7.18 (d, J = 8.1 Hz, 1H), 7.12 (d, J = 8.1 Hz, 1H), 6.2 (s, 2H), 6.15 (s, 2H). ^{13}C NMR (100.6 MHz, DMSO[D$_6$]) δ = 174.9, 167.8, 151.6, 150.1, 148.2, 148.0, 123.8, 122.1, 119.0, 117.0, 109.2, 109.0, 107.3, 106.6, 102.4, 101.9.

3,5-Bis(3,4-Dihydroxyphenyl)-1,2,4-oxadiazole hydrobromide (sery139)

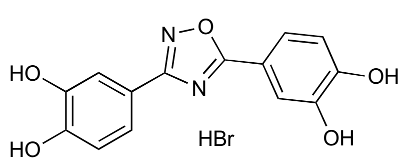

The title compound was prepared according to the GEP 3 starting from **sery129** to provide **sery139** as a grey solid. Yield 90 %. m.p.: 235-240 °C. ^{1}H NMR (400 MHz, DMSO[D$_6$]) δ = 7.55-7.43 (m, 3H), 7.37 (dd, J = 8.2, 1.8 Hz, 1H), 6.94 (d, J = 8.2 Hz, 1H), 6.88 (d, J = 8.2 Hz, 1H). ^{13}C NMR (100.6 MHz, DMSO[D$_6$]) δ = 175.1, 168.0, 150.5, 148.6, 145.9, 145.7, 120.4, 119.1, 117.4, 116.3, 116.1, 114.6, 114.5, 114.1. HRMS (*m*/*z*) [M+H]$^+$ calcd for M = $C_{14}H_{10}N_2O_5$, 287.0668; found, 287.0663.

2.4 Synthesis of 2,4-diaryl-1*H*-imidazoles

General Experimental Procedure for the synthesis of 2,4-diaryl-1*H*-imidazoles (GEP 11)[48]

4-(1,3-Benzodioxol-5-yl)-2-phenyl-1*H*-imidazole (anle131b)

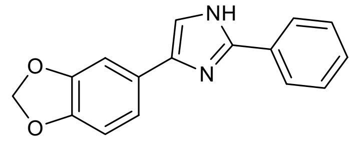

The title compound was prepared according to the published protocol[48] with minor modifications. A mixture of benzamidine hydrochloride (313 mg, 2.0 mmol) and sodium bicarbonate (672 mg, 8.0 mmol) in THF (8 ml) and water (2 ml) was heated under reflux. A solution of 1-(1,3-benzodioxol-5-yl)-2-bromoethanone (486 mg, 2.0 mmol) in THF (2 ml) was added over a period of 30 min, while keeping the reaction under reflux. After addition, the reaction was heated under reflux for 2 h, THF was evaporated under reduced pressure. Ethyl acetate (20 ml) was added to the mixture, organic phase was separated, washed with the brine (10 ml), dried over Na_2SO_4 and evaporated under reduced pressure. The resulting crude product was purified by column chromatography on silica gel ($CHCl_3$:MeOH, 50:1 v/v) to provide **anle131b** (376 mg, 71 %) as a white solid. TLC ($CHCl_3$:MeOH, 50:1 v/v): RF = 0.28. m.p.: 168 °C. ^{1}H NMR (400 MHz, DMSO[D_6] + 1-2% conc. DCl) δ = 7.98 (d, J = 7.2 Hz, 2H), 7.61 (s, 1H), 7.45 (t, J = 7.6 Hz, 2H), 7.40 (d, J = 1.5 Hz, 1H), 7.39-7.30 (m, 2H), 6.93 (d, J = 8.0 Hz, 1H), 6.01 (s, 2H). ^{13}C NMR (100.6 MHz, DMSO[D_6] + 1-2% conc. DCl) δ = 147.8, 146.1, 145.9, 139.5, 130.7, 128.9, 128.3, 125.2, 118.1, 116.2 (2C), 108.7, 105.3, 101.0. HRMS (*m*/*z*) $[M+H]^+$ calcd for M = $C_{16}H_{12}N_2O_2$, 265.0977; found, 265.0976.

4-(3,4-Dihydroxyphenyl)-2-phenyl-1*H*-imidazole hydrobromide (anle132)

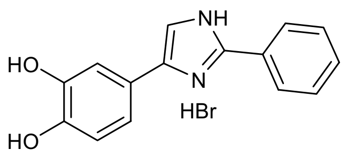

The title compound was prepared according to the GEP 3 starting from **anle131b**. Gray solid. Yield 75%. m.p.: 240-245 °C (dec). ^{1}H NMR (400 MHz, DMSO[D_6]) δ = 9.54 (bs, 1H), 9.19 (bs, 1H), 8.40-7.93 (m, 3H), 7.65 (s, 3H), 7.27 (s, 1H), 7.20 (d, J = 8.0 Hz, 1H), 6.90 (d, J = 8.0 Hz, 1H). ^{13}C NMR (100.6 MHz, DMSO[D_6]) δ = 147.1, 146.0, 143.9, 134.7, 132.2, 129.6, 127.4, 123.4, 118.2, 118.0, 116.2, 115.1, 113.9. HRMS (*m*/*z*) $[M+H]^+$ calcd for M = $C_{15}H_{12}N_2O_2$, 253.0977; found, 253.0972.

2,4-Bis(1,3-benzodioxol-5-yl)-1*H*-imidazole (anle237)

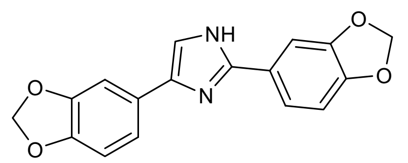

The title compound has been reported previously[5] (CAS 1147315-16-9). It was prepared according to the GEP 11 using 1,3-benzodioxole-5-carboximidamide hydrochloride and 1-(1,3-benzodioxol-5-yl)-2-bromoethanone and purified by recrystallisation from ethanol/water to provide **anle237** as a pale brown solid. Yield 73%. m.p.: 136-138 °C. ^{1}H NMR (400 MHz, DMSO[D_6]) δ = 12.37 (bs, 1H), 7.54 (bs, 1H), 7.52-7.46 (m, 2H), 7.36 (d, J = 1.6 Hz, 1H), 7.33 (dd, J = 8.0, 1.5 Hz, 1H), 7.00 (d, J = 8.5 Hz, 1H), 6.91 (d, J = 8.0 Hz, 1H), 6.06 (s, 2H), 6.00 (s, 2H).

4-(3,4-Dimethoxyphenyl)-2-phenyl-1*H*-imidazole (sery152)

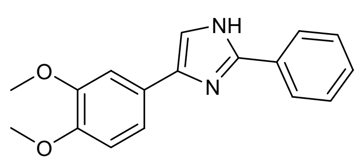

The title compound has been reported previously[49] (CAS 1204141-45-6). It was prepared according to the GEP 11 using benzamidine hydrochloride and 2-bromo-1-(3,4-dimethoxyphenyl)ethanone and purified by column chromatography on silica gel ($CHCl_3$:MeOH, 100:1 v/v). Yield 84%. m.p. 95-98 °C. TLC ($CHCl_3$:MeOH, 100:1 v/v): RF = 0.31. ^{1}H NMR (400 MHz, DMSO[D_6]) δ = 12.57 (s, 1H), 8.00 (d, J = 7.5 Hz, 2H), 7.62 (s, 1H), 7.47 (t, J = 7.5 Hz, 2H), 7.43 (d, J = 1.8 Hz, 1H), 7.41-7.32 (m, 2H), 6.97 (d, J = 8.3 Hz, 1H), 3.84 (s, 3H), 3.77 (s, 3H).

2-(1,3-Benzodioxol-5-yl)-4-(3,4-dimethoxyphenyl)-1*H*-imidazole (sery165)

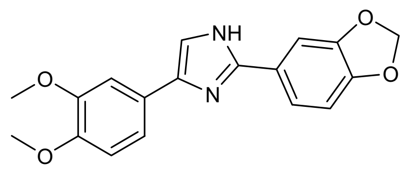

The title compound was prepared according to the GEP 11 using 1,3-benzodioxole-5-carboximidamide hydrochloride and 2-bromo-1-(3,4-dimethoxyphenyl)ethanone and purified by column chromatography on silica gel ($CHCl_3$:MeOH, 95:5 v/v) followed by recrystallisation from methanol/water to provide **sery165** as a pale yellow solid. Yield 82%. TLC ($CHCl_3$:MeOH, 95:5 v/v): RF = 0.55. m.p.: 107-110 °C. ^{1}H NMR (400 MHz, DMSO[D_6] + 1-2% conc. DCl) δ = 7.58 (s, 1H), 7.55-7.51 (m, 2H), 7.39 (d, J = 1.8 Hz, 1H), 7.34 (dd, J = 8.2, 1.8 Hz, 1H), 7.03 (d, J = 8.4 Hz, 1H), 6.97 (d, J = 8.4 Hz, 1H), 6.08 (s, 2H), 3.83 (s, 3H), 3.76 (s, 3H).

^{13}C NMR (100.6 MHz, DMSO[D_6] + 1-2% conc. DCl) δ = 149.0, 147.9 (2C), 147.3, 145.7, 138.9, 126.6, 124.9, 119.1, 116.8, 116.1, 112.1, 108.7, 108.6, 105.5, 101.3, 55.6 (2C). HRMS (*m*/*z*) [M+H]$^+$ calcd for M = $C_{18}H_{16}N_2O_4$, 325.1188; found, 325.1192.

2,4-Bis(3,4-dihydroxyphenyl)-1*H*-imidazole hydrobromide (sery166a)

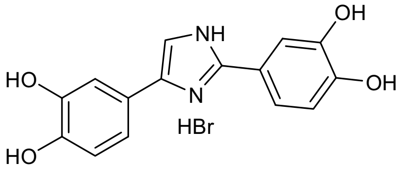

This compound has been reported previously[5] (CAS 1147315-18-1 as a free base). It was prepared according to the GEP 3 starting from **sery165**. Pale brown solid. Yield 89%. m.p.: >300 °C. ^{1}H NMR (400 MHz, DMSO[D_6]) δ = 14.27 (bs, 1H), 13.99 (bs, 1H), 10.40-8.60 (m, 4H), 7.92 (s, 1H), 7.46 (d, J = 2.3 Hz, 1H), 7.41 (dd, J = 8.2, 2.3 Hz, 1H), 7.23 (d, J = 2.2 Hz, 1H), 7.16 (dd, J = 8.2, 2.2 Hz, 1H), 6.98 (d, J = 8.3 Hz, 1H), 6.88 (d, J = 8.2 Hz, 1H).

2-(1,3-Benzodioxol-5-yl)-4-(3-bromophenyl)-1*H*-imidazole (sery345)

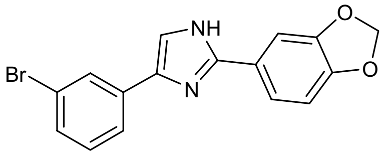

The title compound was prepared according to the GEP 11 using 1,3-benzodioxole-5-carboximidamide hydrochloride and 2-bromo-1-(3-bromophenyl)ethanone and purified by column chromatography on silica gel ($CHCl_3$:MeOH, 100:1 v/v) to provide **sery345** as a white solid. Yield 85%. TLC ($CHCl_3$:MeOH, 100:1 v/v): RF = 0.33. m.p.: 236-238 °C. ^{1}H NMR (400 MHz, DMSO[D_6] + 1-2% conc. DCl) δ = 12.57 (bs, 1H), 8.04 (s, 1H), 7.83 (d, J = 7.6 Hz, 1H), 7.80 (s, 1H), 7.58-7.49 (m, 2H), 7.38 (d, J = 8.0 Hz, 1H), 7.33 (t, J = 7.0 Hz, 1H), 7.02 (d, J = 8.6 Hz, 1H), 6.08 (s, 2H). ^{13}C NMR (100.6 MHz, DMSO[D_6] + 1-2% conc. DCl) δ = 147.7, 147.4, 146.2, 138.3, 136.6, 130.8, 128.7, 126.6, 124.7, 123.1, 122.1, 119.1, 117.0, 108.6, 105.4, 101.3. HRMS (*m*/*z*) [M+H]$^+$ calcd for M = $C_{16}H_{11}{}^{79}BrN_2O_2$, 343.0082; found, 343.0090.

2.5 Synthesis of 1-acetyl-3,5-diaryl-4,5-dihydro-1*H*-pyrazoles

General Experimental Procedure for the synthesis of 1-acetyl-3,5-diaryl-4,5-dihydro-1*H*-pyrazoles (GEP 12)[50]

1-Acetyl-3-(1,3-benzodioxol-5-yl)-4,5-dihydro-5-phenyl-1*H*-pyrazole (anle129)

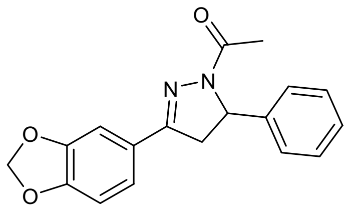

The title compound was prepared according to the published protocol[50]. To a solution of **sery89** (504 mg, 2.0 mmol) in AcOH (12 mL) was added dropwise hydrazine hydrate (243 µl, 250 mg, 5.0 mmol) and kept under stirring at 120 °C for 24 h. The mixture was then poured in ice-water at stirring, the resulting precipitate was collected by filtration and dried. The resulting crude product was purified by recrystallisation from ethanol to provide **anle129** (458 mg, 74%) as a white solid. m.p.: 190-192 °C. ^{1}H NMR (400 MHz, DMSO[D_6]) δ = 7.35 (d, J = 1.6 Hz, 1H), 7.31 (t, J = 7.4 Hz, 2H), 7.28-7.20 (m, 2H), 7.20-7.13 (m, 2H), 6.97 (d, J = 8.1 Hz, 1H), 6.08 (s, 2H), 5.50 (dd, J = 11.8, 4.4 Hz, 1H), 3.80 and 3.76 (2 × d, J = 11.8 Hz, 1H), 3.09 and 3.05 (2 × d, J = 4.5 Hz, 1H), 2.28 (s, 3H). ^{13}C NMR (100.6 MHz, DMSO[D_6]) δ = 167.4, 154.0, 149.3, 148.0, 142.6, 128.8, 127.3, 125.6, 125.4, 122.0, 108.6, 106.1, 101.8, 59.6, 42.4, 21.8. HRMS (*m*/*z*) [M+H]$^+$ calcd for M = $C_{18}H_{16}N_2O_3$, 309.1239; found, 309.1241.

1-Acetyl-4,5-dihydro-3-(3,4-dimethoxyphenyl)-5-(3-fluorophenyl)-1*H*-pyrazole (sery115)

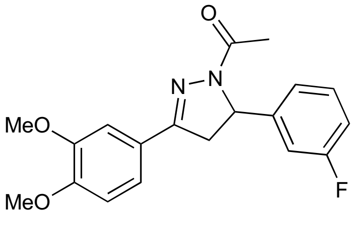

The title compound was prepared according to the GEP 12 starting from **sery97** and purified by recrystallisation from methanol to provide **sery115** as a white solid. Yield 70%. m.p.: 161 °C. ^{1}H NMR (400 MHz, DMSO[D_6]) δ = 7.42-7.32 (m, 2H), 7.28 (dd, J = 8.3, 1.8 Hz, 1H), 7.08 (td, J = 9.0, 1.8 Hz, 1H), 7.04-6.97 (m, 3H), 5.55 (dd, J = 11.8, 4.5 Hz, 1H), 3.87-3.74 (m, 7H), 3.17 (dd, J = 18.1, 4.7 Hz, 1H), 2.31 (s, 3H). ^{13}C NMR (100.6 MHz, DMSO[D_6]) δ = 167.3, 162.3 (d, J = 243.6 Hz), 154.2, 150.8, 148.8, 145.3 (d, J = 6.7 Hz), 130.7 (d, J = 8.1 Hz), 123.5, 121.4, 120.4, 113.9 (d, J = 21.0 Hz), 112.4 (d, J = 21.7 Hz), 111.4, 109.2, 58.8, 55.6, 55.5, 42.1, 21.7. HRMS (*m*/*z*) [M+Na]$^+$ calcd for M = $C_{19}H_{19}FN_2O_3$, 365.1277; found, 365.1276.

1-Acetyl-4,5-dihydro-5-(3,4-dimethoxyphenyl)-3-(4-methoxyphenyl)-1*H*-pyrazole (sery108)

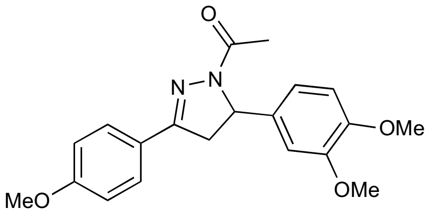

The title compound was prepared according to the GEP 12 starting from **sery96** and purified by recrystallisation from ethanol to provide **sery108** as a white solid. Yield 58%. m.p.: 128-129 °C. ^{1}H NMR (400 MHz, DMSO[D$_6$]) δ = 7.72 (d, J = 8.7 Hz, 2H), 7.01 (d, J = 8.7 Hz, 2H), 6.87 (d, J = 8.3 Hz, 1H), 6.8 (s, 1H), 6.65 (d, J = 8.3 Hz, 1H), 5.45 (dd, J = 11.6, 4.0 Hz, 1H), 3.86-3.75 (m, 4H), 3.72 (s, 3H), 3.71 (s, 3H), 3.10 (dd, J = 17.8, 4.0 Hz, 1H), 2.29 (s, 3H). ^{13}C NMR (100.6 MHz, DMSO[D$_6$]) δ = 167.1, 160.9, 153.9, 148.8, 147.9, 135.0, 128.3, 123.7, 117.0, 114.2, 112.0, 109.6, 59.0, 55.6, 55.5, 55.3, 42.2, 21.7. HRMS (m/z) [M+H]$^+$ calcd for M = $C_{20}H_{22}N_2O_4$, 355.1658; found, 355.1656.

1-Acetyl-4,5-dihydro-3-(3,4-dihydroxyphenyl)-5-phenyl-1*H*-pyrazole (sery105)

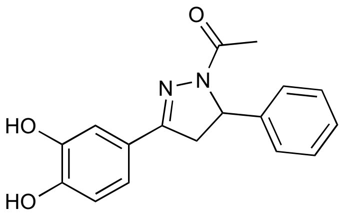

The title compound was prepared according to the GEP 3 starting from **anle129** and purified by column chromatography ($CHCl_3$:MeOH, 19:1 v/v) to provide **sery105** as a yellow solid. Yield 37 %. TLC ($CHCl_3$:MeOH, 19:1 v/v): RF = 0.27. m.p.: 100-102 °C (dec). ^{1}H NMR (400 MHz, $CDCl_3$) δ = 7.55 (bs, 1H), 7.32-7.17 (m, 7H), 7.06 (dd, J = 8.3, 2.0 Hz, 1H), 6.77 (d, J = 8.3 Hz, 1H), 5.55 (dd, J = 11.6, 4.4 Hz, 1H), 3.66 (dd, J = 17.7, 11.6 Hz, 1H), 3.09 (dd, J = 17.7, 4.4 Hz, 1H), 2.46 (s, 3H). ^{13}C NMR (100.6 MHz, DMSO[D$_6$]) δ = 166.9, 154.3, 148.1, 145.5, 142.6, 128.6, 127.1, 125.4, 122.4, 119.3, 115.5, 113.1, 59.1, 42.2, 21.7. HRMS (m/z) [M+H]$^+$ calcd for M = $C_{17}H_{16}N_2O_3$, 297.1239; found, 297.1241.

2.6 Synthesis of 3,5-diaryl-4,5-dihydro-1*H*-pyrazoles

(*E*)-1,3-Bis(3,4-dimethoxyphenyl)-2-propene-1-one (sery112)

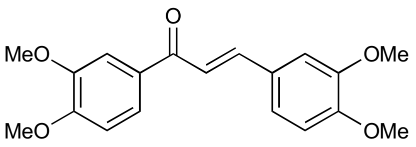

The title compound has been reported previously[51] (CAS 130768-85-3). It was prepared according to the GEP 5 using 1-(3,4-dimethoxyphenyl)ethanone, 3,4-dimethoxybenzaldehyde to provide **sery112** as a yellow solid. Yield 82%. m.p.: 91-92 °C. ^{1}H NMR (400 MHz, DMSO[D$_6$]) δ = 7.92 (dd, J = 8.4, 1.8 Hz, 1H), 7.82 (d, J = 15.5 Hz, 1H), 7.67 (d, J = 15.5 Hz, 1H), 7.60 (d, J = 1.8 Hz, 1H), 7.52 (d, J = 1.7 Hz, 1H), 7.40 (dd, J = 8.5, 1.7 Hz, 1H), 7.10 (d, J = 8.5 Hz, 1H), 7.02 (d, J = 8.4 Hz, 1H), 3.88 (s, 3H), 3.87-3.84 (m, 6H), 3.82 (s, 3H).

General Experimental Procedure for the synthesis of 3,5-diaryl-4,5-dihydro-1*H*-pyrazoles (GEP 13)

4,5-Dihydro-3,5-bis(3,4-dimethoxyphenyl)-1*H*-pyrazole (sery132)

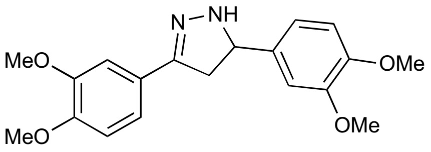

A suspension of **sery112** (330 mg, 1 mmol) and hydrazine hydrate (2.5 ml, 50 mmol) in water (0.7 ml) was heated at 100°C for 1.5 h with stirring. The reaction mixture was cooled down, water (0.2 ml) was added and a resulting precipitate was collected by filtration, washed with water and dried to provide **sery132** (310 mg) as a white solid. Yield 90%. m.p.: 54-57 °C. ^{1}H NMR (400 MHz, DMSO[D$_6$]) δ = 7.42 (s, 1H), 7.03 (d, J = 8.2 Hz, 1H), 6.95 (s, 1H), 6.90 (d, J = 8.2 Hz, 1H), 6.87-6.79 (m, 2H), 4.86 (t, J = 9.9 Hz, 1H), 3.99-3.81 (m, 12H), 3.43 (dd, J = 16.2, 10.5 Hz, 1H), 3.00 (dd, J = 16.2, 9.4 Hz, 1H). ^{13}C NMR (100.6 MHz, DMSO[D$_6$]) δ = 151.7, 150.1, 149.4, 149.3, 148.7, 135.4, 126.0, 119.5, 118.8, 111.3, 110.7, 109.5, 108.3, 64.4, 56.2-55.9 (4C), 41.8. HRMS (m/z) [M+H]$^+$ calcd for M = $C_{19}H_{22}N_2O_4$, 343.1658; found, 343.1657.

4,5-Dihydro-5-(3,4-dimethoxyphenyl)-3-(4-methoxyphenyl)-1*H*-pyrazole (sery133)

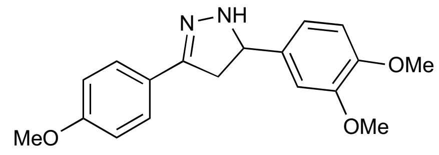

The title compound has been reported previously[52] (CAS 130768-85-3). It was prepared according to the GEP 13 starting from **sery96** to provide **sery133** as a white solid. Yield 85%. m.p.: 60-65 °C (lit[52] 100-105 °C). ^{1}H NMR (400 MHz, DMSO[D$_6$]) δ = 7.56 (d, J = 8.1 Hz, 2H), 7.29 (s, 1H), 7.06-6.82 (m, 5H), 4.74 (t, J = 10.7 Hz, 1H), 3.76 (s, 3H), 3.74 (s, 3H), 3.73 (s, 3H), 3.36 (dd, J = 15.7, 10.7 Hz, 1H), 2.80 (dd, J = 15.7, 11.5 Hz, 1H). ^{13}C NMR (100.6 MHz, DMSO[D$_6$]) δ = 159.3, 148.8, 148.7, 147.9, 135.4, 126.8, 126.0, 118.6, 113.9, 111.7, 110.4, 63.5, 55.6, 55.4, 55.1, 40.8.

(*E*)-3-(3,4-Dimethoxyphenyl)-1-phenyl-2-propene-1-one (sery113)

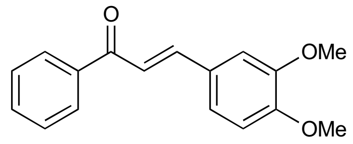

The title compound has been reported previously[42] (CAS 53744-27-7). It was prepared according to the GEP 5 using 1-phenylethanone, 3,4-dimethoxybenzaldehyde to provide **sery113** as a yellow solid. Yield 90%. m.p.: 85-87 °C (lit[42] 84 °C). ^{1}H NMR (400 MHz, DMSO[D$_6$]) δ = 8.15 (d, J = 8.3 Hz, 2H), 7.83 (d, J = 15.5 Hz, 1H), 7.72 (d, J = 15.5 Hz, 1H), 7.66 (d, J = 7.4 Hz, 1H), 7.6-7.53 (m, 3H), 7.39 (dd, J = 8.4, 1.9 Hz, 1H), 7.02 (d, J = 8.4 Hz, 1H), 3.87 (s, 3H), 3.82 (s, 3H).

4,5-Dihydro-5-(3,4-dimethoxyphenyl)-3-phenyl-1*H*-pyrazole (sery135)

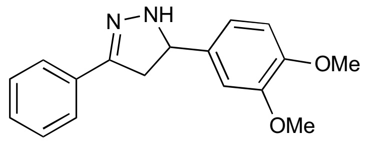

The title compound has been reported previously[52] (CAS 399031-89-1). It was prepared according to the GEP 13 starting from **sery113** to provide **sery135** as a white solid. Yield 82%. m.p.: 59-64 °C (lit[52] 85-88 °C). ^{1}H NMR (400 MHz, DMSO[D$_6$]) δ = 7.63 (d, J = 7.4 Hz, 2H), 7.50 (s, 1H), 7.37 (t, J = 7.4 Hz, 2H), 7.30 (t, J = 7.2 Hz, 1H), 7.00 (s, 1H), 6.90 (s, 2H), 4.79 (t, J = 10.7 Hz, 1H), 3.74 (s, 3H), 3.73 (s, 3H), 3.40 (dd, J = 16.2, 10.7 Hz, 1H), 2.84 (dd, J = 16.2, 11.1 Hz, 1H). ^{13}C NMR (100.6 MHz, DMSO[D$_6$]) δ = 148.7, 148.6, 148.0, 135.3, 133.3, 128.5, 128.0, 125.3, 118.6, 111.7, 110.4, 63.6, 55.6, 55.4, 40.5.

3-(1,3-Benzodioxol-5-yl)-4,5-dihydro-5-phenyl-1*H*-pyrazole (sery136)

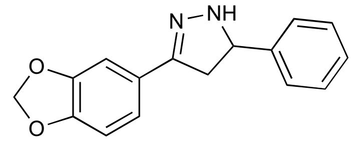

The title compound was prepared according to the GEP 13 starting from **sery89** to provide **sery136** as a white solid. Yield 85%. m.p.: 108-110 °C. ^{1}H NMR (400 MHz, $CDCl_3$) δ = 7.44-7.23 (m, 6H), 7.05 (d, J = 7.9 Hz, 1H), 6.81 (d, J = 8.0 Hz, 1H), 6.10-5.82 (m, 3H), 4.92 (t, J = 9.4 Hz, 1H), 3.44 (dd, J = 16.2, 10.6 Hz, 1H), 3.02 (dd, J = 16.2, 8.6 Hz, 1H). ^{13}C NMR (100.6 MHz, $CDCl_3$) δ = 151.2, 148.4, 148.1, 142.9, 129.0, 127.9, 127.4, 126.4, 120.6, 108.2, 106.1, 101.4, 64.4, 41.7. HRMS (*m/z*) $[M+H]^+$ calcd for M = $C_{16}H_{14}N_2O_2$, 267.1134; found, 267.1133.

(*E*)-3-(3,4-Dihydroxyphenyl)-1-(3,4-dimethoxyphenyl)-2-propene-1-one (sery131)

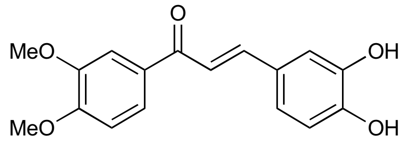

The title compound has been reported previously[53] (CAS 152809-90-0) and prepared according to the published protocol[53] using 1-(3,4-dimethoxyphenyl)ethanone, 3,4-Di(tetrahydropyran-2-oxy)benzaldehyde to provide **sery131** as a yellow solid. Yield 40%. m.p.: 142-144 °C (lit[53] 132-137 °C). ^{1}H NMR (400 MHz, DMSO[D$_6$]) δ = 9.38 (bs, 1,6H), 7.83 (dd, J = 8.5, 1.8 Hz, 1H), 7.67-7.53 (m, 3H), 7.26 (d, J = 1.8 Hz, 1H), 7.18 (dd, J = 8.2, 1.8 Hz, 1H), 7.08 (d, J = 8.5 Hz, 1H), 7.81 (d, J = 8.2 Hz, 1H), 3.86 (s, 3H), 3.85 (s, 3H). ^{13}C NMR (100.6 MHz, DMSO[D$_6$]) δ = 187.2, 152.9, 148.7, 148.5, 145.6, 144.0, 130.9, 126.4, 122.9, 122.0, 118.3, 115.7, 115.5, 110.9, 110.7, 55.7, 55.6.

4,5-Dihydro-5-(3,4-dihydroxyphenyl)-3-(3,4-dimethoxyphenyl)-1*H*-pyrazole (sery144)

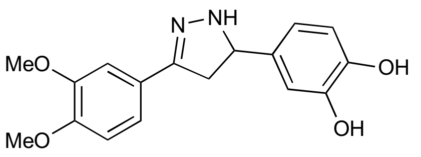

The title compound was prepared according to the GEP 13 starting from **sery131** to provide **sery144** as a white solid. Yield 53%. m.p.: 190-191 °C. ^{1}H NMR (400 MHz, DMSO[D$_6$]) δ = 8.80 (s, 1.9H), 7.26 (d, J = 1.3 Hz, 1H), 7.19 (d, J = 2.5 Hz, 1H), 7.06 (dd, J = 8.2, 1.3 Hz, 1H), 6.94 (d, J = 8.2 Hz, 1H), 6.76 (d, J = 1.5 Hz, 1H), 6.66 (d, J = 8.1 Hz, 1H), 6.59 (dd, J = 8.1, 1.5 Hz, 1H), 4.62 (td, J = 10.3, 2.5 Hz, 1H), 3.77-3.76 (6H), 3.40-3.24 (m, 1H, overlapping with water), 2.74 (dd, J = 16.2, 10.3 Hz, 1H). ^{13}C NMR (100.6 MHz, DMSO[D$_6$]) δ = 149.1, 148.7, 148.6, 145.2, 144.4, 134.1, 126.3, 118.6, 117.4, 115.3, 113.8, 111.4, 108.2, 63.3, 55.5, 55.3, 40.8. HRMS (m/z) $[M+H]^+$ calcd for M = $C_{17}H_{18}N_2O_4$, 315.1345; found, 315.1346.

4,5-Dihydro-3-(3,4-dimethoxyphenyl)-5-(3-fluorophenyl)-1*H*-pyrazole (sery145)

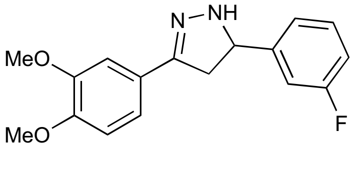

The title compound was prepared according to the GEP 13 starting from **sery97** to provide **sery145** as a white solid. Yield 62%. m.p.: 119-122 °C. ^{1}H NMR (400 MHz, CDCl$_3$) δ = 7.41 (d, J = 1.6 Hz, 1H), 7.34-7.27 (m, 1H), 7.17-7.08 (m, 2H), 7.02 (dd, J = 8.3, 1.7 Hz, 1H), 6.97 (td, J = 8.3, 2.0 Hz, 1H), 6.84 (d, J = 8.3 Hz, 1H), 5.92 (bs, 1H), 4.90 (t, J = 9.8 Hz, 1H), 3.92 (s, 3H), 3.90 (s, 3H), 3.47 (dd, J = 16.3, 10.6 Hz, 1H), 3.00 (d, J = 16.3, 9.0 Hz, 1H). ^{13}C NMR (100.6 MHz, CDCl$_3$) δ = 163.2 (d, J = 246.7 Hz), 151.5, 150.2, 149.3, 145.6 (d, J = 6.5 Hz), 130.5 (d, J = 8.2 Hz), 125.8, 122.1 (d, J = 2.6 Hz), 119.5, 114.7 (d, J = 21.2 Hz), 113.4 (d, J = 21.9 Hz), 110.7, 108.4, 63.9, 56.05, 56.0, 41.7. HRMS (m/z) $[M+H]^+$ calcd for M = $C_{17}H_{17}FN_2O_2$, 301.1352; found, 301.1350.

2.7 Synthesis of 2,4-diaryl-1*H*-pyrroles[54]

1-(1,3-Benzodioxol-5-yl)-4-nitro-3-phenylbutane-1-one (anle130a)

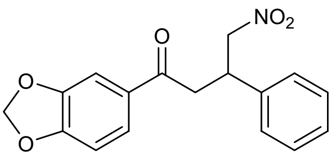

The title compound was prepared according to the published protocol[54]. A solution of **sery89** (757 mg, 3 mmol) in MeOH (30 mL) was treated with Et_2NH (2.07 ml, 1.46 g, 20 mmol) and CH_3NO_2 (1.08 ml, 1.22 g, 20 mmol) and heated under reflux for 15 h. The solvent was evaporated under reduced pressure, the residue was partitioned between CH_2Cl_2 (30 ml) and water (30 ml), and acidified with 1 M HCl. The aqueous layer was extracted with a further portion of CH_2Cl_2 (30 ml), and the combined organics were washed with water (30 ml), brine (30 ml) and dried over Na_2SO_4. The solvent was removed under reduced pressure, and the resulting oil was purified by column chromatography on silica gel (hexane:EtOAc, 2:1 v/v) to provide **anle130a** (750 mg, 80%) as a yellow oil. TLC (hexane:EtOAc, 2:1 v/v): RF = 0.57. ^{1}H NMR (400 MHz, CDCl$_3$) δ = 7.52 (dd, J = 8.2, 1.7 Hz, 1H), 7.39 (d, J = 1.7 Hz, 1H), 7.37-7.31 (m, 2H), 7.31-7.24 (m, 3H), 6.84 (d, J = 8.2 Hz, 1H), 6.04 (s, 2H), 4.83 and 4.82 (2 × d, J = 12.5 Hz, 1H), 4.69 and 4.67 (2 × d, J = 12.5 Hz, 1H), 4.20 (q, J = 7.1 Hz, 1H), 3.45-3.29 (m, 2H). ^{13}C NMR (100.6 MHz, CDCl$_3$) δ = 194.8, 152.1, 148.3, 139.2, 131.2, 129.0, 127.8, 127.4, 124.4, 107.9, 107.8, 101.9, 79.6, 41.2, 39.4. HRMS (m/z) $[M+Na]^+$ calcd for M = $C_{17}H_{15}NO_5$, 336.0848; found, 336.0847.

2-(1,3-Benzodioxol-5-yl)-4-phenyl-1*H*-pyrrole (anle130b)

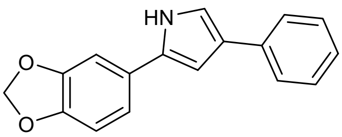

The title compound was prepared according to the published protocol[54]. A stirred solution of **anle130a** (707 mg, 2.25 mmol) in MeOH (20 ml) and THF (40 ml) at room temperature was treated with KOH (633 mg, 11.3 mmol). After 1 h the reaction mixture was added dropwise to a solution of H_2SO_4 (4 ml) in MeOH (20 ml) at 0 °C and stirred 1.5 h at room temperature. Water (50 ml) and ice (50 ml) were added, and the mixture was neutralized with aqueous 4 M NaOH and extracted with CH_2Cl_2 (2 × 50 ml). The combined organic fractions were washed with water (50 ml) and brine (50 ml), dried over Na_2SO_4 and evaporated under reduced pressure. The resulting oil was treated with AcOH (10 ml) and NH_4OAc (870 mg, 11.3 mmol), the solution was heated at 100 °C for 1 h. The reaction mixture was cooled down, ice (50 ml) was added, and the mixture was neutralized with aqueous 4M NaOH. The solution was extracted with CH_2Cl_2 (2 × 50 ml), the combined organic fractions were washed with brine (50 ml), dried over Na_2SO_4 and evaporated under reduced pressure. The crude product was purified by column chromatography on silica gel (CH_2Cl_2) followed by recrystallisation from $CHCl_3$/petroleum benzine 40-60 °C to provide **anle130b** (321 mg, 54%) as a pink solid. TLC (CH_2Cl_2): RF = 0.7. m.p.: 188-190 °C (dec). ^{1}H NMR (400 MHz, CDCl$_3$) δ = 8.32 (bs, 1H), 7.56 (dd, J = 8.1, 1.0 Hz, 2H), 7.36 (t, J = 7.7 Hz, 2H), 7.20 (t, J = 7.4 Hz, 1H), 7.10 (t, J = 1.9 Hz, 1H), 7.01 (d, J = 1.5 Hz, 1H), 6.98 (dd, J = 8.0, 1.6 Hz, 1H), 6.84 (d, J = 8.0 Hz, 1H), 6.69 (t, J = 2.2 Hz, 1H), 5.99 (s, 2H). ^{13}C NMR (100.6 MHz, CDCl$_3$) δ = 148.2, 146.5, 135.5, 133.1, 128.6, 127.1, 126.5, 125.7, 125.2, 117.3, 115.0, 108.7, 105.1, 103.4, 101.1. HRMS (m/z) $[M+H]^+$ calcd for M = $C_{17}H_{13}NO_2$, 264.1025; found, 264.1023.

3-(3,4-Dimethoxyphenyl)-4-nitro-1-phenylbutane-1-one (sery155)

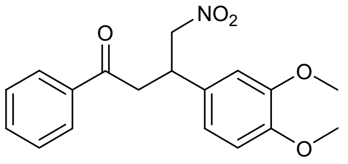

The title compound was prepared analogously to the procedure for **anle130a** using **sery113** and purified by column chromatography on silica gel (hexane:EtOAc, 1:1 v/v) to provide **sery155** as a white solid. Yield 79%. TLC (hexane:EtOAc, 1:1 v/v): RF = 0.7. m.p.: 98-100 °C. ^{1}H NMR (400 MHz, DMSO[D_6]) δ= 7.94 (d, J = 7.6 Hz, 2H), 7.63 (t, J = 7.6 Hz, 1H), 7.52 (t, J = 7.6 Hz, 2H), 7.00 (s, 1H), 6.84 (s, 2H), 4.95 (dd, J = 12.8, 5.8 Hz, 1H), 4.83 (dd, J = 12.8, 9.5 Hz, 1H), 3.99 (m, 1H), 3.73 (s, 3H), 3.70 (s, 3H), 3.55 (dd, J = 17.7, 7.1 Hz, 1H), 3.46 (dd, J = 17.7, 7.0 Hz, 1H). ^{13}C NMR (100.6 MHz, DMSO[D_6]) δ= 197.6, 148.7, 147.9, 136.5, 133.4, 132.2, 128.7, 127.9, 119.9, 111.6, 111.4, 79.8, 55.5, 55.4, 41.2, 39.0. HRMS (*m*/*z*) $[M+H]^+$ calcd for M = $C_{18}H_{19}NO_5$, 330.1341; found, 330.1341.

4-(3,4-Dimethoxyphenyl)-2-phenyl-1*H*-pyrrole (sery160)

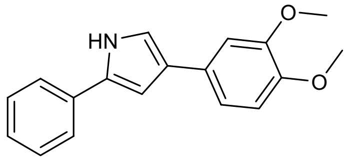

The title compound was prepared analogously to the procedure for **anle130b** starting from **sery155** and purified by column chromatography on silica gel (hexane:EtOAc, 3:1 v/v) followed by recrystallisation from hexane/EtOAc to provide **sery155** as a white solid. Yield 45%. TLC (hexane:EtOAc, 3:1 v/v): RF = 0.25. m.p.: 167-169 °C. ^{1}H NMR (400 MHz, DMSO[D_6]) δ= 11.34 (s, 1H), 7.68 (d, J = 7.5 Hz, 2H), 7.36 (t, J = 7.5 Hz, 2H), 7.26 (s, 1H), 7.20-7.14 (m, 2H), 7.12 (dd, J = 8.2, 1.9 Hz, 1H), 6.94-6.87 (m, 2H), 3.82 (s, 3H), 3.74 (s, 3H). ^{13}C NMR (100.6 MHz, DMSO[D_6]) δ= 149.0, 146.7, 132.8, 131.9, 128.9, 128.7, 125.5, 124.9, 123.3, 116.5, 116.0, 112.3, 108.9, 103.3, 55.6, 55.5. HRMS (*m*/*z*) $[M+H]^+$ calcd for M = $C_{18}H_{17}NO_2$, 280.1338; found, 330.1337.

4-(3,4-Dihydroxyphenyl)-2-phenyl-1*H*-pyrrole (sery166b)

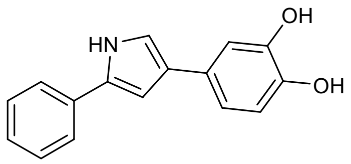

The title compound was prepared according to the GEP 3 starting from **anle160** and purified by column chromatography on silica gel ($CHCl_3$:MeOH, 95:5 v/v) followed by recrystallisation from $CHCl_3/CH_3CN$. Brown solid. Yield 50%. TLC ($CHCl_3$:MeOH, 95:5 v/v): RF = 0.4. m.p.: >300 °C. ^{1}H NMR (400 MHz, DMSO[D_6]) δ= 11.24 (s, 1H), 8.69 (s, 2H), 7.65 (d, J = 7.4 Hz, 2H), 7.35 (t, J = 7.4 Hz, 2H), 7.15 (t, J = 7.4 Hz, 1H), 7.07 (s, 1H), 6.95 (d, J = 2.0 Hz, 1H), 6.85 (dd, J = 8.1, 2.0 Hz, 1H), 6.74 (s, 1H), 6.69 (d, J = 8.1 Hz, 1H). ^{13}C NMR (100.6 MHz, DMSO[D_6]) δ= 145.3, 143.1, 132.8, 131.7, 128.7, 127.4, 125.5, 125.3, 123.3, 115.8, 115.7, 115.2, 112.4, 102.9. HRMS (*m*/*z*) $[M+H]^+$ calcd for M = $C_{16}H_{13}NO_2$, 252.1025; found, 252.1023.

1,3-Bis(3,4-dimethoxyphenyl)-4-nitrobutane-1-one (sery154)

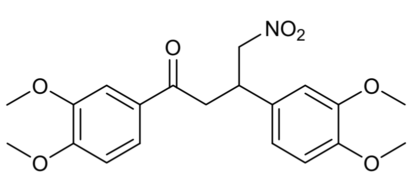

The title compound was prepared analogously to the procedure for **anle130a** using **sery112** and purified by column chromatography on silica gel (hexane:EtOAc, 1:1 v/v) followed by recrystallisation from CH_2Cl_2/Et_2O to provide **sery154** as a white solid. Yield 73%. TLC (hexane:EtOAc, 1:1 v/v): RF = 0.33. m.p.: 134 °C. ^{1}H NMR (400 MHz, DMSO[D_6]) δ= 7.63 (dd, J = 8.4, 1.8 Hz, 1H), 7.40 (d, J = 1.8 Hz, 1H), 7.05 (d, J = 8.4 Hz, 1H), 7.00 (s, 1H), 6.83 (s, 2H), 4.98-4.78 (m, 2H), 3.98 (m, 1H), 3.83 (s, 3H), 3.79 (s, 3H), 3.73 (s, 3H), 3.69 (s, 3H), 3.52 (dd, J = 17.4, 7.3 Hz, 1H), 3.34 (dd, J = 17.4, 6.9 Hz, 1H). ^{13}C NMR (100.6 MHz, DMSO[D_6]) δ= 196.0, 153.2, 148.6, 148.5, 147.9, 132.3, 129.4, 122.8, 119.9, 111.6, 111.5, 110.8, 110.2, 79.8, 55.8, 55.6, 55.5, 55.4, 40.7, 39.3. HRMS (*m*/*z*) $[M+Na]^+$ calcd for M = $C_{20}H_{23}NO_7$, 412.1372; found, 412.1365.

2,4-Bis(3,4-dimethoxyphenyl)-1*H*-pyrrole (sery167)

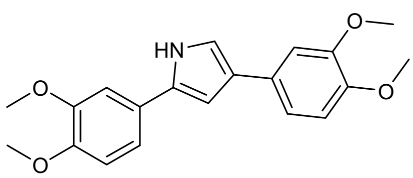

The title compound was prepared analogously to the procedure for **anle130b** starting from **sery154** and purified by column chromatography on silica gel (hexane:EtOAc, 1:1 v/v) to provide **sery167** as a white solid. Yield 39%. TLC (hexane:EtOAc, 1:1 v/v): RF = 0.39. m.p.: 181-182 °C. ^{1}H NMR (400 MHz, $CDCl_3$) δ= 8.42 (bs, 1H), 7.12 (dd, J = 8.2, 2.0 Hz, 1H), 7.08 (d, J = 2.0 Hz, 1H), 7.06 (d, J = 2.0 Hz, 1H), 7.05-6.99 (m, 2H), 6.88 (d, J = 8.2 Hz, 2H), 6.65 (dd, J = 2.6, 1.7 Hz, 1H), 3.94 (s, 3H), 3.93 (s, 3H), 3.90 (s, 3H), 3.89 (s, 3H). ^{13}C NMR (100.6 MHz, $CDCl_3$) δ= 149.3, 149.1, 148.0, 147.4, 133.1, 128.9, 126.4,

126.0, 117.4, 116.2, 114.4, 111.6 (2C), 109.0, 108.0, 103.1, 56.0 (3C), 55.9. HRMS (*m*/*z*) $[M+H]^+$ calcd for M = $C_{20}H_{21}NO_4$, 340.1549; found, 340.1553.

2.6 Synthesis of other compounds

3-(1,3-Benzodioxol-5-yl)-4,5-dihydro-4-hydroxy-5-phenyl-1*H*-pyrazole (anle127b)

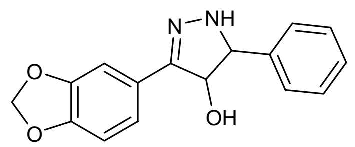

The title compound was prepared according to the published protocol[13]. A mixture of *trans*-1-(1,3-benzodioxol-5-yl)-2,3-epoxy-3-phenylpropane-1-one[55] (587 mg, 2.19 mmol) and hydrazine hydrate (181 µl, 186 mg, 3.72 mmol) in ethanol (10 ml) was heated under reflux for 6 h. Solvent was evaporated and the residue crystallised from MeOH to provide **anle127b** (348 mg, 56%) as a pale yellow solid. m.p.: 175-177 °C. ^{1}H NMR (400 MHz, DMSO[D$_6$]) δ = 7.56 (d, *J* = 3.3 Hz, 1H), 7.38-7.29 (m, 4H), 7.28-7.19 (m, 3H), 6.90 (d, *J* = 8.0 Hz, 1H), 6.01 (s, 2H), 5.95 (d, *J* = 7.8 Hz, 1H), 4.91 (t, *J* = 7.3 Hz, 1H), 4.49 (dd, J = 7.0, 3.3 Hz, 1H). ^{13}C NMR (100.6 MHz, DMSO[D$_6$]) δ = 149.2, 147.5, 147.2, 141.7, 128.6, 127.4, 127.1, 126.7, 120.0, 108.3, 105.7, 101.1, 82.3, 72.3. HRMS (*m*/*z*) $[M+H]^+$ calcd for M = $C_{16}H_{14}N_2O_3$, 283.1083; found, 283.1084.

3-(1,3-Benzodioxol-5-yl)-4-hydroxy-5-phenyl-1*H*-pyrazole (anle127c)

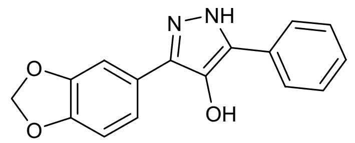

The title compound was prepared according to the published protocol[56]. A solution of bromine (62 µl, 192 mg, 1.20 mmol) in $CHCl_3$ (2 ml) was added to a suspension of **anle127b** (340 mg, 1.20 mmol) in $CHCl_3$ (5 ml). The mixture was stirred for 30 min, the resulting precipitate was filtered off, washed with $CHCl_3$ (5 ml), and dried 10 min on the air. The solid was dissolved in DMSO (3 ml), the solution was added slowly to a ice water (30 ml) at stirring, the resulting precipitate was collected by filtration and dried. The resulting crude product was purified by column chromatography on silica gel (hexane:EtOAc, 2:1 v/v) to provide **anle127c** (70 mg, 20 %) as a white solid. TLC (hexane:EtOAc, 2:1 v/v): RF = 0.21. m.p.: 218-219 °C. ^{1}H NMR (400 MHz, DMSO[D$_6$] + 1-2% conc. DCl) δ = 8.23 (bs, 1H), 7.89 (d, *J* = 7.6 Hz, 2H), 7.53-7.36 (m, 4H), 7.28 (t, *J* = 7.4 Hz, 1H), 6.99 (d, *J* = 8.6 Hz, 1H), 6.04 (s, 2H). ^{13}C NMR (100.6 MHz, DMSO[D$_6$] + 1-2% conc. DCl) δ = 147.6, 146.5, 135.8 (2C), 134.9, 131.7, 128.8, 127.2, 125.9, 125.7, 119.5, 108.8, 106.1, 101.2. HRMS (*m*/*z*) $[M+H]^+$ calcd for M = $C_{16}H_{12}N_2O_3$, 281.0926; found, 281.0928.

trans-1-(1,3-Benzodioxol-5-yl)-3-(3-bromophenyl)-2,3-epoxypropane-1-one (sery298b)

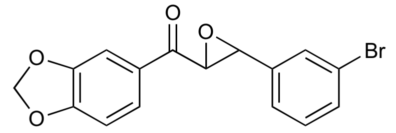

The title compound was prepared according to the published protocol[13] starting from **sery298a** to provide **sery298b** as a white solid. Yield 93%. m.p.: 82-83 °C. ^{1}H NMR (400 MHz, DMSO[D$_6$]) δ = 7.74 (dd, *J* = 8.2, 1.3 Hz, 1H), 7.63 (s, 1H), 7.58 (d, *J* = 8.0 Hz, 1H), 7.51 (d, *J* = 1.3 Hz, 1H), 7.46 (d, *J* = 7.7 Hz, 1H), 7.37 (t, *J* = 7.7 Hz, 1H), 7.08 (d, *J* = 8.2 Hz, 1H), 6.16 (s, 1H), 6.15 (s, 1H), 4.83 (d, *J* = 1.4 Hz, 1H), 4.13 (d, *J* = 1.4 Hz, 1H). ^{13}C NMR (100.6 MHz, DMSO[D$_6$]) δ = 190.6, 152.3, 148.1, 138.6, 131.6, 130.6, 130.0, 129.0, 125.7, 125.4, 121.9, 108.4, 107.3, 102.3, 59.1, 57.5. HRMS (*m*/*z*) $[M+Na]^+$ calcd for M = $C_{16}H_{11}{}^{79}BrO_4$, 368.9738; found, 368.9741.

3-(1,3-Benzodioxol-5-yl)-5-(3-bromophenyl)-4,5-dihydro-4-hydroxy-1*H*-pyrazole (sery300a)

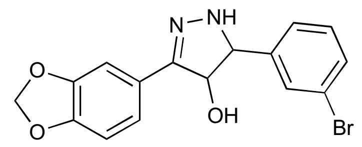

The title compound was prepared using an analogous procedure to that described for **anle127b** starting from **sery298b** and purified by recrystallisation from methanol to provide **sery300a** as a white solid. Yield 51%. m.p.: 180-182 °C. ^{1}H NMR (400 MHz, DMSO[D$_6$]) δ = 7.62 (d, *J* = 3.4 Hz, 1H), 7.49-7.44 (m, 2H), 7.34-7.29 (m, 2H), 7.26-7.21 (m, 2H), 6.90 (d, *J* = 8.0 Hz, 1H), 6.04-5.98 (m, 3H), 4.92 (t, *J* = 7.4 Hz, 1H), 4.51 (dd, *J* = 7.0, 3.4 Hz, 1H). ^{13}C NMR (100.6 MHz, DMSO[D$_6$]) δ = 149.2, 147.3, 147.1, 144.4, 130.7, 130.0, 129.2, 126.7, 125.6, 121.7, 119.9, 108.2, 105.6, 101.0, 82.0, 71.3. HRMS (*m*/*z*) $[M+H]^+$ calcd for M = $C_{16}H_{13}{}^{79}BrN_2O_3$, 361.0188; found, 361.0191.

References

1. Anselme, J. P. Convenient and practical preparation of dibenzoylmethane. *J. Org. Chem.* **32**, 3716-3718 (1967).
2. Choshi, T. *et al.* Synthesis of dibenzoylmethane derivatives and inhibition of mutagenicity in Salmonella typhimurium. *Chem. Pharm. Bull.* **40**, 1047-1049 (1992).
3. Hauser, C. R.; Eby, C. J. N- and C-benzoylation of p-aminoacetophenone with methyl benzoate by sodium amide. Synthesis of β-diketones having p-acylamino and p-hydroxy groups. *J. Org. Chem.* **22**, 909-912 (1957).
4. Vanelle, P. *et al.* Functional derivatives of 5-benzo[1,3]dioxol-5-yl-1-methyl-1*H*-imidazole-2-carbaldehyde and evaluation of leishmanicidal activity. *Eur. J. Med. Chem.* **35**, 157-162 (2000).
5. Esposito, L. A. *et al.* Compounds, compositions and methods for the treatment of β-amyloid diseases and synucleinopathies. *U.S. Pat. Appl. Publ.* US 2009111863 A1 (2009).
6. Hu, Y.-Z.; Clive, D. L. J. Synthesis of the aromatic unit of calicheamicin γ 1I. *J. Chem. Soc., Perkin Trans. 1* **9**, 1421-1424 (1997).
7. Nam, N.-H. *et al.* Synthesis and cytotoxicity of 2,5-dihydroxychalcones and related compounds. *Arch. Pharm. Res.* **27**, 581-588 (2004).
8. Harris, R. L. N.; Huppatz, J. L. Synthetic plant growth regulators. The synthesis of C-o-Carboxyphenyl derivatives of some five-membered heterocycles. *Aust. J. Chem.* **30**, 2225-2240 (1977).
9. Pinto, D. C. G. A. *et al.* New bis(chalcones) and their transformation into bis(pyrazoline) and bis(pyrazole) derivatives. *Eur. J. Org. Chem.*, 747-755 (2003).
10. Liu, Y. *et al.* A practical and chemoselective reduction of nitroarenes to anilines using activated iron. *Adv. Synth. Catal.* **347**, 217-219 (2005).
11. Bhagwan, J. *et al.* Synthesis and spectral studies of some potential antibacterial diaryl 1,3-diketones. *J. Inst. Chemists (India)* **54**, 266-268 (1982).
12. Chauhan, S. S. *et al.* Solid phase synthesis of novel pyrazole derivatives from diaryl 1,3-diketones under microwave irradiation. *J. Ind. Chem. Soc.* **82**, 1016-1018 (2005).
13. Bhat, B. A. *et al.* Synthesis of 3,5-diphenyl-1*H*-pyrazoles. *Synth. Commun.* **35**, 1135-1142 (2005).
14. LeBlanc, R. *et al.* Synthesis and cytotoxicity of epoxide and pyrazole analogs of the combretastatins. *Bioorg. Med. Chem.* **13**, 6025-6034 (2005).
15. Lopez, J. A. *et al.* Galiposin A new *β*-hydroxychalcone from Galipea granulosa. *Planta Medica* **64**, (1998).
16. Lake, T.; Snow, A. D. Dihydroxy aryl compounds, compositions and methods for the treatment of islet amyloid polypeptide (IAPP) accumulation in diabetes. *U.S. Pat. Appl. Publ.* WO 2009111611 A2 (2009).
17. Bradley, W.; Robinson, R. Hydrolytic fission of some substituted dibenzoylmethanes. *J. Chem. Soc.*, 2356-2367 (1926).
18. Cao, S. X. *et al.* Pyrazole and triazole derivatives as glucokinase activators and their preparation, pharmaceutical compositions and use in the treatment of diseases. *PCT Int. Appl.* WO 2007061923 A2 (2007).
19. Vangoor, F. F. *et al.* Preparation of substituted pyrazoles as modulators of ATP-binding cassette transporters. *PCT Int. Appl.* WO 2004080972 A1 (2004).
20. Hubaud, J.-C. *et al.* Synthesis and spectroscopic examination of various substituted 1,3-dibenzoylmethane, active agents for UVA/UVB photoprotection. *J. Photochem. Photobiol., B* **92**, 103-109 (2008).
21. Shaw, A. Y. *et al.* 3,5-Diaryl-1*H*-pyrazole as a molecular scaffold for the synthesis of apoptosis-inducing agents. *Bioorg. Med. Chem.* **18**, 3270-3278 (2010).
22. Lal, B. *et al.* Preparation of pyrrolidinylchromenones as inhibitors of cyclin-dependent kinases. *U.S. Pat. Appl.* US 20070015802 A1 (2007).
23. Winkle, M. R.; Ronald, R. C. Regioselective metalation reactions of some substituted (methoxymethoxy)arenes. *J. Org. Chem.* **47**, 2101-2108 (1982).
24. Li, Y.-X. *et al.* Design and syntheses of novel phthalazin-1(2*H*)-one derivatives as acetohydroxyacid synthase inhibitors. *J. Agric. Food Chem.* **54**, 9135-9139 (2006).
25. Nigam, S. *et al.* Synthesis of novel pyrazole derivatives from diaryl 1,3-diketones. Part I. *Heterocycl. Commun.* **9**, 405-410 (2003).
26. Roush, D. M. *et al.* Preparation of aryl 2-fluoroethyl ethers as pesticides. US 4960884 A (1990).
27. Saxty, G. *et al.* Bicyclic heterocyclic compounds as selective protein tyrosine kinase inhibitors, their preparation and use in treating cancer and other diseases mediated by FGFR kinase. *PCT Int. Appl.* WO 2009047506 A1 (2009).
28. Heller, S. T.; Natarajan, S. R. 1,3-Diketones from acid chlorides and ketones: a rapid and general one-pot synthesis of pyrazoles. *Org. Lett.* **8**, 2675-2678 (2006).

29. Zhong, Q. *et al.* Organotellurium ylide reactions. Part I. Synthesis of α, β -unsaturated ketones via telluronium ylides. *Youji Huaxue* **10**, 459-463 (1990).
30. Mahmoodi, N. O. *et al.* Synthesis and photochromic properties of new heterocyclic derivatives of 1,3-diazabicyclo[3.1.0]hex-3-ene. *J. Chin. Chem. Soc. (Taipei, Taiwan)* **54**, 635-641 (2007).
31. Okunrobo, L. O. *et al.* Anti-inflammatory and gastroprotective properties of some chalcones. *Acta Pol. Pharm.* **63**, 195-199 (2006).
32. Weber, F. G. Preparation, N.M.R. and IR spectra of 2,3-dibromo- and 2-iodo-3-chloro-1,3-diphenyl-1-propanones. *Tetrahedron* **25**, 4283-4289 (1969).
33. Popat, K. H. *et al.* Synthesis of some new cyanopyrans and cyanopyridines and their biological activities. *J. Ind. Chem. Soc.* **81**, 157-159 (2004).
34. Popat, K. H. *et al.* Synthesis and biological activities of some quinoxaline derivatives bearing aromatic halogen nucleus. *J. Ind. Chem. Soc.* **80**, 709-710 (2003).
35. Nimavat, K. S. *et al.* 2-(3-Bromophenyl)-3-(substituted benzyl)quinoxalines as anticancer, antitubercular and antimicrobial agents. *J. Inst. Chem. (India)* **74**, 171-174 (2002).
36. Cunningham, B. D. M. *et al.* Structural studies on bioactive compounds. Part 12. Tautomerism and conformation of aryl-substituted 1-(2-hydroxyphenyl)-3-phenylpropane-1,3-diones in the solid phase and in solution. *J. Chem. Soc., Perkin Trans. 2*, 1275-1283 (1989).
37. Beutner, S. *et al.* Carotenylflavonoids, a novel group of potent, dual-functional antioxidants. *ARKIVOC (Gainesville, FL, U. S.)*, 279-295 (2007).
38. Sharma, T. C. *et al.* Oxidation of some hydroxyarylpyrazolines with manganese dioxide. *Acta Chim. Acad. Sci. Hung.* **93**, 415-419 (1977).
39. FERLUX. Pharmacologically active 5-(o-hydroxyphenyl)pyrazoles. FR 2104932 A1 (1972).
40. Kashima, C. *et al.* The preparation of *N*-acylpyrazoles and their behavior toward alcohols. *Synthesis*, 61-65 (1994).
41. Zhao, Z.-G.; Wang, Z.-X. Halogenation of pyrazoles using *N*-halosuccinimides in CCl_4 and in Water. *Synth. Commun.* **37**, 137-147 (2007).
42. Silva, V. D. *et al.* Efficient chemoselective biohydrogenation of 1,3-diaryl-2-propen-1-ones catalyzed by Saccharomyces cerevisiae yeasts in biphasic system. *J. Mol. Catal. B: Enzym.* **63**, 157-163 (2010).
43. Larkin, J. *et al.* Reactions of β -diketone with hydroxylamine hydrochloride; synthesis of 3,5-disubstituted isoxazoles. *J. Chem. Soc. C* **7**, 947-949 (1970).
44. Shettigar, V. *et al.* Crystal growth and characterization of new nonlinear optical chalcone derivative: 1-(4-Methoxyphenyl)-3-(3,4-dimethoxyphenyl)-2-propen-1-one. *J. Cryst. Growth* **295**, 44-49 (2006).
45. Zicmanis, A. *et al.* Alkoxydibenzoylmethanes. *Latv. PSR Zinat. Akad. Vestis, Kim. Ser.* **2**, 229-234 (1974).
46. Korbonits, D. *et al.* Ring transformation of 3-(2-aminoaryl)-1,2,4-oxadiazoles into 3-acylaminoindazoles; extension of the Boulton–Katritzky scheme. *J. Chem. Soc., Perkin Trans. 1*, 759-766 (1982).
47. Dallacker, F. Preparation of 1,2,4,5-tetrazines from pyridine and methylenedioxybenzene. *Monatsh. Chem.* **91**, 294-304 (1960).
48. Li, B. *et al.* An optimized process for formation of 2,4-disubstituted imidazoles from condensation of amidines and α -haloketones. *Org. Process Res. Dev.* **6**, 682-683 (2002).
49. Naruse, S. *et al.* Novel imidazole compounds as surface treatment agents used in manufacture of printed circuit boards. *Jpn. Kokai Tokkyo Koho* (2010).
50. Chimenti, F. *et al.* Synthesis and delective inhibitory activity of 1-Acetyl-3,5-diphenyl-4,5-dihydro-(1*H*)-pyrazole derivatives against Monoamine Oxidase. *J. Med. Chem.* **47**, 2071-2074 (2004).
51. Yamakoshi, H. *et al.* Structure-activity relationship of C5-curcuminoids and synthesis of their molecular probes thereof. *Bioorg. Med. Chem.* **18**, 1083-1092 (2010).
52. Palaska, E. *et al.* Synthesis and antidepressant activities of some 3,5-diphenyl-2-pyrazolines. *Eur. J. Med. Chem.* **36**, 539-543 (2001).
53. Sogawa, S. *et al.* 3,4-Dihydroxychalcones as potent 5-lipoxygenase and cyclooxygenase inhibitors. *J. Med. Chem.* **36**, 3904-3909 (1993).
54. Hall, M. J. *et al.* A modular synthesis of unsymmetrical tetraarylazadipyrromethenes. *J. Org. Chem.* **70**, 5571-5578 (2005).
55. Lygo, B.; Wainwright, P. G. Phase-transfer catalysed asymmetric epoxidation of enones using *N*-Anthracenylmethyl-substituted cinchona alkaloids. *Tetrahedron* **55**, 6289-6300 (1999).
56. Sabate-Alduy, C. *et al.* Synthesis of 2-pyrazolin-4-ones (4-hydroxypyrazoles). *Bull. Soc. Chim. Fr. (Chim. Mol.)*, 1841-1842 (1976).

anle127B

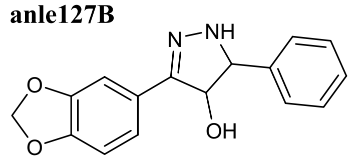
N
NH
O
O
OH

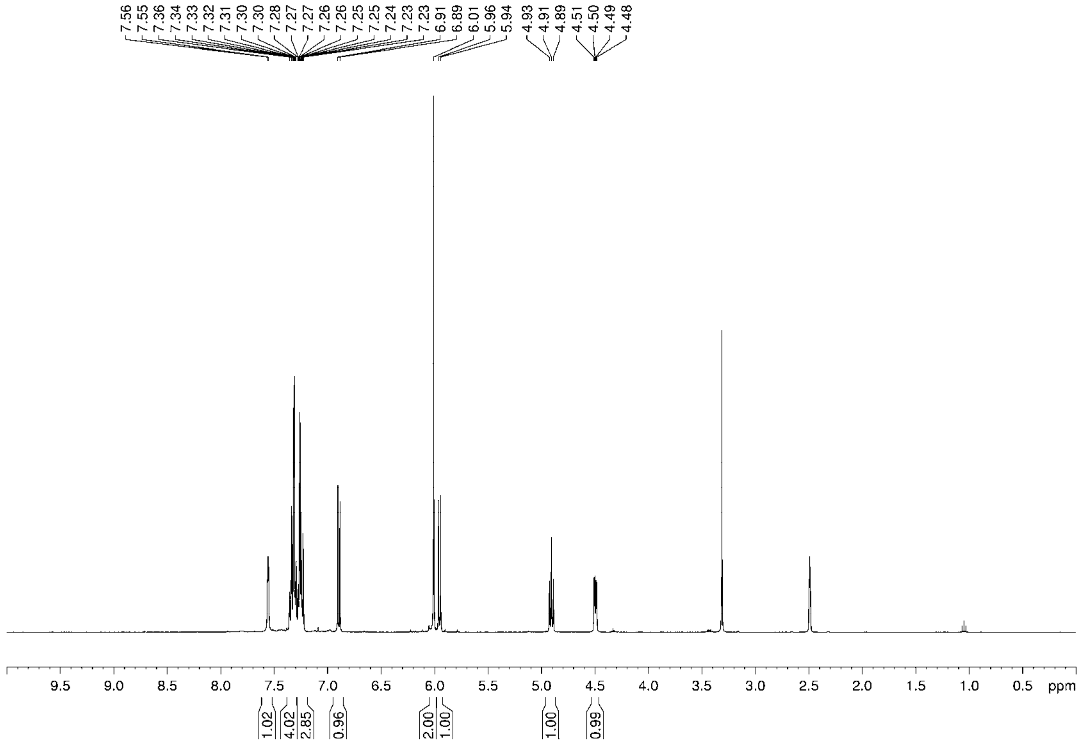
7.56
7.55
7.36
7.34
7.33
7.32
7.31
7.30
7.28
7.27
7.27
7.26
7.26
7.25
7.25
7.24
7.23
7.23
6.91
6.89
6.01
5.96
5.94
4.93
4.91
4.89
4.51
4.50
4.49
4.48
9.5 9.0 8.5 8.0 7.5 7.0 6.5 6.0 5.5 5.0 4.5 4.0 3.5 3.0 2.5 2.0 1.5 1.0 0.5 ppm
1.02
4.02
2.85
0.96
2.00
1.00
1.00
0.99

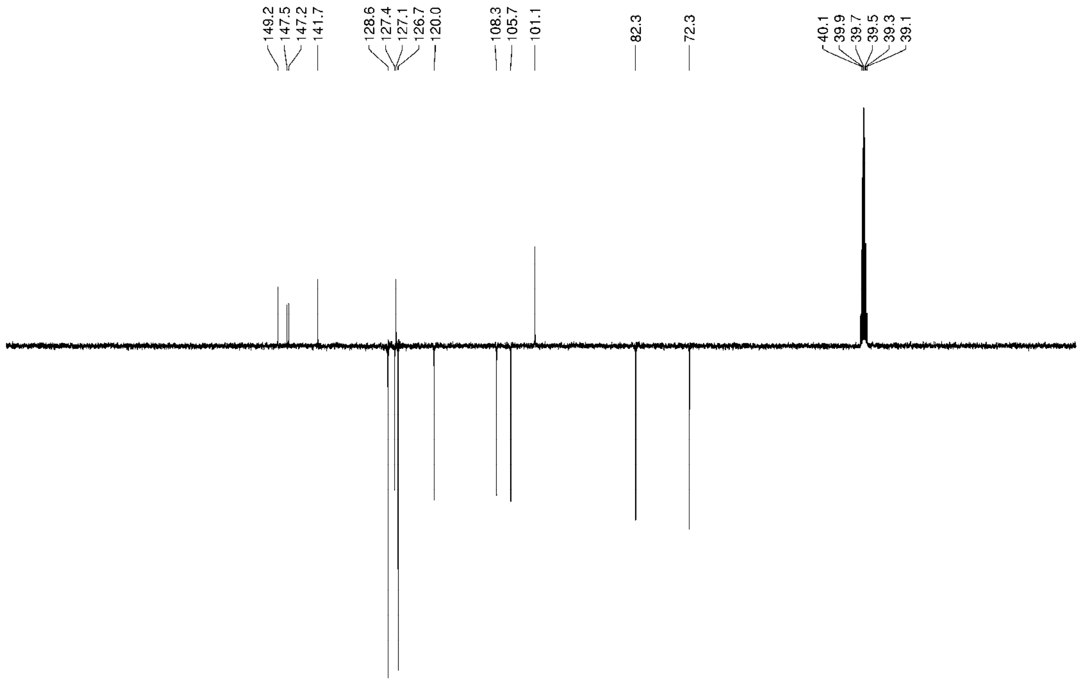
149.2
147.5
147.2
141.7
128.6
127.4
127.1
126.7
120.0
108.3
105.7
101.1
82.3
72.3
40.1
39.9
39.7
39.5
39.3
39.1
190 180 170 160 150 140 130 120 110 100 90 80 70 60 50 40 30 20 10 ppm

anle127C

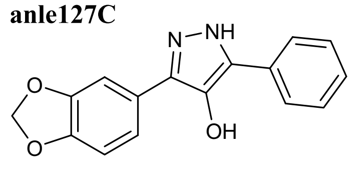
N–NH
OH

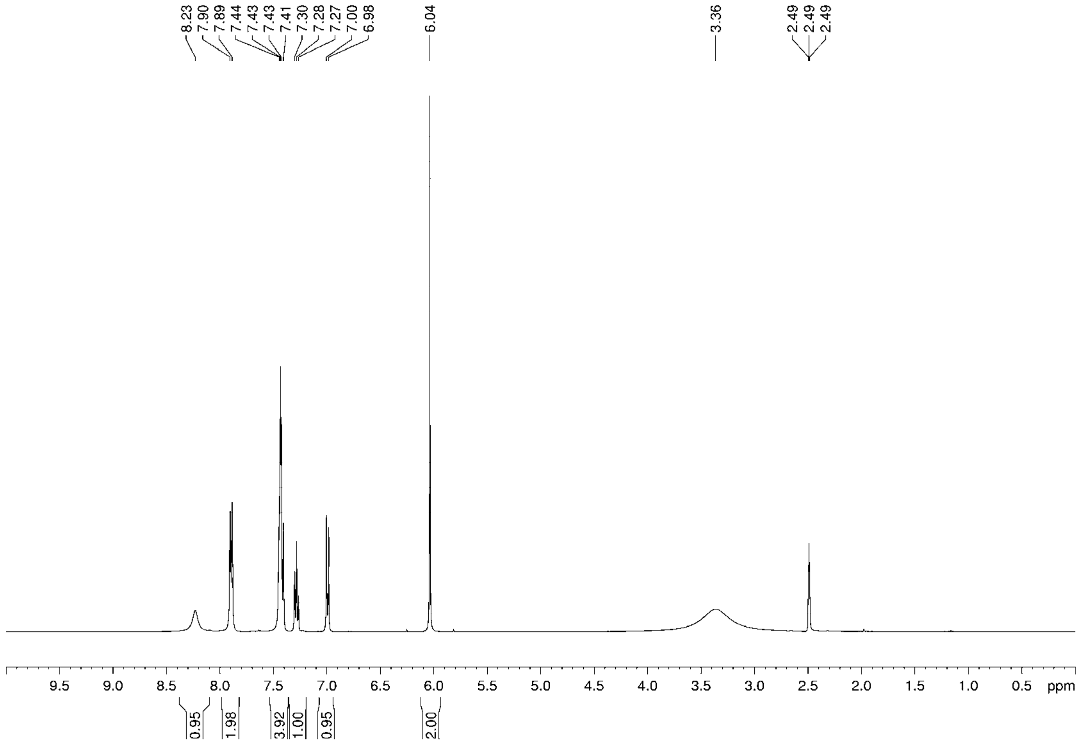
8.23
7.90
7.89
7.44
7.43
7.43
7.41
7.30
7.28
7.27
7.00
6.98
6.04
3.36
2.49
2.49
2.49
9.5 9.0 8.5 8.0 7.5 7.0 6.5 6.0 5.5 5.0 4.5 4.0 3.5 3.0 2.5 2.0 1.5 1.0 0.5 ppm
0.95
1.98
3.92
1.00
0.95
2.00

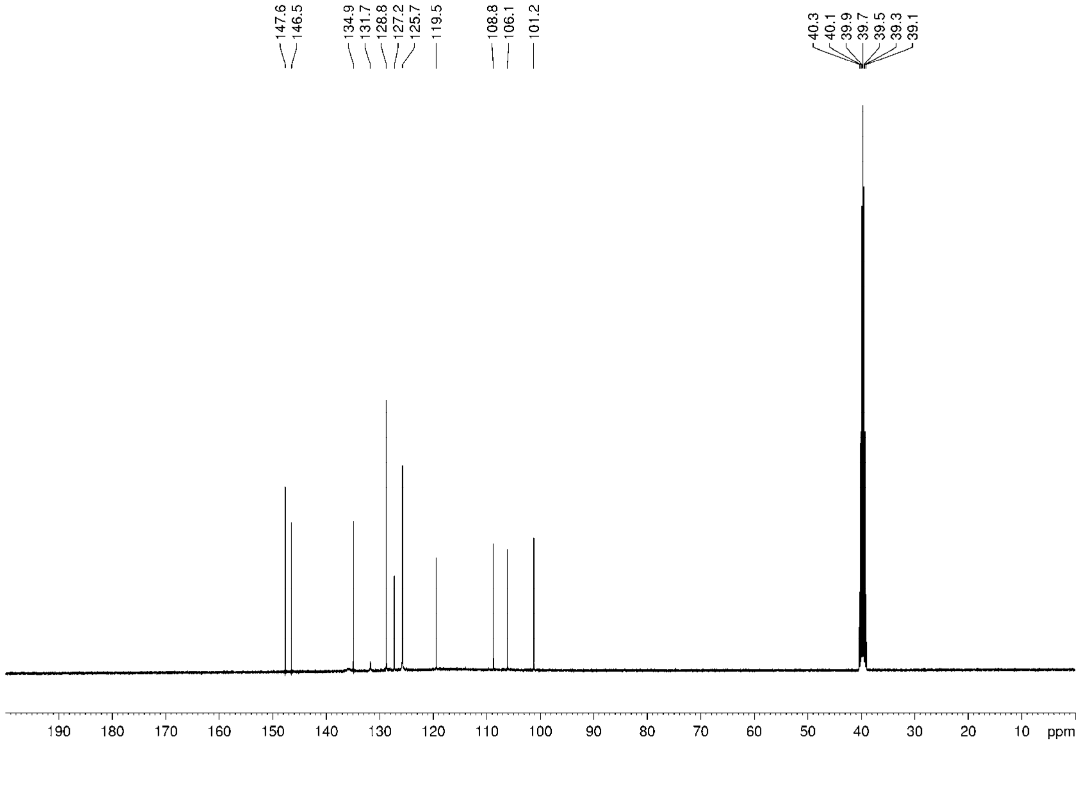
147.6
146.5
134.9
131.7
128.8
127.2
125.7
119.5
108.8
106.1
101.2
40.3
40.1
39.9
39.7
39.5
39.3
39.1
190 180 170 160 150 140 130 120 110 100 90 80 70 60 50 40 30 20 10 ppm

anle128B

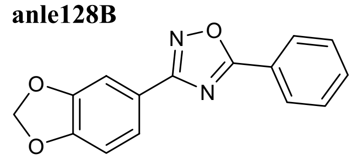

N
O
O
O
N

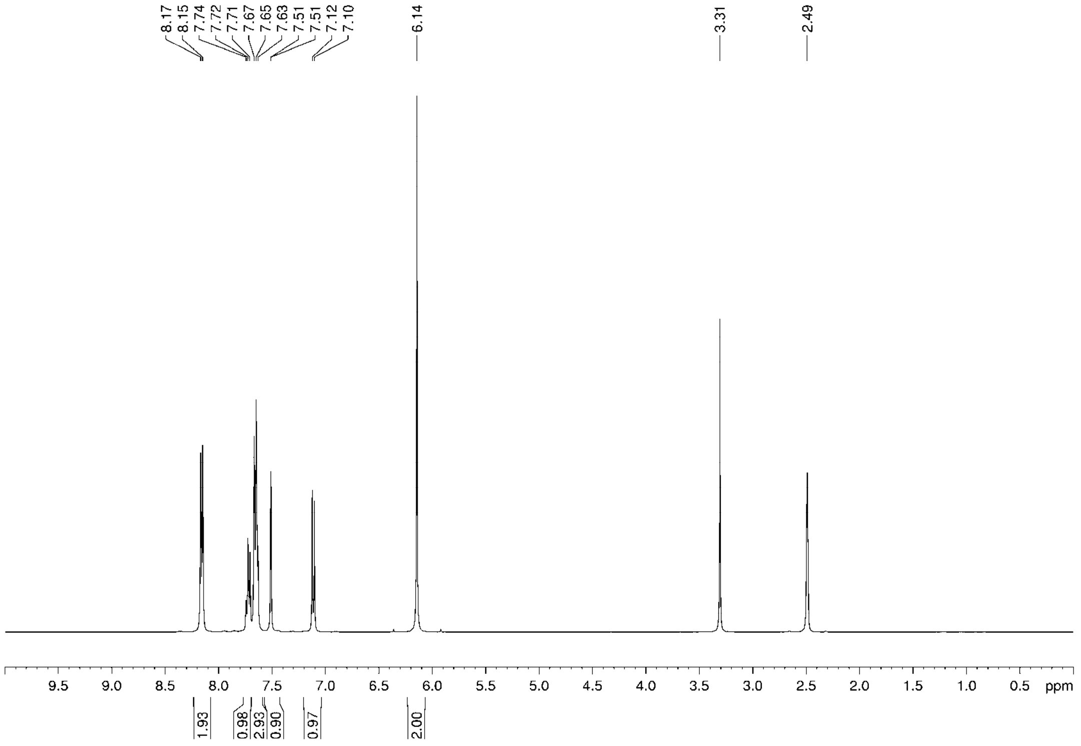

8.17
8.15
7.74
7.72
7.71
7.67
7.65
7.63
7.51
7.51
7.12
7.10
6.14
3.31
2.49
9.5 9.0 8.5 8.0 7.5 7.0 6.5 6.0 5.5 5.0 4.5 4.0 3.5 3.0 2.5 2.0 1.5 1.0 0.5 ppm
1.93
0.98
2.93
0.90
0.97
2.00

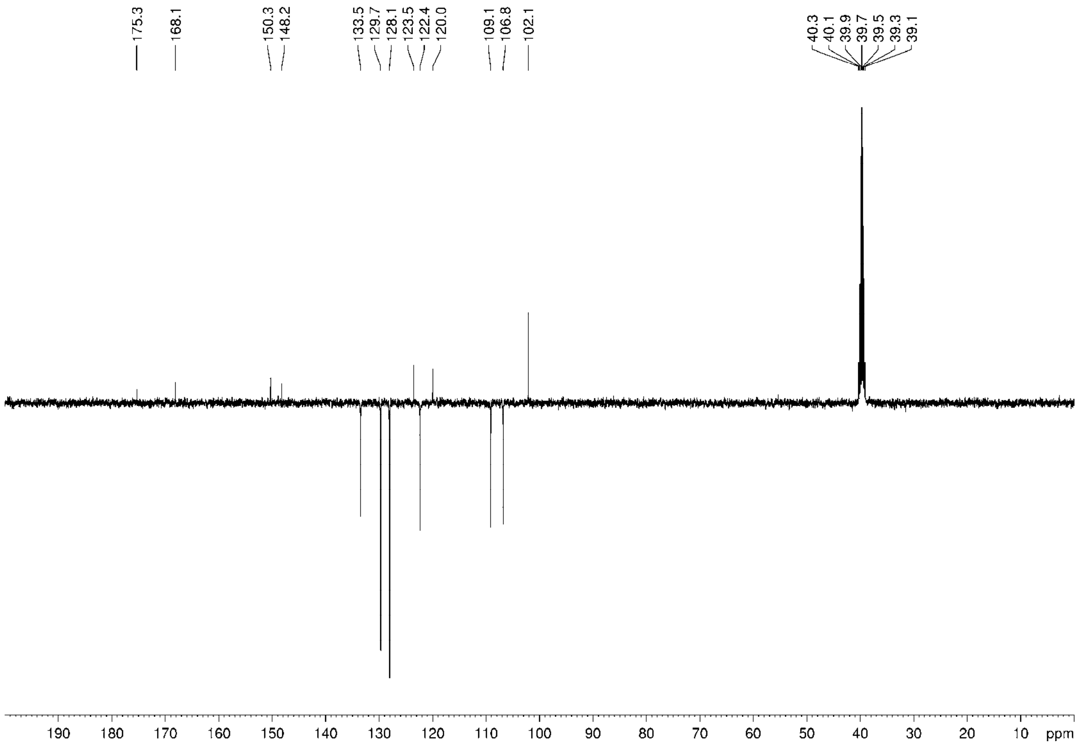

175.3
168.1
150.3
148.2
133.5
129.7
128.1
123.5
122.4
120.0
109.1
106.8
102.1
40.3
40.1
39.9
39.7
39.5
39.3
39.1
190 180 170 160 150 140 130 120 110 100 90 80 70 60 50 40 30 20 10 ppm

anle129

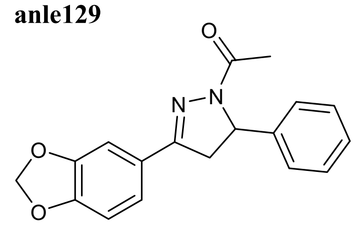
O
N
N
O
O

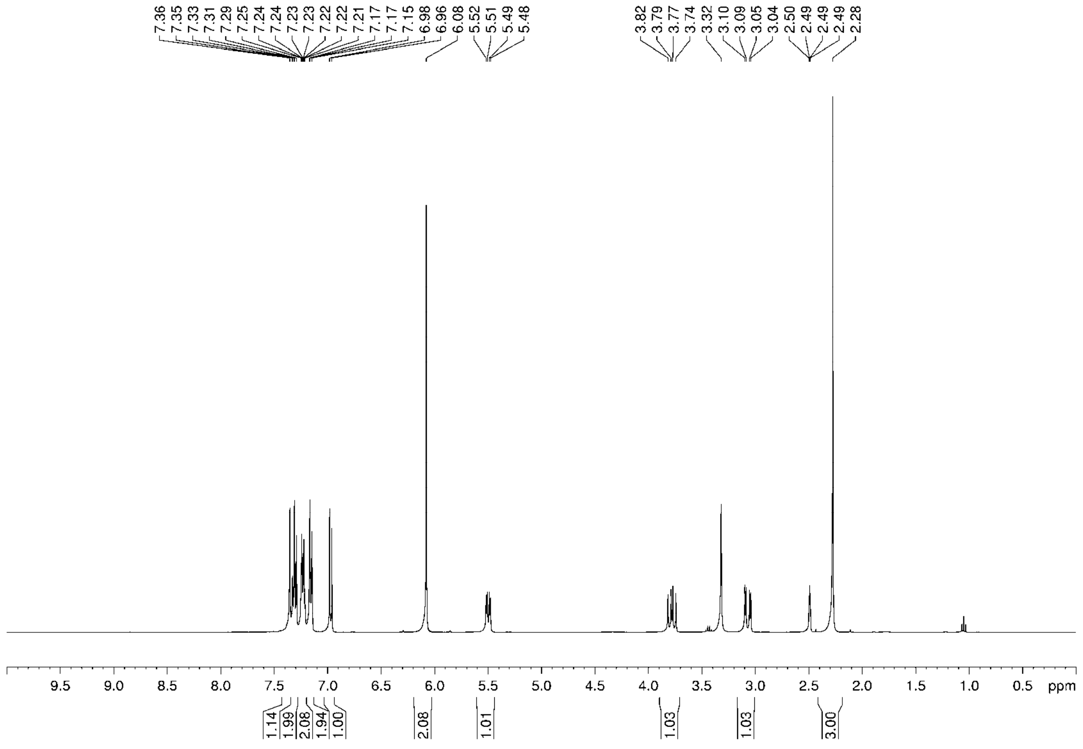
7.36 7.35 7.33 7.31 7.29 7.25 7.24 7.24 7.23 7.23 7.22 7.22 7.21 7.17 7.15 6.98 6.96 6.08 5.52 5.51 5.49 5.48
3.82 3.79 3.77 3.74 3.32 3.10 3.09 3.05 3.04 2.50 2.49 2.49 2.49 2.28
9.5 9.0 8.5 8.0 7.5 7.0 6.5 6.0 5.5 5.0 4.5 4.0 3.5 3.0 2.5 2.0 1.5 1.0 0.5 ppm
1.14 1.99 2.08 1.94 1.00 2.08 1.01 1.03 1.03 3.00

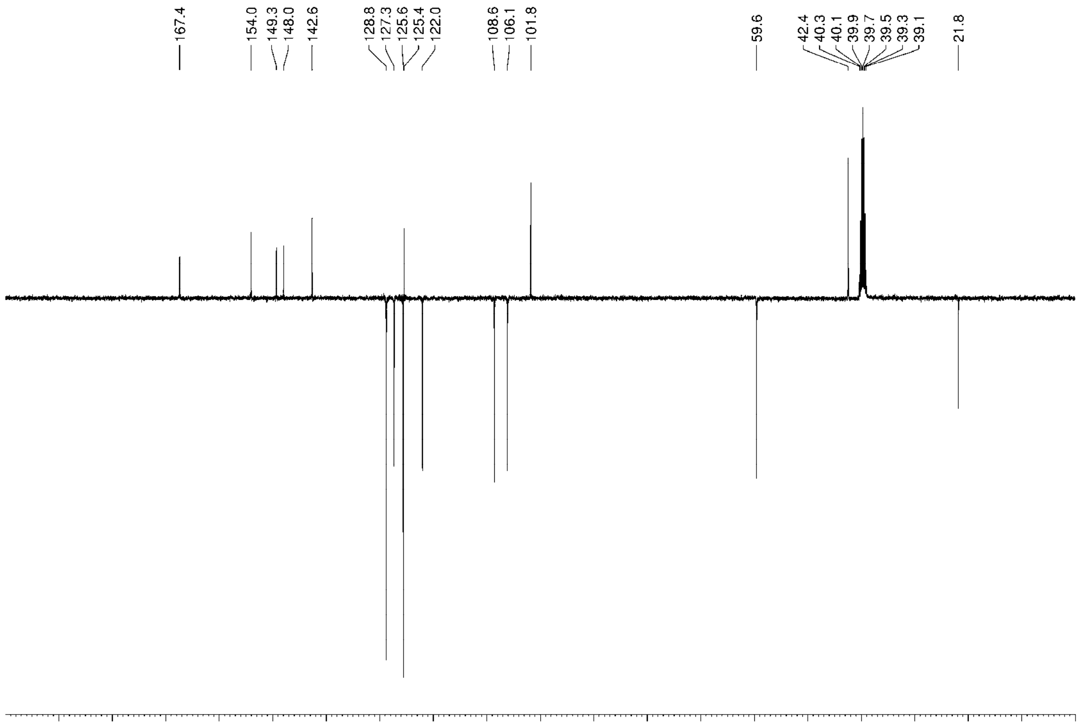
167.4 154.0 149.3 148.0 142.6 128.8 127.3 125.6 125.4 122.0 108.6 106.1 101.8
59.6 42.4 40.3 40.1 39.9 39.7 39.5 39.3 39.1 21.8
190 180 170 160 150 140 130 120 110 100 90 80 70 60 50 40 30 20 10 ppm

anle130A

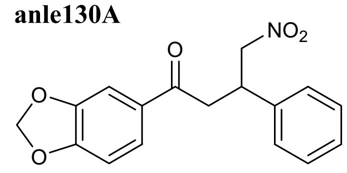

O
NO2
O
O

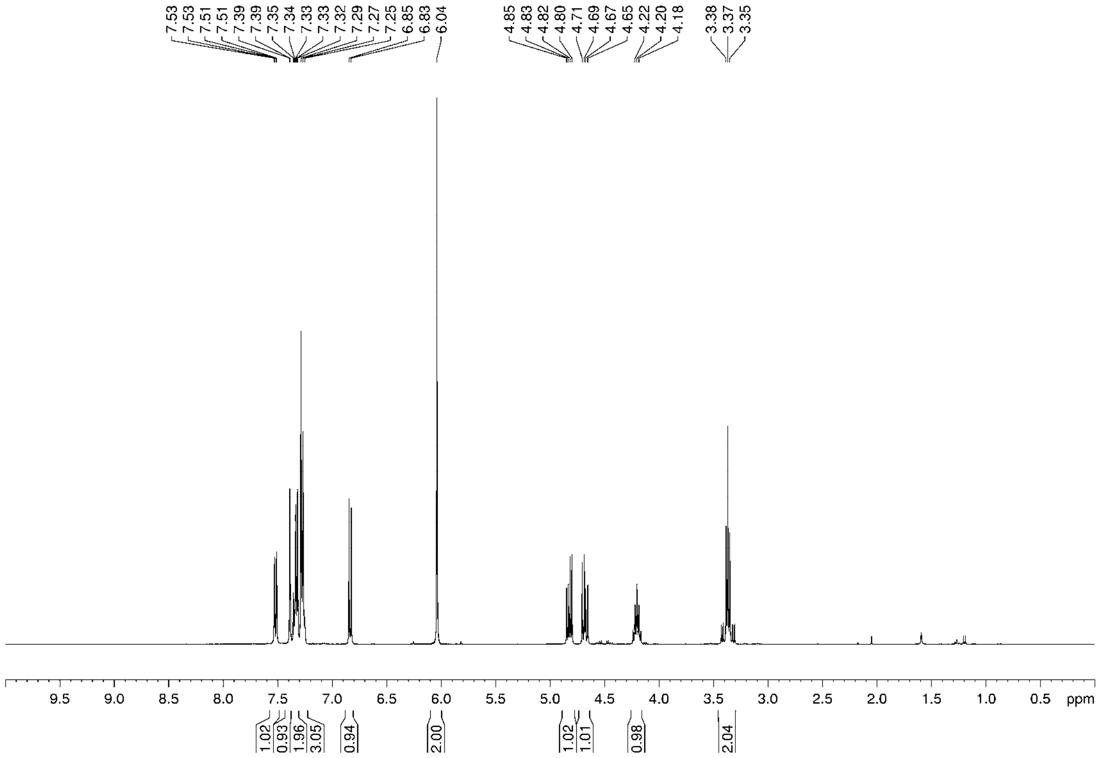

7.53 7.53 7.51 7.51 7.39 7.39 7.35 7.34 7.33 7.33 7.32 7.29 7.27 7.25 6.85 6.83 6.04
4.85 4.83 4.82 4.80 4.71 4.69 4.67 4.65 4.22 4.20 4.18
3.38 3.37 3.35
9.5 9.0 8.5 8.0 7.5 7.0 6.5 6.0 5.5 5.0 4.5 4.0 3.5 3.0 2.5 2.0 1.5 1.0 0.5 ppm
1.02 0.93 1.96 3.05 0.94 2.00 1.02 1.01 0.98 2.04

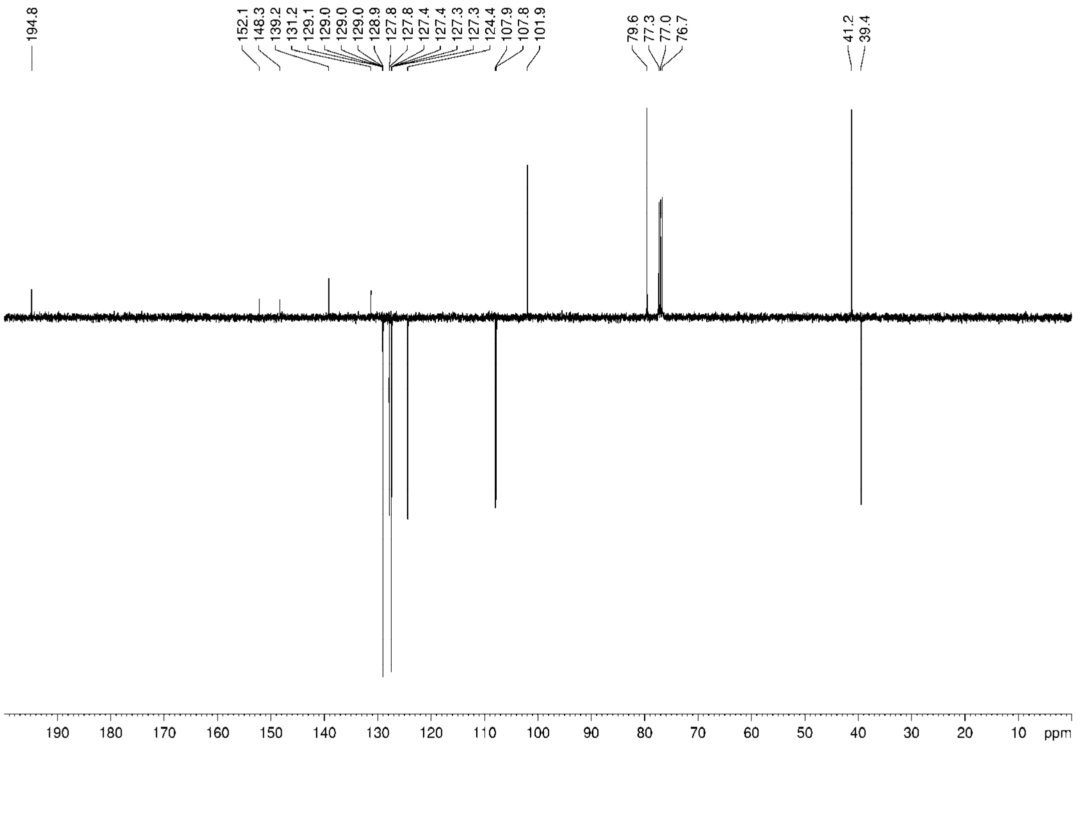

194.8
152.1 148.3 139.2 131.2 129.1 129.0 129.0 128.9 127.8 127.8 127.4 127.3 127.3 124.4 107.9 107.8 101.9
79.6 77.3 77.0 76.7
41.2 39.4
190 180 170 160 150 140 130 120 110 100 90 80 70 60 50 40 30 20 10 ppm

anle130B

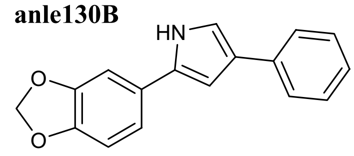
HN
O
O

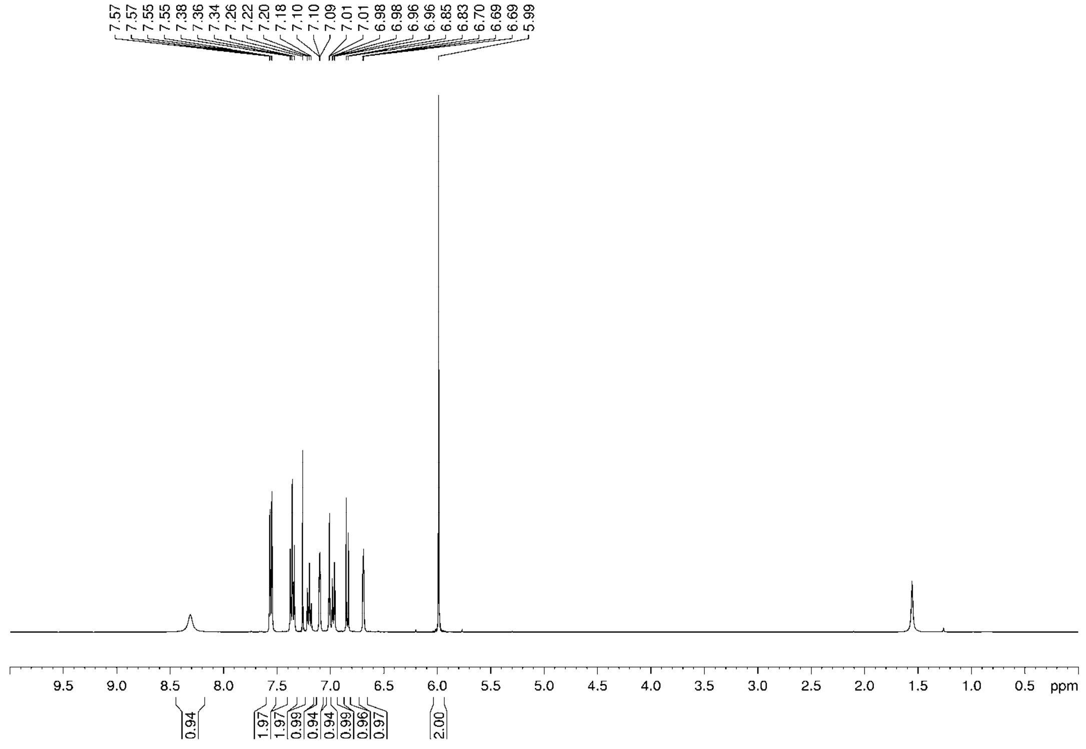
7.57
7.57
7.55
7.55
7.38
7.36
7.34
7.26
7.22
7.20
7.18
7.10
7.10
7.09
7.01
6.98
6.98
6.96
6.96
6.85
6.83
6.70
6.69
6.69
5.99
9.5
9.0
8.5
8.0
7.5
7.0
6.5
6.0
5.5
5.0
4.5
4.0
3.5
3.0
2.5
2.0
1.5
1.0
0.5
ppm
0.94
1.97
1.97
0.99
0.94
0.94
0.94
0.99
0.96
0.97
2.00

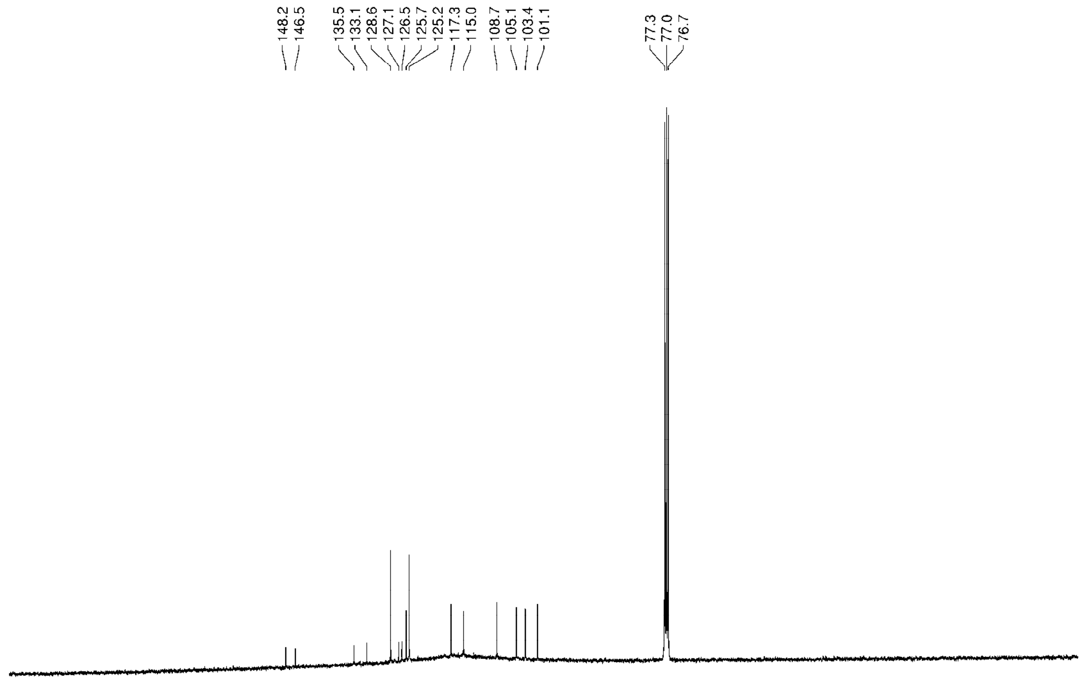
148.2
146.5
135.5
133.1
128.6
127.1
126.5
125.7
125.2
117.3
115.0
108.7
105.1
103.4
101.1
77.3
77.0
76.7
190
180
170
160
150
140
130
120
110
100
90
80
70
60
50
40
30
20
10
ppm

anle131B

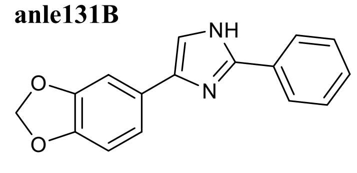

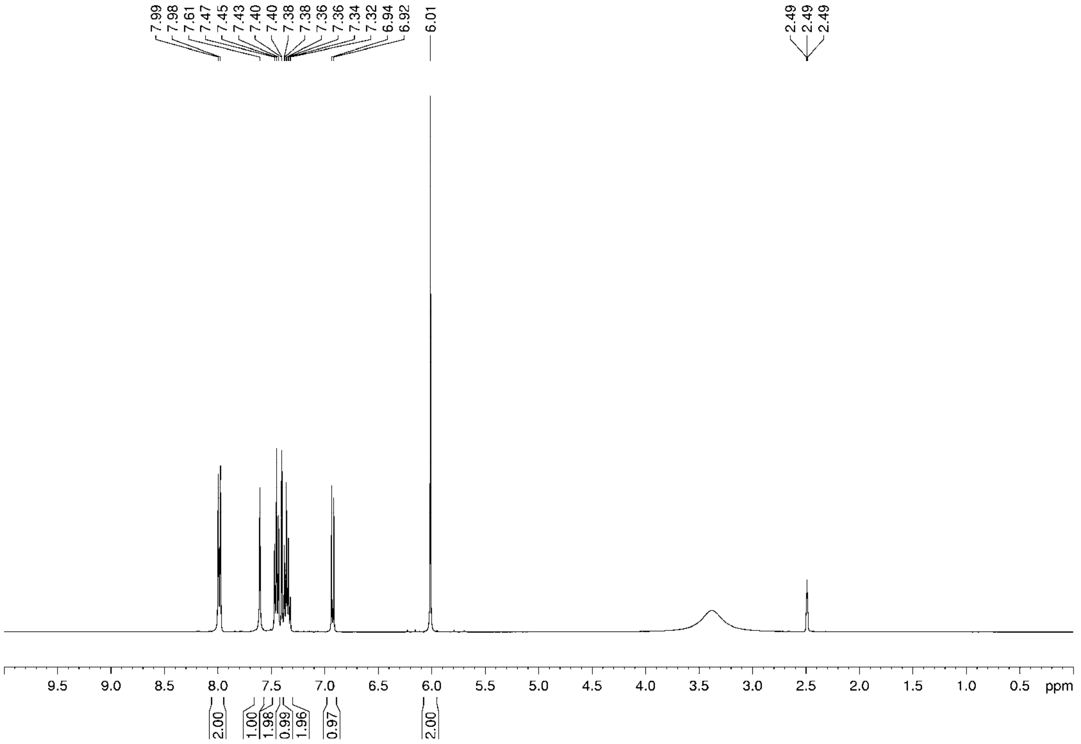

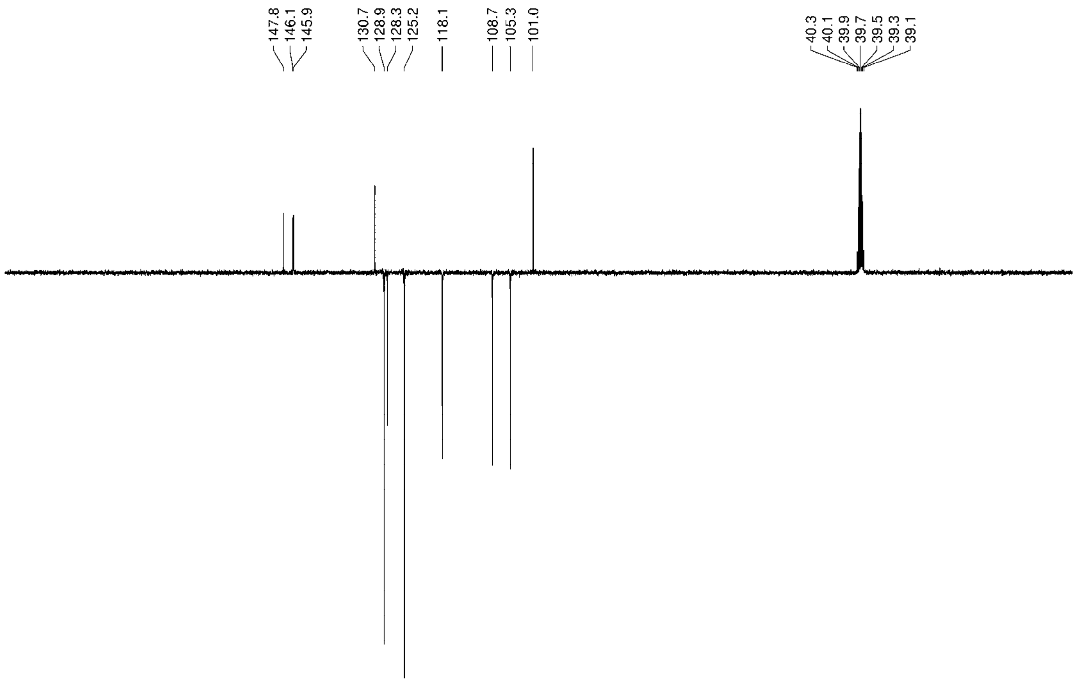

anle132

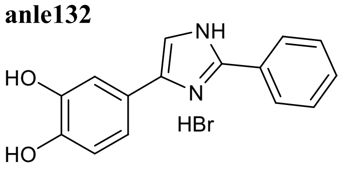
HO
HO
NH
N
HBr

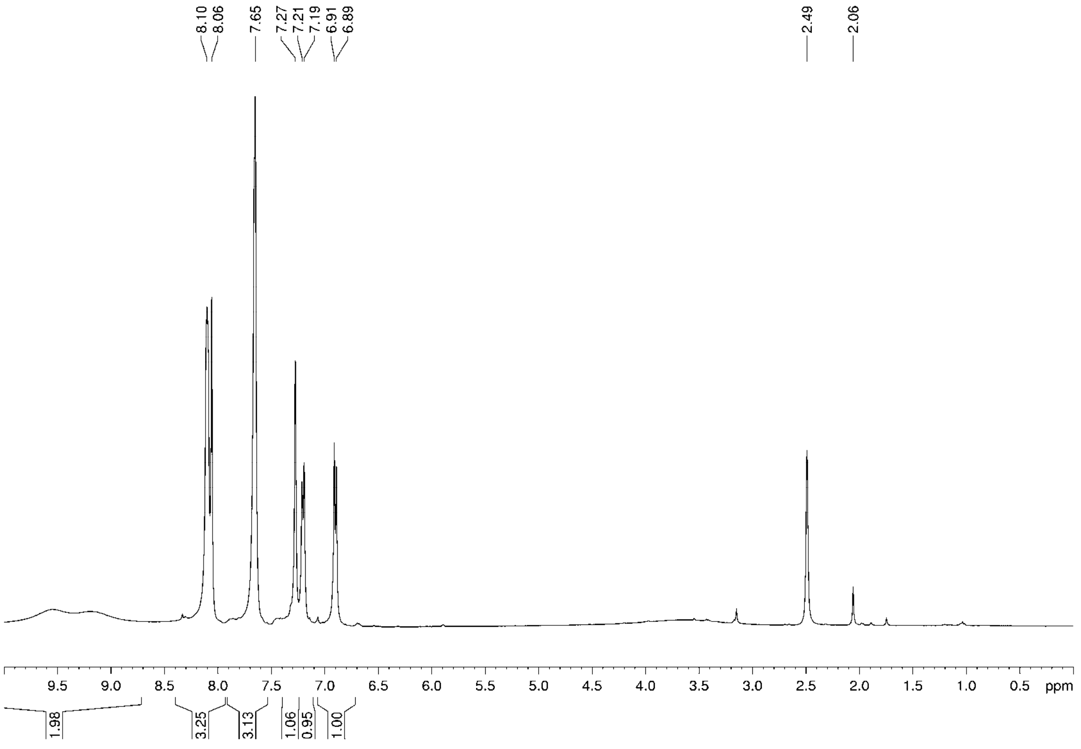
8.10
8.06
7.65
7.27
7.21
7.19
6.91
6.89
2.49
2.06
9.5 9.0 8.5 8.0 7.5 7.0 6.5 6.0 5.5 5.0 4.5 4.0 3.5 3.0 2.5 2.0 1.5 1.0 0.5 ppm
1.98
3.25
3.13
1.06
0.95
1.00

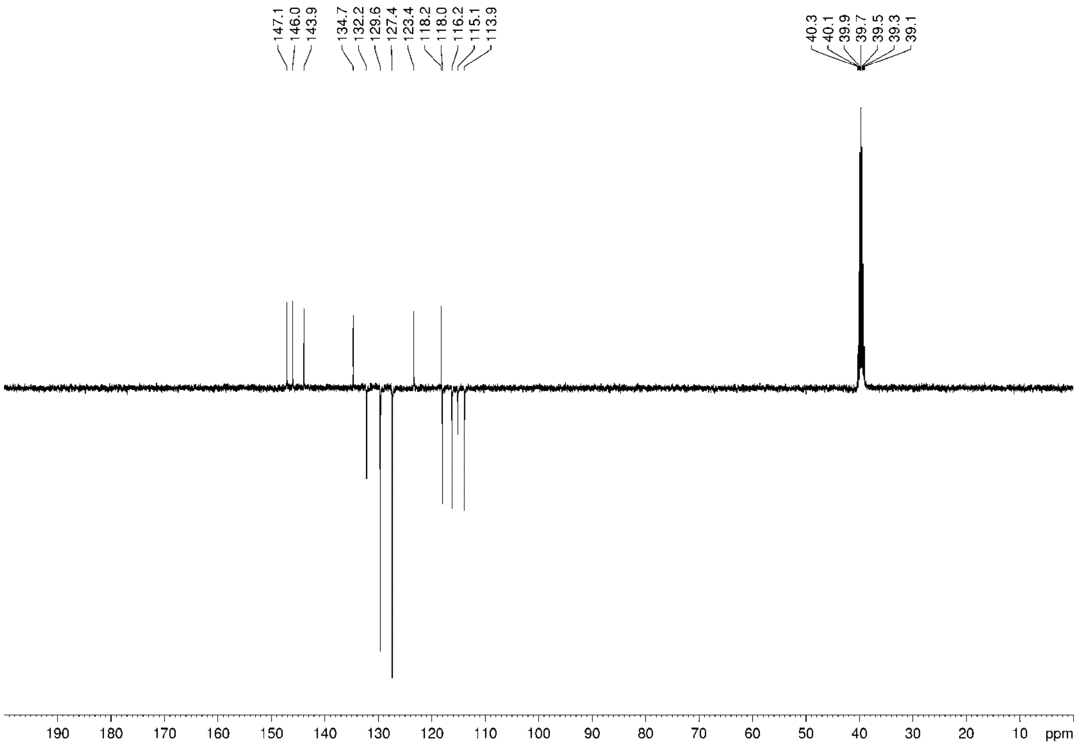
147.1
146.0
143.9
134.7
132.2
129.6
127.4
123.4
118.2
118.0
116.2
115.1
113.9
40.3
40.1
39.9
39.7
39.5
39.3
39.1
190 180 170 160 150 140 130 120 110 100 90 80 70 60 50 40 30 20 10 ppm

anle134

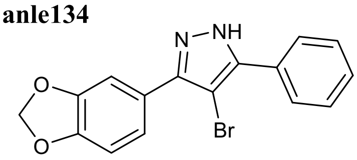
N–NH
O
O
Br

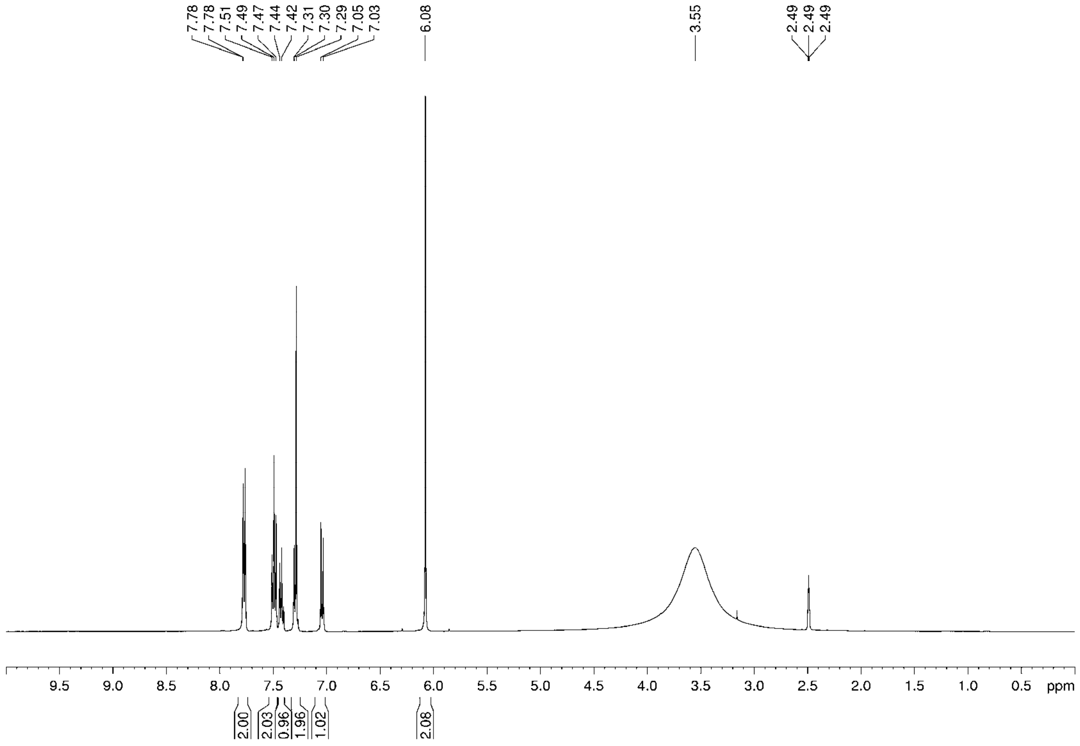
7.78
7.78
7.51
7.49
7.47
7.44
7.42
7.31
7.30
7.29
7.05
7.03
6.08
3.55
2.49
2.49
2.49
9.5 9.0 8.5 8.0 7.5 7.0 6.5 6.0 5.5 5.0 4.5 4.0 3.5 3.0 2.5 2.0 1.5 1.0 0.5 ppm
2.00
2.03
0.96
1.96
1.02
2.08

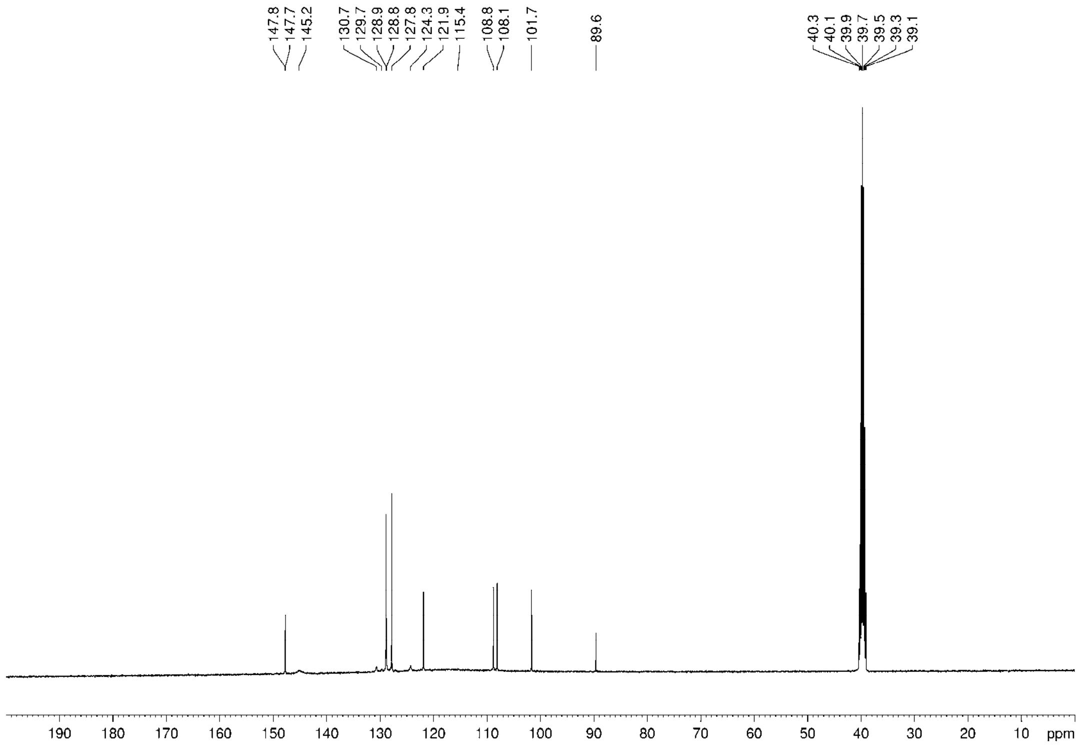
147.8
147.7
145.2
130.7
129.7
128.9
128.8
127.8
124.3
121.9
115.4
108.8
108.1
101.7
89.6
40.3
40.1
39.9
39.7
39.5
39.3
39.1
190 180 170 160 150 140 130 120 110 100 90 80 70 60 50 40 30 20 10 ppm

anle136A

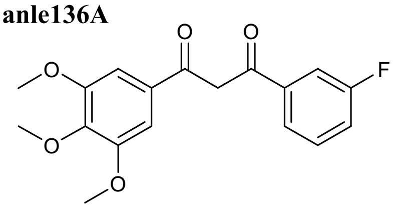
O
O
O
O
O
F

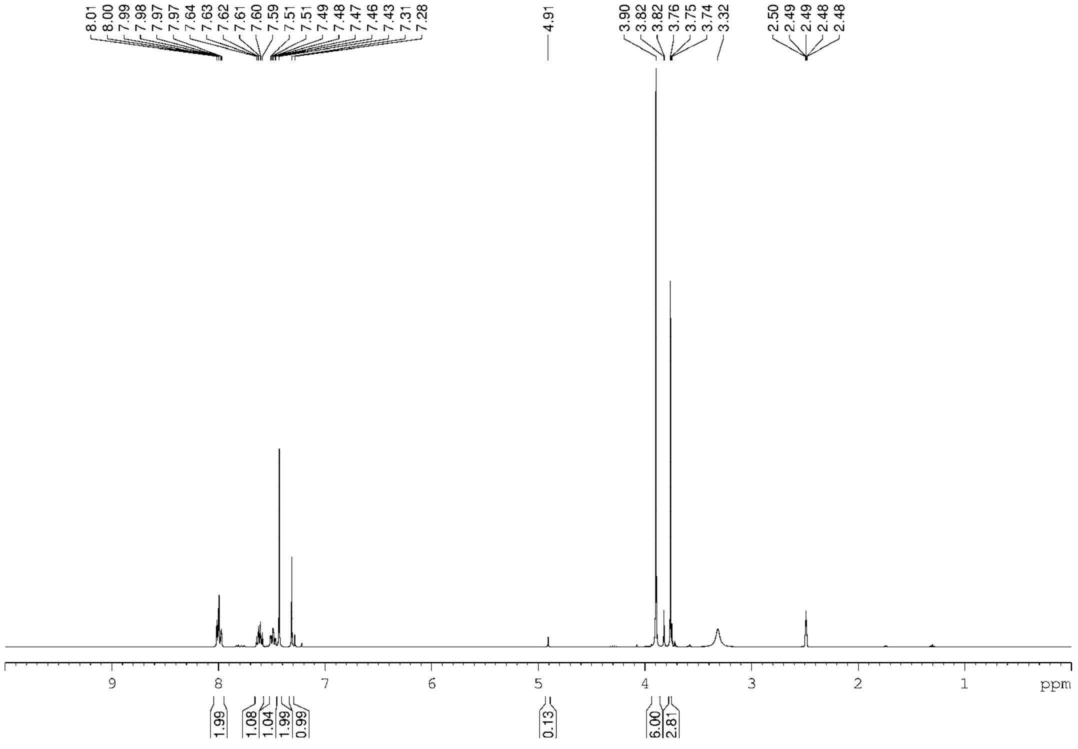
8.01
8.00
7.99
7.98
7.97
7.97
7.64
7.63
7.62
7.61
7.60
7.59
7.51
7.51
7.49
7.48
7.47
7.46
7.43
7.31
7.28
4.91
3.90
3.82
3.82
3.76
3.75
3.74
3.32
2.50
2.49
2.49
2.48
2.48
9
8
7
6
5
4
3
2
1
ppm
1.99
1.08
1.04
1.99
0.99
0.13
6.00
2.81

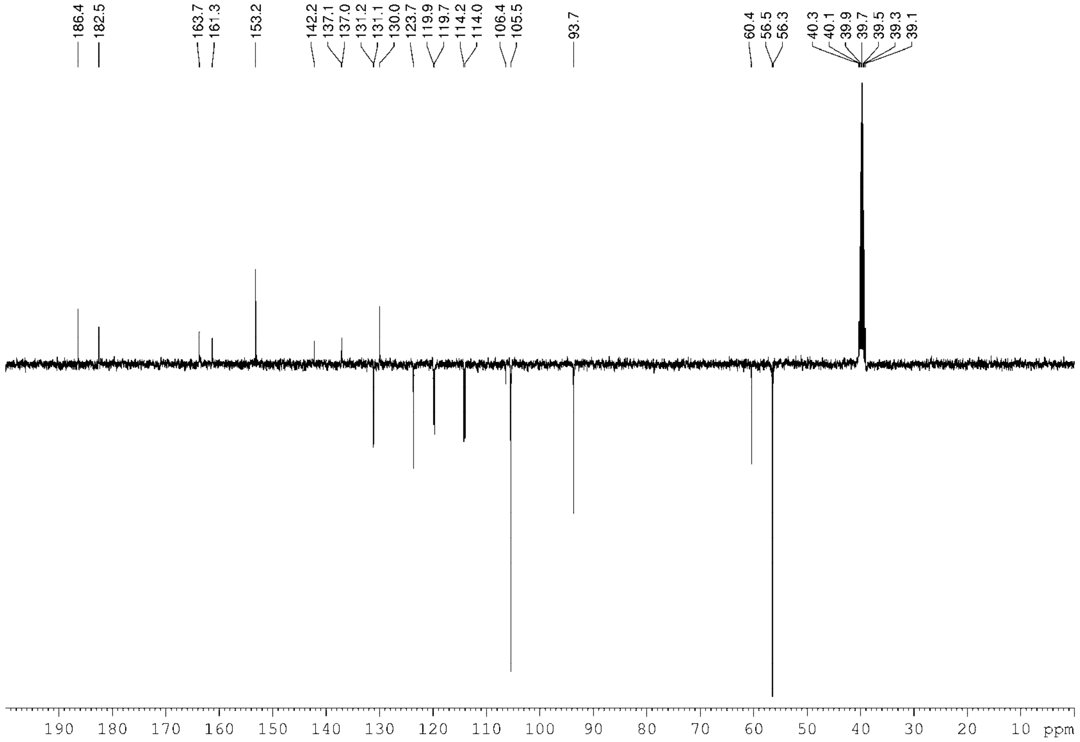
186.4
182.5
163.7
161.3
153.2
142.2
137.1
137.0
131.2
131.1
130.0
123.7
119.9
119.7
114.2
114.0
106.4
105.5
93.7
60.4
56.5
56.3
40.3
40.1
39.9
39.7
39.5
39.3
39.1
190
180
170
160
150
140
130
120
110
100
90
80
70
60
50
40
30
20
10 ppm

anle136B

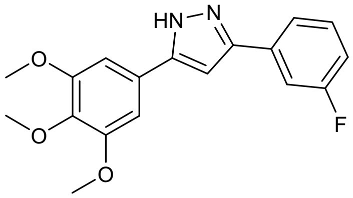
HN–N
O
O
O
F

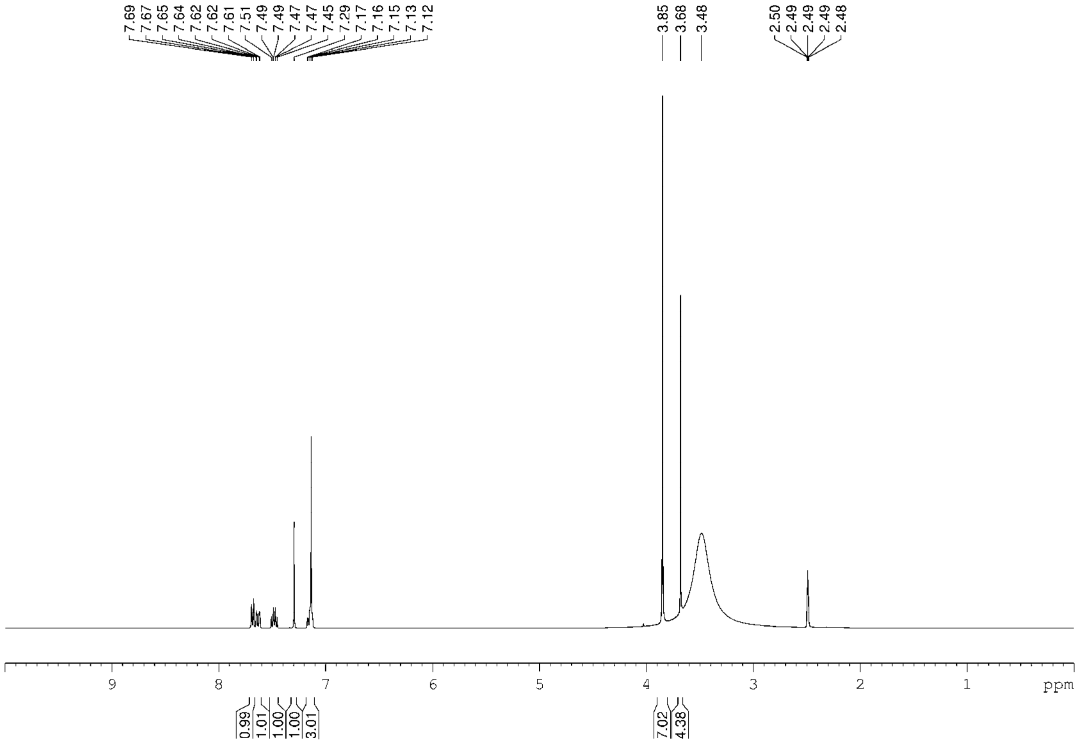
7.69 7.67 7.65 7.64 7.62 7.62 7.61 7.51 7.49 7.49 7.47 7.47 7.45 7.29 7.17 7.16 7.15 7.13 7.12
3.85 3.68 3.48
2.50 2.49 2.49 2.49 2.48
9 8 7 6 5 4 3 2 1 ppm
0.99 1.01 1.00 1.00 3.01
7.02 4.38

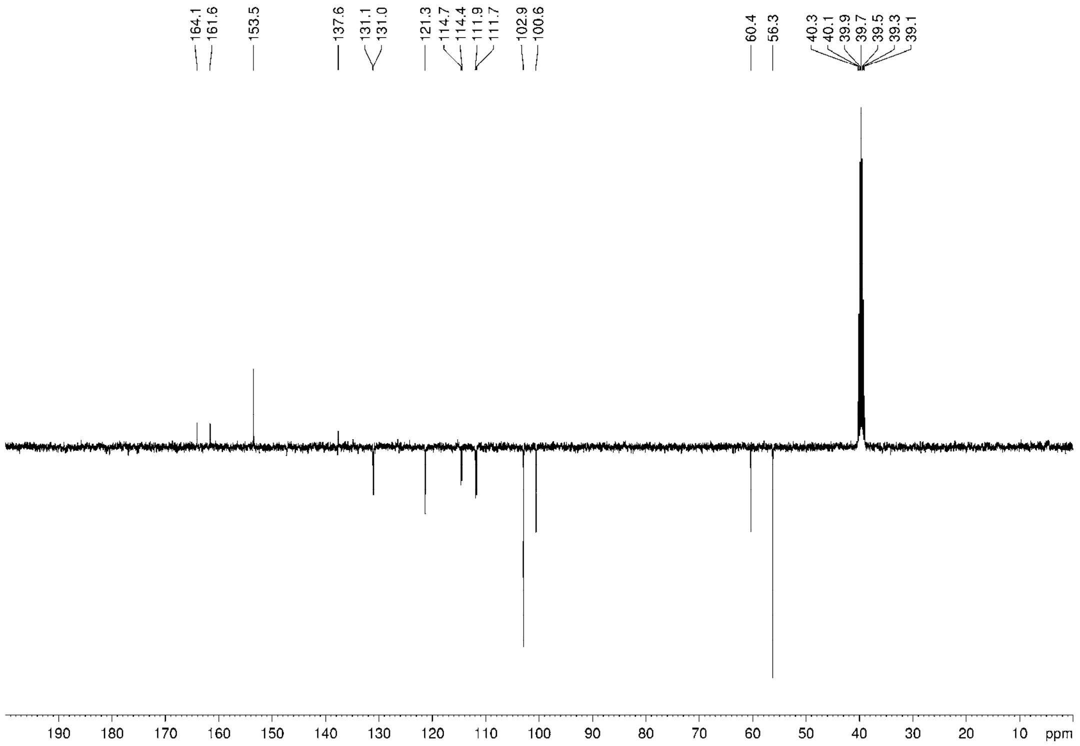
164.1 161.6 153.5 137.6 131.1 131.0 121.3 114.7 114.4 111.9 111.7 102.9 100.6
60.4 56.3 40.3 40.1 39.9 39.7 39.5 39.3 39.1
190 180 170 160 150 140 130 120 110 100 90 80 70 60 50 40 30 20 10 ppm

anle136C

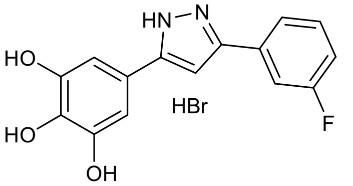
HN
N
HO
HO
OH
HBr
F

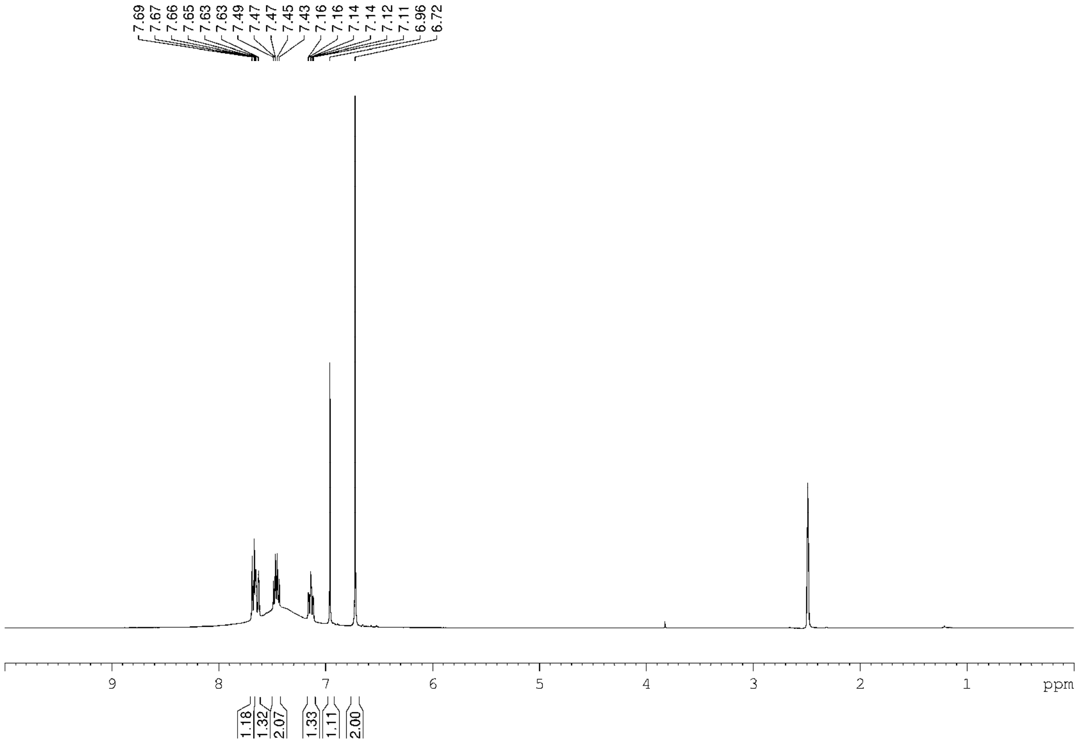
7.69
7.67
7.66
7.65
7.63
7.63
7.49
7.47
7.47
7.45
7.43
7.16
7.16
7.14
7.14
7.12
7.11
6.96
6.72
9
8
7
6
5
4
3
2
1
ppm
1.18
1.32
2.07
1.33
1.11
2.00

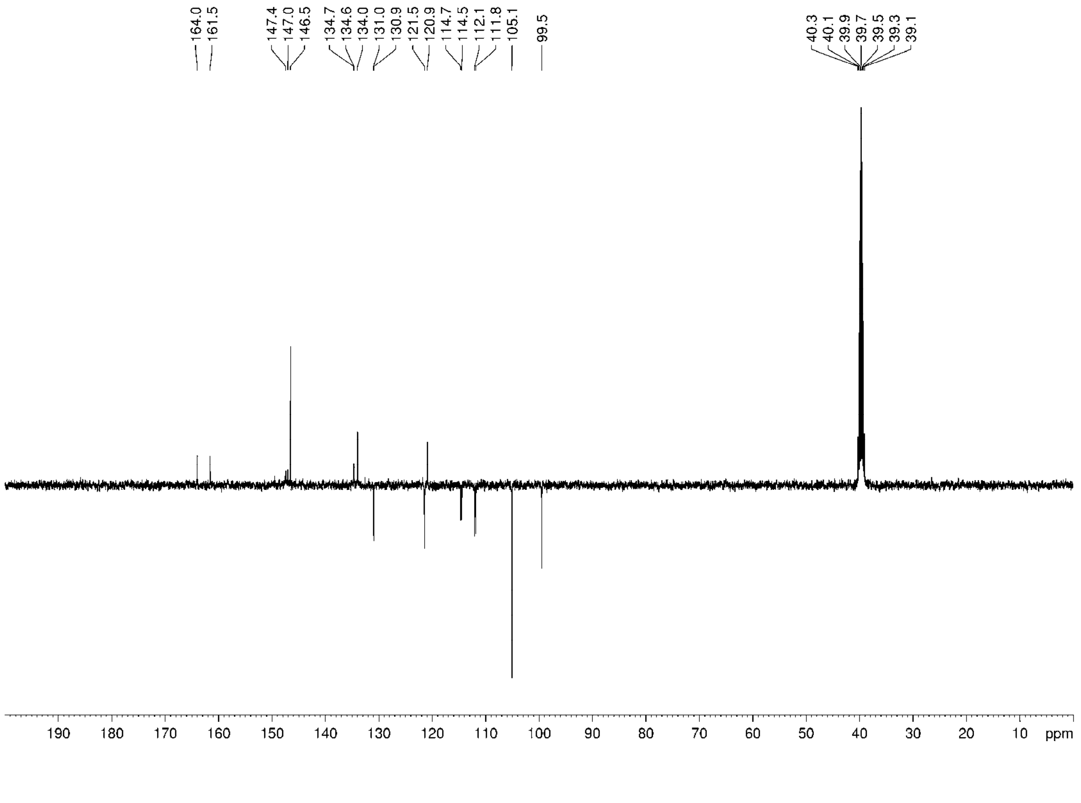
164.0
161.5
147.4
147.0
146.5
134.7
134.6
134.0
131.0
130.9
121.5
120.9
114.7
114.5
112.1
111.8
105.1
99.5
40.3
40.1
39.9
39.7
39.5
39.3
39.1
190
180
170
160
150
140
130
120
110
100
90
80
70
60
50
40
30
20
10
ppm

anle137A

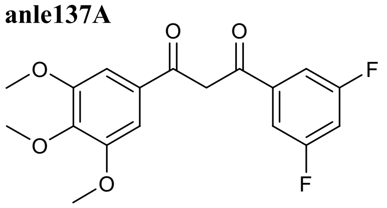
O
O
O
O
O
F
F

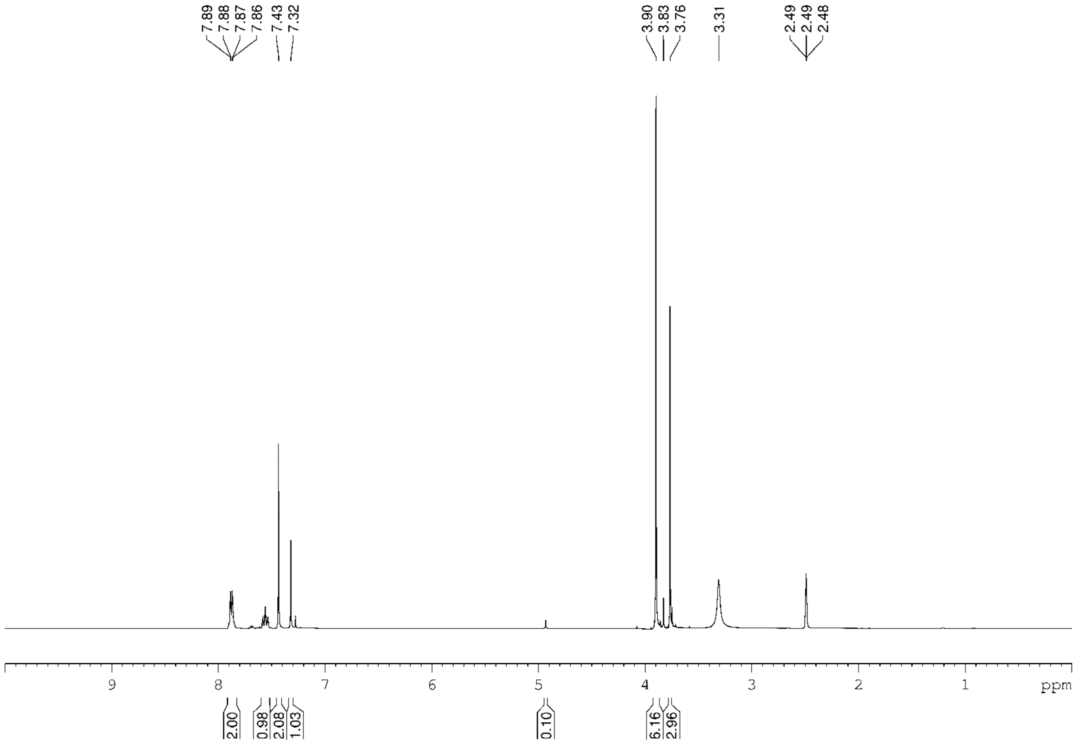
7.89
7.88
7.87
7.86
7.43
7.32
3.90
3.83
3.76
3.31
2.49
2.49
2.48
9
8
7
6
5
4
3
2
1
ppm
2.00
0.98
2.08
1.03
0.10
6.16
2.96

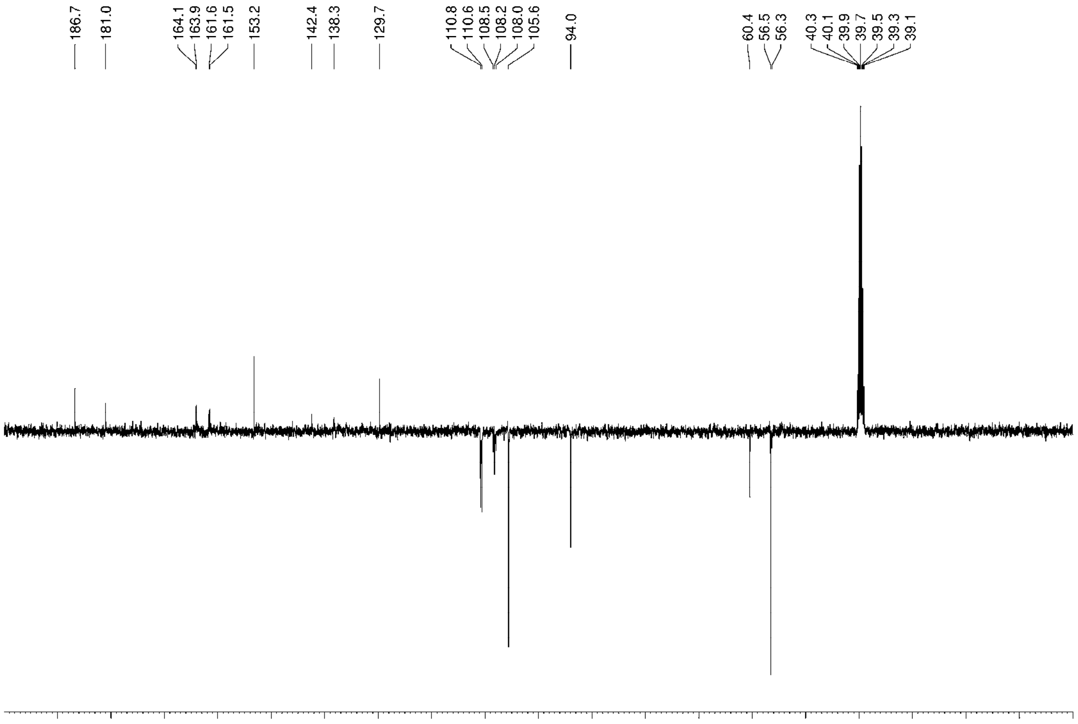
186.7
181.0
164.1
163.9
161.6
161.5
153.2
142.4
138.3
129.7
110.8
110.6
108.5
108.2
108.0
105.6
94.0
60.4
56.5
56.3
40.3
40.1
39.9
39.7
39.5
39.3
39.1
190
180
170
160
150
140
130
120
110
100
90
80
70
60
50
40
30
20
10
ppm

anle137B

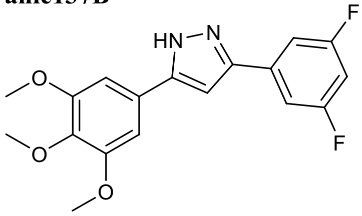
HN
N
F
F
O
O
O

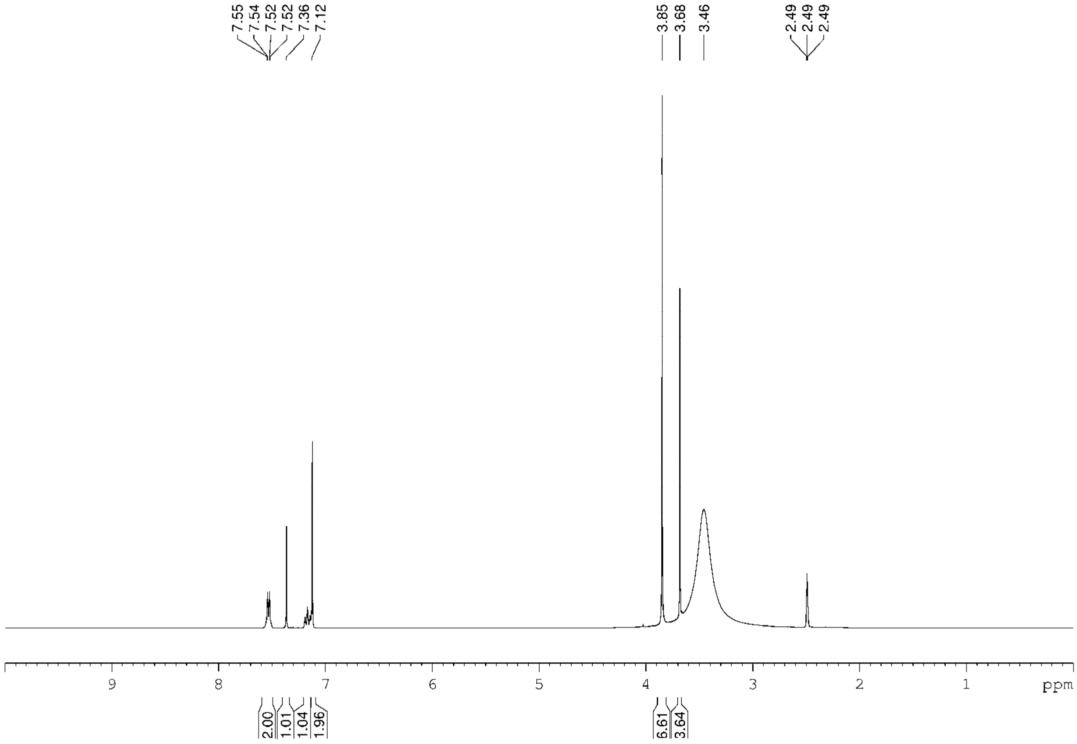
7.55
7.54
7.52
7.52
7.36
7.12
3.85
3.68
3.46
2.49
2.49
2.49
9
8
7
6
5
4
3
2
1
ppm
2.00
1.01
1.04
1.96
6.61
3.64

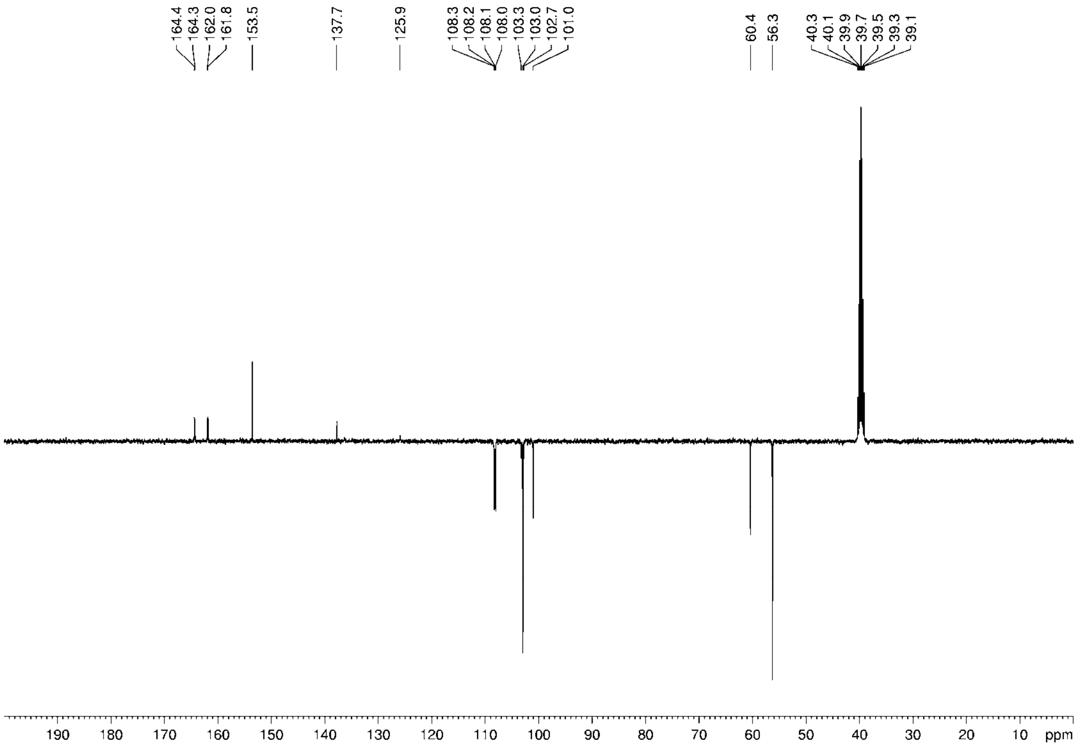
164.4
164.3
162.0
161.8
153.5
137.7
125.9
108.3
108.2
108.1
108.0
103.3
103.0
102.7
101.0
60.4
56.3
40.3
40.1
39.9
39.7
39.5
39.3
39.1
190
180
170
160
150
140
130
120
110
100
90
80
70
60
50
40
30
20
10
ppm

anle137C

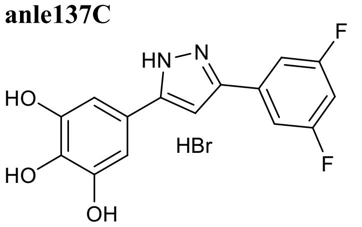

HN
N
F
F
HO
HO
OH
HBr

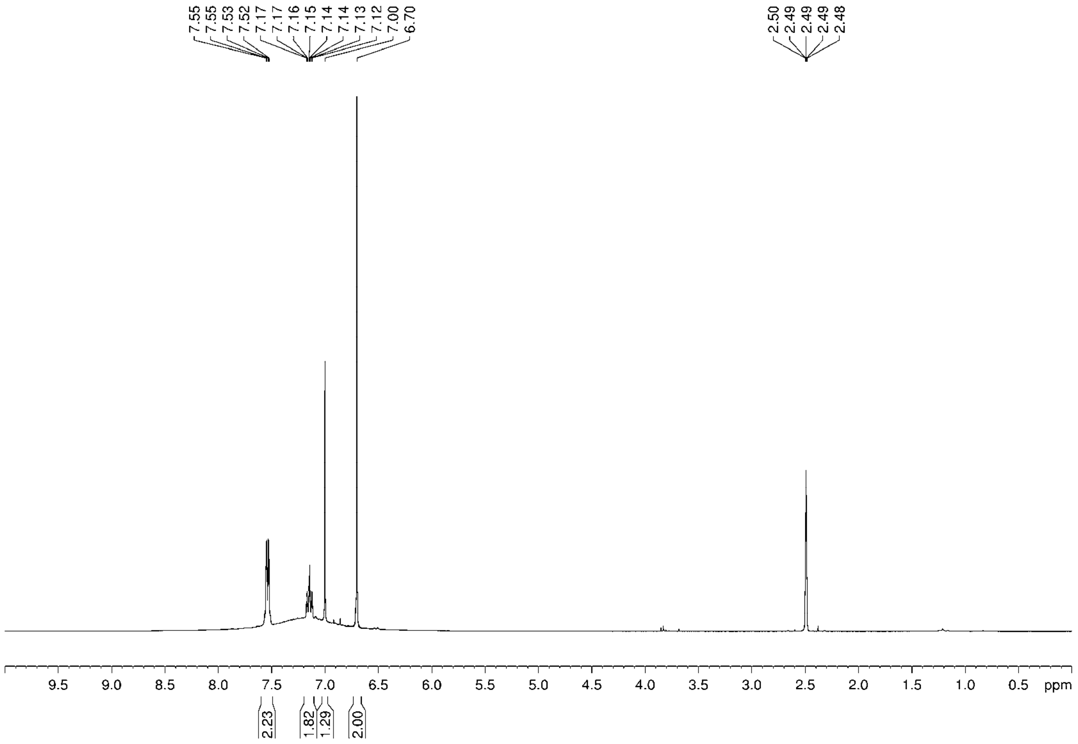

7.55 7.55 7.53 7.52 7.17 7.17 7.16 7.15 7.14 7.14 7.13 7.12 7.00 6.70
2.50 2.49 2.49 2.49 2.48
9.5 9.0 8.5 8.0 7.5 7.0 6.5 6.0 5.5 5.0 4.5 4.0 3.5 3.0 2.5 2.0 1.5 1.0 0.5 ppm
2.23 1.82 1.29 2.00

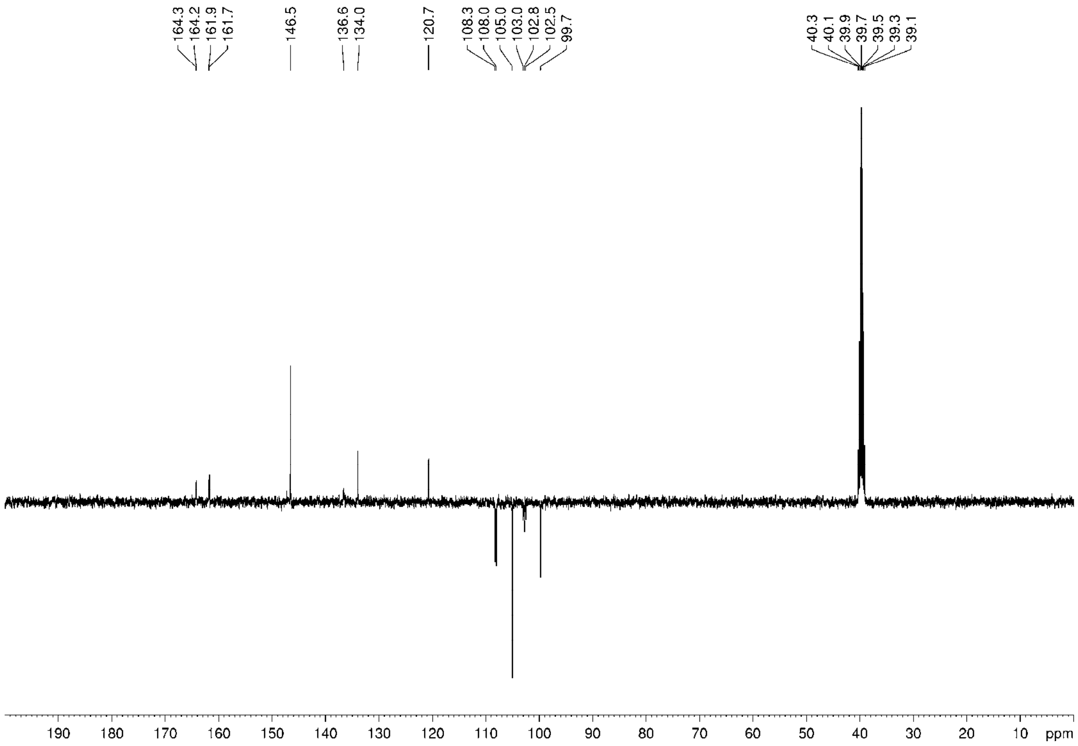

164.3 164.2 161.9 161.7
146.5
136.6 134.0
120.7
108.3 108.0 105.0 103.0 102.8 102.5 99.7
40.3 40.1 39.9 39.7 39.5 39.3 39.1
190 180 170 160 150 140 130 120 110 100 90 80 70 60 50 40 30 20 10 ppm

anle138C

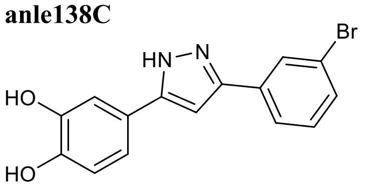

HN
N
Br
HO
HO

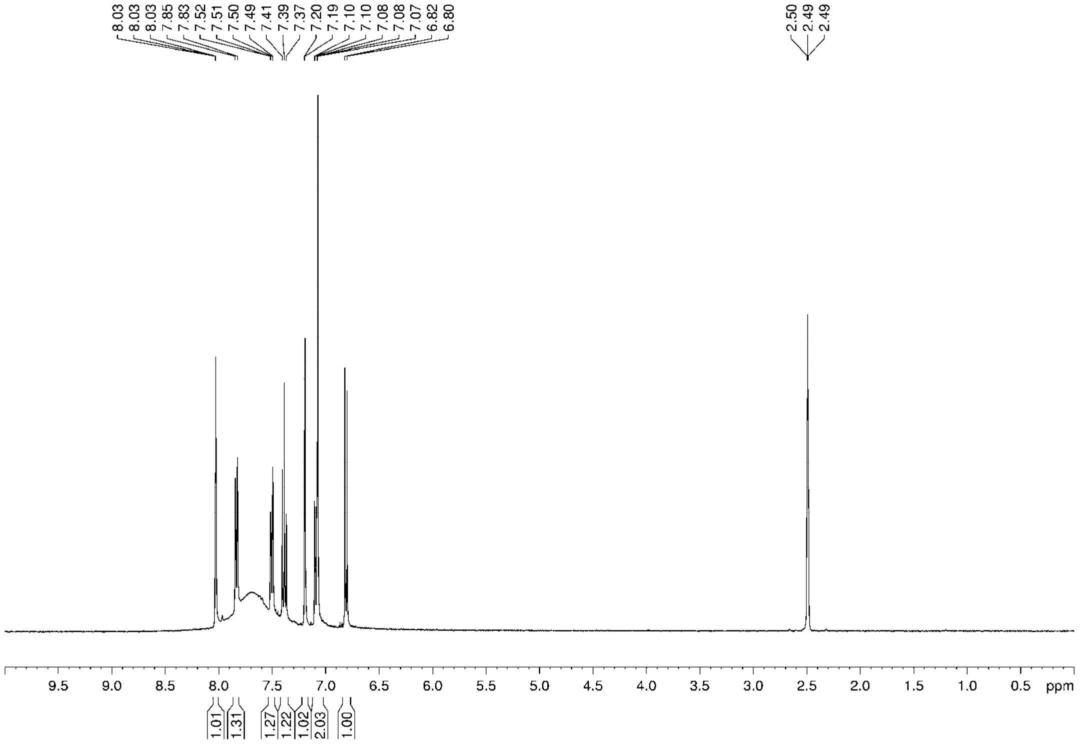

8.03
8.03
8.03
7.85
7.83
7.52
7.51
7.50
7.49
7.41
7.39
7.37
7.20
7.19
7.10
7.08
7.07
6.82
6.80
2.50
2.49
2.49
9.5
9.0
8.5
8.0
7.5
7.0
6.5
6.0
5.5
5.0
4.5
4.0
3.5
3.0
2.5
2.0
1.5
1.0
0.5
ppm
1.01
1.31
1.27
1.22
1.02
2.03
1.00

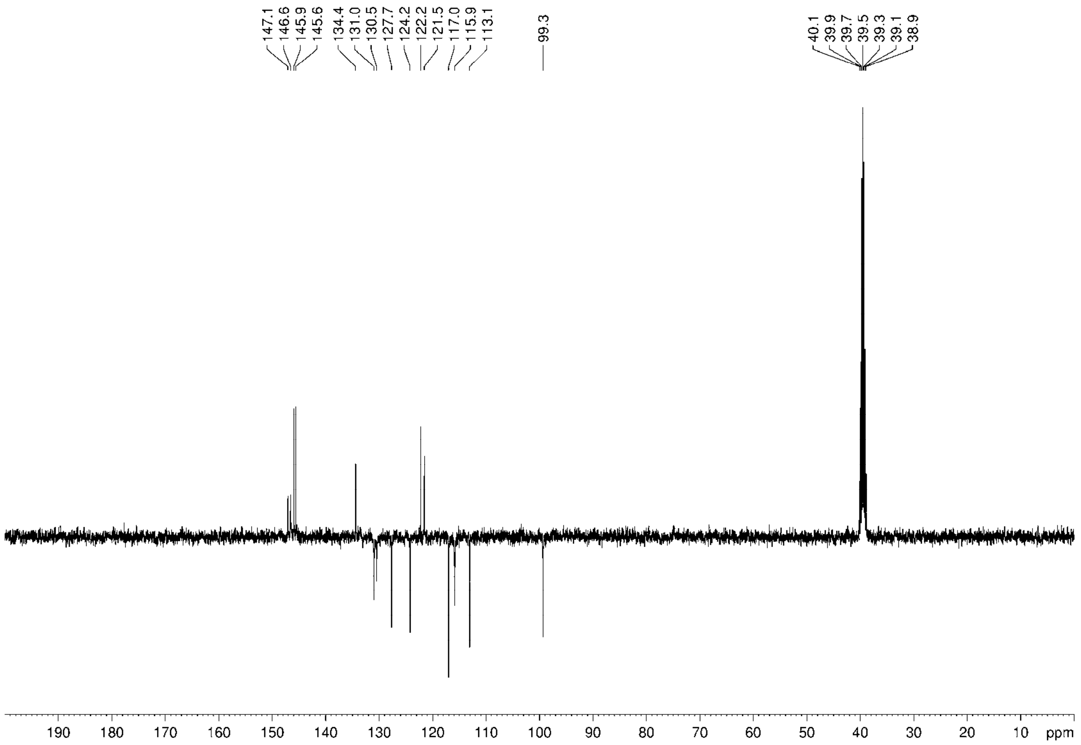

147.1
146.6
145.9
145.6
134.4
131.0
130.5
127.7
124.2
122.2
121.5
117.0
115.9
113.1
99.3
40.1
39.9
39.7
39.5
39.3
39.1
38.9
190
180
170
160
150
140
130
120
110
100
90
80
70
60
50
40
30
20
10
ppm

anle142A

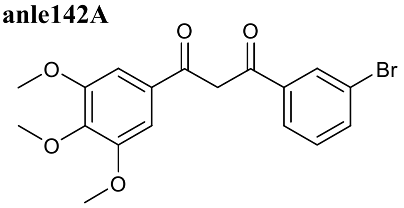

O
O
O
O
O
Br

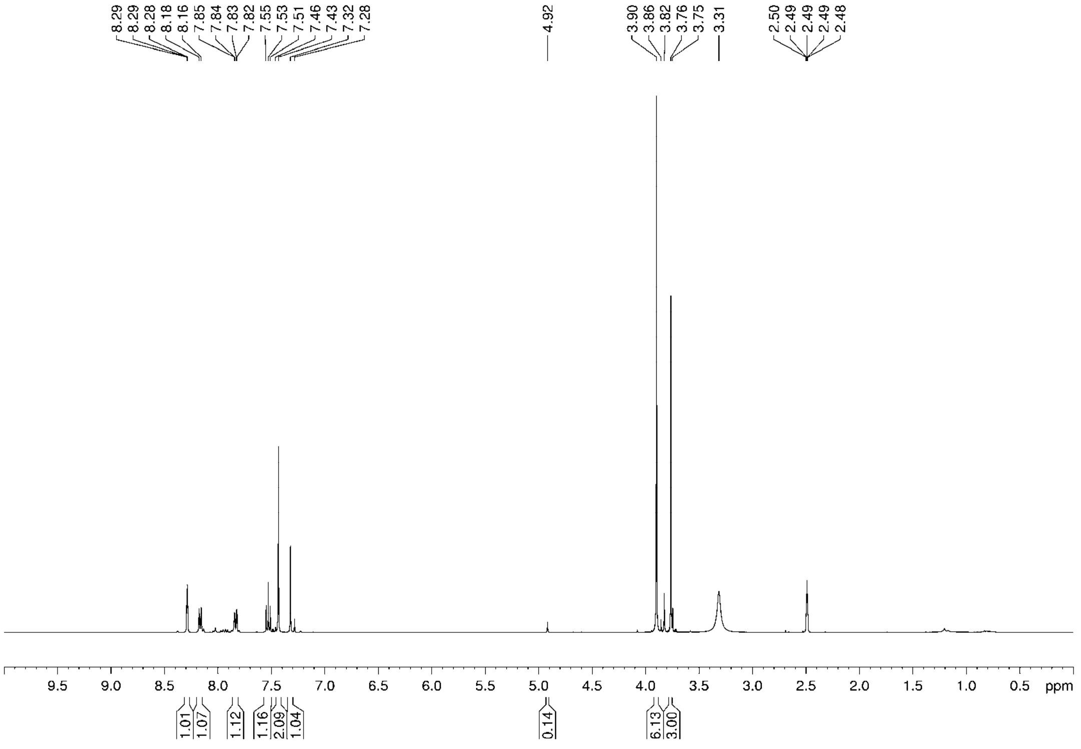

8.29
8.29
8.28
8.18
8.16
7.85
7.84
7.83
7.82
7.55
7.53
7.51
7.46
7.43
7.32
7.28
4.92
3.90
3.86
3.82
3.76
3.75
3.31
2.50
2.49
2.49
2.49
2.48
9.5
9.0
8.5
8.0
7.5
7.0
6.5
6.0
5.5
5.0
4.5
4.0
3.5
3.0
2.5
2.0
1.5
1.0
0.5
ppm
1.01
1.07
1.12
1.16
2.09
1.04
0.14
6.13
3.00

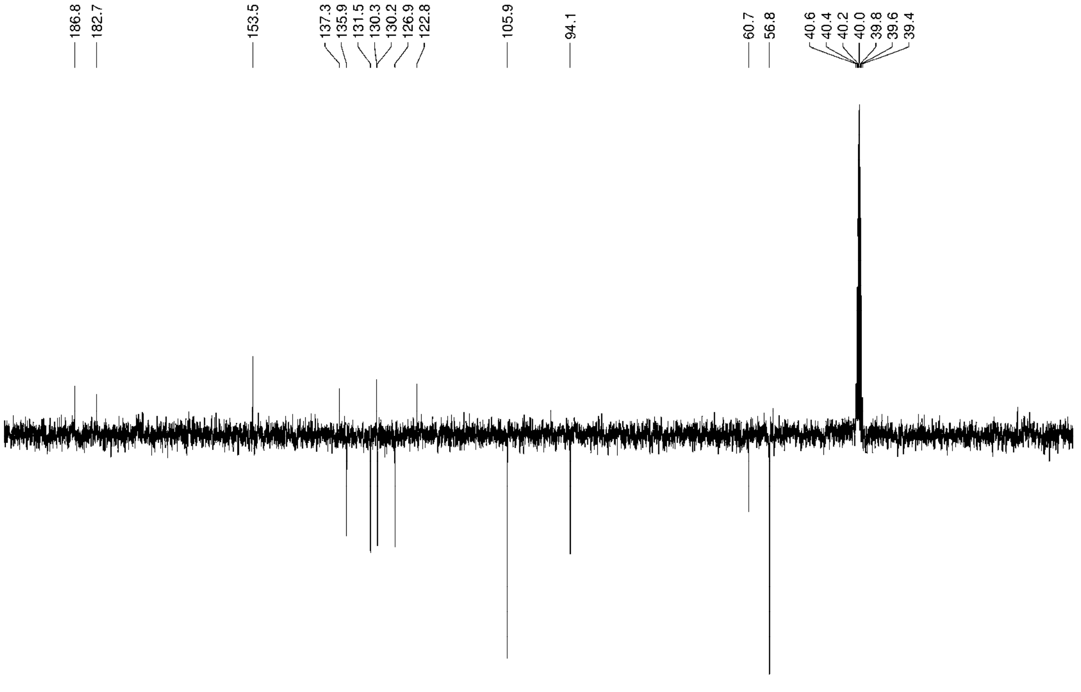

186.8
182.7
153.5
137.3
135.9
131.5
130.3
130.2
126.9
122.8
105.9
94.1
60.7
56.8
40.6
40.4
40.2
40.0
39.8
39.6
39.4
190
180
170
160
150
140
130
120
110
100
90
80
70
60
50
40
30
20
10
ppm

anle142B

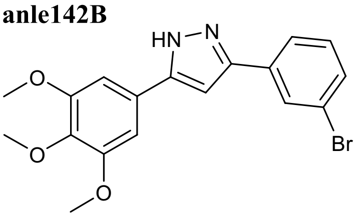
HN
N
O
O
O
Br

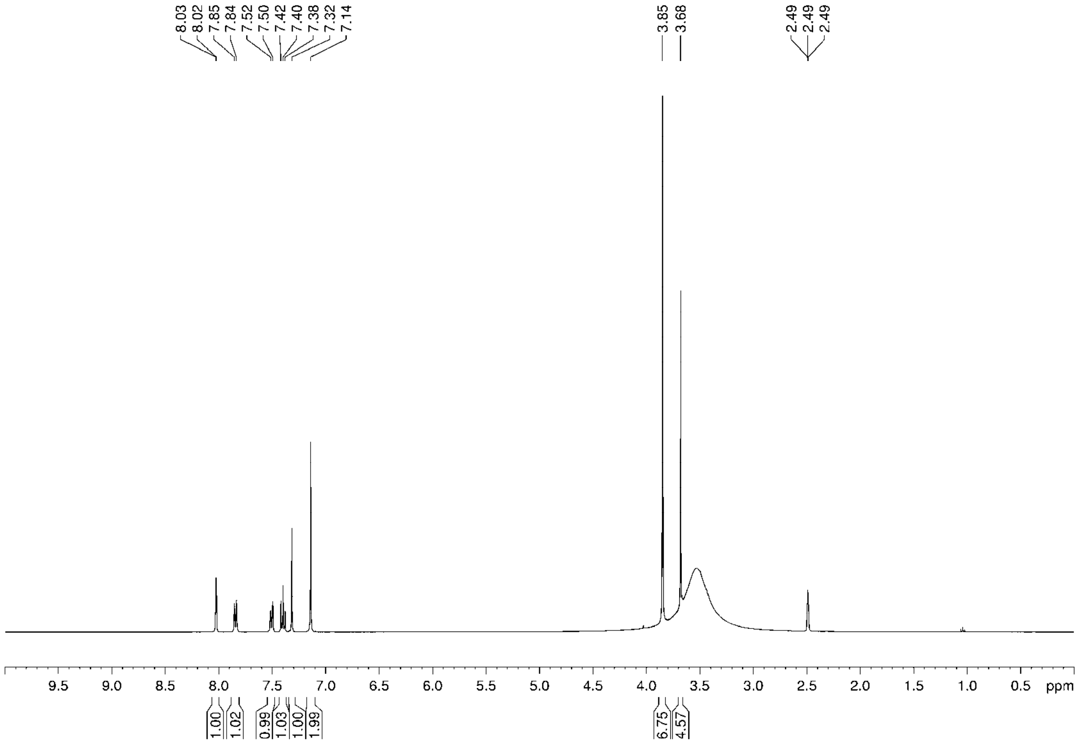
8.03
8.02
7.85
7.84
7.52
7.50
7.42
7.40
7.38
7.32
7.14
3.85
3.68
2.49
2.49
2.49
9.5 9.0 8.5 8.0 7.5 7.0 6.5 6.0 5.5 5.0 4.5 4.0 3.5 3.0 2.5 2.0 1.5 1.0 0.5 ppm
1.00
1.02
0.99
1.03
1.00
1.99
6.75
4.57

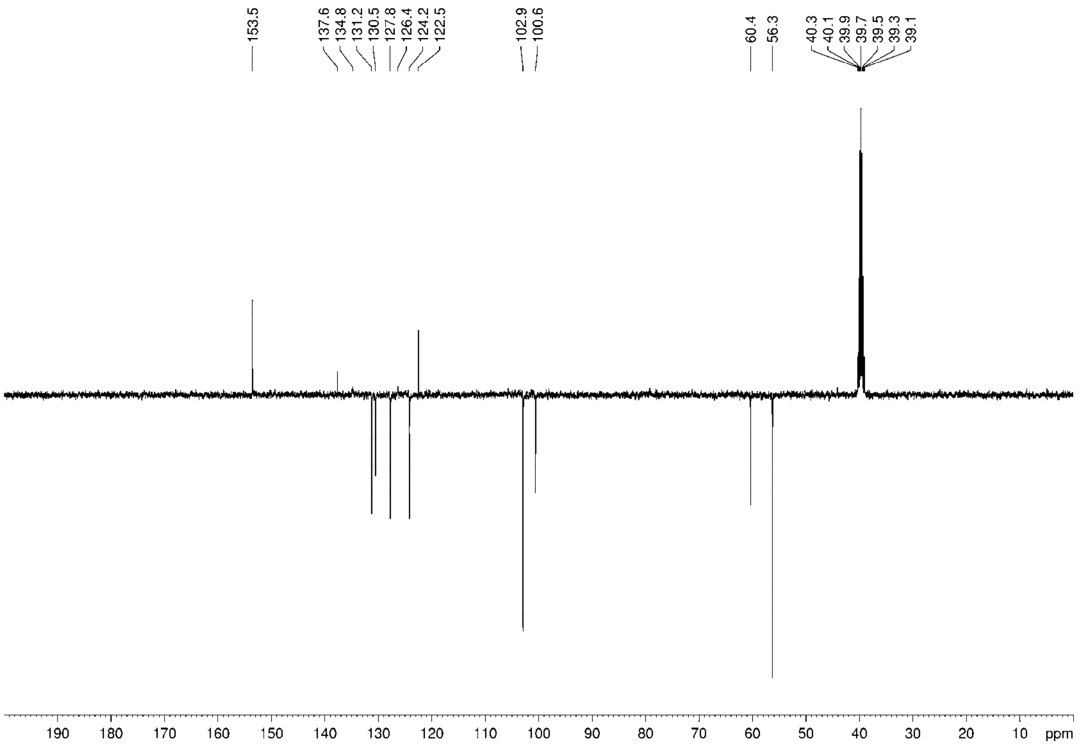
153.5
137.6
134.8
131.2
130.5
127.8
126.4
124.2
122.5
102.9
100.6
60.4
56.3
40.3
40.1
39.9
39.7
39.5
39.3
39.1
190 180 170 160 150 140 130 120 110 100 90 80 70 60 50 40 30 20 10 ppm

anle142C

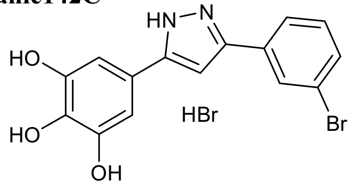
HN–N
HO
HO
OH
HBr
Br

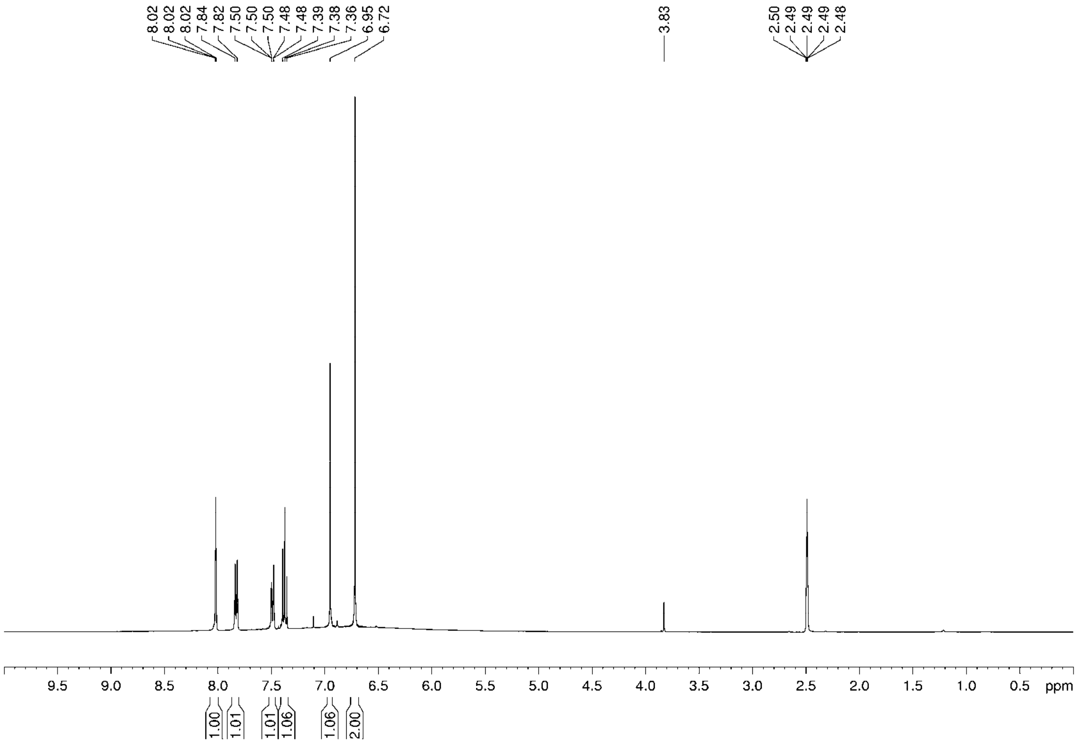
8.02
8.02
8.02
7.84
7.82
7.50
7.50
7.50
7.48
7.48
7.39
7.38
7.36
6.95
6.72
3.83
2.50
2.49
2.49
2.49
2.48
9.5 9.0 8.5 8.0 7.5 7.0 6.5 6.0 5.5 5.0 4.5 4.0 3.5 3.0 2.5 2.0 1.5 1.0 0.5 ppm
1.00
1.01
1.01
1.06
1.06
2.00

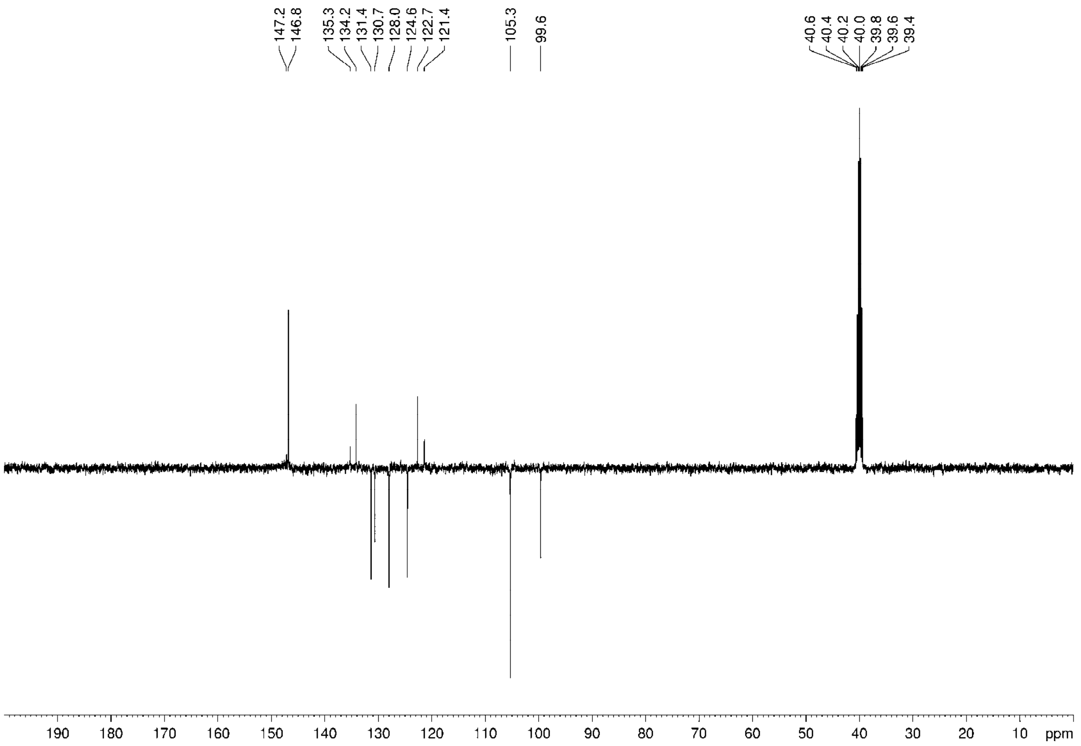
147.2
146.8
135.3
134.2
131.4
130.7
128.0
124.6
122.7
121.4
105.3
99.6
40.6
40.4
40.2
40.0
39.8
39.6
39.4
190 180 170 160 150 140 130 120 110 100 90 80 70 60 50 40 30 20 10 ppm

anle143A

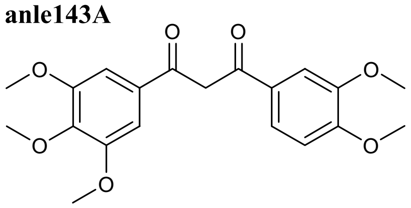

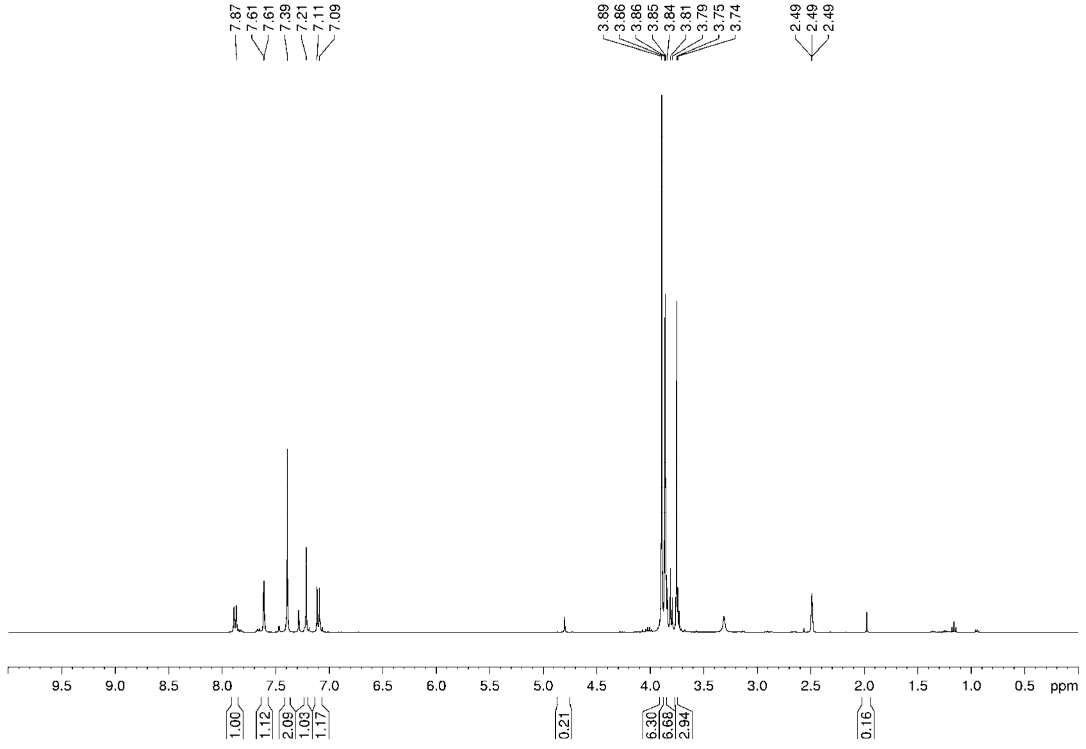

7.87
7.61
7.61
7.39
7.21
7.11
7.09
3.89
3.86
3.85
3.84
3.81
3.79
3.75
3.74
2.49
2.49
2.49
9.5 9.0 8.5 8.0 7.5 7.0 6.5 6.0 5.5 5.0 4.5 4.0 3.5 3.0 2.5 2.0 1.5 1.0 0.5 ppm
1.00
1.12
2.09
1.03
1.17
0.21
6.30
6.68
2.94
0.16

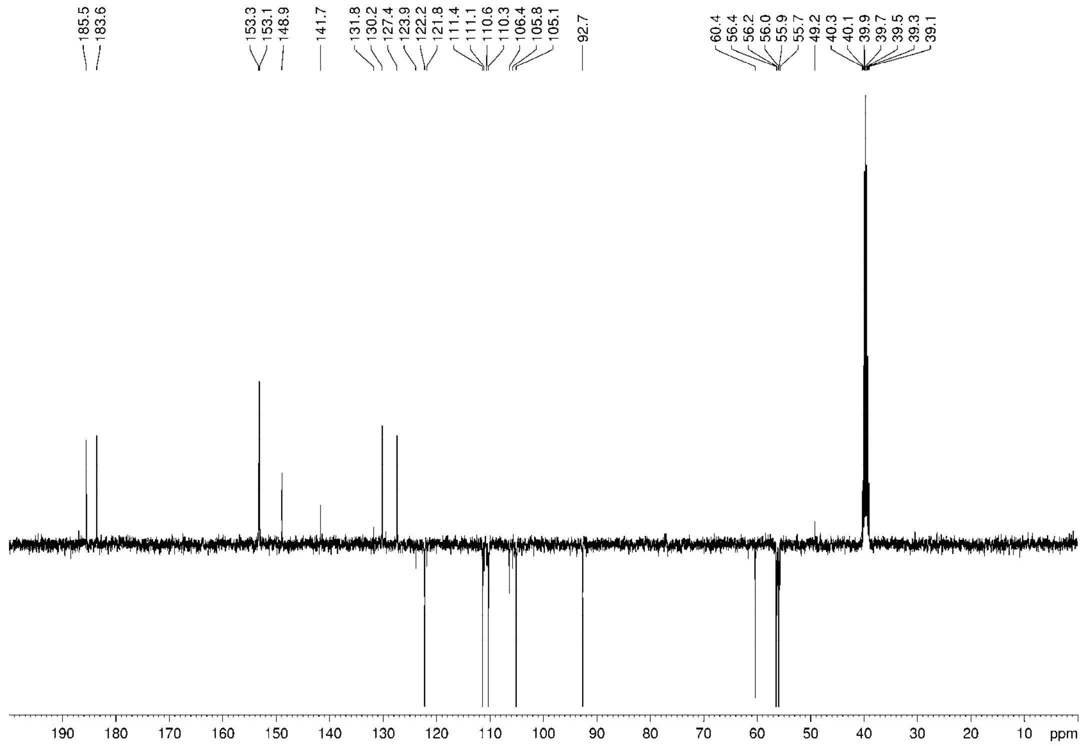

185.5
183.6
153.3
153.1
148.9
141.7
131.8
130.2
127.4
123.9
122.2
121.8
111.4
111.1
110.6
110.3
106.4
105.8
105.1
92.7
60.4
56.4
56.2
56.0
55.9
55.7
49.2
40.3
40.1
39.9
39.7
39.5
39.3
39.1
190 180 170 160 150 140 130 120 110 100 90 80 70 60 50 40 30 20 10 ppm

anle143C

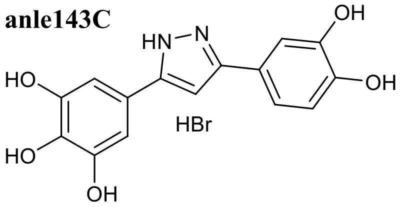

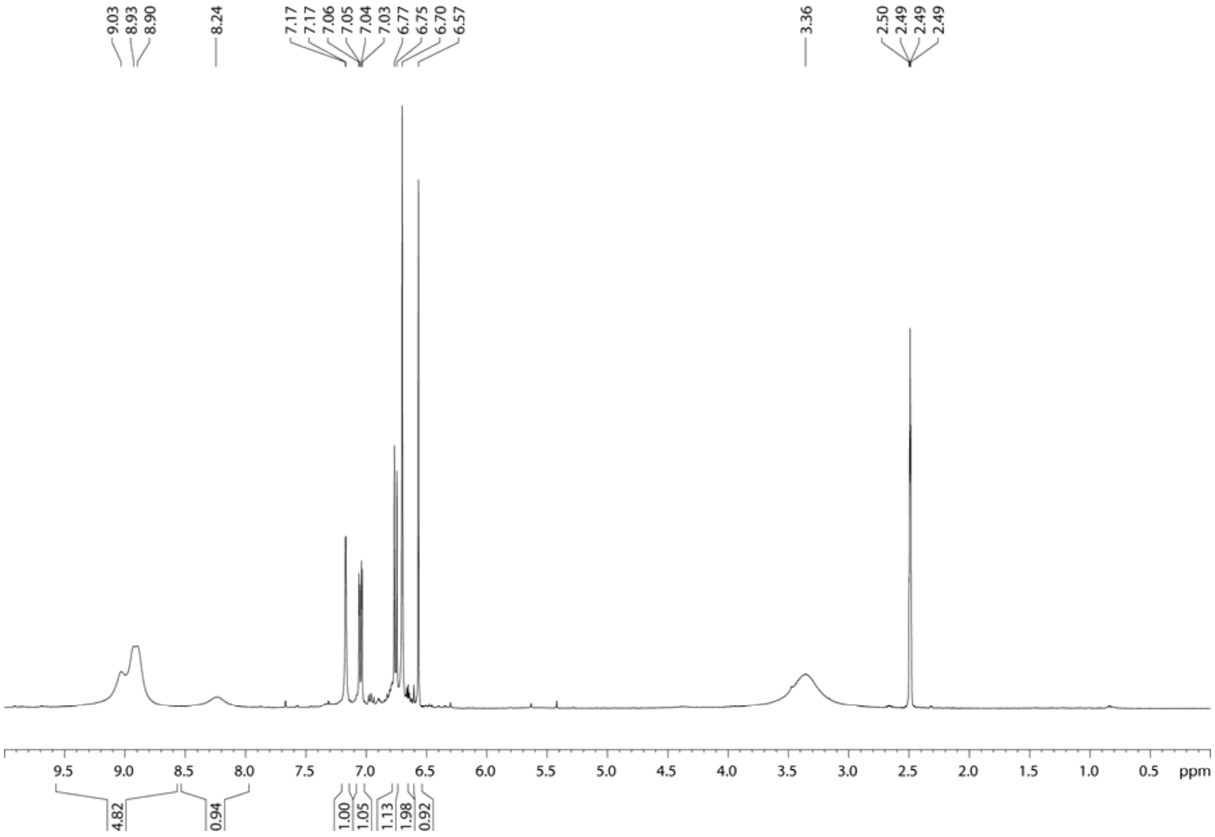

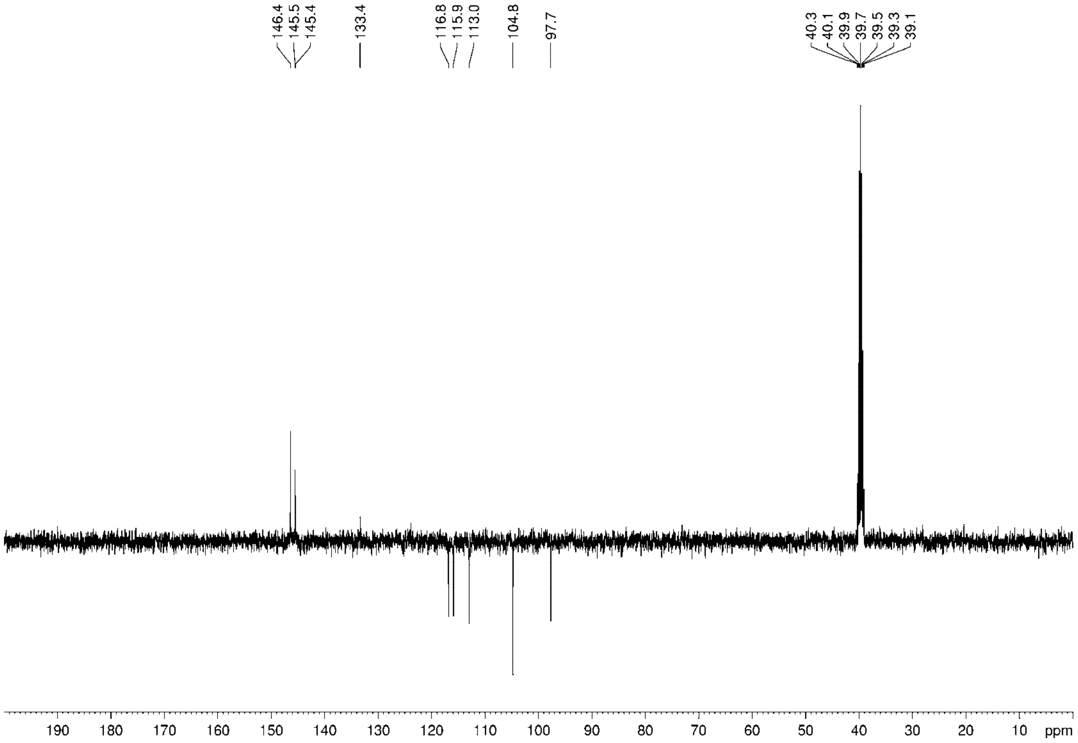

anle145A

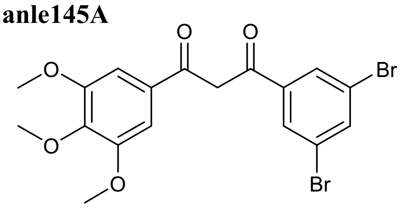

O
O
O
O
O
Br
Br

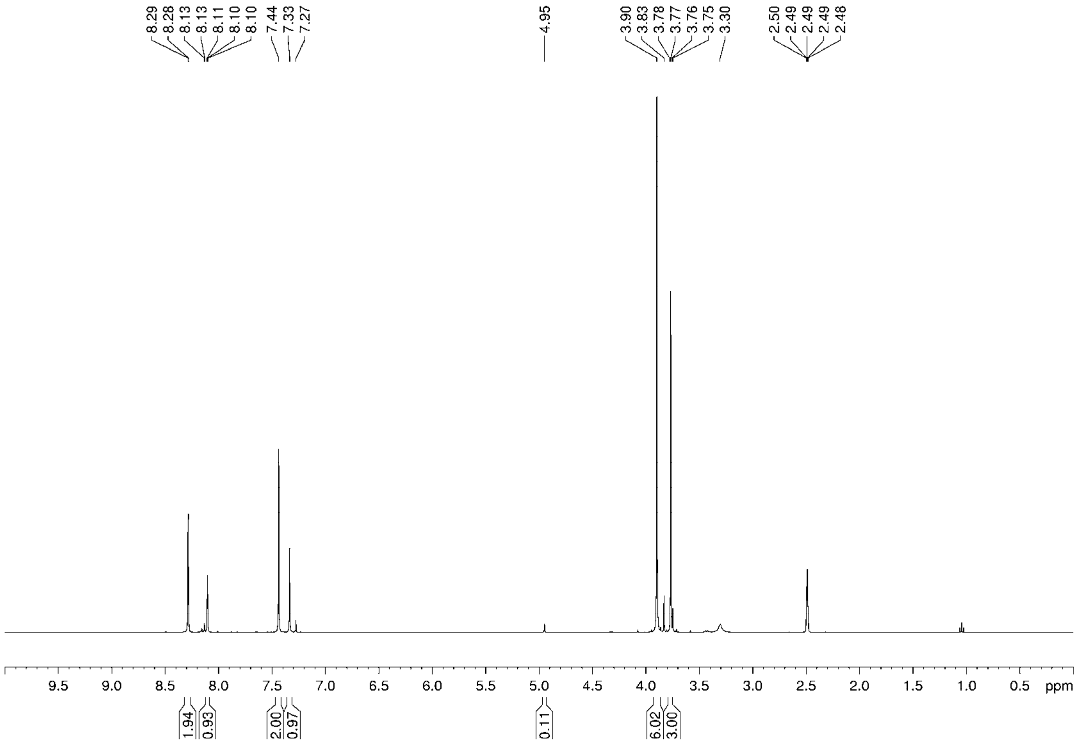

8.29
8.28
8.13
8.13
8.11
8.10
8.10
7.44
7.33
7.27
4.95
3.90
3.83
3.78
3.77
3.76
3.75
3.30
2.50
2.49
2.49
2.49
2.48
9.5
9.0
8.5
8.0
7.5
7.0
6.5
6.0
5.5
5.0
4.5
4.0
3.5
3.0
2.5
2.0
1.5
1.0
0.5
ppm
1.94
0.93
2.00
0.97
0.11
6.02
3.00

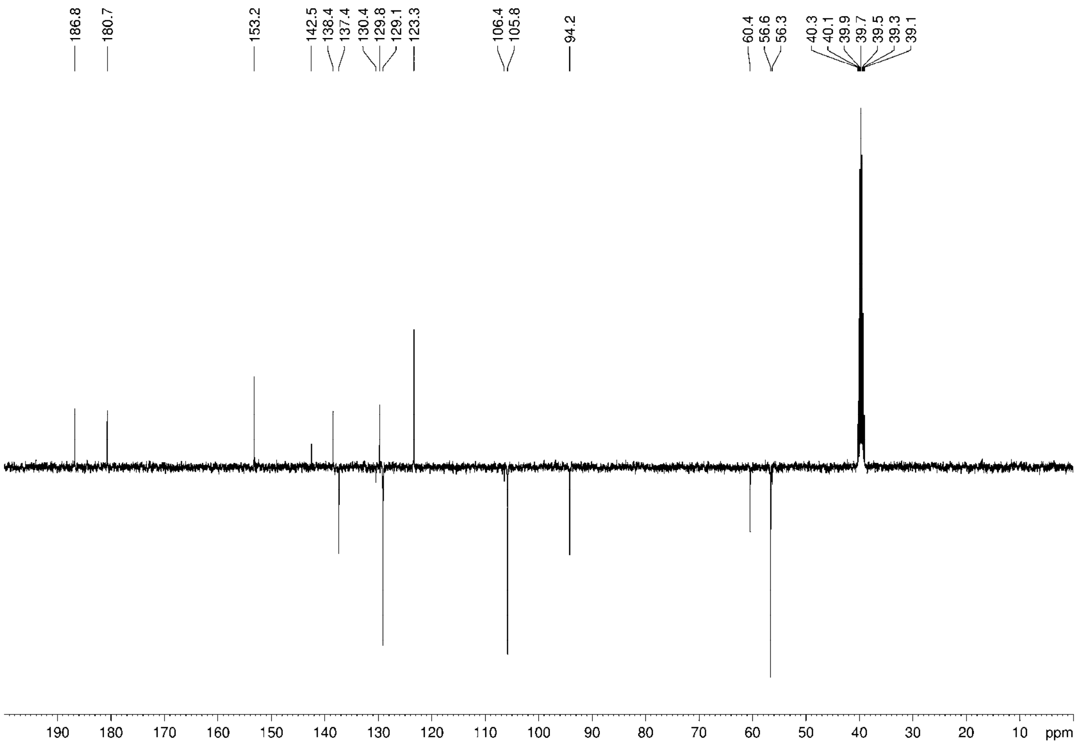

186.8
180.7
153.2
142.5
138.4
137.4
130.4
129.8
129.1
123.3
106.4
105.8
94.2
60.4
56.6
56.3
40.3
40.1
39.9
39.7
39.5
39.3
39.1
190
180
170
160
150
140
130
120
110
100
90
80
70
60
50
40
30
20
10
ppm

anle145B

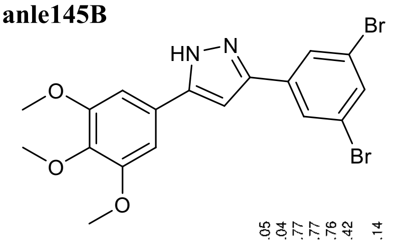
HN
N
Br
Br
O
O
O

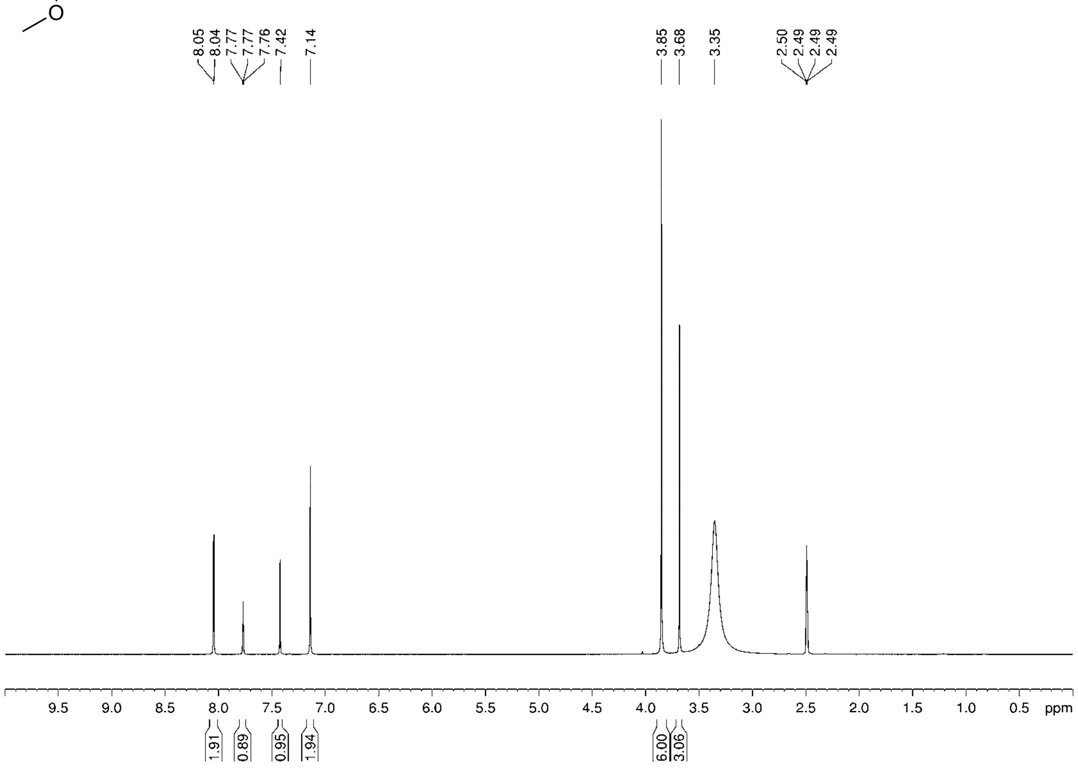
8.05
8.04
7.77
7.77
7.76
7.42
7.14
3.85
3.68
3.35
2.50
2.49
2.49
2.49
9.5 9.0 8.5 8.0 7.5 7.0 6.5 6.0 5.5 5.0 4.5 4.0 3.5 3.0 2.5 2.0 1.5 1.0 0.5 ppm
1.91
0.89
0.95
1.94
6.00
3.06

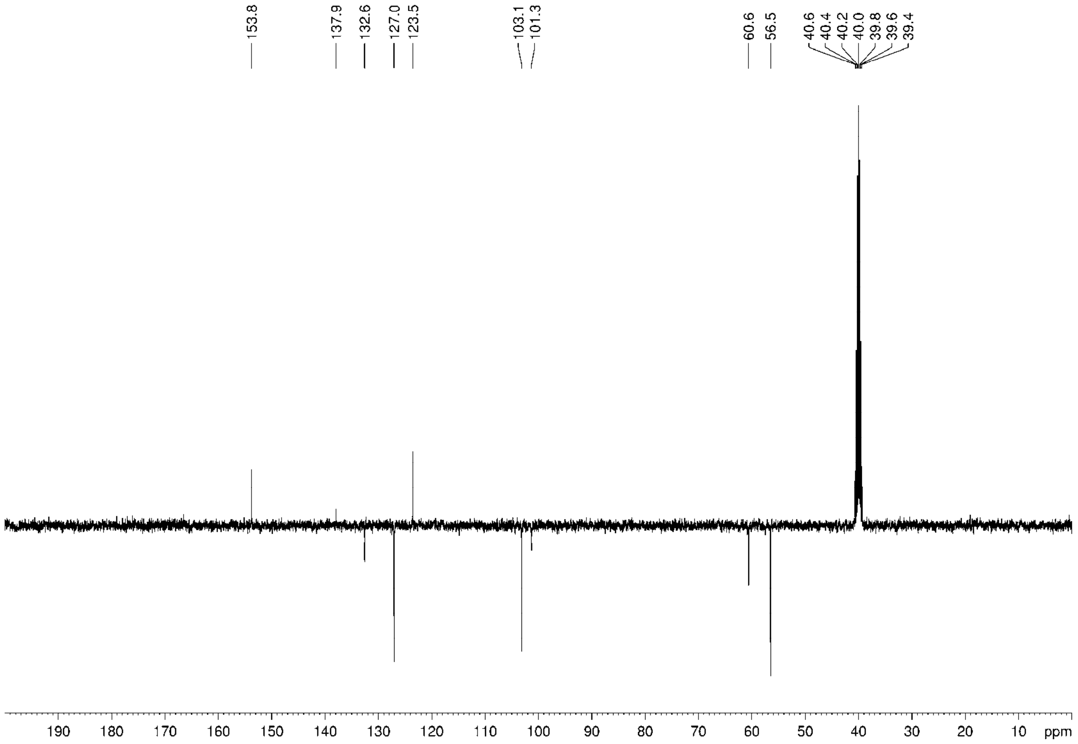
153.8
137.9
132.6
127.0
123.5
103.1
101.3
60.6
56.5
40.6
40.4
40.2
40.0
39.8
39.6
39.4
190 180 170 160 150 140 130 120 110 100 90 80 70 60 50 40 30 20 10 ppm

anle145C

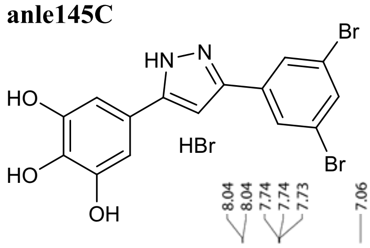
HN
N
Br
HO
HO
OH
HBr
Br

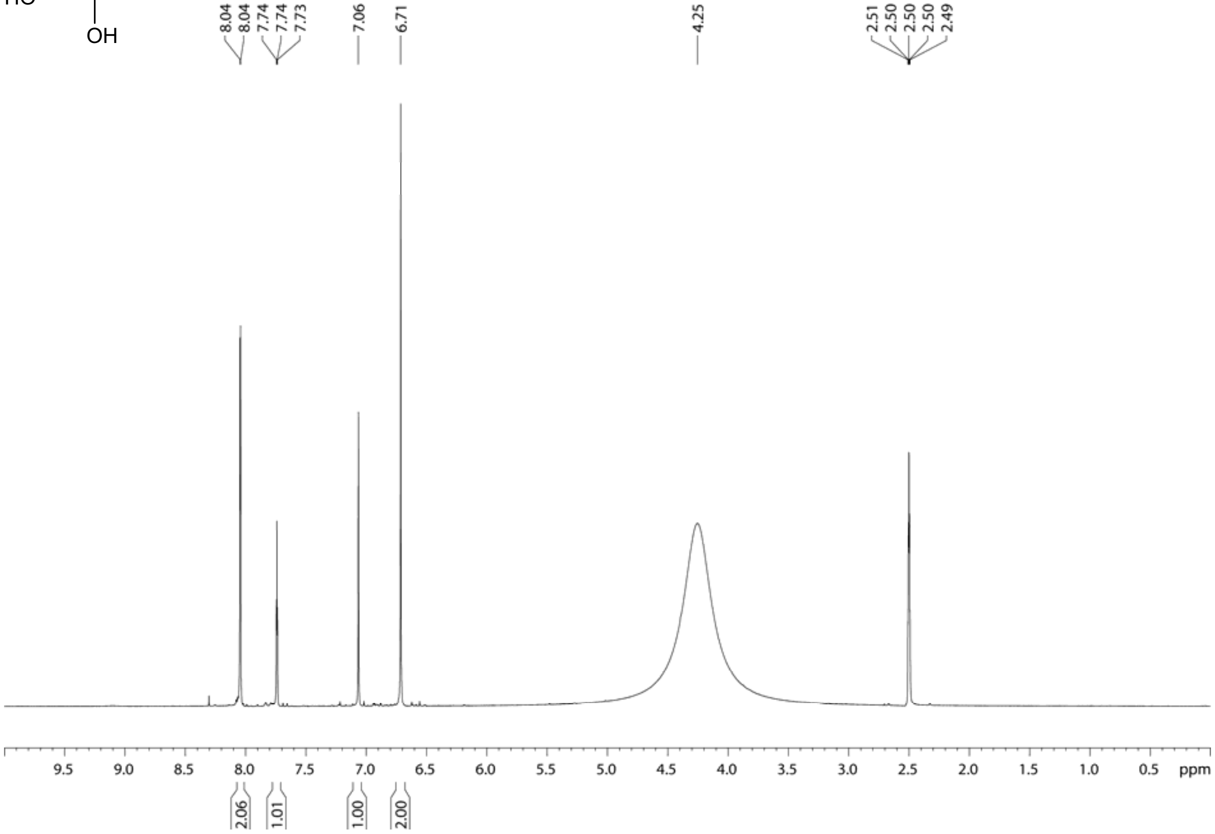
8.04
8.04
7.74
7.74
7.73
7.06
6.71
4.25
2.51
2.50
2.50
2.50
2.49
9.5 9.0 8.5 8.0 7.5 7.0 6.5 6.0 5.5 5.0 4.5 4.0 3.5 3.0 2.5 2.0 1.5 1.0 0.5 ppm
2.06
1.01
1.00
2.00

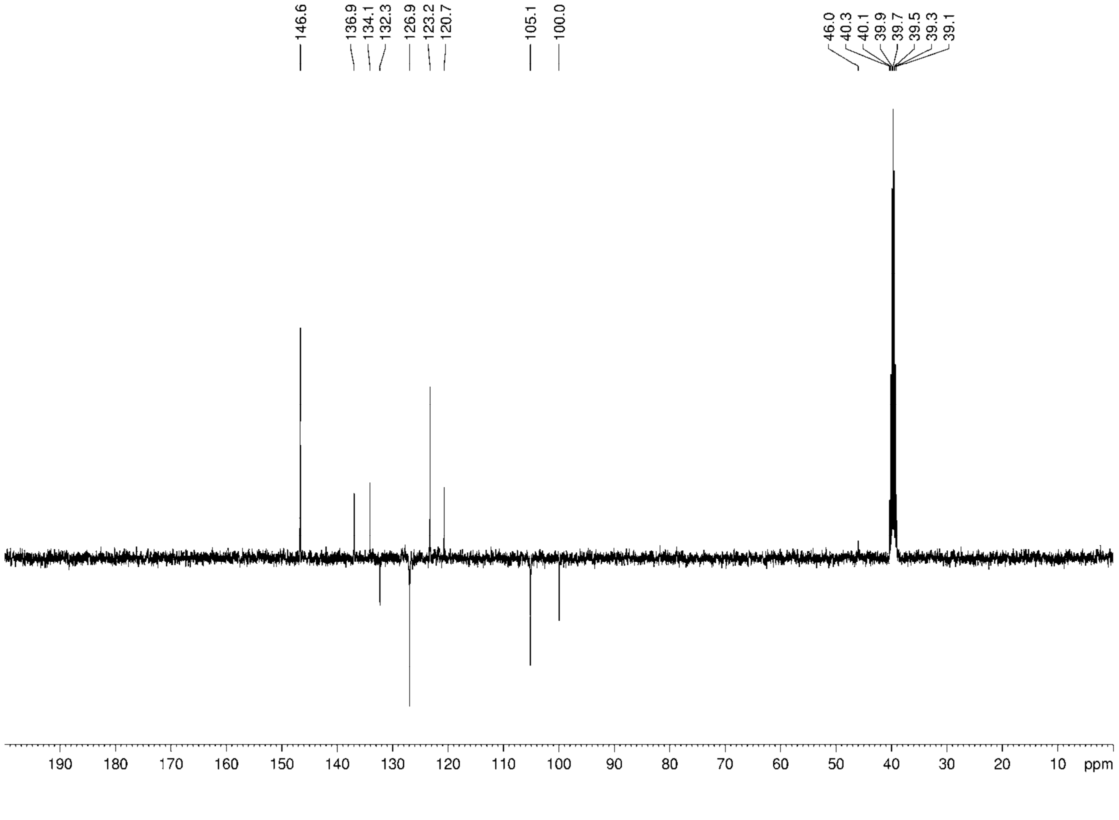
146.6
136.9
134.1
132.3
126.9
123.2
120.7
105.1
100.0
46.0
40.3
40.1
39.9
39.7
39.5
39.3
39.1
190 180 170 160 150 140 130 120 110 100 90 80 70 60 50 40 30 20 10 ppm

anle186A

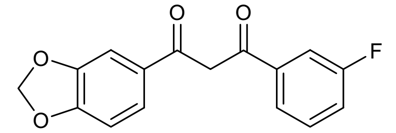

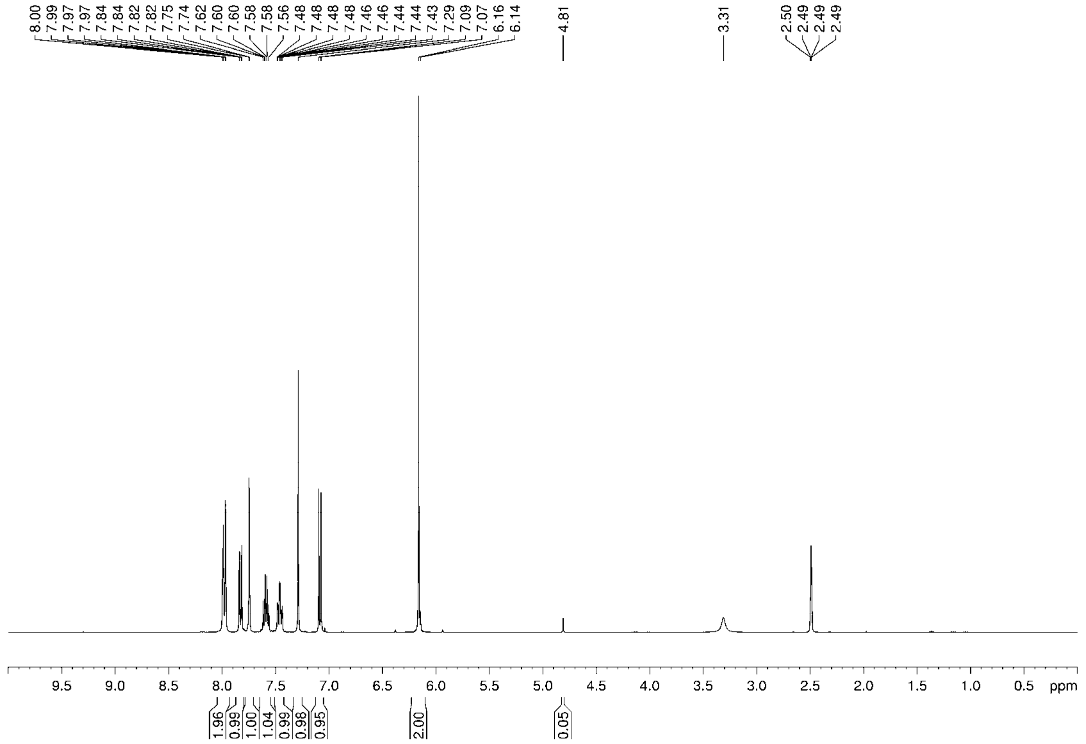

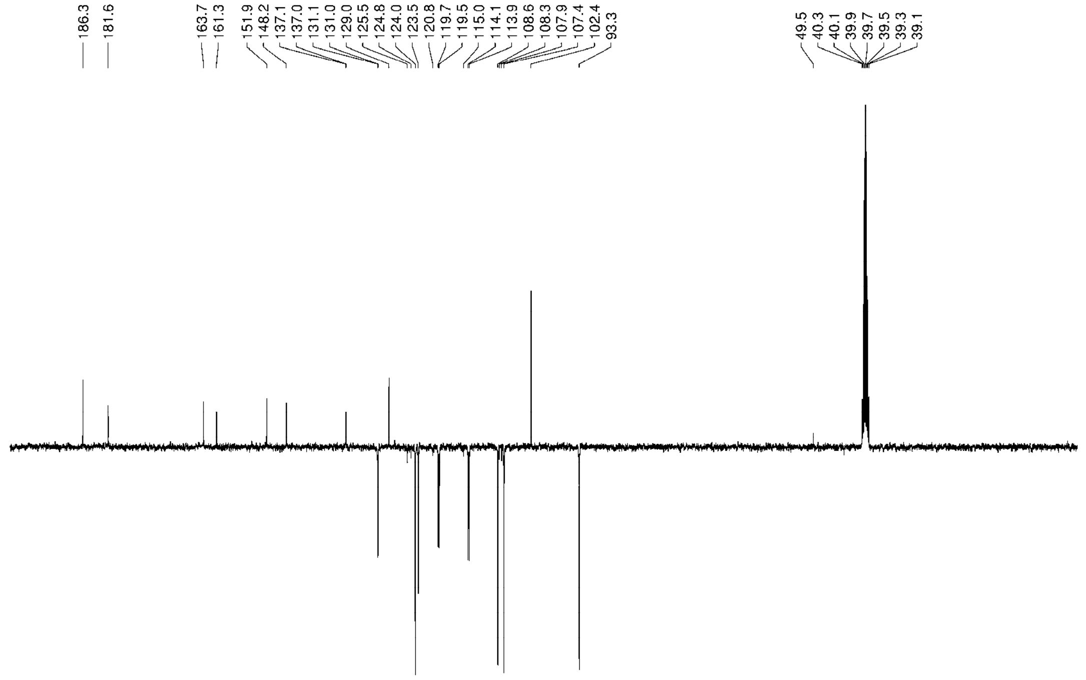

anle186B

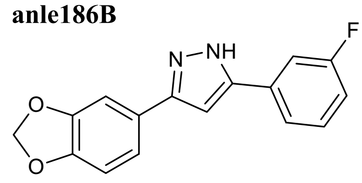

N
NH
F
O
O

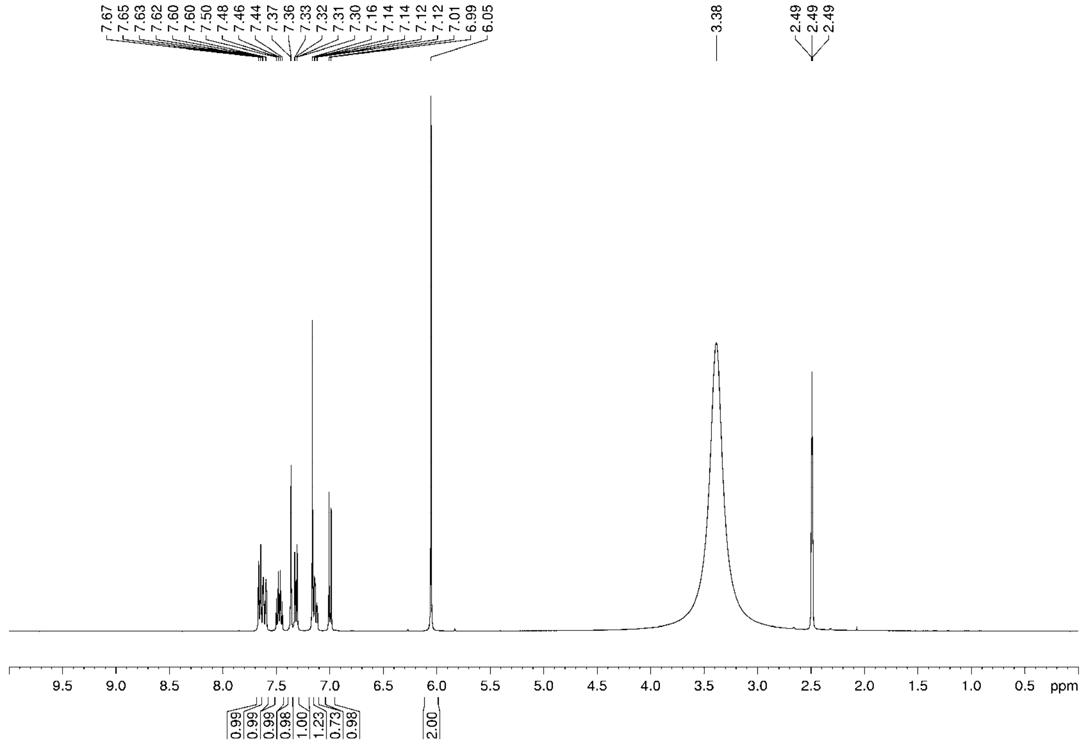

7.67
7.65
7.63
7.62
7.60
7.60
7.50
7.48
7.46
7.44
7.37
7.36
7.33
7.32
7.31
7.30
7.16
7.14
7.14
7.12
7.12
7.01
6.99
6.05
3.38
2.49
2.49
2.49
9.5
9.0
8.5
8.0
7.5
7.0
6.5
6.0
5.5
5.0
4.5
4.0
3.5
3.0
2.5
2.0
1.5
1.0
0.5
ppm
0.99
0.99
0.99
0.98
1.00
1.23
0.73
0.98
2.00

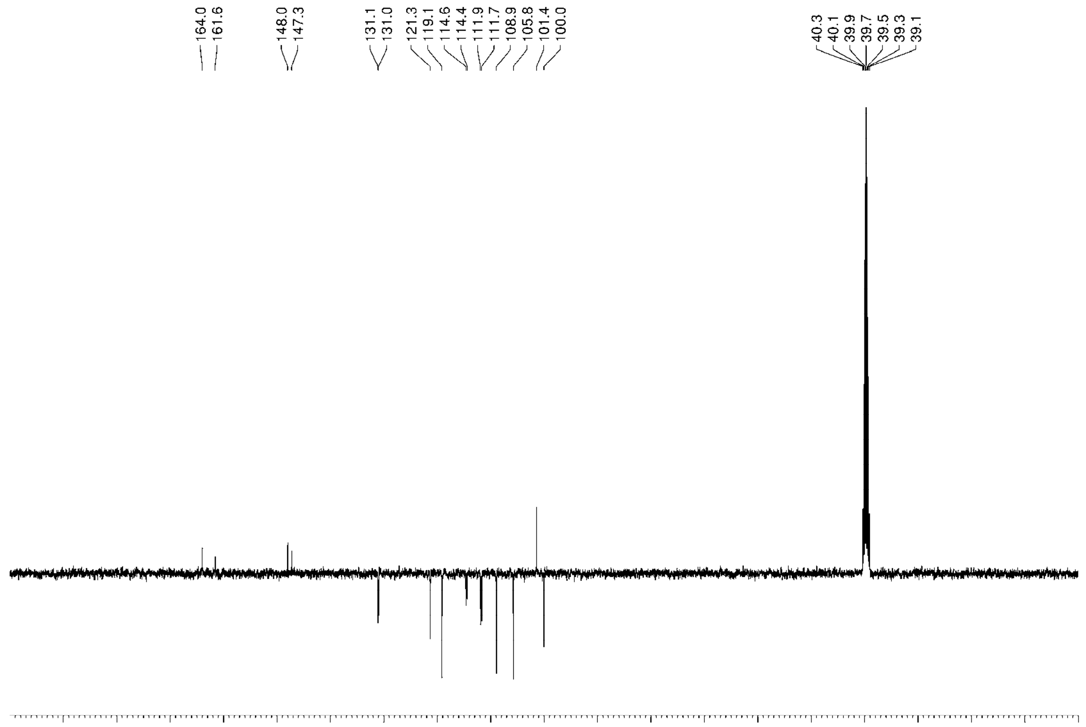

164.0
161.6
148.0
147.3
131.1
131.0
121.3
119.1
114.6
114.4
111.9
111.7
108.9
105.8
101.4
100.0
40.3
40.1
39.9
39.7
39.5
39.3
39.1
190
180
170
160
150
140
130
120
110
100
90
80
70
60
50
40
30
20
10
ppm

anle197A

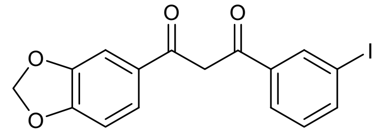

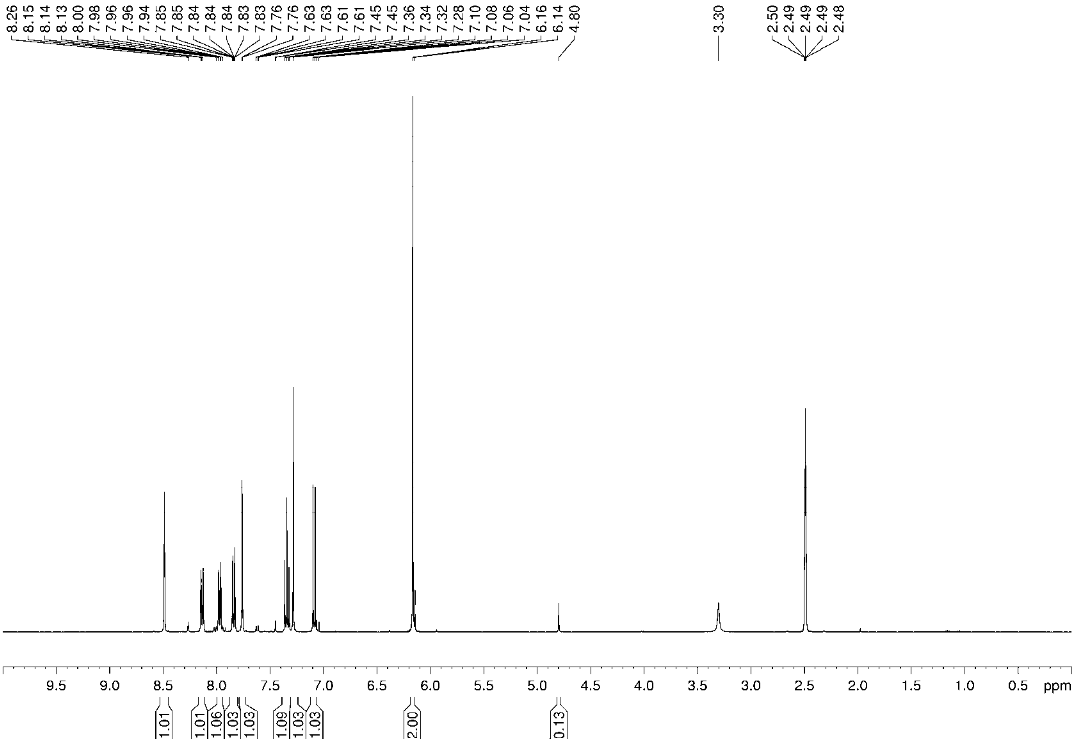
8.26 8.15 8.14 8.13 8.00 7.98 7.96 7.96 7.94 7.85 7.85 7.84 7.84 7.84 7.83 7.83 7.76 7.76 7.63 7.63 7.61 7.61 7.45 7.45 7.36 7.34 7.32 7.28 7.10 7.08 7.06 7.04 6.16 6.14 4.80
3.30
2.50 2.49 2.49 2.49 2.48
9.5 9.0 8.5 8.0 7.5 7.0 6.5 6.0 5.5 5.0 4.5 4.0 3.5 3.0 2.5 2.0 1.5 1.0 0.5 ppm
1.01 1.01 1.06 1.03 1.03 1.09 1.03 1.03 2.00 0.13

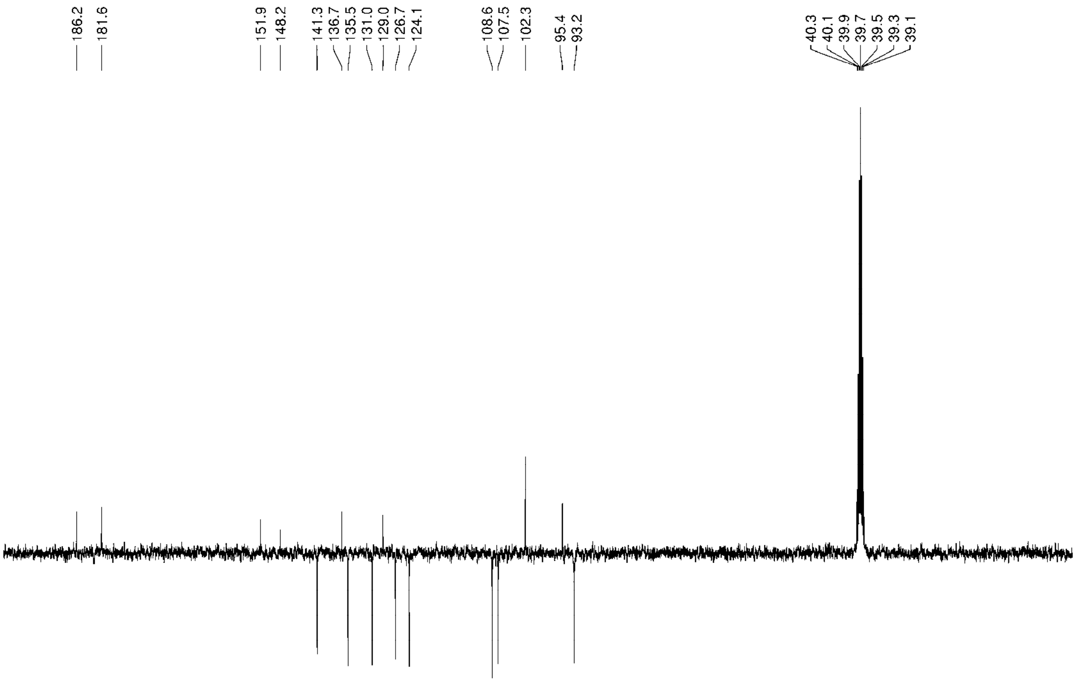
186.2 181.6
151.9 148.2
141.3 136.7 135.5 131.0 129.0 126.7 124.1
108.6 107.5 102.3
95.4 93.2
40.3 40.1 39.9 39.7 39.5 39.3 39.1
190 180 170 160 150 140 130 120 110 100 90 80 70 60 50 40 30 20 10 ppm

anle197B

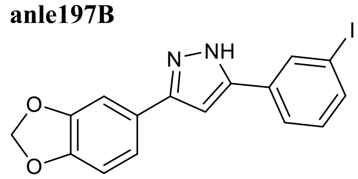

N
NH
I
O
O

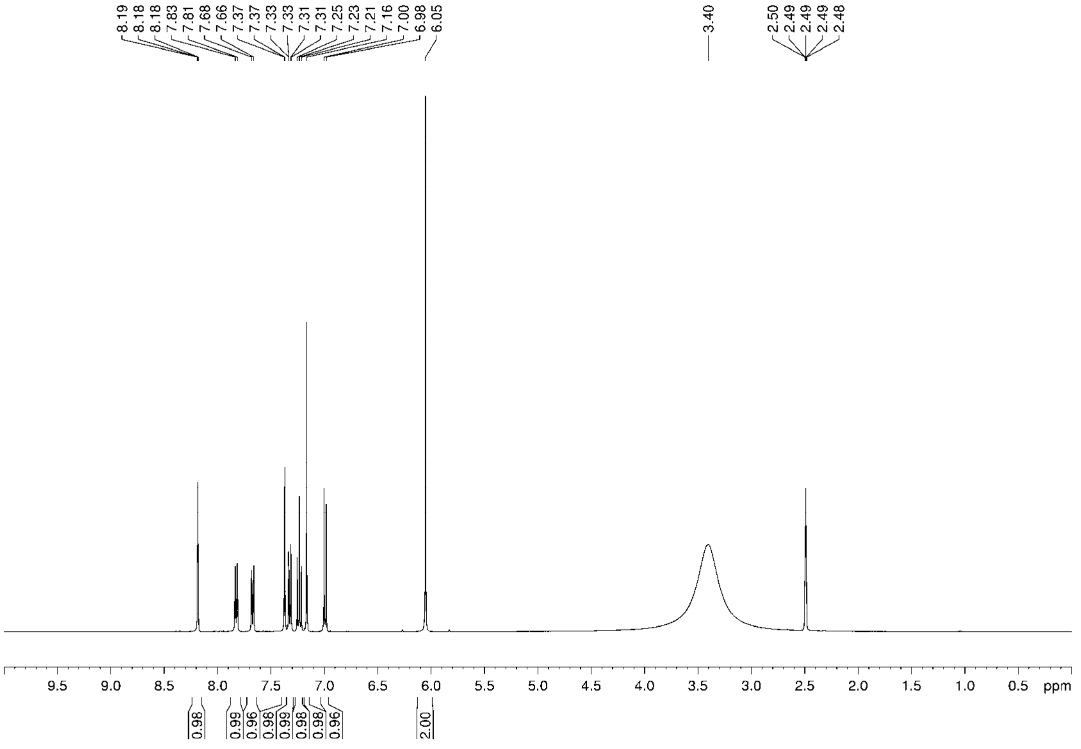

8.19 8.18 8.18 7.83 7.81 7.68 7.66 7.37 7.37 7.33 7.33 7.31 7.31 7.25 7.23 7.21 7.16 7.00 6.98 6.05
3.40
2.50 2.49 2.49 2.49 2.48
9.5 9.0 8.5 8.0 7.5 7.0 6.5 6.0 5.5 5.0 4.5 4.0 3.5 3.0 2.5 2.0 1.5 1.0 0.5 ppm
0.98 0.99 0.96 0.96 0.99 0.98 0.98 0.96 2.00

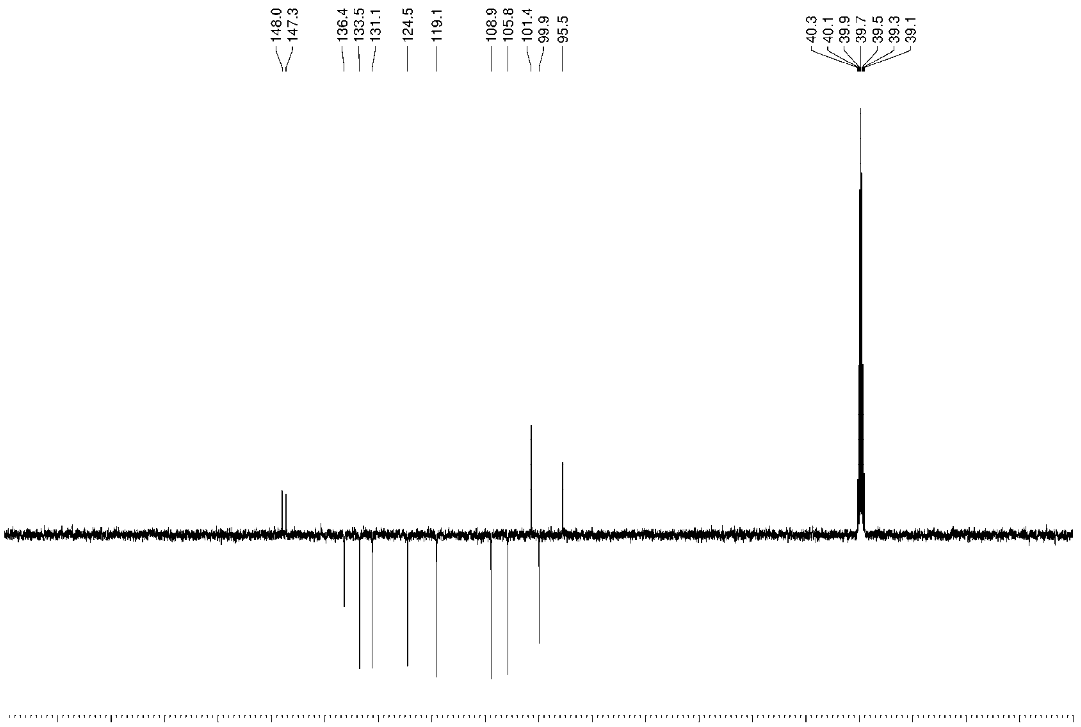

148.0 147.3 136.4 133.5 131.1 124.5 119.1 108.9 105.8 101.4 99.9 95.5
40.3 40.1 39.9 39.7 39.5 39.3 39.1
190 180 170 160 150 140 130 120 110 100 90 80 70 60 50 40 30 20 10 ppm

anle234A

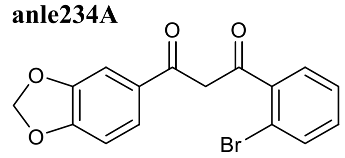
O
O
O
O
Br

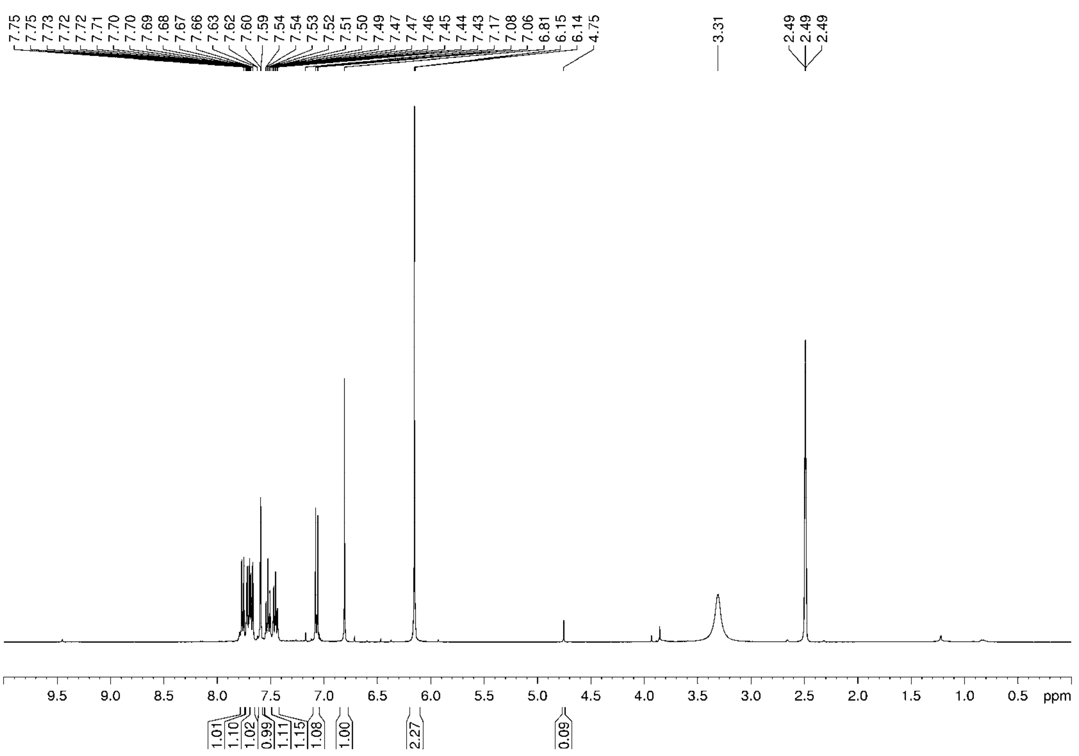
7.75 7.75 7.73 7.72 7.72 7.71 7.70 7.70 7.69 7.68 7.67 7.66 7.63 7.62 7.60 7.59 7.54 7.54 7.53 7.52 7.51 7.50 7.49 7.47 7.47 7.46 7.45 7.44 7.43 7.17 7.08 7.06 6.81 6.15 6.14 4.75
3.31
2.49 2.49 2.49
9.5 9.0 8.5 8.0 7.5 7.0 6.5 6.0 5.5 5.0 4.5 4.0 3.5 3.0 2.5 2.0 1.5 1.0 0.5 ppm
1.01 1.10 1.02 0.99 1.11 1.15 1.08 1.00 2.27 0.09

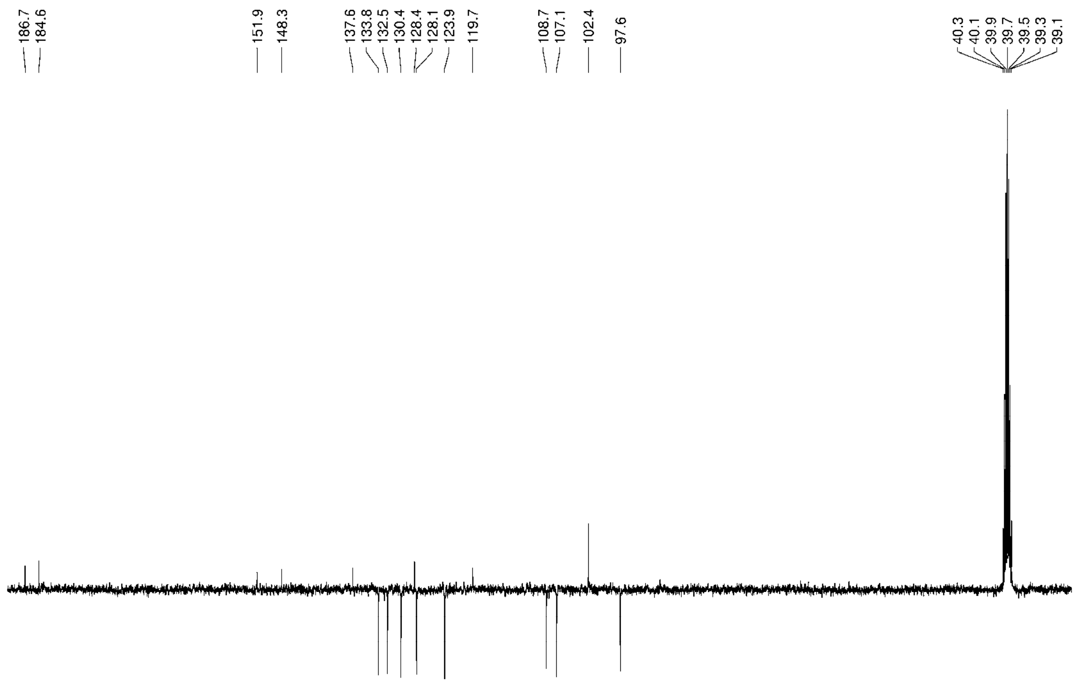
186.7 184.6
151.9 148.3
137.6 133.8 132.5 130.4 128.4 128.1 123.9 119.7
108.7 107.1 102.4 97.6
40.3 40.1 39.9 39.7 39.5 39.3 39.1
180 170 160 150 140 130 120 110 100 90 80 70 60 50 40 ppm

anle234B

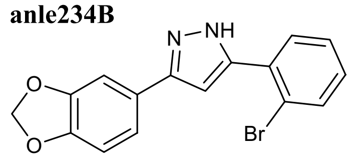

N
NH
O
O
Br

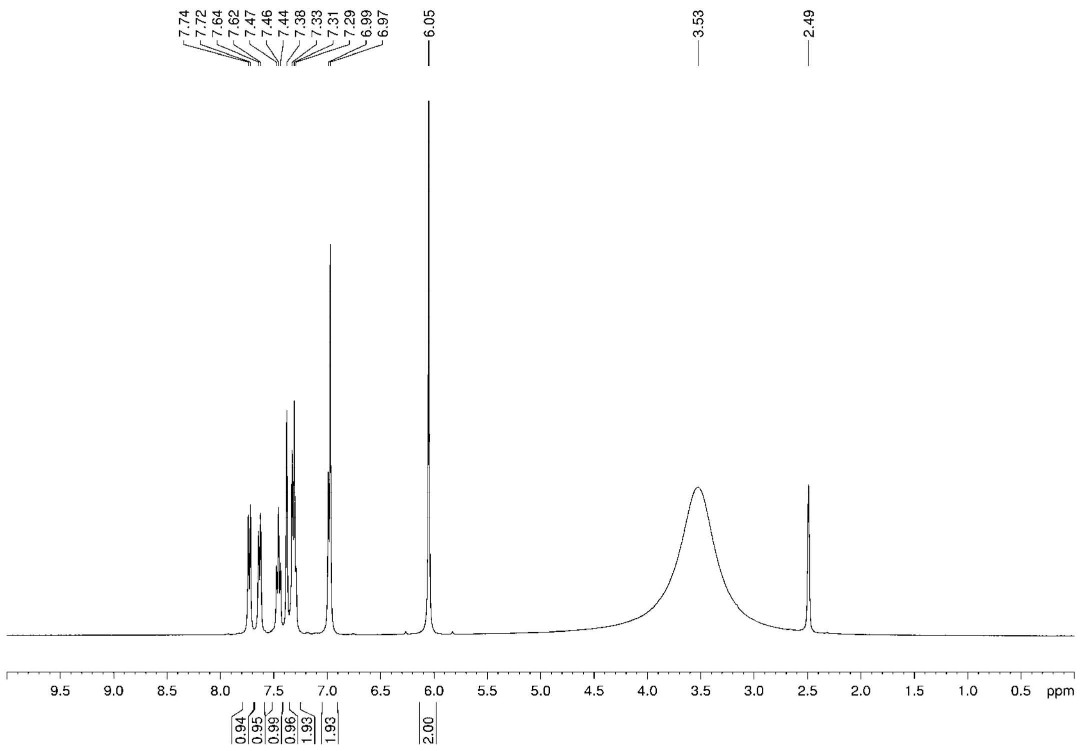

7.74
7.72
7.64
7.62
7.47
7.46
7.44
7.38
7.33
7.31
7.29
6.99
6.97
6.05
3.53
2.49
9.5
9.0
8.5
8.0
7.5
7.0
6.5
6.0
5.5
5.0
4.5
4.0
3.5
3.0
2.5
2.0
1.5
1.0
0.5
ppm
0.94
0.95
0.99
0.96
1.93
1.93
2.00

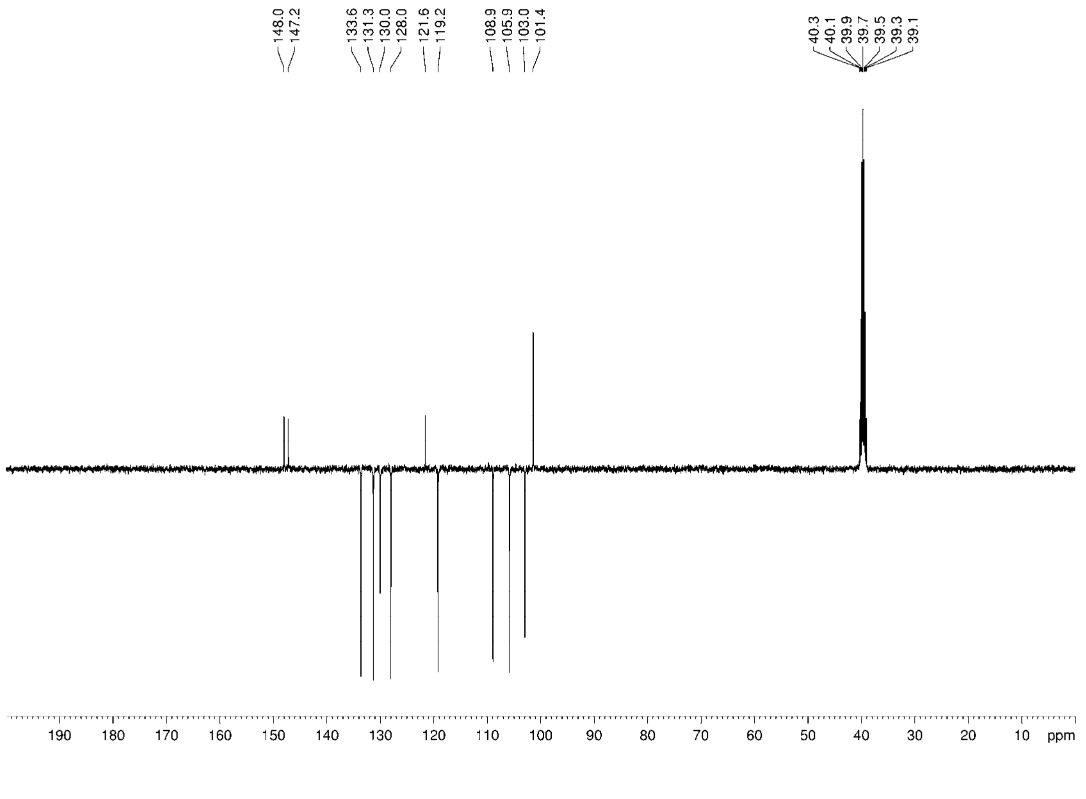

148.0
147.2
133.6
131.3
130.0
128.0
121.6
119.2
108.9
105.9
103.0
101.4
40.3
40.1
39.9
39.7
39.5
39.3
39.1
190
180
170
160
150
140
130
120
110
100
90
80
70
60
50
40
30
20
10
ppm

anle236A

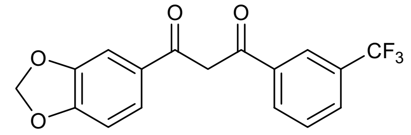

O
O
O
O
CF3

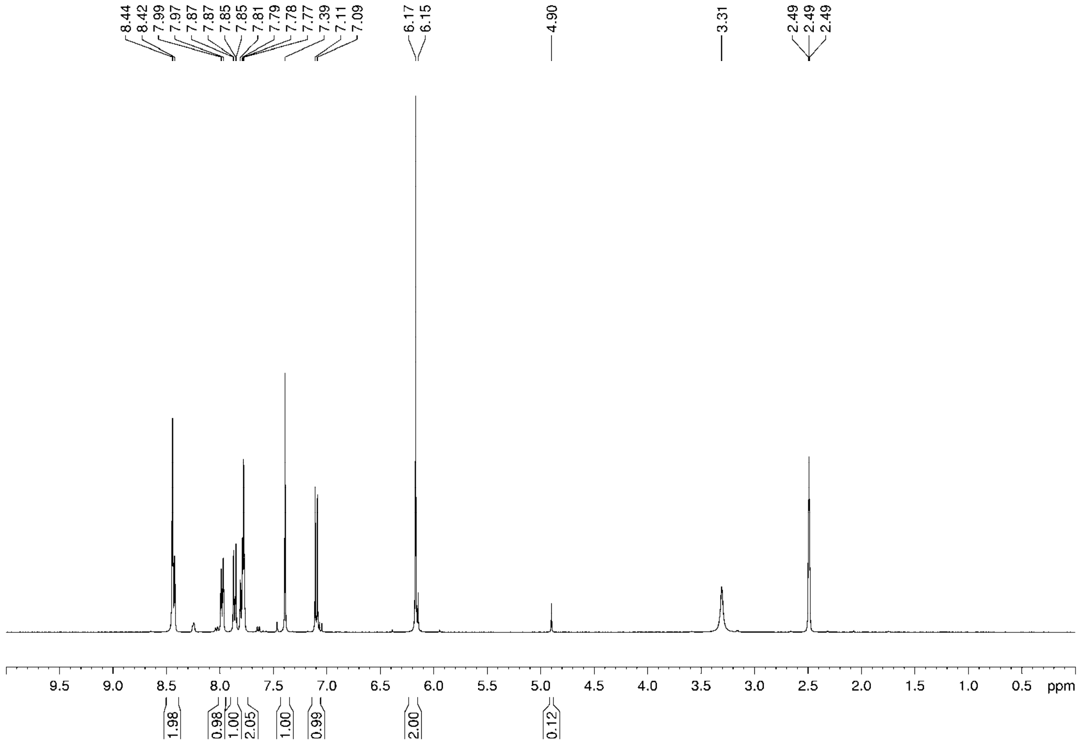

8.44 8.42 7.99 7.97 7.87 7.87 7.85 7.85 7.81 7.79 7.78 7.77 7.39 7.11 7.09
6.17 6.15
4.90
3.31
2.49 2.49 2.49
9.5 9.0 8.5 8.0 7.5 7.0 6.5 6.0 5.5 5.0 4.5 4.0 3.5 3.0 2.5 2.0 1.5 1.0 0.5 ppm
1.98 0.98 1.00 2.05 1.00 0.99 2.00 0.12

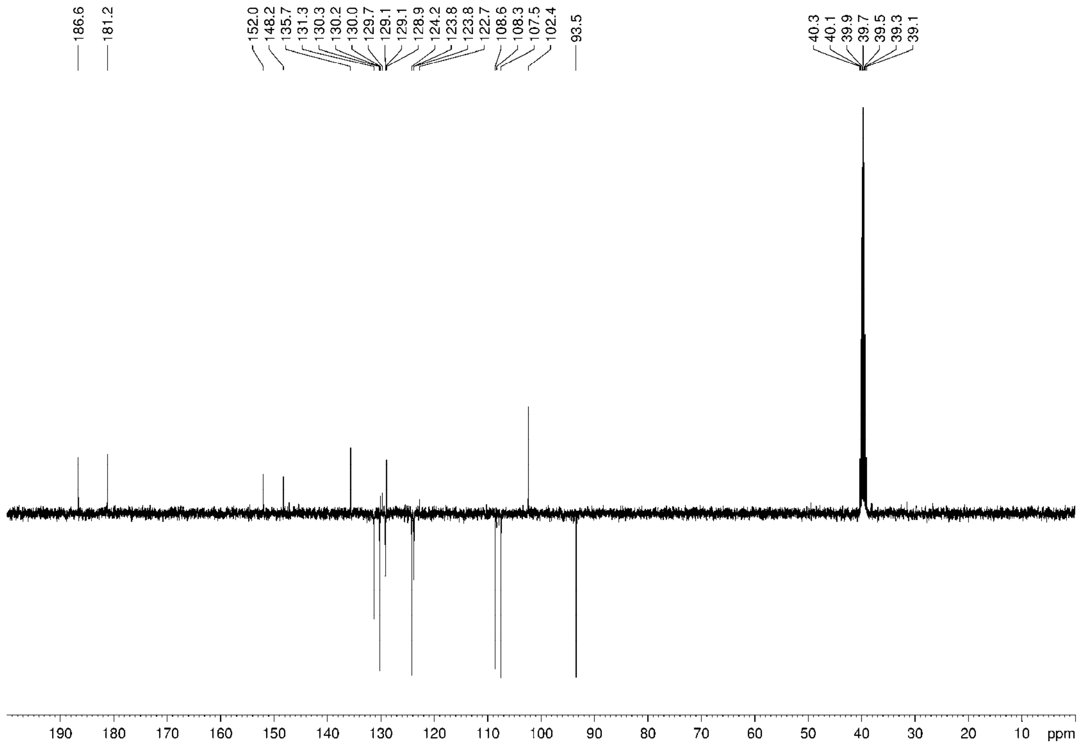

186.6
181.2
152.0 148.2 135.7 131.3 130.3 130.2 130.0 129.7 129.1 129.1 128.9 124.2 123.8 123.8 122.7 108.6 108.3 107.5 102.4
93.5
40.3 40.1 39.9 39.7 39.5 39.3 39.1
190 180 170 160 150 140 130 120 110 100 90 80 70 60 50 40 30 20 10 ppm

anle236B

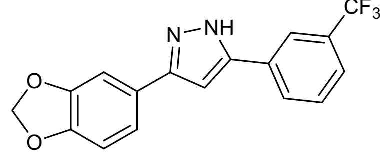
N
NH
CF3
O
O

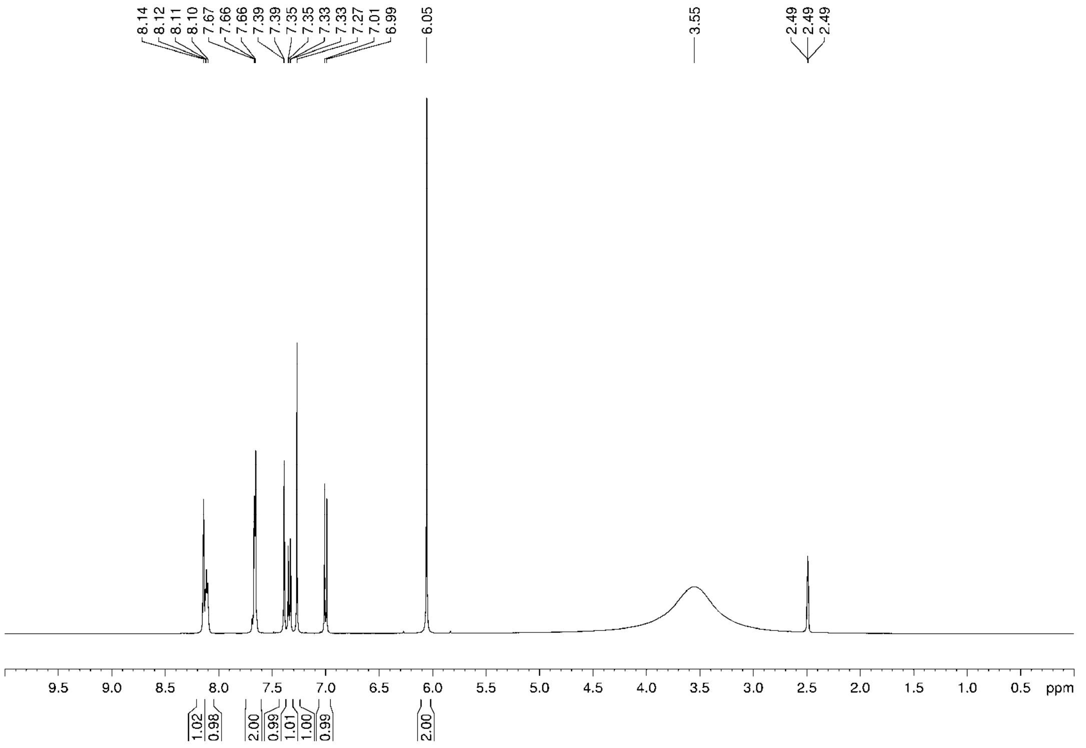
8.14
8.12
8.11
8.10
7.67
7.66
7.66
7.39
7.39
7.35
7.35
7.33
7.33
7.27
7.01
6.99
6.05
3.55
2.49
2.49
2.49
9.5
9.0
8.5
8.0
7.5
7.0
6.5
6.0
5.5
5.0
4.5
4.0
3.5
3.0
2.5
2.0
1.5
1.0
0.5
ppm
1.02
0.98
2.00
0.99
1.01
1.00
0.99
2.00

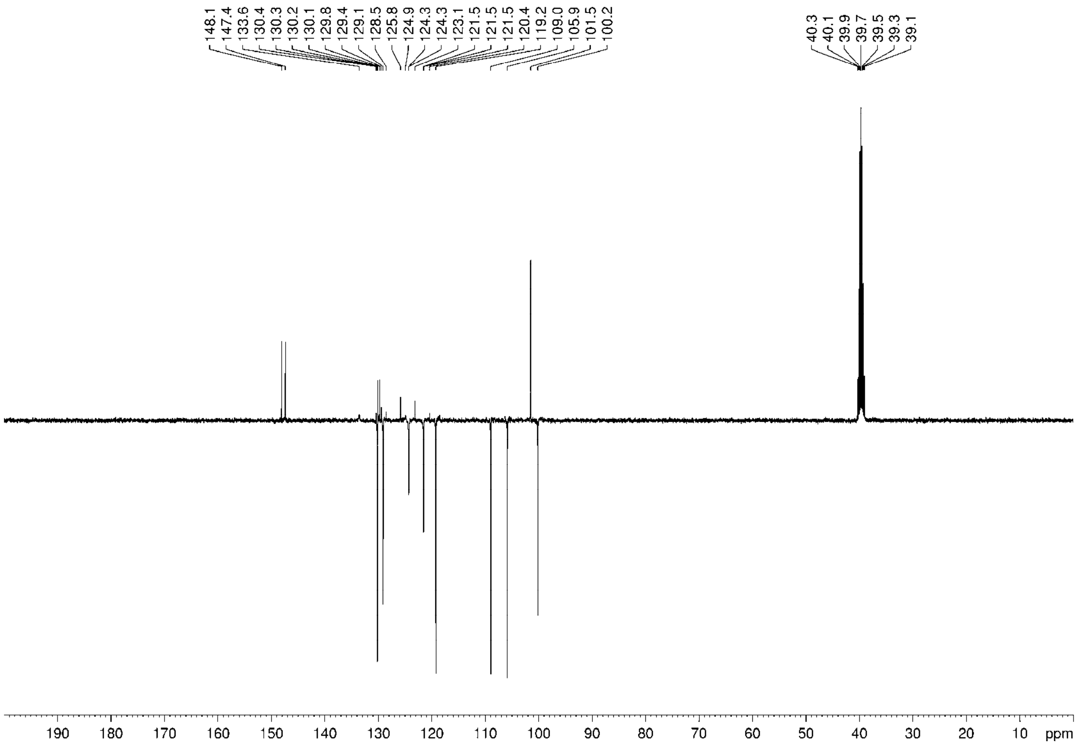
148.1
147.4
133.6
130.4
130.3
130.2
130.1
129.8
129.4
129.1
128.5
125.8
124.9
124.3
124.3
123.1
121.5
121.5
120.4
119.2
109.0
105.9
101.5
100.2
40.3
40.1
39.9
39.7
39.5
39.3
39.1
190
180
170
160
150
140
130
120
110
100
90
80
70
60
50
40
30
20
10
ppm

anle236C

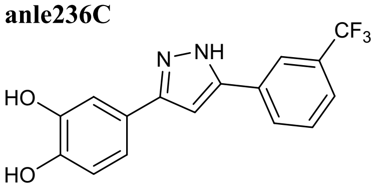
CF3
N–NH
HO
HO

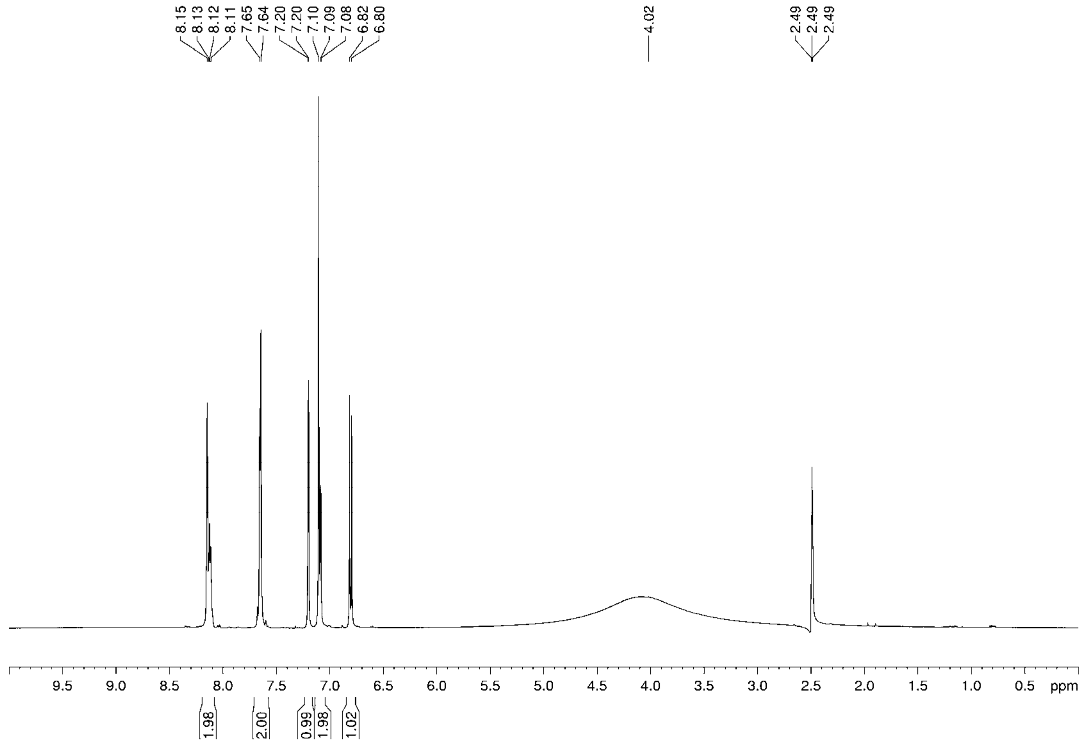
8.15
8.13
8.12
8.11
7.65
7.64
7.20
7.20
7.10
7.09
7.08
6.82
6.80
4.02
2.49
2.49
2.49
9.5
9.0
8.5
8.0
7.5
7.0
6.5
6.0
5.5
5.0
4.5
4.0
3.5
3.0
2.5
2.0
1.5
1.0
0.5
ppm
1.98
2.00
0.99
1.98
1.02

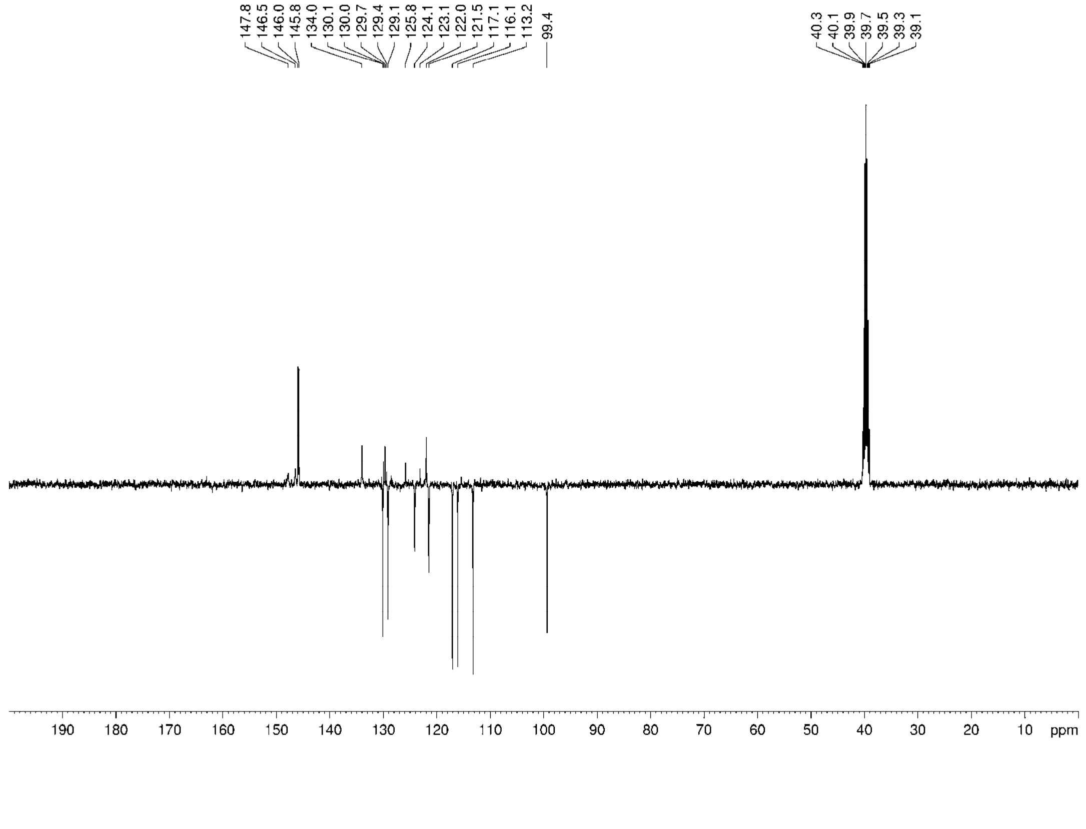
147.8
146.5
146.0
145.8
134.0
130.1
130.0
129.7
129.4
129.1
125.8
124.1
123.1
122.0
121.5
117.1
116.1
113.2
99.4
40.3
40.1
39.9
39.7
39.5
39.3
39.1
190
180
170
160
150
140
130
120
110
100
90
80
70
60
50
40
30
20
10
ppm

anle246A

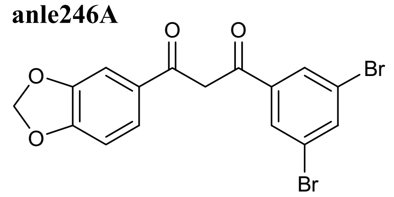
O
O
O
O
Br
Br

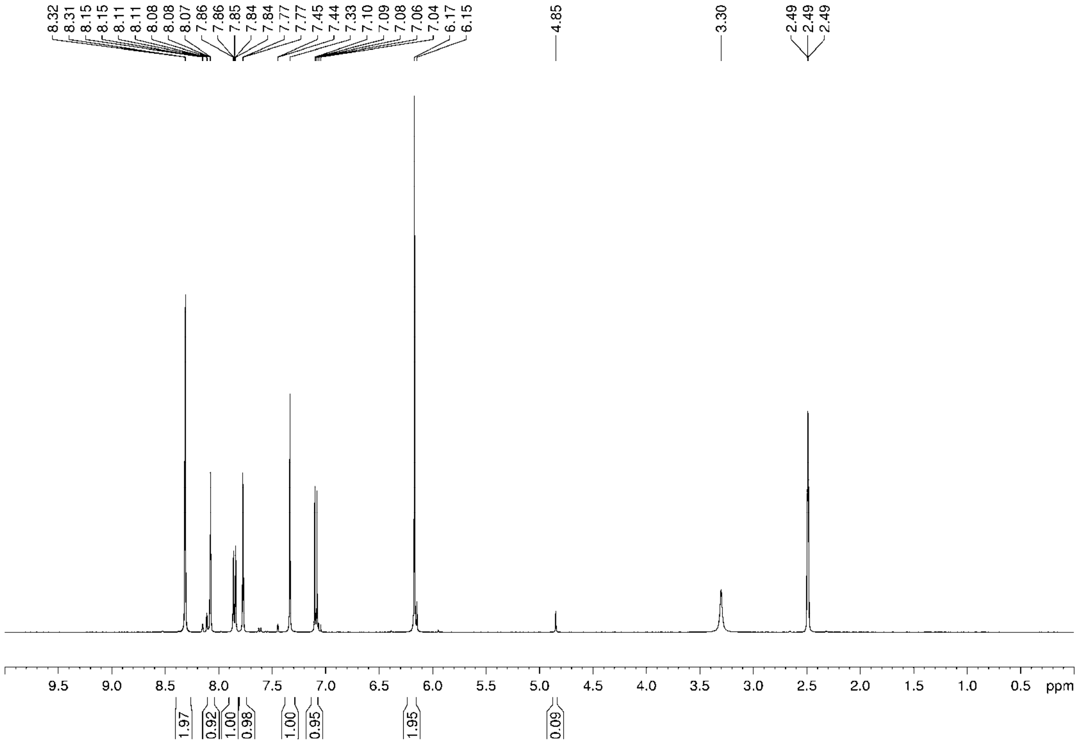
8.32
8.31
8.15
8.15
8.11
8.11
8.08
8.07
7.86
7.86
7.85
7.84
7.84
7.77
7.77
7.45
7.44
7.33
7.10
7.09
7.08
7.06
7.04
6.17
6.15
4.85
3.30
2.49
2.49
2.49
9.5 9.0 8.5 8.0 7.5 7.0 6.5 6.0 5.5 5.0 4.5 4.0 3.5 3.0 2.5 2.0 1.5 1.0 0.5 ppm
1.97
0.92
1.00
0.98
1.00
0.95
1.95
0.09

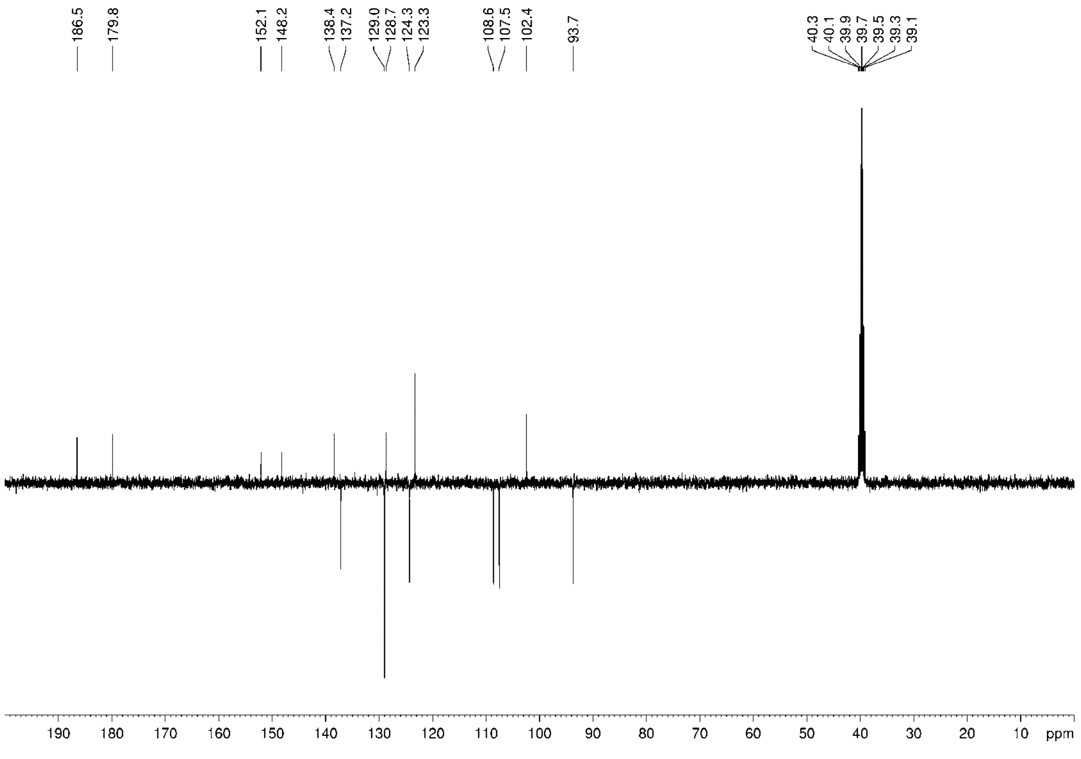
186.5
179.8
152.1
148.2
138.4
137.2
129.0
128.7
124.3
123.3
108.6
107.5
102.4
93.7
40.3
40.1
39.9
39.7
39.5
39.3
39.1
190 180 170 160 150 140 130 120 110 100 90 80 70 60 50 40 30 20 10 ppm

anle246B

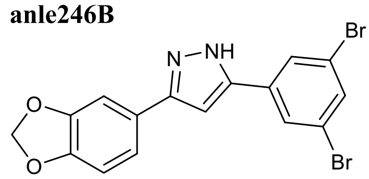
N
NH
O
O
Br
Br

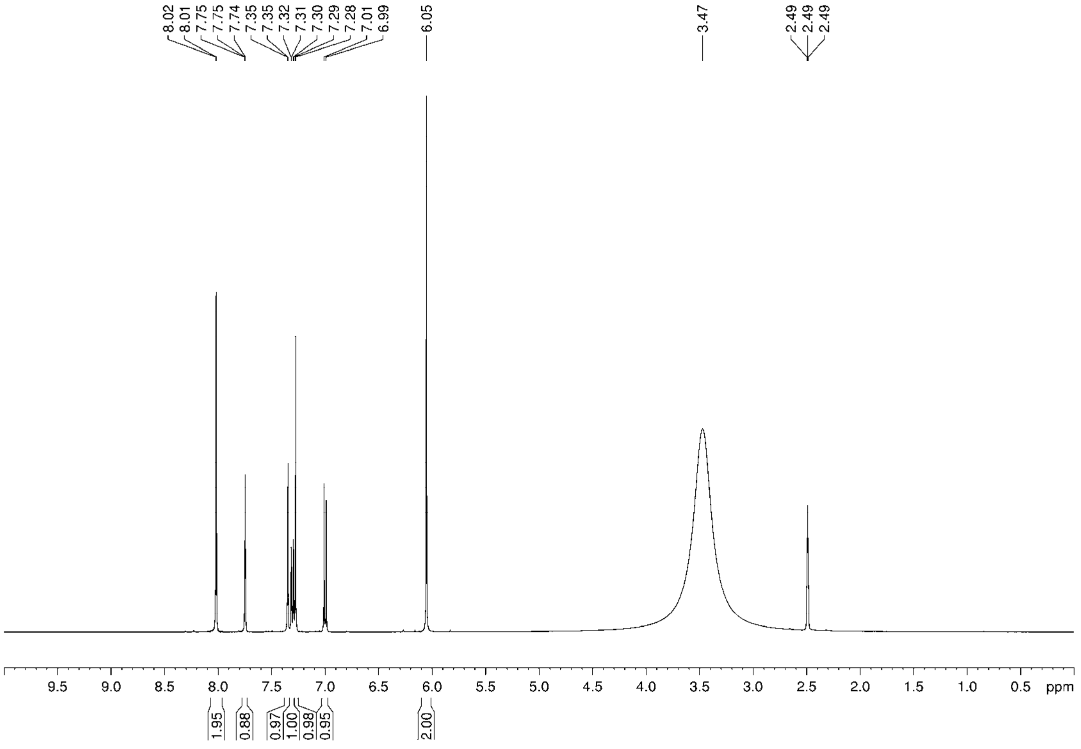
8.02
8.01
7.75
7.75
7.74
7.35
7.35
7.32
7.31
7.30
7.29
7.28
7.01
6.99
6.05
3.47
2.49
2.49
2.49
9.5 9.0 8.5 8.0 7.5 7.0 6.5 6.0 5.5 5.0 4.5 4.0 3.5 3.0 2.5 2.0 1.5 1.0 0.5 ppm
1.95
0.88
0.97
1.00
0.98
0.95
2.00

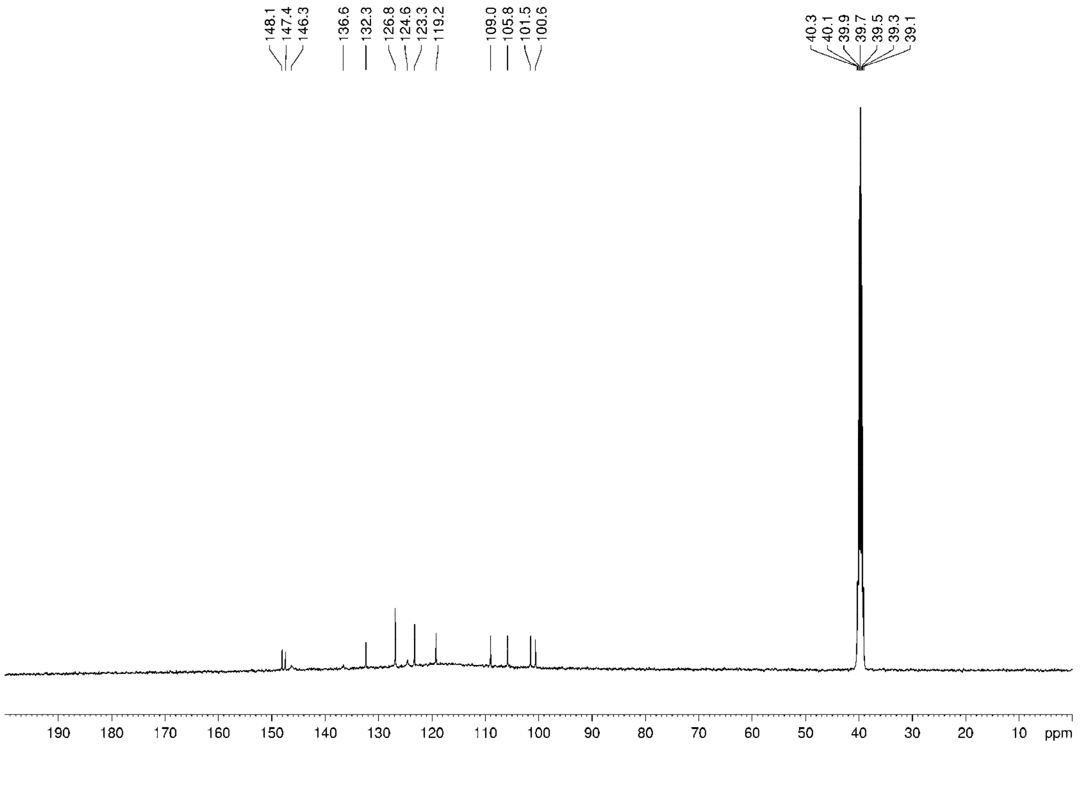
148.1
147.4
146.3
136.6
132.3
126.8
124.6
123.3
119.2
109.0
105.8
101.5
100.6
40.3
40.1
39.9
39.7
39.5
39.3
39.1
190 180 170 160 150 140 130 120 110 100 90 80 70 60 50 40 30 20 10 ppm

anle253A

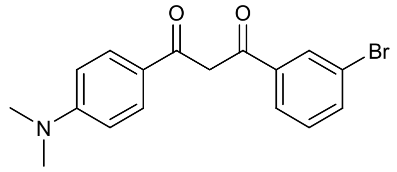
O
O
Br
N

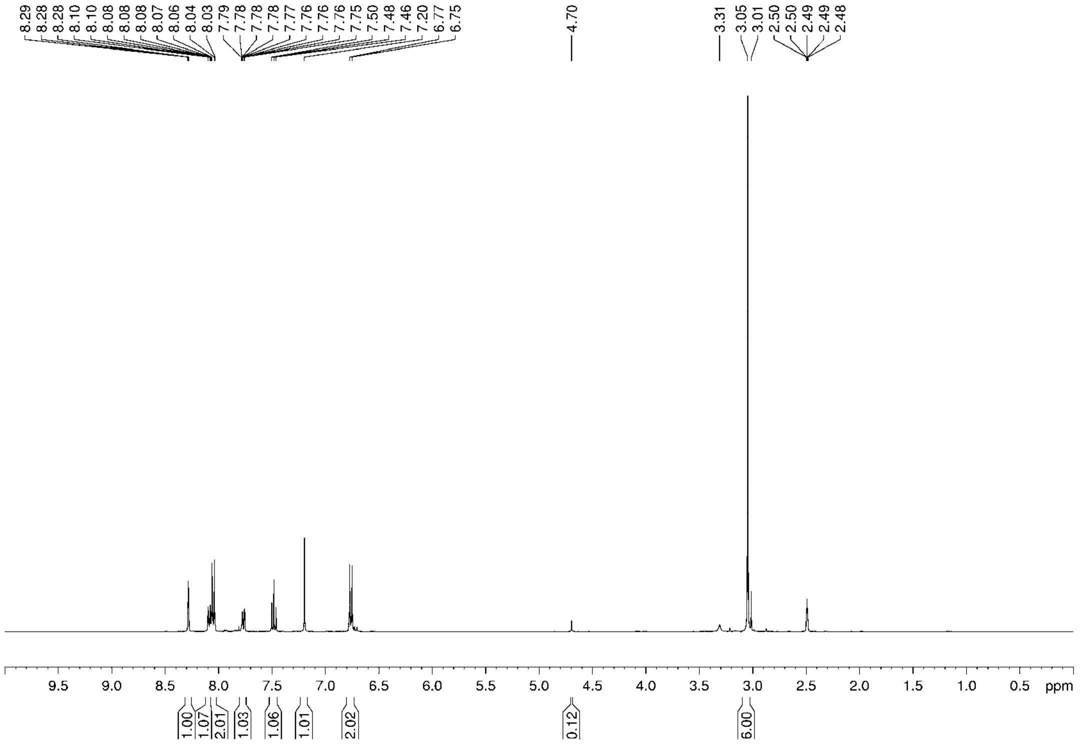
8.29
8.28
8.28
8.10
8.10
8.08
8.08
8.08
8.07
8.06
8.04
8.03
7.79
7.78
7.78
7.78
7.77
7.76
7.76
7.75
7.50
7.48
7.46
7.20
6.77
6.75
4.70
3.31
3.05
3.01
2.50
2.50
2.49
2.49
2.48
9.5 9.0 8.5 8.0 7.5 7.0 6.5 6.0 5.5 5.0 4.5 4.0 3.5 3.0 2.5 2.0 1.5 1.0 0.5 ppm
1.00
1.07
2.01
1.03
1.06
1.01
2.02
0.12
6.00

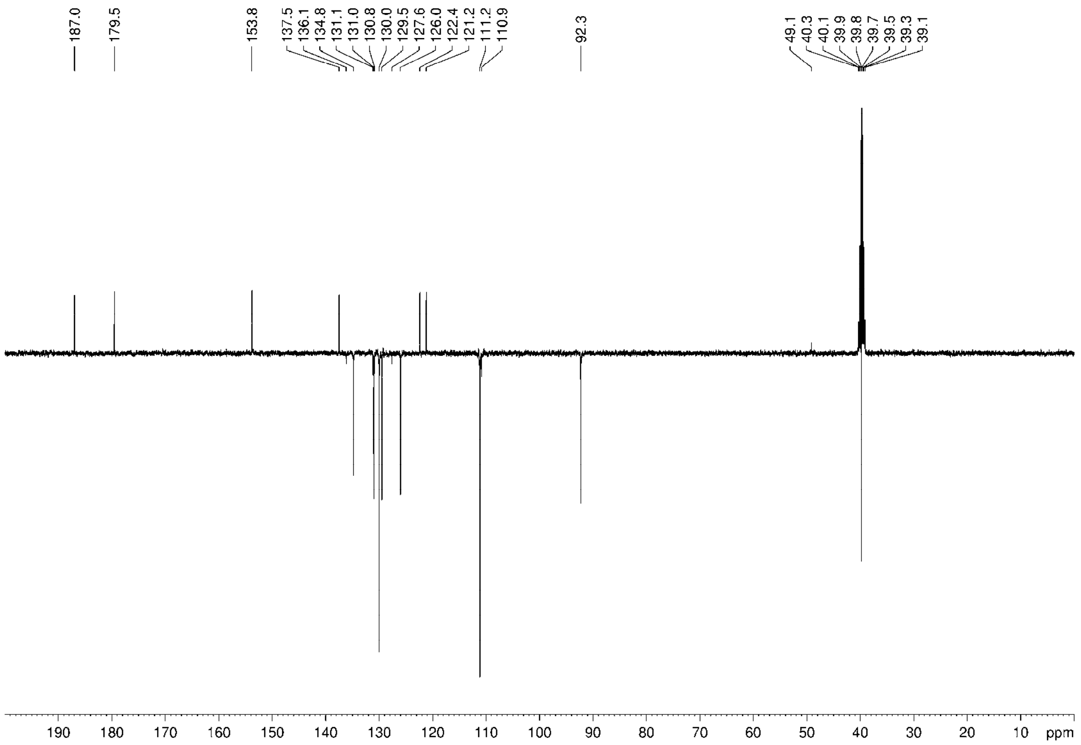
187.0
179.5
153.8
137.5
136.1
134.8
131.1
131.0
130.8
130.0
129.5
127.6
126.0
122.4
121.2
111.2
110.9
92.3
49.1
40.3
40.1
39.9
39.8
39.7
39.5
39.3
39.1
190 180 170 160 150 140 130 120 110 100 90 80 70 60 50 40 30 20 10 ppm

anle253B

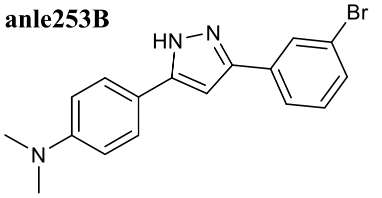

Br
HN
N
N

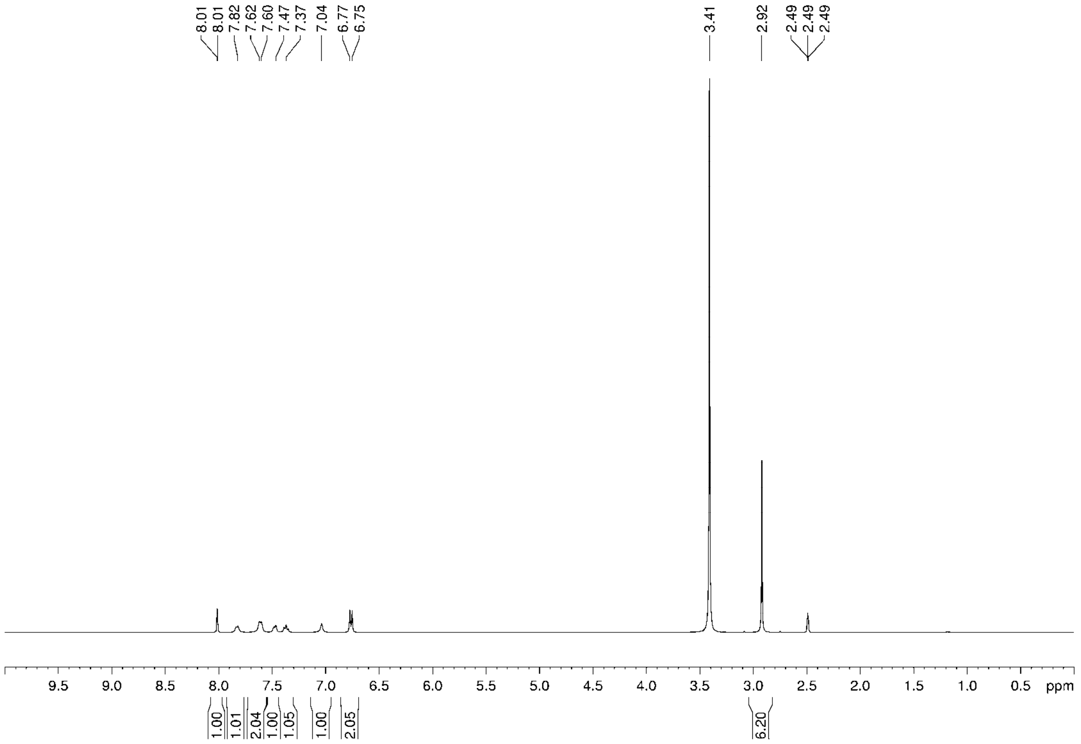

8.01
8.01
7.82
7.62
7.60
7.47
7.37
7.04
6.77
6.75
3.41
2.92
2.49
2.49
2.49
9.5 9.0 8.5 8.0 7.5 7.0 6.5 6.0 5.5 5.0 4.5 4.0 3.5 3.0 2.5 2.0 1.5 1.0 0.5 ppm
1.00
1.01
2.04
1.00
1.05
1.00
2.05
6.20

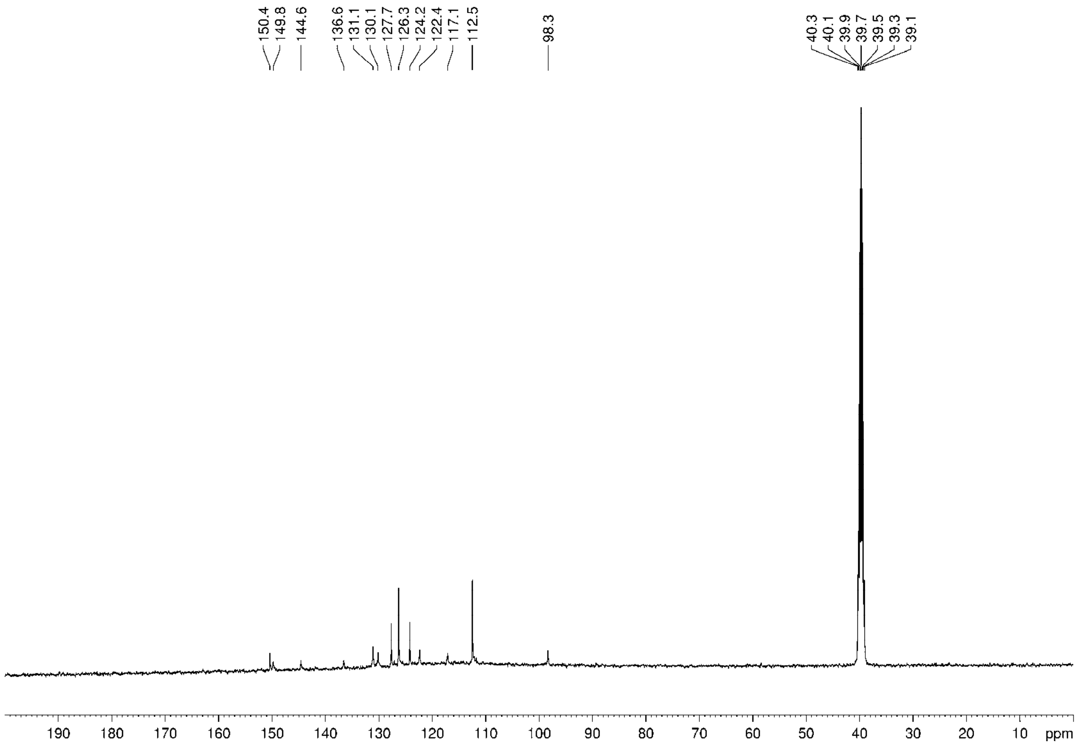

150.4
149.8
144.6
136.6
131.1
130.1
127.7
126.3
124.2
122.4
117.1
112.5
98.3
40.3
40.1
39.9
39.7
39.5
39.3
39.1
190 180 170 160 150 140 130 120 110 100 90 80 70 60 50 40 30 20 10 ppm

anle254A

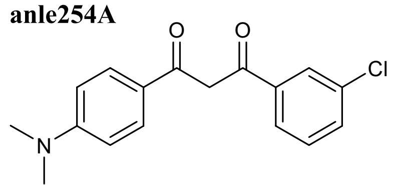

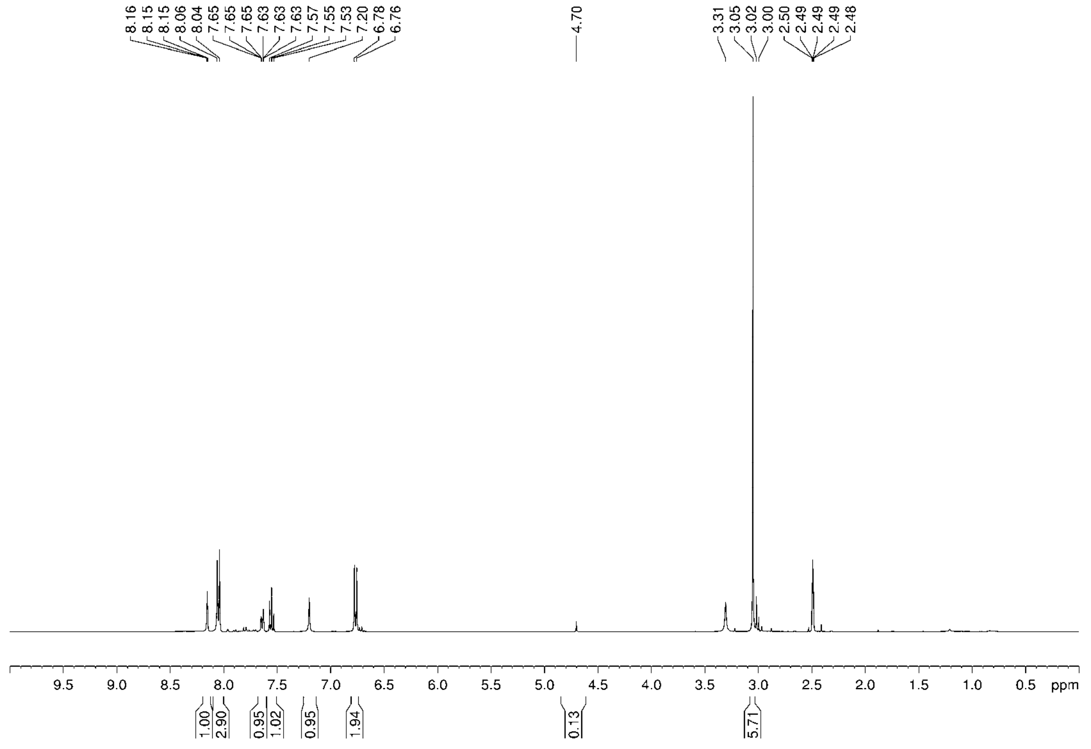

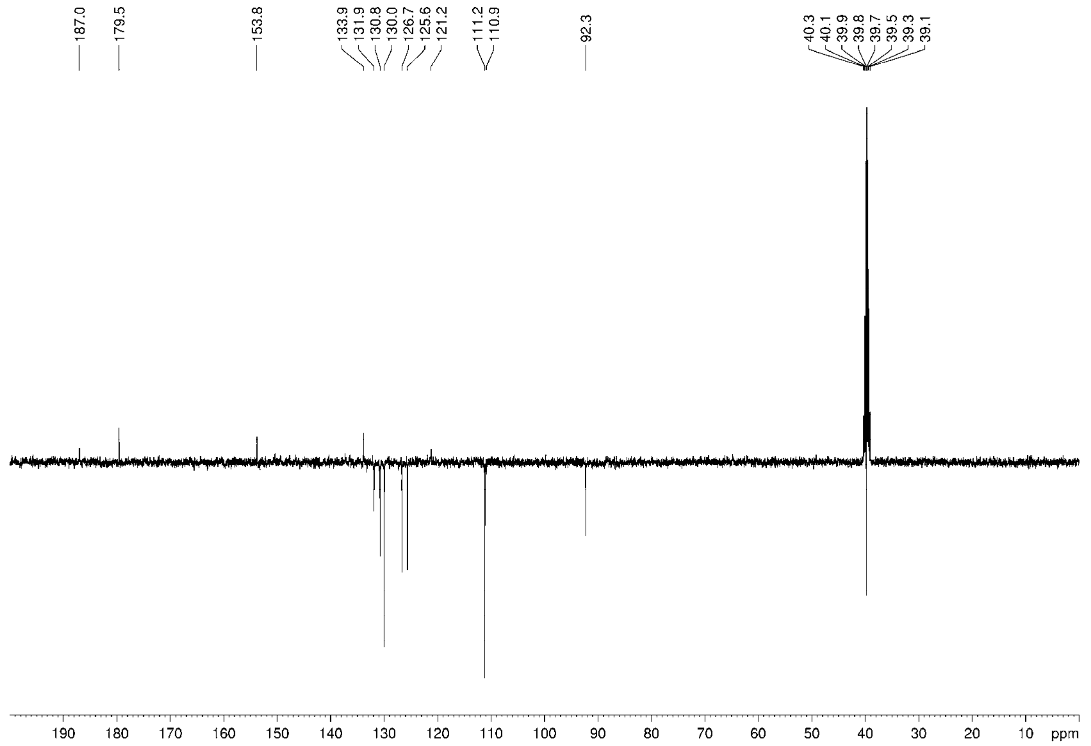

anle254B

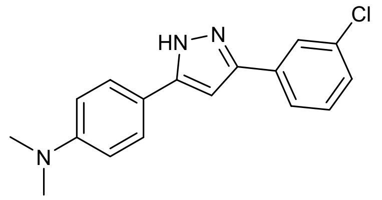

HN
N
Cl
N

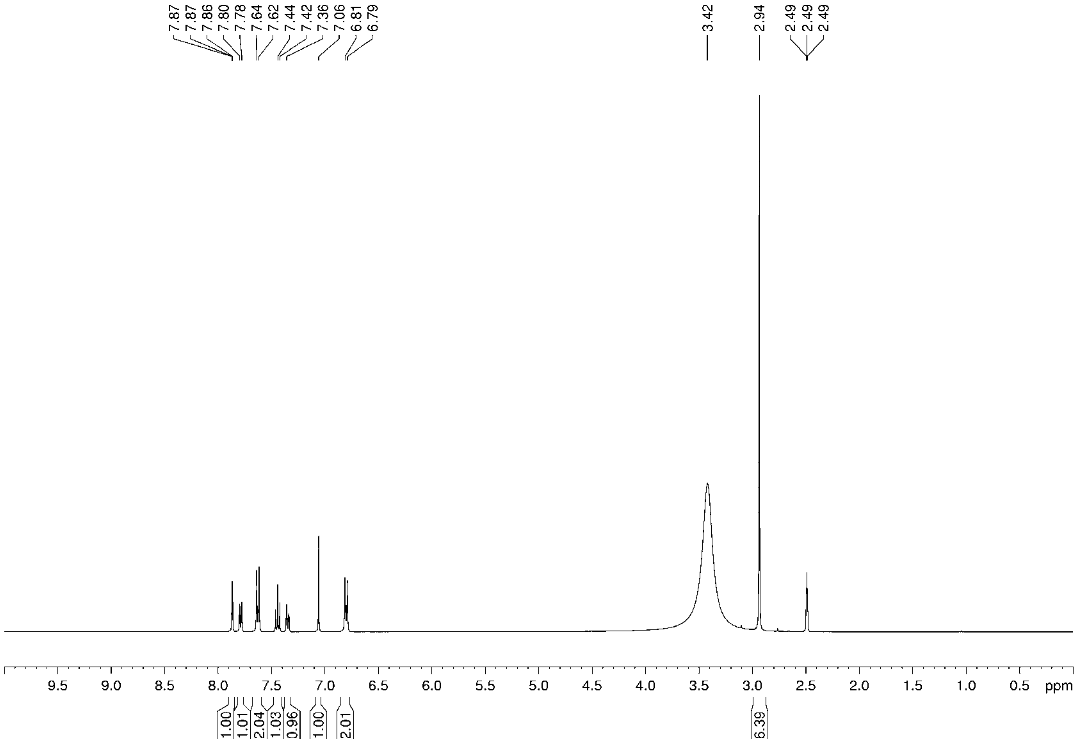

7.87
7.87
7.86
7.80
7.78
7.64
7.62
7.44
7.42
7.36
7.06
6.81
6.79
3.42
2.94
2.49
2.49
2.49
9.5
9.0
8.5
8.0
7.5
7.0
6.5
6.0
5.5
5.0
4.5
4.0
3.5
3.0
2.5
2.0
1.5
1.0
0.5
ppm
1.00
1.01
2.04
1.03
0.96
1.00
2.01
6.39

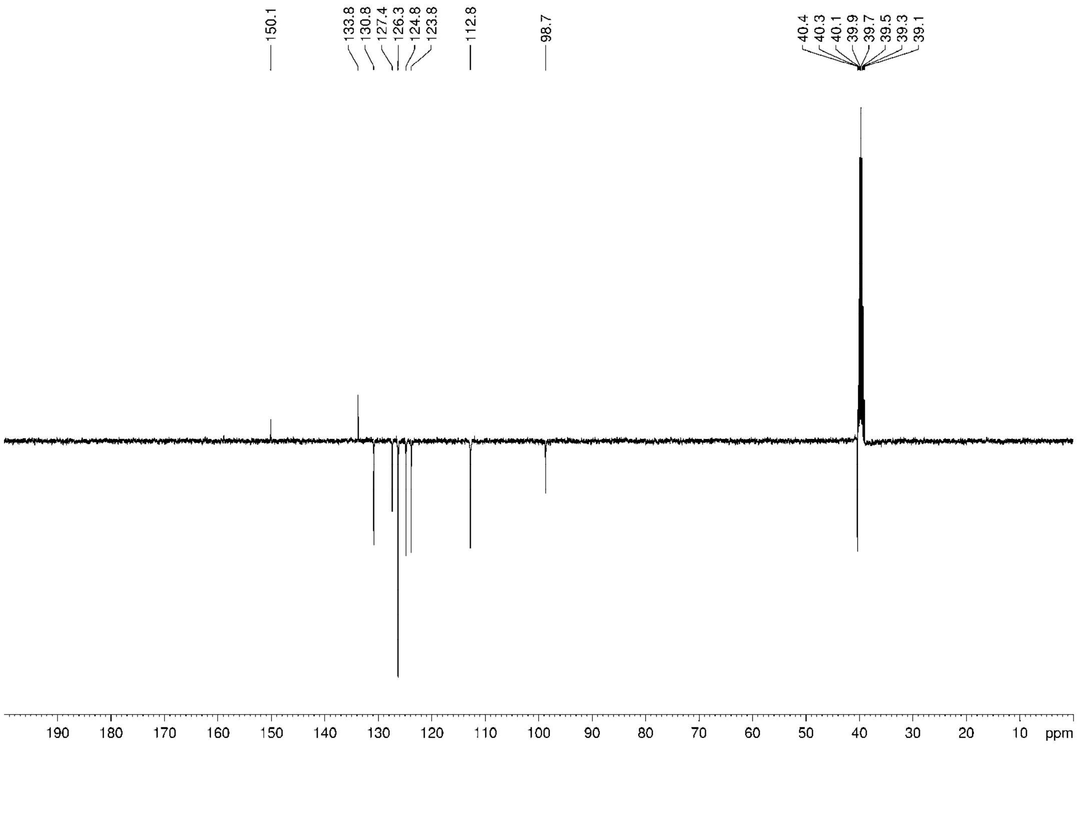

150.1
133.8
130.8
127.4
126.3
124.8
123.8
112.8
98.7
40.4
40.3
40.1
39.9
39.7
39.5
39.3
39.1
190
180
170
160
150
140
130
120
110
100
90
80
70
60
50
40
30
20
10
ppm

anle270

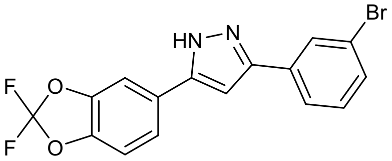

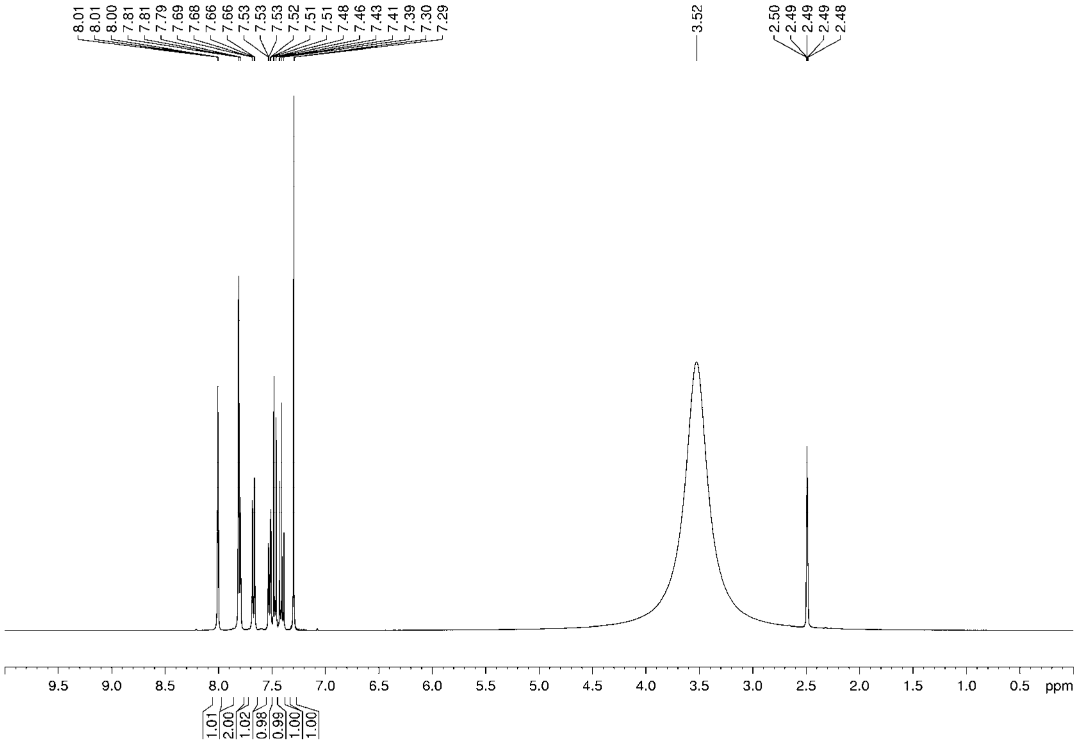

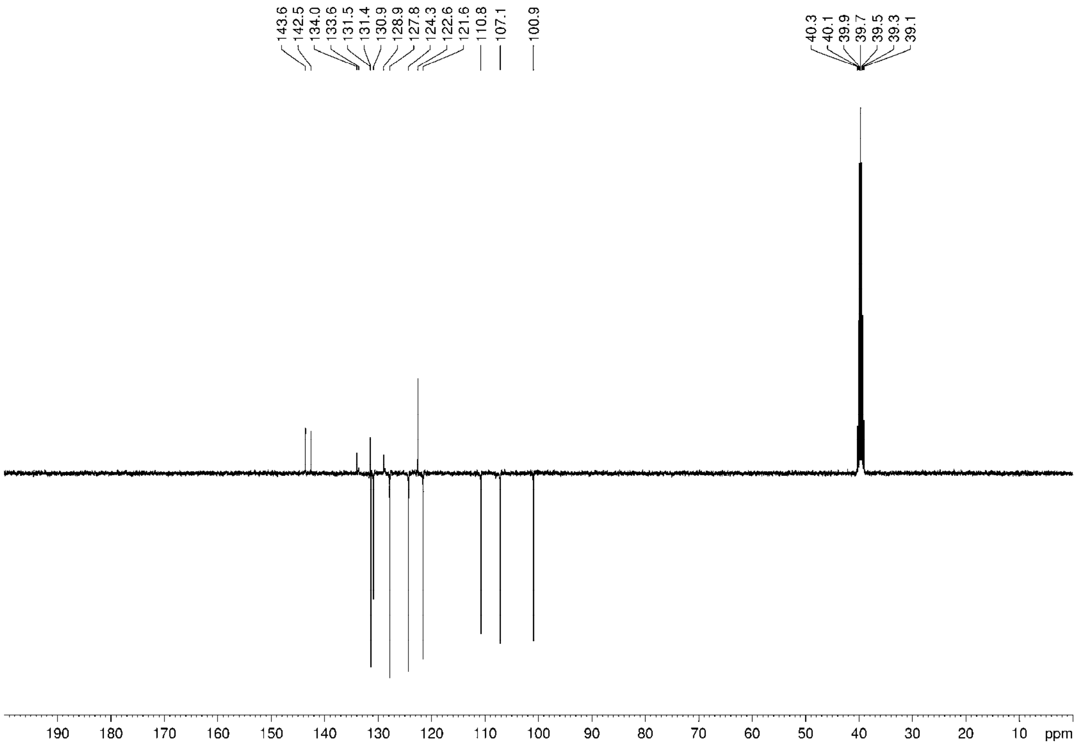

sery85

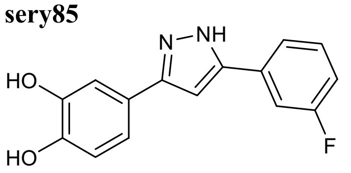

HO
HO
N
NH
F

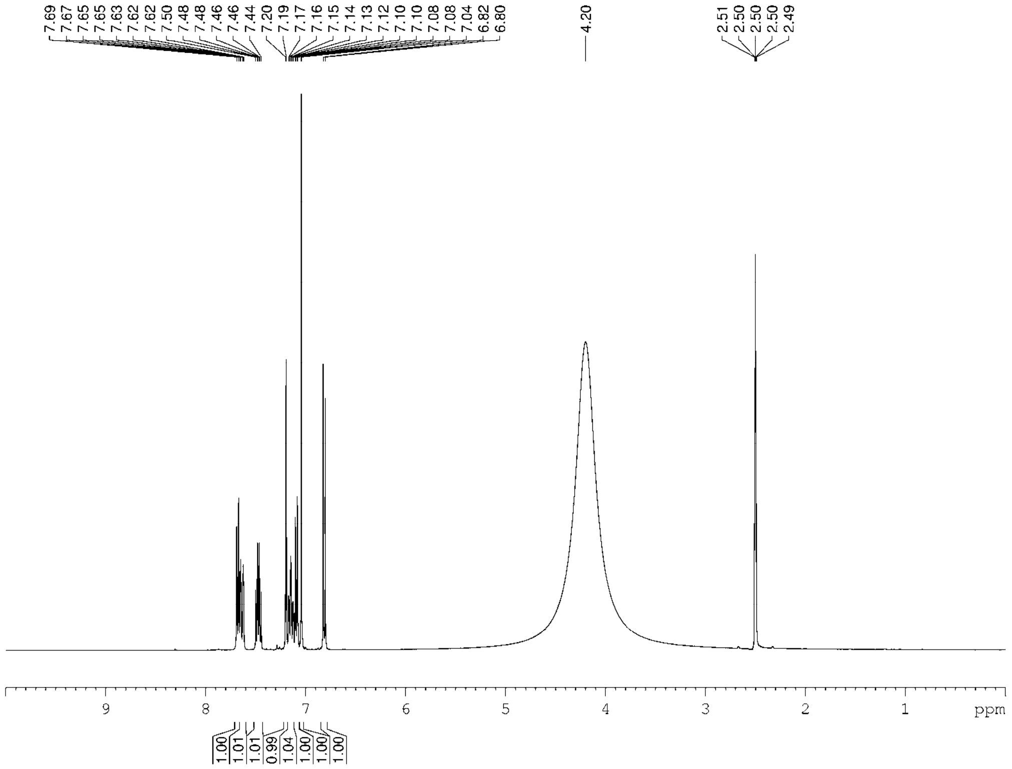

7.69 7.67 7.65 7.63 7.62 7.62 7.50 7.48 7.48 7.46 7.46 7.44 7.20 7.19 7.17 7.16 7.15 7.14 7.13 7.12 7.10 7.10 7.08 7.08 7.04 6.82 6.80
4.20
2.51 2.50 2.50 2.50 2.49
9 8 7 6 5 4 3 2 1 ppm
1.00 1.01 1.01 0.99 1.04 1.00 1.00 1.00

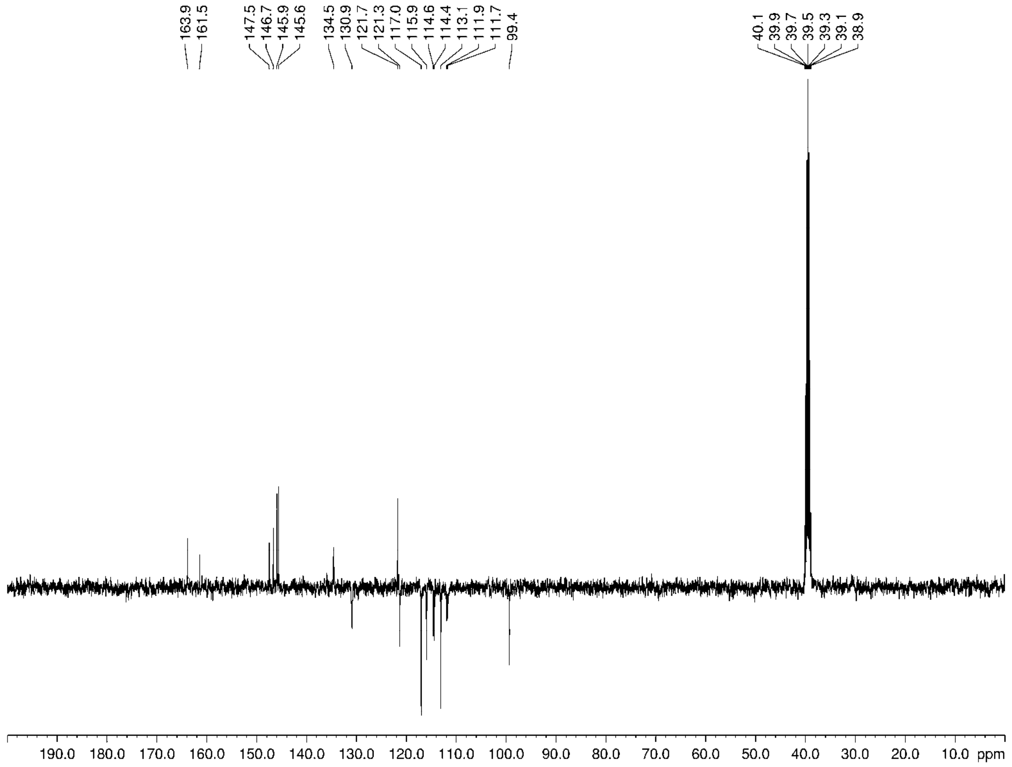

163.9 161.5
147.5 146.7 145.9 145.6
134.5 130.9 121.7 121.3 117.0 115.9 114.6 114.4 113.1 111.9 111.7 99.4
40.1 39.9 39.7 39.5 39.3 39.1 38.9
190.0 180.0 170.0 160.0 150.0 140.0 130.0 120.0 110.0 100.0 90.0 80.0 70.0 60.0 50.0 40.0 30.0 20.0 10.0 ppm

sery95

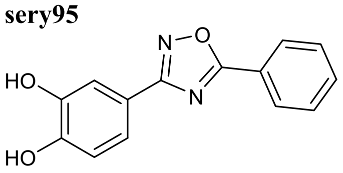

HO
HO
N
O
N

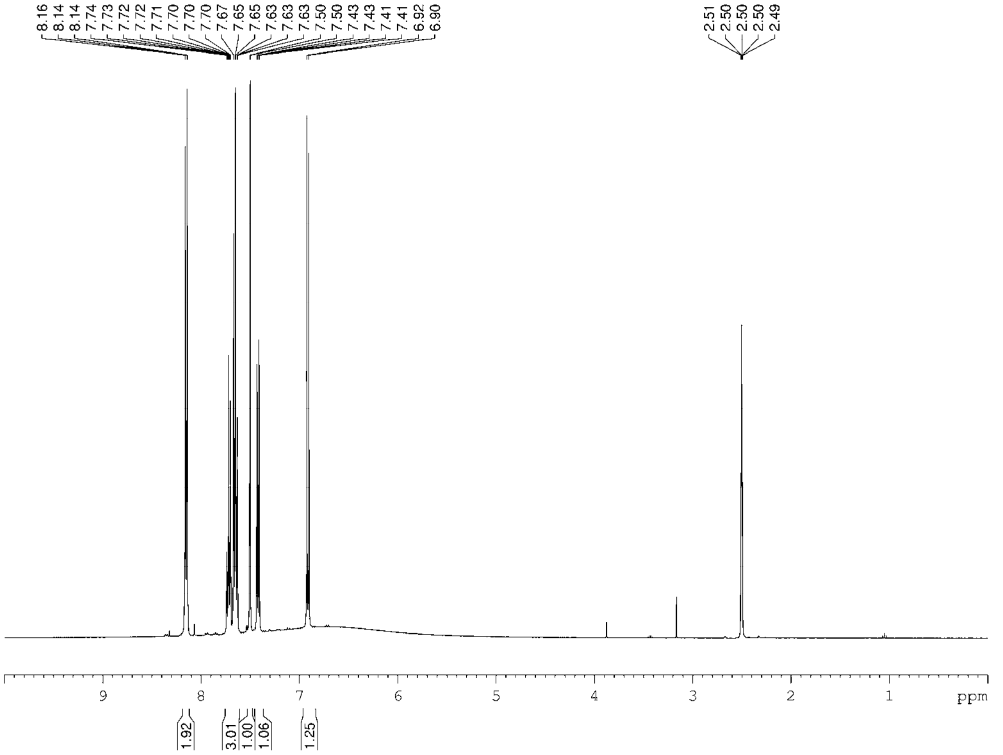

8.16
8.14
8.14
7.74
7.73
7.72
7.72
7.71
7.70
7.70
7.70
7.67
7.65
7.65
7.63
7.63
7.63
7.50
7.50
7.43
7.43
7.41
7.41
6.92
6.90
2.51
2.50
2.50
2.50
2.49
9
8
7
6
5
4
3
2
1
ppm
1.92
3.01
1.00
1.06
1.25

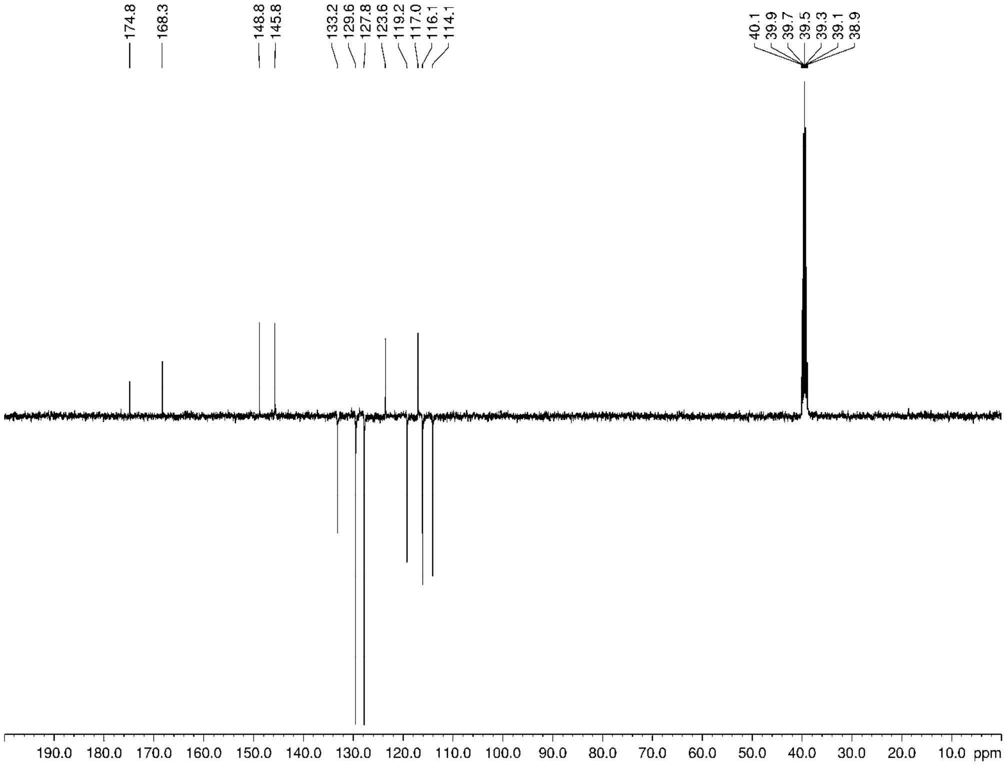

174.8
168.3
148.8
145.8
133.2
129.6
127.8
123.6
119.2
117.0
116.1
114.1
40.1
39.9
39.7
39.5
39.3
39.1
38.9
190.0
180.0
170.0
160.0
150.0
140.0
130.0
120.0
110.0
100.0
90.0
80.0
70.0
60.0
50.0
40.0
30.0
20.0
10.0
ppm

sery97

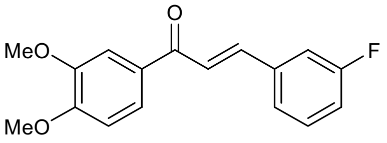
O
MeO
MeO
F

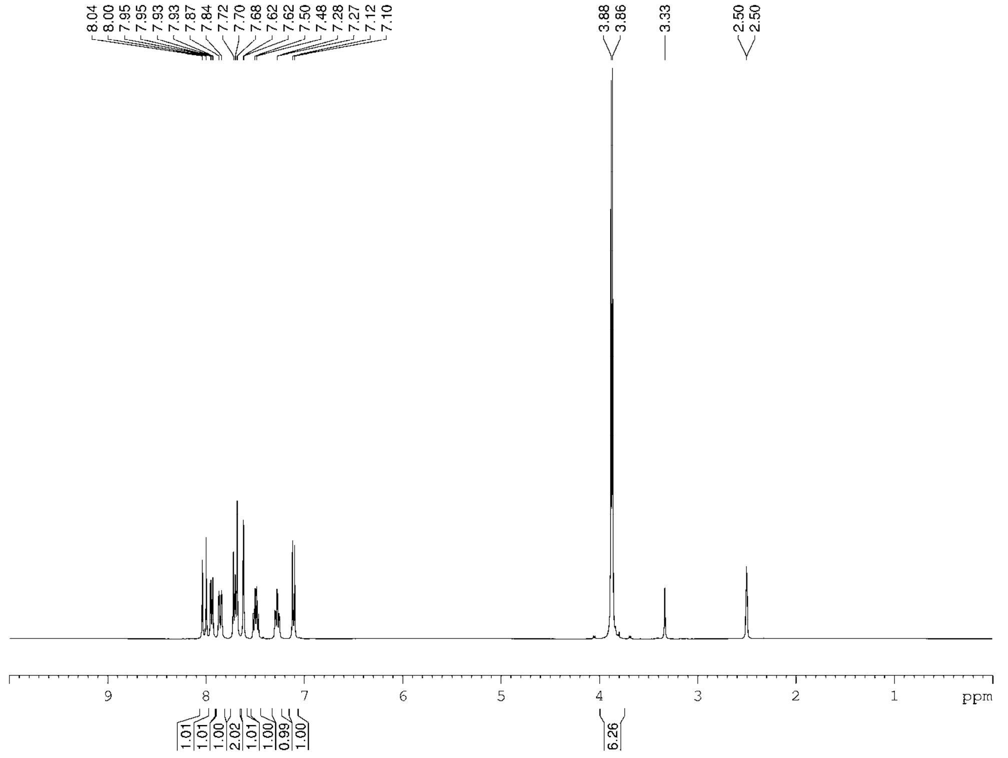
8.04 8.00 7.95 7.95 7.93 7.93 7.87 7.84 7.72 7.70 7.68 7.62 7.62 7.50 7.48 7.28 7.27 7.12 7.10
3.88 3.86
3.33
2.50 2.50
9 8 7 6 5 4 3 2 1 ppm
1.01 1.01 1.00 2.02 1.01 1.00 0.99 1.00
6.26

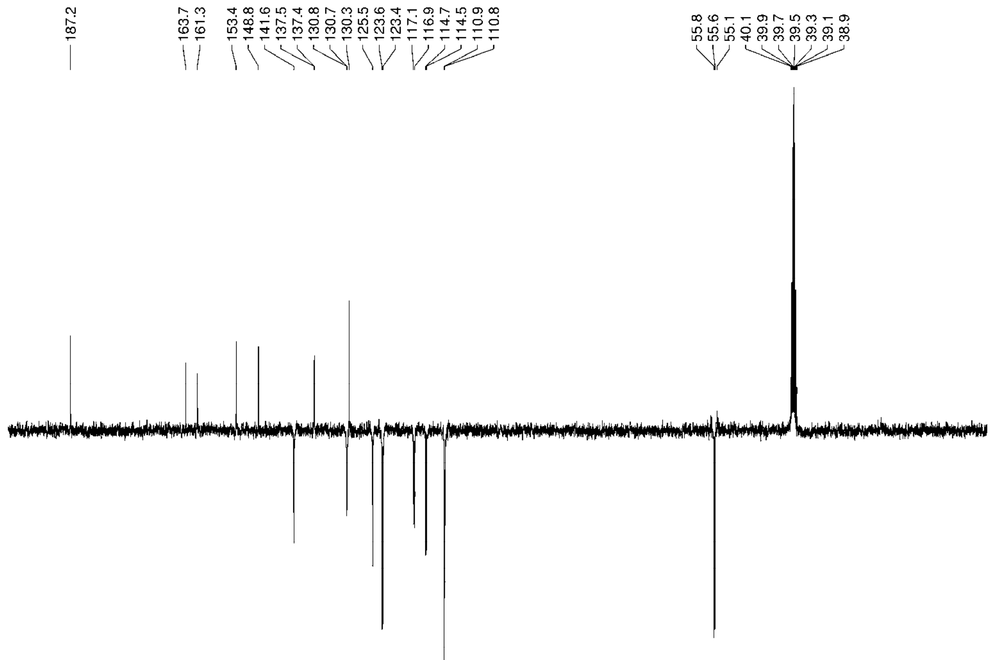
187.2
163.7 161.3
153.4 148.8 141.6 137.5 137.4 130.8 130.7 130.3 125.5 123.6 123.4 117.1 116.9 114.7 114.5 110.9 110.8
55.8 55.6 55.1 40.1 39.9 39.7 39.5 39.3 39.1 38.9
190.0 180.0 170.0 160.0 150.0 140.0 130.0 120.0 110.0 100.0 90.0 80.0 70.0 60.0 50.0 40.0 30.0 20.0 10.0 ppm

sery102

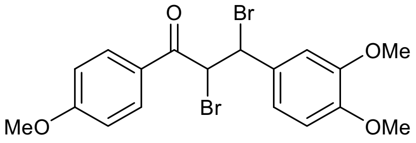

O
Br
OMe
Br
MeO
OMe

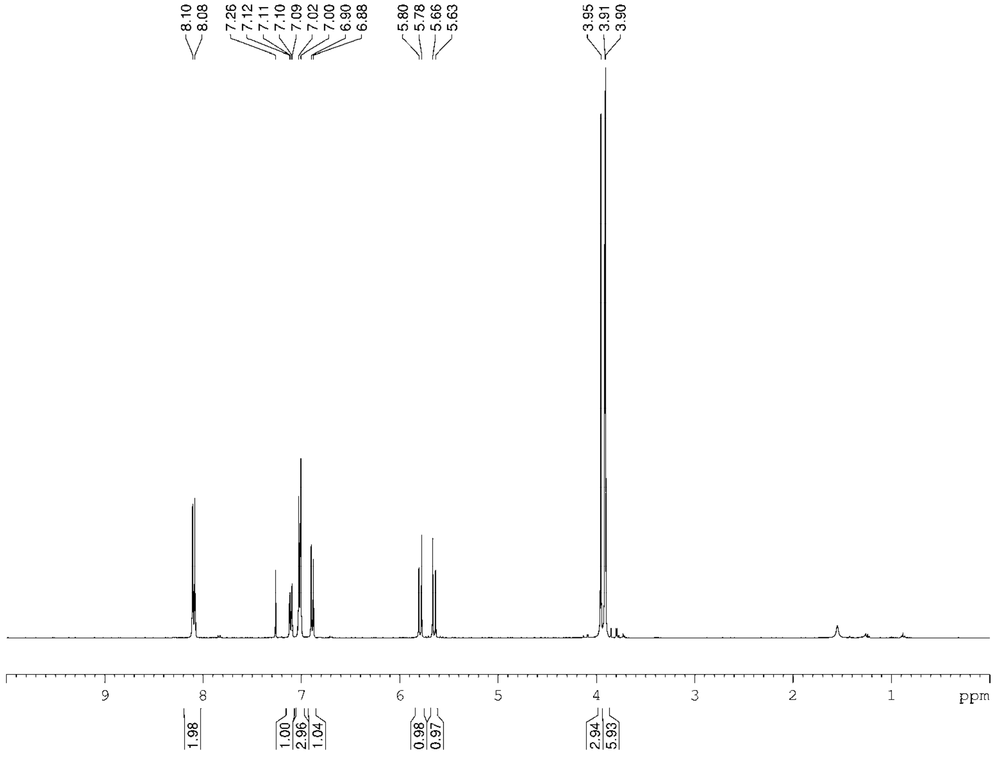

8.10 8.08
7.26 7.12 7.11 7.10 7.09 7.02 7.00 6.90 6.88
5.80 5.78 5.66 5.63
3.95 3.91 3.90
9 8 7 6 5 4 3 2 1 ppm
1.98
1.00 2.96 1.04
0.98 0.97
2.94 5.93

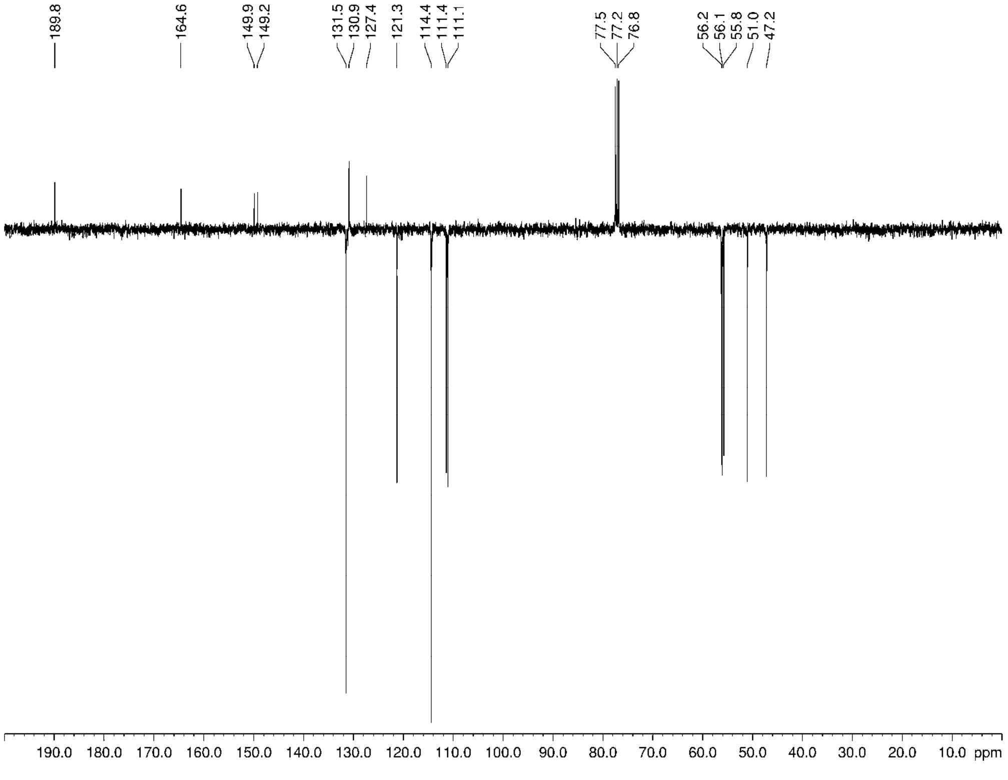

189.8
164.6
149.9 149.2
131.5 130.9 127.4
121.3
114.4 111.4 111.1
77.5 77.2 76.8
56.2 56.1 55.8 51.0 47.2
190.0 180.0 170.0 160.0 150.0 140.0 130.0 120.0 110.0 100.0 90.0 80.0 70.0 60.0 50.0 40.0 30.0 20.0 10.0 ppm

sery103

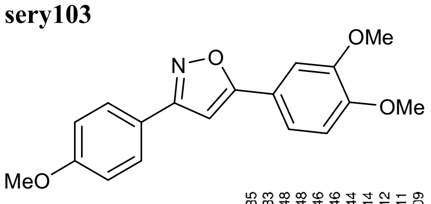
N–O
OMe
OMe
MeO

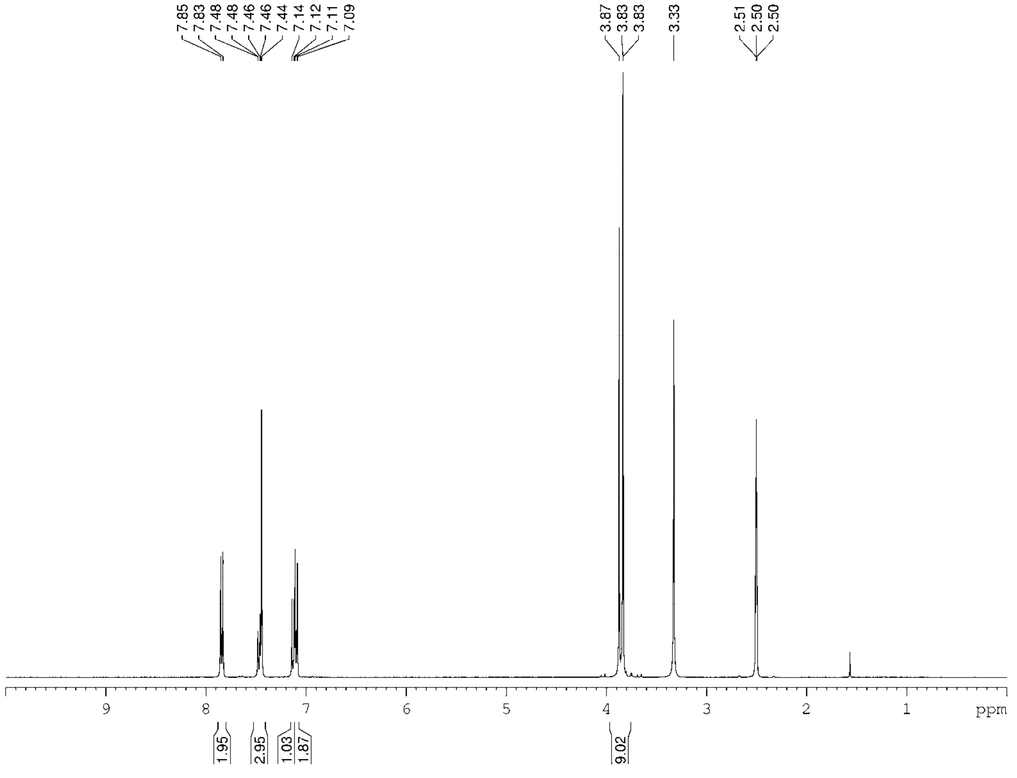
7.85
7.83
7.48
7.48
7.46
7.46
7.44
7.14
7.12
7.11
7.09
3.87
3.83
3.83
3.33
2.51
2.50
2.50
9 8 7 6 5 4 3 2 1 ppm
1.95
2.95
1.03
1.87
9.02

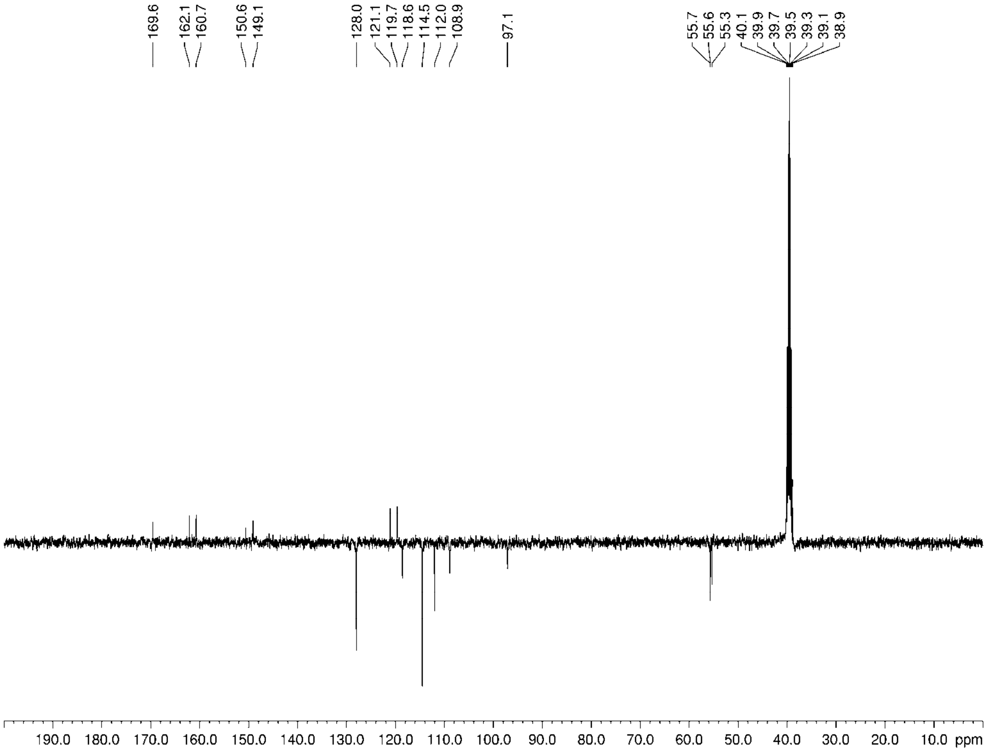
169.6
162.1
160.7
150.6
149.1
128.0
121.1
119.7
118.6
114.5
112.0
108.9
97.1
55.7
55.6
55.3
40.1
39.9
39.7
39.5
39.3
39.1
38.9
190.0 180.0 170.0 160.0 150.0 140.0 130.0 120.0 110.0 100.0 90.0 80.0 70.0 60.0 50.0 40.0 30.0 20.0 10.0 ppm

sery105

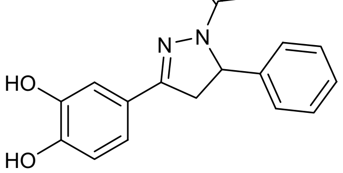

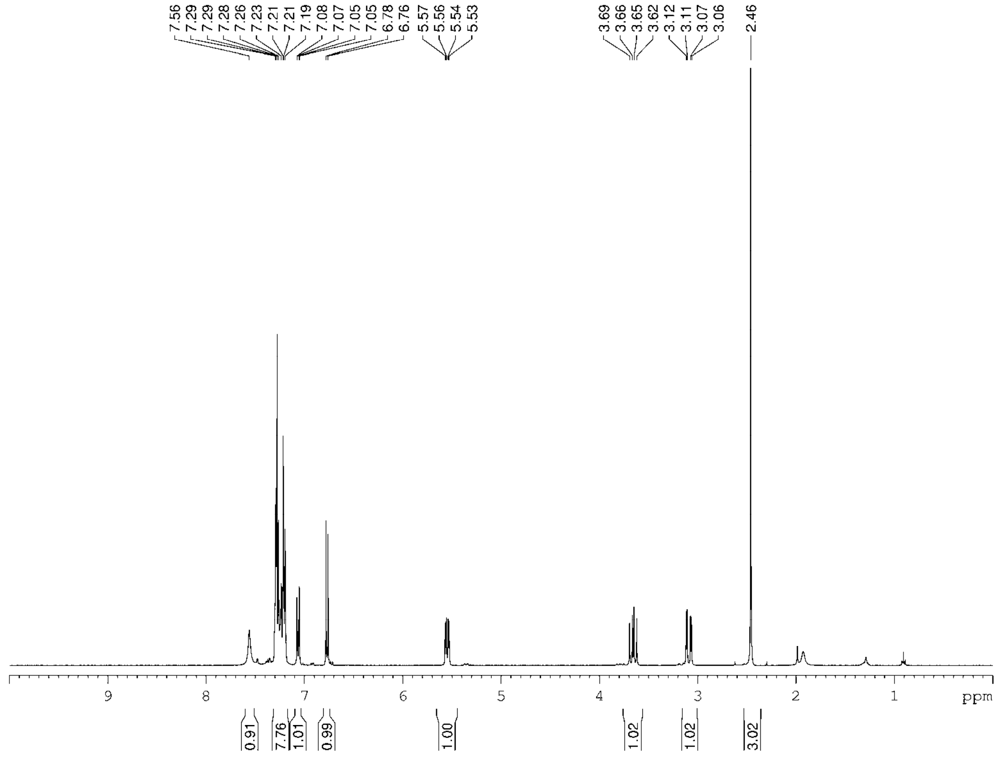
7.56 7.29 7.29 7.28 7.26 7.23 7.21 7.21 7.19 7.08 7.07 7.05 7.05 6.78 6.76 5.57 5.56 5.54 5.53
3.69 3.66 3.65 3.62 3.12 3.11 3.07 3.06
2.46
9 8 7 6 5 4 3 2 1 ppm
0.91 7.76 1.01 0.99 1.00 1.02 1.02 3.02

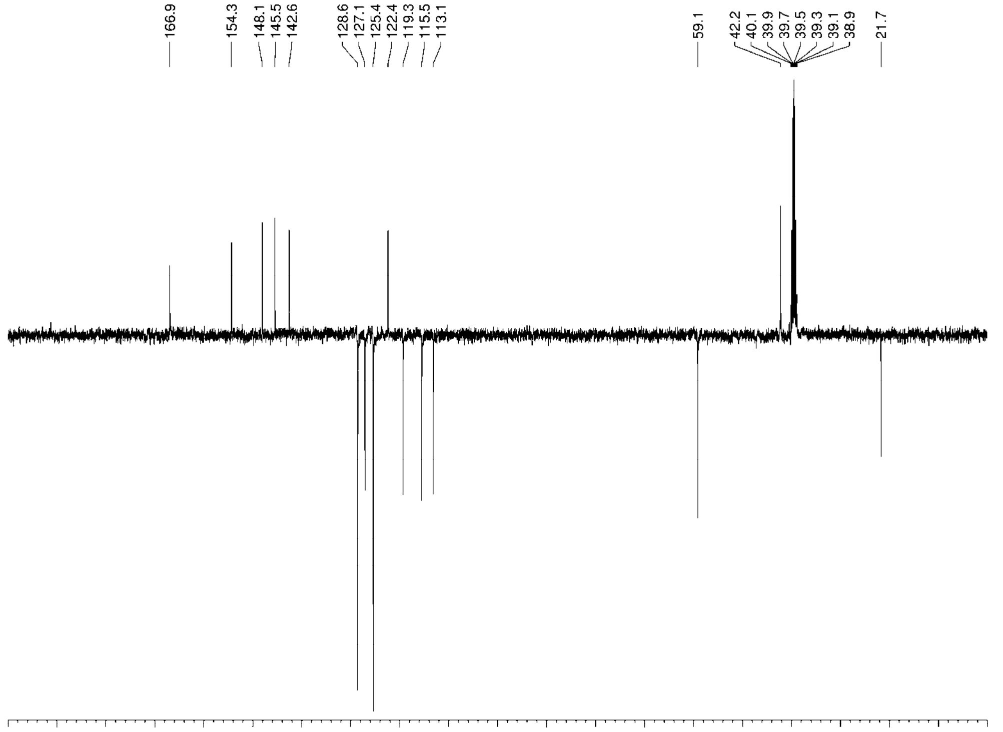
166.9
154.3 148.1 145.5 142.6
128.6 127.1 125.4 122.4 119.3 115.5 113.1
59.1
42.2 40.1 39.9 39.7 39.5 39.3 39.1 38.9
21.7
190.0 180.0 170.0 160.0 150.0 140.0 130.0 120.0 110.0 100.0 90.0 80.0 70.0 60.0 50.0 40.0 30.0 20.0 10.0 ppm

sery106

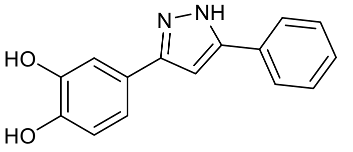
N–NH
HO
HO

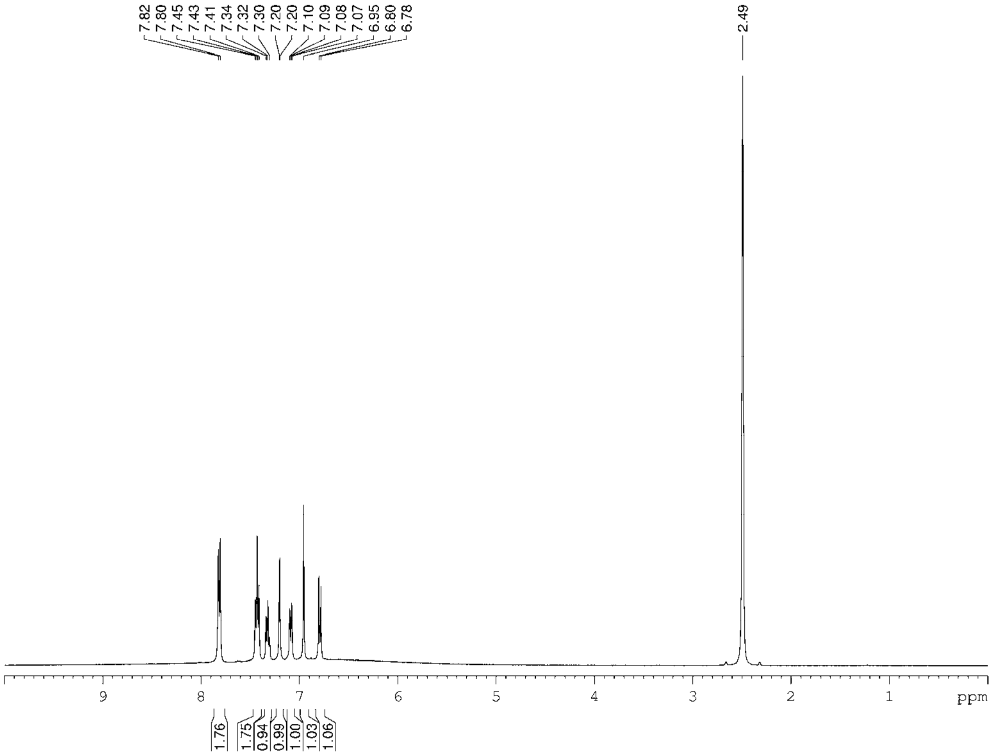
7.82 7.80 7.45 7.43 7.41 7.34 7.32 7.30 7.20 7.20 7.10 7.09 7.08 7.07 6.95 6.80 6.78
2.49
9 8 7 6 5 4 3 2 1 ppm
1.76 1.75 0.94 0.99 1.00 1.03 1.06

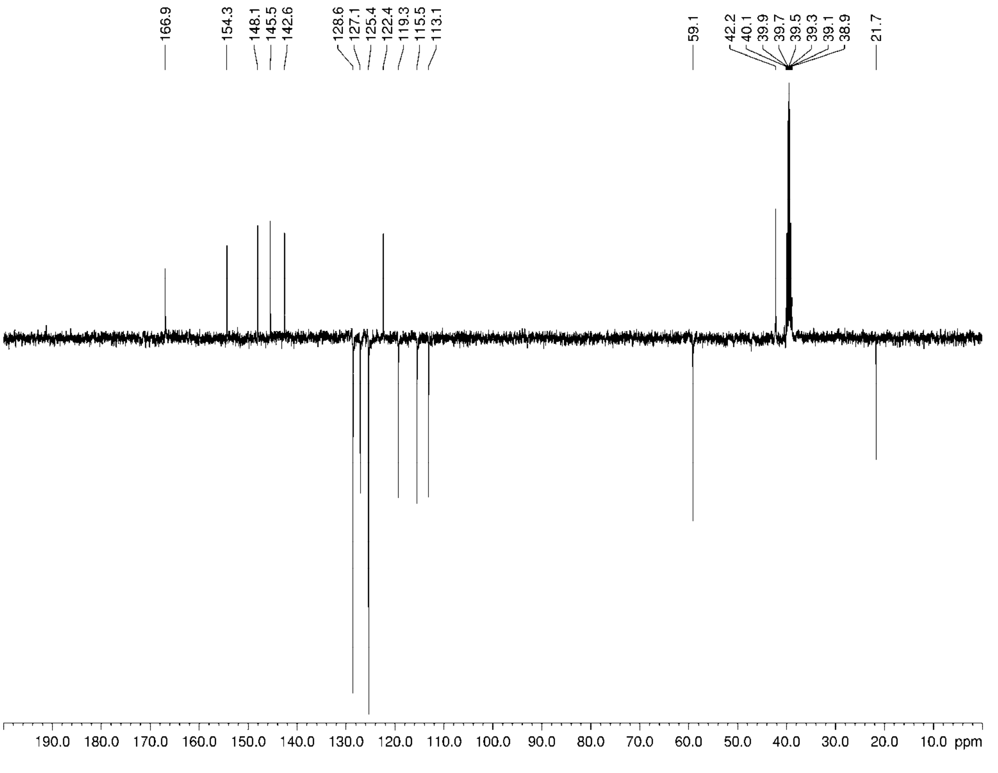
166.9
154.3 148.1 145.5 142.6
128.6 127.1 125.4 122.4 119.3 115.5 113.1
59.1
42.2 40.1 39.9 39.7 39.5 39.3 39.1 38.9
21.7
190.0 180.0 170.0 160.0 150.0 140.0 130.0 120.0 110.0 100.0 90.0 80.0 70.0 60.0 50.0 40.0 30.0 20.0 10.0 ppm

sery107

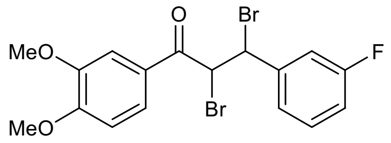

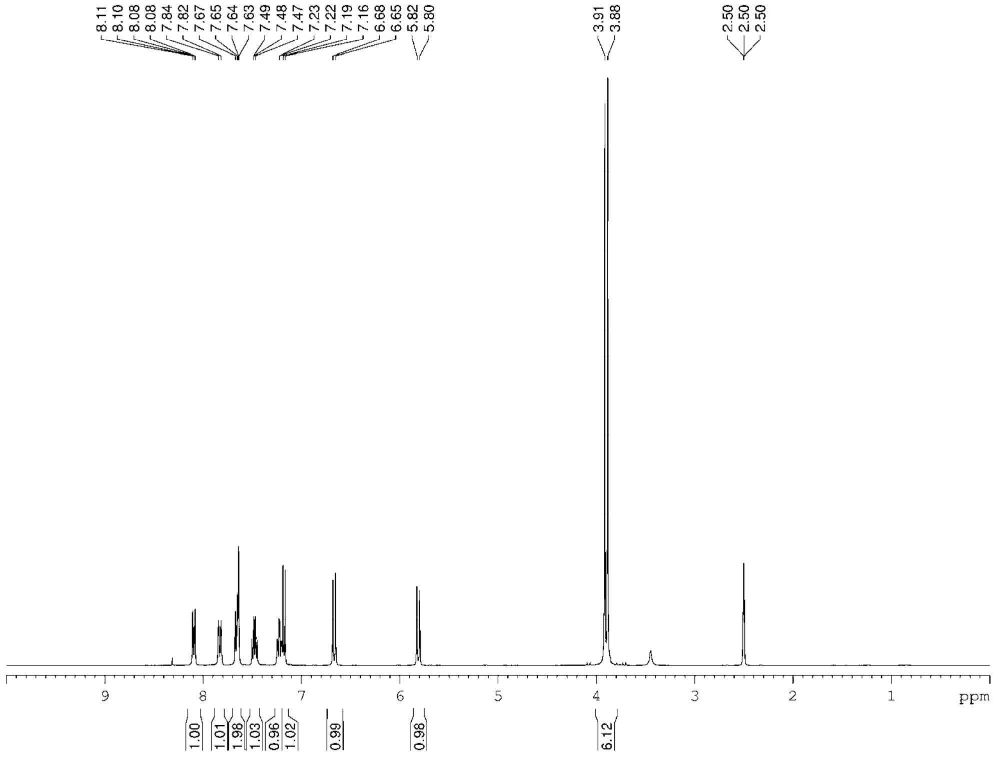

8.11
8.10
8.08
8.08
7.84
7.82
7.67
7.65
7.64
7.63
7.49
7.48
7.47
7.23
7.22
7.19
7.16
6.68
6.65
5.82
5.80
3.91
3.88
2.50
2.50
2.50
9
8
7
6
5
4
3
2
1
ppm
1.00
1.01
1.98
1.03
0.96
1.02
0.99
0.98
6.12

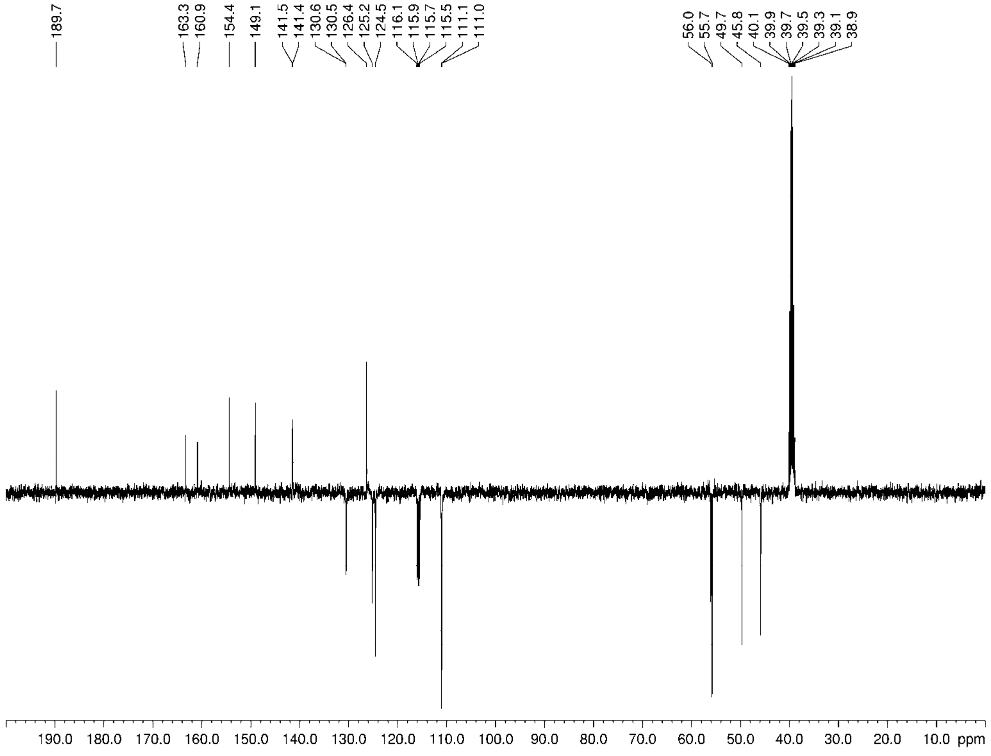

189.7
163.3
160.9
154.4
149.1
141.5
141.4
130.6
130.5
126.4
125.2
124.5
116.1
115.9
115.7
115.5
111.1
111.0
56.0
55.7
49.7
45.8
40.1
39.9
39.7
39.5
39.3
39.1
38.9
190.0
180.0
170.0
160.0
150.0
140.0
130.0
120.0
110.0
100.0
90.0
80.0
70.0
60.0
50.0
40.0
30.0
20.0
10.0 ppm

sery108

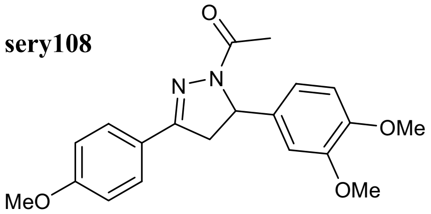
O
N
N
OMe
OMe
MeO

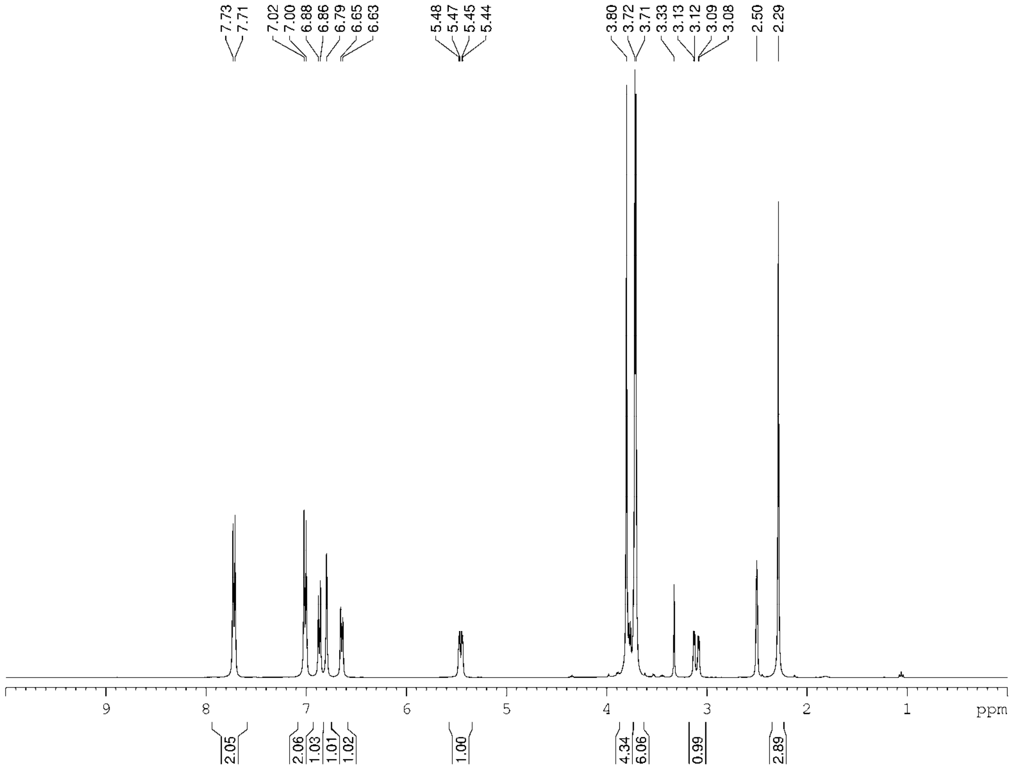
7.73
7.71
7.02
7.00
6.88
6.86
6.79
6.65
6.63
5.48
5.47
5.45
5.44
3.80
3.72
3.71
3.33
3.13
3.12
3.09
3.08
2.50
2.29
9
8
7
6
5
4
3
2
1
ppm
2.05
2.06
1.03
1.01
1.02
1.00
4.34
6.06
0.99
2.89

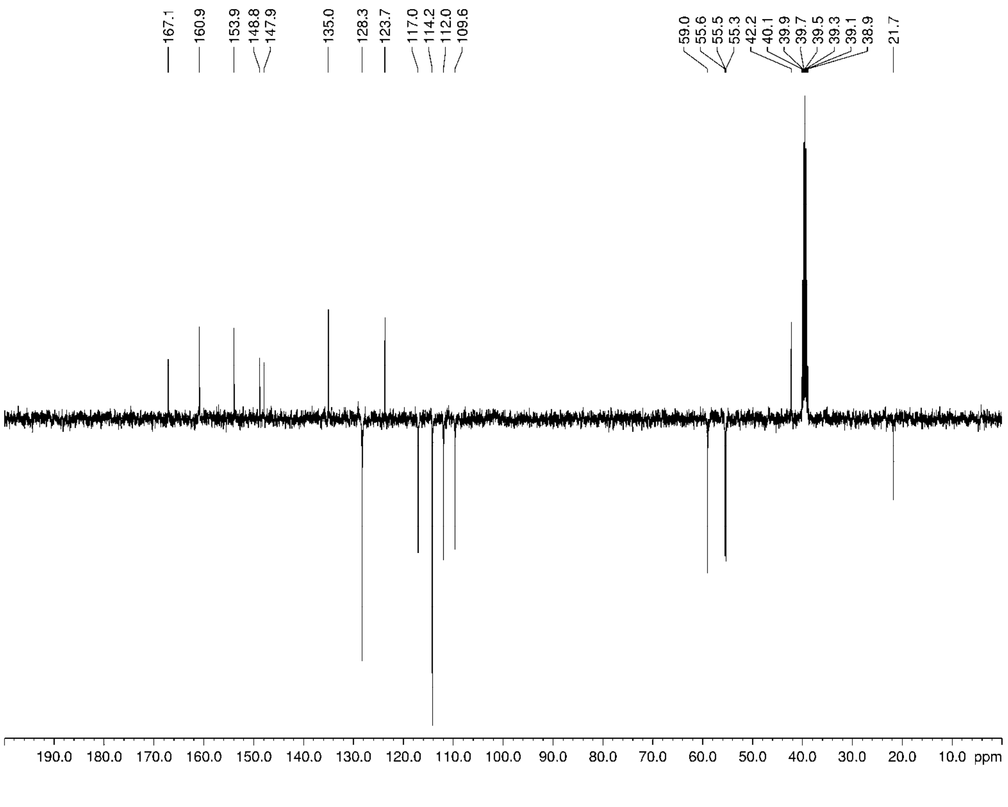
167.1
160.9
153.9
148.8
147.9
135.0
128.3
123.7
117.0
114.2
112.0
109.6
59.0
55.6
55.5
55.3
42.2
40.1
39.9
39.7
39.5
39.3
39.1
38.9
21.7
190.0
180.0
170.0
160.0
150.0
140.0
130.0
120.0
110.0
100.0
90.0
80.0
70.0
60.0
50.0
40.0
30.0
20.0
10.0
ppm

sery109

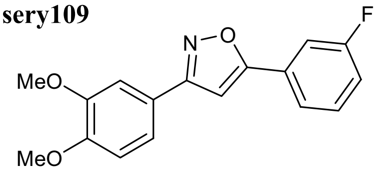
N–O
F
MeO
MeO

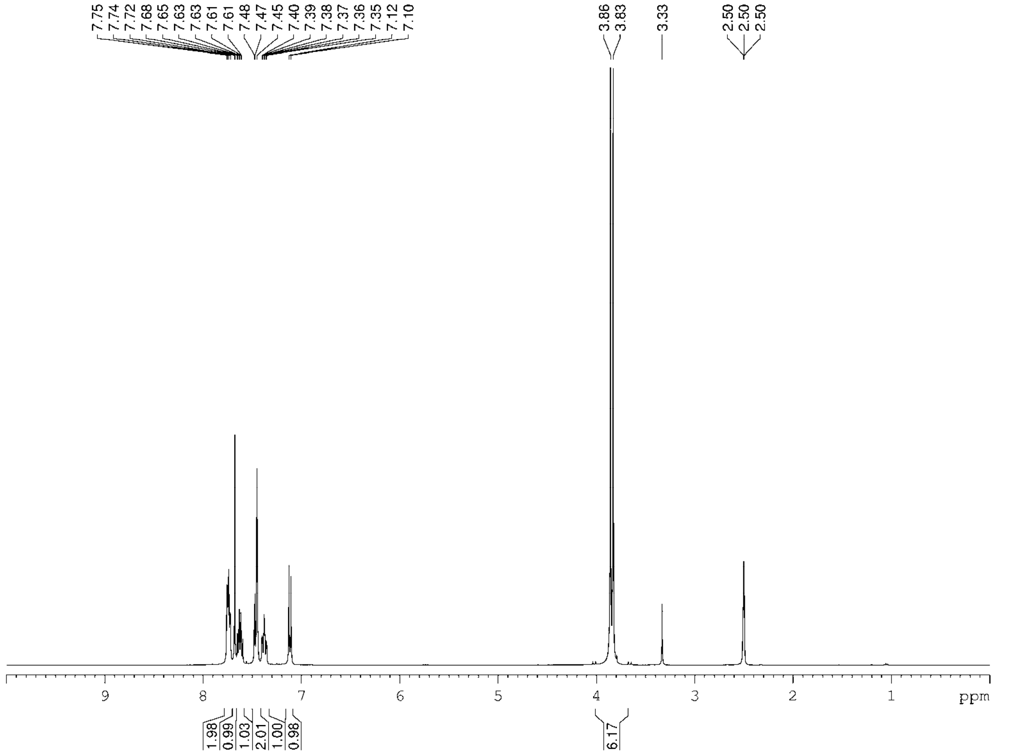
7.75 7.74 7.72 7.68 7.65 7.63 7.63 7.61 7.61 7.48 7.47 7.45 7.40 7.39 7.38 7.37 7.36 7.35 7.12 7.10
3.86 3.83
3.33
2.50 2.50 2.50
1.98 0.99 1.03 2.01 1.00 0.98
6.17
9 8 7 6 5 4 3 2 1 ppm

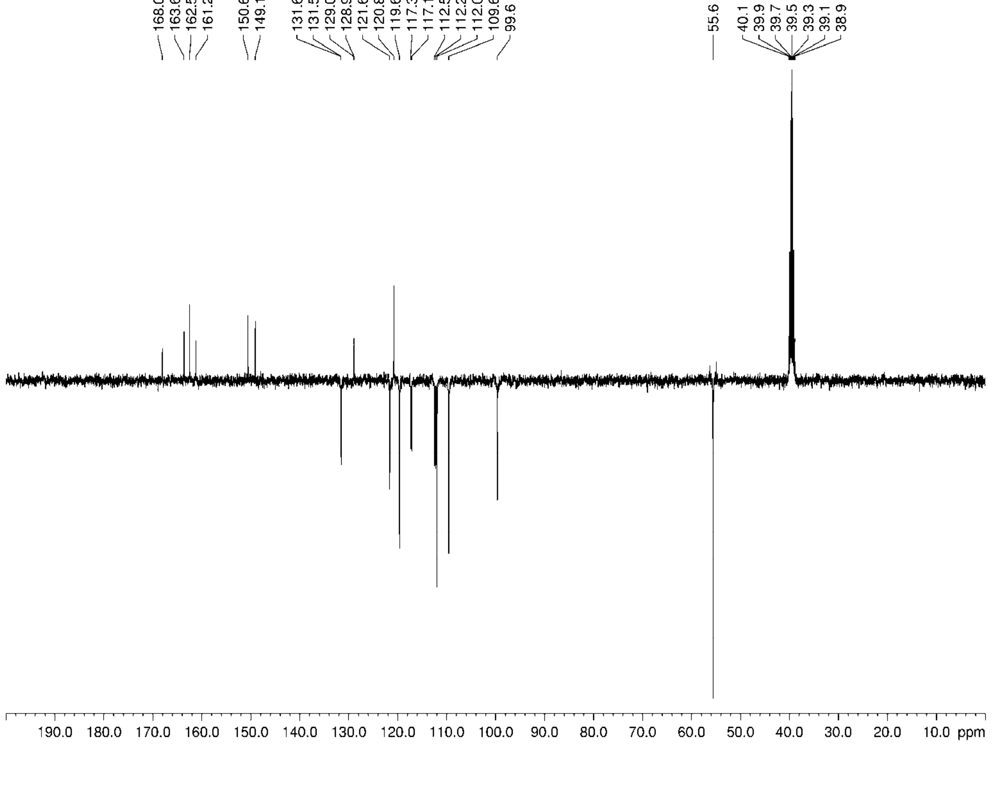
168.0 163.6 162.5 161.2
150.6 149.1
131.6 131.5 129.0 128.9 121.6 120.8 119.6 117.3 117.1 112.5 112.2 112.0 109.6 99.6
55.6
40.1 39.9 39.7 39.5 39.3 39.1 38.9
190.0 180.0 170.0 160.0 150.0 140.0 130.0 120.0 110.0 100.0 90.0 80.0 70.0 60.0 50.0 40.0 30.0 20.0 10.0 ppm

sery115

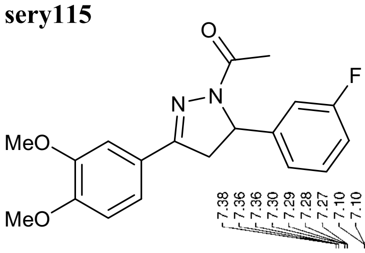
O
N
N
F
MeO
MeO

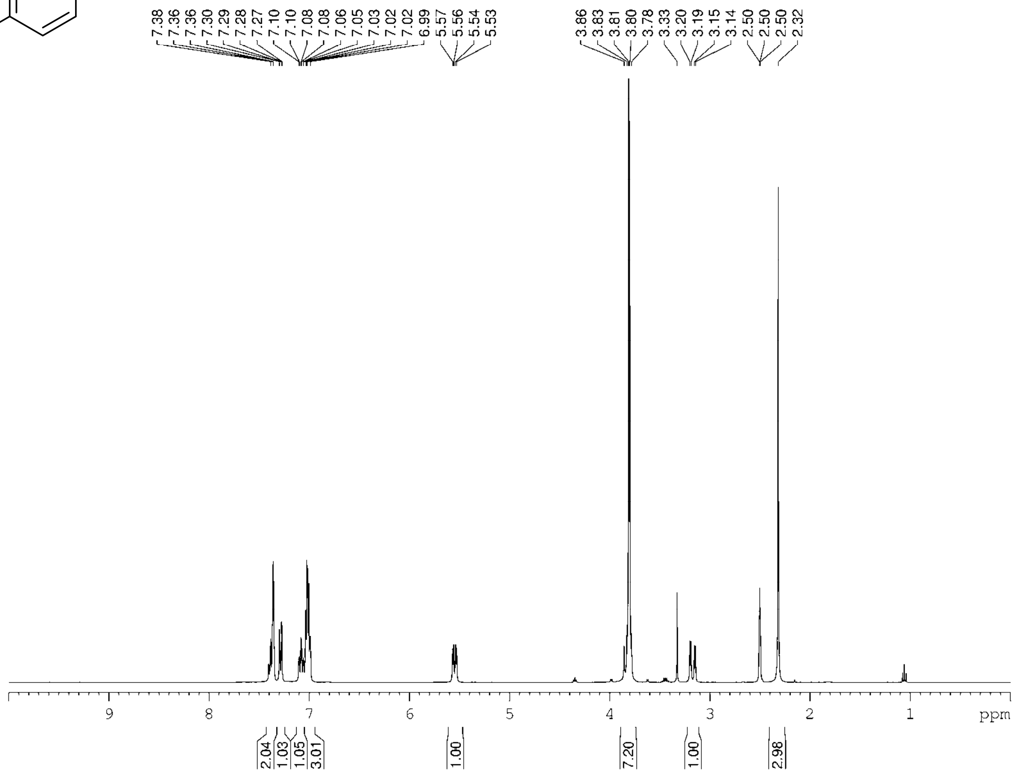
7.38
7.36
7.36
7.30
7.29
7.28
7.27
7.10
7.10
7.08
7.08
7.06
7.05
7.03
7.02
7.02
6.99
5.57
5.56
5.54
5.53
3.86
3.83
3.81
3.80
3.78
3.33
3.20
3.19
3.15
3.14
2.50
2.50
2.50
2.32
9
8
7
6
5
4
3
2
1
ppm
2.04
1.03
1.05
3.01
1.00
7.20
1.00
2.98

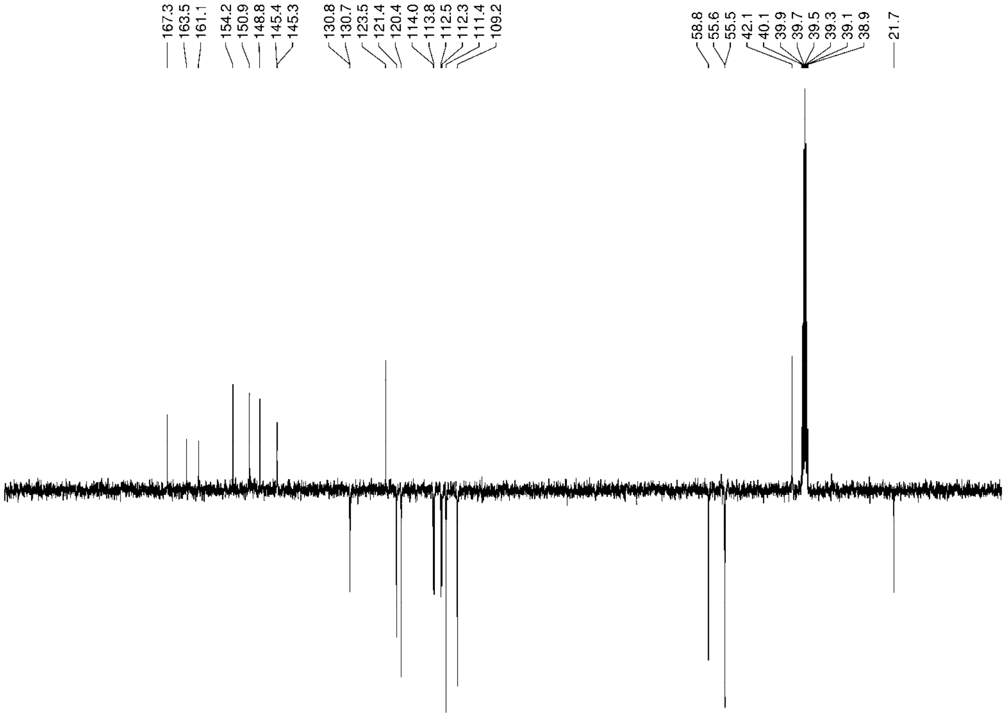
167.3
163.5
161.1
154.2
150.9
148.8
145.4
145.3
130.8
130.7
123.5
121.4
120.4
114.0
113.8
112.5
112.3
111.4
109.2
58.8
55.6
55.5
42.1
40.1
39.9
39.7
39.5
39.3
39.1
38.9
21.7
190.0
180.0
170.0
160.0
150.0
140.0
130.0
120.0
110.0
100.0
90.0
80.0
70.0
60.0
50.0
40.0
30.0
20.0
10.0 ppm

sery117

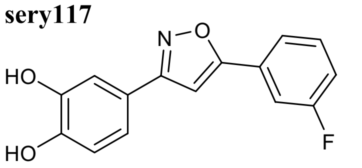
HO
HO
N
O
F

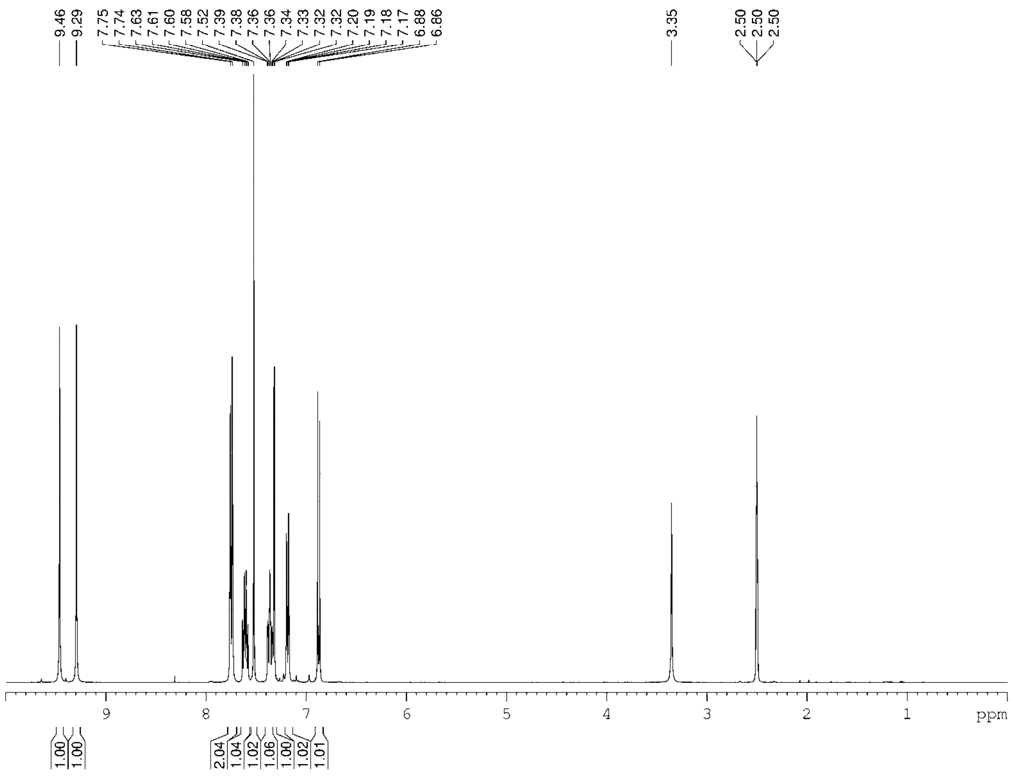
9.46
9.29
7.75
7.74
7.63
7.61
7.60
7.58
7.52
7.39
7.38
7.36
7.36
7.34
7.33
7.32
7.32
7.20
7.19
7.18
7.17
6.88
6.86
3.35
2.50
2.50
2.50
9
8
7
6
5
4
3
2
1
ppm
1.00
1.00
2.04
1.04
1.02
1.06
1.00
1.02
1.01

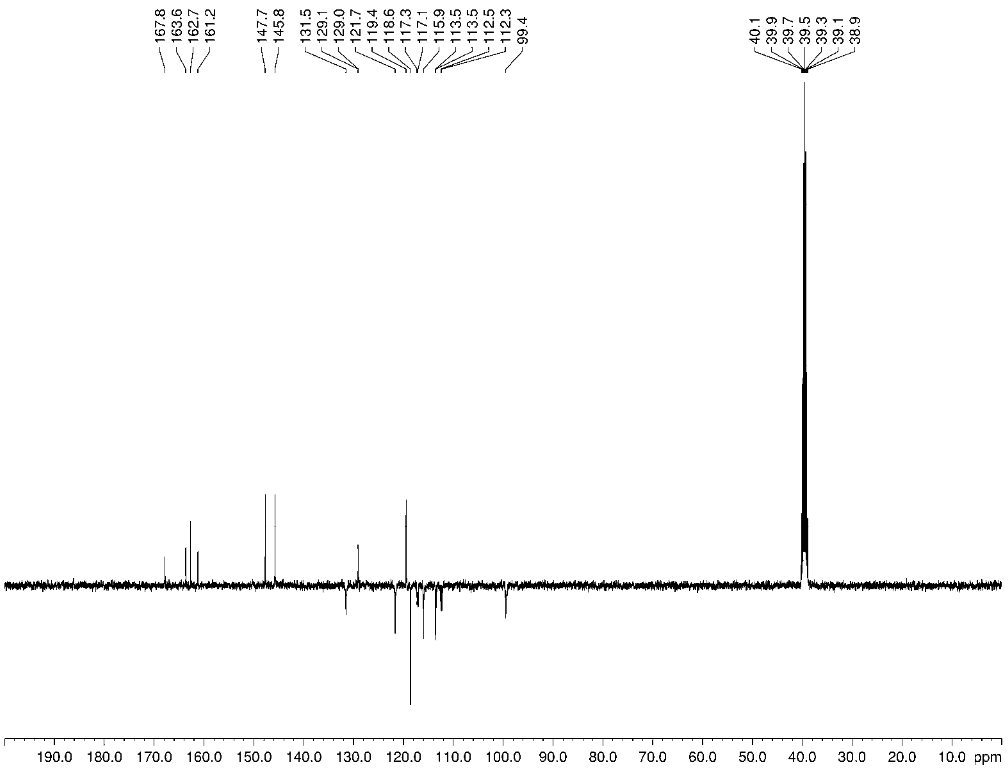
167.8
163.6
162.7
161.2
147.7
145.8
131.5
129.1
129.0
121.7
119.4
118.6
117.3
117.1
115.9
113.5
112.5
112.3
99.4
40.1
39.9
39.7
39.5
39.3
39.1
38.9
190.0
180.0
170.0
160.0
150.0
140.0
130.0
120.0
110.0
100.0
90.0
80.0
70.0
60.0
50.0
40.0
30.0
20.0
10.0 ppm

sery118

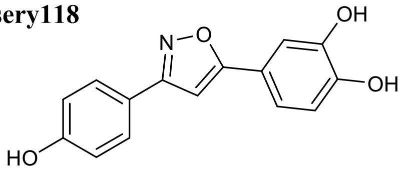
N–O
OH
OH
HO

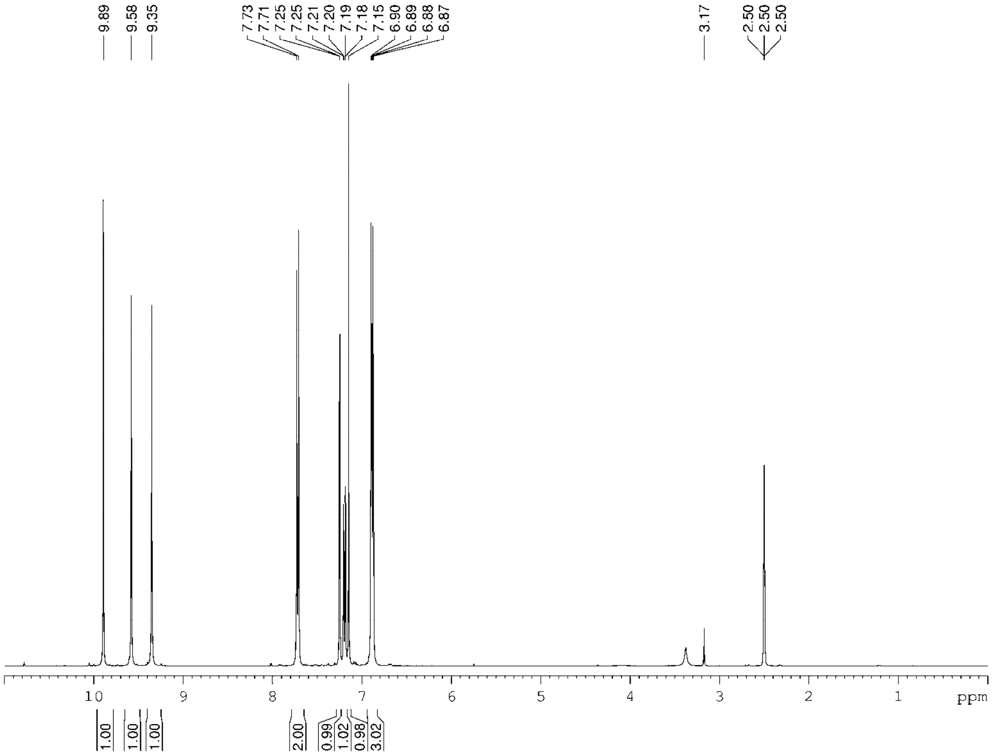
9.89
9.58
9.35
7.73
7.71
7.25
7.25
7.21
7.20
7.19
7.18
7.15
6.90
6.89
6.88
6.87
3.17
2.50
2.50
2.50
10 9 8 7 6 5 4 3 2 1 ppm
1.00
1.00
1.00
2.00
0.99
1.02
0.98
3.02

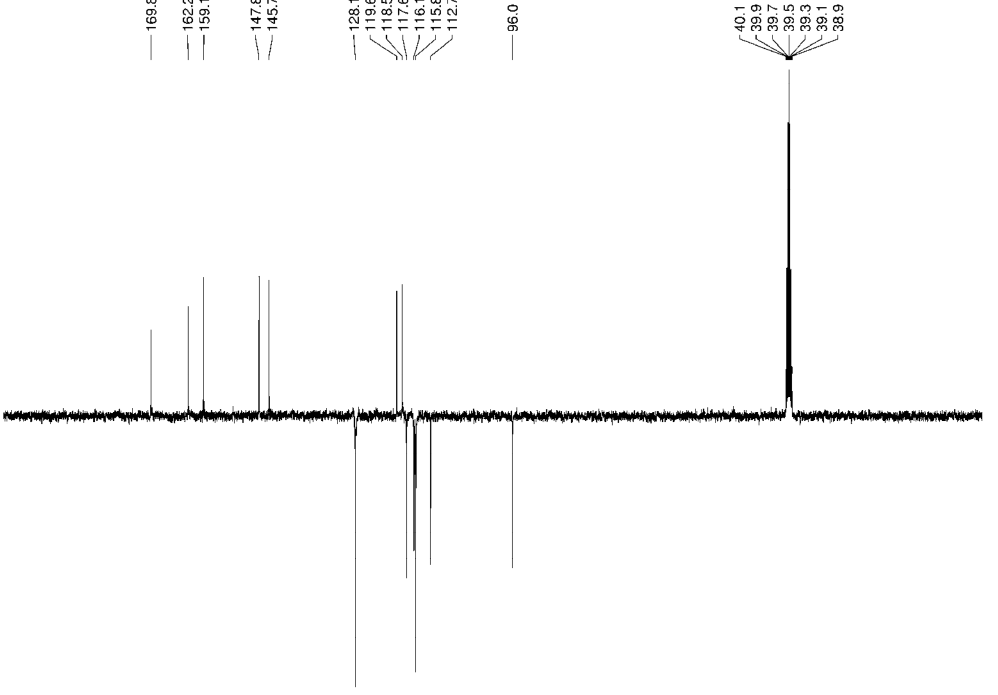
169.8
162.2
159.1
147.8
145.7
128.1
119.6
118.5
117.6
116.1
115.8
112.7
96.0
40.1
39.9
39.7
39.5
39.3
39.1
38.9
190.0 180.0 170.0 160.0 150.0 140.0 130.0 120.0 110.0 100.0 90.0 80.0 70.0 60.0 50.0 40.0 30.0 20.0 10.0 ppm

sery128

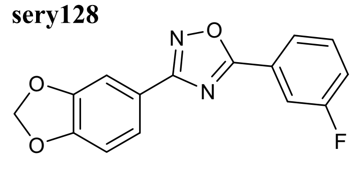

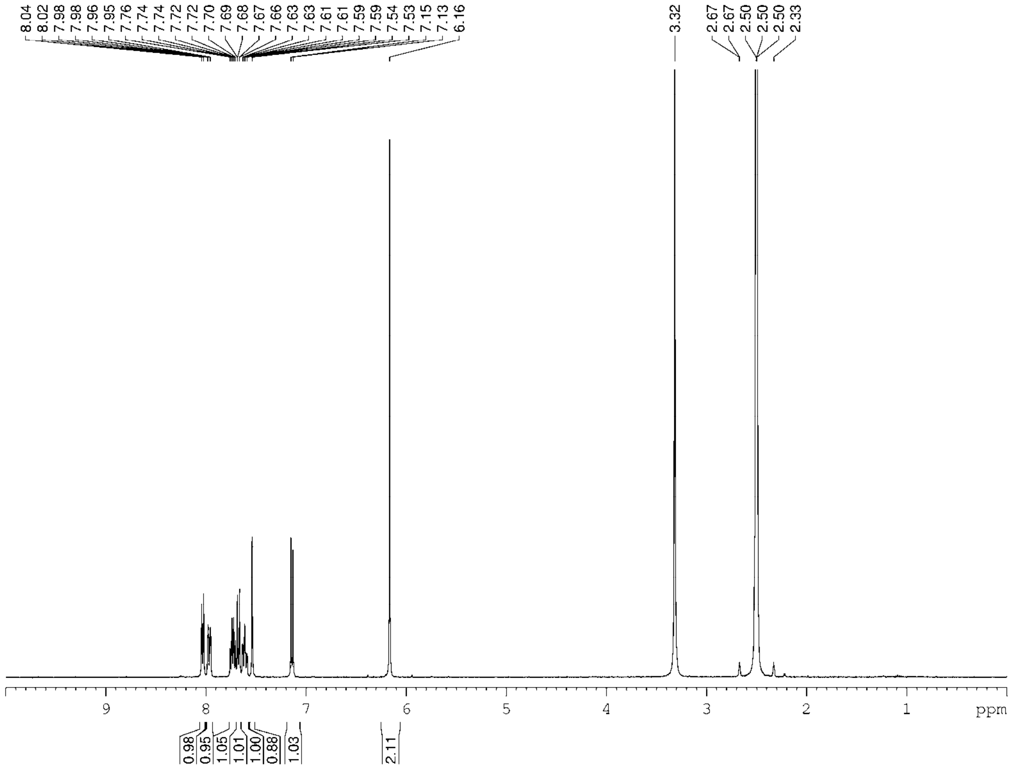
8.04
8.02
7.98
7.98
7.96
7.95
7.76
7.74
7.74
7.72
7.72
7.70
7.69
7.68
7.67
7.66
7.63
7.63
7.61
7.61
7.59
7.59
7.54
7.53
7.15
7.13
6.16
3.32
2.67
2.67
2.50
2.50
2.50
2.33
9
8
7
6
5
4
3
2
1
ppm
0.98
0.95
1.05
1.01
1.00
0.88
1.03
2.11

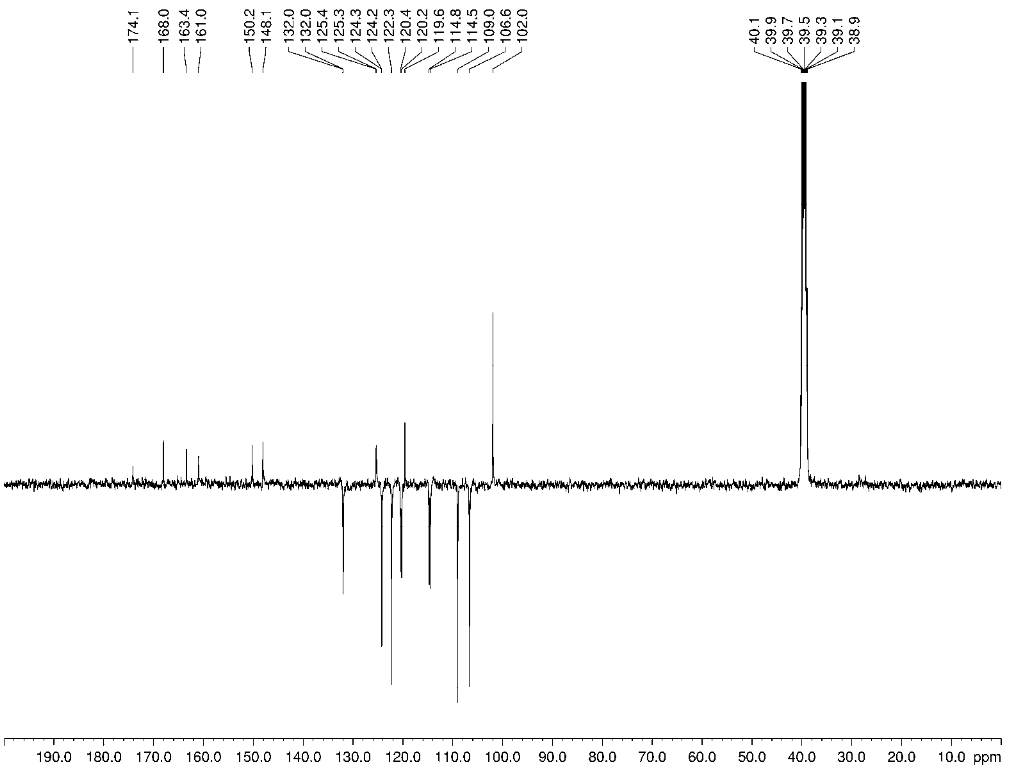
174.1
168.0
163.4
161.0
150.2
148.1
132.0
132.0
125.4
125.3
124.3
124.2
122.3
120.4
120.2
119.6
114.8
114.5
109.0
106.6
102.0
40.1
39.9
39.7
39.5
39.3
39.1
38.9
190.0
180.0
170.0
160.0
150.0
140.0
130.0
120.0
110.0
100.0
90.0
80.0
70.0
60.0
50.0
40.0
30.0
20.0
10.0
ppm

sery132

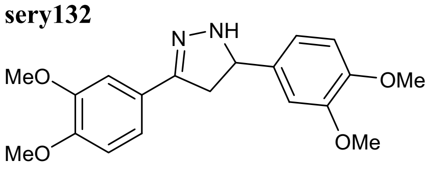
N
NH
MeO
MeO
OMe
OMe

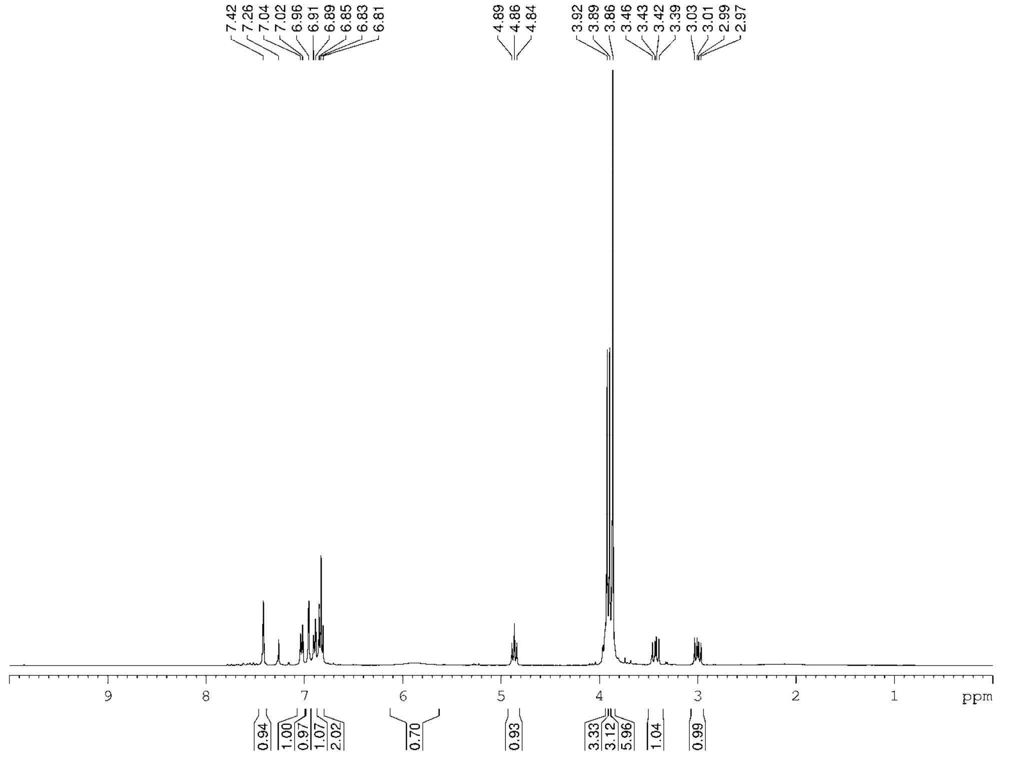
7.42
7.26
7.04
7.02
6.96
6.91
6.89
6.85
6.83
6.81
4.89
4.86
4.84
3.92
3.89
3.86
3.46
3.43
3.42
3.39
3.03
3.01
2.99
2.97
0.94
1.00
0.97
1.07
2.02
0.70
0.93
3.33
3.12
5.96
1.04
0.99
9
8
7
6
5
4
3
2
1
ppm

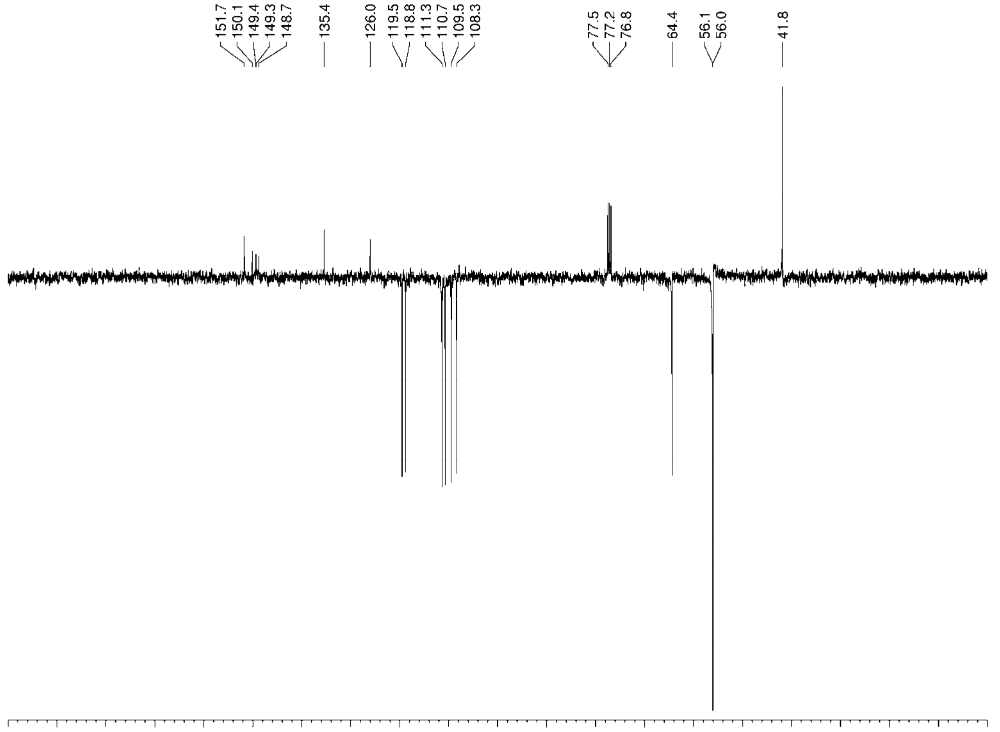
151.7
150.1
149.4
149.3
148.7
135.4
126.0
119.5
118.8
111.3
110.7
109.5
108.3
77.5
77.2
76.8
64.4
56.1
56.0
41.8
190.0
180.0
170.0
160.0
150.0
140.0
130.0
120.0
110.0
100.0
90.0
80.0
70.0
60.0
50.0
40.0
30.0
20.0
10.0 ppm

sery136

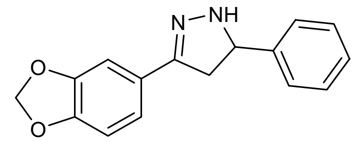
N
NH
O
O

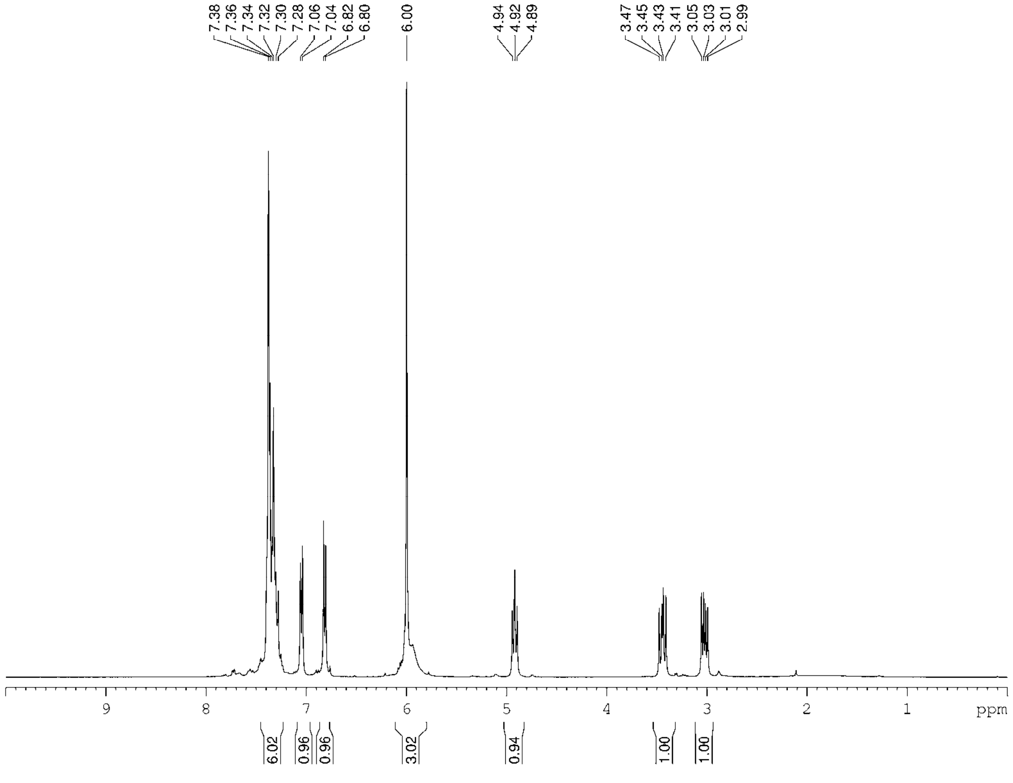
7.38
7.36
7.34
7.32
7.30
7.28
7.06
7.04
6.82
6.80
6.00
4.94
4.92
4.89
3.47
3.45
3.43
3.41
3.05
3.03
3.01
2.99
9
8
7
6
5
4
3
2
1
ppm
6.02
0.96
0.96
3.02
0.94
1.00
1.00

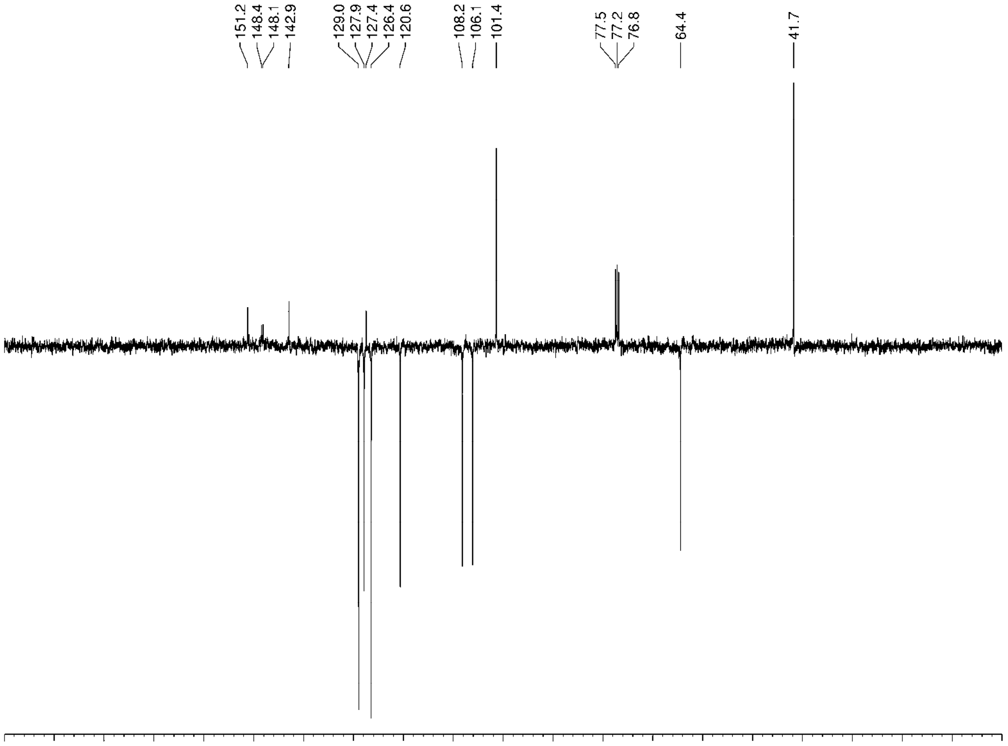
151.2
148.4
148.1
142.9
129.0
127.9
127.4
126.4
120.6
108.2
106.1
101.4
77.5
77.2
76.8
64.4
41.7
190.0
180.0
170.0
160.0
150.0
140.0
130.0
120.0
110.0
100.0
90.0
80.0
70.0
60.0
50.0
40.0
30.0
20.0
10.0 ppm

sery139

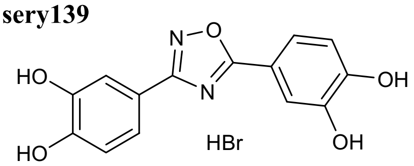
HO
HO
N
O
N
HBr
OH
OH

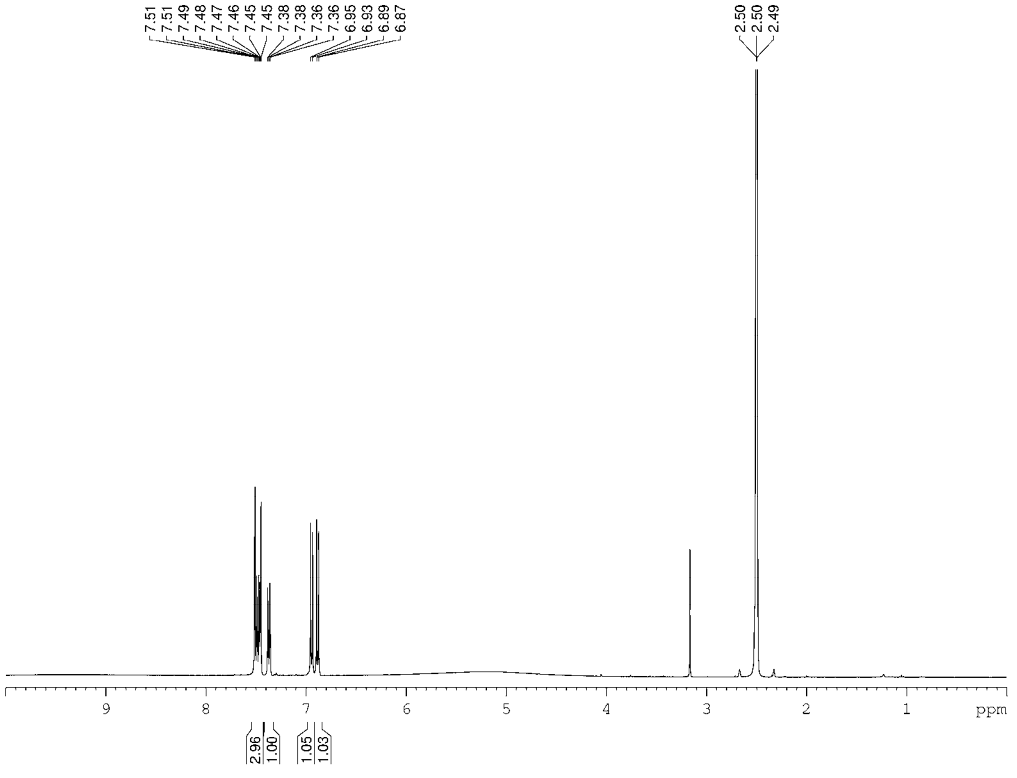
7.51
7.51
7.49
7.48
7.47
7.46
7.45
7.45
7.38
7.38
7.36
7.36
6.95
6.93
6.89
6.87
2.50
2.50
2.49
9
8
7
6
5
4
3
2
1
ppm
2.96
1.00
1.05
1.03

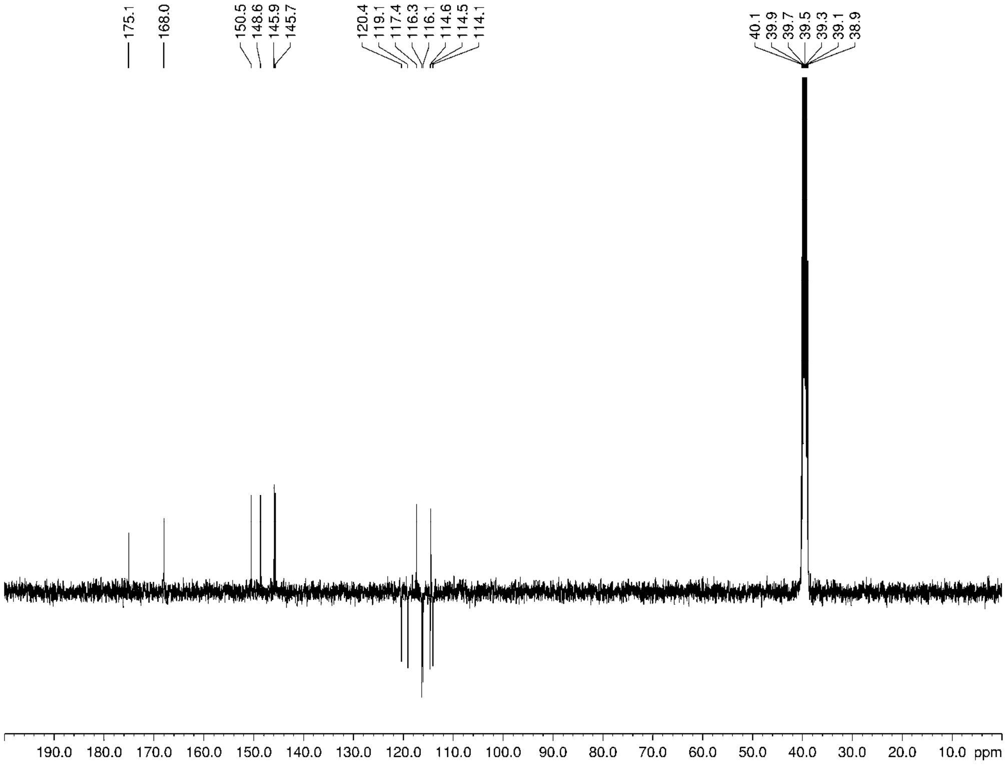
175.1
168.0
150.5
148.6
145.9
145.7
120.4
119.1
117.4
116.3
116.1
114.6
114.5
114.1
40.1
39.9
39.7
39.5
39.3
39.1
38.9
190.0
180.0
170.0
160.0
150.0
140.0
130.0
120.0
110.0
100.0
90.0
80.0
70.0
60.0
50.0
40.0
30.0
20.0
10.0 ppm

sery140

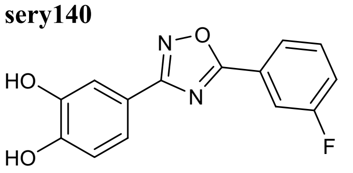

HO
HO
N
O
N
F

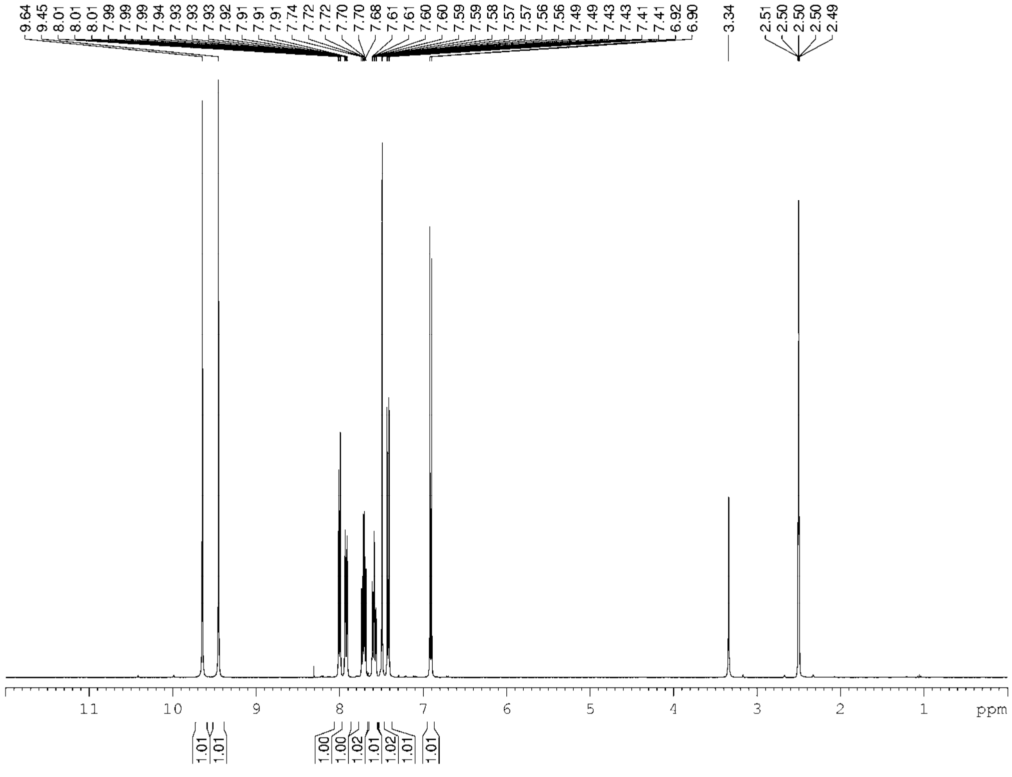

9.64 9.45 8.01 8.01 7.99 7.99 7.99 7.94 7.93 7.93 7.92 7.91 7.91 7.91 7.74 7.72 7.72 7.70 7.70 7.68 7.61 7.60 7.60 7.59 7.59 7.58 7.57 7.57 7.56 7.56 7.49 7.49 7.43 7.43 7.41 7.41 6.92 6.90
3.34
2.51 2.50 2.50 2.50 2.49
11 10 9 8 7 6 5 4 3 2 1 ppm
1.01 1.01
1.00 1.00 1.02 1.01 1.02 1.01 1.01

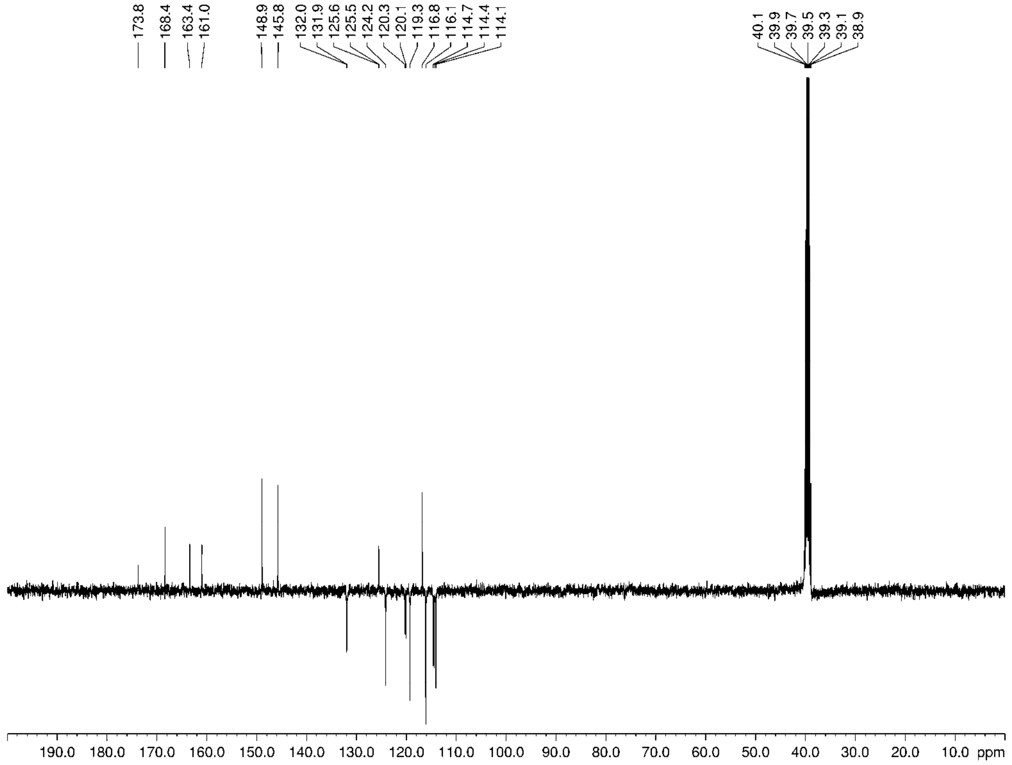

173.8 168.4 163.4 161.0
148.9 145.8
132.0 131.9 125.6 125.5 124.2 120.3 120.1 119.3 116.8 116.1 114.7 114.4 114.1
40.1 39.9 39.7 39.5 39.3 39.1 38.9
190.0 180.0 170.0 160.0 150.0 140.0 130.0 120.0 110.0 100.0 90.0 80.0 70.0 60.0 50.0 40.0 30.0 20.0 10.0 ppm

sery144

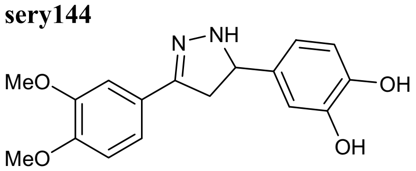
N–NH
MeO
MeO
OH
OH

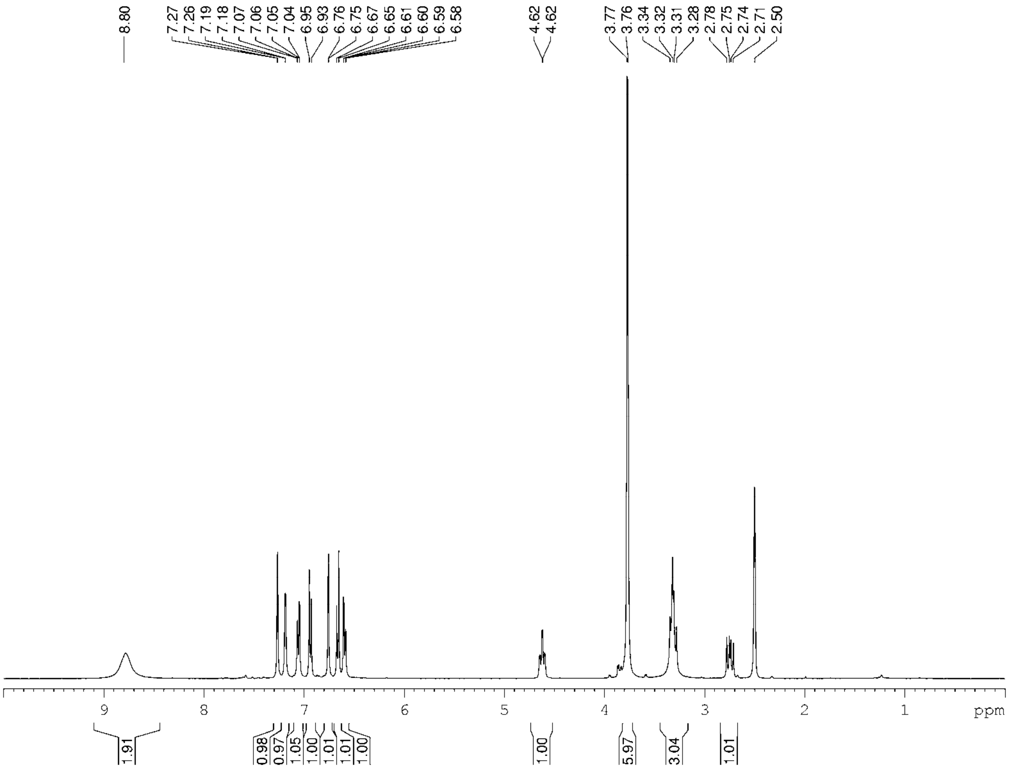
8.80
7.27 7.26 7.19 7.18 7.07 7.06 7.05 7.04 6.95 6.93 6.76 6.75 6.67 6.65 6.61 6.60 6.59 6.58
4.62 4.62
3.77 3.76 3.34 3.32 3.31 3.28 2.78 2.75 2.74 2.71 2.50
9 8 7 6 5 4 3 2 1 ppm
1.91
0.98 0.97 1.05 1.00 1.01 1.01 1.00
1.00
5.97
3.04
1.01

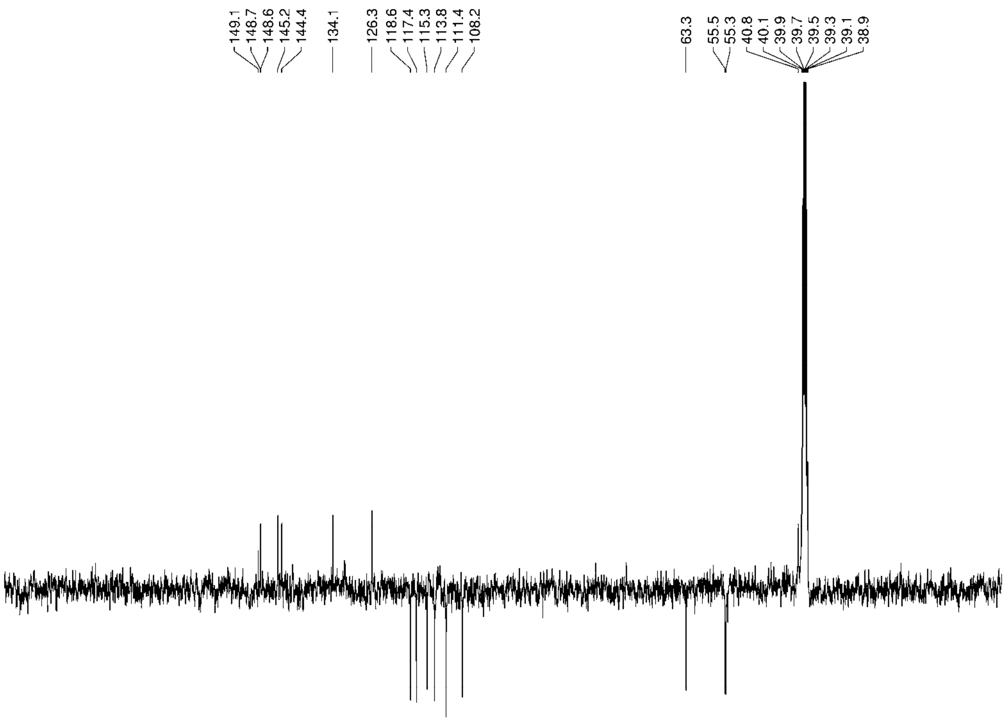
149.1 148.7 148.6 145.2 144.4
134.1
126.3 118.6 117.4 115.3 113.8 111.4 108.2
63.3
55.5 55.3 40.8 40.1 39.9 39.7 39.5 39.3 39.1 38.9
190 180 170 160 150 140 130 120 110 100 90 80 70 60 50 40 30 20 10 ppm

sery145

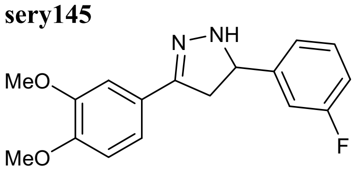
N–NH
MeO
MeO
F

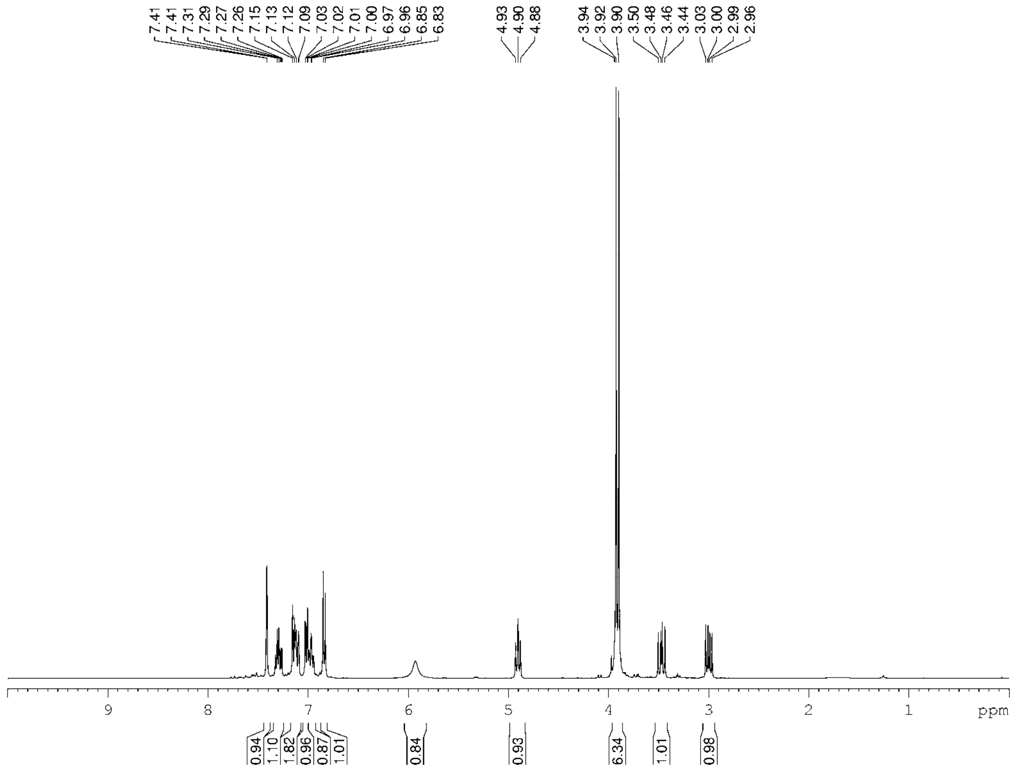
7.41 7.41 7.31 7.29 7.27 7.26 7.15 7.13 7.12 7.09 7.03 7.02 7.01 7.00 6.97 6.96 6.85 6.83
4.93 4.90 4.88
3.94 3.92 3.90 3.50 3.48 3.46 3.44 3.03 3.00 2.99 2.96
9 8 7 6 5 4 3 2 1 ppm
0.94 1.10 1.82 0.96 0.87 1.01 0.84 0.93 6.34 1.01 0.98

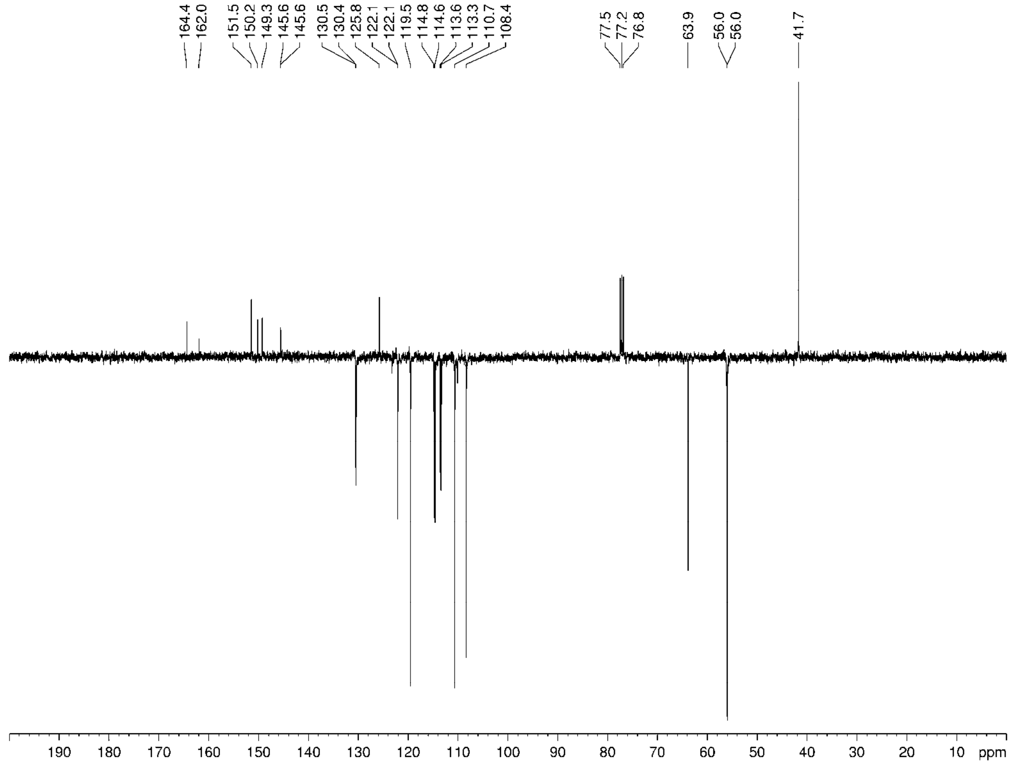
164.4 162.0 151.5 150.2 149.3 145.6 145.6 130.5 130.4 125.8 122.1 122.1 119.5 114.8 114.6 113.6 113.3 110.7 108.4
77.5 77.2 76.8 63.9 56.0 56.0 41.7
190 180 170 160 150 140 130 120 110 100 90 80 70 60 50 40 30 20 10 ppm

sery153

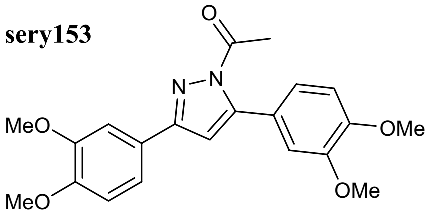
O
N
N
MeO
MeO
OMe
OMe

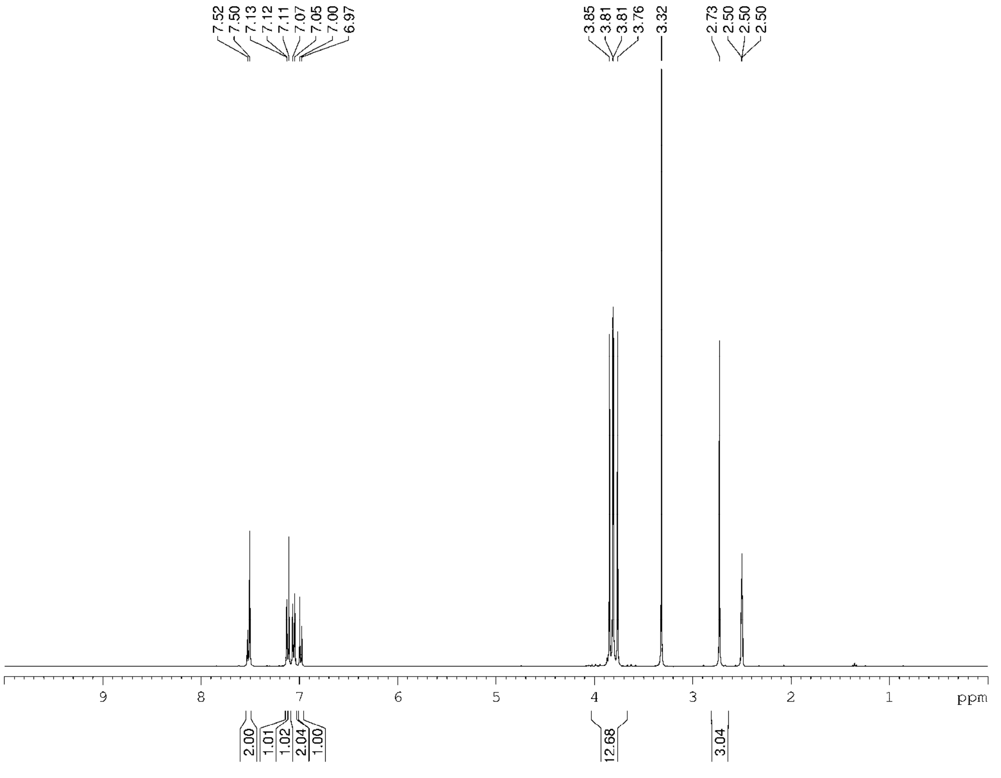
7.52
7.50
7.13
7.12
7.11
7.07
7.05
7.00
6.97
3.85
3.81
3.81
3.76
3.32
2.73
2.50
2.50
2.50
9
8
7
6
5
4
3
2
1
ppm
2.00
1.01
1.02
2.04
1.00
12.68
3.04

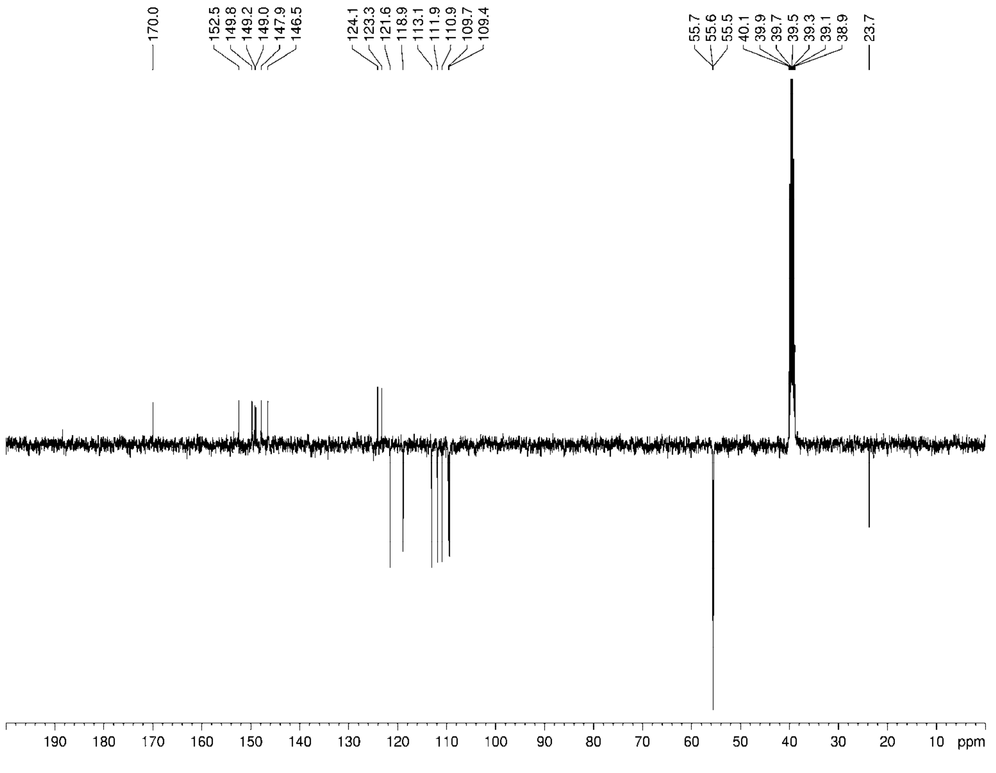
170.0
152.5
149.8
149.2
149.0
147.9
146.5
124.1
123.3
121.6
118.9
113.1
111.9
110.9
109.7
109.4
55.7
55.6
55.5
40.1
39.9
39.7
39.5
39.3
39.1
38.9
23.7
190
180
170
160
150
140
130
120
110
100
90
80
70
60
50
40
30
20
10
ppm

sery154

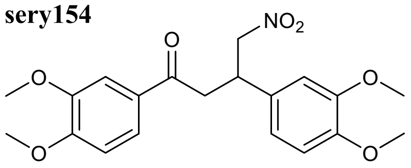

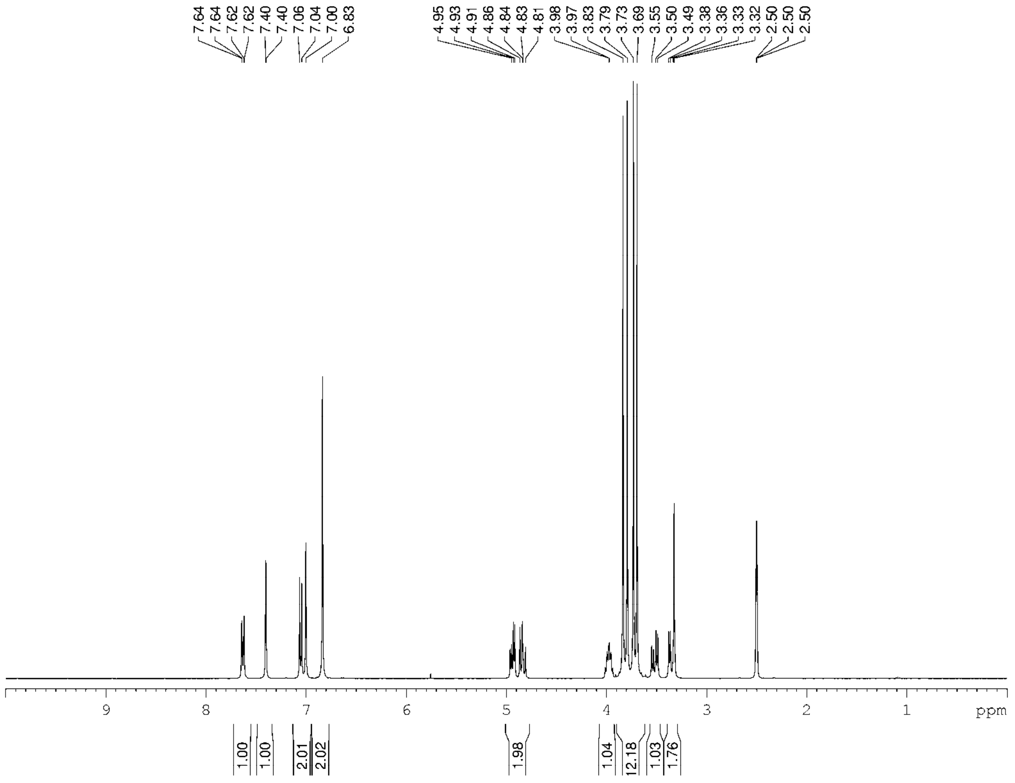

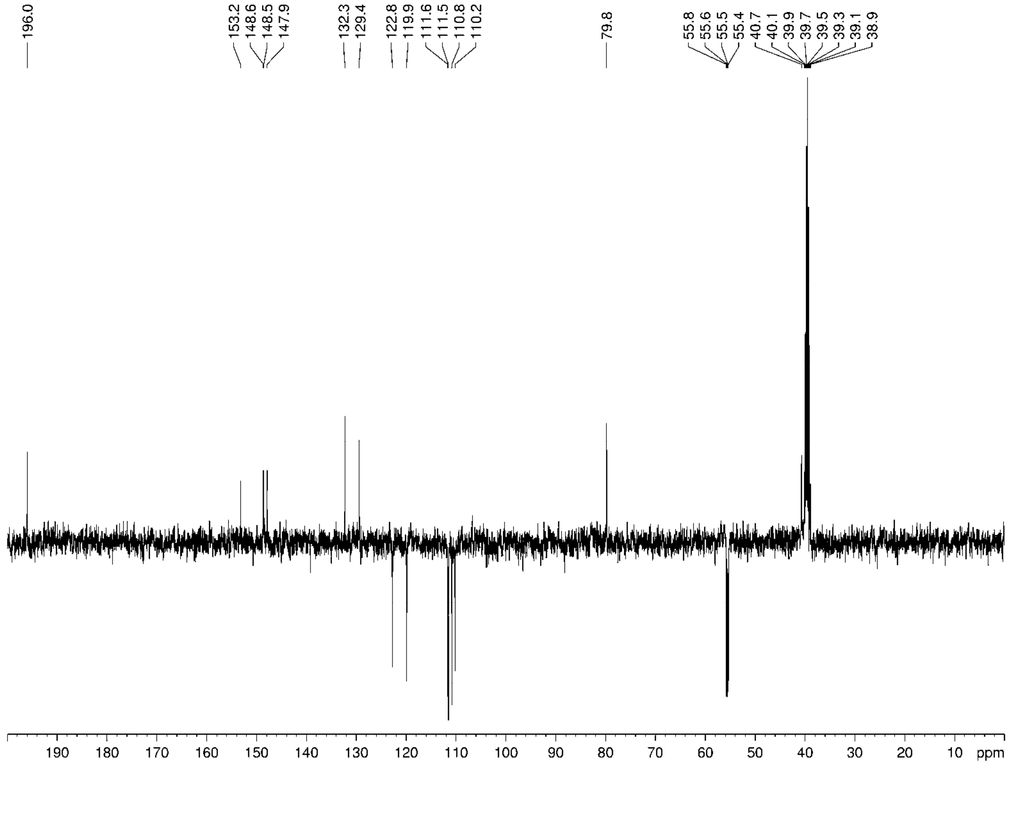

sery155

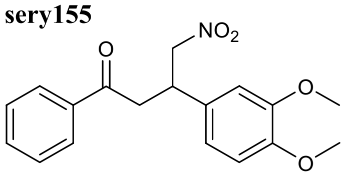

O
NO_2
O
O

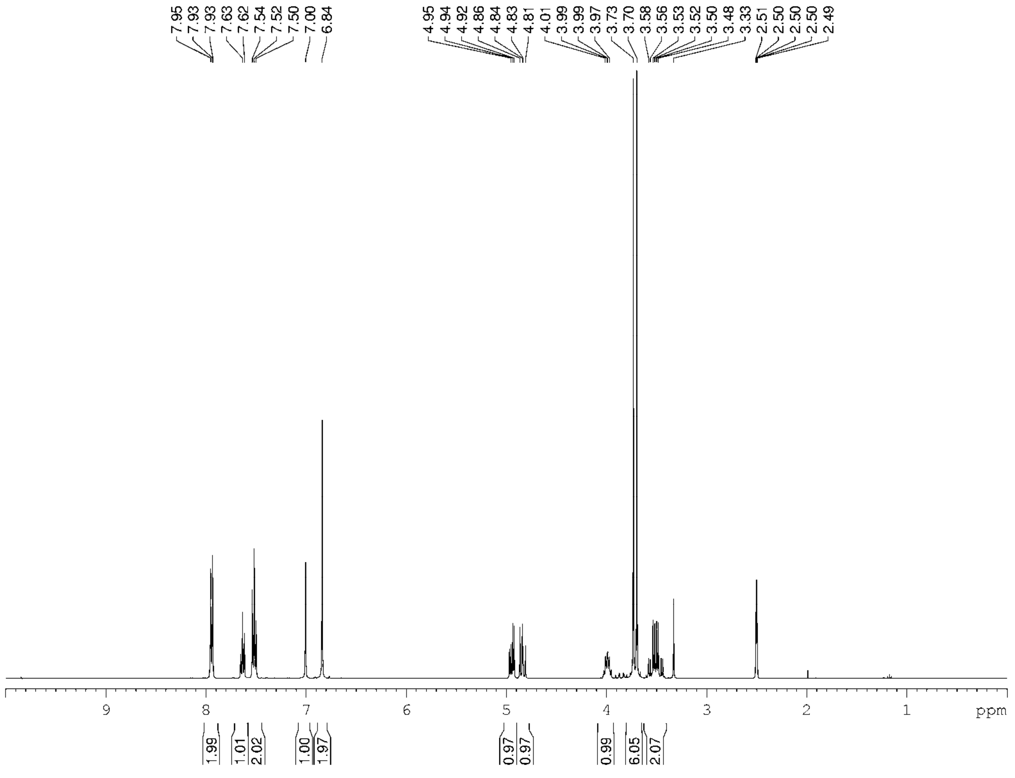

7.95 7.93 7.93 7.63 7.62 7.54 7.52 7.50 7.00 6.84
4.95 4.94 4.92 4.86 4.84 4.83 4.81 4.01 3.99 3.99 3.97 3.73 3.70 3.58 3.56 3.53 3.52 3.50 3.48 3.33 2.51 2.50 2.50 2.50 2.49
9 8 7 6 5 4 3 2 1 ppm
1.99 1.01 2.02 1.00 1.97 0.97 0.97 0.99 6.05 2.07

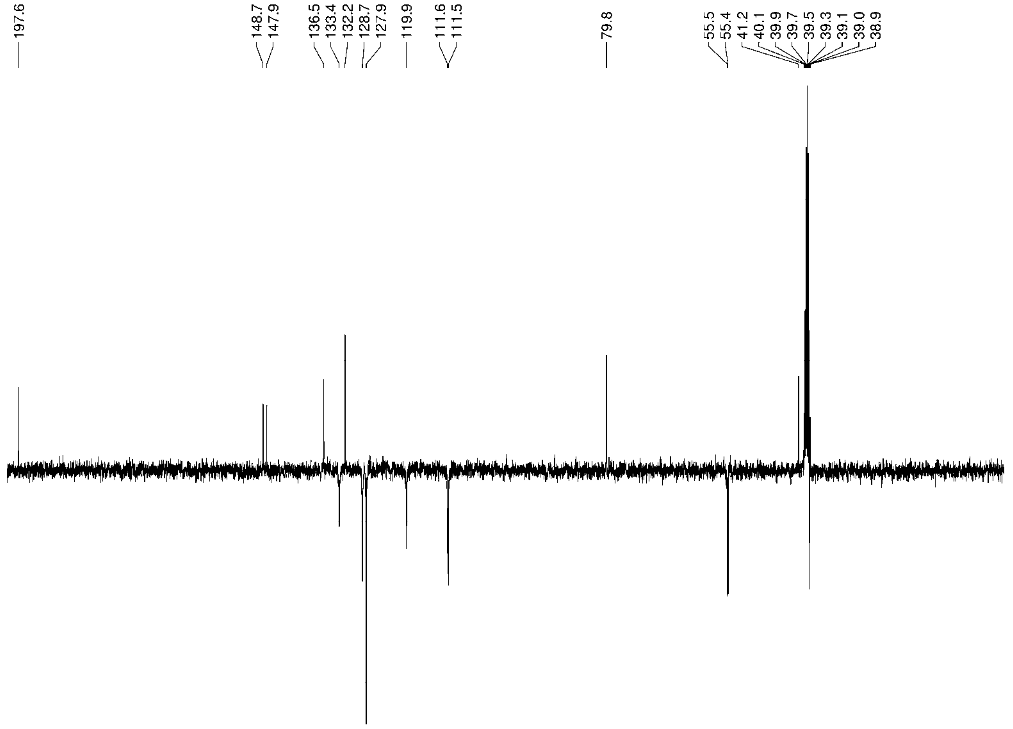

197.6
148.7 147.9
136.5 133.4 132.2 128.7 127.9
119.9
111.6 111.5
79.8
55.5 55.4 41.2 40.1 39.9 39.7 39.5 39.3 39.1 39.0 38.9
190 180 170 160 150 140 130 120 110 100 90 80 70 60 50 40 30 20 10 ppm

sery156

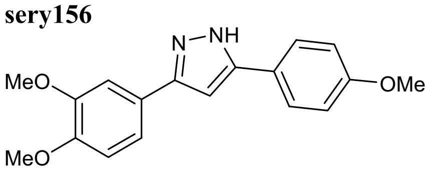

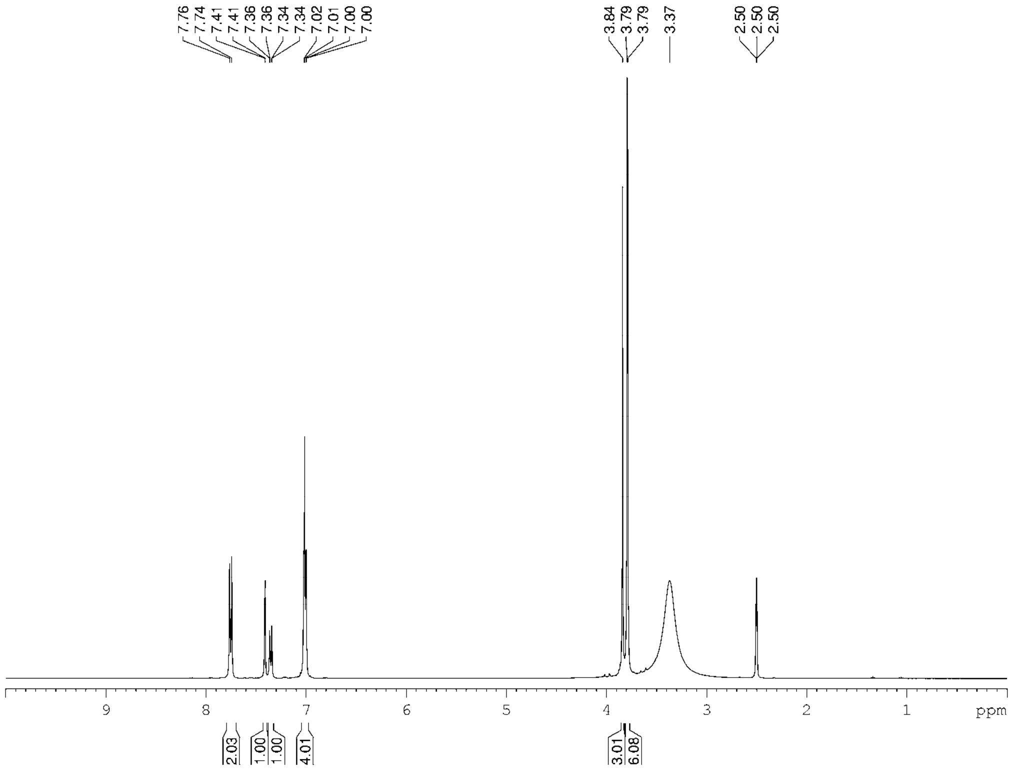

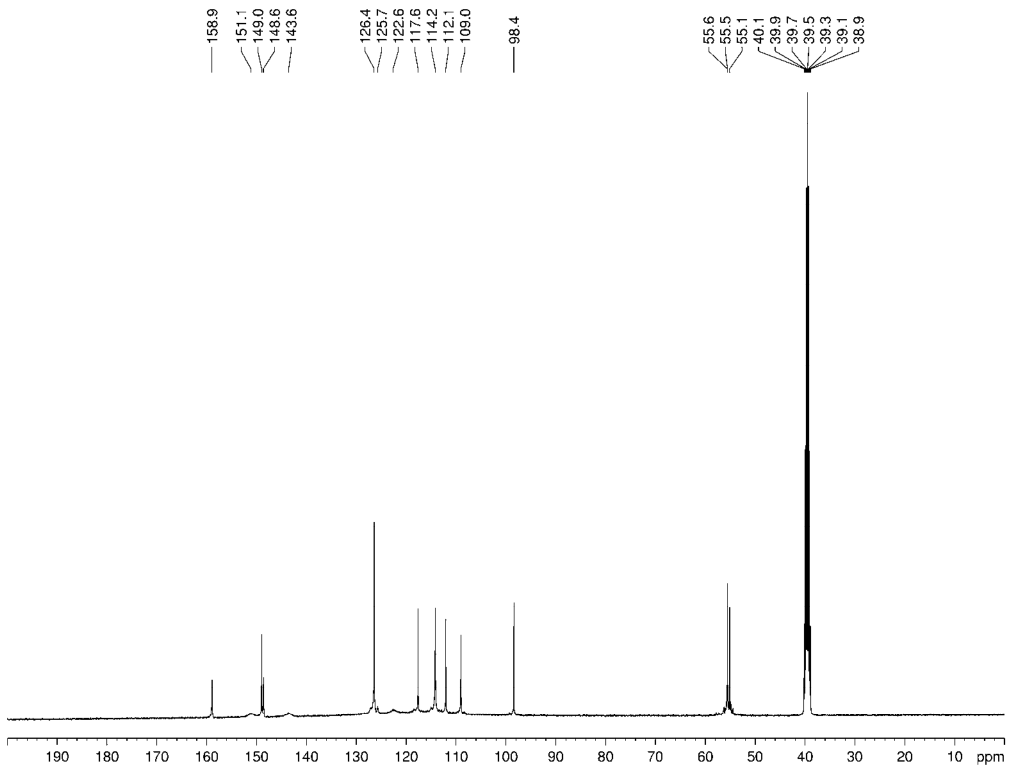

sery157

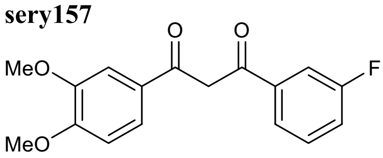

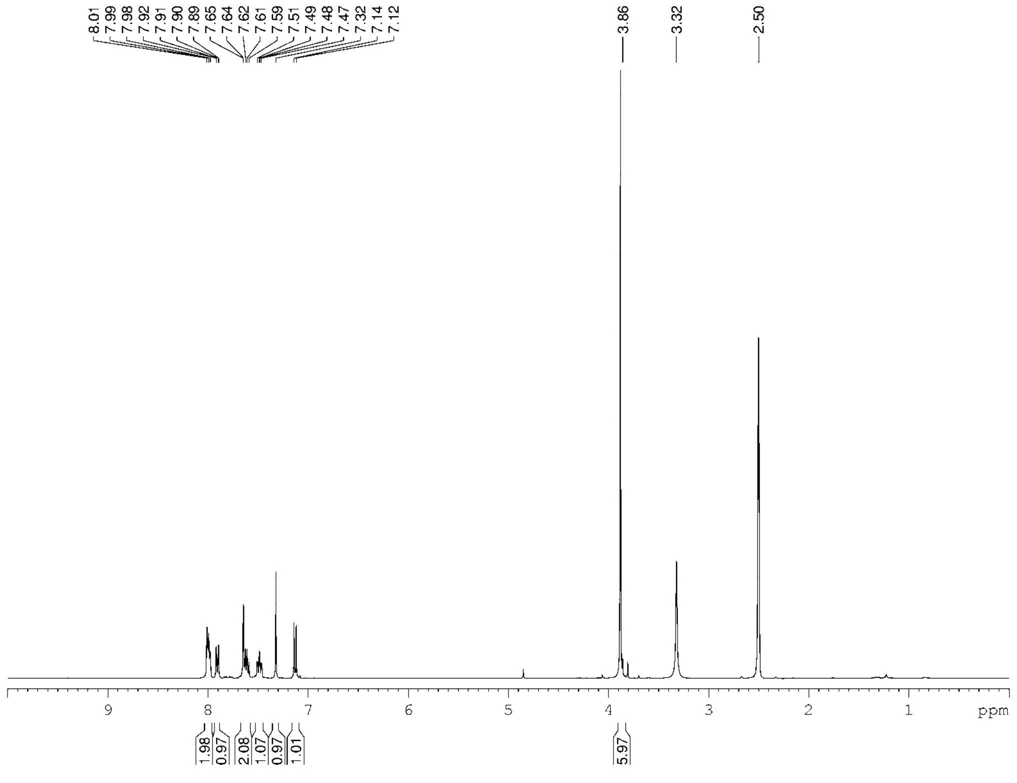

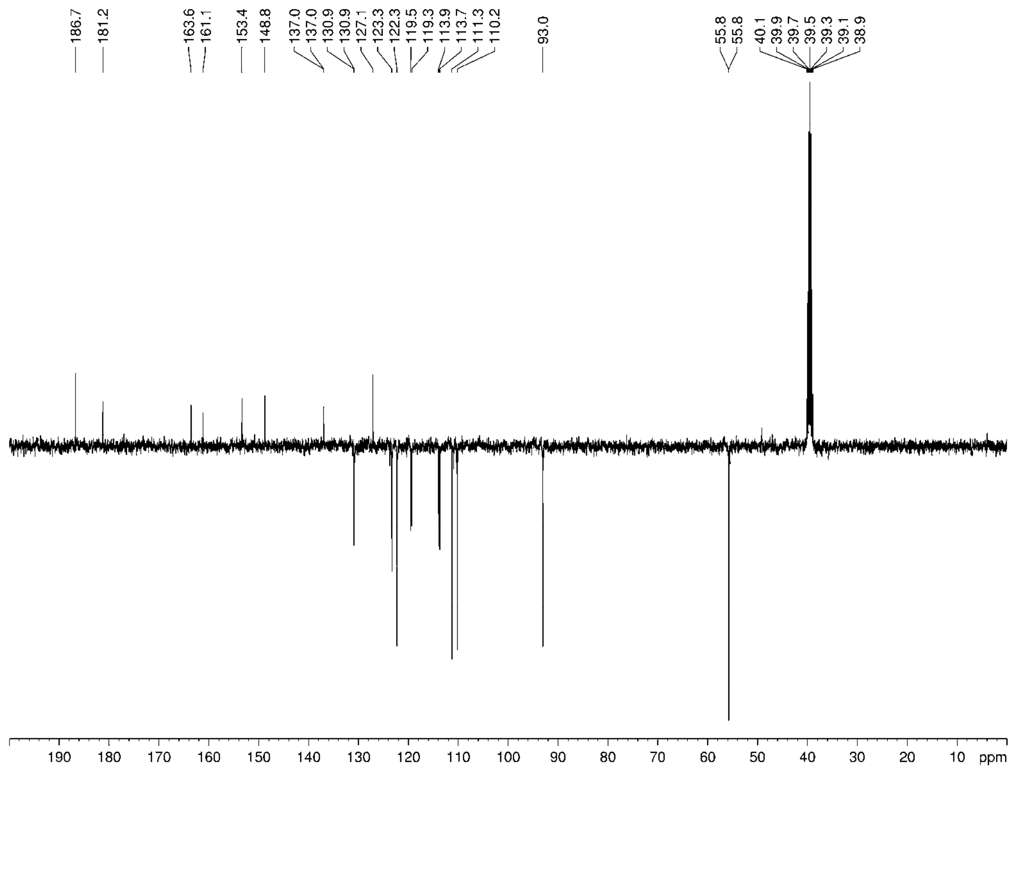

sery158B

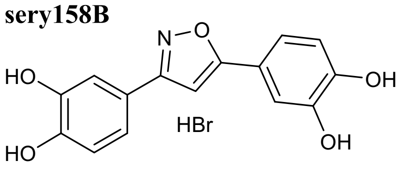
HO
HO
N
O
HBr
OH
OH

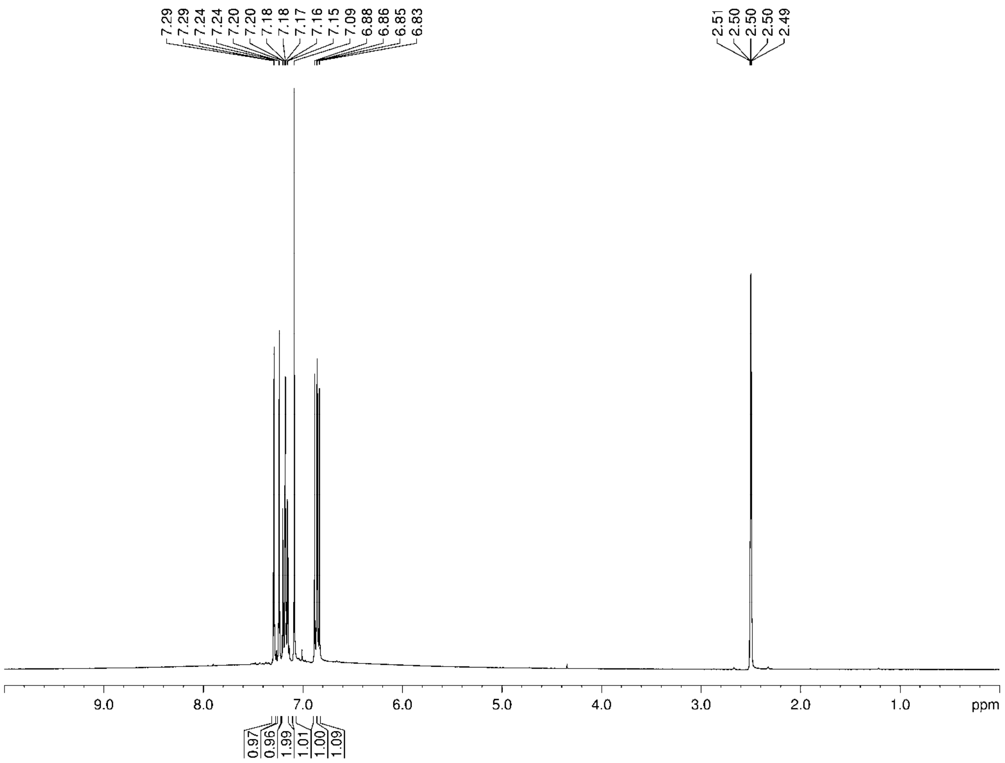
7.29
7.29
7.24
7.24
7.20
7.20
7.18
7.18
7.17
7.16
7.15
7.09
6.88
6.86
6.85
6.83
2.51
2.50
2.50
2.50
2.49
9.0
8.0
7.0
6.0
5.0
4.0
3.0
2.0
1.0
ppm
0.97
0.96
1.99
1.01
1.00
1.09

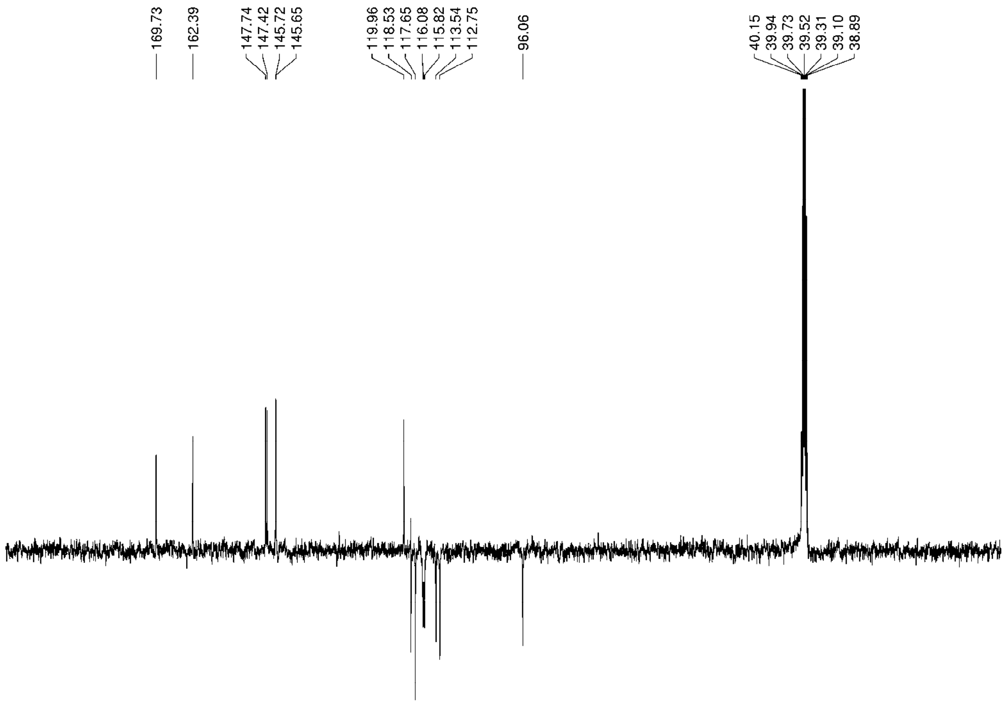
169.73
162.39
147.74
147.42
145.72
145.65
119.96
118.53
117.65
116.08
115.82
113.54
112.75
96.06
40.15
39.94
39.73
39.52
39.31
39.10
38.89
190
180
170
160
150
140
130
120
110
100
90
80
70
60
50
40
30
20
10
ppm

sery160

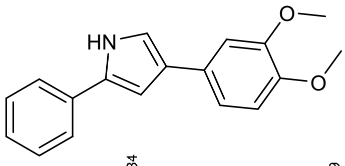

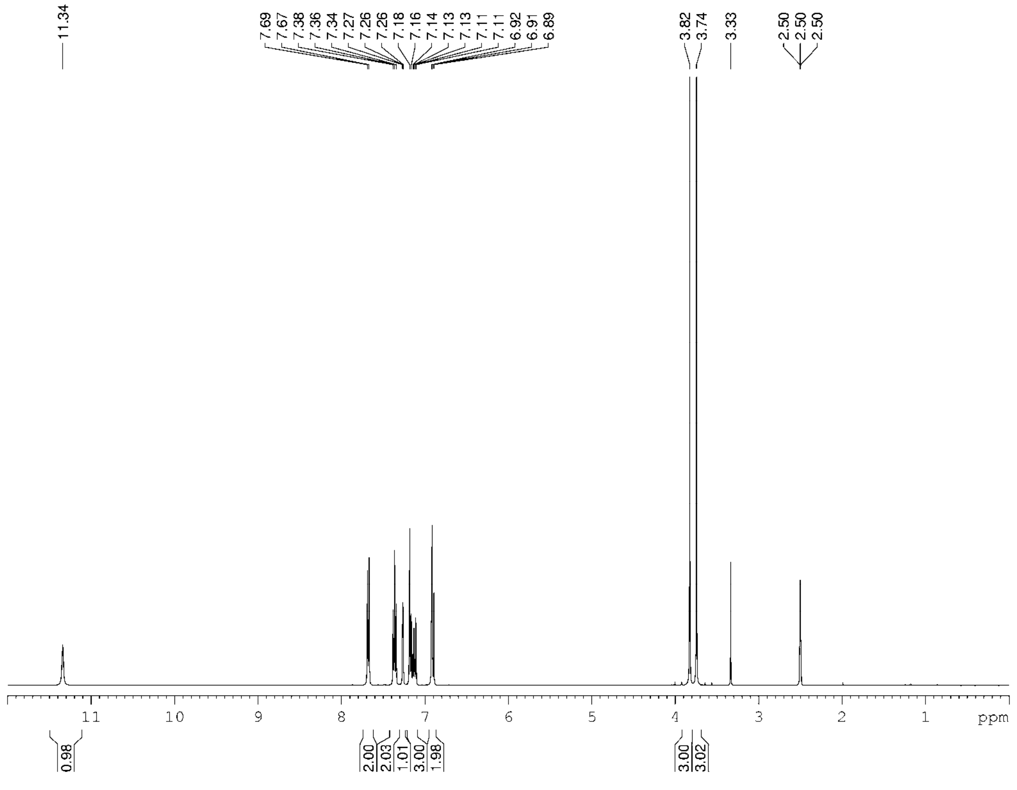

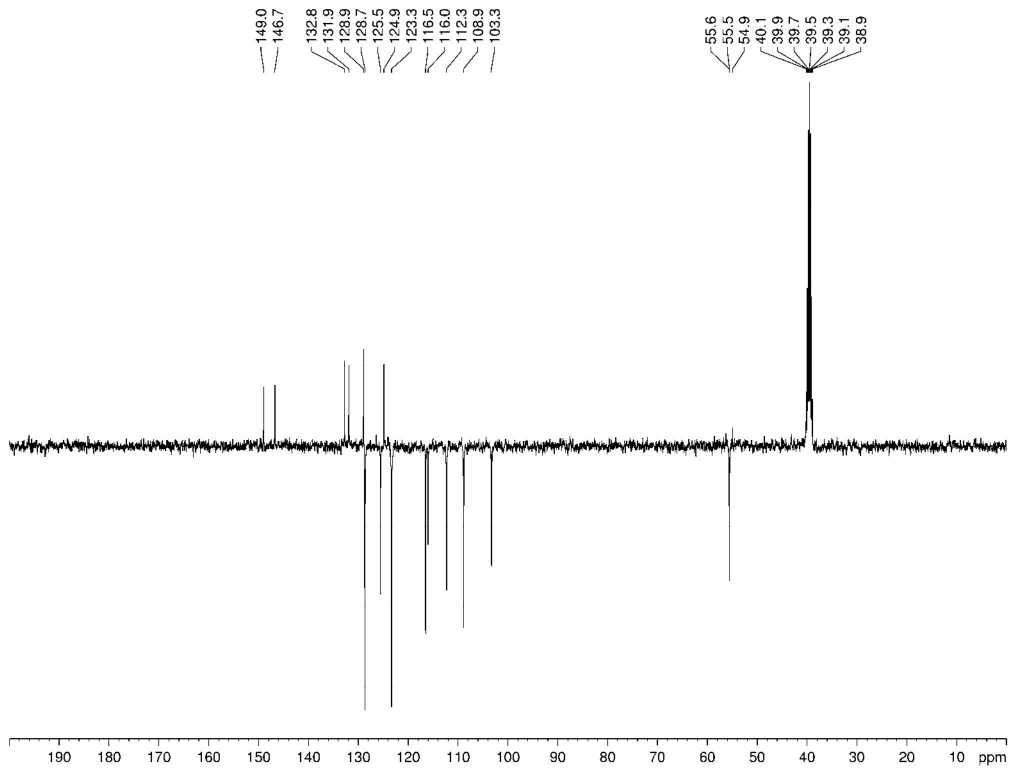

sery161

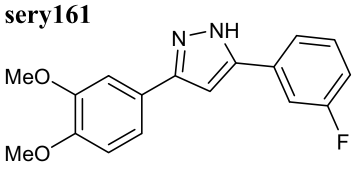

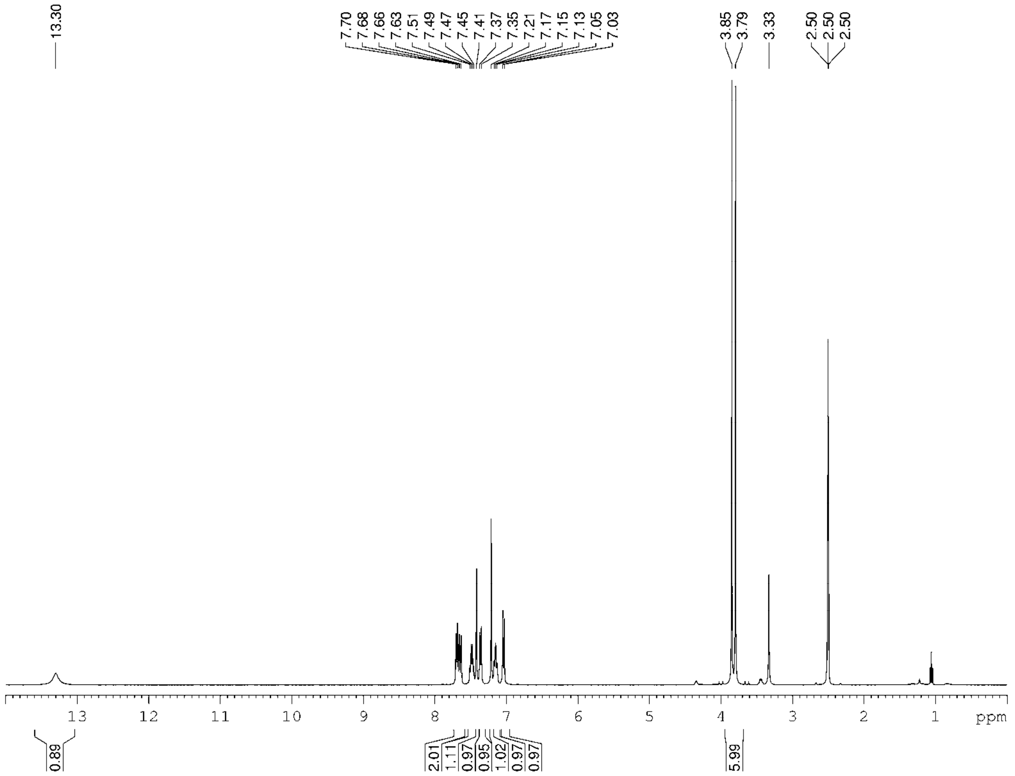

13.30
7.70
7.68
7.66
7.63
7.51
7.49
7.47
7.45
7.41
7.37
7.35
7.21
7.17
7.15
7.13
7.05
7.03
3.85
3.79
3.33
2.50
2.50
2.50
13
12
11
10
9
8
7
6
5
4
3
2
1
ppm
0.89
2.01
1.11
0.97
0.95
1.02
0.97
0.97
5.99

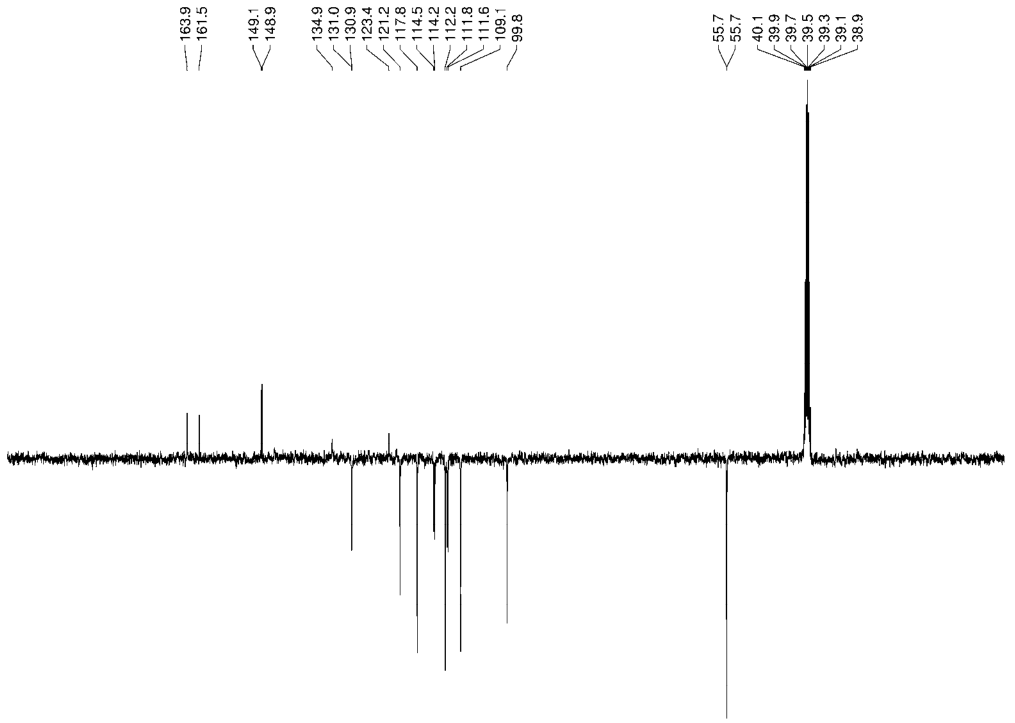

163.9
161.5
149.1
148.9
134.9
131.0
130.9
123.4
121.2
117.8
114.5
114.2
112.2
111.8
111.6
109.1
99.8
55.7
55.7
40.1
39.9
39.7
39.5
39.3
39.1
38.9
190
180
170
160
150
140
130
120
110
100
90
80
70
60
50
40
30
20
10
ppm

sery165

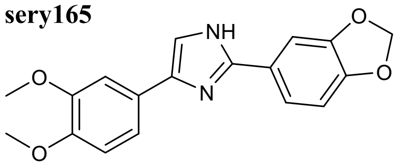

NH
N
O
O
O
O

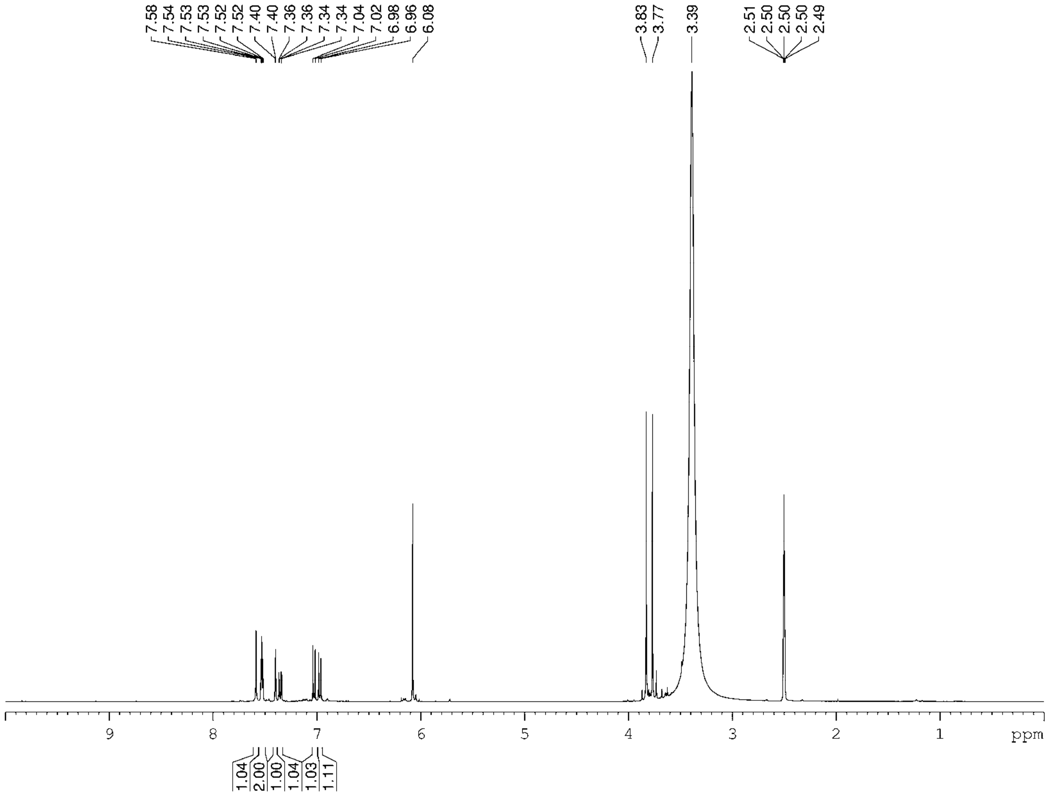

7.58
7.54
7.53
7.53
7.52
7.52
7.40
7.40
7.36
7.36
7.34
7.34
7.04
7.02
6.98
6.96
6.08
3.83
3.77
3.39
2.51
2.50
2.50
2.50
2.49
1.04
2.00
1.00
1.04
1.03
1.11
ppm

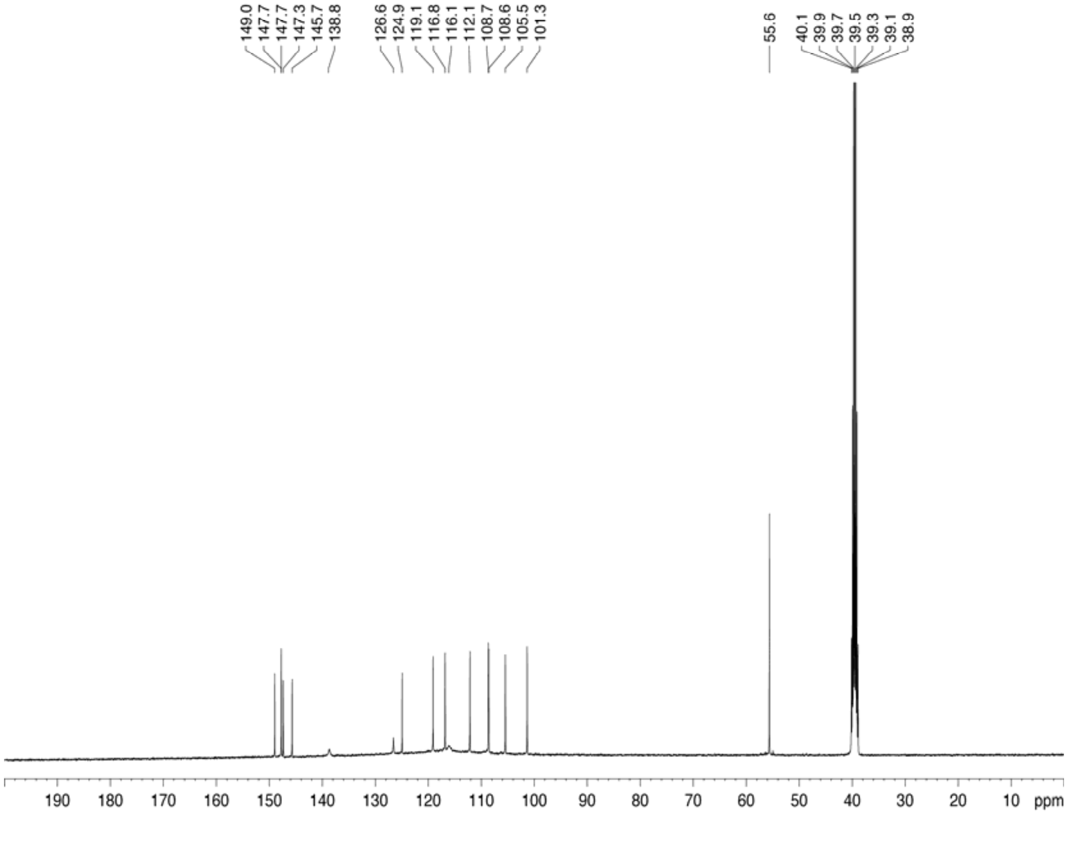

149.0
147.7
147.7
147.3
145.7
138.8
126.6
124.9
119.1
116.8
116.1
112.1
108.7
108.6
105.5
101.3
55.6
40.1
39.9
39.7
39.5
39.3
39.1
38.9
ppm

sery166B

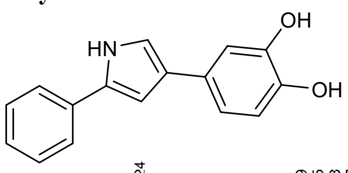
HN
OH
OH

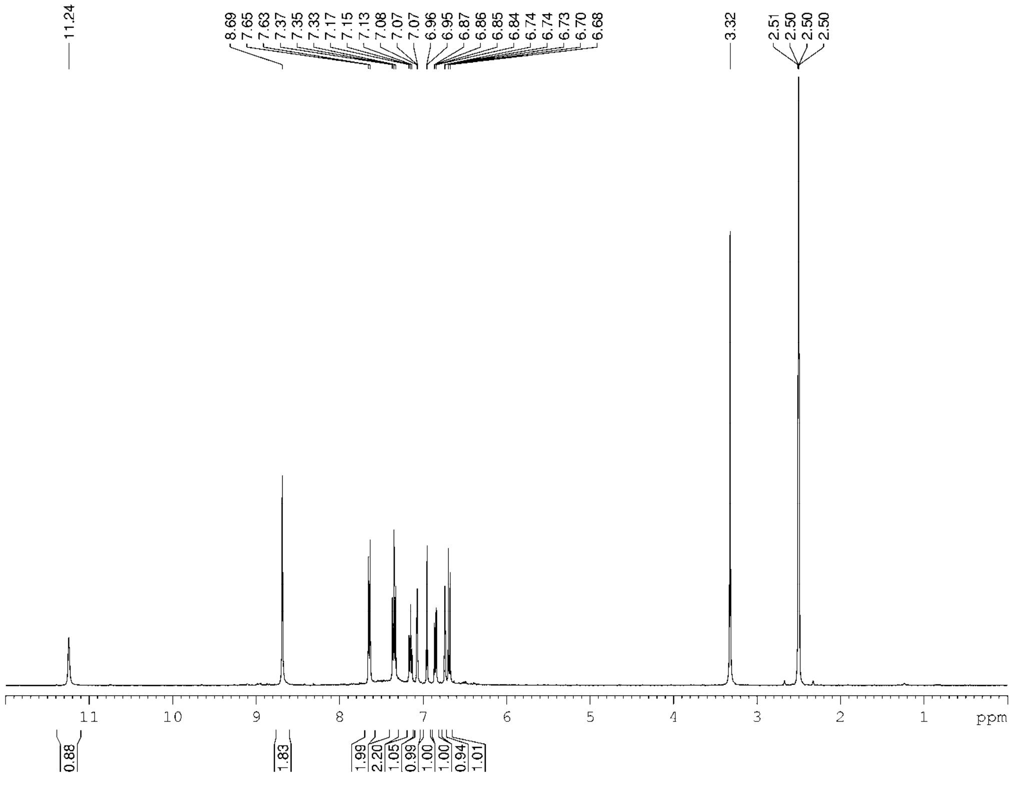
11.24
8.69 7.65 7.63 7.37 7.35 7.33 7.17 7.15 7.13 7.08 7.07 7.07 6.96 6.95 6.87 6.86 6.85 6.84 6.74 6.74 6.73 6.70 6.68
3.32
2.51 2.50 2.50 2.50
11 10 9 8 7 6 5 4 3 2 1 ppm
0.88
1.83
1.99 2.20 1.05 0.99 1.00 1.00 0.94 1.01

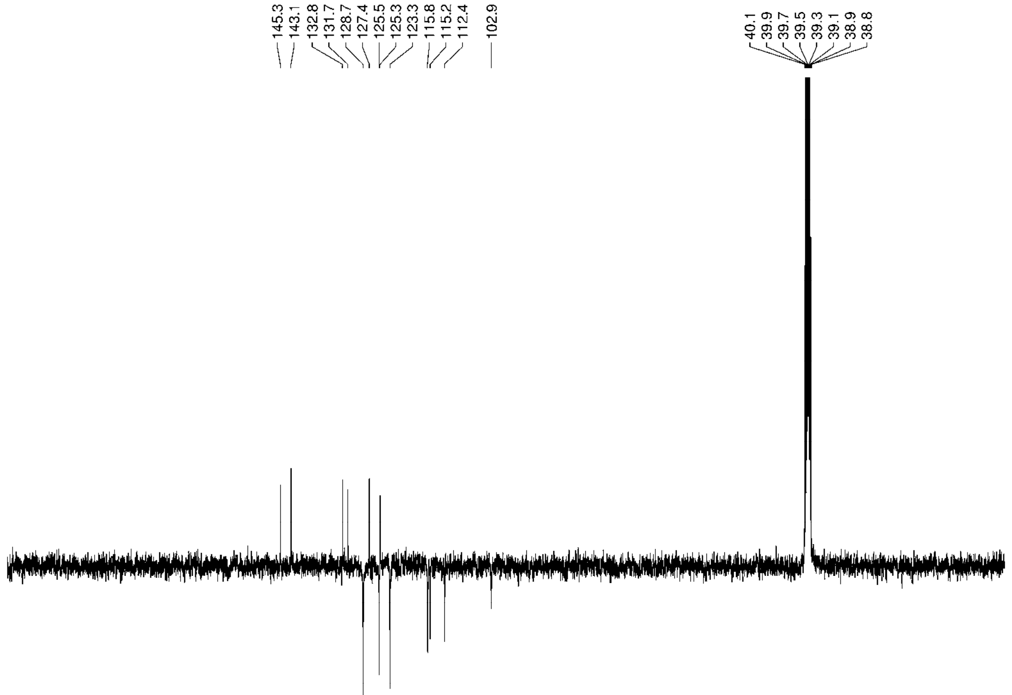
145.3 143.1 132.8 131.7 128.7 127.4 125.5 125.3 123.3 115.8 115.2 112.4
102.9
40.1 39.9 39.7 39.5 39.3 39.1 38.9 38.8
190 180 170 160 150 140 130 120 110 100 90 80 70 60 50 40 30 20 10 ppm

sery167

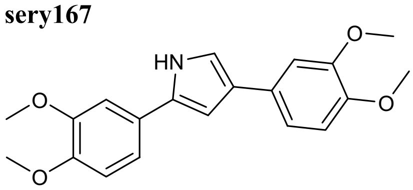

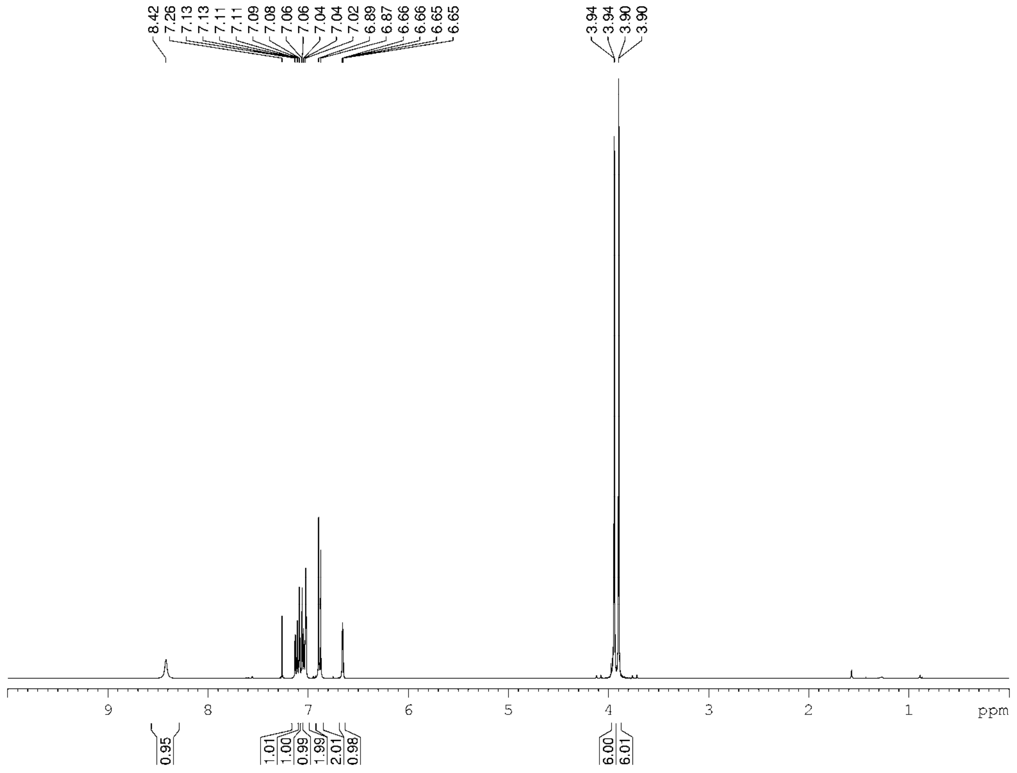

8.42
7.26
7.13
7.13
7.11
7.11
7.09
7.08
7.06
7.06
7.04
7.04
7.02
6.89
6.87
6.66
6.66
6.65
6.65
3.94
3.94
3.90
3.90
9
8
7
6
5
4
3
2
1
ppm
0.95
1.01
1.00
0.99
1.99
2.01
0.98
6.00
6.01

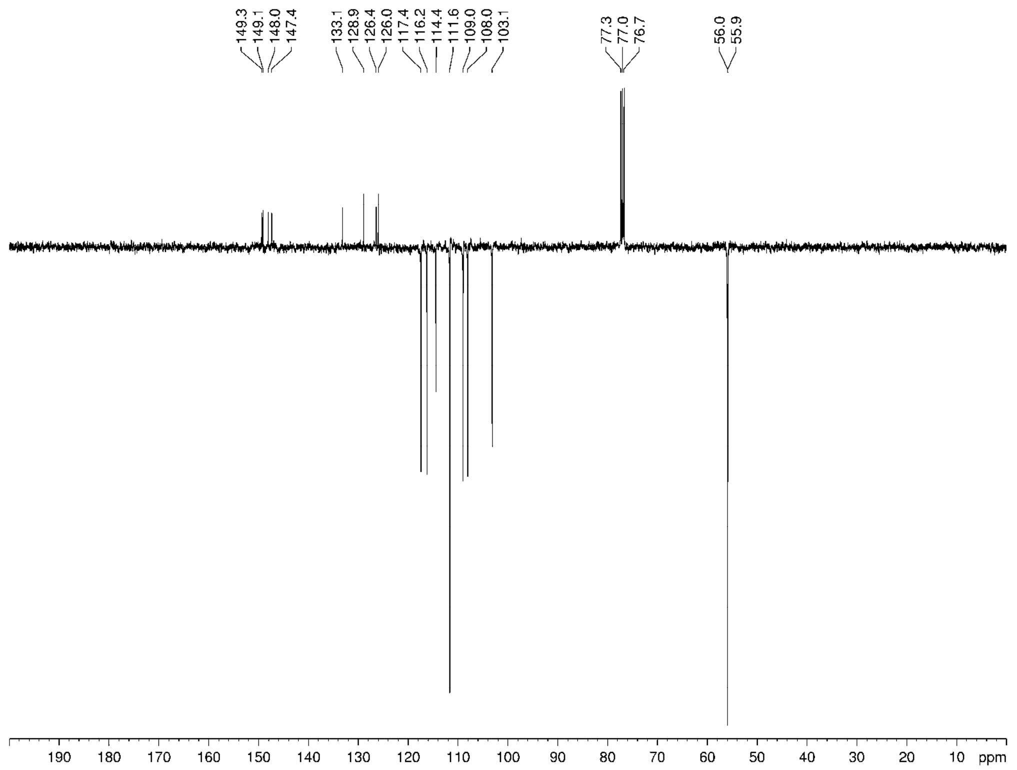

149.3
149.1
148.0
147.4
133.1
128.9
126.4
126.0
117.4
116.2
114.4
111.6
109.0
108.0
103.1
77.3
77.0
76.7
56.0
55.9
190
180
170
160
150
140
130
120
110
100
90
80
70
60
50
40
30
20
10
ppm

sery255A

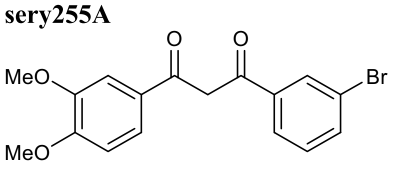
MeO
MeO
O
O
Br

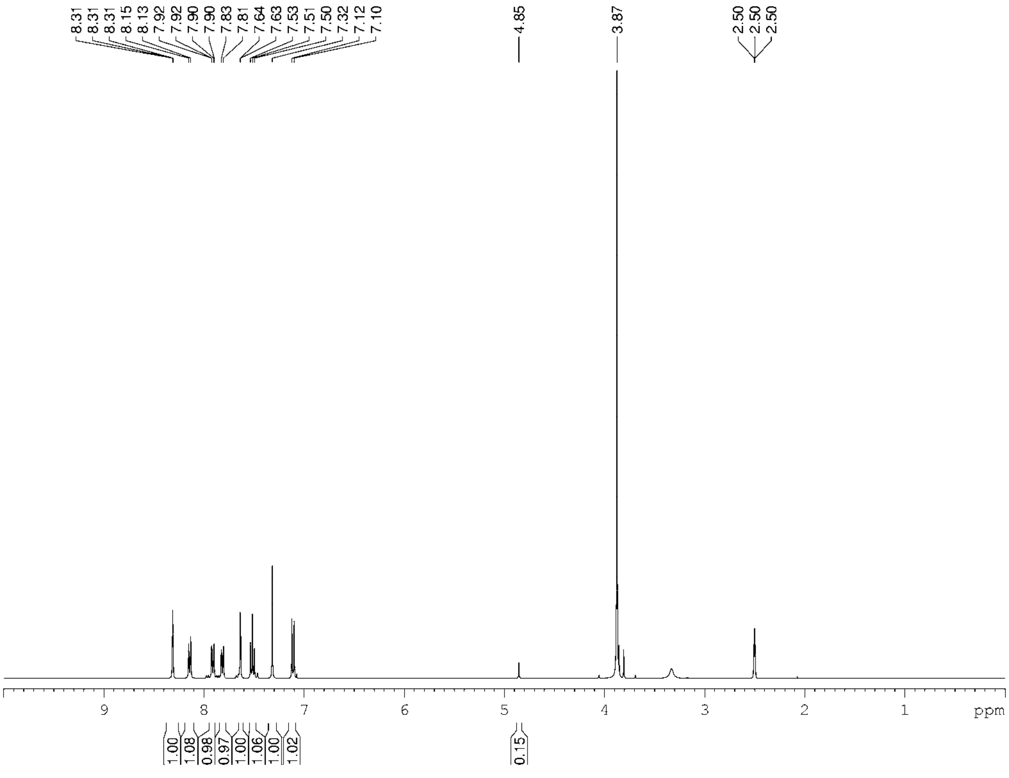
8.31
8.31
8.31
8.15
8.13
7.92
7.92
7.90
7.90
7.83
7.81
7.64
7.63
7.53
7.51
7.50
7.32
7.12
7.10
4.85
3.87
2.50
2.50
2.50
9
8
7
6
5
4
3
2
1
ppm
1.00
1.08
0.98
0.97
1.00
1.06
1.00
1.02
0.15

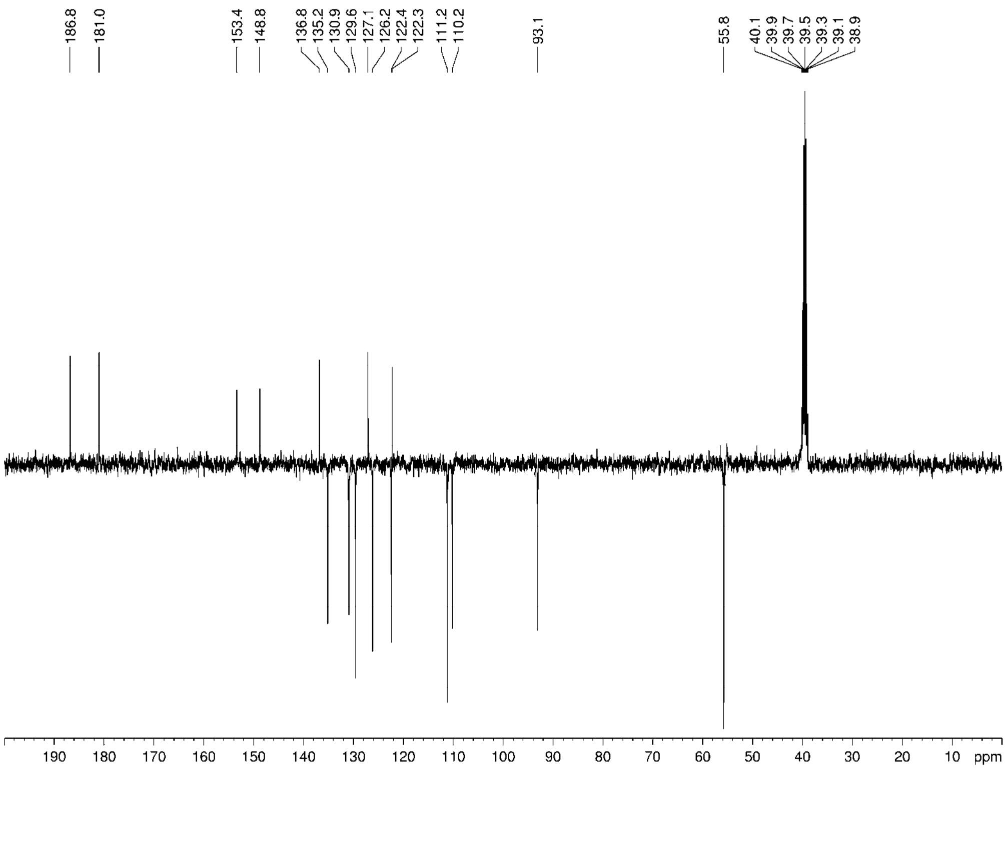
186.8
181.0
153.4
148.8
136.8
135.2
130.9
129.6
127.1
126.2
122.4
122.3
111.2
110.2
93.1
55.8
40.1
39.9
39.7
39.5
39.3
39.1
38.9
190
180
170
160
150
140
130
120
110
100
90
80
70
60
50
40
30
20
10
ppm

sery255B

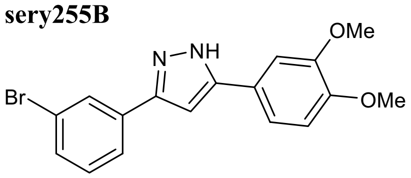
N–NH
OMe
Br
OMe

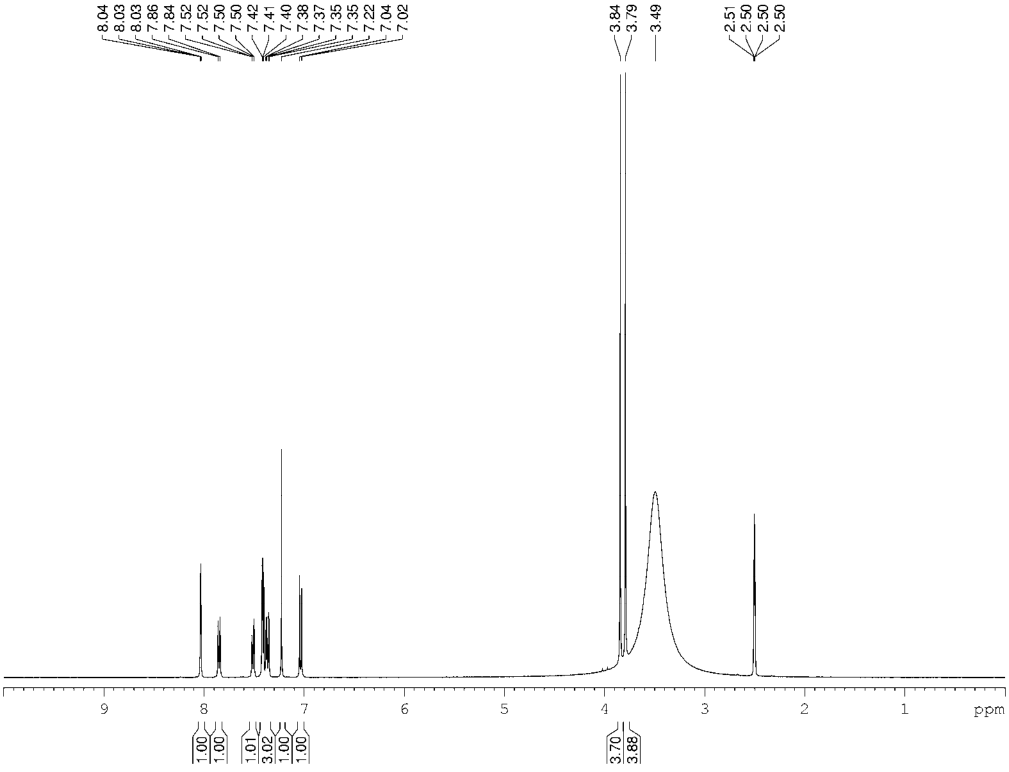
8.04 8.03 8.03 7.86 7.84 7.52 7.52 7.50 7.50 7.42 7.41 7.40 7.38 7.37 7.35 7.35 7.22 7.04 7.02
3.84 3.79 3.49
2.51 2.50 2.50 2.50
9 8 7 6 5 4 3 2 1 ppm
1.00 1.00 1.01 3.02 1.00 1.00
3.70 3.88

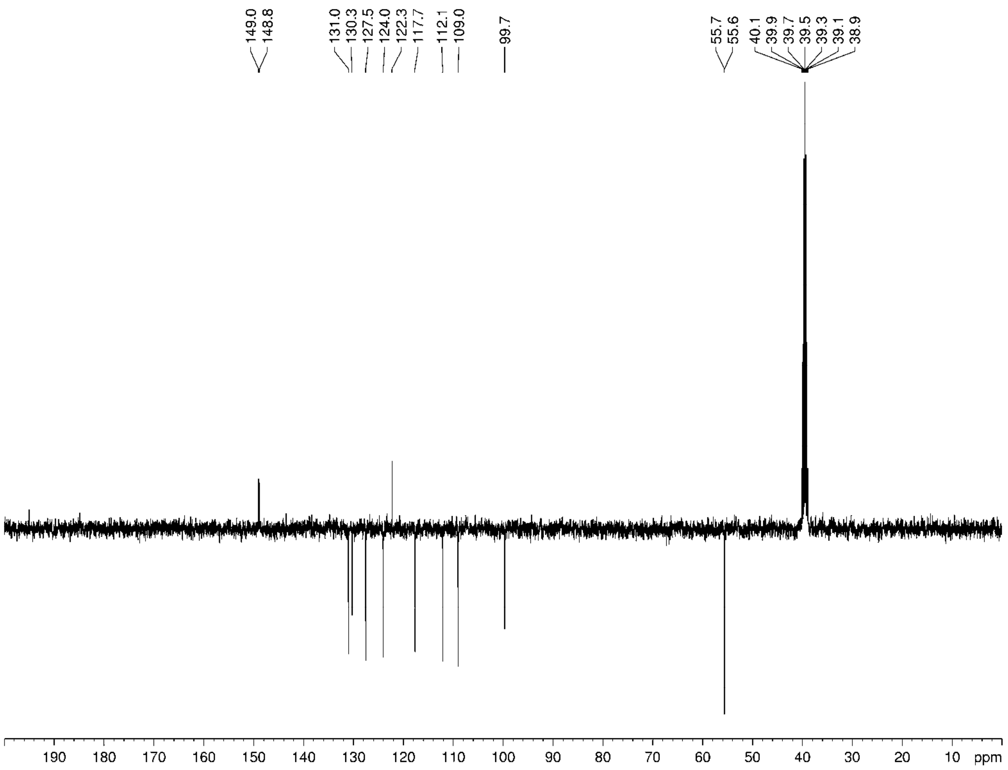
149.0 148.8
131.0 130.3 127.5 124.0 122.3 117.7 112.1 109.0
99.7
55.7 55.6 40.1 39.9 39.7 39.5 39.3 39.1 38.9
190 180 170 160 150 140 130 120 110 100 90 80 70 60 50 40 30 20 10 ppm

sery256A

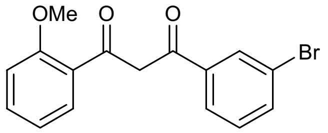

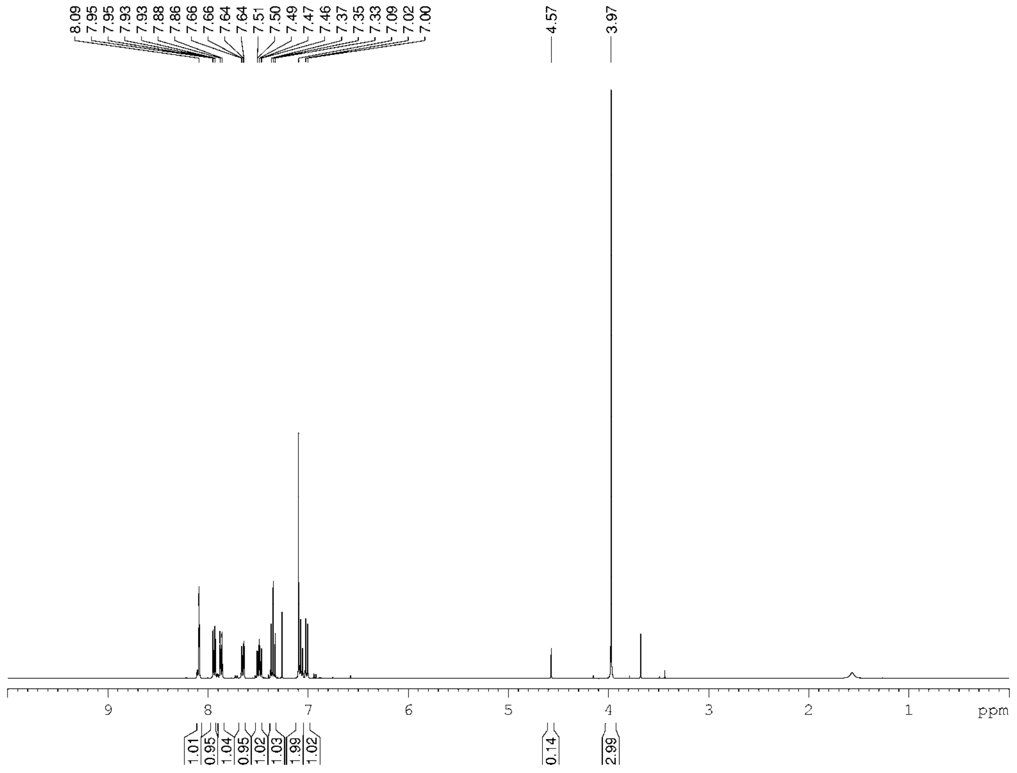
8.09
7.95
7.95
7.93
7.93
7.88
7.86
7.66
7.66
7.64
7.64
7.51
7.50
7.49
7.47
7.46
7.37
7.35
7.33
7.09
7.02
7.00
4.57
3.97
9
8
7
6
5
4
3
2
1
ppm
1.01
0.95
1.04
0.95
1.02
1.03
1.99
1.02
0.14
2.99

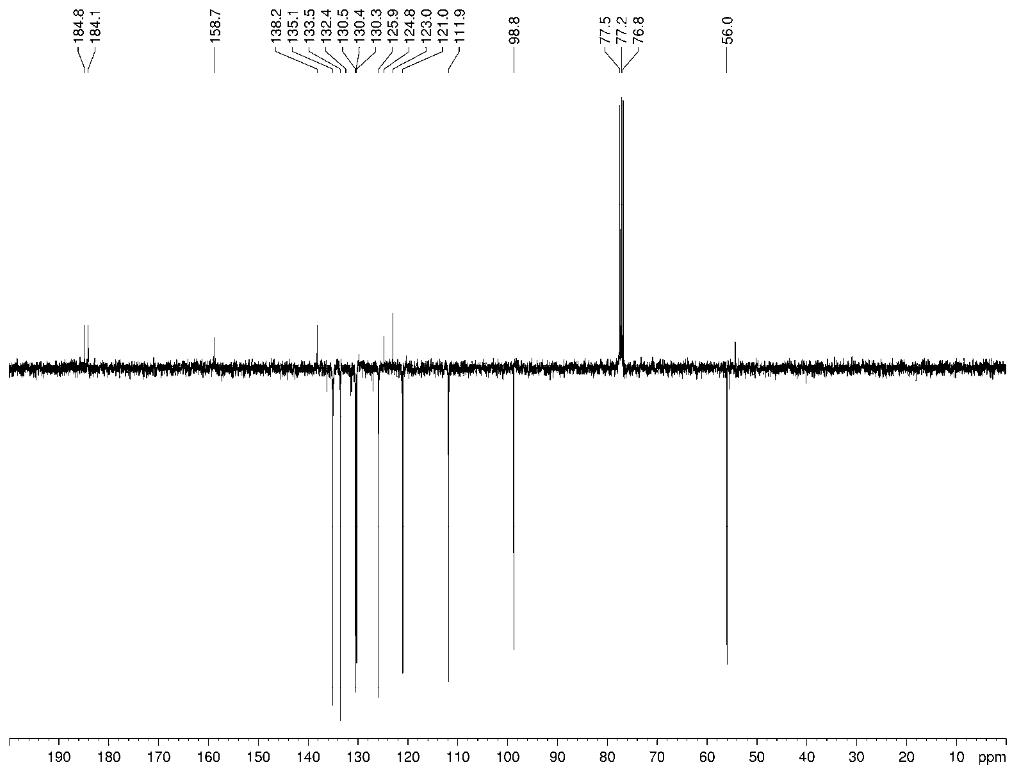
184.8
184.1
158.7
138.2
135.1
133.5
132.4
130.5
130.4
130.3
125.9
124.8
123.0
121.0
111.9
98.8
77.5
77.2
76.8
56.0
190
180
170
160
150
140
130
120
110
100
90
80
70
60
50
40
30
20
10
ppm

sery256B

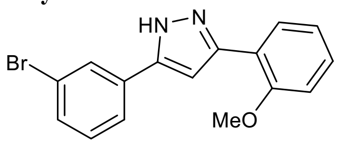
HN
N
Br
MeO

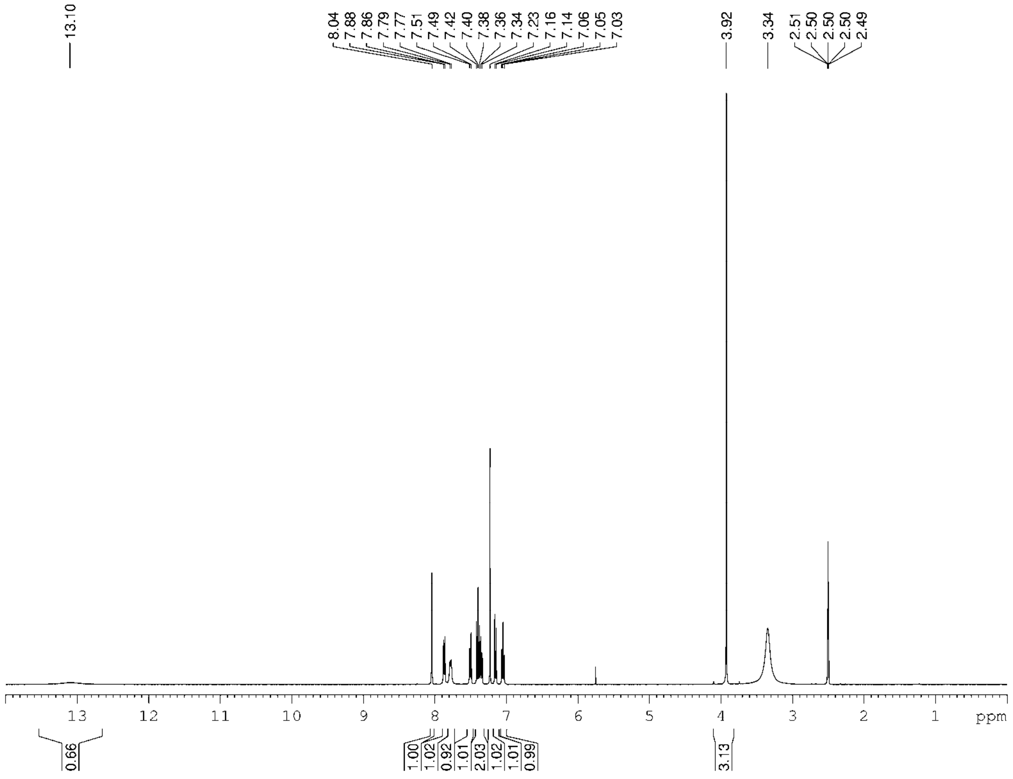
13.10
8.04
7.88
7.86
7.79
7.77
7.51
7.49
7.42
7.40
7.38
7.36
7.34
7.23
7.16
7.14
7.06
7.05
7.03
3.92
3.34
2.51
2.50
2.50
2.50
2.49
13 12 11 10 9 8 7 6 5 4 3 2 1 ppm
0.66
1.00
1.02
0.92
1.01
2.03
1.02
1.01
0.99
3.13

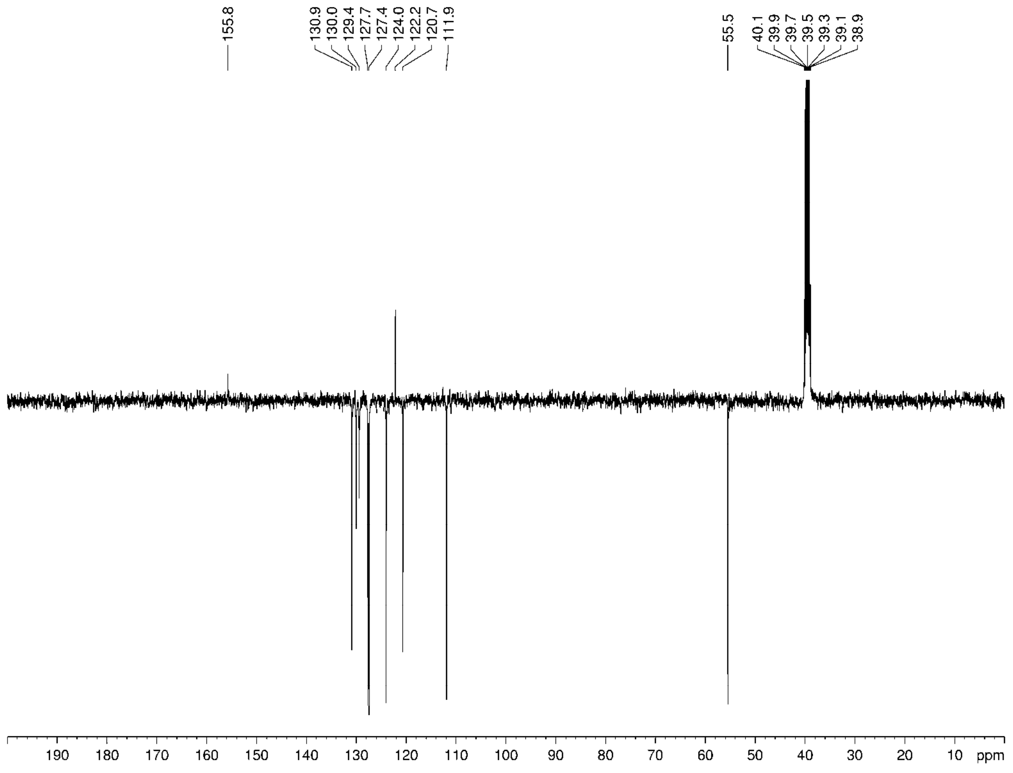
155.8
130.9
130.0
129.4
127.7
127.4
124.0
122.2
120.7
111.9
55.5
40.1
39.9
39.7
39.5
39.3
39.1
38.9
190 180 170 160 150 140 130 120 110 100 90 80 70 60 50 40 30 20 10 ppm

sery257A

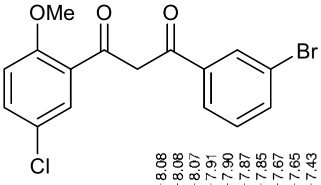

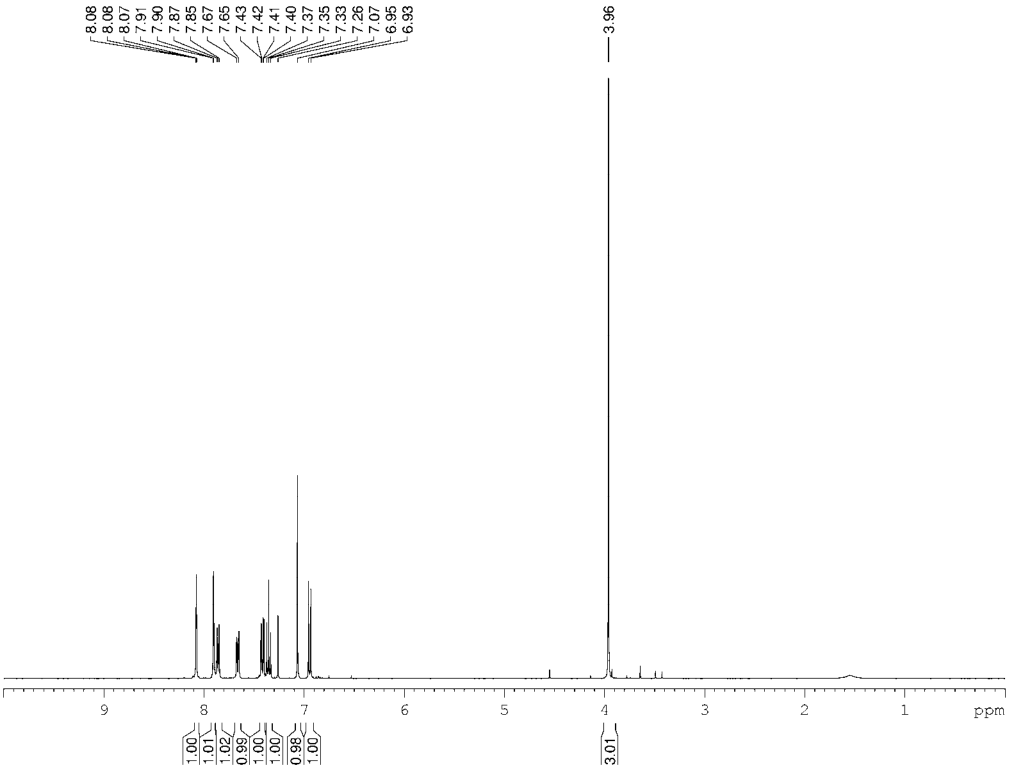

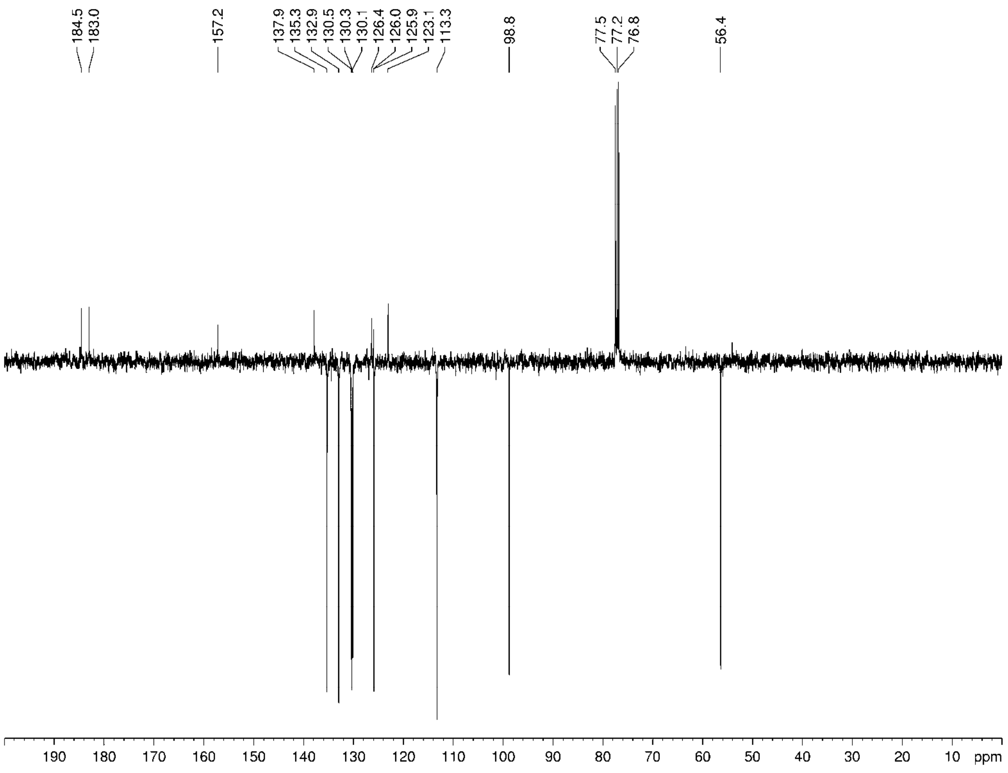

sery257B

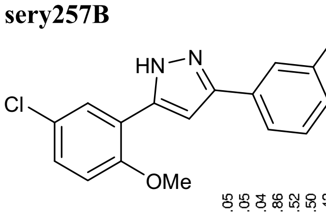
HN–N
Br
Cl
OMe

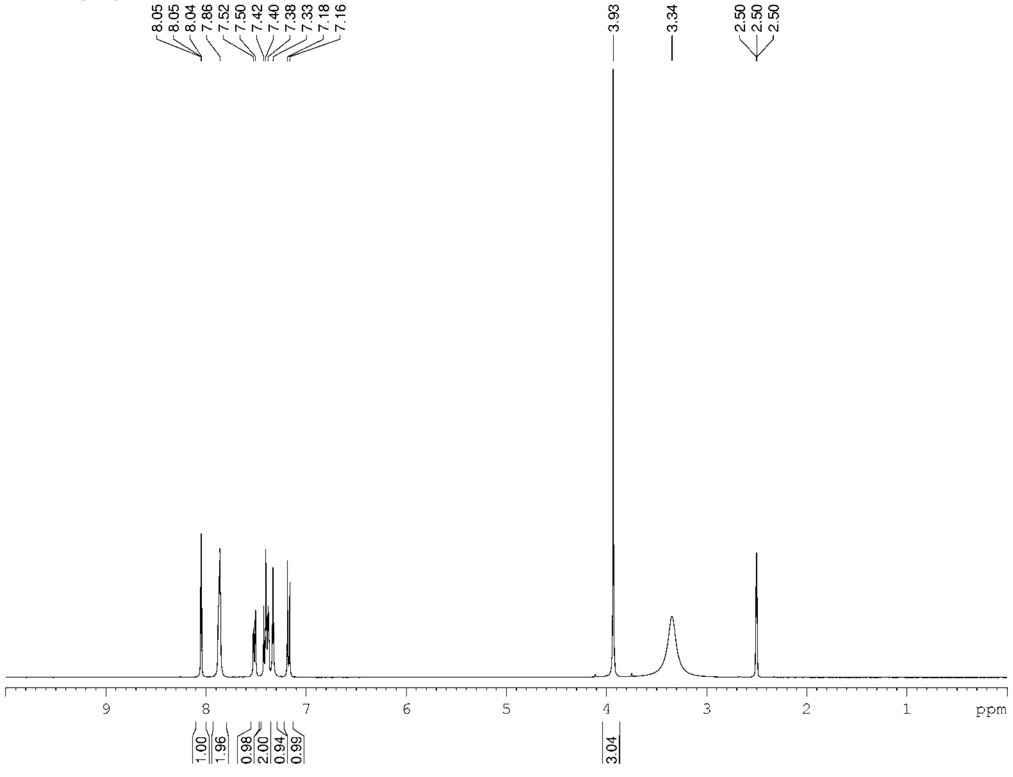
8.05
8.05
8.04
7.86
7.52
7.50
7.42
7.40
7.38
7.33
7.18
7.16
3.93
3.34
2.50
2.50
2.50
9
8
7
6
5
4
3
2
1
ppm
1.00
1.96
0.98
2.00
0.94
0.99
3.04

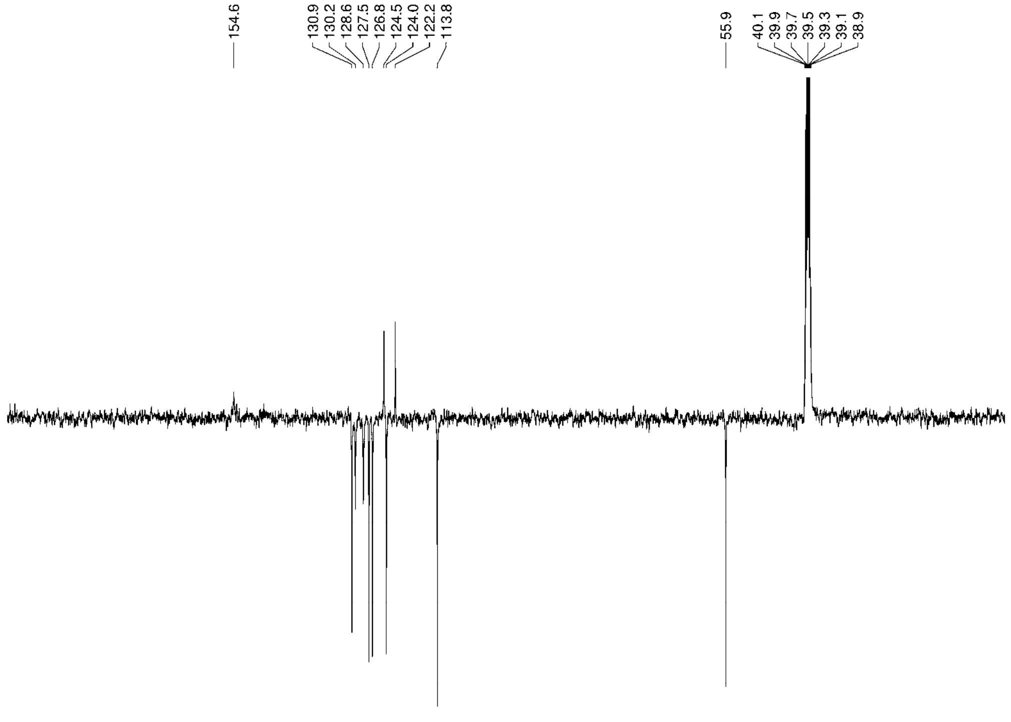
154.6
130.9
130.2
128.6
127.5
126.8
124.5
124.0
122.2
113.8
55.9
40.1
39.9
39.7
39.5
39.3
39.1
38.9
190
180
170
160
150
140
130
120
110
100
90
80
70
60
50
40
30
20
10
ppm

sery258

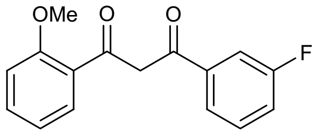

OMe
O
O
F

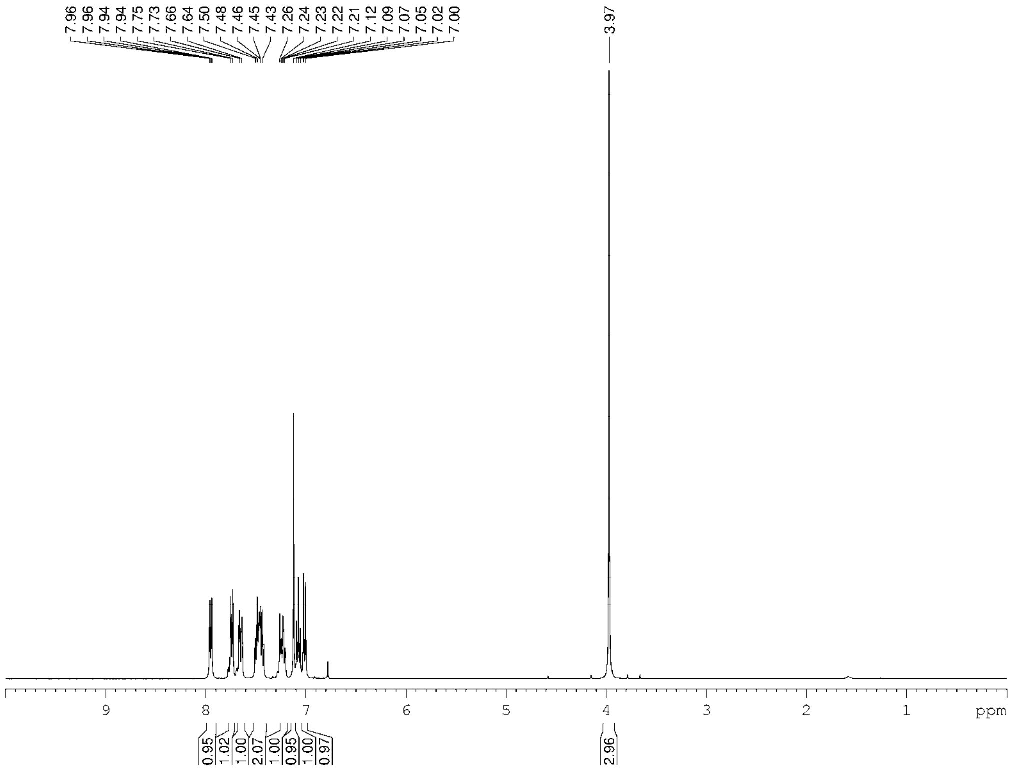

7.96
7.96
7.94
7.94
7.75
7.73
7.66
7.64
7.50
7.48
7.46
7.45
7.43
7.26
7.24
7.23
7.22
7.21
7.12
7.09
7.07
7.05
7.02
7.00
3.97
9
8
7
6
5
4
3
2
1
ppm
0.95
1.02
1.00
2.07
1.00
0.95
1.00
0.97
2.96

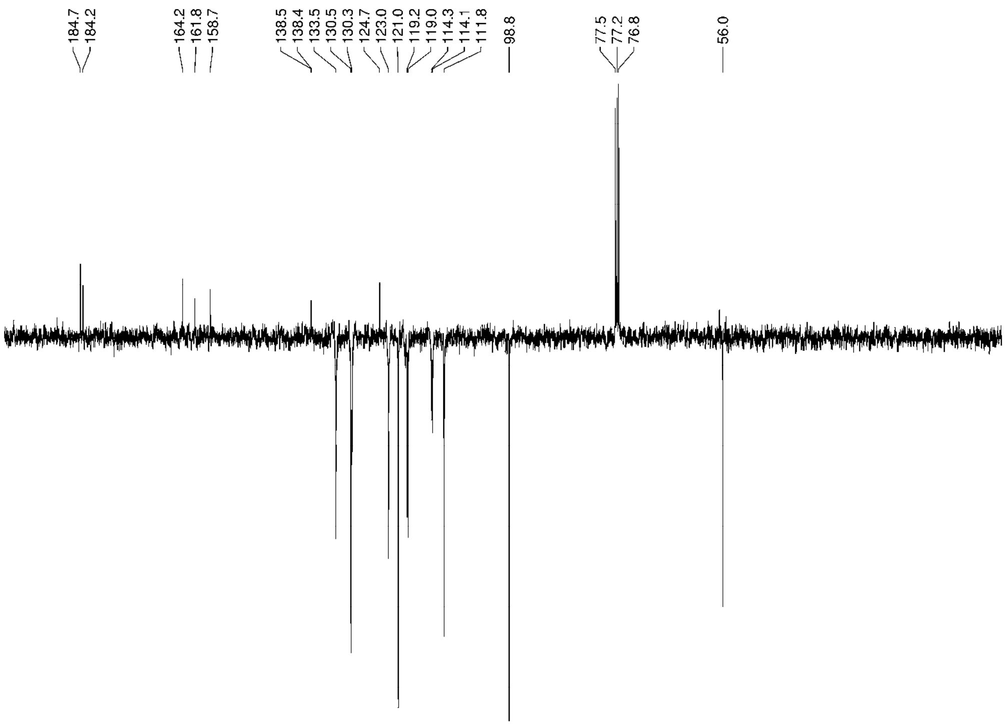

184.7
184.2
164.2
161.8
158.7
138.5
138.4
133.5
130.5
130.3
124.7
123.0
121.0
119.2
119.0
114.3
114.1
111.8
98.8
77.5
77.2
76.8
56.0
190
180
170
160
150
140
130
120
110
100
90
80
70
60
50
40
30
20
10
ppm

sery259

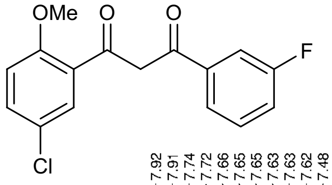
OMe
O
O
F
Cl

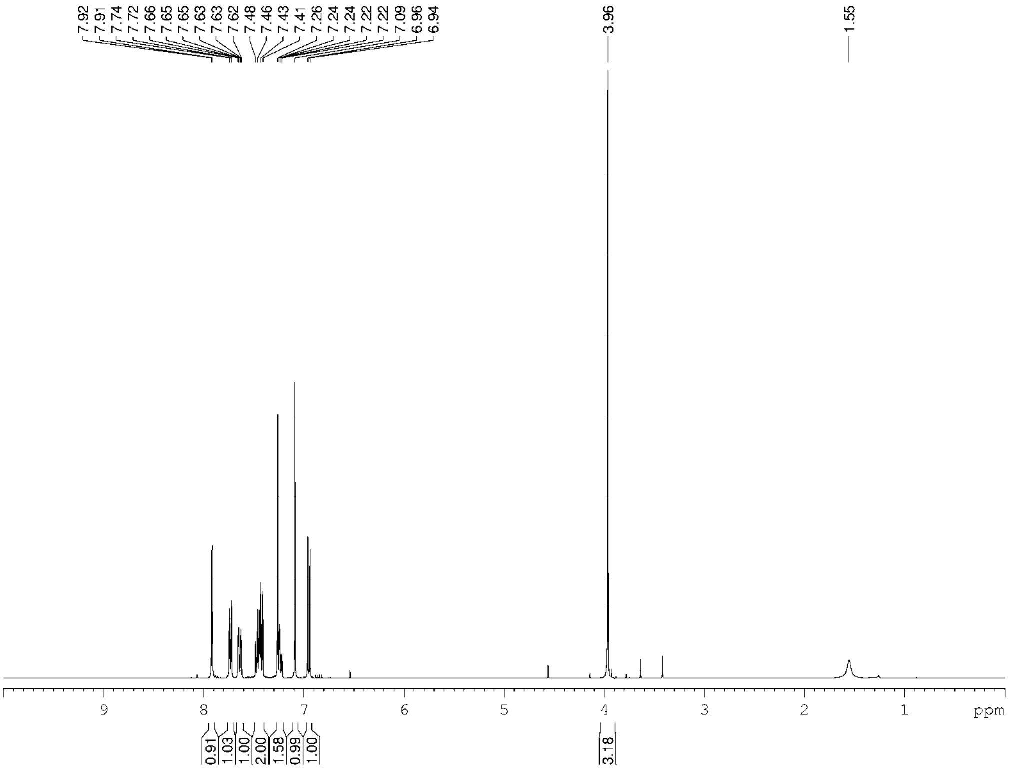
7.92
7.91
7.74
7.72
7.66
7.65
7.65
7.63
7.63
7.62
7.48
7.46
7.43
7.41
7.26
7.24
7.24
7.22
7.22
7.22
7.09
6.96
6.94
3.96
1.55
9
8
7
6
5
4
3
2
1
ppm
0.91
1.03
1.00
2.00
1.58
0.99
1.00
3.18

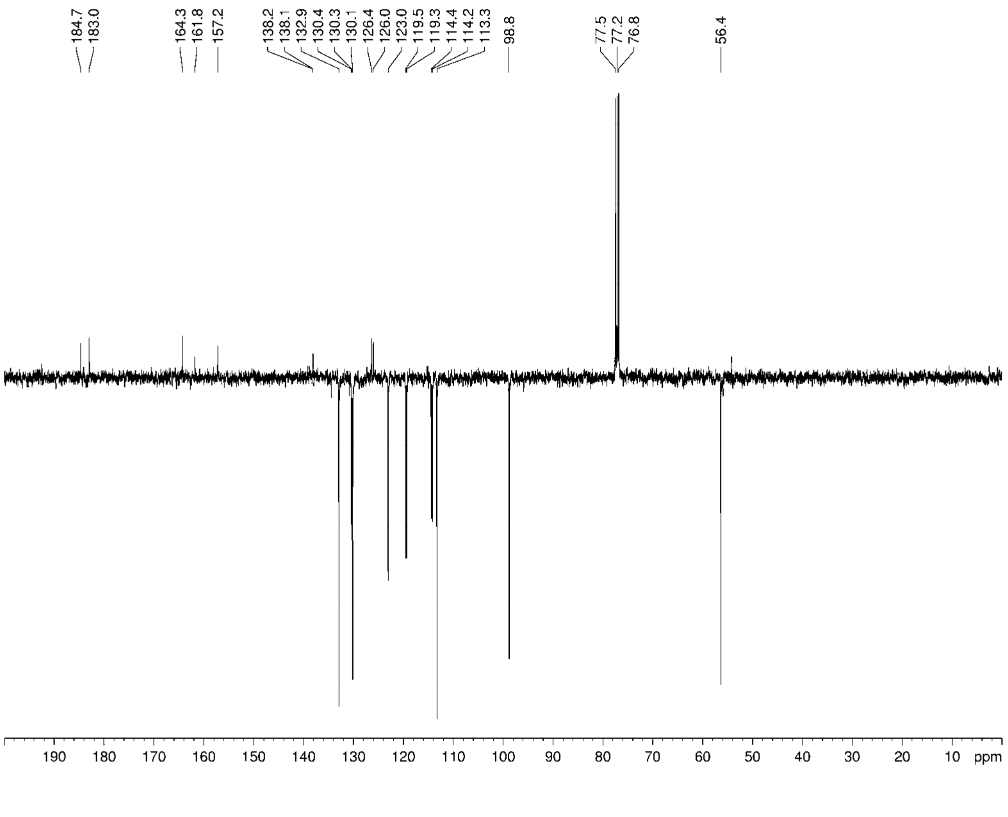
184.7
183.0
164.3
161.8
157.2
138.2
138.1
132.9
130.4
130.3
130.1
126.4
126.0
123.0
119.5
119.3
114.4
114.2
113.3
98.8
77.5
77.2
76.8
56.4
190
180
170
160
150
140
130
120
110
100
90
80
70
60
50
40
30
20
10
ppm

sery260A

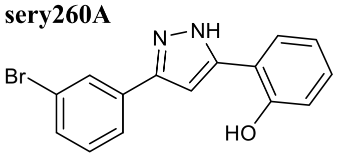
N–NH
Br
HO

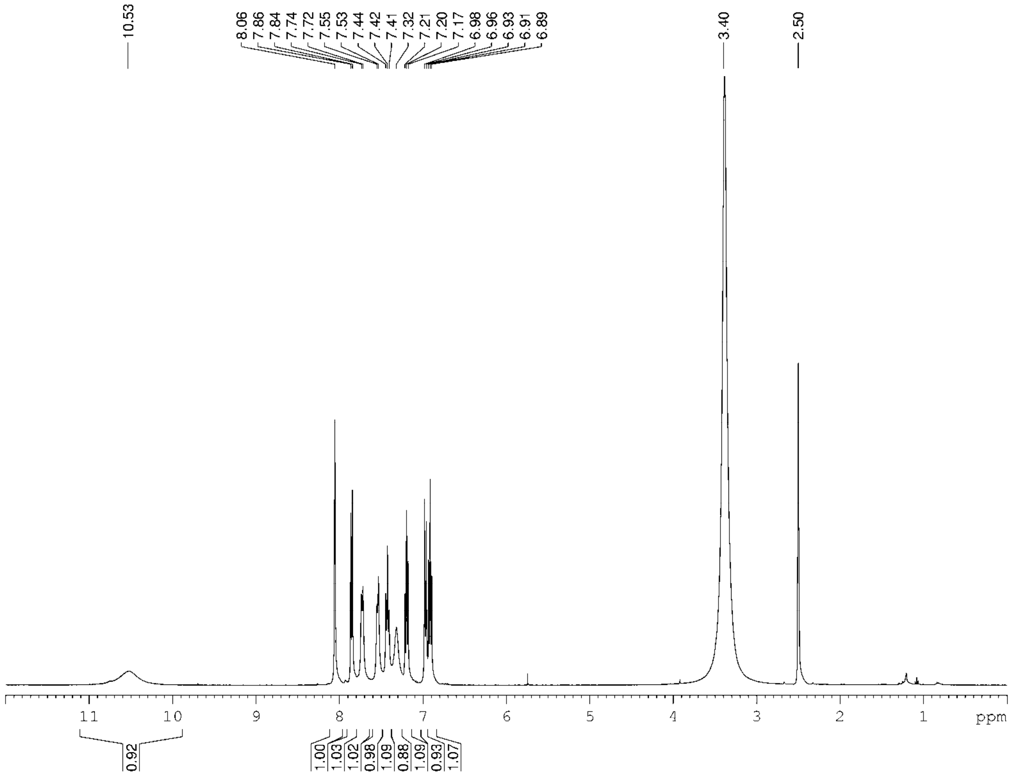
10.53
8.06
7.86
7.84
7.74
7.72
7.55
7.53
7.44
7.42
7.41
7.32
7.21
7.20
7.17
6.98
6.96
6.93
6.91
6.89
3.40
2.50
11 10 9 8 7 6 5 4 3 2 1 ppm
0.92
1.00
1.03
1.02
0.98
1.09
0.88
1.09
0.93
1.07

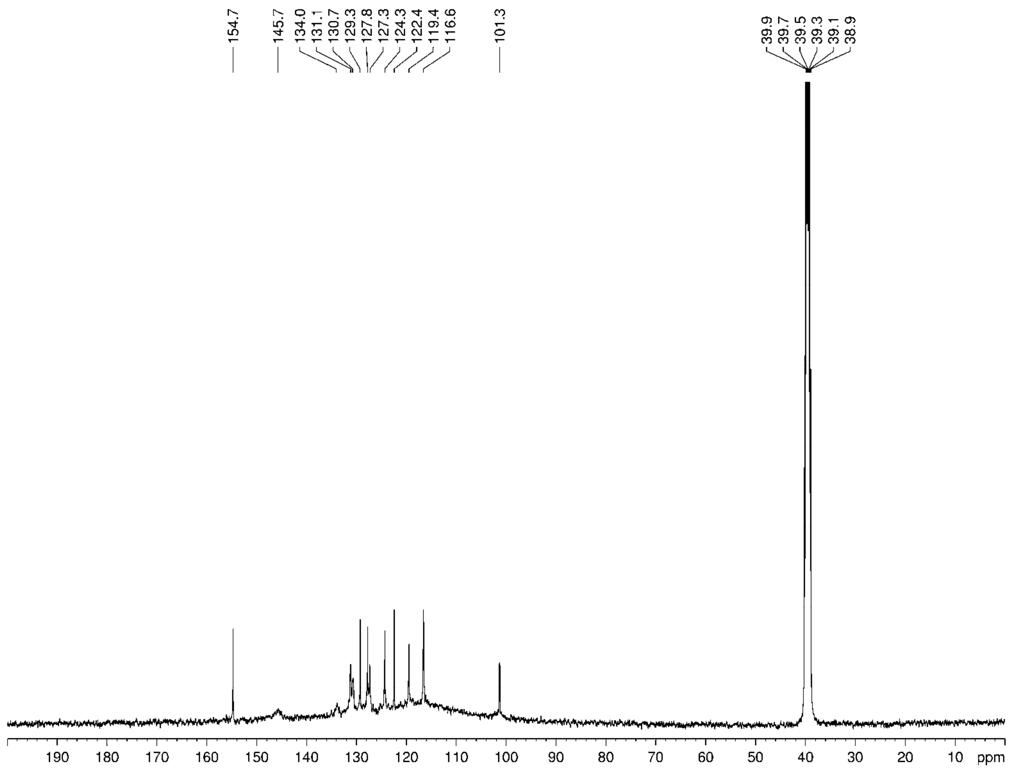
154.7
145.7
134.0
131.1
130.7
129.3
127.8
127.3
124.3
122.4
119.4
116.6
101.3
39.9
39.7
39.5
39.3
39.1
38.9
190 180 170 160 150 140 130 120 110 100 90 80 70 60 50 40 30 20 10 ppm

sery260B

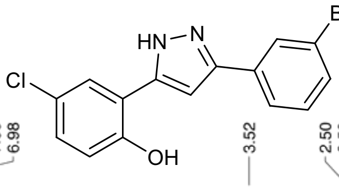

Br
HN–N
Cl
OH

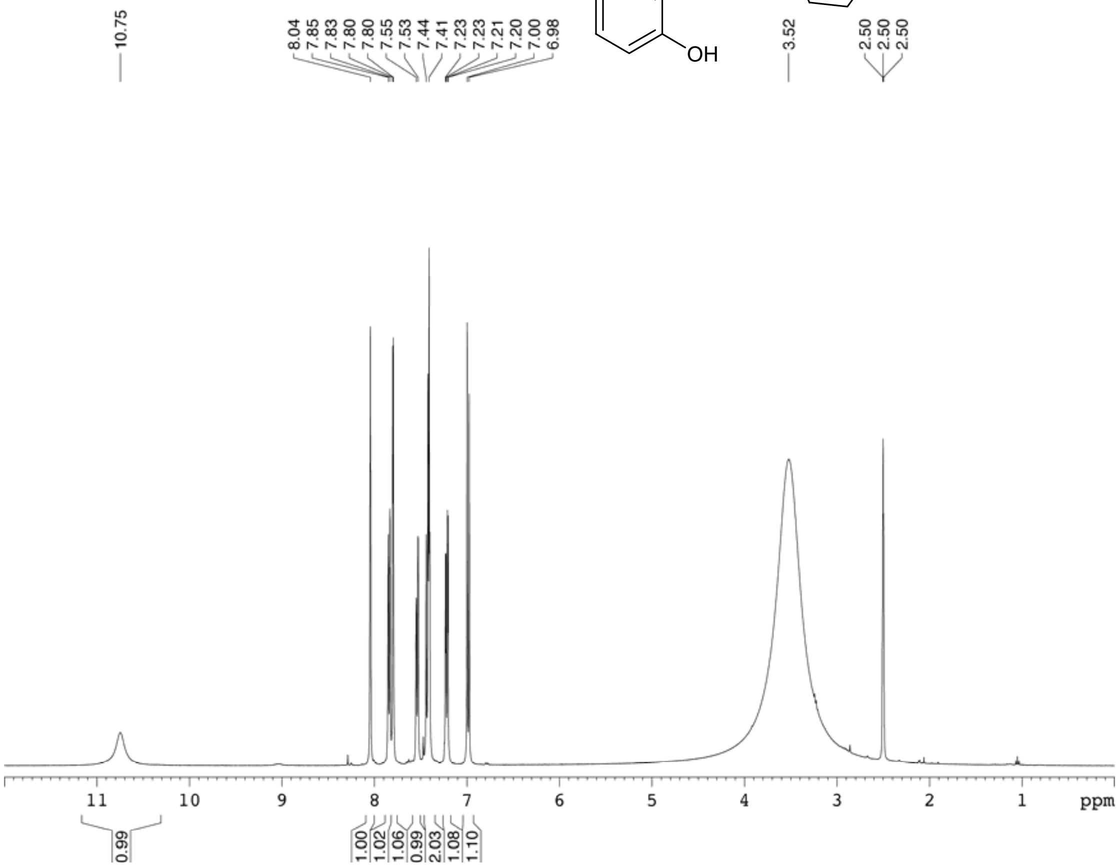

10.75
8.04 7.85 7.83 7.80 7.80 7.55 7.53 7.44 7.41 7.23 7.23 7.21 7.20 7.00 6.98
3.52
2.50 2.50 2.50
11 10 9 8 7 6 5 4 3 2 1 ppm
0.99
1.00 1.02 1.06 0.99 2.03 1.08 1.10

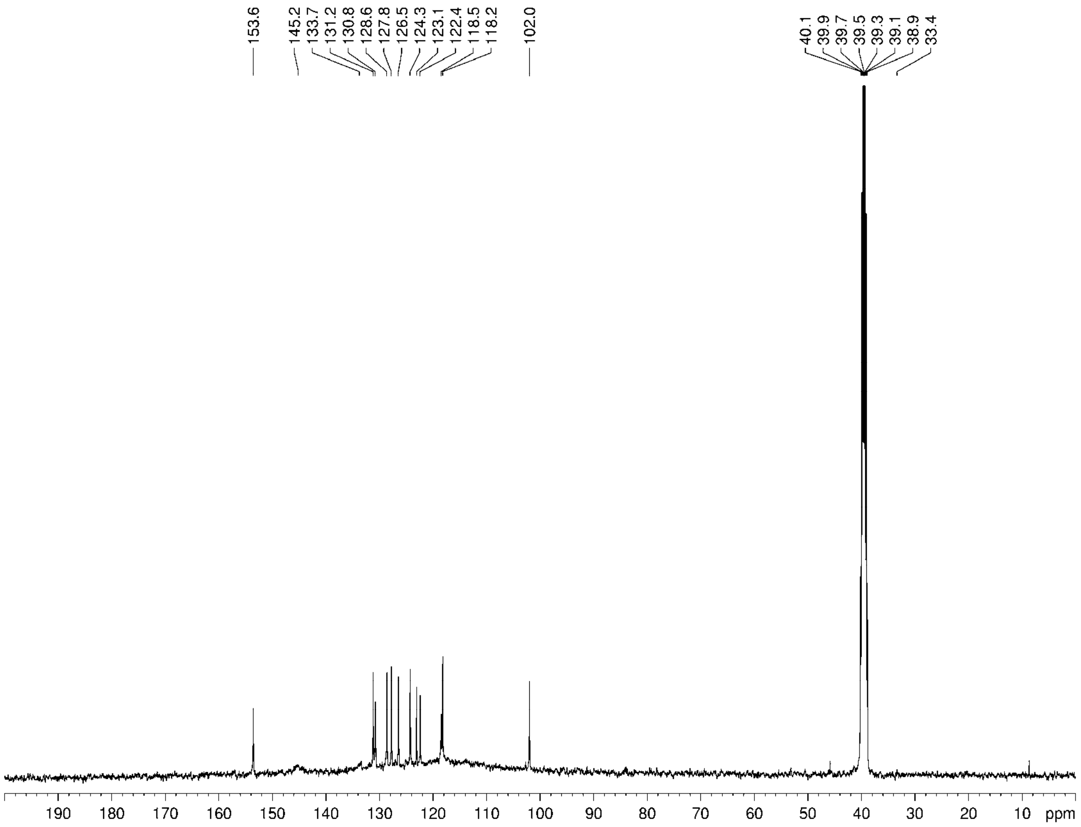

153.6
145.2 133.7 131.2 130.8 128.6 127.8 126.5 124.3 123.1 122.4 118.5 118.2
102.0
40.1 39.9 39.7 39.5 39.3 39.1 38.9 33.4
190 180 170 160 150 140 130 120 110 100 90 80 70 60 50 40 30 20 10 ppm

sery261B

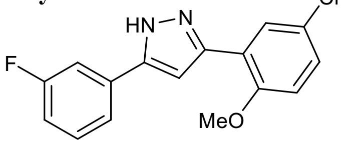
HN–N
Cl
F
MeO

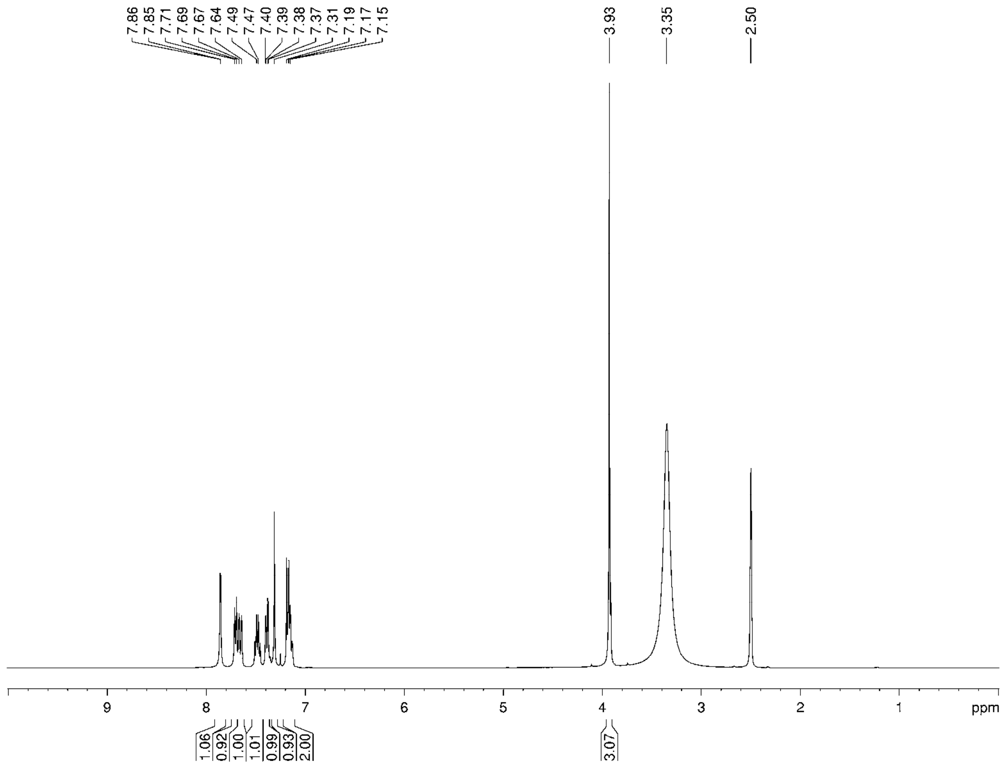
7.86
7.85
7.71
7.69
7.67
7.64
7.49
7.47
7.40
7.39
7.38
7.37
7.31
7.19
7.17
7.15
3.93
3.35
2.50
9
8
7
6
5
4
3
2
1
ppm
1.06
0.92
1.00
1.01
0.99
0.93
2.00
3.07

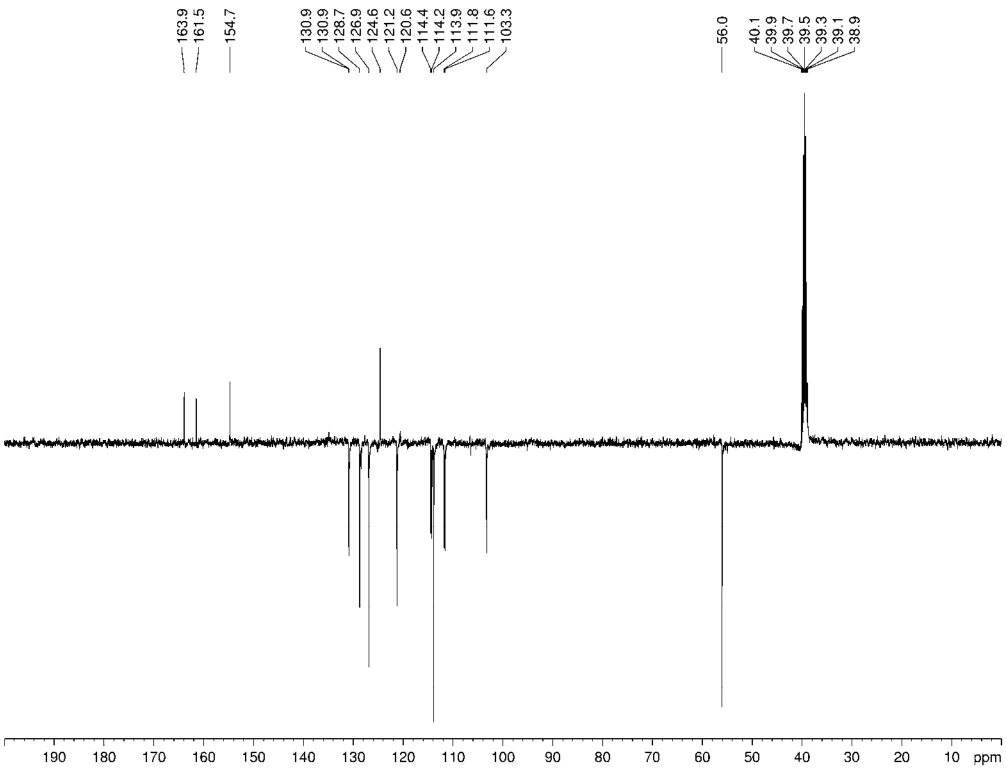
163.9
161.5
154.7
130.9
130.9
128.7
126.9
124.6
121.2
120.6
114.4
114.2
113.9
111.8
111.6
103.3
56.0
40.1
39.9
39.7
39.5
39.3
39.1
38.9
190
180
170
160
150
140
130
120
110
100
90
80
70
60
50
40
30
20
10
ppm

sery263B

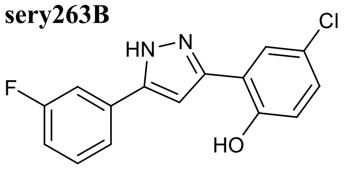

HN
N
Cl
F
HO

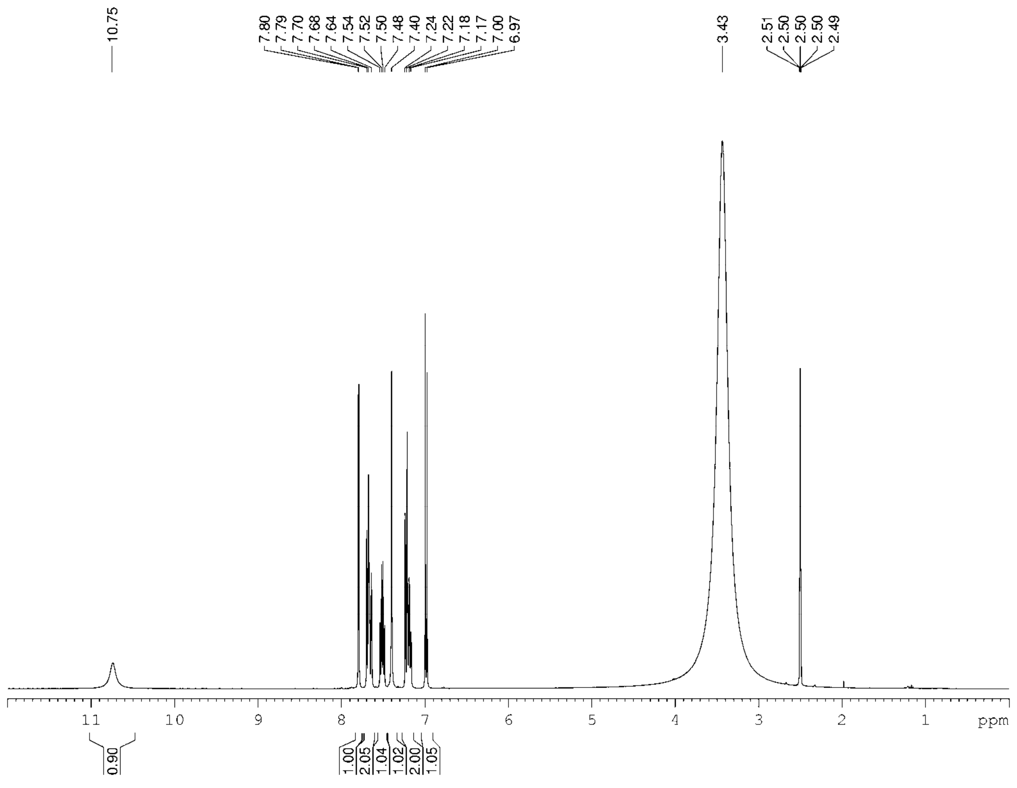

10.75
7.80
7.79
7.70
7.68
7.64
7.54
7.52
7.50
7.48
7.40
7.24
7.22
7.18
7.17
7.00
6.97
3.43
2.51
2.50
2.50
2.50
2.49
11
10
9
8
7
6
5
4
3
2
1
ppm
0.90
1.00
2.05
1.04
1.02
2.00
1.05

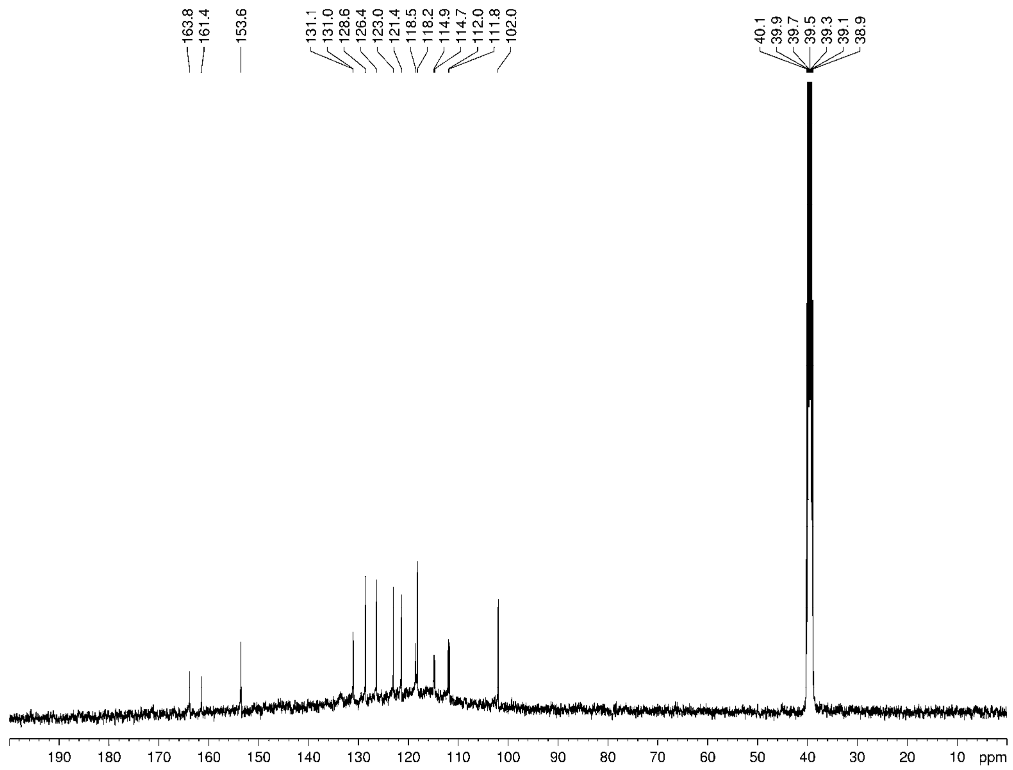

163.8
161.4
153.6
131.1
131.0
128.6
126.4
123.0
121.4
118.5
118.2
114.9
114.7
112.0
111.8
102.0
40.1
39.9
39.7
39.5
39.3
39.1
38.9
190
180
170
160
150
140
130
120
110
100
90
80
70
60
50
40
30
20
10
ppm

sery273A

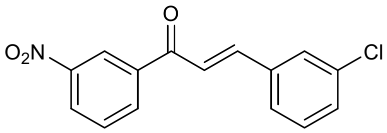

O
O_2N
Cl

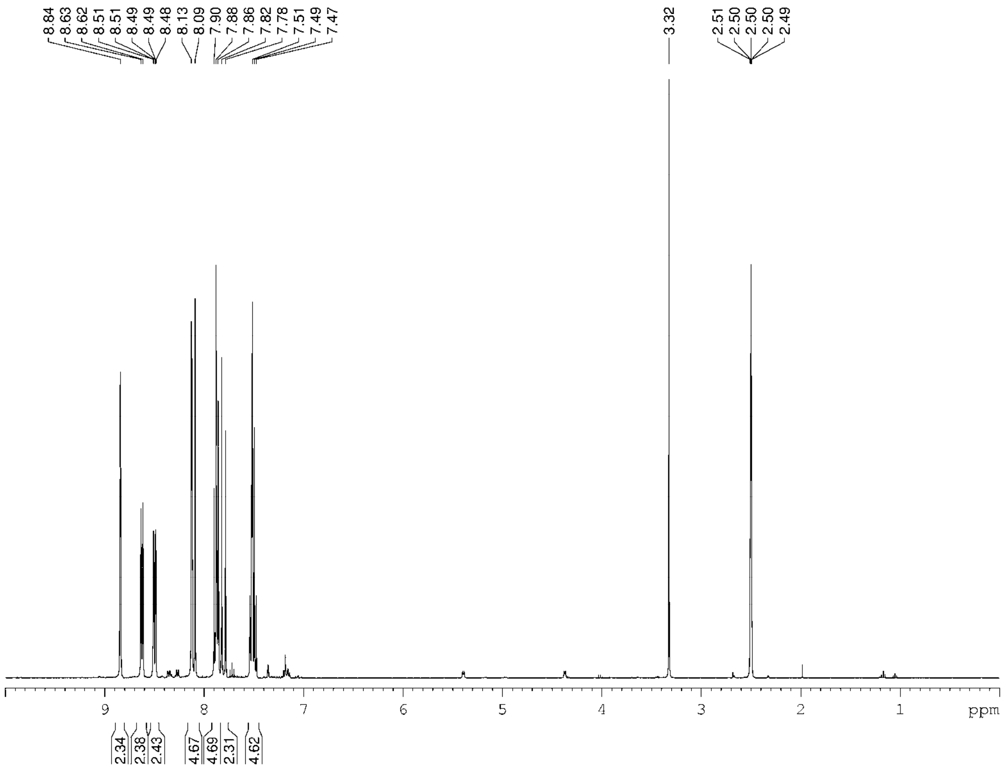

8.84 8.63 8.62 8.51 8.51 8.49 8.49 8.48 8.13 8.09 7.90 7.88 7.86 7.82 7.78 7.51 7.49 7.47
3.32
2.51 2.50 2.50 2.50 2.49
2.34 2.38 2.43 4.67 4.69 2.31 4.62
9 8 7 6 5 4 3 2 1 ppm

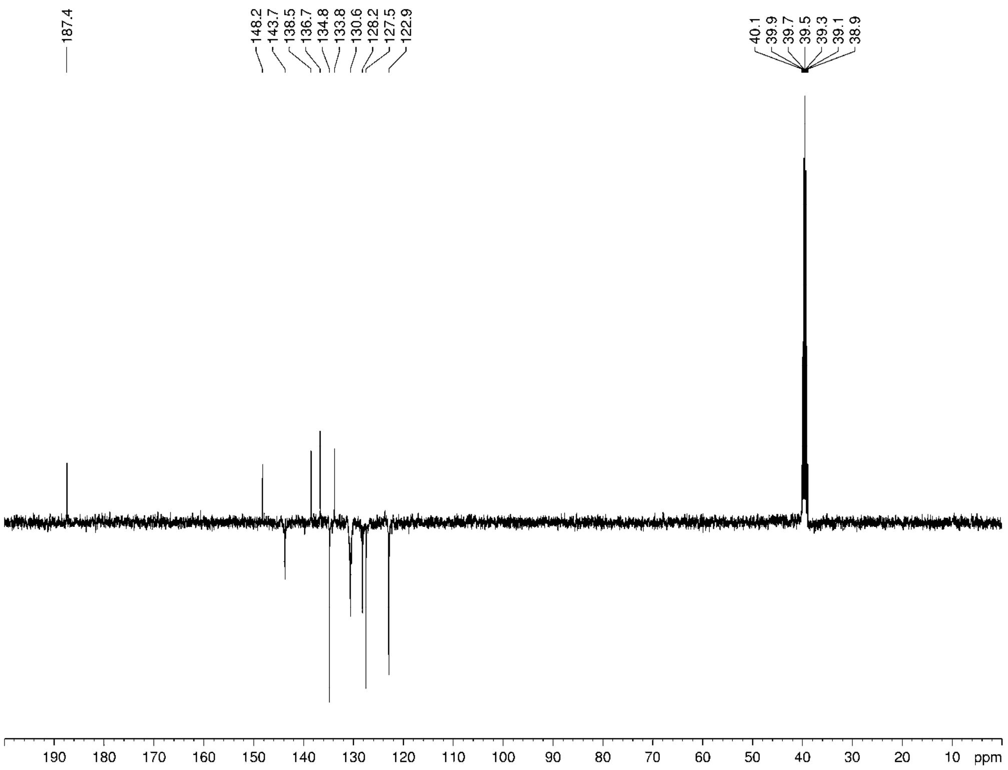

187.4
148.2 143.7 138.5 136.7 134.8 133.8 130.6 128.2 127.5 122.9
40.1 39.9 39.7 39.5 39.3 39.1 38.9
190 180 170 160 150 140 130 120 110 100 90 80 70 60 50 40 30 20 10 ppm

sery273B

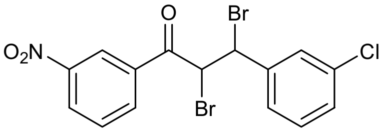

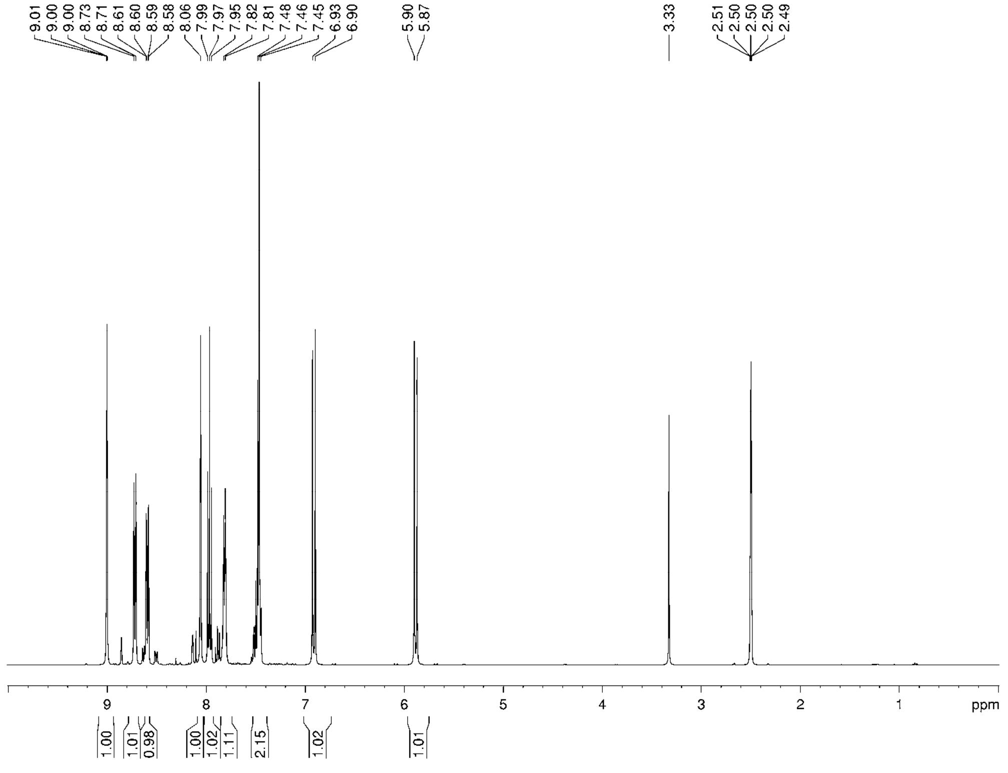
9.01
9.00
9.00
8.73
8.71
8.61
8.60
8.59
8.58
8.06
7.99
7.97
7.95
7.82
7.81
7.48
7.46
7.45
6.93
6.90
5.90
5.87
3.33
2.51
2.50
2.50
2.50
2.49
1.00
1.01
0.98
1.00
1.02
1.11
2.15
1.02
1.01
ppm

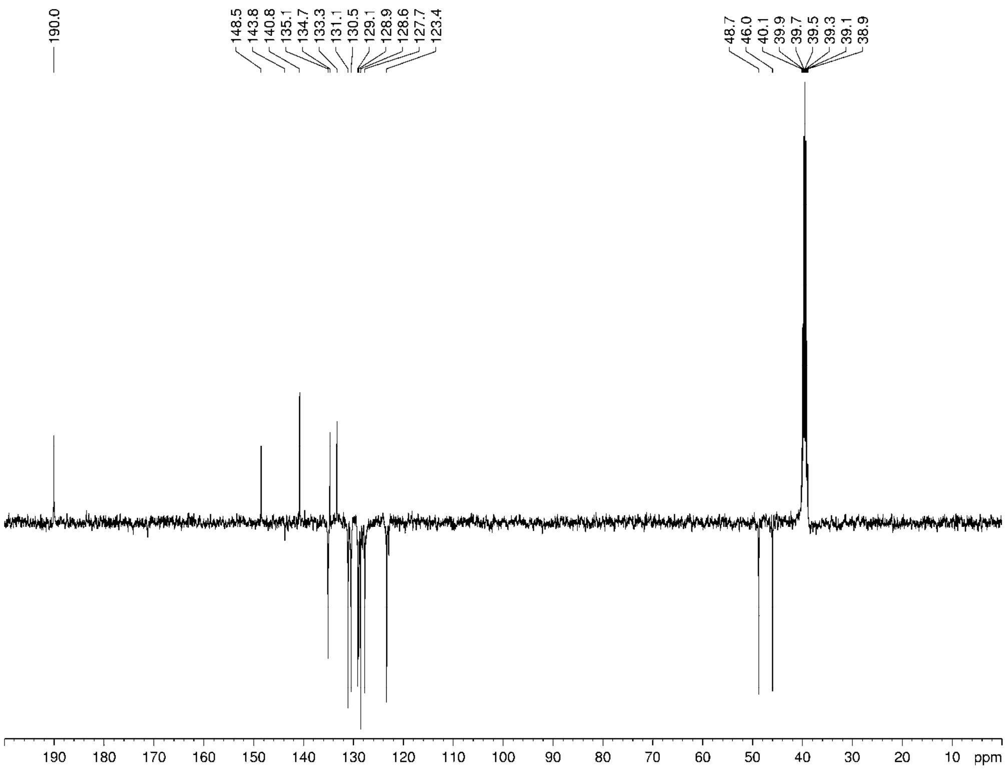
190.0
148.5
143.8
140.8
135.1
134.7
133.3
131.1
130.5
129.1
128.9
128.6
127.7
123.4
48.7
46.0
40.1
39.9
39.7
39.5
39.3
39.1
38.9
ppm

sery275A

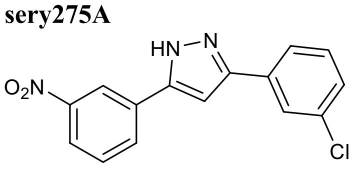
HN
N
O2N
Cl

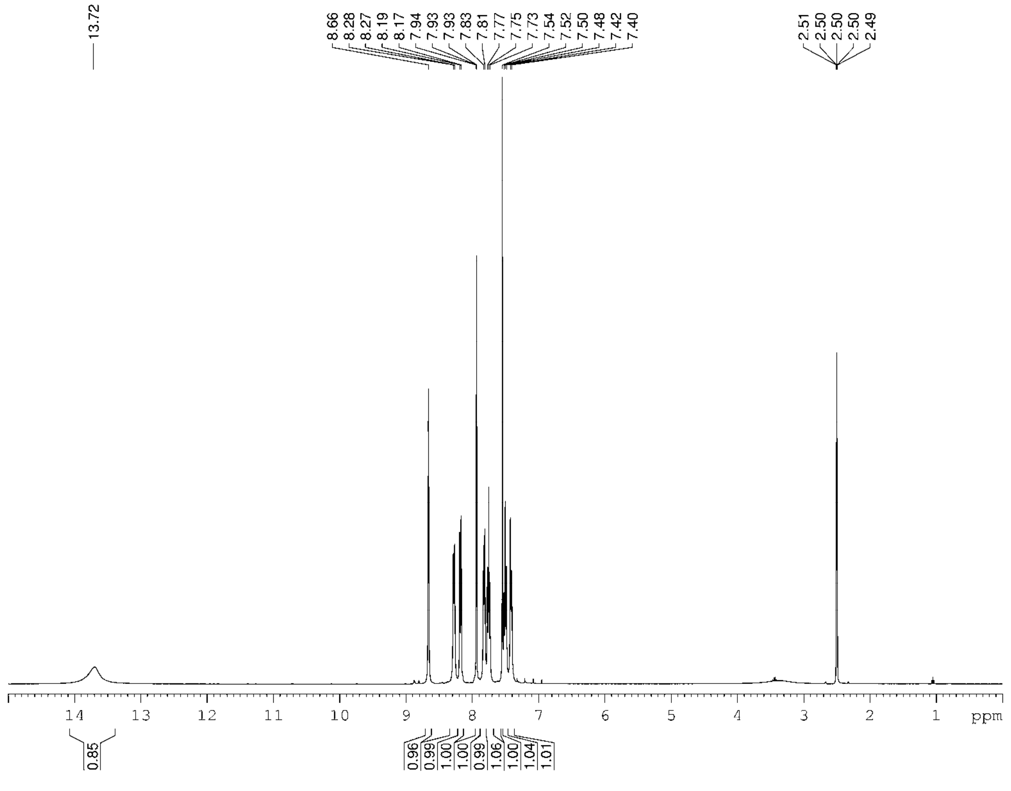
13.72
8.66
8.28
8.27
8.19
8.17
7.94
7.93
7.93
7.83
7.81
7.77
7.75
7.73
7.54
7.52
7.50
7.48
7.42
7.40
2.51
2.50
2.50
2.50
2.49
14 13 12 11 10 9 8 7 6 5 4 3 2 1 ppm
0.85
0.96
0.99
1.00
1.00
0.99
1.06
1.00
1.04
1.01

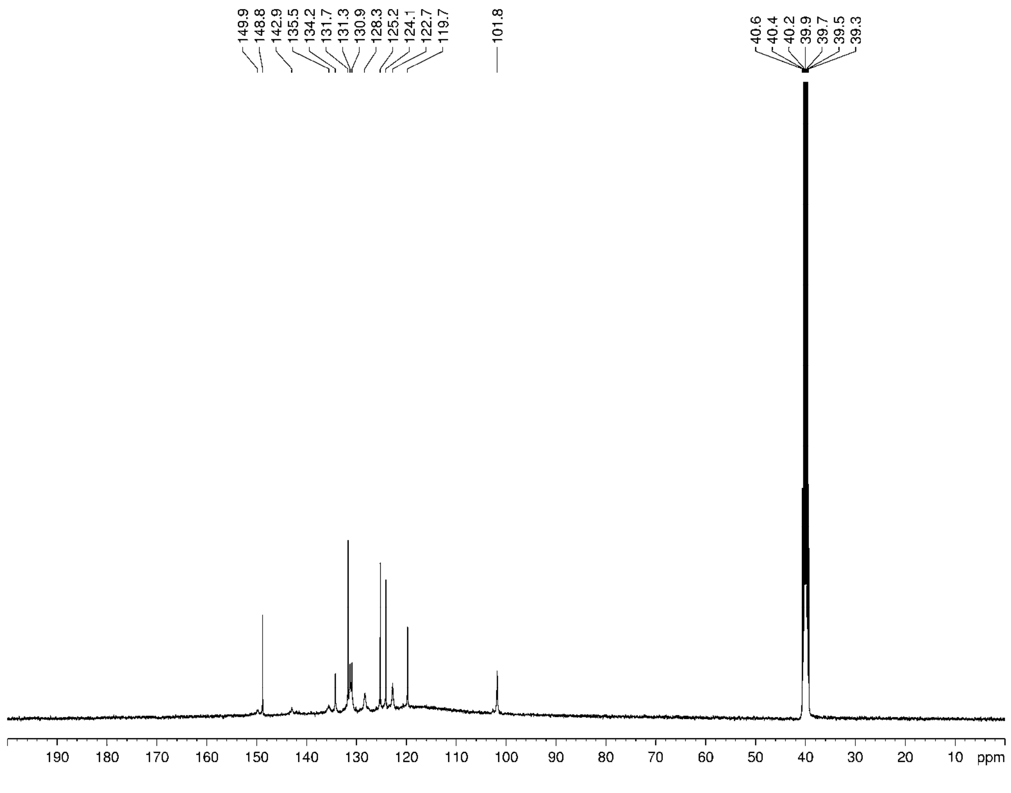
149.9
148.8
142.9
135.5
134.2
131.7
131.3
130.9
128.3
125.2
124.1
122.7
119.7
101.8
40.6
40.4
40.2
39.9
39.7
39.5
39.3
190 180 170 160 150 140 130 120 110 100 90 80 70 60 50 40 30 20 10 ppm

sery275B

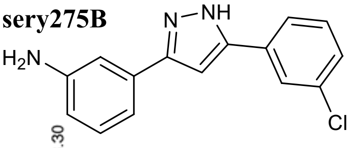

N–NH
H2N
Cl

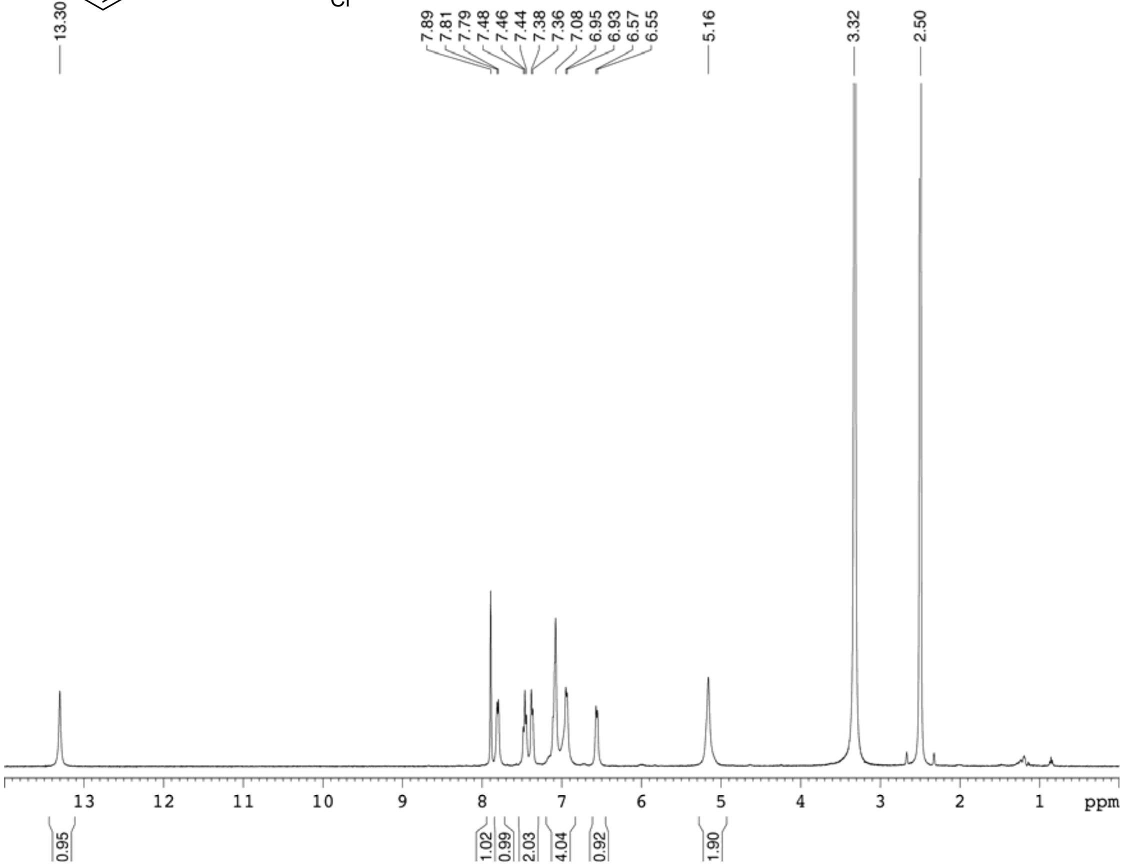

13.30
7.89 7.81 7.79 7.48 7.46 7.44 7.38 7.36 7.08 6.95 6.93 6.57 6.55
5.16
3.32
2.50
13 12 11 10 9 8 7 6 5 4 3 2 1 ppm
0.95
1.02 0.99 2.03 4.04 0.92
1.90

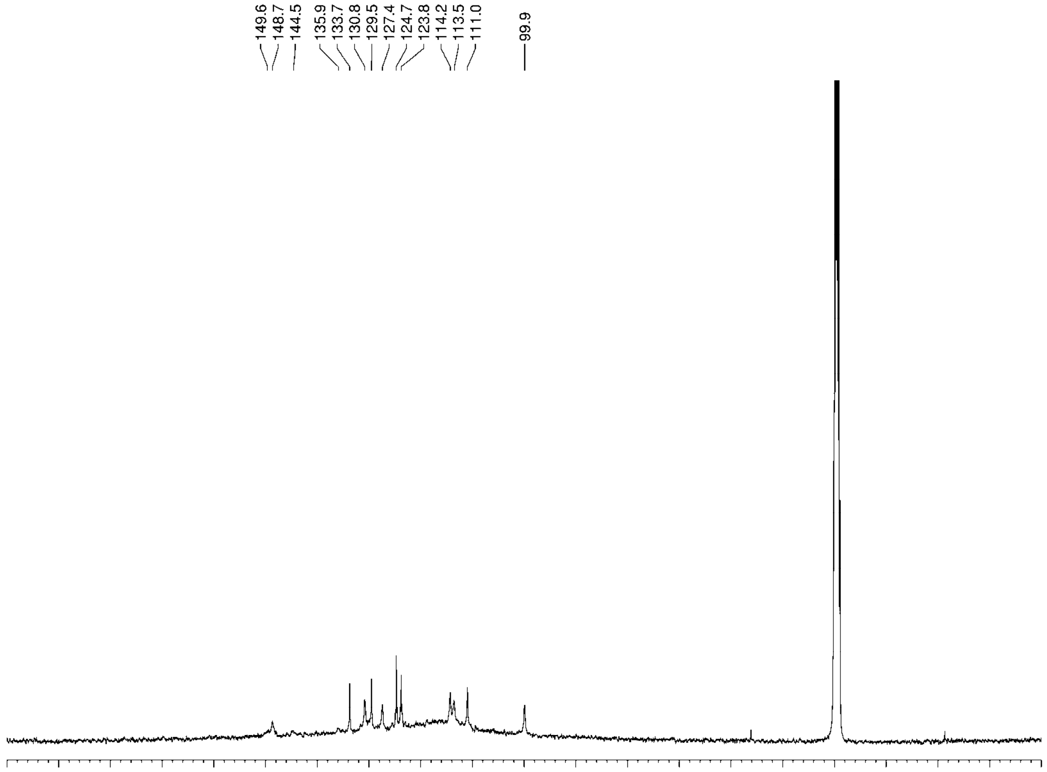

149.6 148.7 144.5 135.9 133.7 130.8 129.5 127.4 124.7 123.8 114.2 113.5 111.0
99.9
190 180 170 160 150 140 130 120 110 100 90 80 70 60 50 40 30 20 10 ppm

sery278A

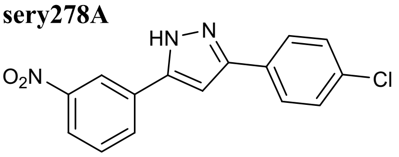
HN
N
O_2N
Cl

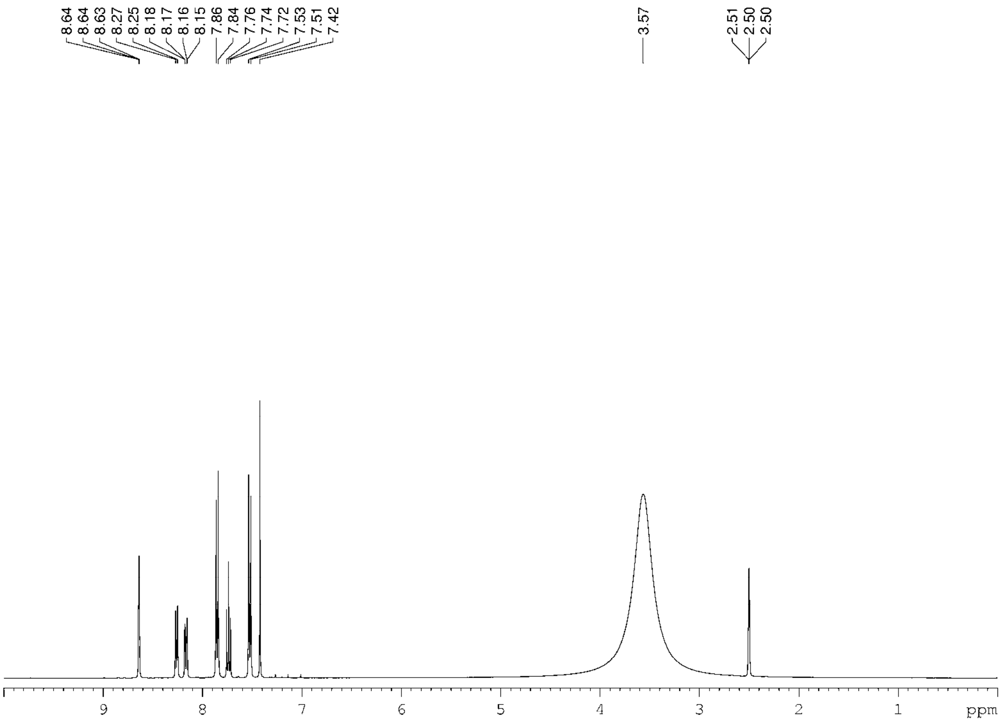
8.64
8.64
8.63
8.27
8.25
8.18
8.17
8.16
8.15
7.86
7.84
7.76
7.74
7.72
7.53
7.51
7.42
3.57
2.51
2.50
2.50
9
8
7
6
5
4
3
2
1
ppm

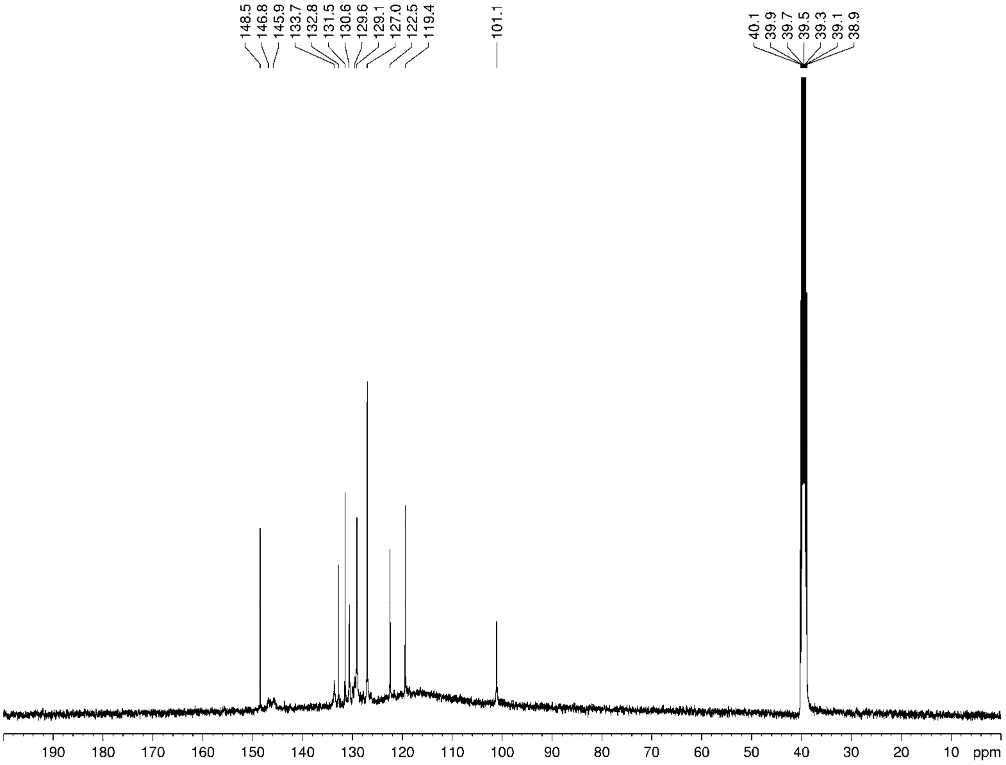
148.5
146.8
145.9
133.7
132.8
131.5
130.6
129.6
129.1
127.0
122.5
119.4
101.1
40.1
39.9
39.7
39.5
39.3
39.1
38.9
190
180
170
160
150
140
130
120
110
100
90
80
70
60
50
40
30
20
10
ppm

sery279B

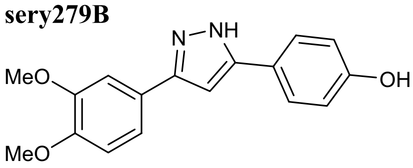
N–NH
MeO
MeO
OH

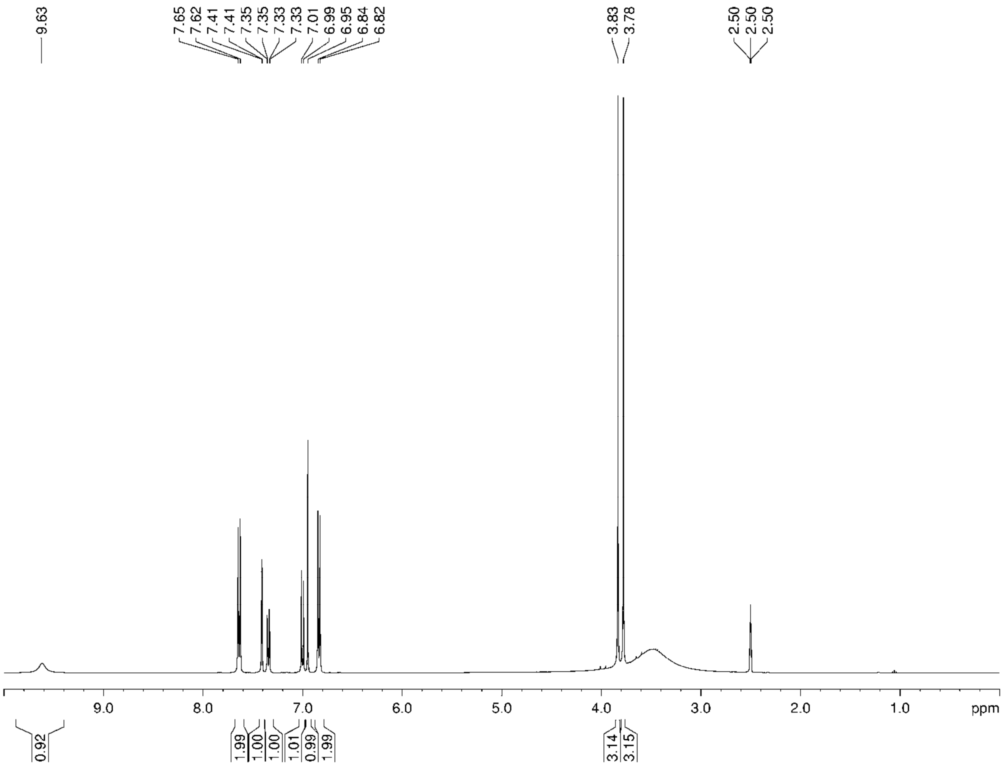
9.63
7.65 7.62 7.41 7.41 7.35 7.35 7.33 7.33 7.01 6.99 6.95 6.84 6.82
3.83 3.78
2.50 2.50 2.50
9.0 8.0 7.0 6.0 5.0 4.0 3.0 2.0 1.0 ppm
0.92
1.99 1.00 1.00 1.01 0.99 1.99
3.14 3.15

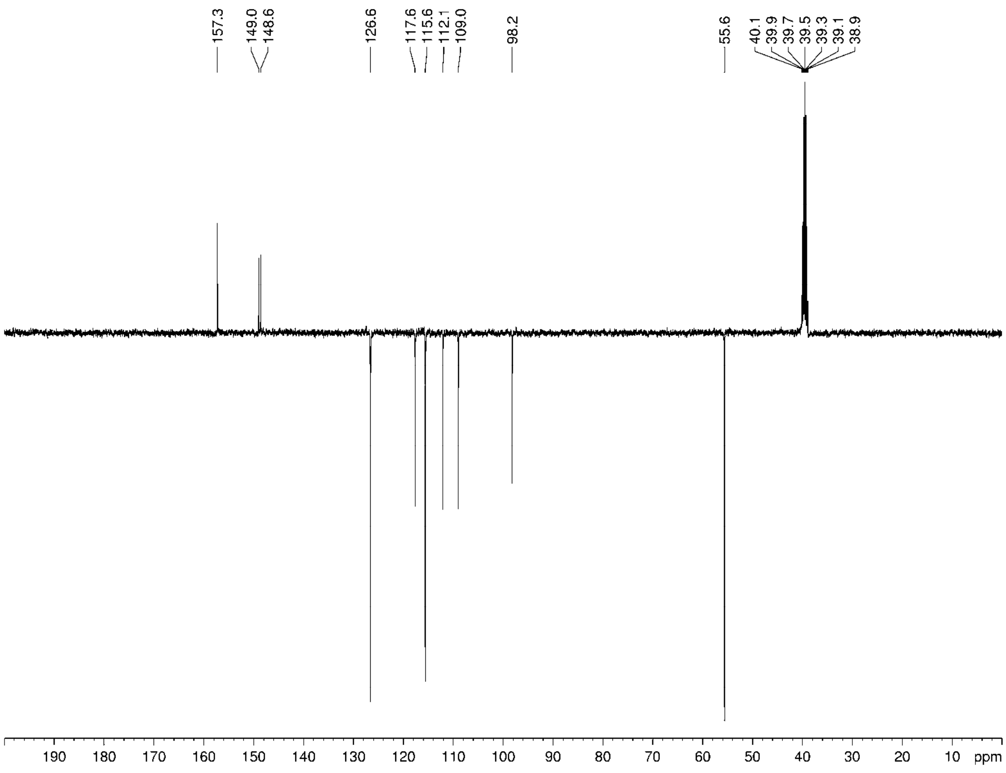
157.3
149.0 148.6
126.6
117.6 115.6 112.1 109.0
98.2
55.6
40.1 39.9 39.7 39.5 39.3 39.1 38.9
190 180 170 160 150 140 130 120 110 100 90 80 70 60 50 40 30 20 10 ppm

sery280A

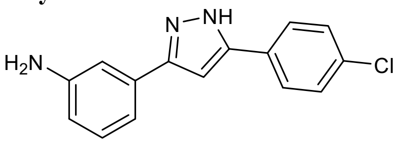

N
NH
H_2N
Cl

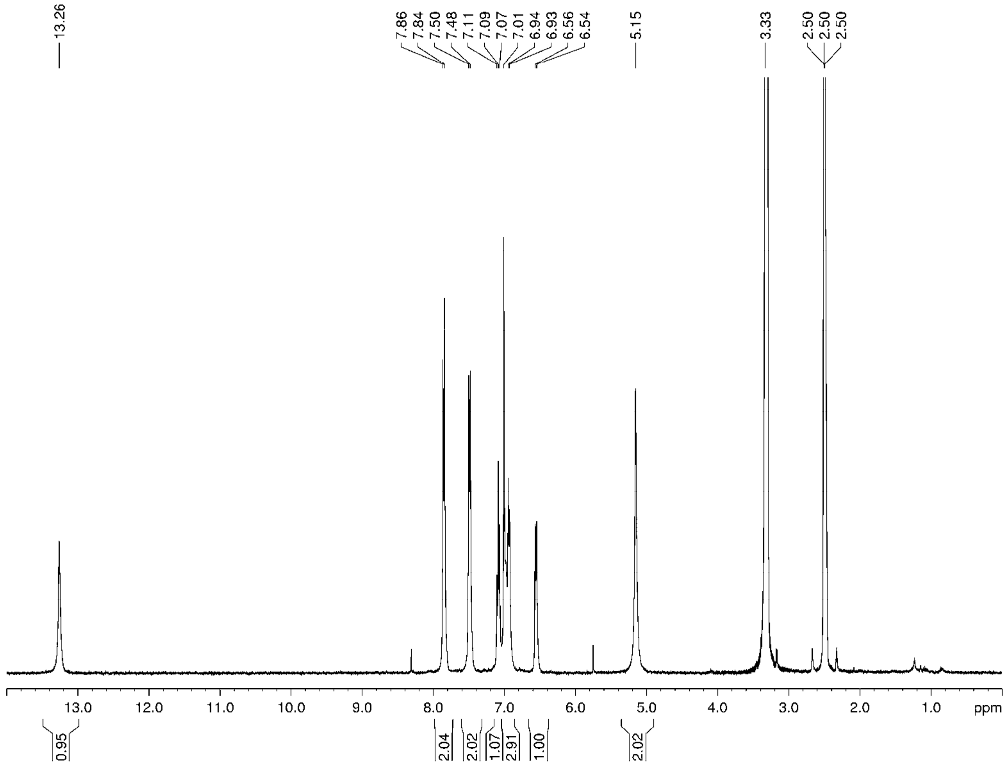

13.26
7.86
7.84
7.50
7.48
7.11
7.09
7.07
7.01
6.94
6.93
6.56
6.54
5.15
3.33
2.50
2.50
2.50
13.0
12.0
11.0
10.0
9.0
8.0
7.0
6.0
5.0
4.0
3.0
2.0
1.0
ppm
0.95
2.04
2.02
1.07
2.91
1.00
2.02

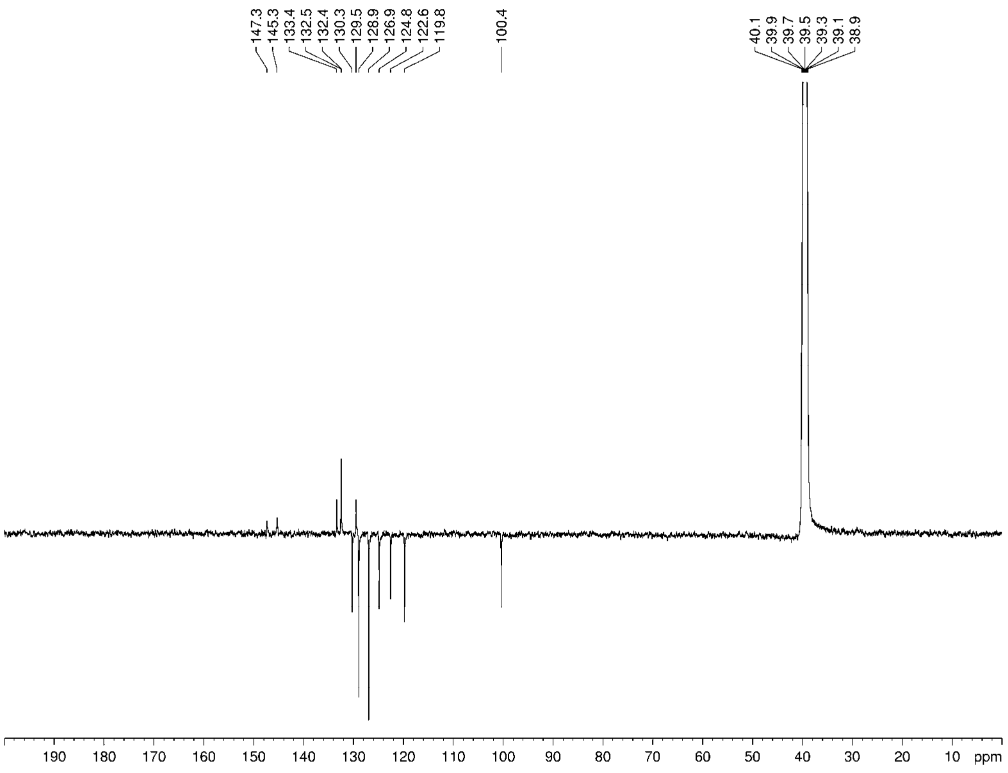

147.3
145.3
133.4
132.5
132.4
130.3
129.5
128.9
126.9
124.8
122.6
119.8
100.4
40.1
39.9
39.7
39.5
39.3
39.1
38.9
190
180
170
160
150
140
130
120
110
100
90
80
70
60
50
40
30
20
10
ppm

sery280B

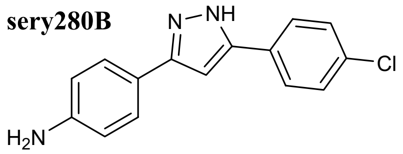
N–NH
Cl
H2N

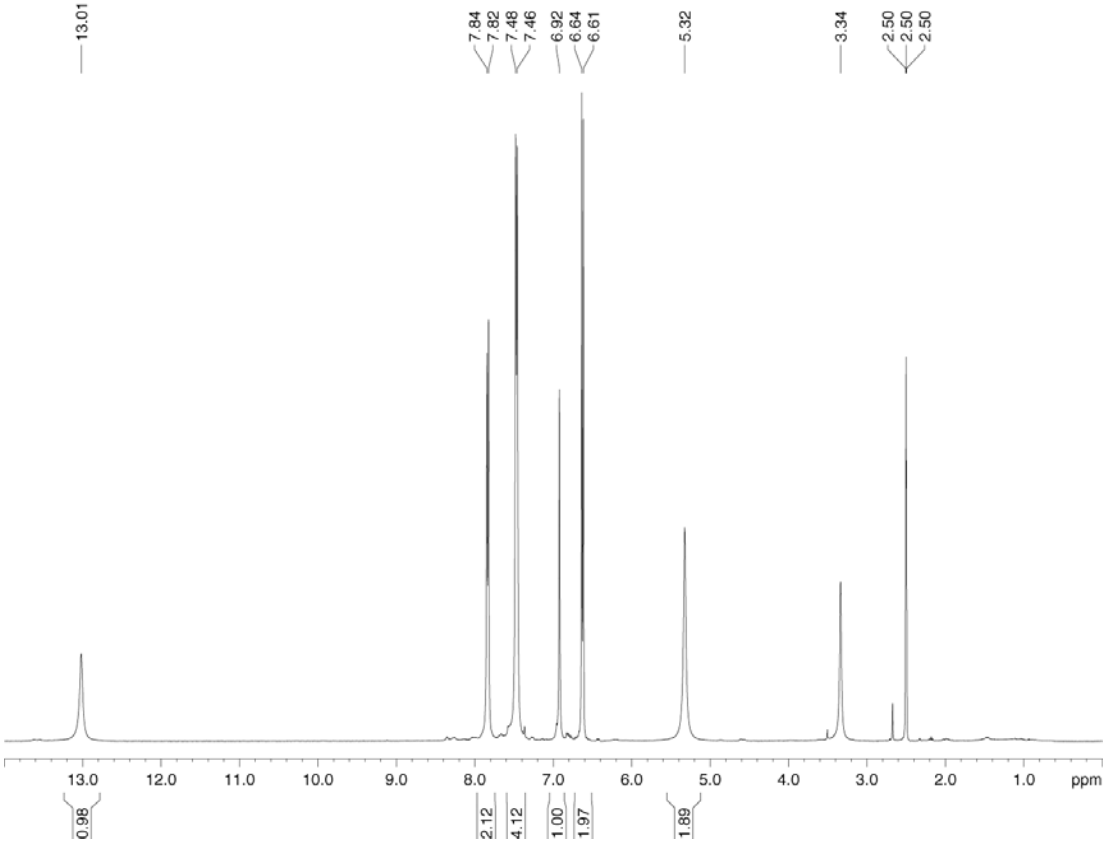
13.01
7.84
7.82
7.48
7.46
6.92
6.64
6.61
5.32
3.34
2.50
2.50
2.50
13.0 12.0 11.0 10.0 9.0 8.0 7.0 6.0 5.0 4.0 3.0 2.0 1.0 ppm
0.98
2.12
4.12
1.00
1.97
1.89

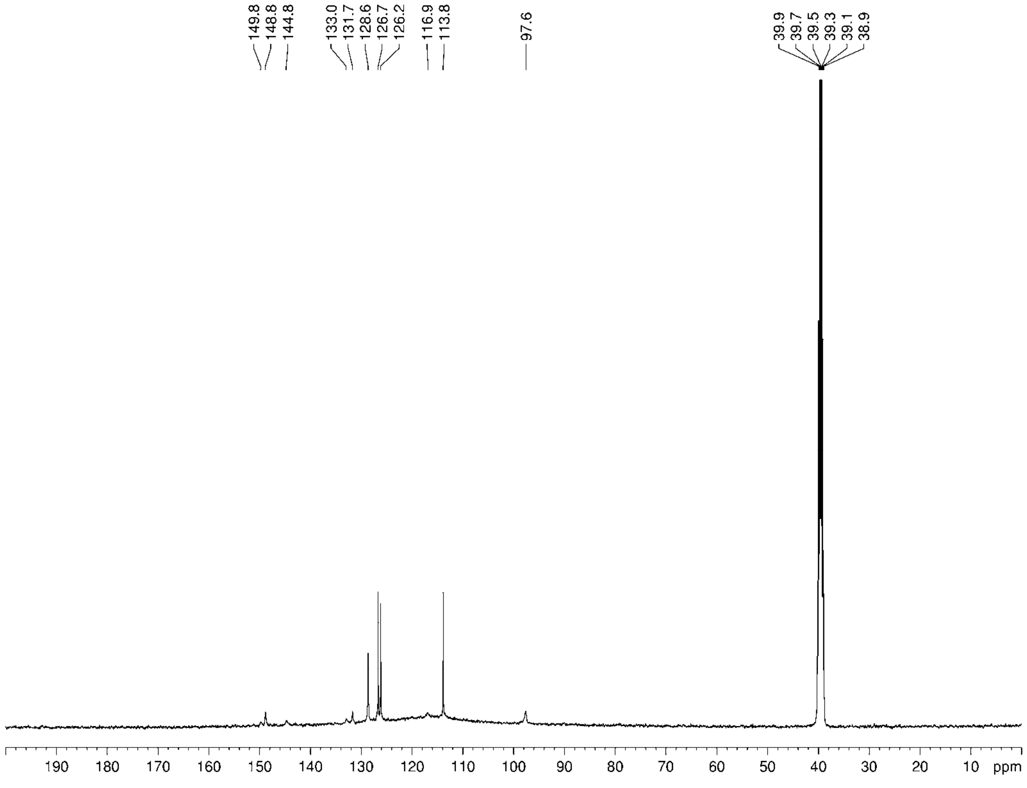
149.8
148.8
144.8
133.0
131.7
128.6
126.7
126.2
116.9
113.8
97.6
39.9
39.7
39.5
39.3
39.1
38.9
190 180 170 160 150 140 130 120 110 100 90 80 70 60 50 40 30 20 10 ppm

sery287A

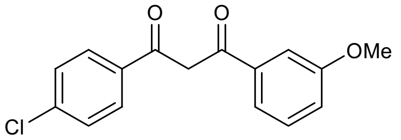

O
O
OMe
Cl

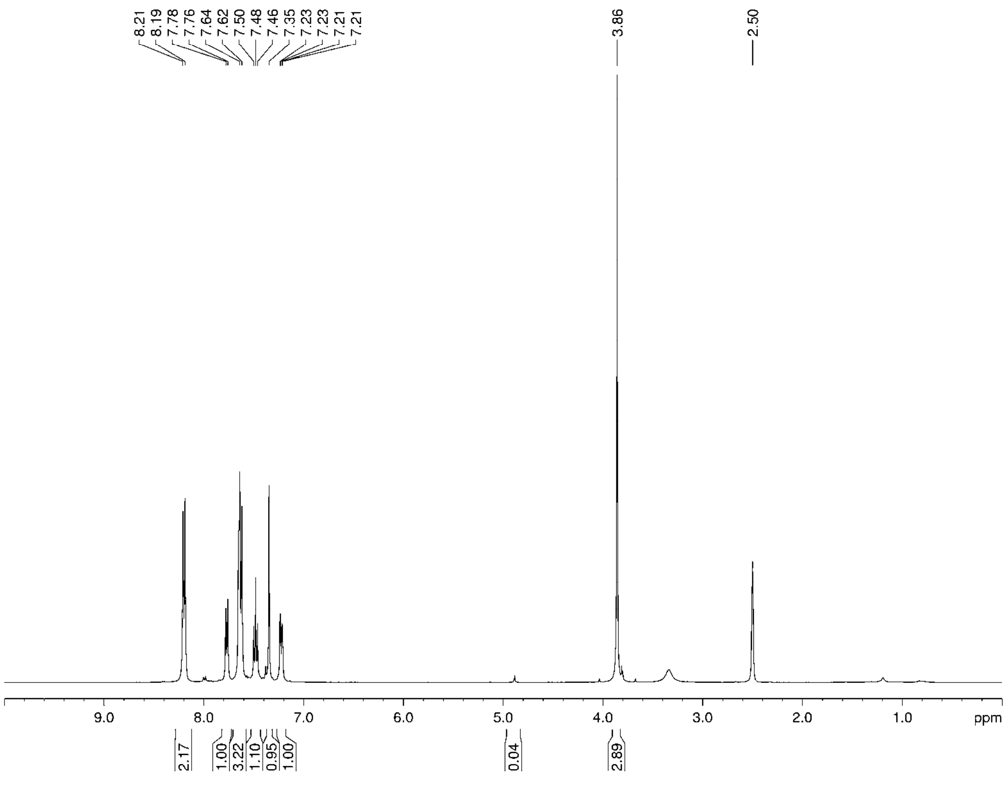

8.21
8.19
7.78
7.76
7.64
7.62
7.50
7.48
7.46
7.35
7.23
7.23
7.21
7.21
3.86
2.50
9.0
8.0
7.0
6.0
5.0
4.0
3.0
2.0
1.0
ppm
2.17
1.00
3.22
1.10
0.95
1.00
0.04
2.89

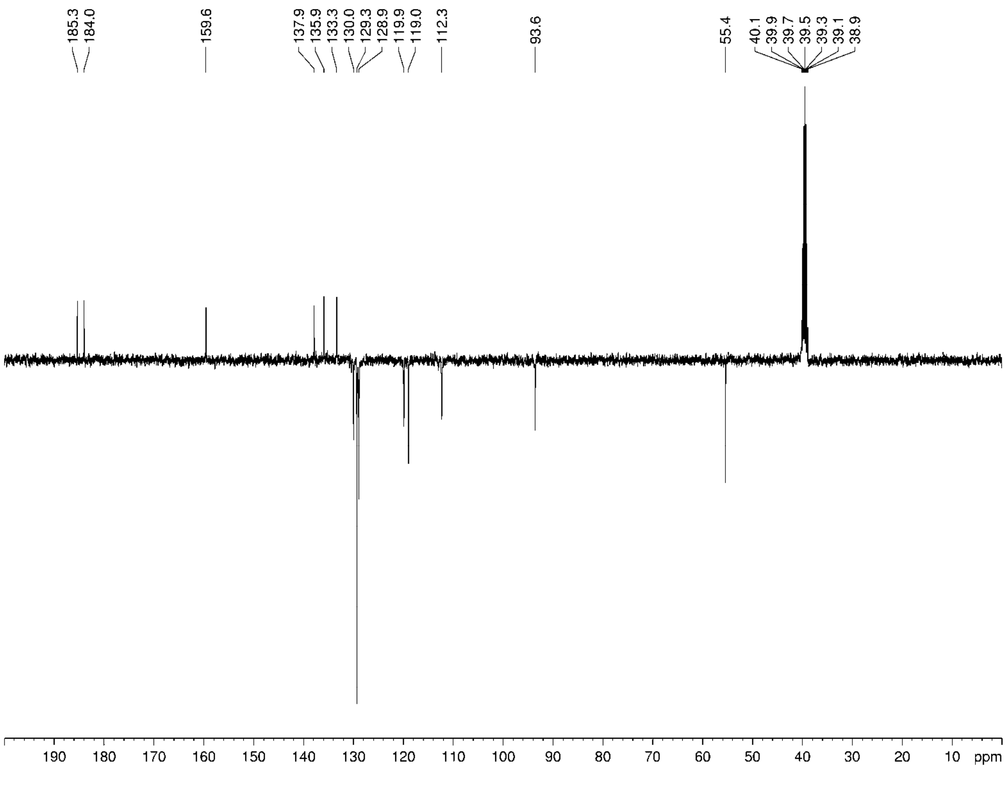

185.3
184.0
159.6
137.9
135.9
133.3
130.0
129.3
128.9
119.9
119.0
112.3
93.6
55.4
40.1
39.9
39.7
39.5
39.3
39.1
38.9
190
180
170
160
150
140
130
120
110
100
90
80
70
60
50
40
30
20
10
ppm

sery287B

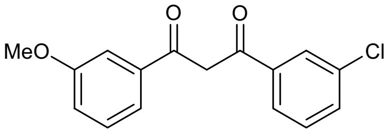

MeO
O
O
Cl

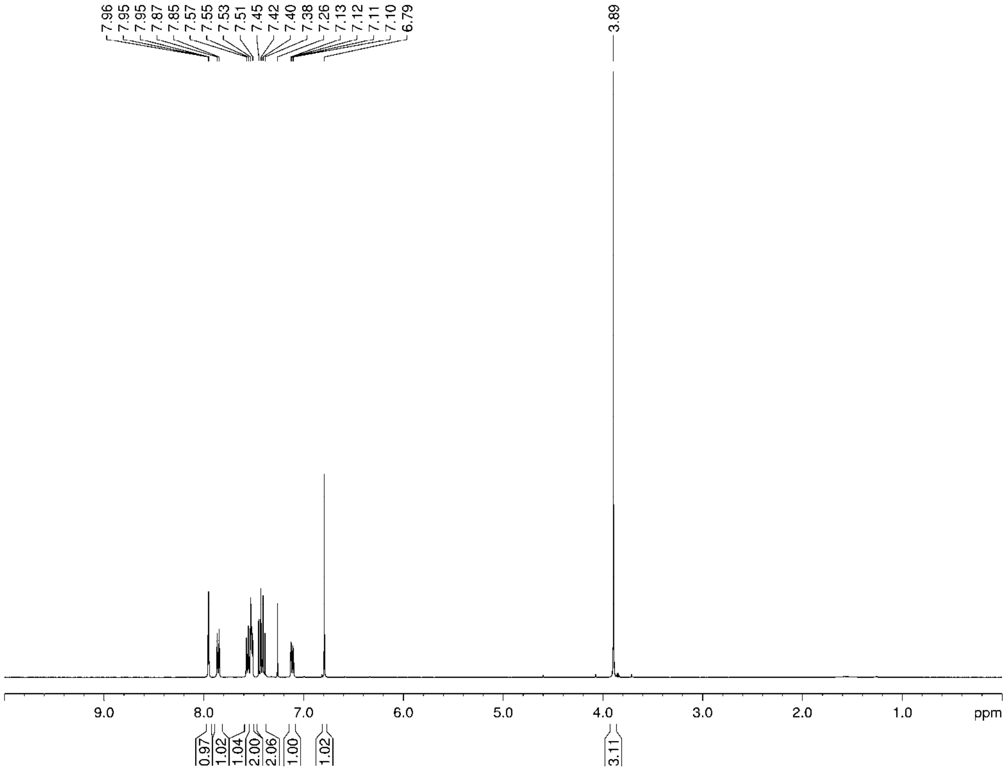

7.96
7.95
7.95
7.87
7.85
7.57
7.55
7.53
7.51
7.45
7.42
7.40
7.38
7.26
7.13
7.12
7.11
7.10
6.79
3.89
9.0
8.0
7.0
6.0
5.0
4.0
3.0
2.0
1.0
ppm
0.97
1.02
1.04
2.00
2.06
1.00
1.02
3.11

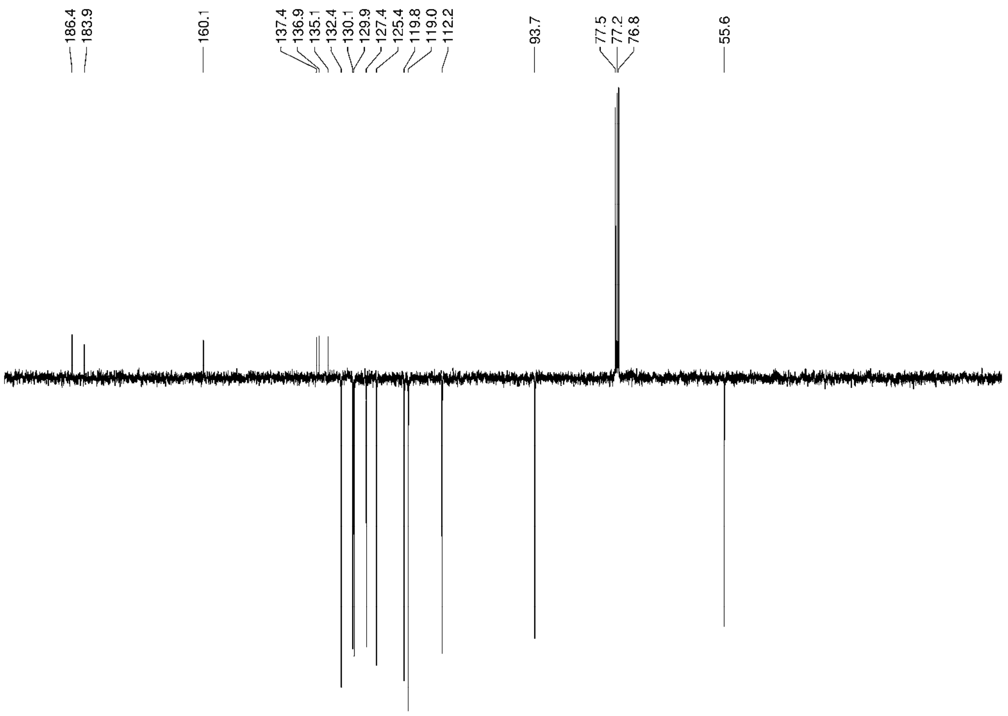

186.4
183.9
160.1
137.4
136.9
135.1
132.4
130.1
129.9
127.4
125.4
119.8
119.0
112.2
93.7
77.5
77.2
76.8
55.6
190
180
170
160
150
140
130
120
110
100
90
80
70
60
50
40
30
20
10
ppm

sery289

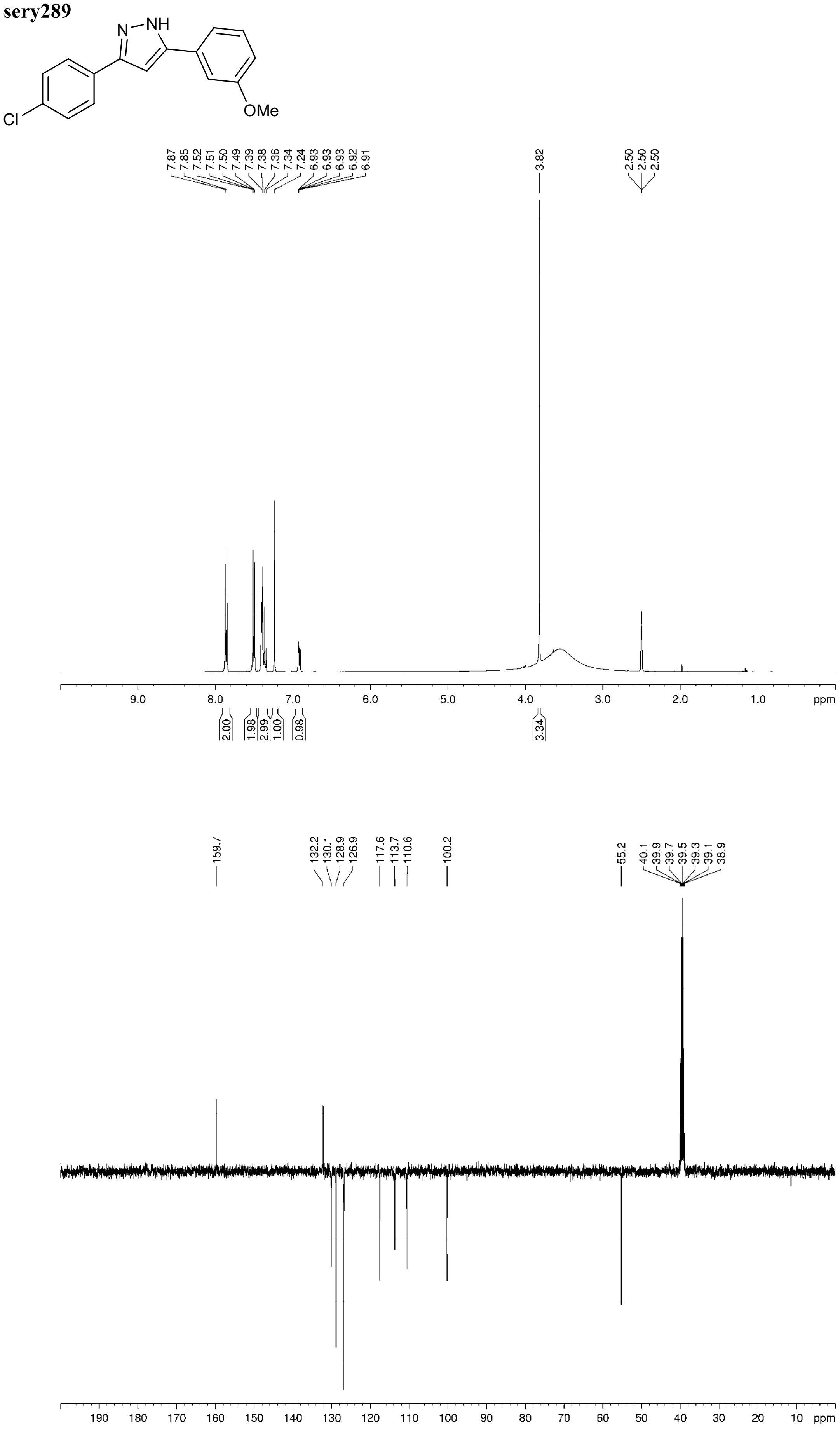
N
NH
Cl
OMe
7.87
7.85
7.52
7.51
7.50
7.49
7.39
7.38
7.36
7.34
7.24
6.93
6.93
6.93
6.92
6.91
3.82
2.50
2.50
2.50
9.0
8.0
7.0
6.0
5.0
4.0
3.0
2.0
1.0
ppm
2.00
1.98
2.99
1.00
0.98
3.34
159.7
132.2
130.1
128.9
126.9
117.6
113.7
110.6
100.2
55.2
40.1
39.9
39.7
39.5
39.3
39.1
38.9
190
180
170
160
150
140
130
120
110
100
90
80
70
60
50
40
30
20
10
ppm

sery292A

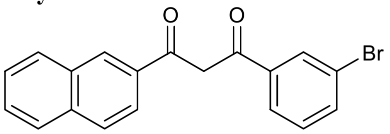

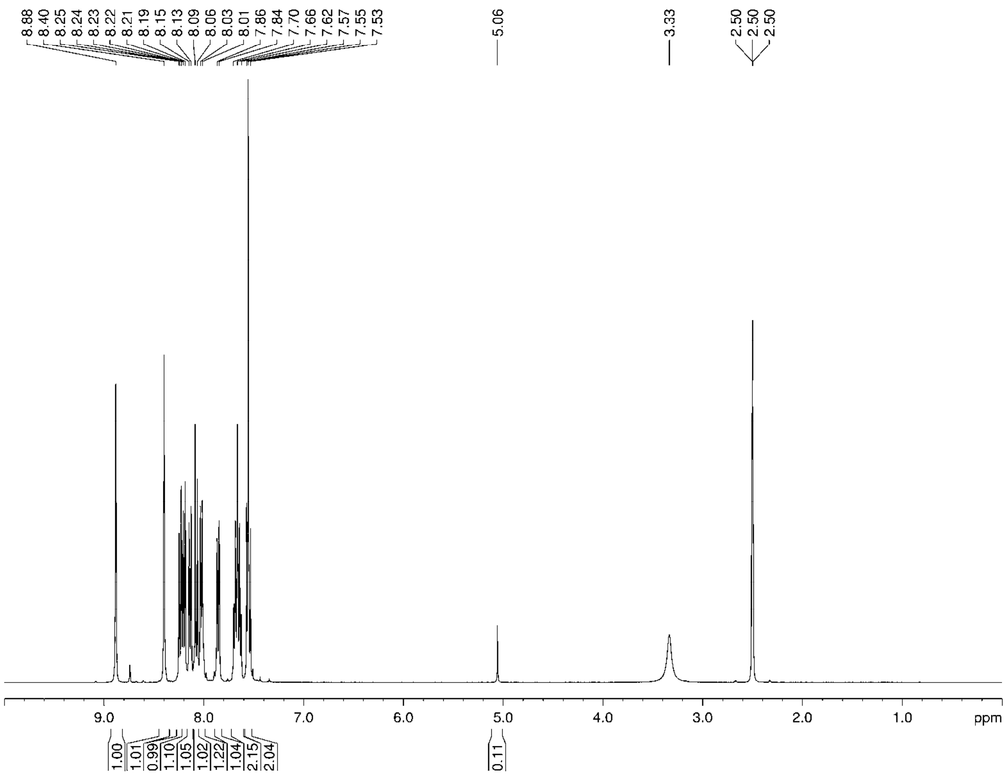
8.88
8.40
8.25
8.24
8.23
8.22
8.21
8.19
8.15
8.13
8.09
8.06
8.03
8.01
7.86
7.84
7.70
7.66
7.62
7.57
7.55
7.53
5.06
3.33
2.50
2.50
2.50
9.0
8.0
7.0
6.0
5.0
4.0
3.0
2.0
1.0
ppm
1.00
1.01
0.99
1.10
1.05
1.02
1.22
1.04
2.15
2.04
0.11

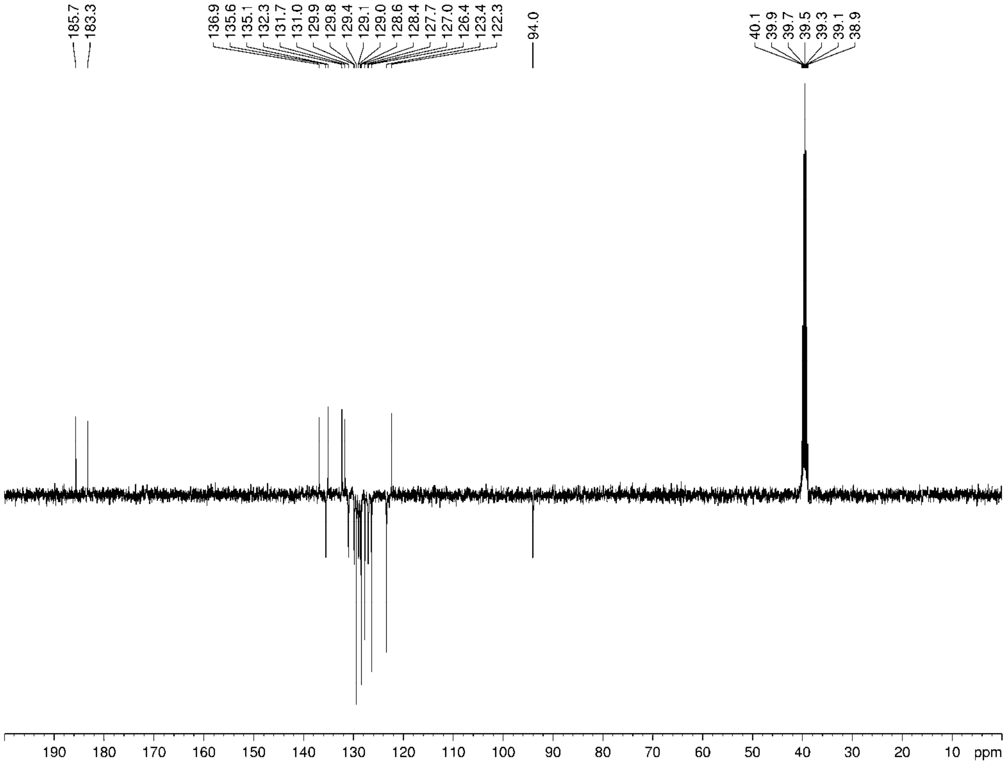
185.7
183.3
136.9
135.6
135.1
132.3
131.7
131.0
129.9
129.8
129.4
129.1
129.0
128.6
128.4
127.7
127.0
126.4
123.4
122.3
94.0
40.1
39.9
39.7
39.5
39.3
39.1
38.9
190
180
170
160
150
140
130
120
110
100
90
80
70
60
50
40
30
20
10
ppm

sery292B

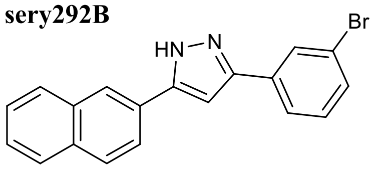
HN–N
Br

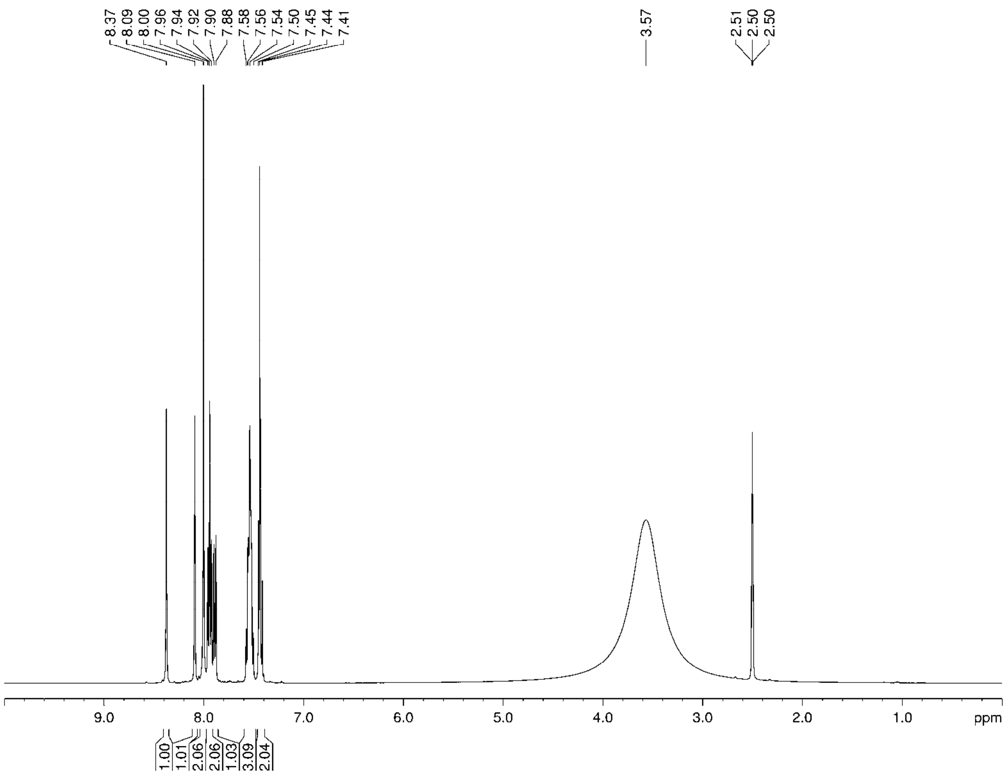
8.37
8.09
8.00
7.96
7.94
7.92
7.90
7.88
7.58
7.56
7.54
7.50
7.45
7.44
7.41
3.57
2.51
2.50
2.50
9.0
8.0
7.0
6.0
5.0
4.0
3.0
2.0
1.0
ppm
1.00
1.01
2.06
2.06
1.03
3.09
2.04

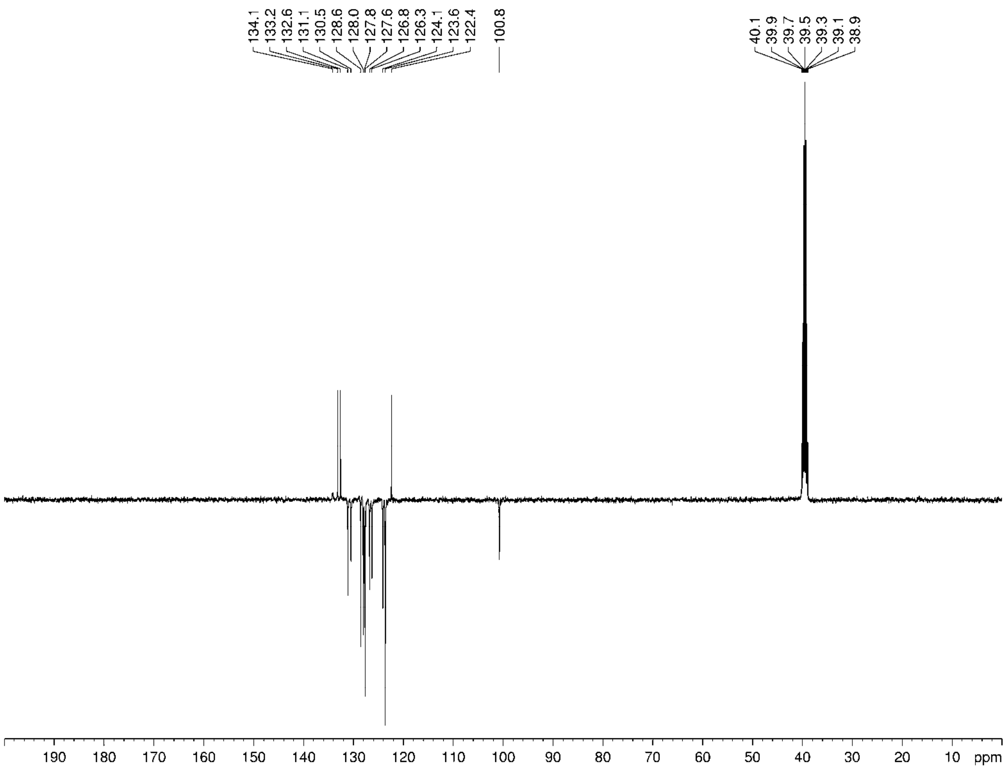
134.1
133.2
132.6
131.1
130.5
128.6
128.0
127.8
127.6
126.8
126.3
124.1
123.6
122.4
100.8
40.1
39.9
39.7
39.5
39.3
39.1
38.9
190
180
170
160
150
140
130
120
110
100
90
80
70
60
50
40
30
20
10
ppm

sery294A

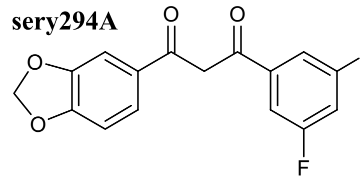
O
O
O
O
F
F

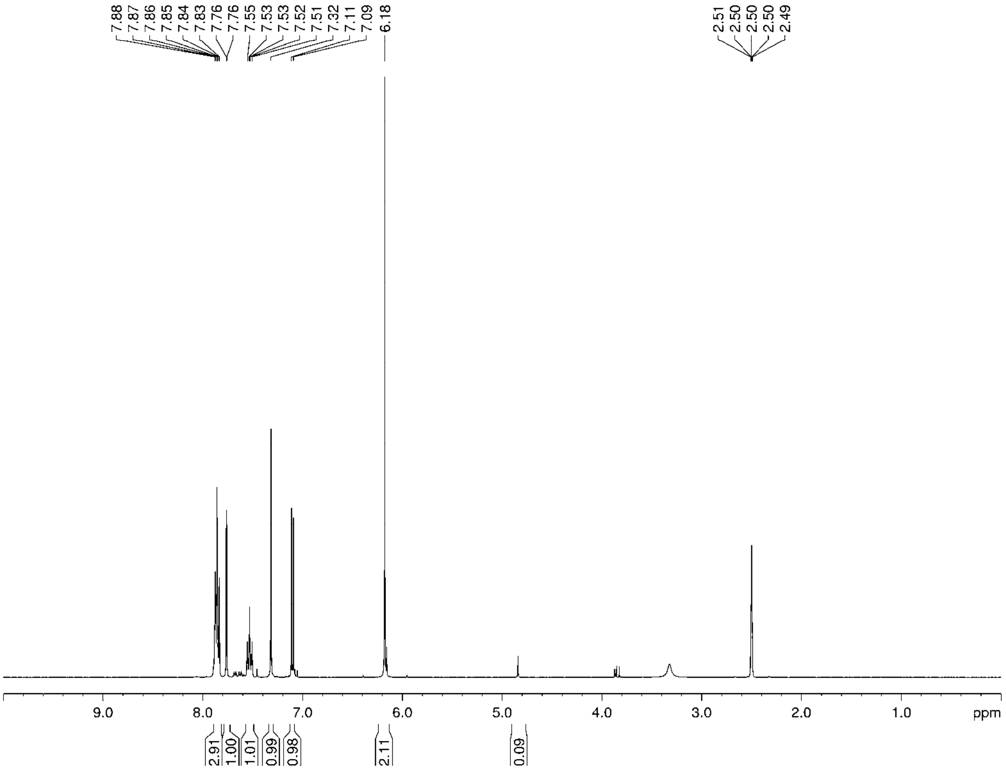
7.88
7.87
7.86
7.85
7.84
7.83
7.76
7.76
7.55
7.53
7.53
7.52
7.51
7.32
7.11
7.09
6.18
2.51
2.50
2.50
2.50
2.49
9.0
8.0
7.0
6.0
5.0
4.0
3.0
2.0
1.0
ppm
2.91
1.00
1.01
0.99
0.98
2.11
0.09

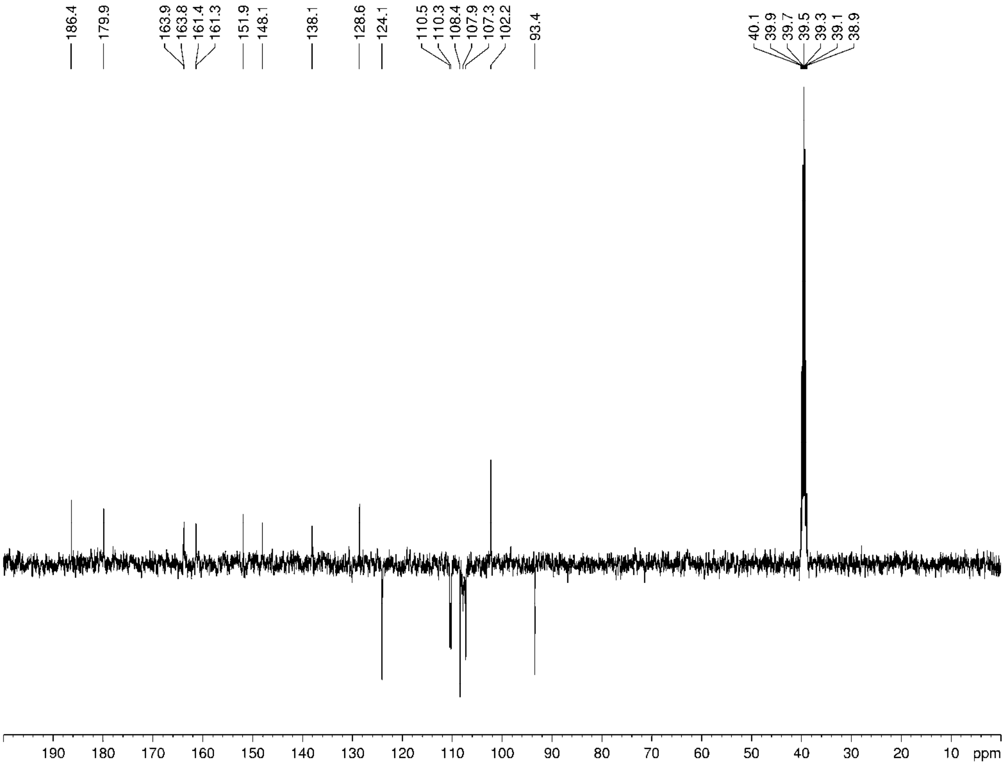
186.4
179.9
163.9
163.8
161.4
161.3
151.9
148.1
138.1
128.6
124.1
110.5
110.3
108.4
107.9
107.3
102.2
93.4
40.1
39.9
39.7
39.5
39.3
39.1
38.9
190
180
170
160
150
140
130
120
110
100
90
80
70
60
50
40
30
20
10
ppm

sery294B

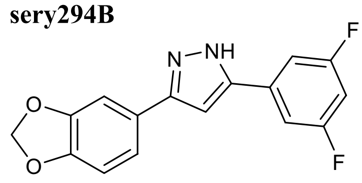
N
NH
O
O
F
F

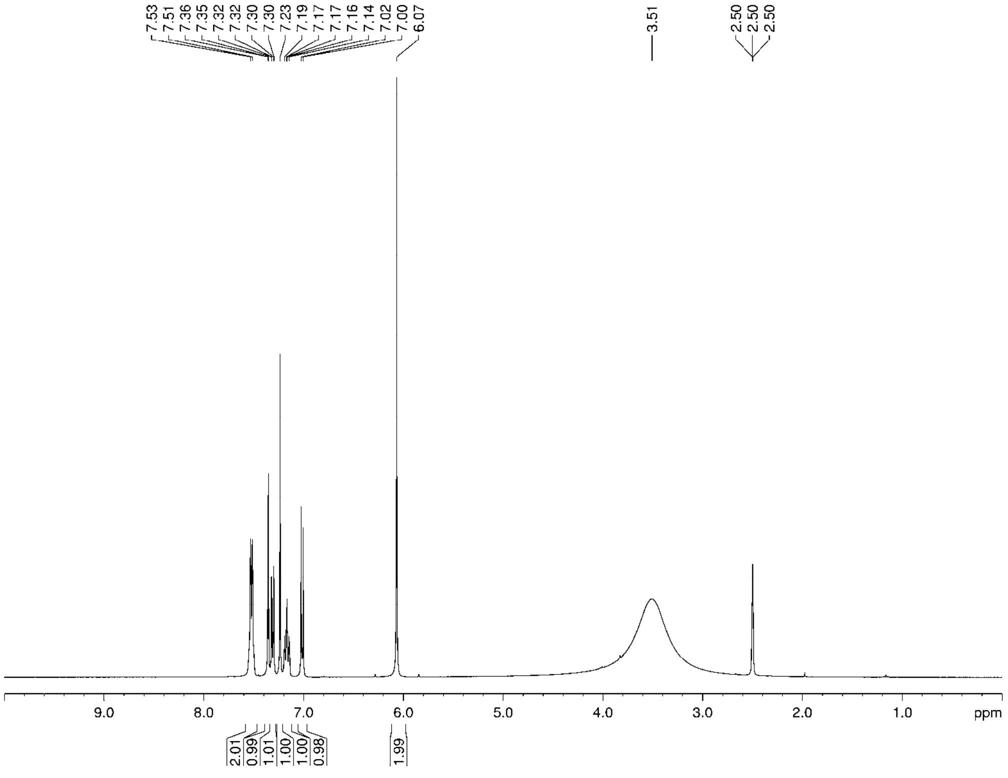
7.53
7.51
7.36
7.35
7.32
7.32
7.30
7.30
7.23
7.19
7.17
7.17
7.16
7.14
7.02
7.00
6.07
3.51
2.50
2.50
2.50
9.0
8.0
7.0
6.0
5.0
4.0
3.0
2.0
1.0
ppm
2.01
0.99
1.01
1.00
1.00
0.98
1.99

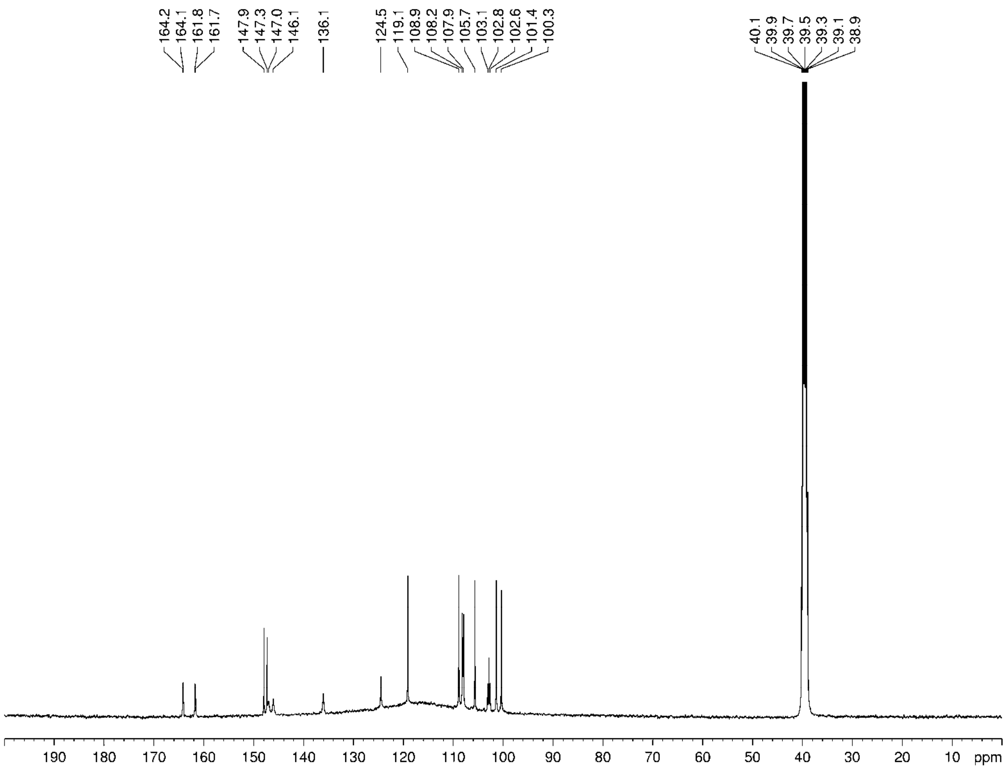
164.2
164.1
161.8
161.7
147.9
147.3
147.0
146.1
136.1
124.5
119.1
108.9
108.2
107.9
105.7
103.1
102.8
102.6
101.4
100.3
40.1
39.9
39.7
39.5
39.3
39.1
38.9
190
180
170
160
150
140
130
120
110
100
90
80
70
60
50
40
30
20
10
ppm

sery297A

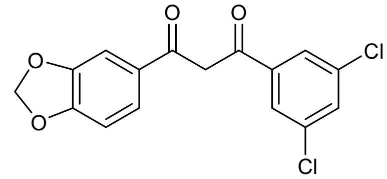

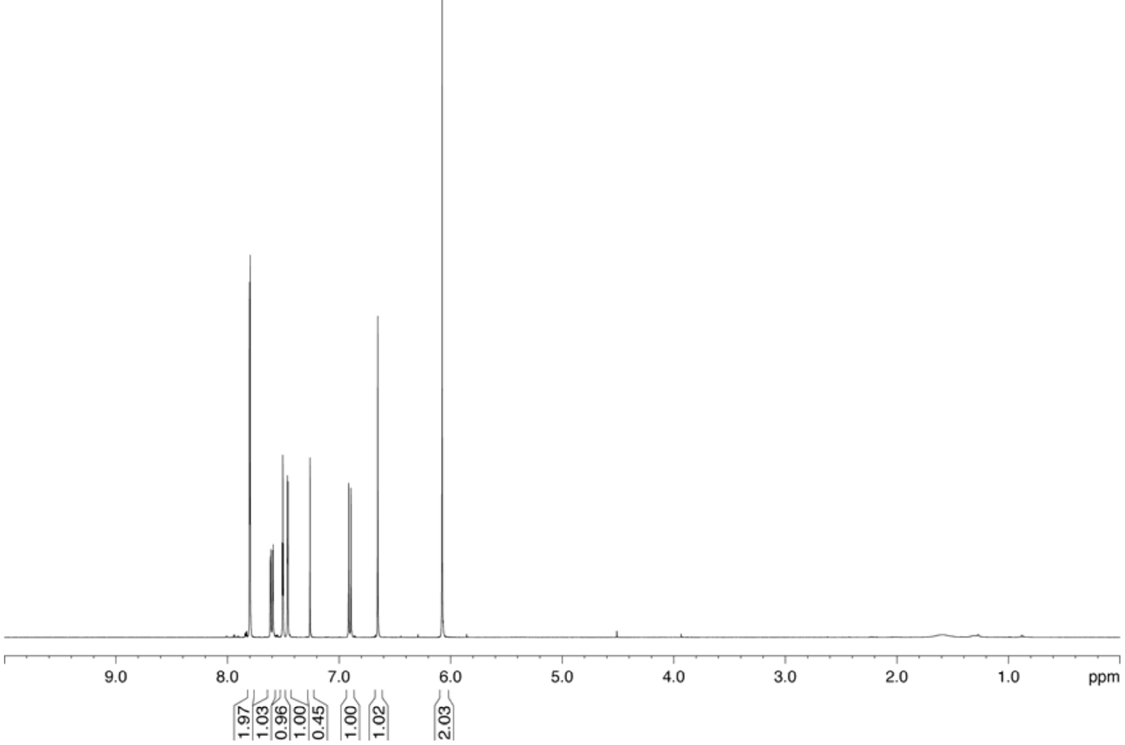
7.80
7.80
7.62
7.61
7.60
7.59
7.51
7.50
7.50
7.46
7.46
7.26
6.91
6.89
6.65
6.08
9.0
8.0
7.0
6.0
5.0
4.0
3.0
2.0
1.0
ppm
1.97
1.03
0.96
1.00
0.45
1.00
1.02
2.03

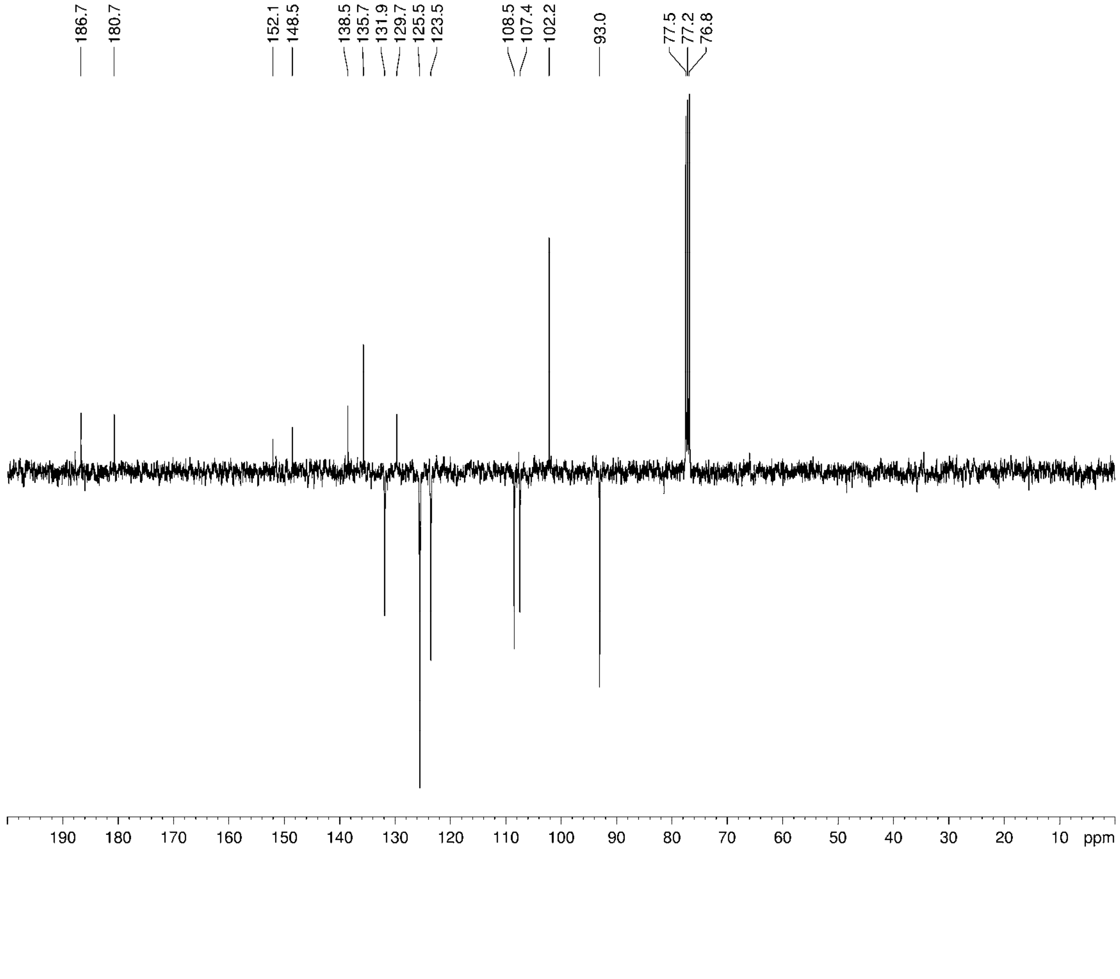
186.7
180.7
152.1
148.5
138.5
135.7
131.9
129.7
125.5
123.5
108.5
107.4
102.2
93.0
77.5
77.2
76.8
190
180
170
160
150
140
130
120
110
100
90
80
70
60
50
40
30
20
10
ppm

sery297B

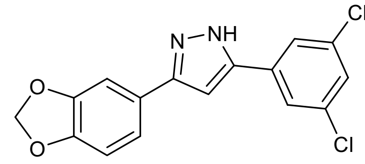
N–NH
O
O
Cl
Cl

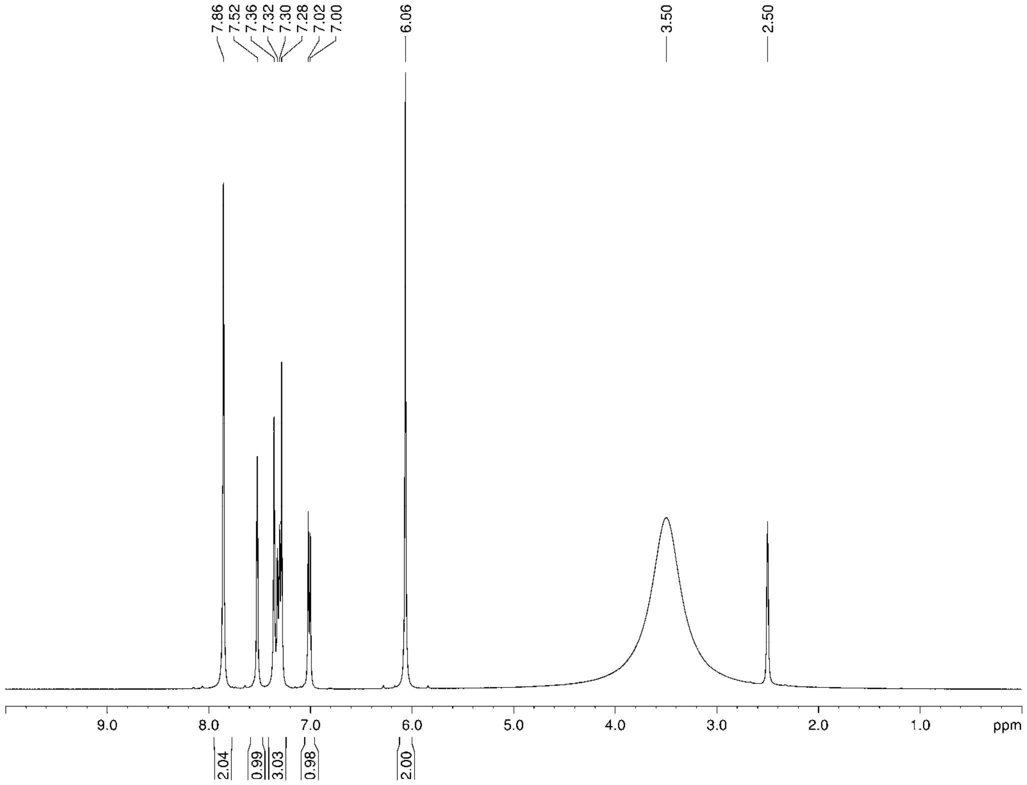
7.86
7.52
7.36
7.32
7.30
7.28
7.02
7.00
6.06
3.50
2.50
9.0 8.0 7.0 6.0 5.0 4.0 3.0 2.0 1.0 ppm
2.04
0.99
3.03
0.98
2.00

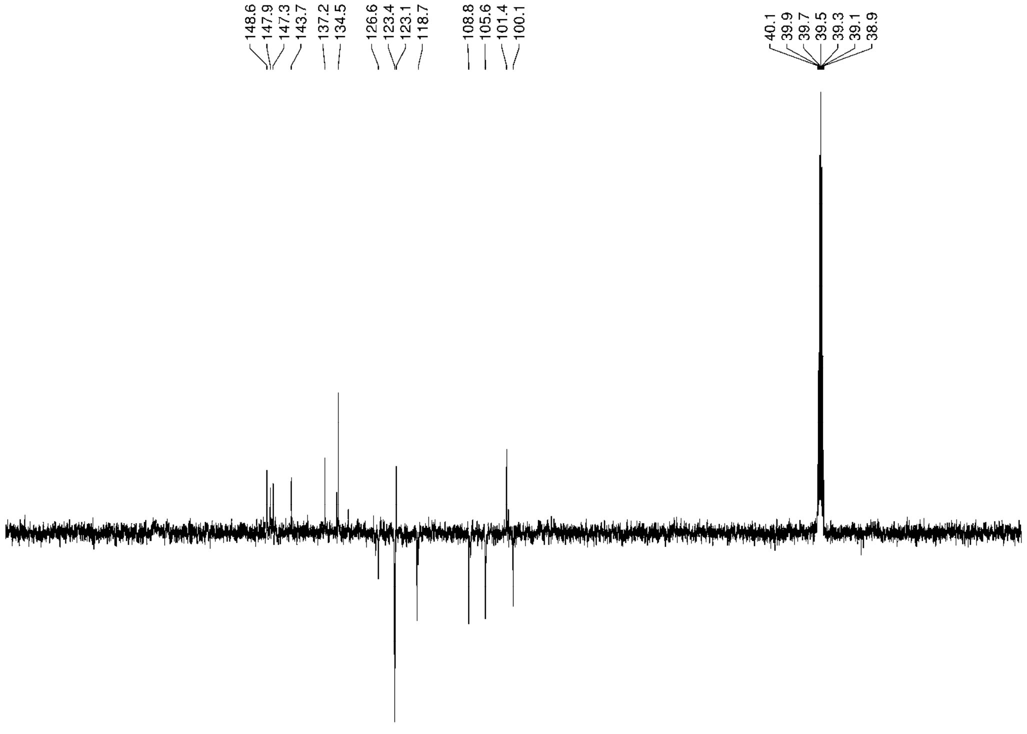
148.6
147.9
147.3
143.7
137.2
134.5
126.6
123.4
123.1
118.7
108.8
105.6
101.4
100.1
40.1
39.9
39.7
39.5
39.3
39.1
38.9
190 180 170 160 150 140 130 120 110 100 90 80 70 60 50 40 30 20 10 ppm

sery298A

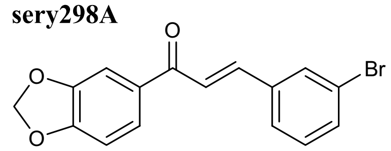
O
O
O
Br

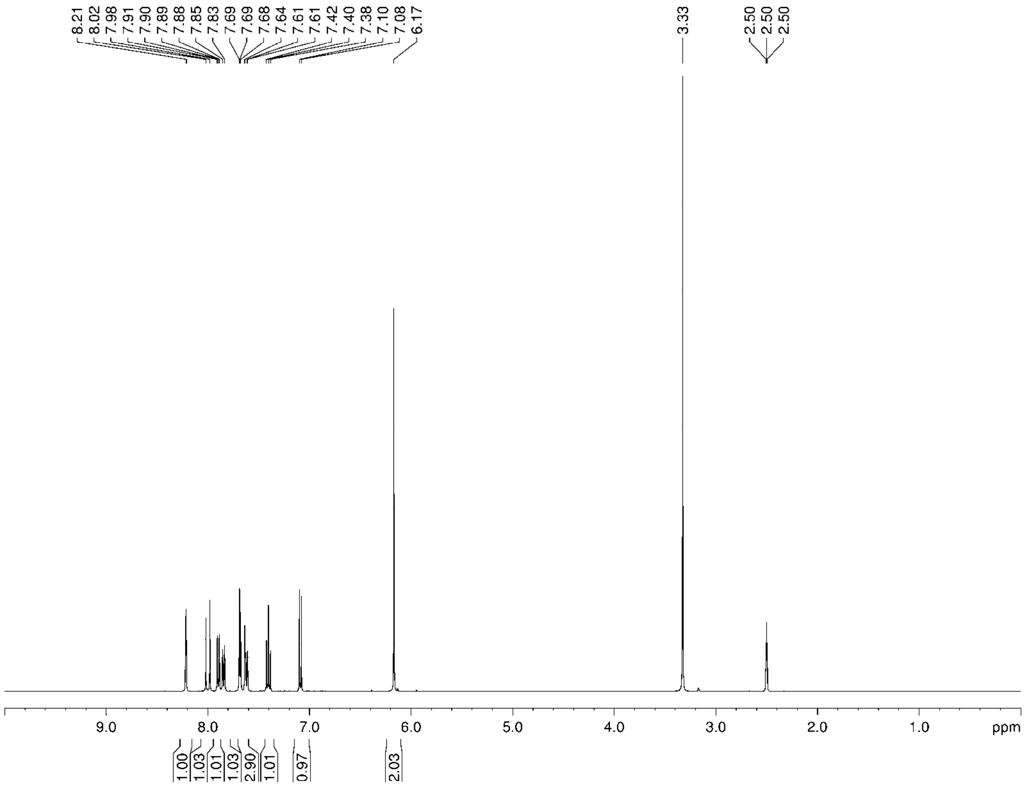
8.21
8.02
7.98
7.91
7.90
7.89
7.88
7.85
7.83
7.69
7.69
7.68
7.64
7.61
7.61
7.42
7.40
7.38
7.10
7.08
6.17
3.33
2.50
2.50
2.50
9.0
8.0
7.0
6.0
5.0
4.0
3.0
2.0
1.0
ppm
1.00
1.03
1.01
1.03
2.90
1.01
0.97
2.03

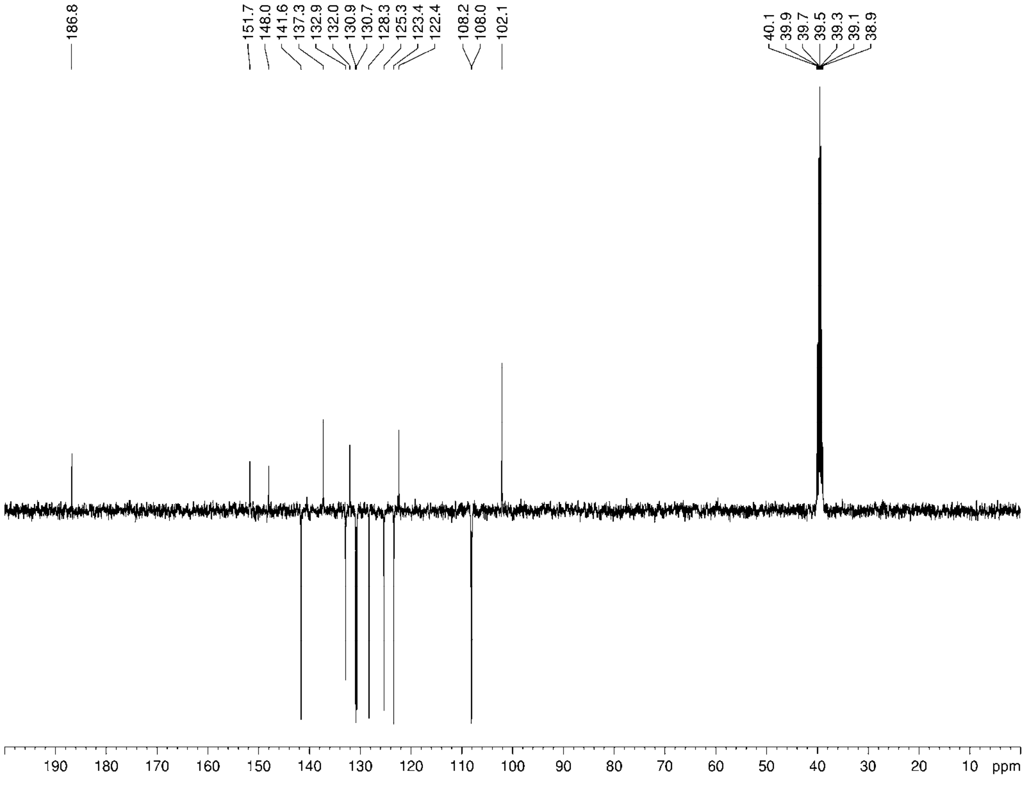
186.8
151.7
148.0
141.6
137.3
132.9
132.0
130.9
130.7
128.3
125.3
123.4
122.4
108.2
108.0
102.1
40.1
39.9
39.7
39.5
39.3
39.1
38.9
190
180
170
160
150
140
130
120
110
100
90
80
70
60
50
40
30
20
10
ppm

sery298B

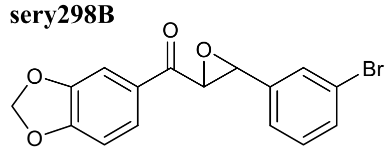
O
O
O
O
Br

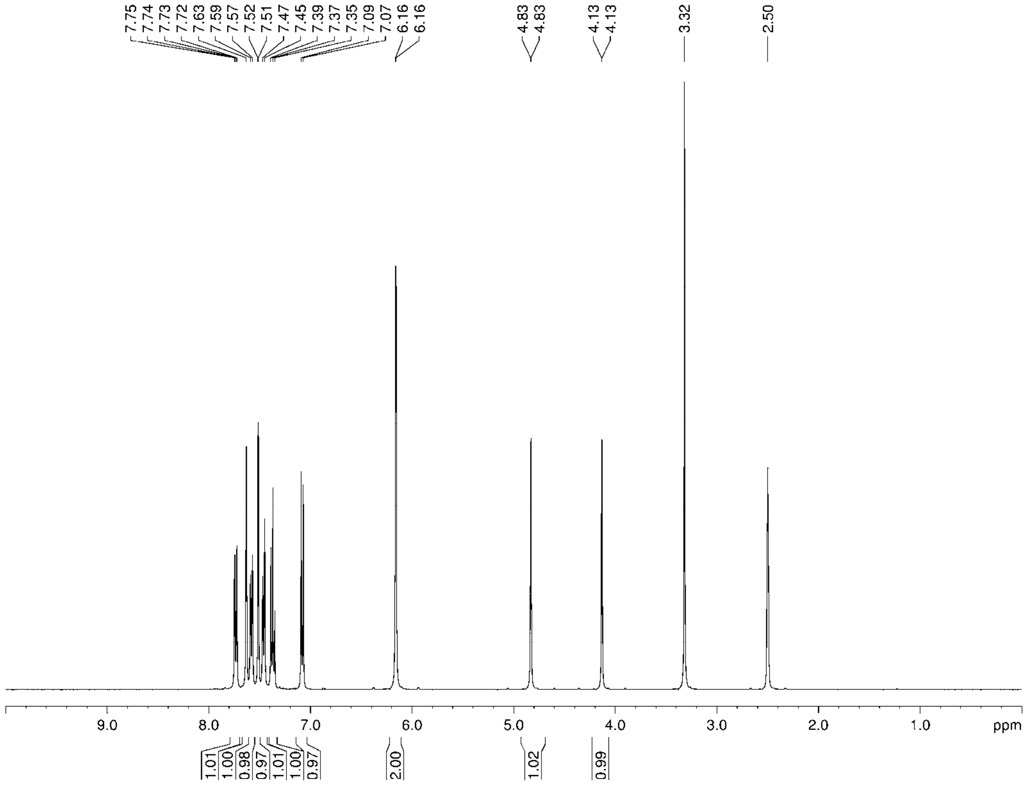
7.75
7.74
7.73
7.72
7.63
7.59
7.57
7.52
7.51
7.47
7.45
7.39
7.37
7.35
7.09
7.07
6.16
6.16
4.83
4.83
4.13
4.13
3.32
2.50
9.0
8.0
7.0
6.0
5.0
4.0
3.0
2.0
1.0
ppm
1.01
1.00
0.98
0.97
1.01
1.00
0.97
2.00
1.02
0.99

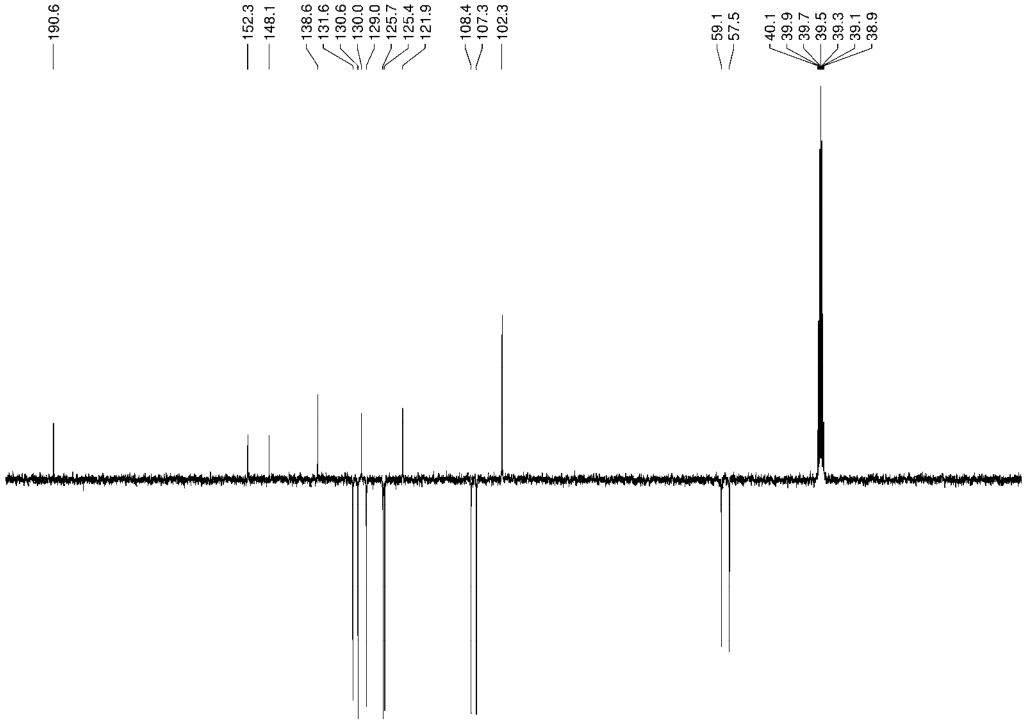
190.6
152.3
148.1
138.6
131.6
130.6
130.0
129.0
125.7
125.4
121.9
108.4
107.3
102.3
59.1
57.5
40.1
39.9
39.7
39.5
39.3
39.1
38.9
190
180
170
160
150
140
130
120
110
100
90
80
70
60
50
40
30
20
10
ppm

sery300A

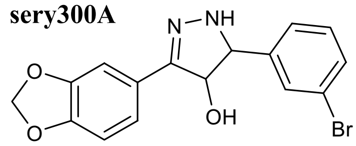
N–NH
O
O
OH
Br

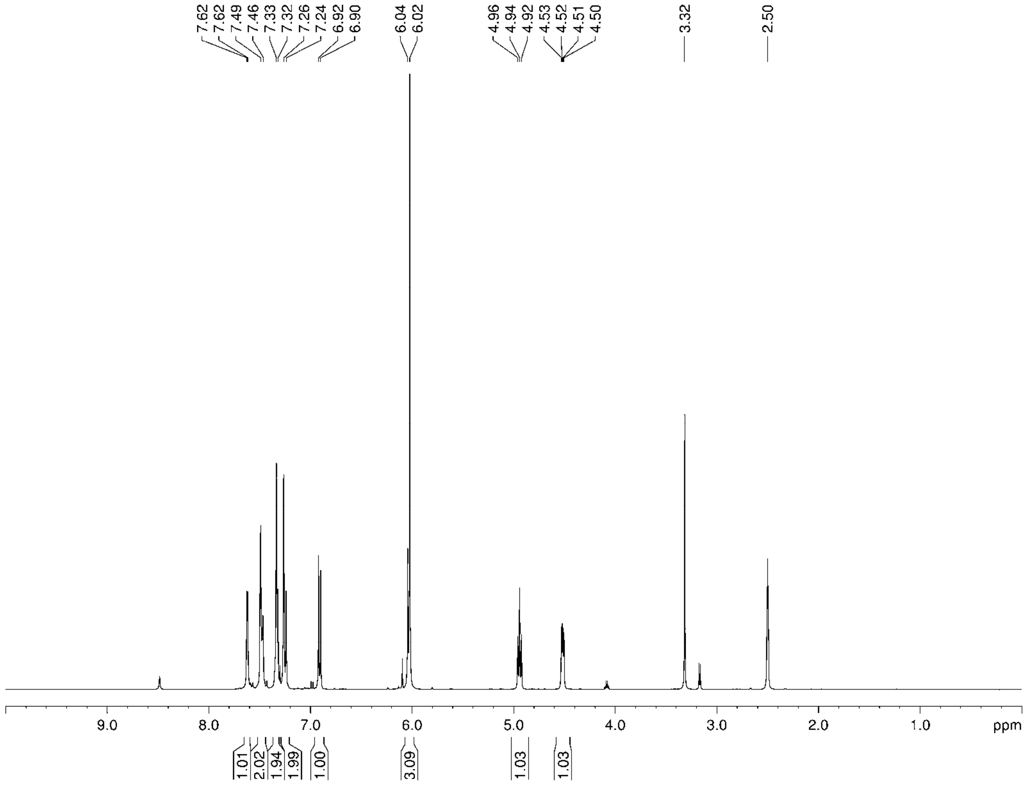
7.62 7.62 7.49 7.46 7.33 7.32 7.26 7.24 6.92 6.90
6.04 6.02
4.96 4.94 4.92 4.53 4.52 4.51 4.50
3.32
2.50
9.0 8.0 7.0 6.0 5.0 4.0 3.0 2.0 1.0 ppm
1.01 2.02 1.94 1.99 1.00 3.09 1.03 1.03

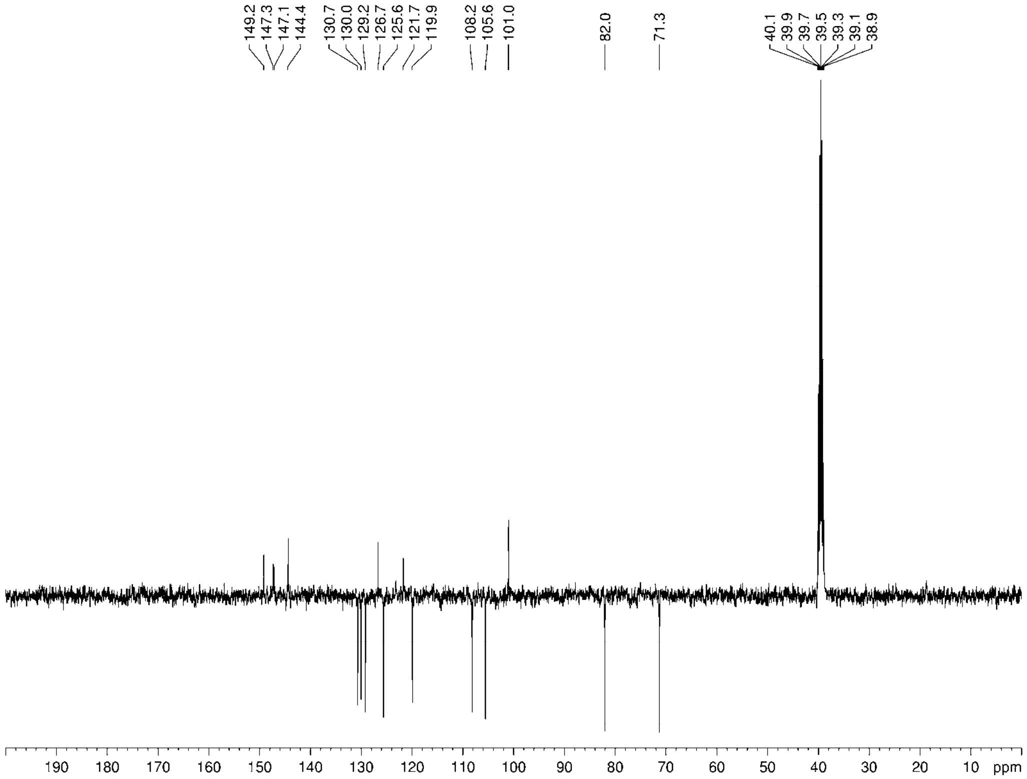
149.2 147.3 147.1 144.4
130.7 130.0 129.2 126.7 125.6 121.7 119.9
108.2 105.6 101.0
82.0
71.3
40.1 39.9 39.7 39.5 39.3 39.1 38.9
190 180 170 160 150 140 130 120 110 100 90 80 70 60 50 40 30 20 10 ppm

sery301

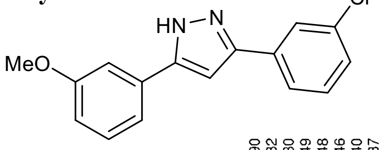

HN–N
Cl
MeO

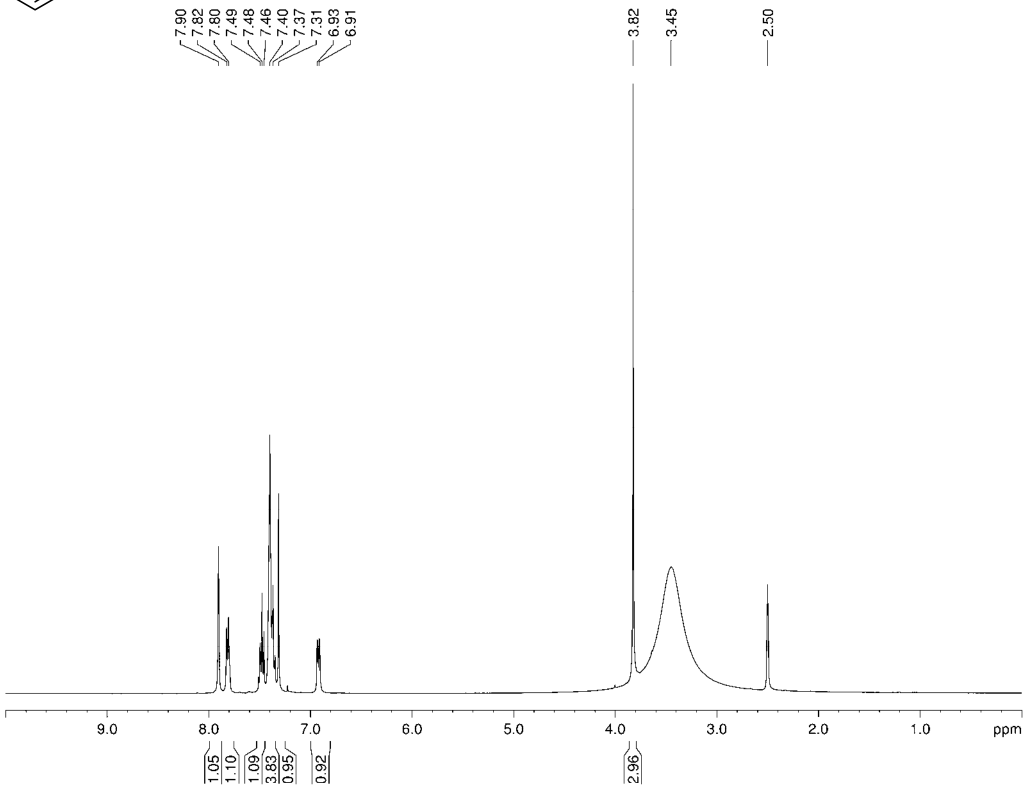

7.90
7.82
7.80
7.49
7.48
7.46
7.40
7.37
7.31
6.93
6.91
3.82
3.45
2.50
9.0
8.0
7.0
6.0
5.0
4.0
3.0
2.0
1.0
ppm
1.05
1.10
1.09
3.83
0.95
0.92
2.96

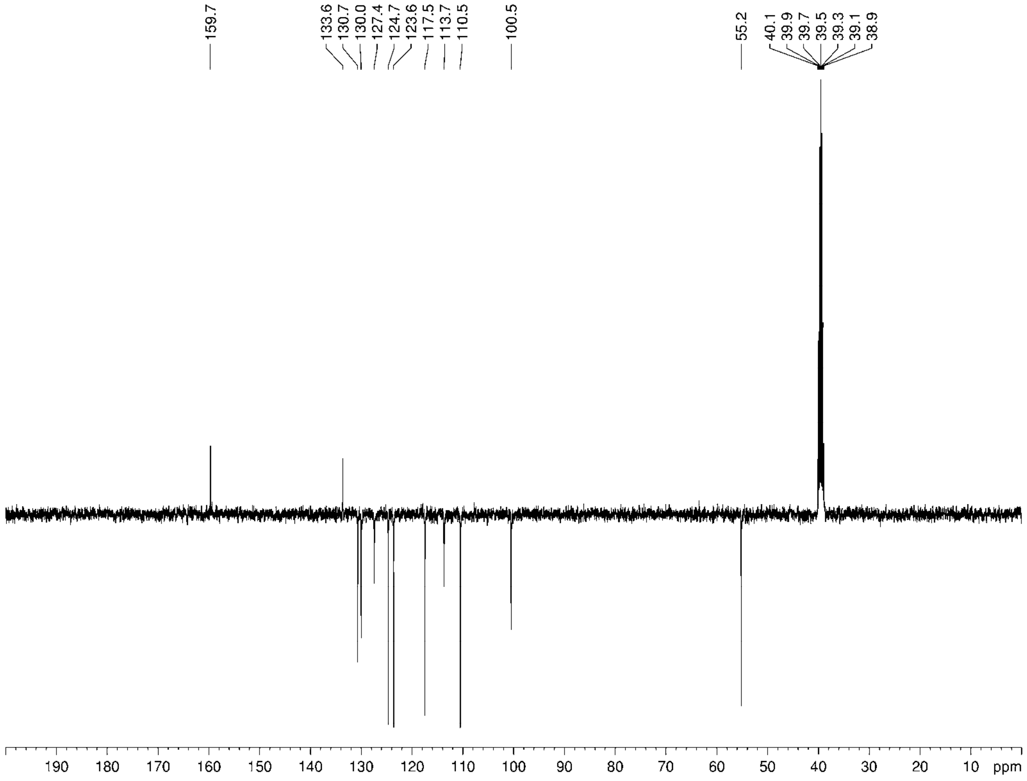

159.7
133.6
130.7
130.0
127.4
124.7
123.6
117.5
113.7
110.5
100.5
55.2
40.1
39.9
39.7
39.5
39.3
39.1
38.9
190
180
170
160
150
140
130
120
110
100
90
80
70
60
50
40
30
20
10
ppm

sery302A

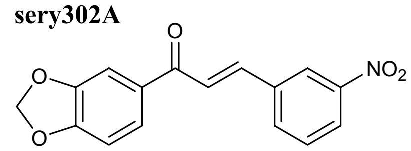

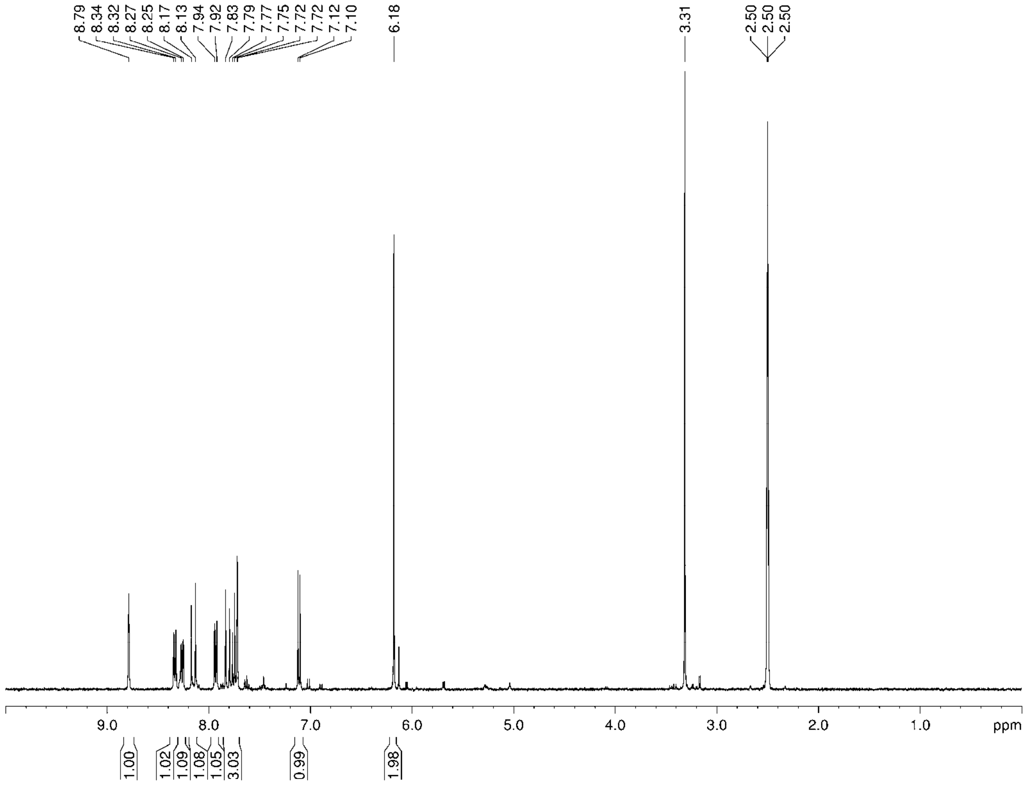

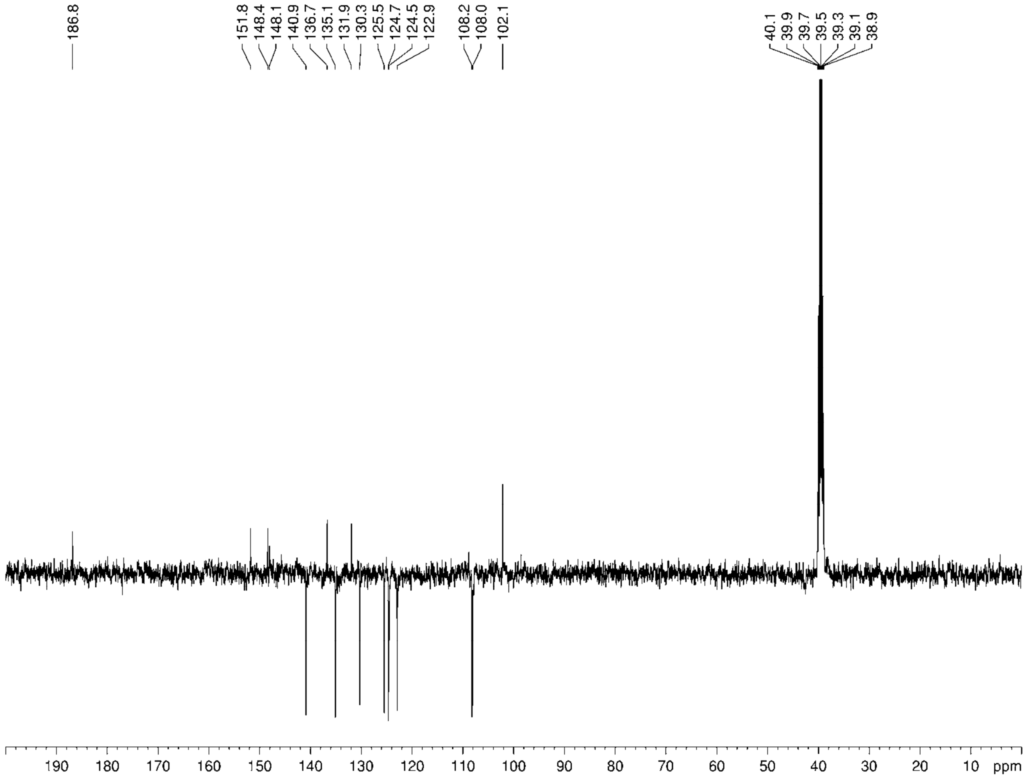

sery302B

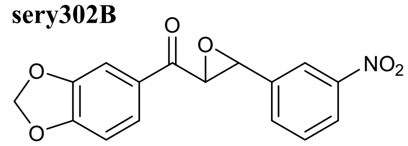

O
O
O
O
NO2

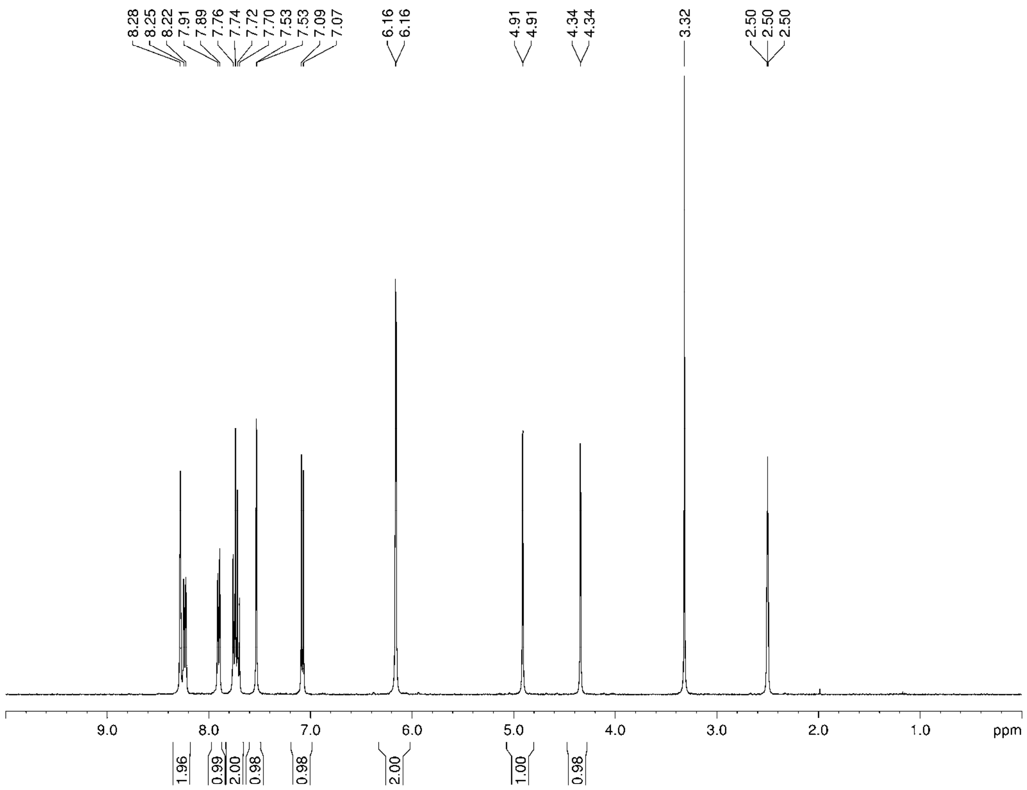

8.28
8.25
8.22
7.91
7.89
7.76
7.74
7.72
7.70
7.53
7.53
7.09
7.07
6.16
6.16
4.91
4.91
4.34
4.34
3.32
2.50
2.50
2.50
9.0
8.0
7.0
6.0
5.0
4.0
3.0
2.0
1.0
ppm
1.96
0.99
2.00
0.98
0.98
2.00
1.00
0.98

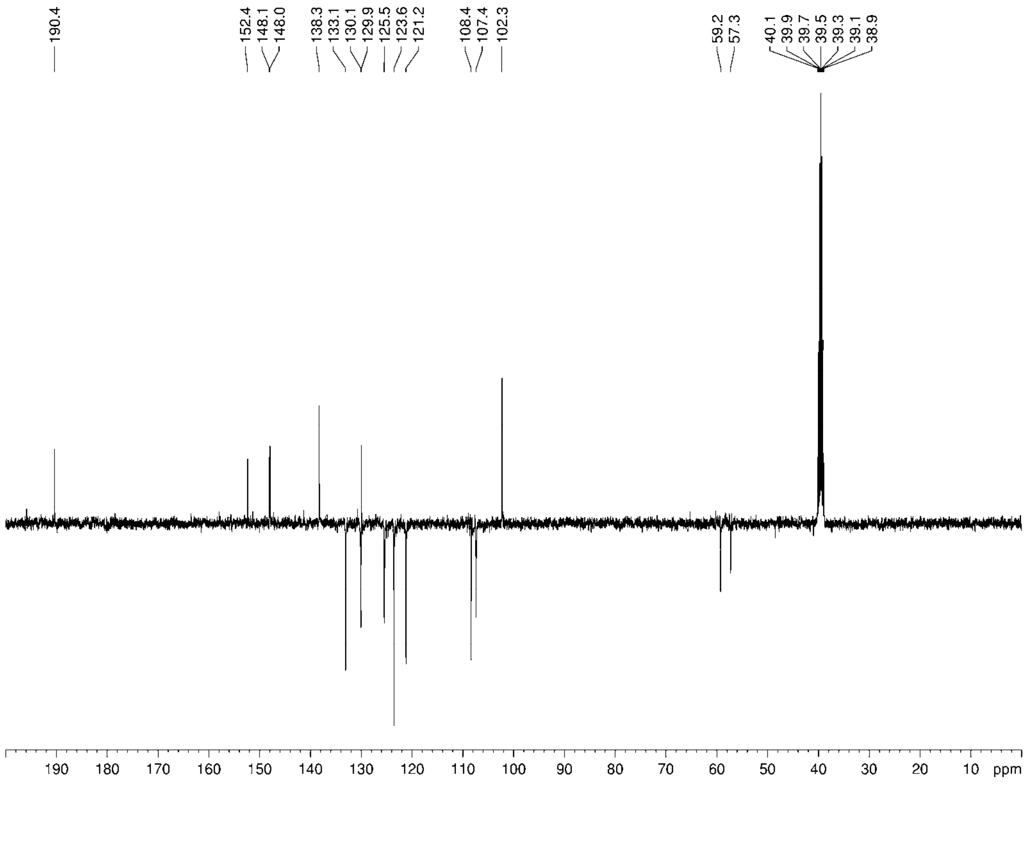

190.4
152.4
148.1
148.0
138.3
133.1
130.1
129.9
125.5
123.6
121.2
108.4
107.4
102.3
59.2
57.3
40.1
39.9
39.7
39.5
39.3
39.1
38.9
190
180
170
160
150
140
130
120
110
100
90
80
70
60
50
40
30
20
10
ppm

sery302C

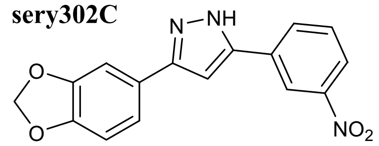
N–NH
O
O
NO2

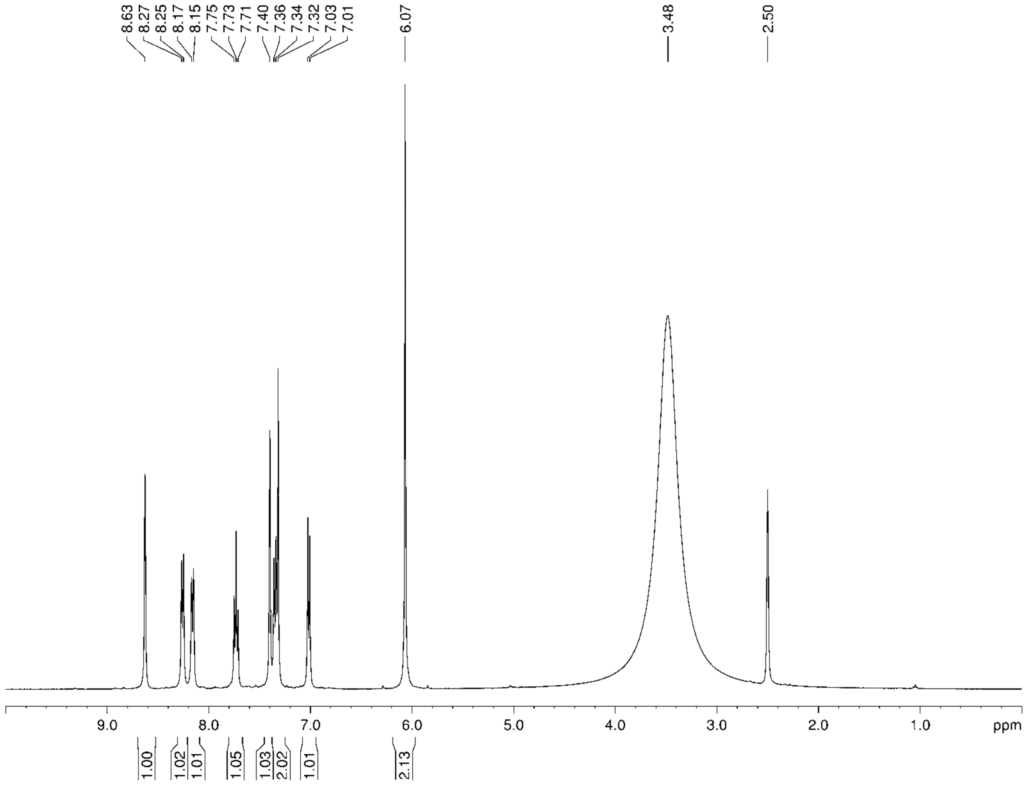
8.63
8.27
8.25
8.17
8.15
7.75
7.73
7.71
7.40
7.36
7.34
7.32
7.03
7.01
6.07
3.48
2.50
9.0 8.0 7.0 6.0 5.0 4.0 3.0 2.0 1.0 ppm
1.00
1.02
1.01
1.05
1.03
2.02
1.01
2.13

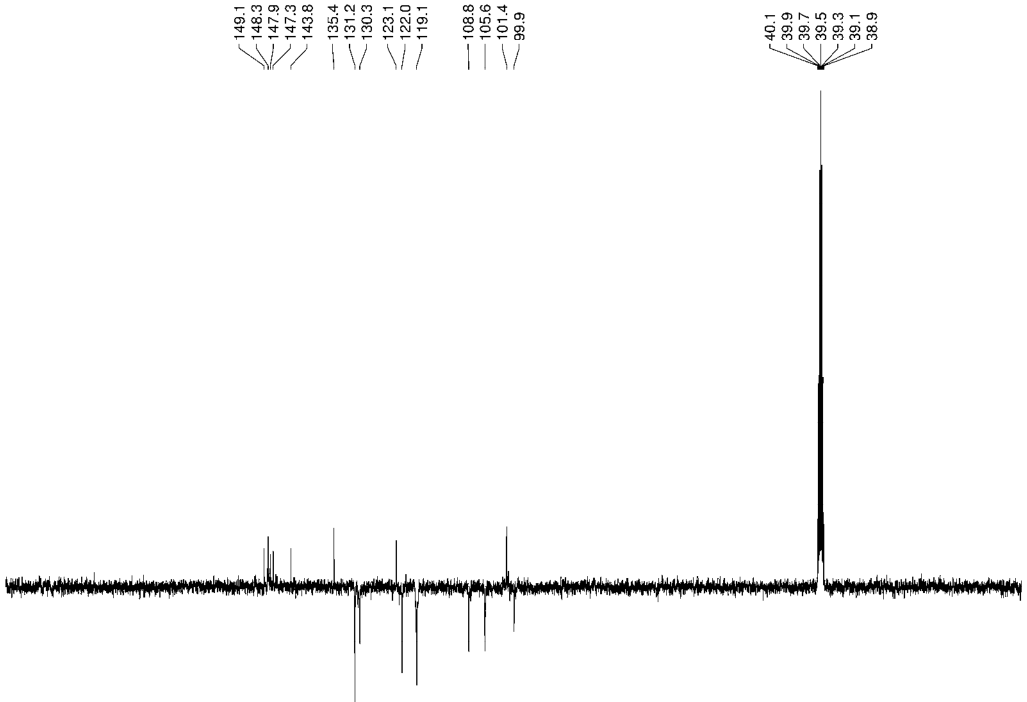
149.1
148.3
147.9
147.3
143.8
135.4
131.2
130.3
123.1
122.0
119.1
108.8
105.6
101.4
99.9
40.1
39.9
39.7
39.5
39.3
39.1
38.9
190 180 170 160 150 140 130 120 110 100 90 80 70 60 50 40 30 20 10 ppm

sery308

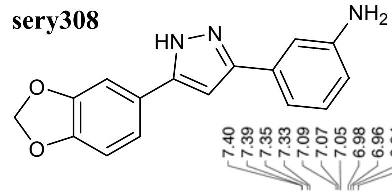
HN
N
NH2
O
O

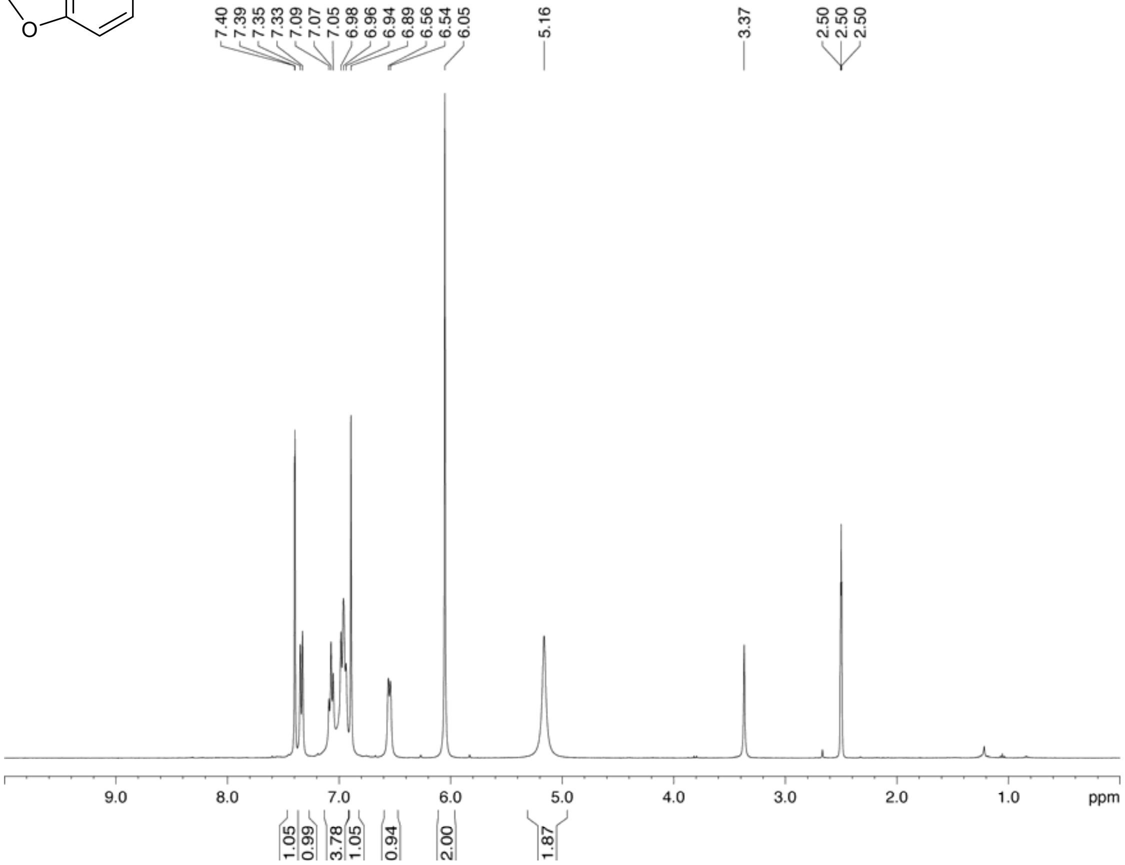
7.40 7.39 7.35 7.33 7.09 7.07 7.05 6.98 6.96 6.94 6.89 6.56 6.54 6.05
5.16
3.37
2.50 2.50 2.50
9.0 8.0 7.0 6.0 5.0 4.0 3.0 2.0 1.0 ppm
1.05 0.99 3.78 1.05 0.94 2.00 1.87

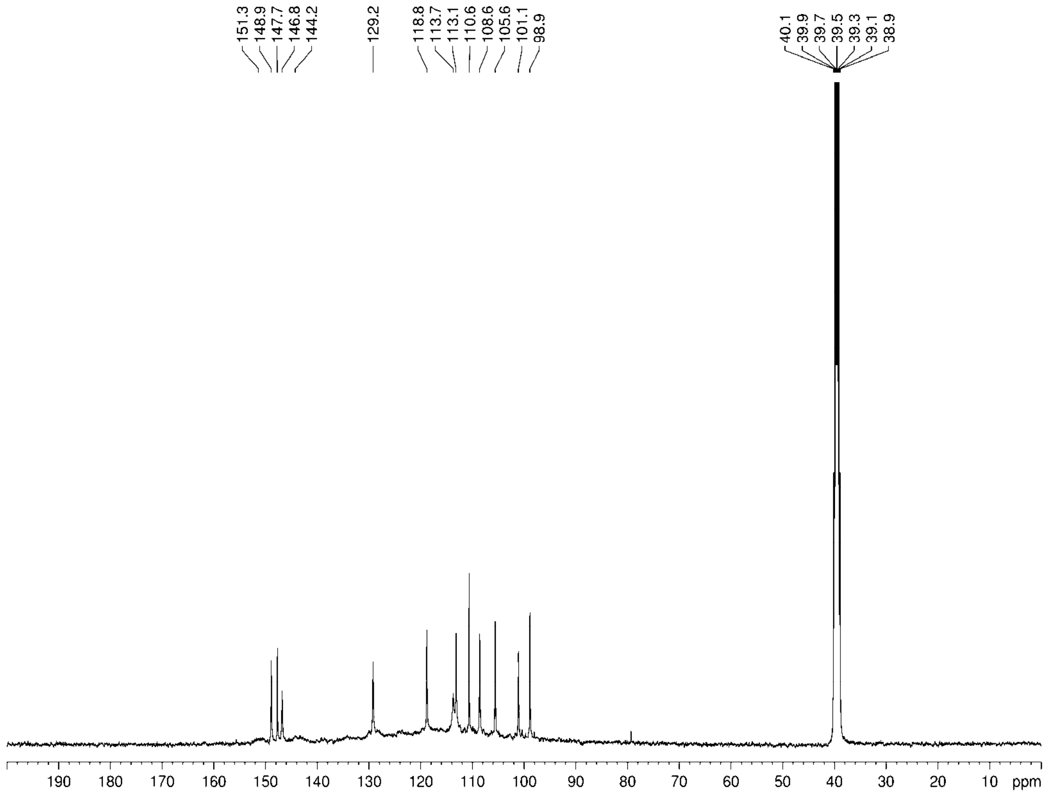
151.3 148.9 147.7 146.8 144.2
129.2
118.8 113.7 113.1 110.6 108.6 105.6 101.1 98.9
40.1 39.9 39.7 39.5 39.3 39.1 38.9
190 180 170 160 150 140 130 120 110 100 90 80 70 60 50 40 30 20 10 ppm

sery309

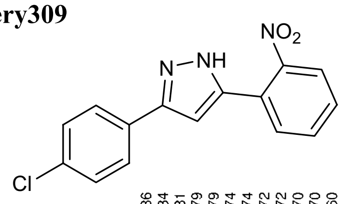

NO_2
N–NH
Cl

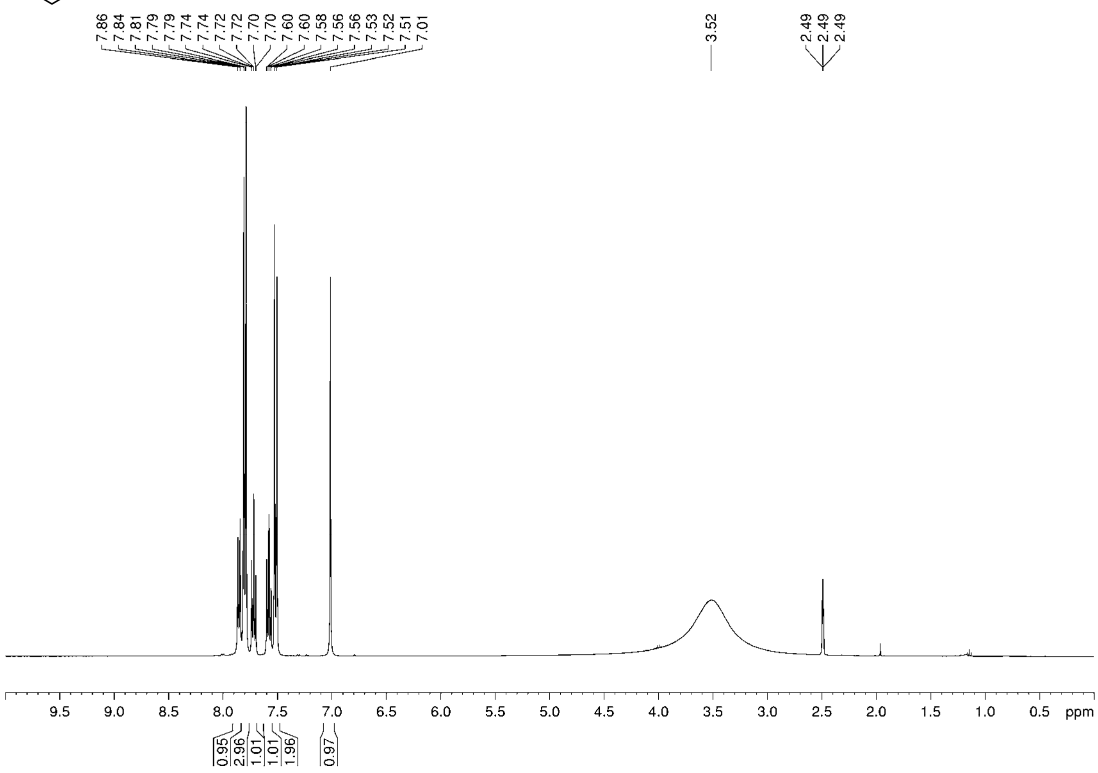

7.86 7.84 7.81 7.79 7.79 7.74 7.74 7.72 7.72 7.70 7.70 7.60 7.60 7.58 7.56 7.56 7.53 7.52 7.51 7.01
3.52
2.49 2.49 2.49
9.5 9.0 8.5 8.0 7.5 7.0 6.5 6.0 5.5 5.0 4.5 4.0 3.5 3.0 2.5 2.0 1.5 1.0 0.5 ppm
0.95 2.96 1.01 1.01 1.96 0.97

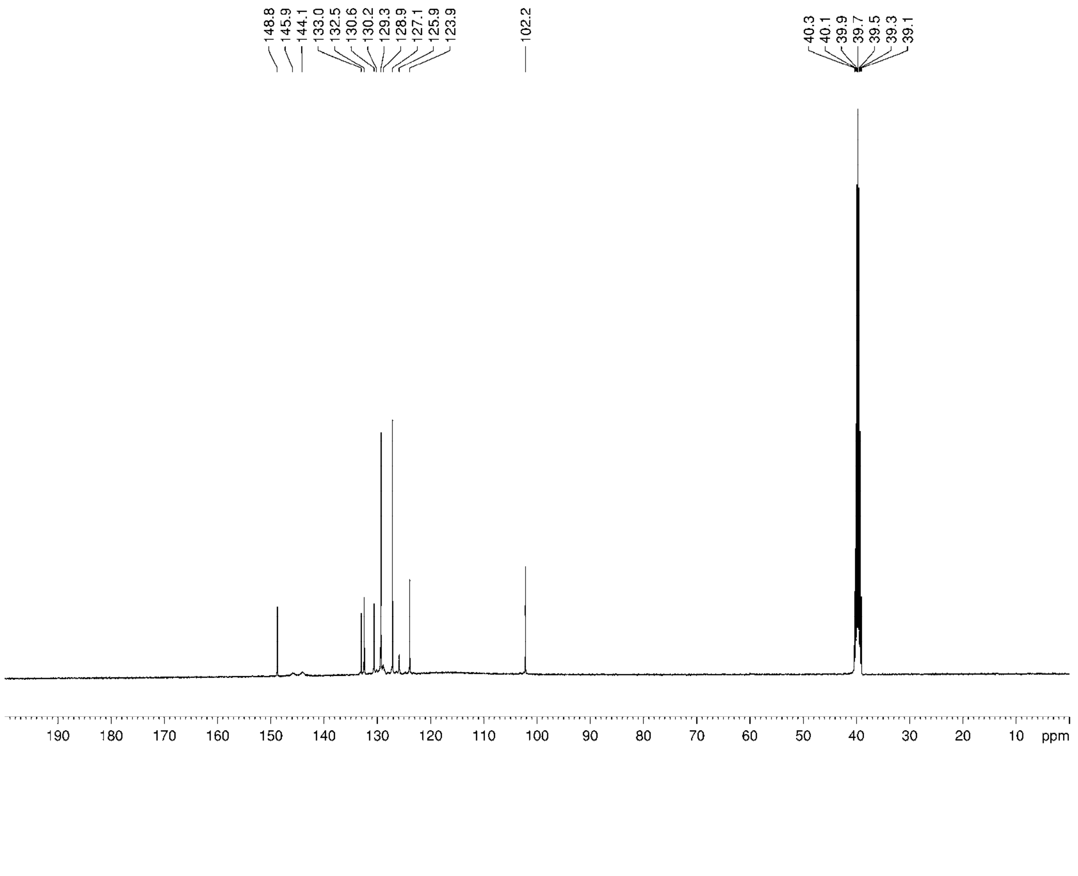

148.8 145.9 144.1 133.0 132.5 130.6 130.2 129.3 128.9 127.1 125.9 123.9
102.2
40.3 40.1 39.9 39.7 39.5 39.3 39.1
190 180 170 160 150 140 130 120 110 100 90 80 70 60 50 40 30 20 10 ppm

sery310B

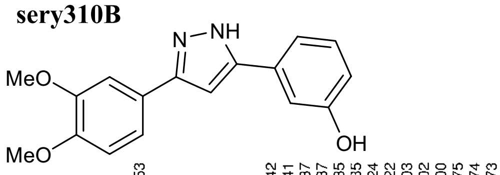
N–NH
MeO
MeO
OH

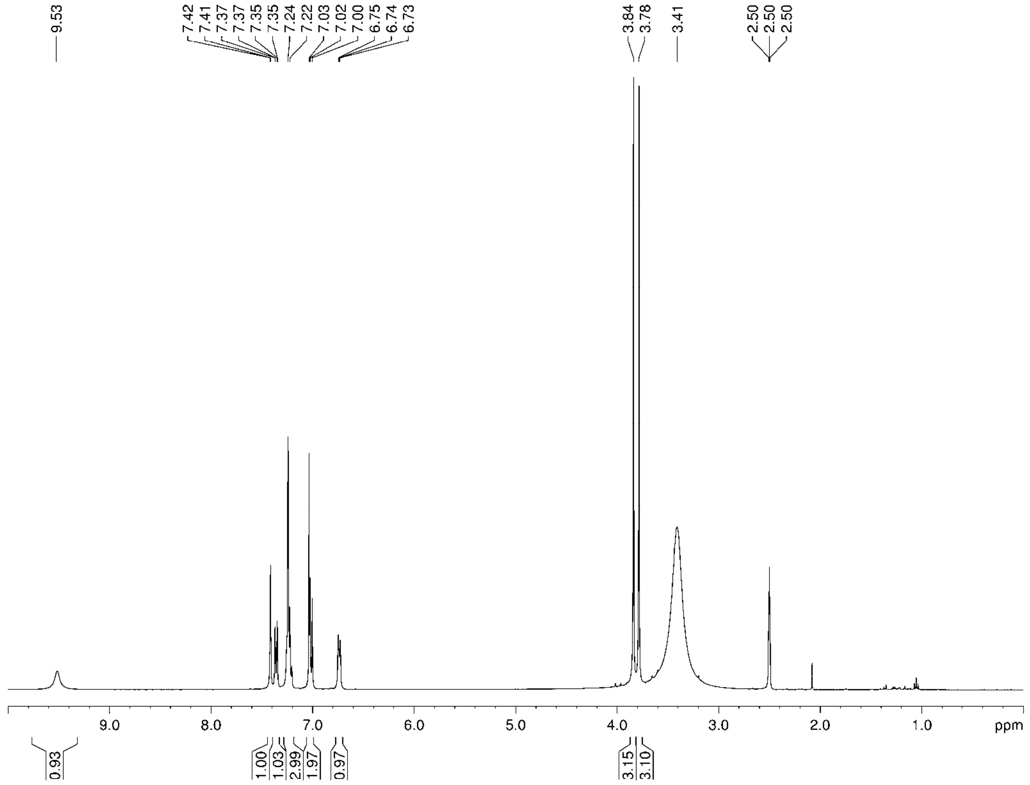
9.53
7.42
7.41
7.37
7.37
7.35
7.35
7.24
7.22
7.03
7.02
7.00
6.75
6.74
6.73
3.84
3.78
3.41
2.50
2.50
2.50
9.0
8.0
7.0
6.0
5.0
4.0
3.0
2.0
1.0
ppm
0.93
1.00
1.03
2.99
1.97
0.97
3.15
3.10

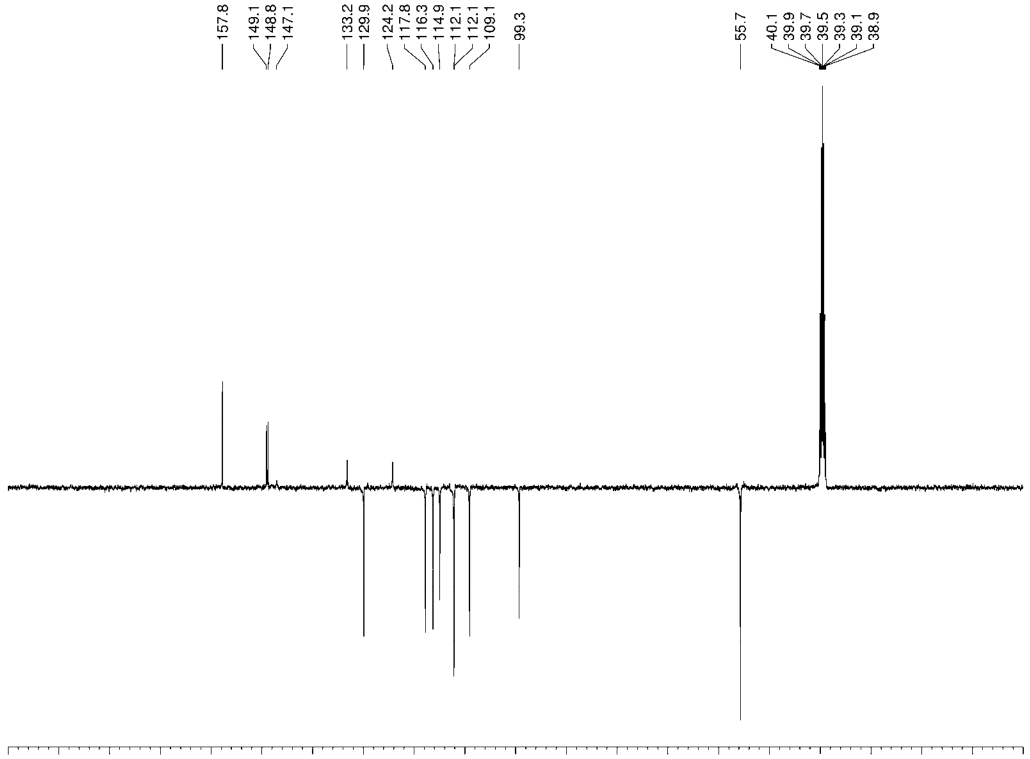
157.8
149.1
148.8
147.1
133.2
129.9
124.2
117.8
116.3
114.9
112.1
112.1
109.1
99.3
55.7
40.1
39.9
39.7
39.5
39.3
39.1
38.9
190
180
170
160
150
140
130
120
110
100
90
80
70
60
50
40
30
20
10
ppm

sery312A

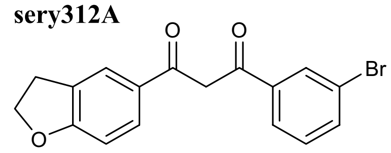
O
O
Br
O

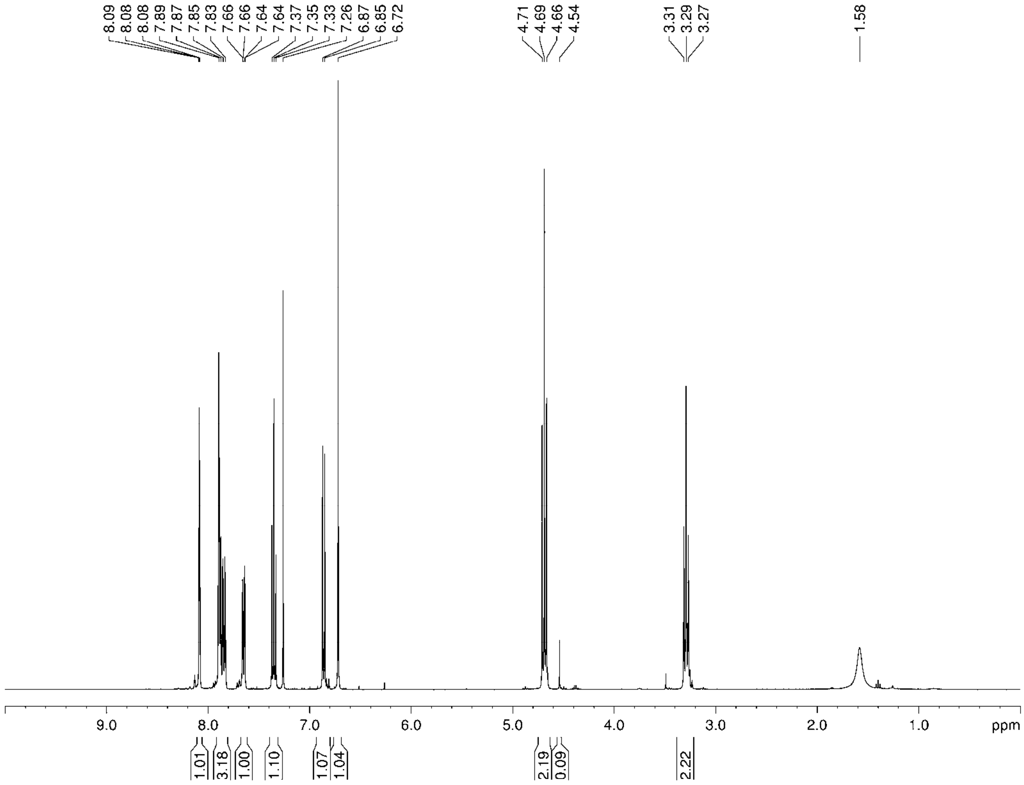
8.09
8.08
8.08
7.89
7.87
7.85
7.83
7.66
7.66
7.64
7.64
7.37
7.35
7.33
7.26
6.87
6.85
6.72
4.71
4.69
4.66
4.54
3.31
3.29
3.27
1.58
9.0
8.0
7.0
6.0
5.0
4.0
3.0
2.0
1.0
ppm
1.01
3.18
1.00
1.10
1.07
1.04
2.19
0.09
2.22

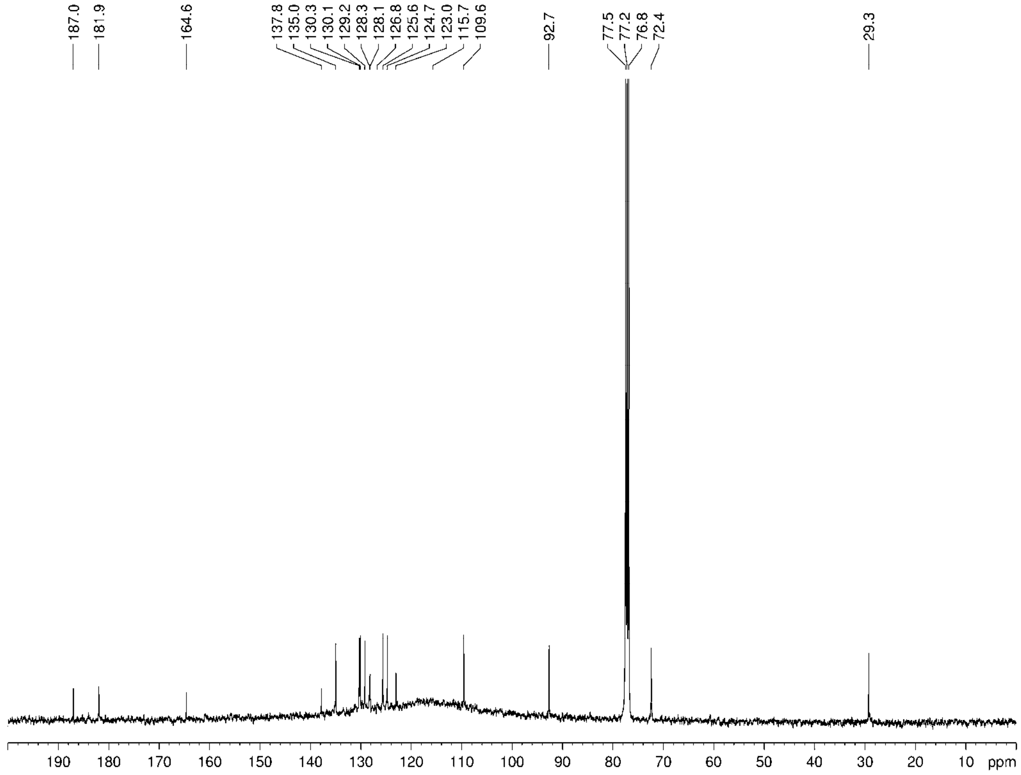
187.0
181.9
164.6
137.8
135.0
130.3
130.1
129.2
128.3
128.1
126.8
125.6
124.7
123.0
115.7
109.6
92.7
77.5
77.2
76.8
72.4
29.3
190
180
170
160
150
140
130
120
110
100
90
80
70
60
50
40
30
20
10
ppm

sery312B

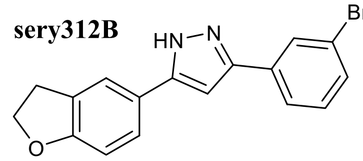
HN
N
Br
O

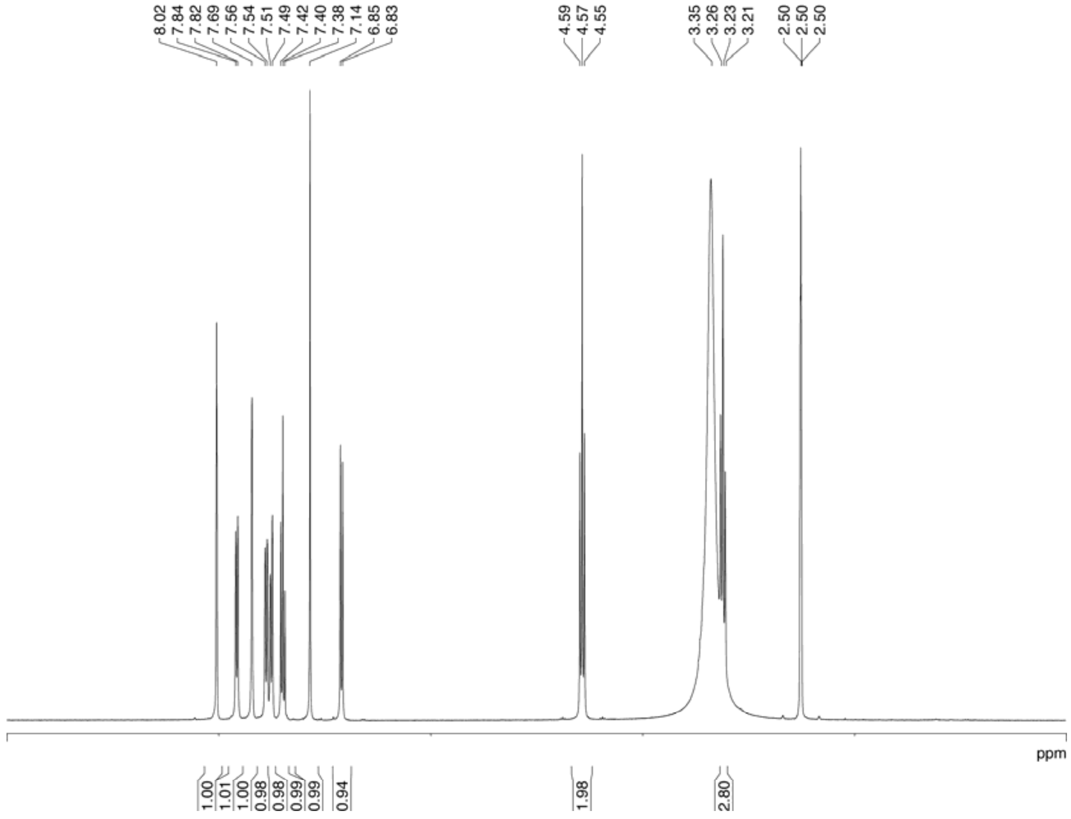
8.02
7.84
7.82
7.69
7.56
7.54
7.51
7.49
7.42
7.40
7.38
7.14
6.85
6.83
4.59
4.57
4.55
3.35
3.26
3.23
3.21
2.50
2.50
2.50
ppm
1.00
1.01
1.00
0.98
0.98
0.99
0.99
0.94
1.98
2.80

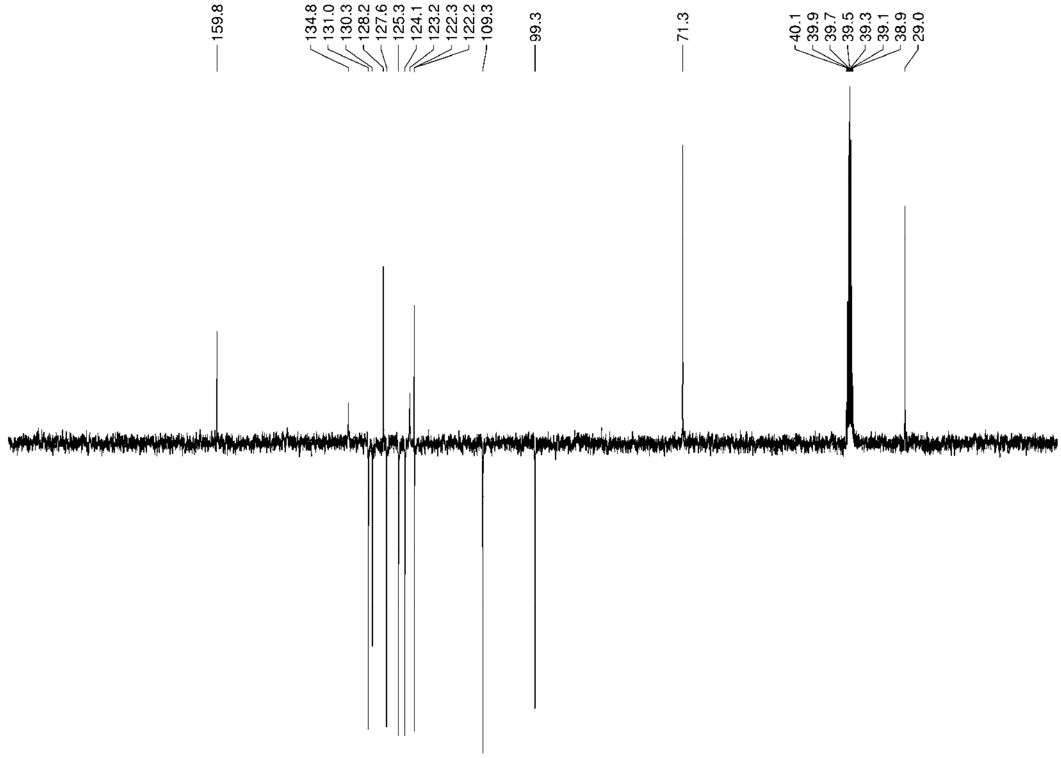
159.8
134.8
131.0
130.3
128.2
127.6
125.3
124.1
123.2
122.3
122.2
109.3
99.3
71.3
40.1
39.9
39.7
39.5
39.3
39.1
38.9
29.0
190
180
170
160
150
140
130
120
110
100
90
80
70
60
50
40
30
20
10
ppm

sery315A

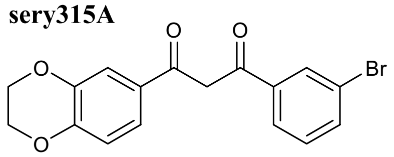

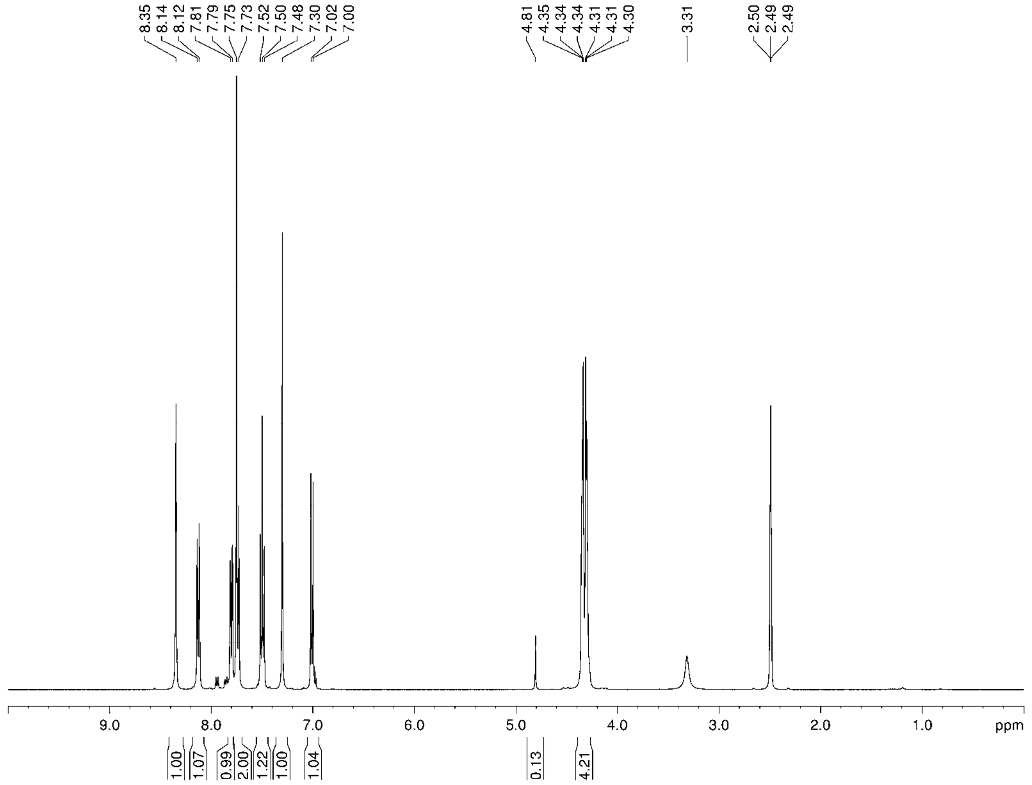

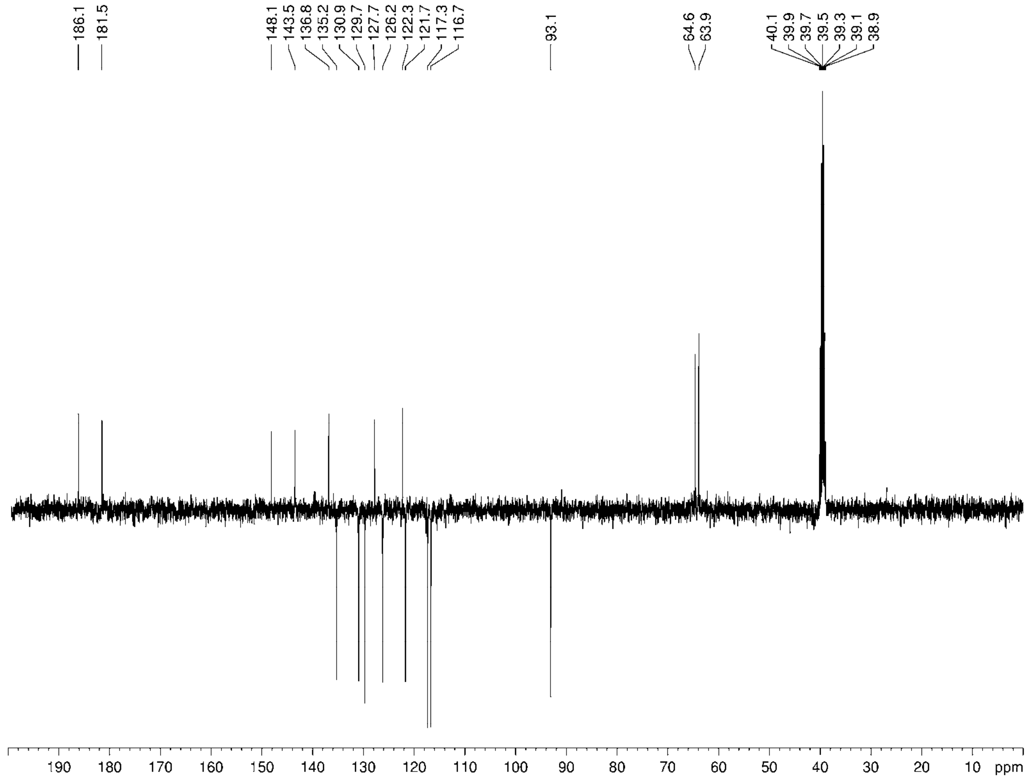

sery315B

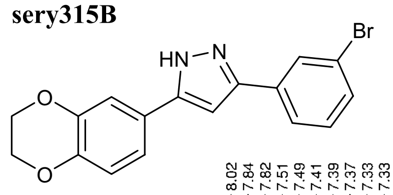

Br
HN
N
O
O

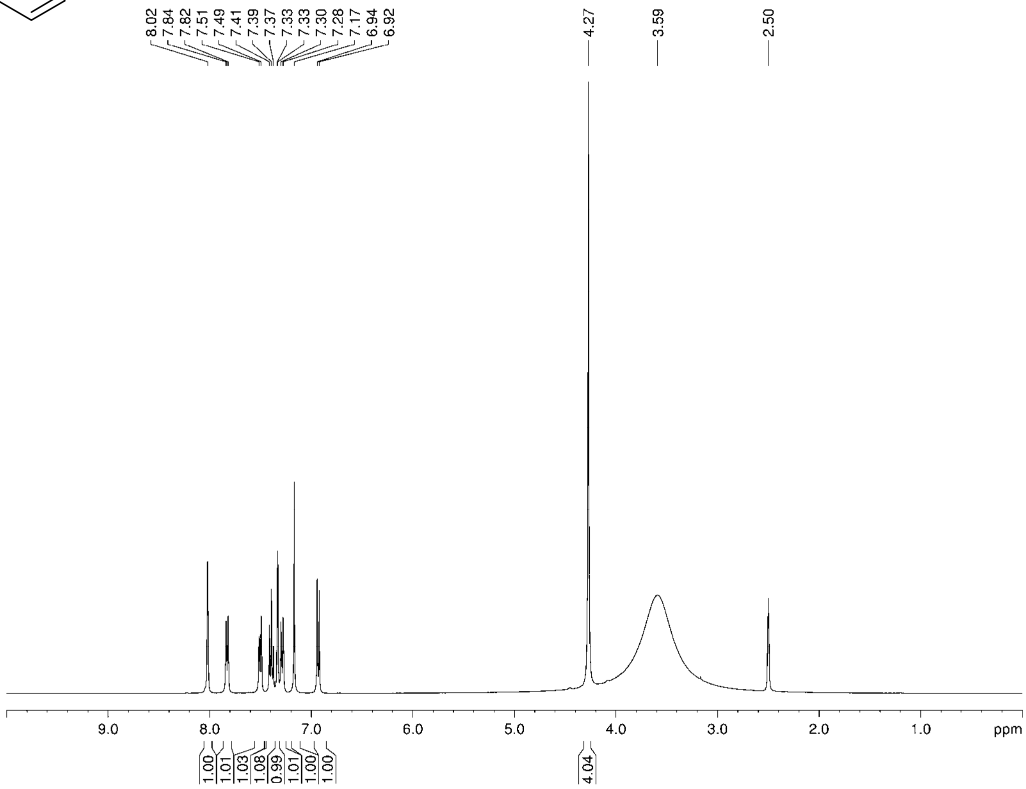

8.02 7.84 7.82 7.51 7.49 7.41 7.39 7.37 7.33 7.33 7.30 7.28 7.17 6.94 6.92
4.27
3.59
2.50
9.0 8.0 7.0 6.0 5.0 4.0 3.0 2.0 1.0 ppm
1.00 1.01 1.03 1.08 0.99 1.01 1.00 1.00
4.04

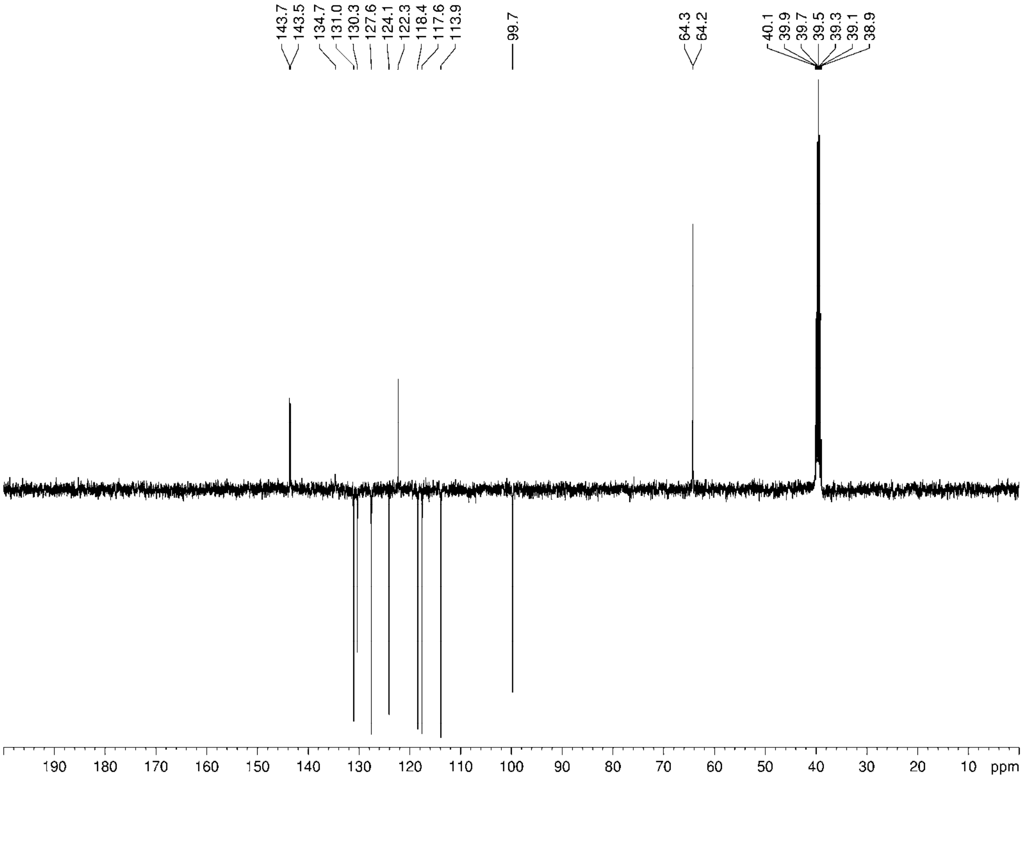

143.7 143.5 134.7 131.0 130.3 127.6 124.1 122.3 118.4 117.6 113.9
99.7
64.3 64.2
40.1 39.9 39.7 39.5 39.3 39.1 38.9
190 180 170 160 150 140 130 120 110 100 90 80 70 60 50 40 30 20 10 ppm

sery316A

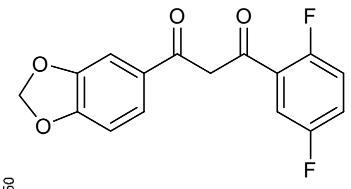
O
O
F
O
O
F

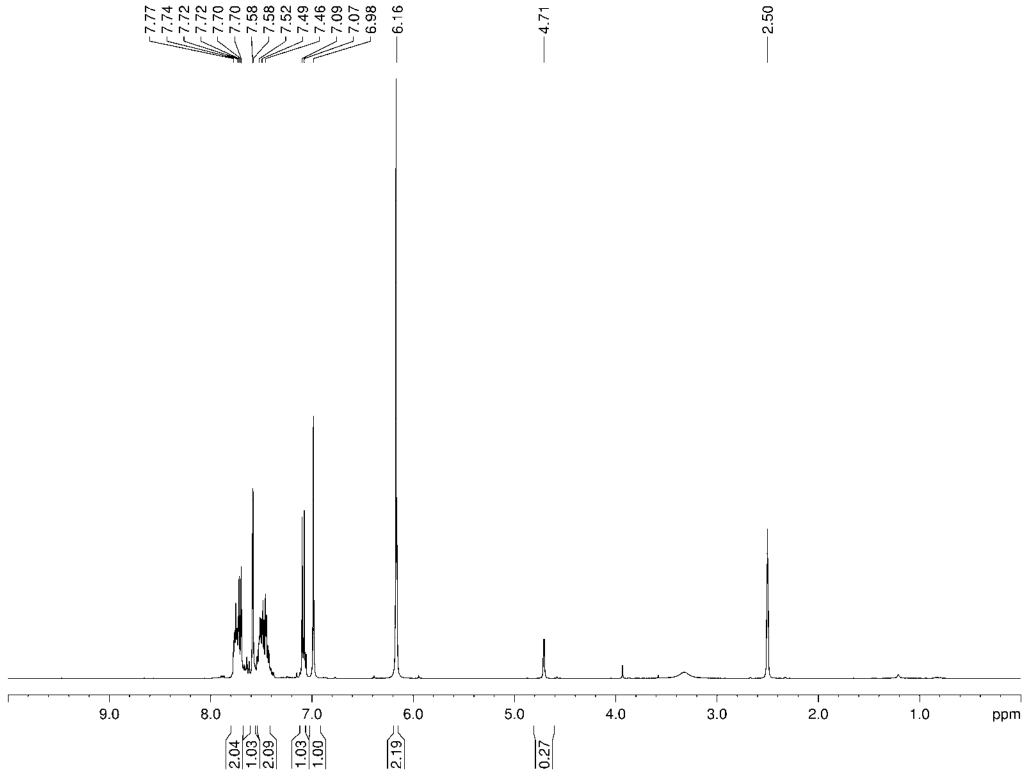
7.77
7.74
7.72
7.72
7.70
7.70
7.58
7.58
7.52
7.49
7.46
7.09
7.07
6.98
6.16
4.71
2.50
9.0
8.0
7.0
6.0
5.0
4.0
3.0
2.0
1.0
ppm
2.04
1.03
2.09
1.03
1.00
2.19
0.27

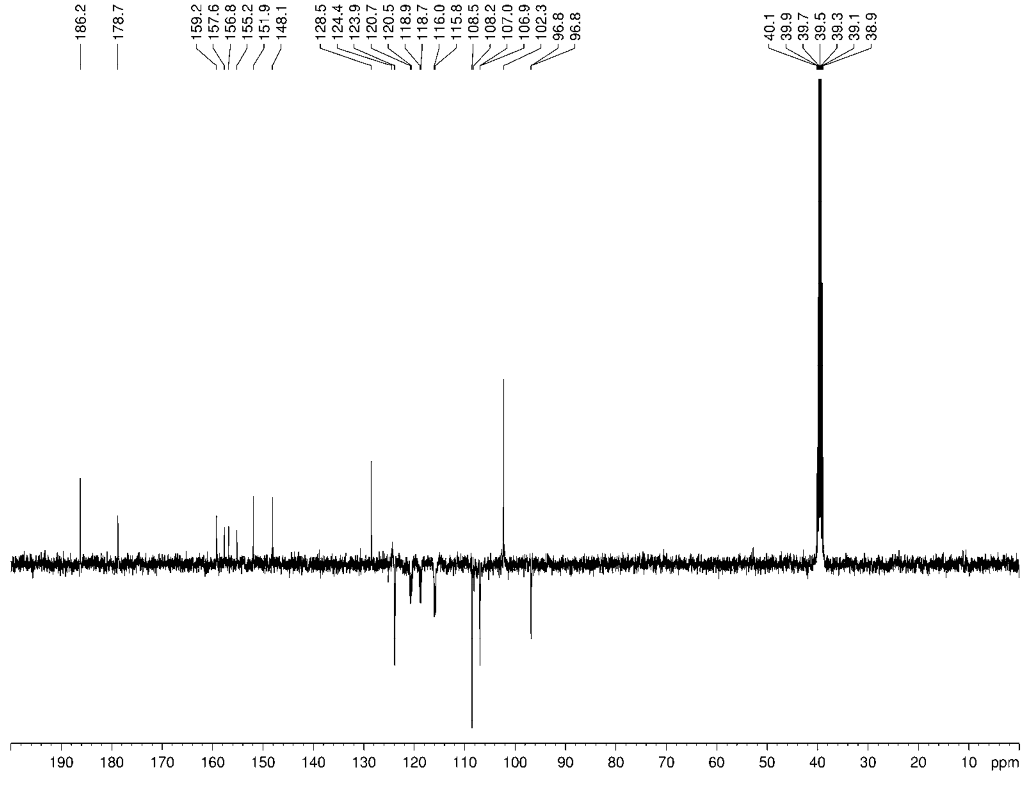
186.2
178.7
159.2
157.6
156.8
155.2
151.9
148.1
128.5
124.4
123.9
120.7
120.5
118.9
118.7
116.0
115.8
108.5
108.2
107.0
106.9
102.3
96.8
96.8
40.1
39.9
39.7
39.5
39.3
39.1
38.9
190
180
170
160
150
140
130
120
110
100
90
80
70
60
50
40
30
20
10
ppm

sery316B

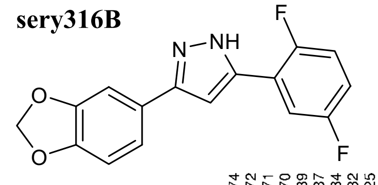

N–NH
F
O
O
F

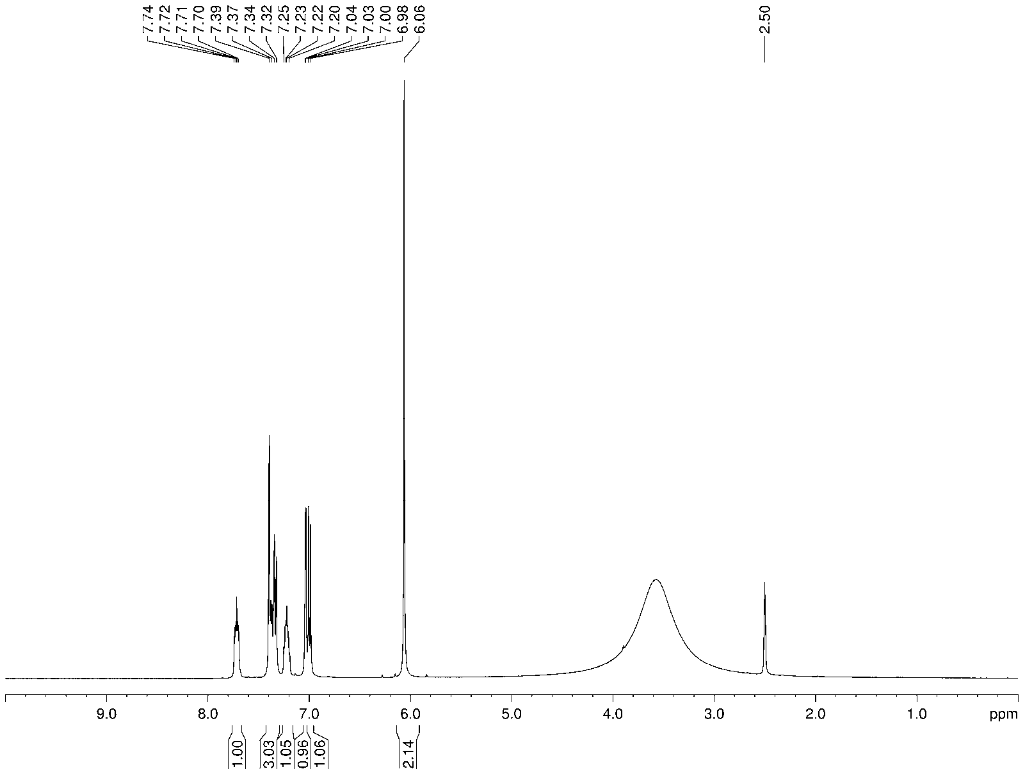

7.74 7.72 7.71 7.70 7.39 7.37 7.34 7.32 7.25 7.23 7.22 7.20 7.04 7.03 7.00 6.98 6.06
2.50
9.0 8.0 7.0 6.0 5.0 4.0 3.0 2.0 1.0 ppm
1.00 3.03 1.05 0.96 1.06 2.14

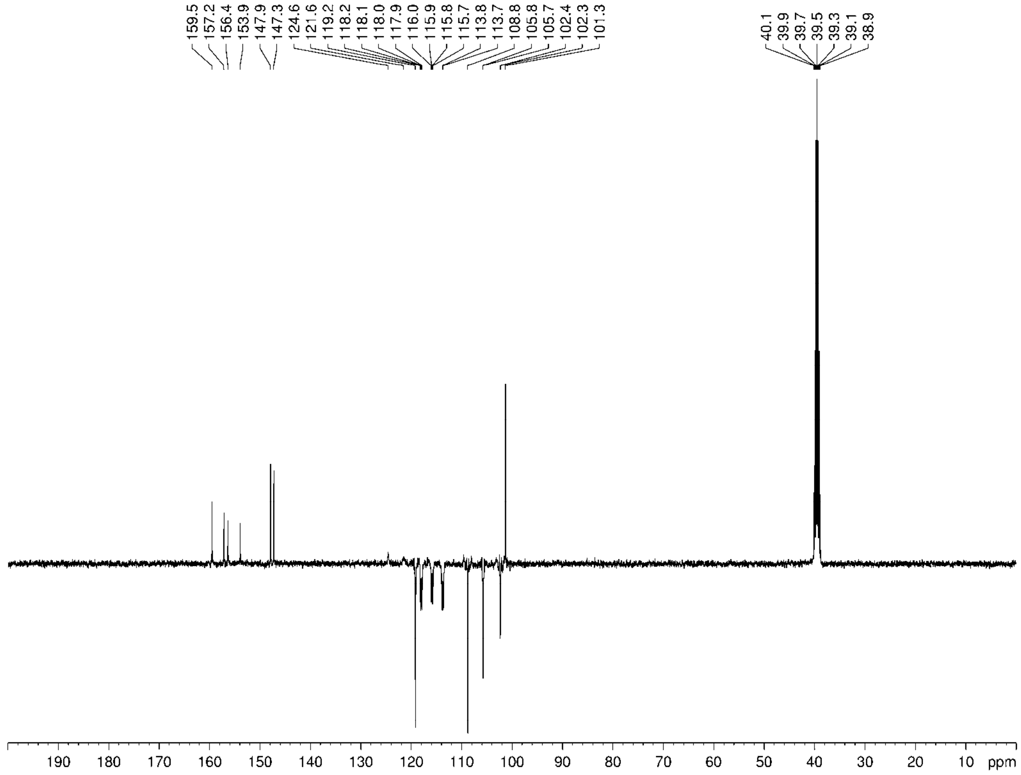

159.5 157.2 156.4 153.9 147.9 147.3 124.6 121.6 119.2 118.2 118.1 118.0 117.9 116.0 115.9 115.8 115.7 113.8 113.7 108.8 105.8 105.7 102.4 102.3 101.3
40.1 39.9 39.7 39.5 39.3 39.1 38.9
190 180 170 160 150 140 130 120 110 100 90 80 70 60 50 40 30 20 10 ppm

sery318A

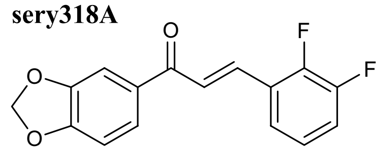
O
O
O
F
F

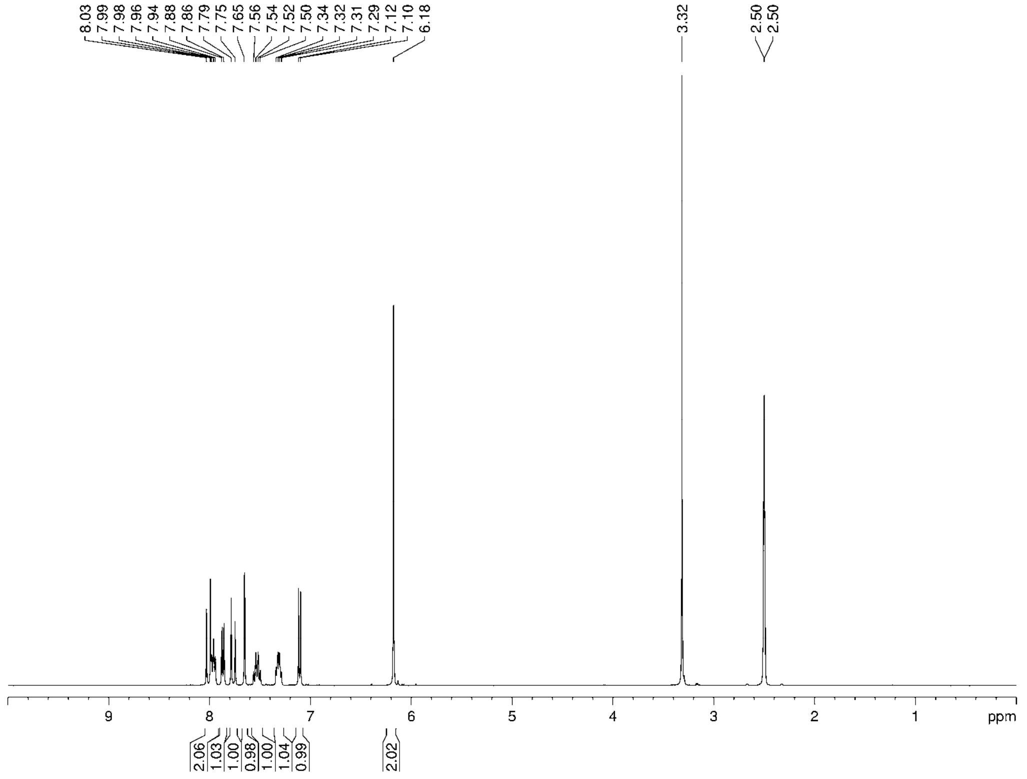
8.03
7.99
7.98
7.96
7.94
7.88
7.86
7.79
7.75
7.65
7.56
7.54
7.52
7.50
7.34
7.32
7.31
7.29
7.12
7.10
6.18
3.32
2.50
2.50
9
8
7
6
5
4
3
2
1
ppm
2.06
1.03
1.00
0.98
1.00
1.04
0.99
2.02

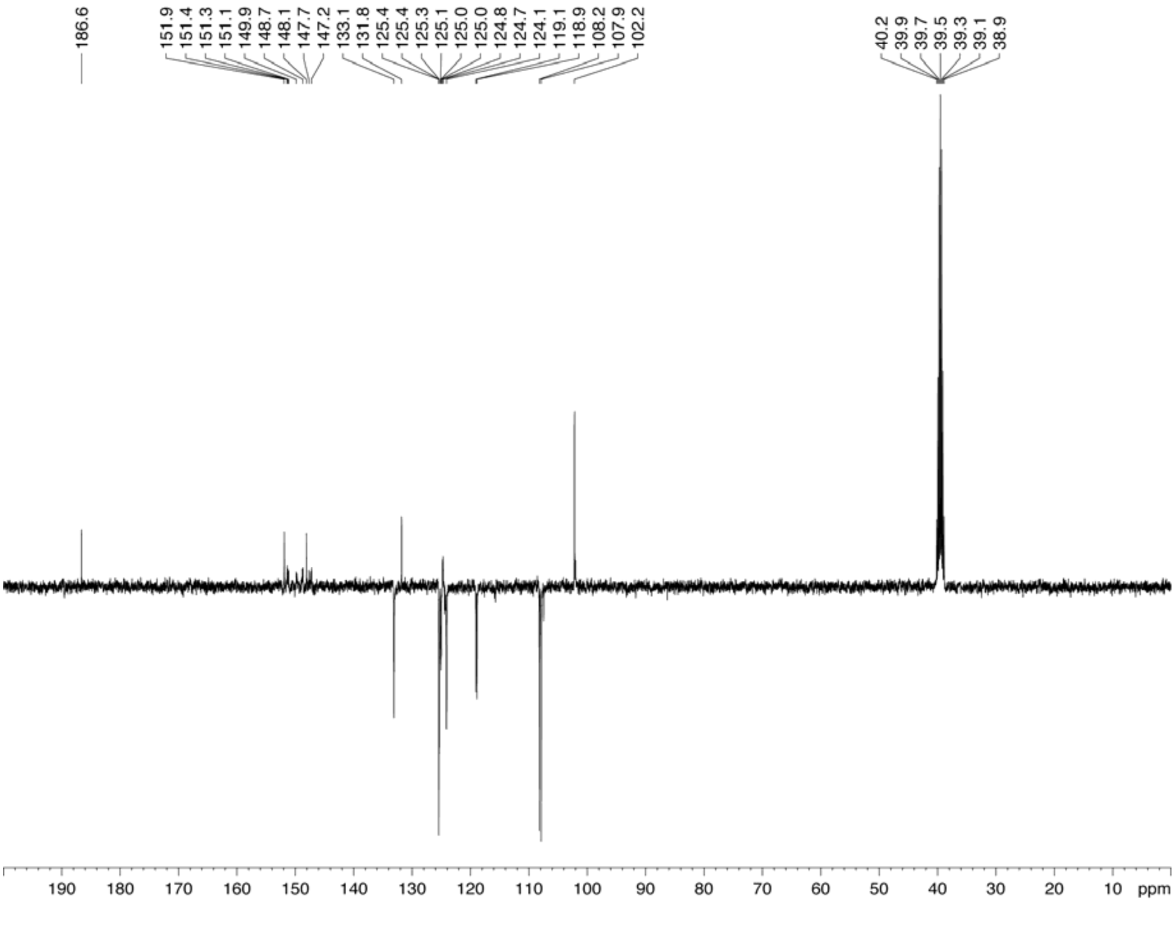
186.6
151.9
151.4
151.3
151.1
149.9
148.7
148.1
147.7
147.2
133.1
131.8
125.4
125.3
125.1
125.0
124.8
124.7
124.1
119.1
118.9
108.2
107.9
102.2
40.2
39.9
39.7
39.5
39.3
39.1
38.9
190
180
170
160
150
140
130
120
110
100
90
80
70
60
50
40
30
20
10
ppm

sery318B

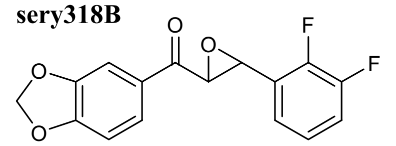
O
O
O
O
F
F

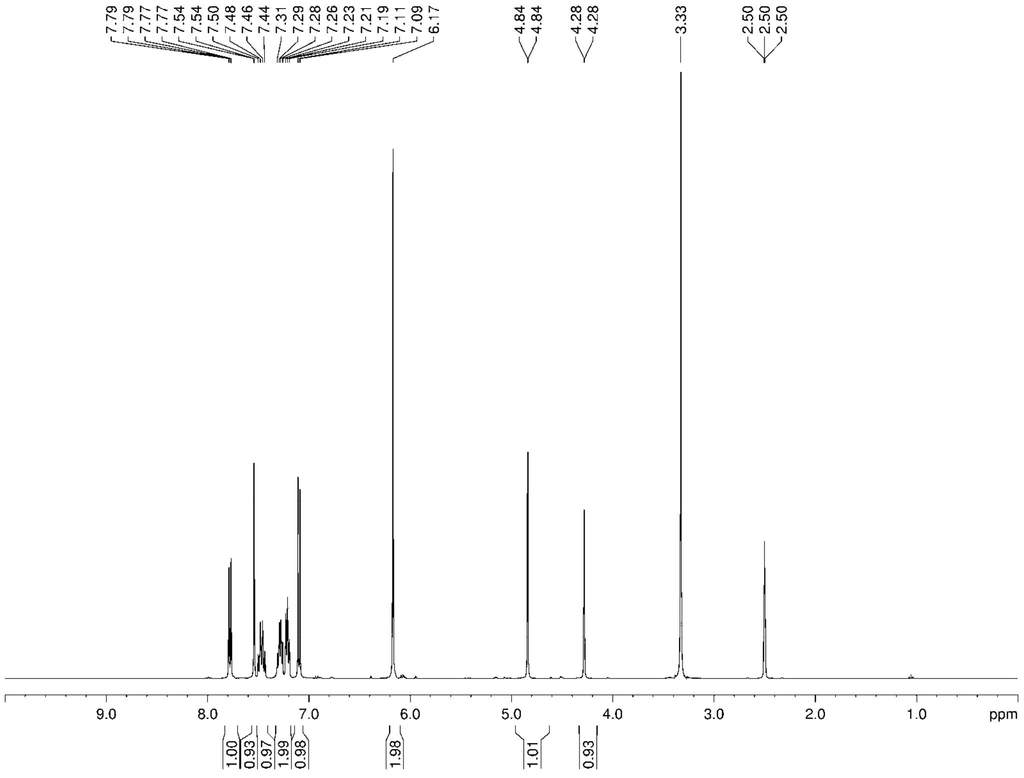
7.79
7.79
7.77
7.77
7.54
7.54
7.50
7.48
7.46
7.44
7.31
7.29
7.28
7.26
7.23
7.21
7.19
7.11
7.09
6.17
4.84
4.84
4.28
4.28
3.33
2.50
2.50
2.50
9.0
8.0
7.0
6.0
5.0
4.0
3.0
2.0
1.0
ppm
1.00
0.93
0.97
1.99
0.98
1.98
1.01
0.93

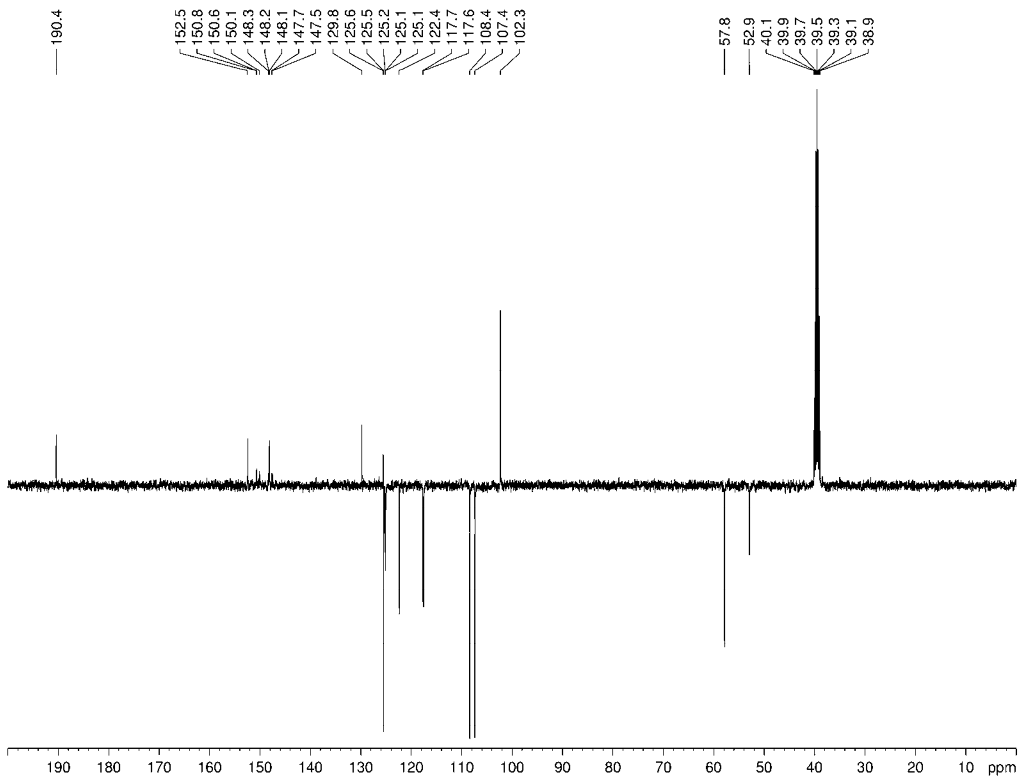
190.4
152.5
150.8
150.6
150.1
148.3
148.2
148.1
147.7
147.5
129.8
125.6
125.5
125.2
125.1
125.1
122.4
117.7
117.6
108.4
107.4
102.3
57.8
52.9
40.1
39.9
39.7
39.5
39.3
39.1
38.9
190
180
170
160
150
140
130
120
110
100
90
80
70
60
50
40
30
20
10
ppm

sery319

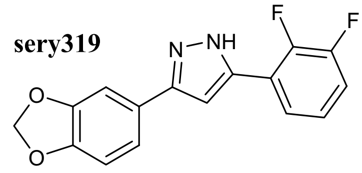

N
NH
F
F
O
O

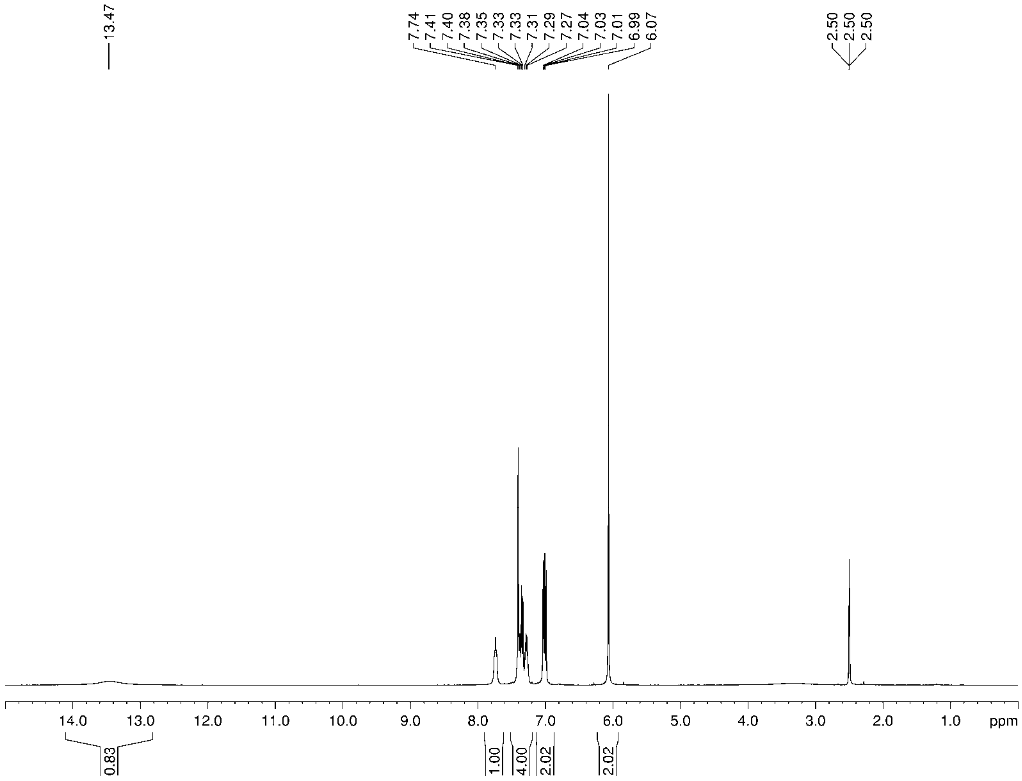

13.47
7.74
7.41
7.40
7.38
7.35
7.33
7.33
7.31
7.29
7.27
7.04
7.03
7.01
6.99
6.07
2.50
2.50
2.50
14.0 13.0 12.0 11.0 10.0 9.0 8.0 7.0 6.0 5.0 4.0 3.0 2.0 1.0 ppm
0.83
1.00
4.00
2.02
2.02

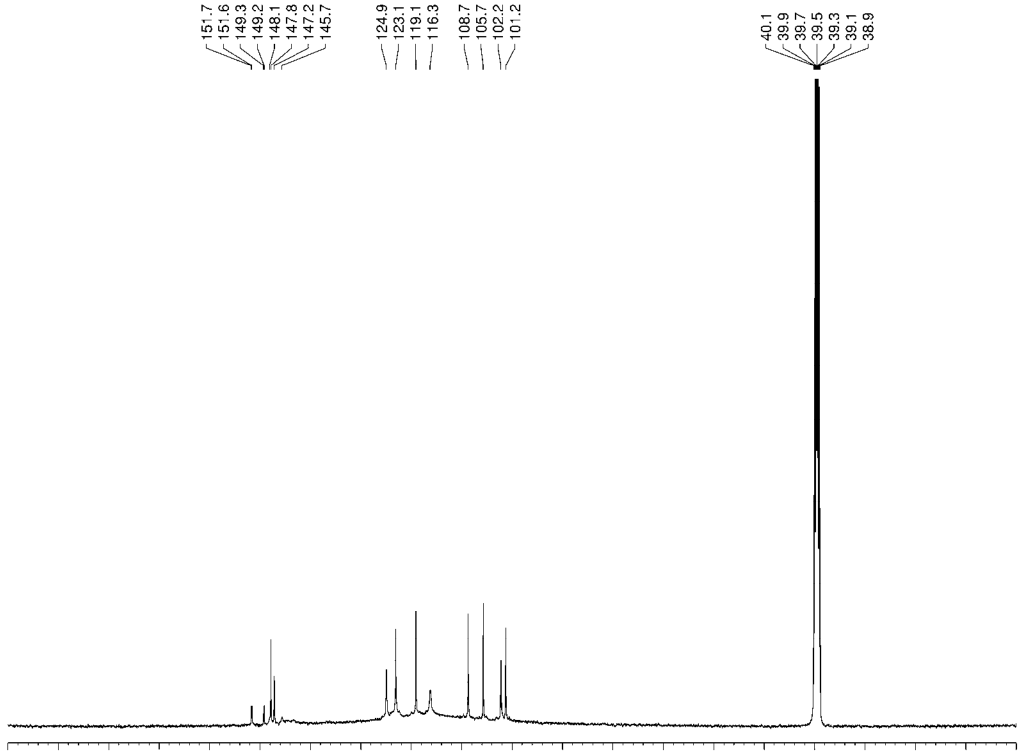

151.7
151.6
149.3
149.2
148.1
147.8
147.2
145.7
124.9
123.1
119.1
116.3
108.7
105.7
102.2
101.2
40.1
39.9
39.7
39.5
39.3
39.1
38.9
190 180 170 160 150 140 130 120 110 100 90 80 70 60 50 40 30 20 10 ppm

sery320A

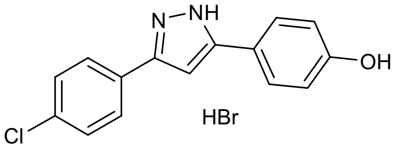

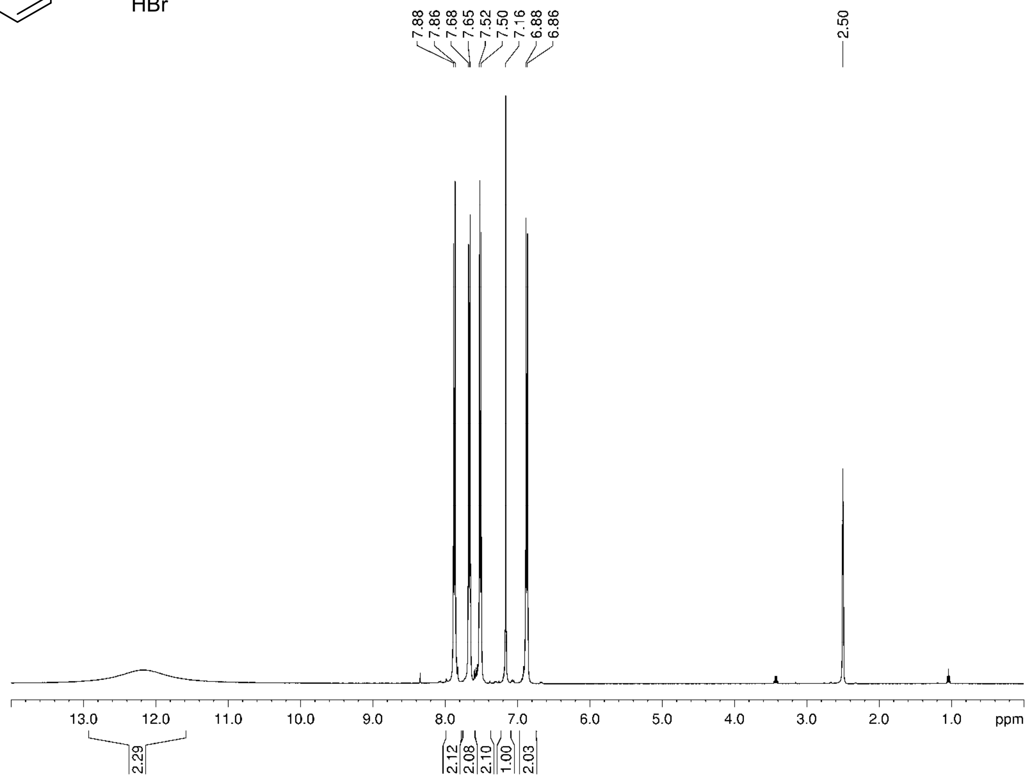

7.88
7.86
7.68
7.65
7.52
7.50
7.16
6.88
6.86
2.50
13.0 12.0 11.0 10.0 9.0 8.0 7.0 6.0 5.0 4.0 3.0 2.0 1.0 ppm
2.29
2.12
2.08
2.10
1.00
2.03

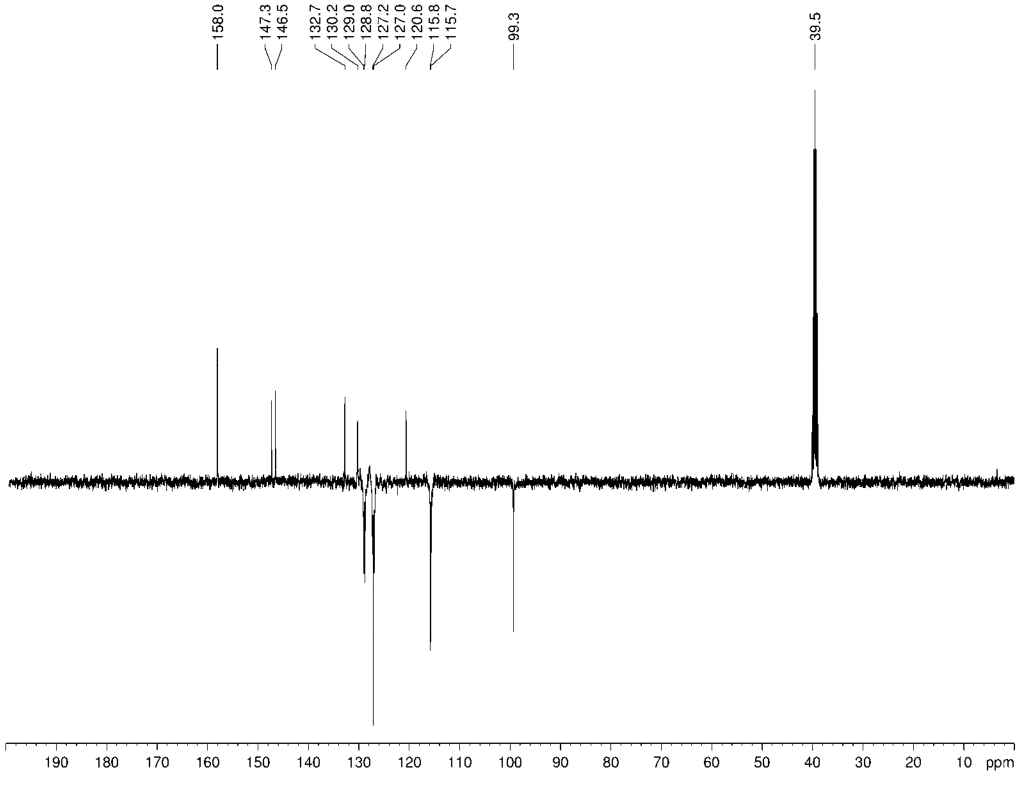

158.0
147.3
146.5
132.7
130.2
129.0
128.8
127.2
127.0
120.6
115.8
115.7
99.3
39.5
190 180 170 160 150 140 130 120 110 100 90 80 70 60 50 40 30 20 10 ppm

sery320B

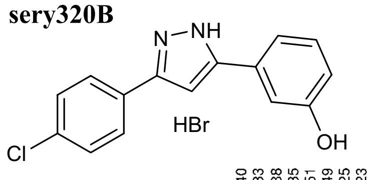
N
NH
HBr
Cl
OH

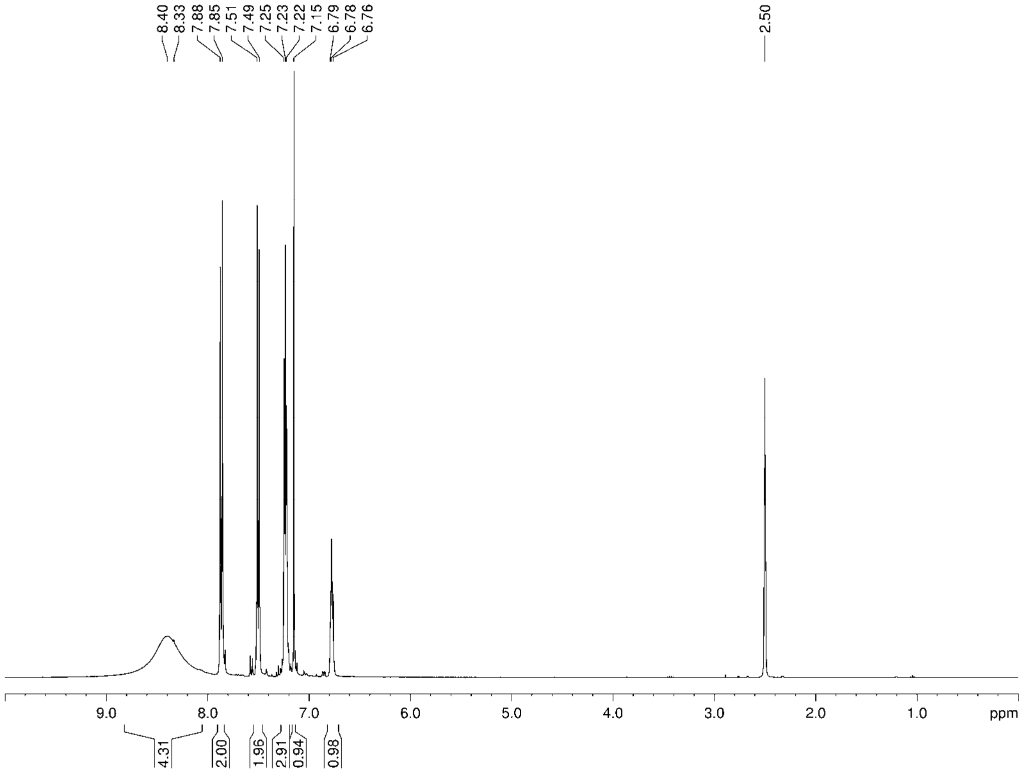
8.40 8.33 7.88 7.85 7.51 7.49 7.25 7.23 7.22 7.15 6.79 6.78 6.76
2.50
9.0 8.0 7.0 6.0 5.0 4.0 3.0 2.0 1.0 ppm
4.31 2.00 1.96 2.91 0.94 0.98

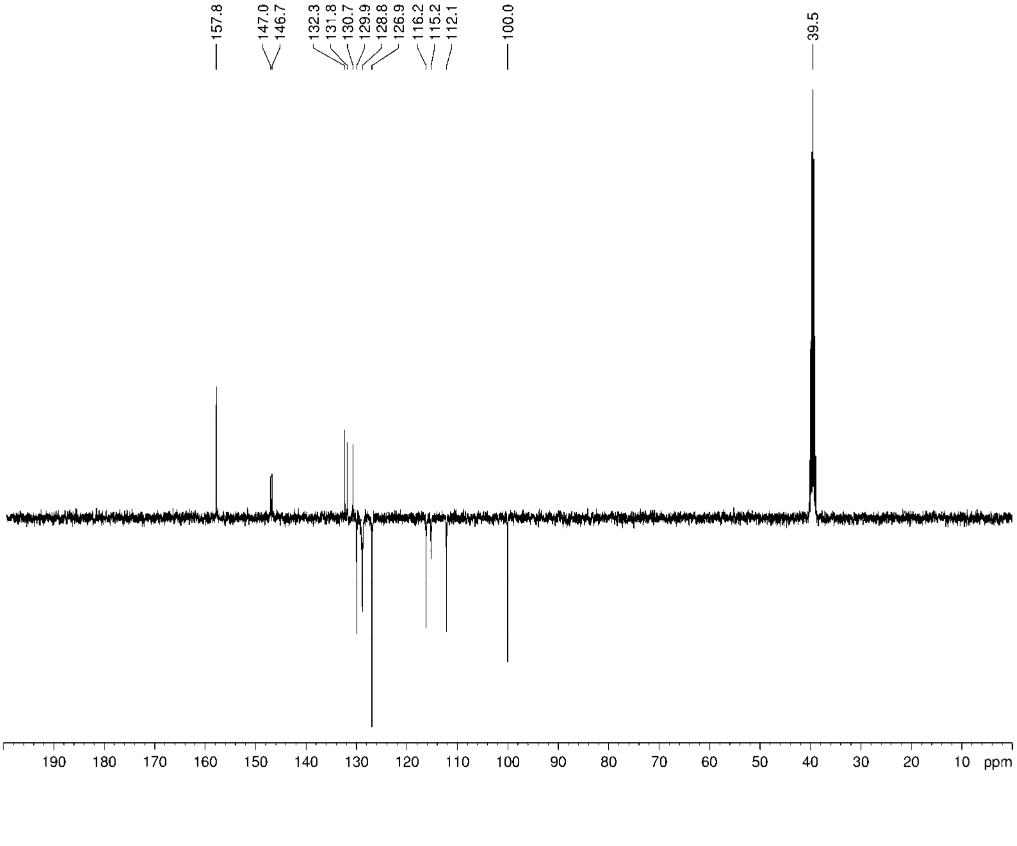
157.8
147.0 146.7
132.3 131.8 130.7 129.9 128.8 126.9 116.2 115.2 112.1
100.0
39.5
190 180 170 160 150 140 130 120 110 100 90 80 70 60 50 40 30 20 10 ppm

sery320C

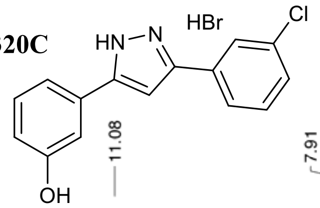

HBr
HN–N
Cl
OH

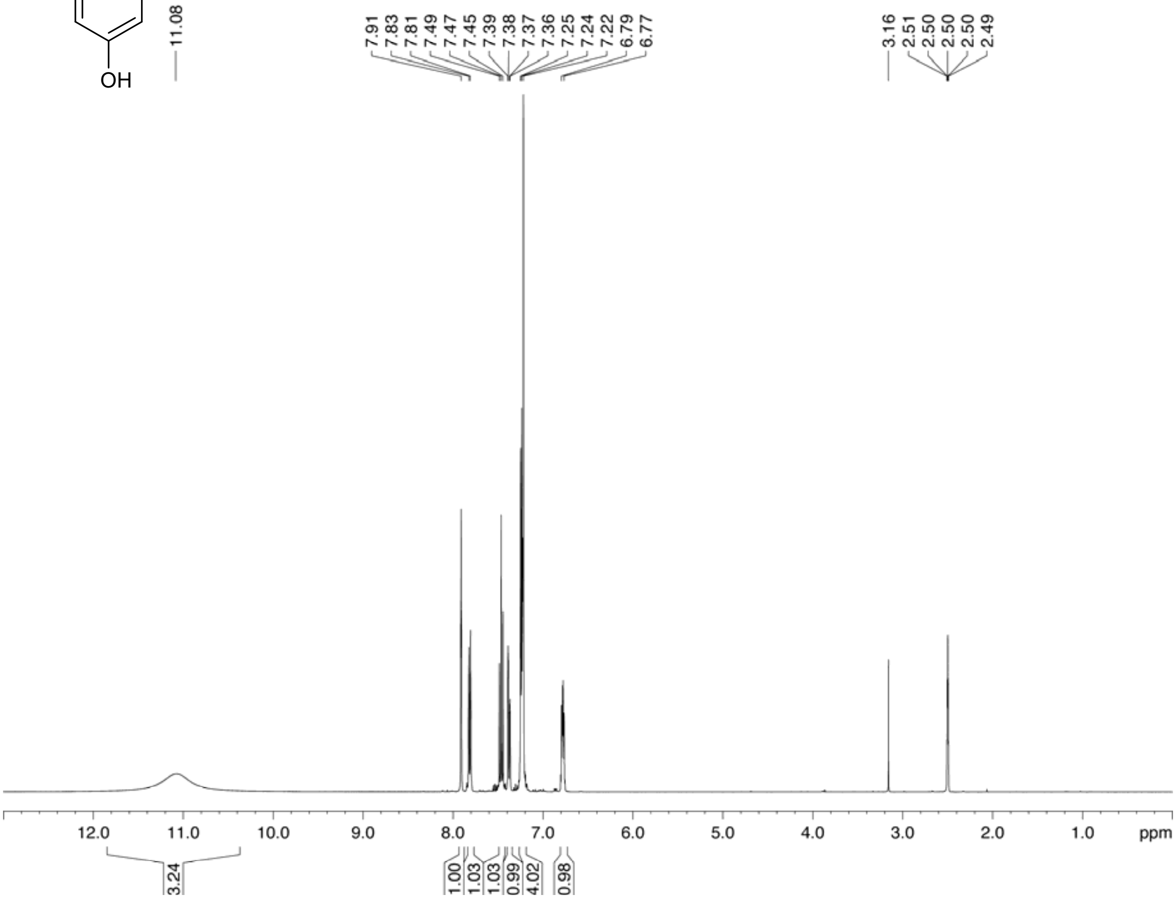

11.08
7.91 7.83 7.81 7.49 7.47 7.45 7.39 7.38 7.37 7.36 7.25 7.24 7.22 6.79 6.77
3.16 2.51 2.50 2.50 2.50 2.49
12.0 11.0 10.0 9.0 8.0 7.0 6.0 5.0 4.0 3.0 2.0 1.0 ppm
3.24
1.00 1.03 1.03 0.99 4.02 0.98

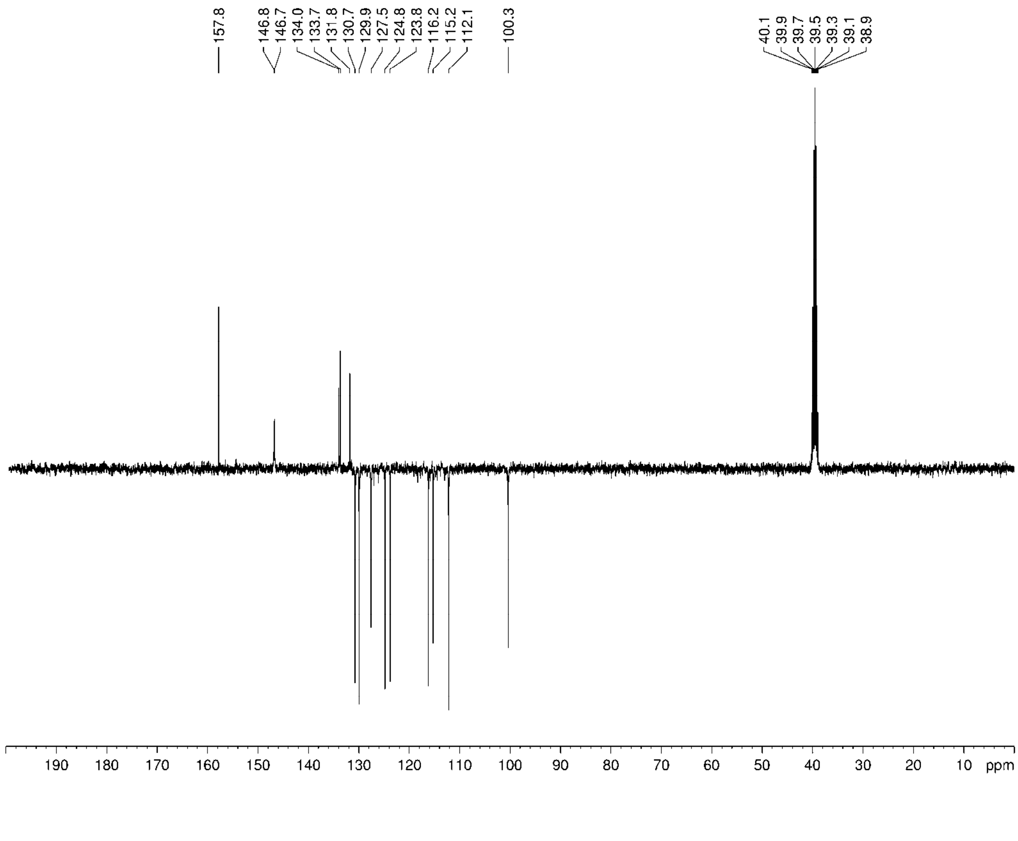

157.8
146.8 146.7 134.0 133.7 131.8 130.7 129.9 127.5 124.8 123.8 116.2 115.2 112.1
100.3
40.1 39.9 39.7 39.5 39.3 39.1 38.9
190 180 170 160 150 140 130 120 110 100 90 80 70 60 50 40 30 20 10 ppm

sery329

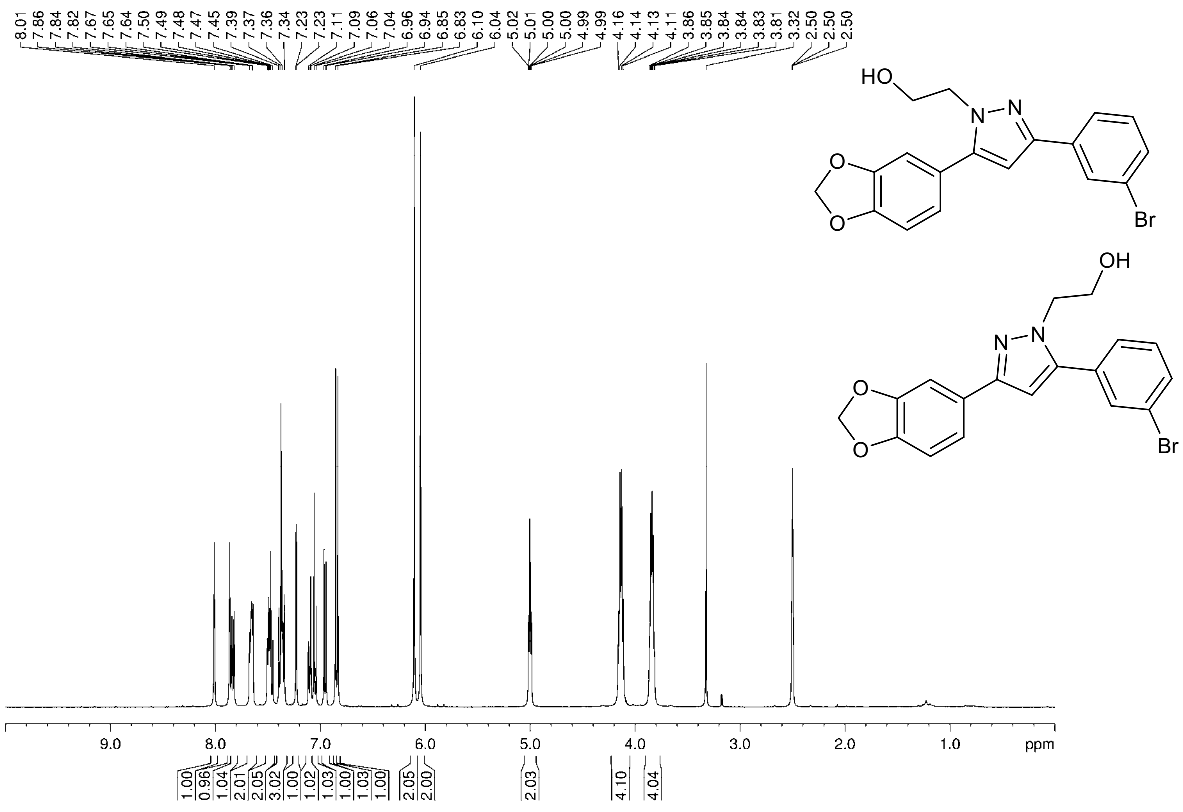
8.01 7.86 7.84 7.82 7.67 7.65 7.64 7.50 7.49 7.48 7.47 7.45 7.39 7.37 7.36 7.34 7.23 7.23 7.11 7.09 7.06 7.04 6.96 6.94 6.85 6.83 6.10 6.04 5.02 5.01 5.00 5.00 4.99 4.16 4.14 4.13 4.11 3.86 3.85 3.84 3.84 3.83 3.81 3.32 2.50 2.50 2.50
HO
N
N
O
O
Br
OH
N
N
O
O
Br
9.0 8.0 7.0 6.0 5.0 4.0 3.0 2.0 1.0 ppm
1.00 0.96 1.04 2.01 2.05 3.02 1.00 1.02 1.03 1.00 1.03 1.00 2.05 2.00 2.03 4.10 4.04

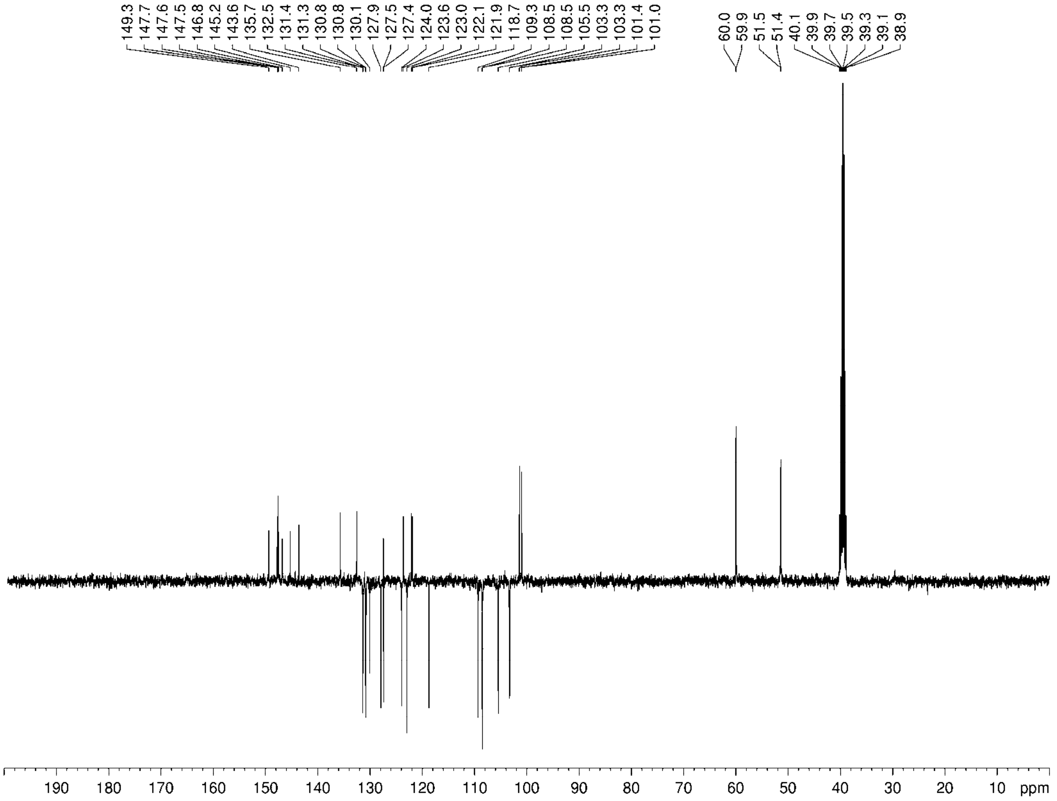
149.3 147.7 147.6 147.5 146.8 145.2 143.6 135.7 132.5 131.4 131.3 130.8 130.8 130.1 127.9 127.5 127.4 124.0 123.6 123.0 122.1 121.9 118.7 109.3 108.5 108.5 105.5 103.3 103.3 101.4 101.0
60.0 59.9 51.5 51.4 40.1 39.9 39.7 39.5 39.3 39.1 38.9
190 180 170 160 150 140 130 120 110 100 90 80 70 60 50 40 30 20 10 ppm

sery330

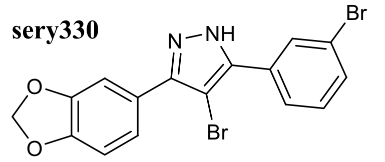
N
NH
Br
Br
O
O

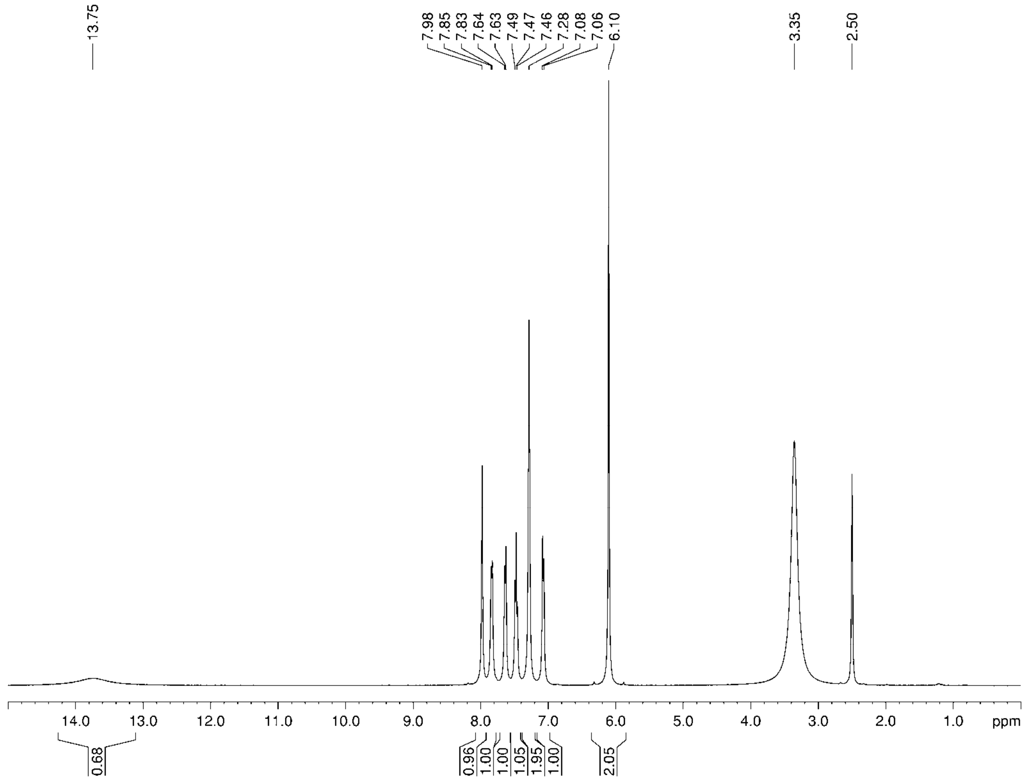
13.75
7.98
7.85
7.83
7.64
7.63
7.49
7.47
7.46
7.28
7.08
7.06
6.10
3.35
2.50
14.0 13.0 12.0 11.0 10.0 9.0 8.0 7.0 6.0 5.0 4.0 3.0 2.0 1.0 ppm
0.68
0.96
1.00
1.00
1.05
1.95
1.00
2.05

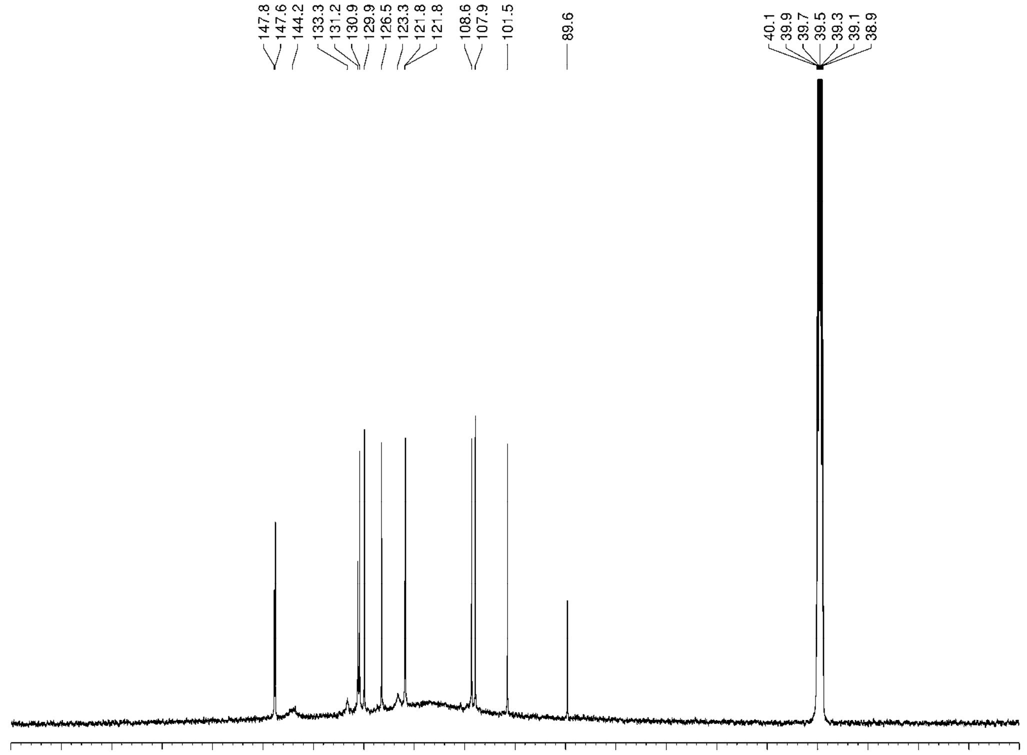
147.8
147.6
144.2
133.3
131.2
130.9
129.9
126.5
123.3
121.8
121.8
108.6
107.9
101.5
89.6
40.1
39.9
39.7
39.5
39.3
39.1
38.9
190 180 170 160 150 140 130 120 110 100 90 80 70 60 50 40 30 20 10 ppm

sery336B

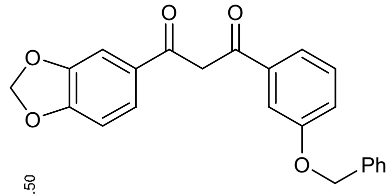
O
O
O
O
O
Ph

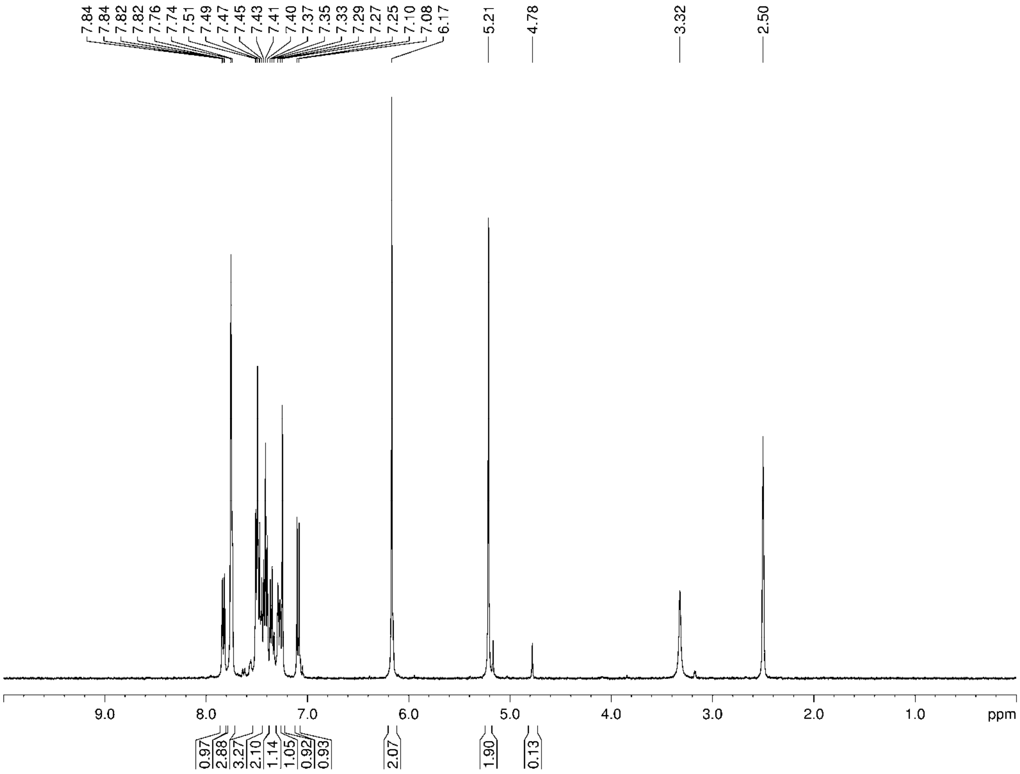
7.84
7.84
7.82
7.82
7.76
7.74
7.51
7.49
7.47
7.45
7.43
7.41
7.40
7.37
7.35
7.33
7.29
7.27
7.25
7.10
7.08
6.17
5.21
4.78
3.32
2.50
9.0
8.0
7.0
6.0
5.0
4.0
3.0
2.0
1.0
ppm
0.97
2.88
3.27
2.10
1.14
1.05
0.92
0.93
2.07
1.90
0.13

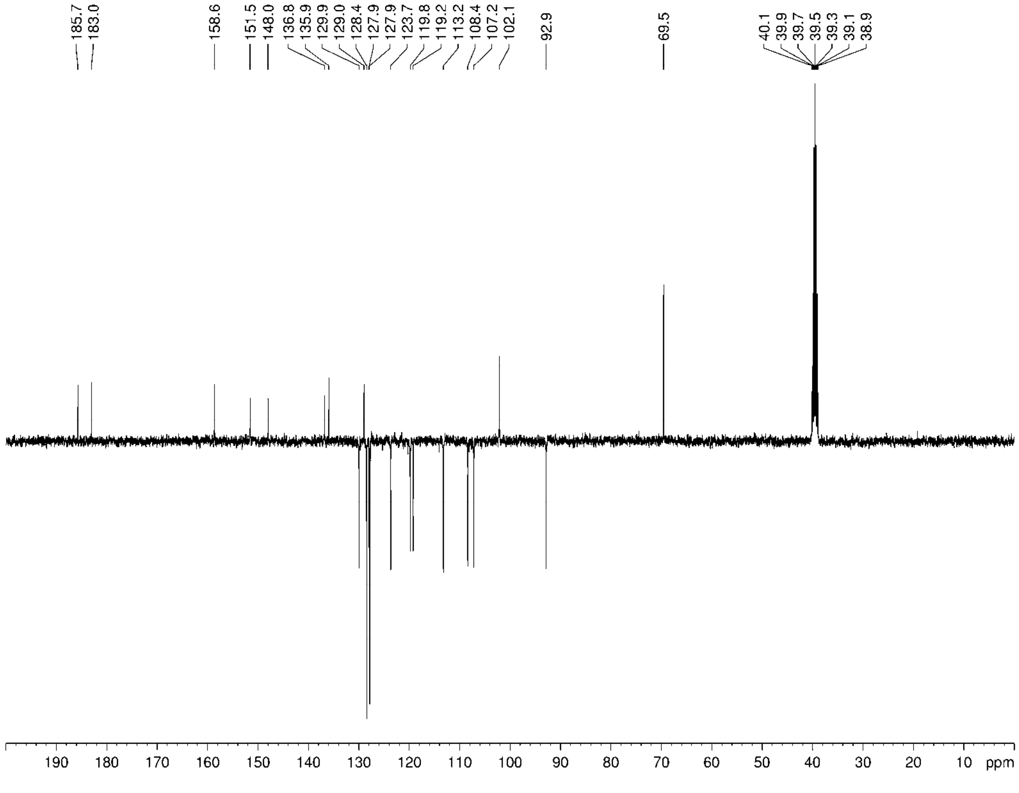
185.7
183.0
158.6
151.5
148.0
136.8
135.9
129.9
129.0
128.4
127.9
127.9
123.7
119.8
119.2
113.2
108.4
107.2
102.1
92.9
69.5
40.1
39.9
39.7
39.5
39.3
39.1
38.9
190
180
170
160
150
140
130
120
110
100
90
80
70
60
50
40
30
20
10
ppm

sery338A

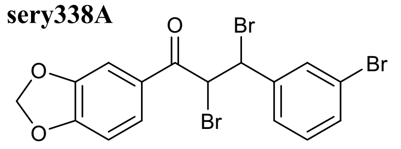
O
Br
Br
Br
O
O

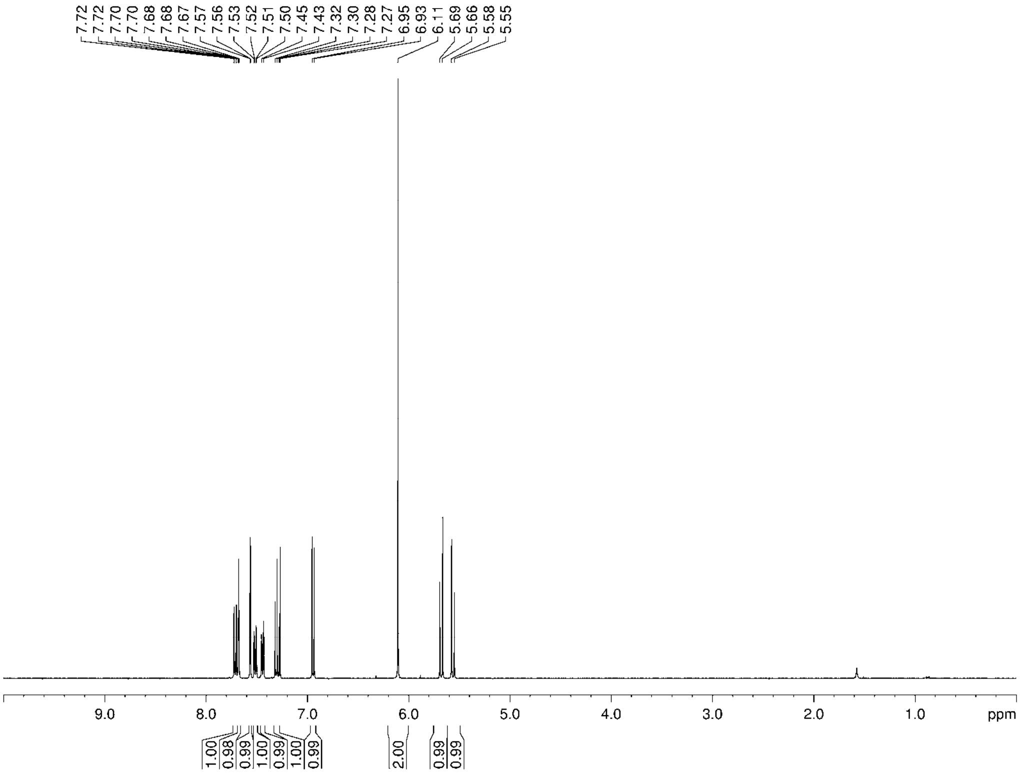
7.72
7.72
7.70
7.70
7.68
7.68
7.67
7.57
7.56
7.53
7.52
7.51
7.50
7.45
7.43
7.32
7.30
7.28
7.27
6.95
6.93
6.11
5.69
5.66
5.58
5.55
9.0
8.0
7.0
6.0
5.0
4.0
3.0
2.0
1.0
ppm
1.00
0.98
0.99
1.00
0.99
1.00
0.99
2.00
0.99
0.99

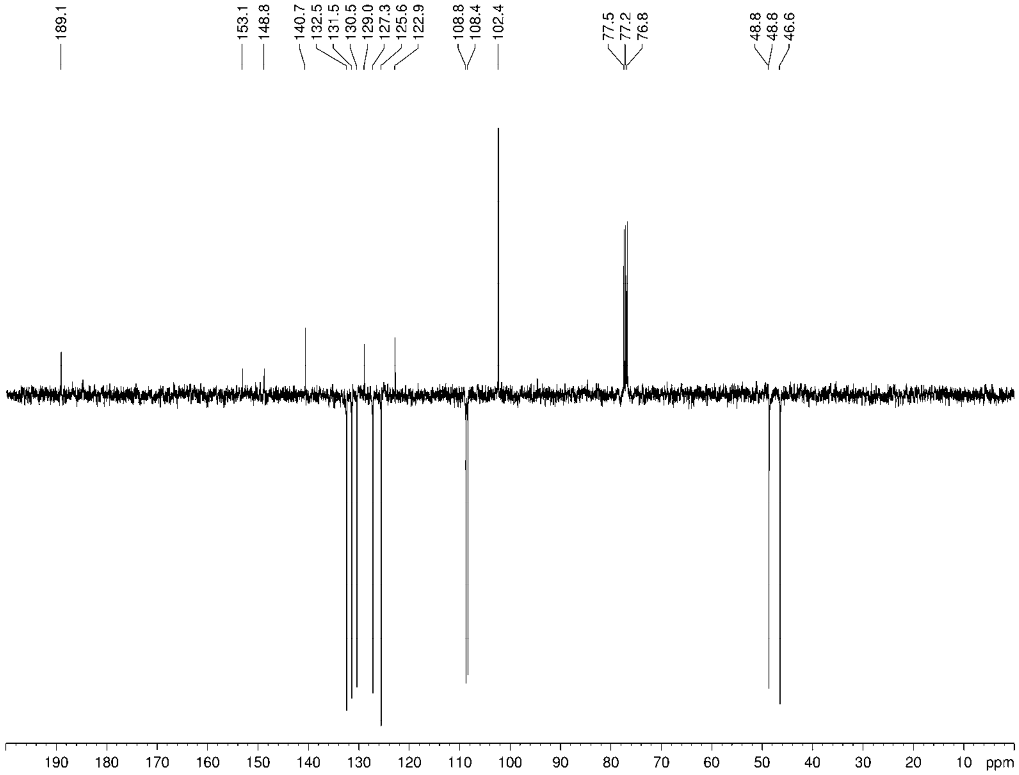
189.1
153.1
148.8
140.7
132.5
131.5
130.5
129.0
127.3
125.6
122.9
108.8
108.4
102.4
77.5
77.2
76.8
48.8
48.8
46.6
190
180
170
160
150
140
130
120
110
100
90
80
70
60
50
40
30
20
10
ppm

sery338B

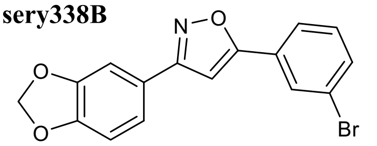
N
O
O
O
Br

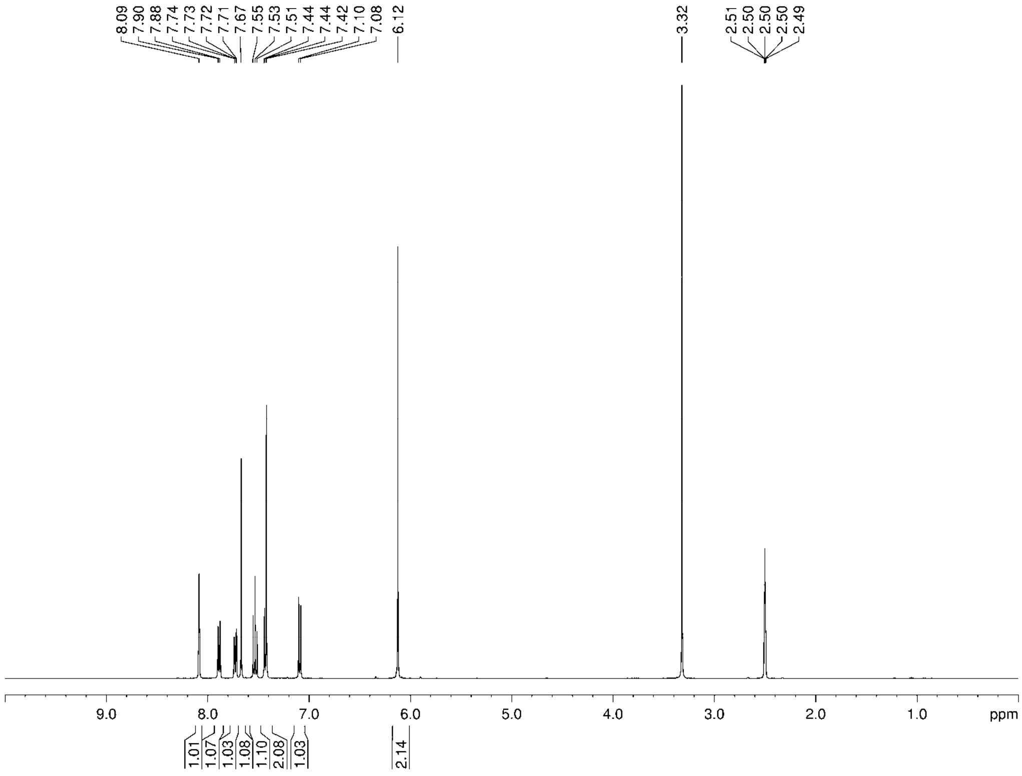
8.09
7.90
7.88
7.74
7.73
7.72
7.71
7.67
7.55
7.53
7.51
7.44
7.44
7.42
7.10
7.08
6.12
3.32
2.51
2.50
2.50
2.50
2.49
9.0
8.0
7.0
6.0
5.0
4.0
3.0
2.0
1.0
ppm
1.01
1.07
1.03
1.08
1.10
2.08
1.03
2.14

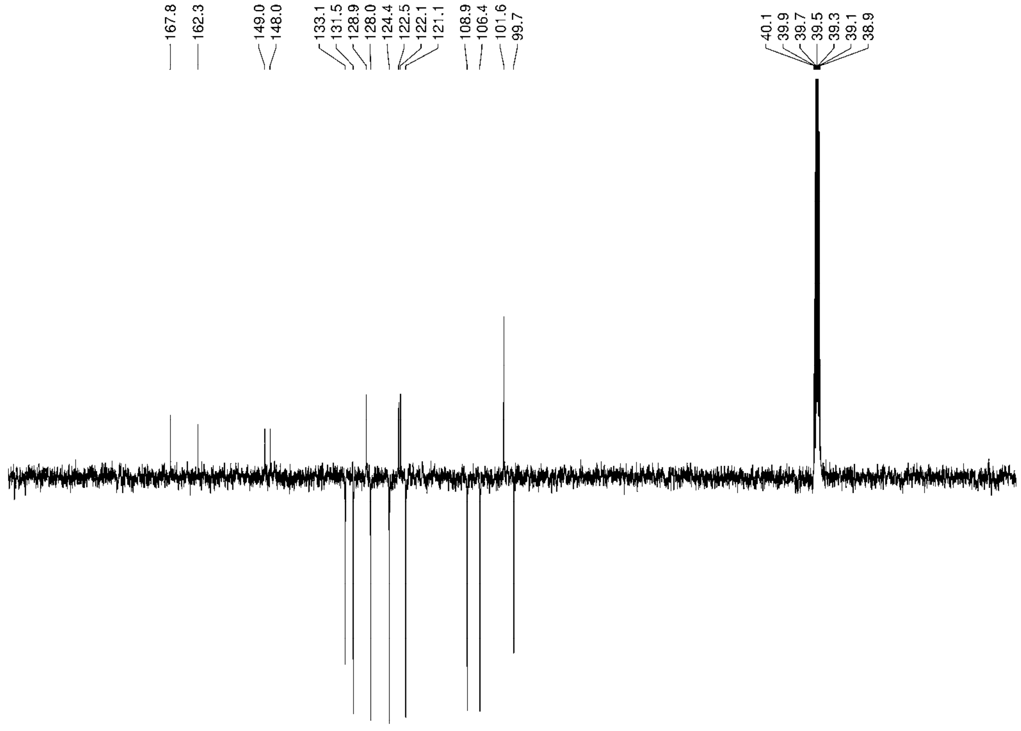
167.8
162.3
149.0
148.0
133.1
131.5
128.9
128.0
124.4
122.5
122.1
121.1
108.9
106.4
101.6
99.7
40.1
39.9
39.7
39.5
39.3
39.1
38.9
190
180
170
160
150
140
130
120
110
100
90
80
70
60
50
40
30
20
10
ppm

sery339A

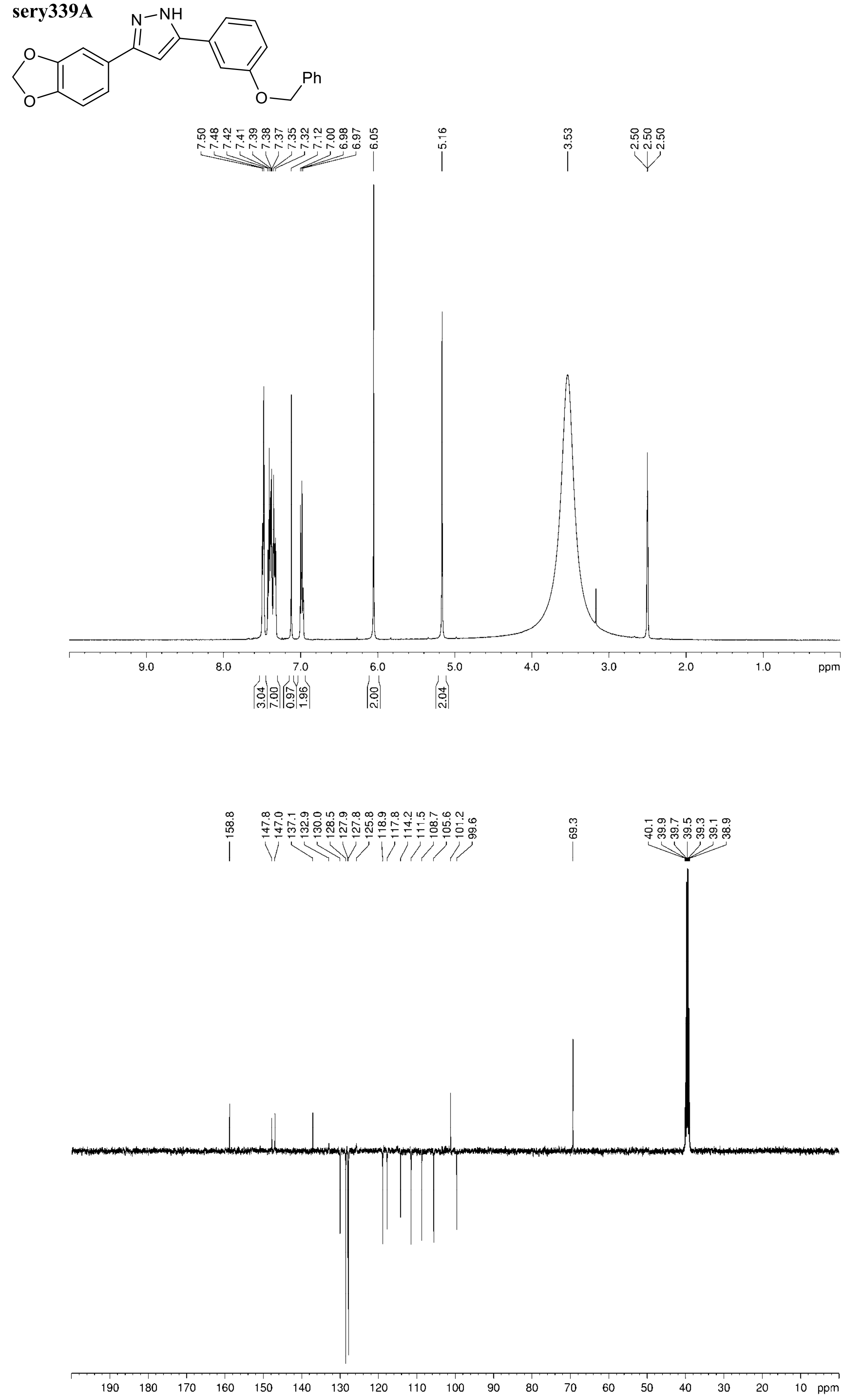
N–NH
O
O
O
Ph
7.50
7.48
7.42
7.41
7.39
7.38
7.37
7.35
7.32
7.12
7.00
6.98
6.97
6.05
5.16
3.53
2.50
2.50
2.50
9.0
8.0
7.0
6.0
5.0
4.0
3.0
2.0
1.0
ppm
3.04
7.00
0.97
1.96
2.00
2.04
158.8
147.8
147.0
137.1
132.9
130.0
128.5
127.9
127.8
125.8
118.9
117.8
114.2
111.5
108.7
105.6
101.2
99.6
69.3
40.1
39.9
39.7
39.5
39.3
39.1
38.9
190
180
170
160
150
140
130
120
110
100
90
80
70
60
50
40
30
20
10
ppm

sery342A

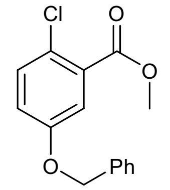
Cl
O
O
O
Ph

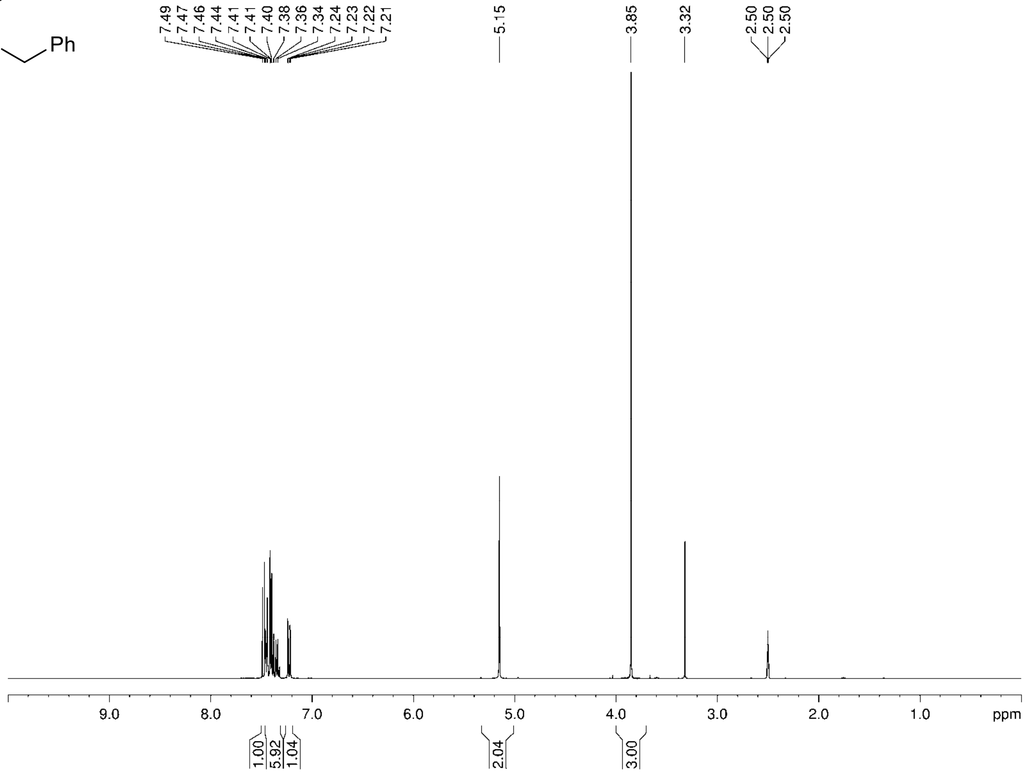
7.49
7.47
7.46
7.44
7.41
7.41
7.40
7.38
7.36
7.34
7.24
7.23
7.22
7.21
5.15
3.85
3.32
2.50
2.50
2.50
9.0
8.0
7.0
6.0
5.0
4.0
3.0
2.0
1.0
ppm
1.00
5.92
1.04
2.04
3.00

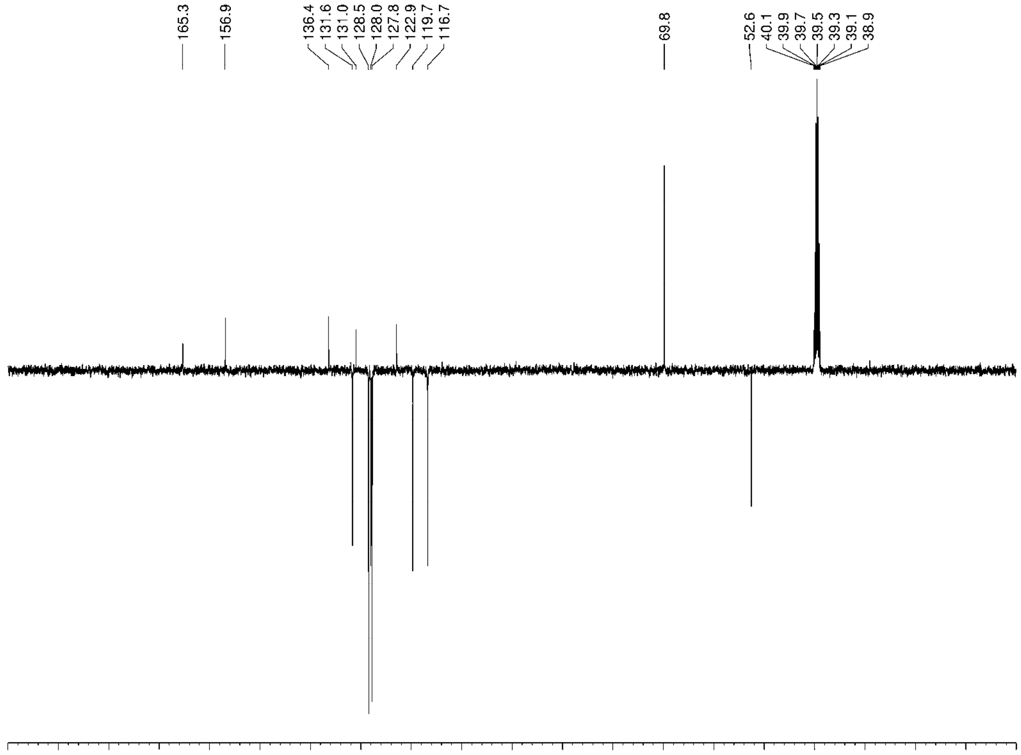
165.3
156.9
136.4
131.6
131.0
128.5
128.0
127.8
122.9
119.7
116.7
69.8
52.6
40.1
39.9
39.7
39.5
39.3
39.1
38.9
190
180
170
160
150
140
130
120
110
100
90
80
70
60
50
40
30
20
10
ppm

sery342B

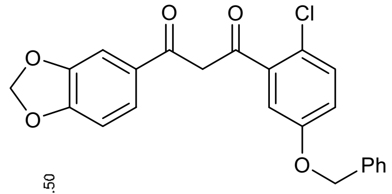

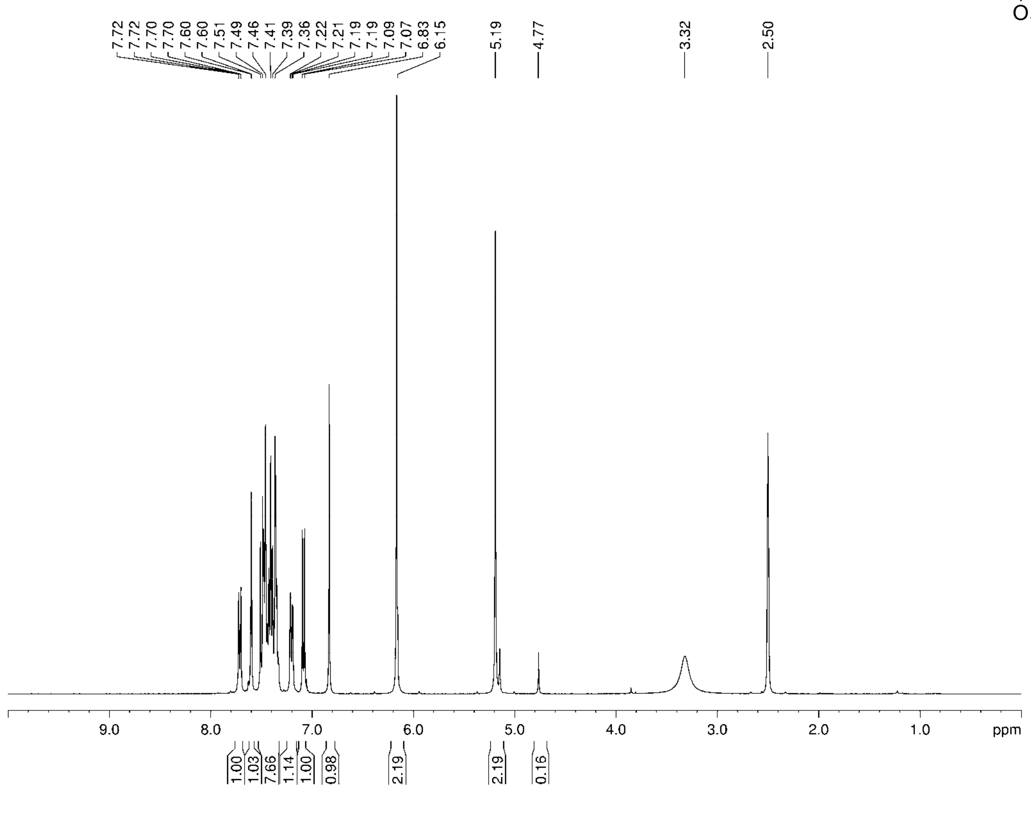

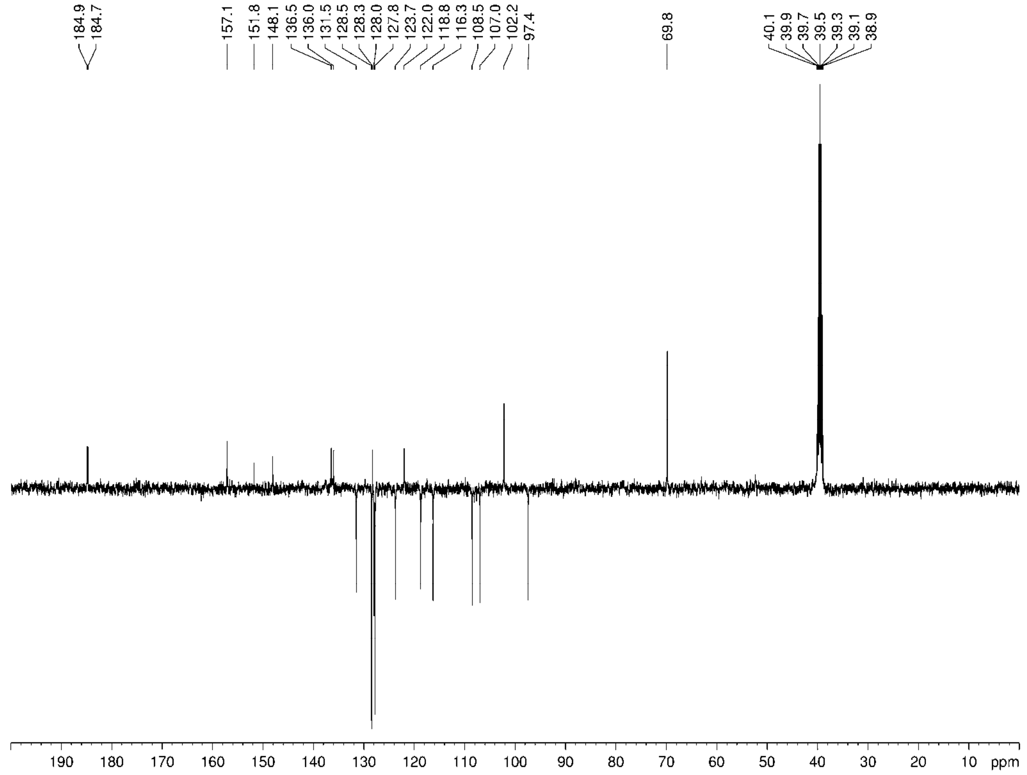

sery342C

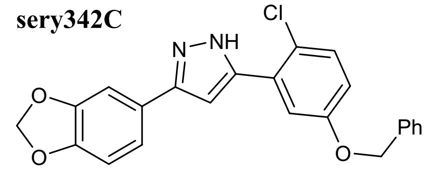

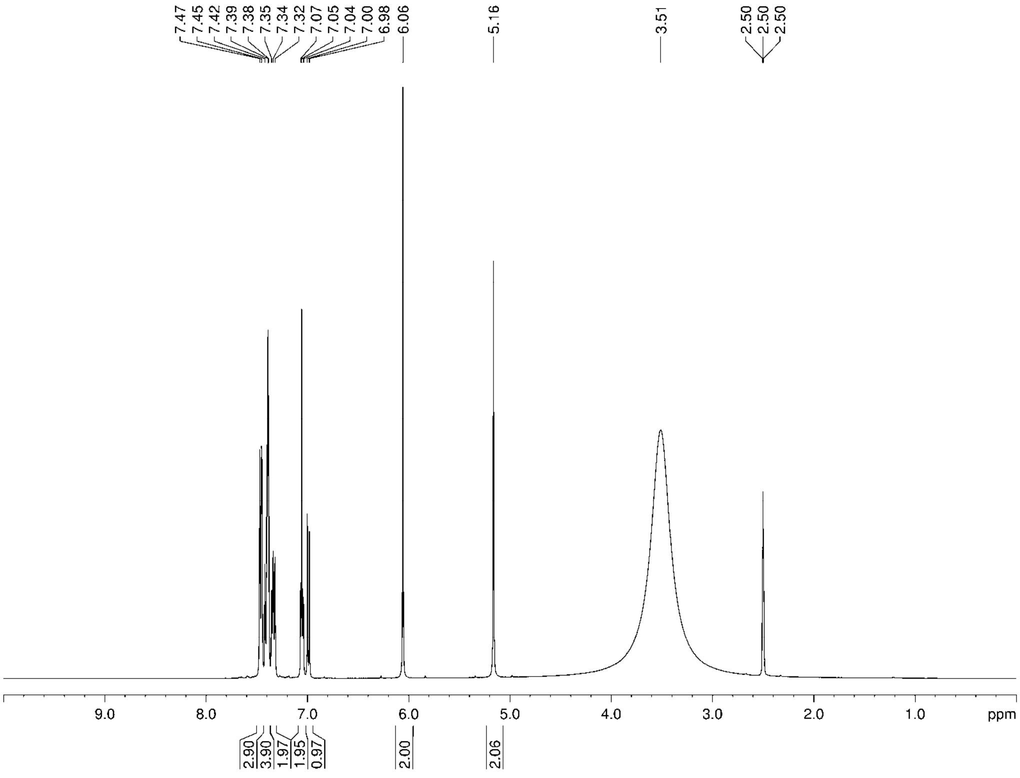

7.47
7.45
7.42
7.39
7.38
7.35
7.34
7.32
7.07
7.05
7.04
7.00
6.98
6.06
5.16
3.51
2.50
2.50
2.50
9.0
8.0
7.0
6.0
5.0
4.0
3.0
2.0
1.0
ppm
2.90
3.90
1.97
1.95
0.97
2.00
2.06

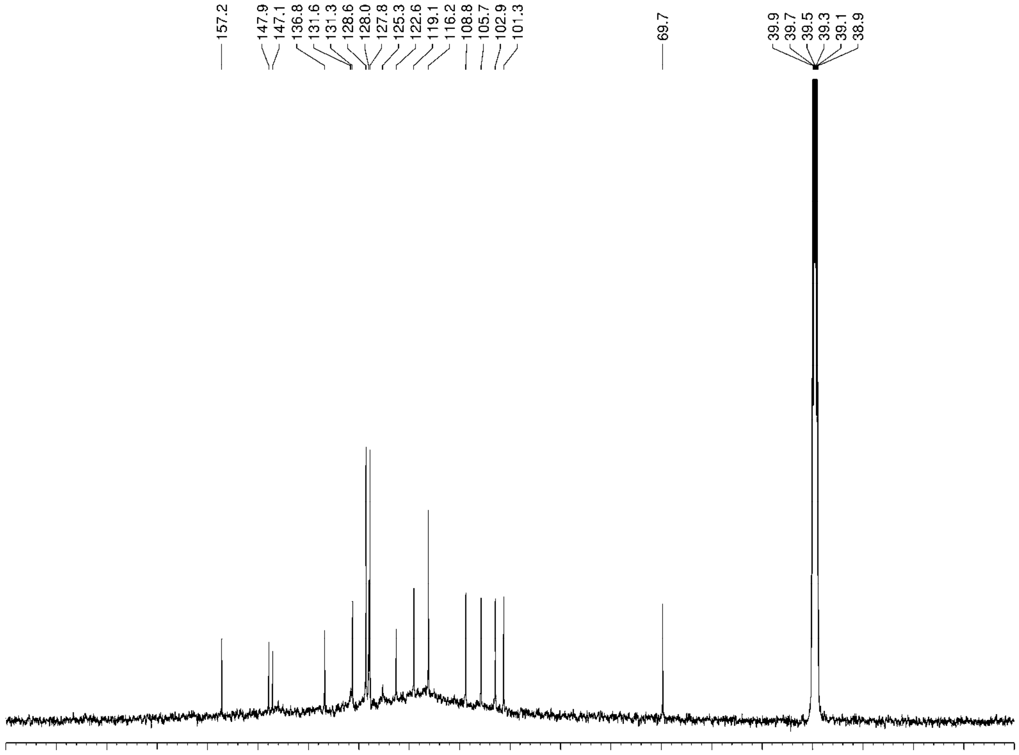

157.2
147.9
147.1
136.8
131.6
131.3
128.6
128.0
127.8
125.3
122.6
119.1
116.2
108.8
105.7
102.9
101.3
69.7
39.9
39.7
39.5
39.3
39.1
38.9
190
180
170
160
150
140
130
120
110
100
90
80
70
60
50
40
30
20
10
ppm

sery344

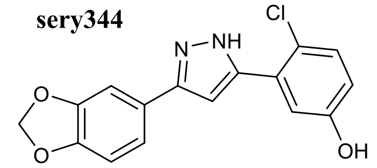
Cl
N
NH
O
O
OH

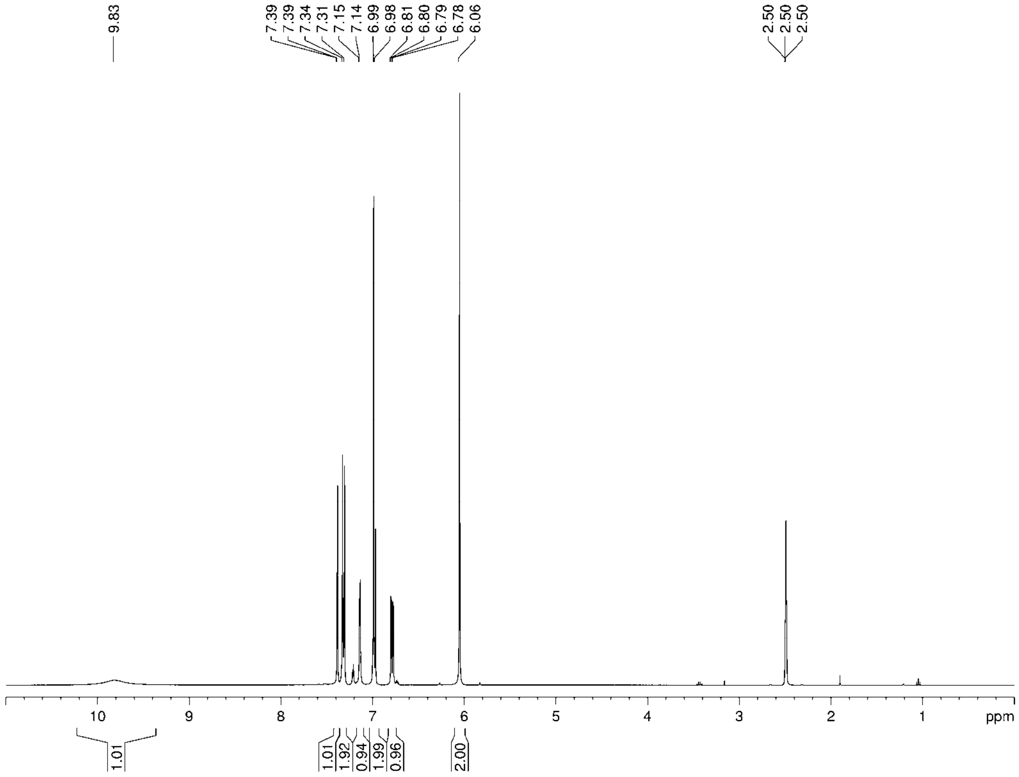
9.83
7.39
7.39
7.34
7.31
7.15
7.14
6.99
6.98
6.81
6.80
6.79
6.78
6.06
2.50
2.50
2.50
10
9
8
7
6
5
4
3
2
1
ppm
1.01
1.01
1.92
0.94
1.99
0.96
2.00

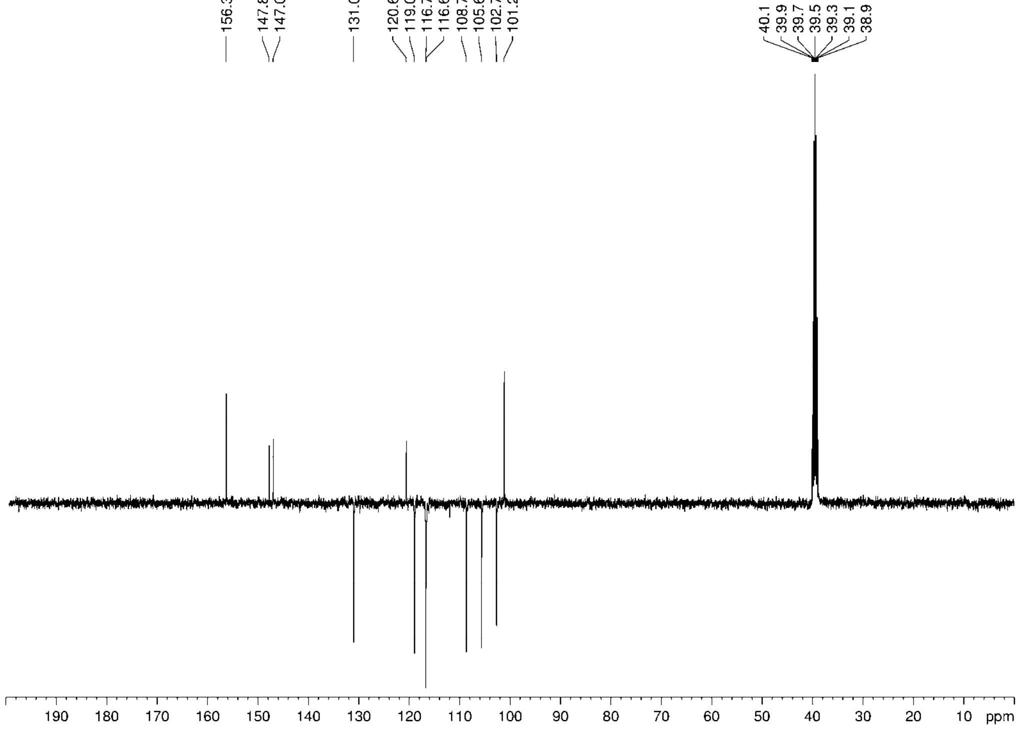
156.3
147.8
147.0
131.0
120.6
119.0
116.7
116.6
108.7
105.6
102.7
101.2
40.1
39.9
39.7
39.5
39.3
39.1
38.9
190
180
170
160
150
140
130
120
110
100
90
80
70
60
50
40
30
20
10
ppm

sery345

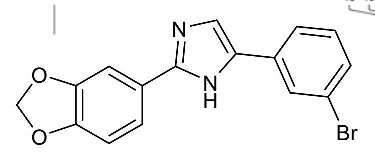

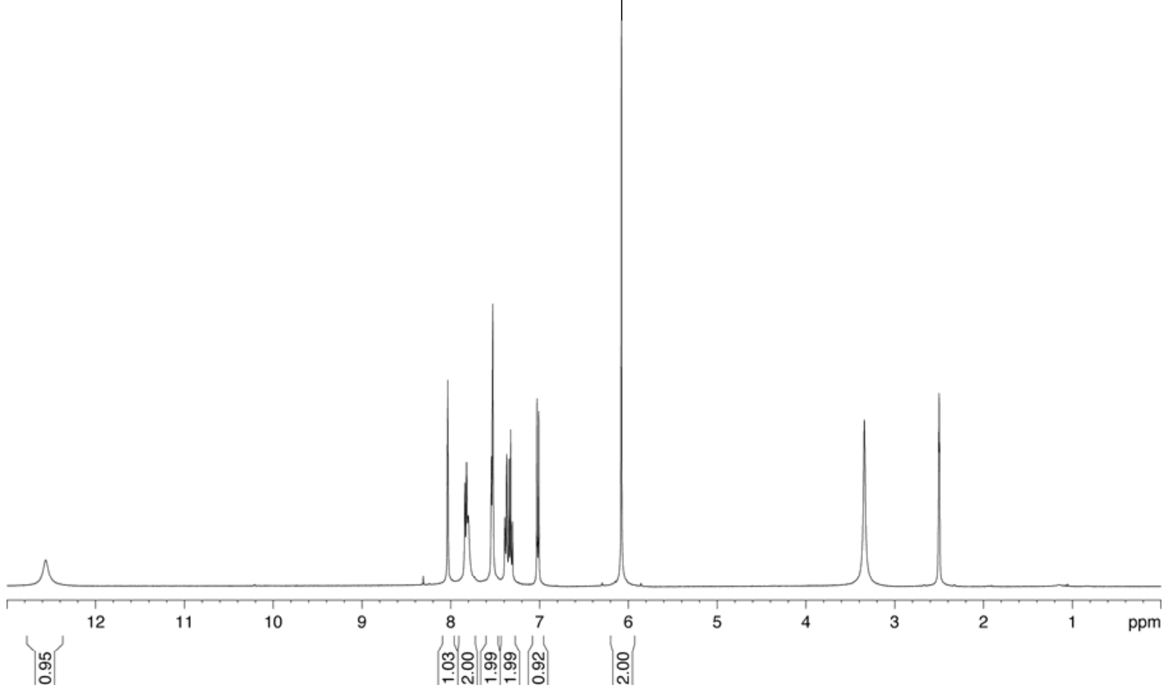
12.57
8.04
8.03
7.84
7.82
7.80
7.54
7.53
7.53
7.39
7.37
7.34
7.33
7.31
7.03
7.02
7.02
7.01
6.08
3.34
2.50
2.50
2.50
1.98
1.17
12
11
10
9
8
7
6
5
4
3
2
1
ppm
0.95
1.03
2.00
1.99
1.99
0.92
2.00

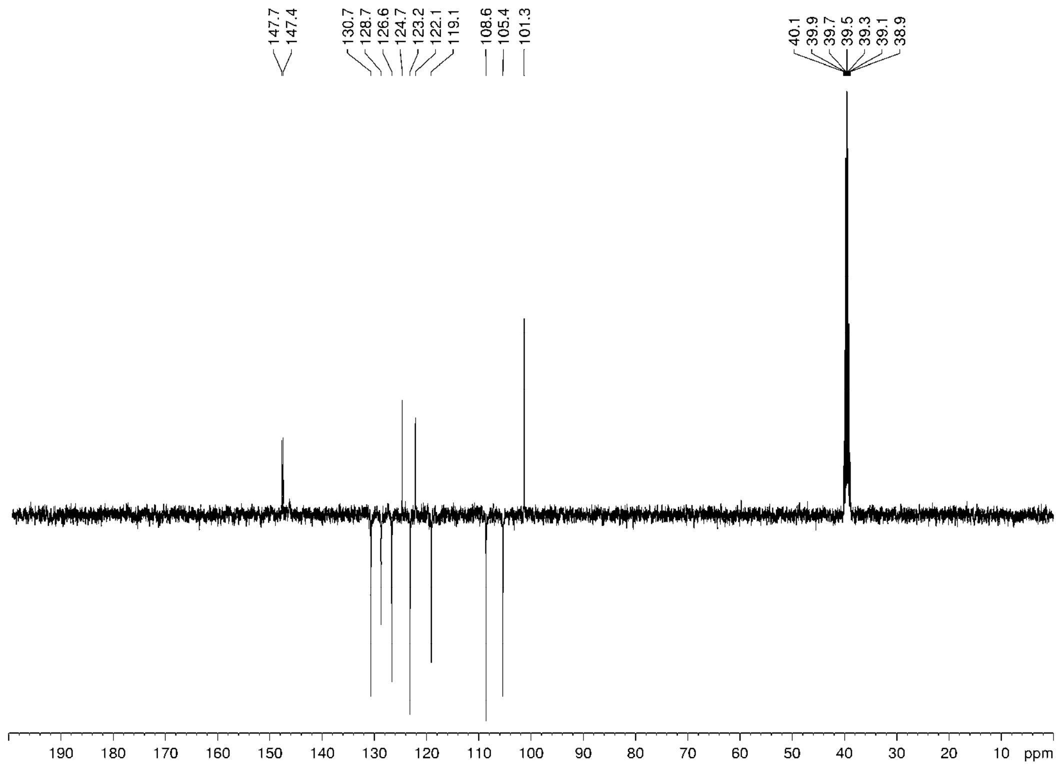
147.7
147.4
130.7
128.7
126.6
124.7
123.2
122.1
119.1
108.6
105.4
101.3
40.1
39.9
39.7
39.5
39.3
39.1
38.9
190
180
170
160
150
140
130
120
110
100
90
80
70
60
50
40
30
20
10
ppm

sery363A

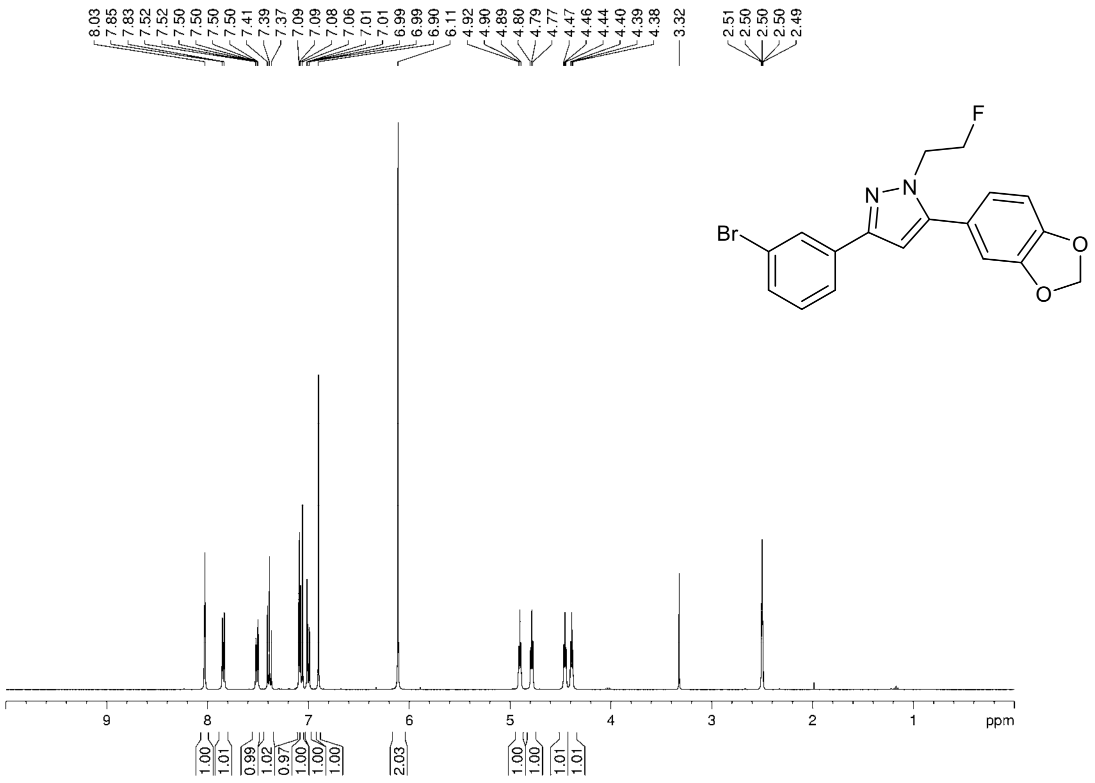

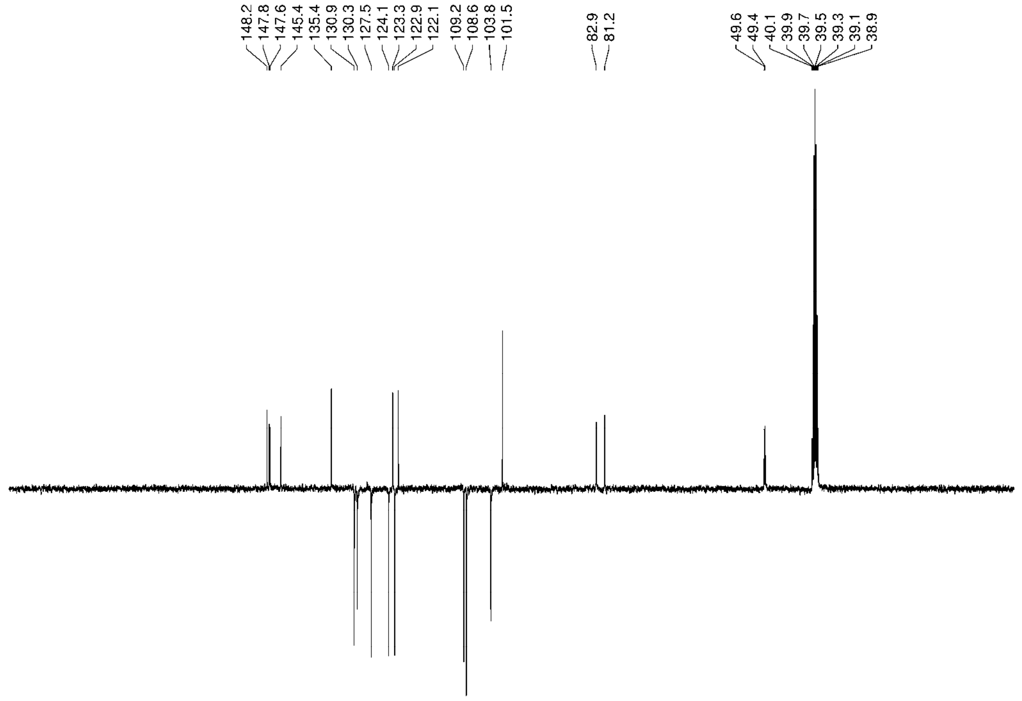

sery363B

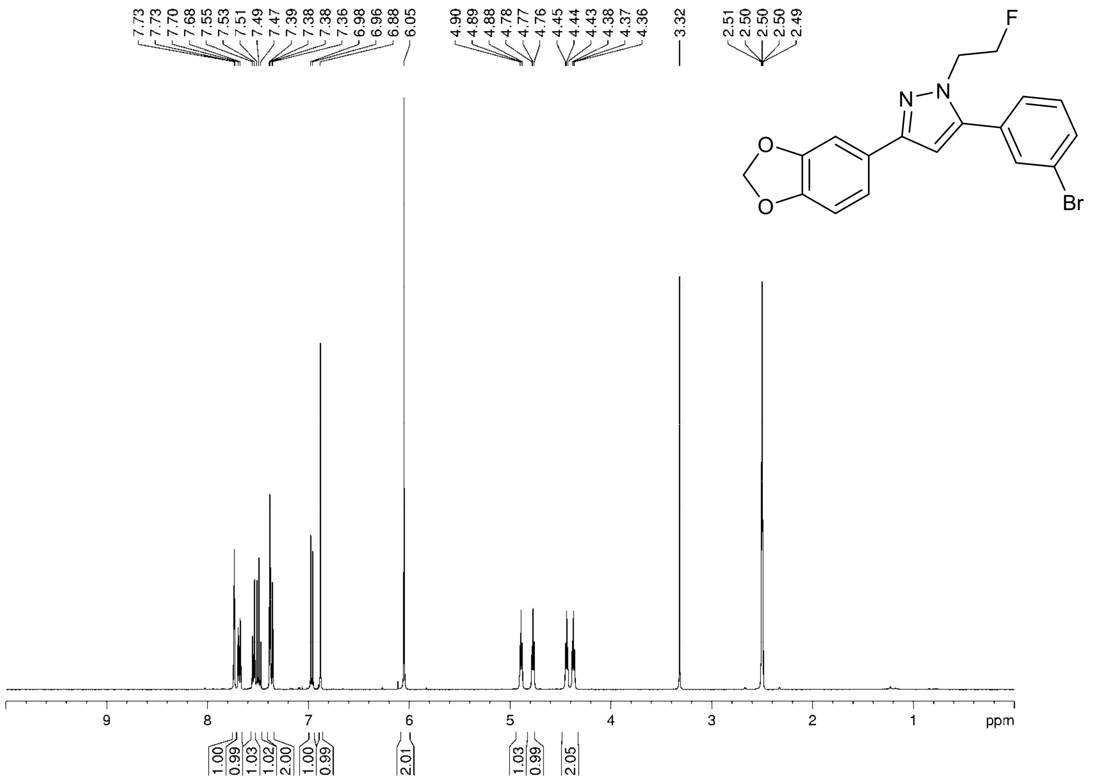

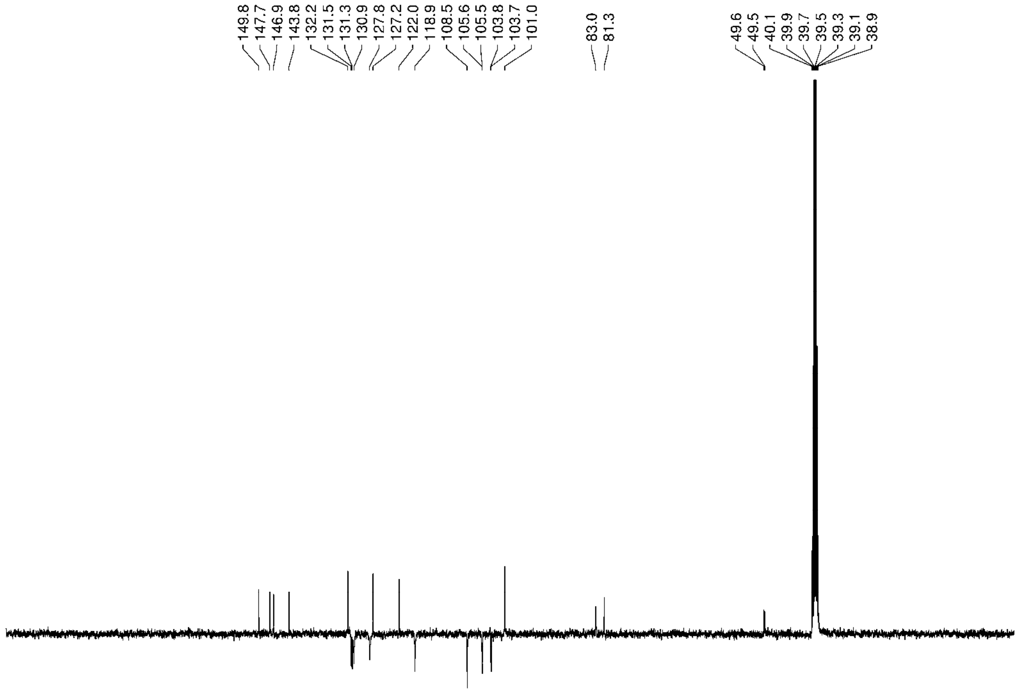

sery369

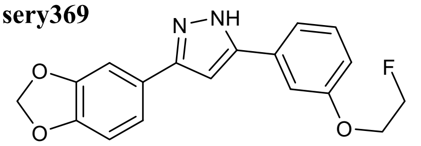
N–NH
O
O
O
F

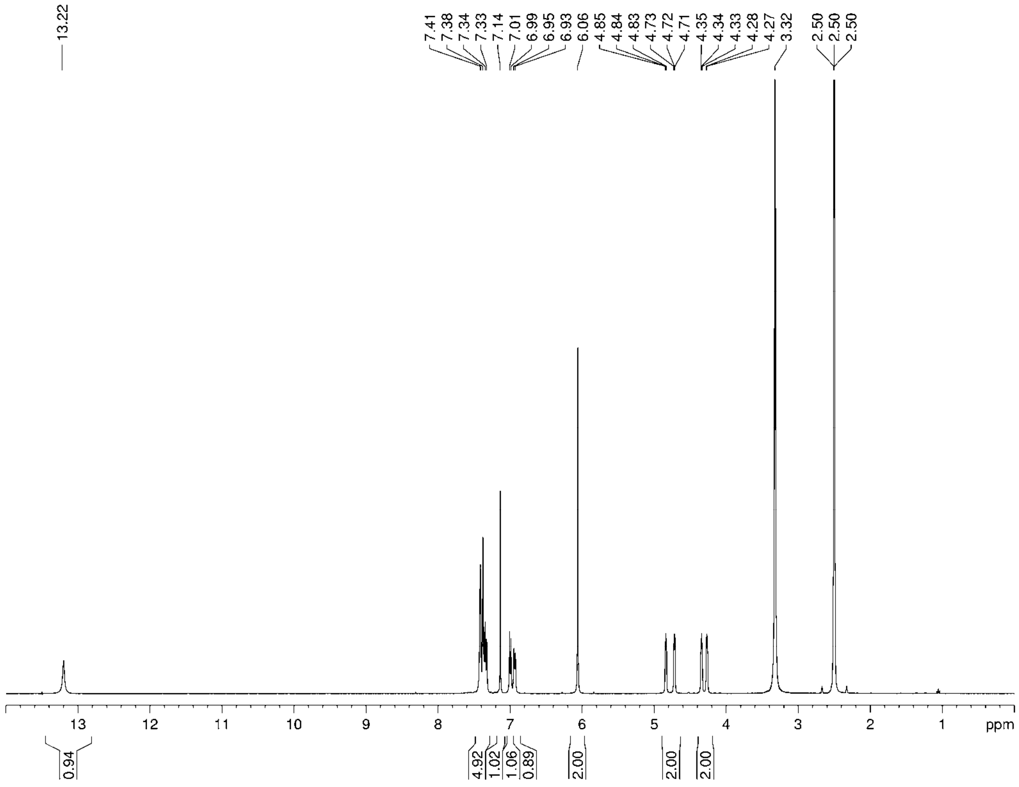
13.22
7.41 7.38 7.34 7.33 7.14 7.01 6.99 6.95 6.93 6.06 4.85 4.84 4.83 4.73 4.72 4.71 4.35 4.34 4.33 4.28 4.27 3.32 2.50 2.50 2.50
13 12 11 10 9 8 7 6 5 4 3 2 1 ppm
0.94
4.92 1.02 1.06 0.89
2.00
2.00
2.00

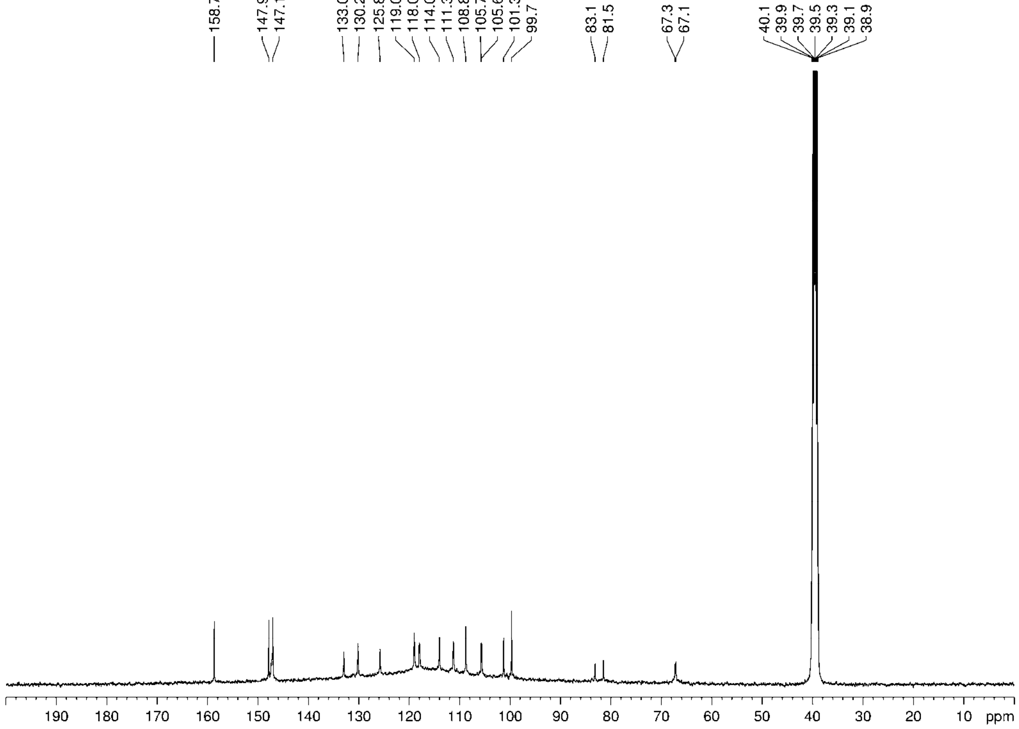
158.7
147.9 147.1
133.0 130.2 125.8 119.0 118.0 114.0 111.3 108.8 105.7 105.6 101.3 99.7
83.1 81.5
67.3 67.1
40.1 39.9 39.7 39.5 39.3 39.1 38.9
190 180 170 160 150 140 130 120 110 100 90 80 70 60 50 40 30 20 10 ppm

sery381A

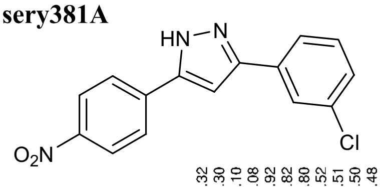
HN–N
O_2N
Cl

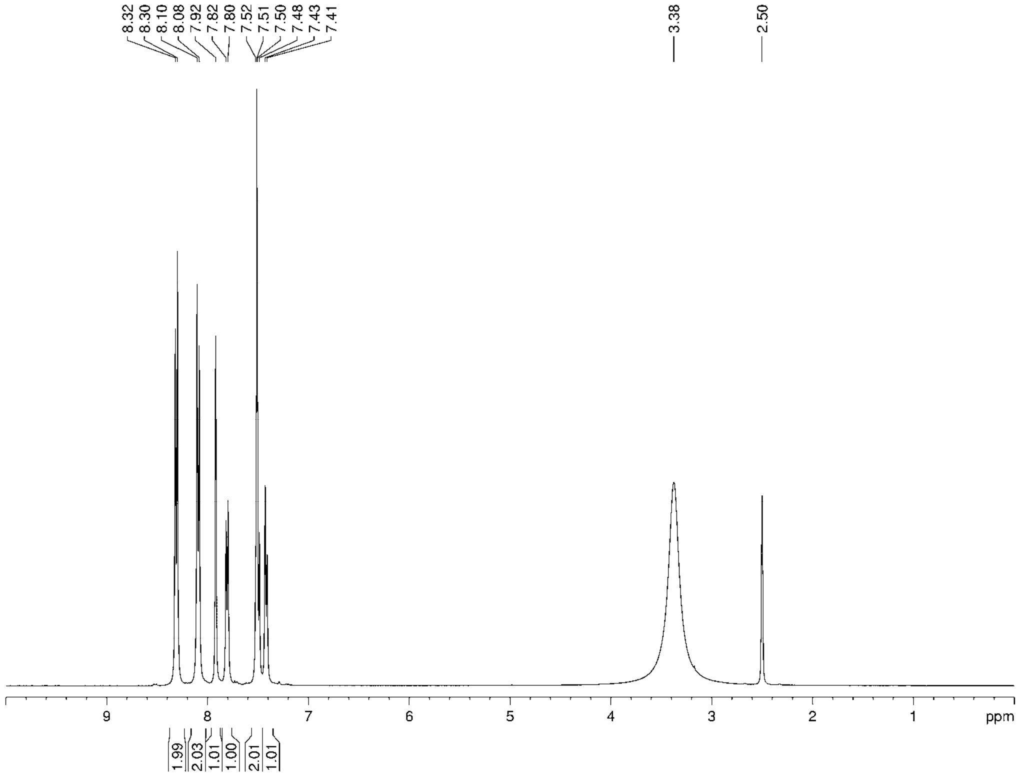
8.32
8.30
8.10
8.08
7.92
7.82
7.80
7.52
7.51
7.50
7.48
7.43
7.41
3.38
2.50
9
8
7
6
5
4
3
2
1
ppm
1.99
2.03
1.01
1.00
2.01
1.01

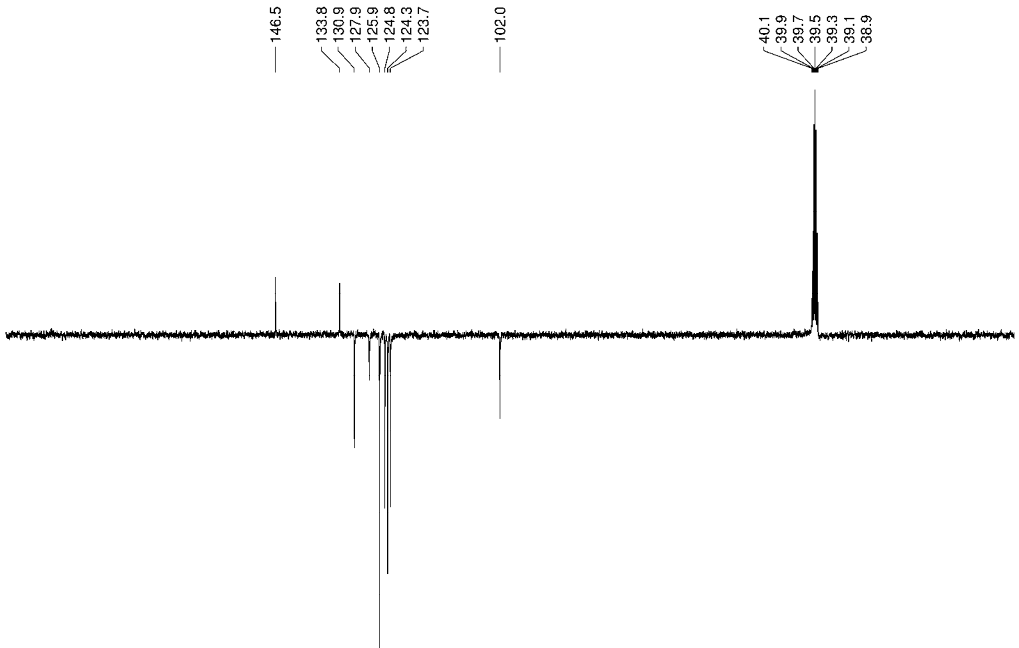
146.5
133.8
130.9
127.9
125.9
124.8
124.3
123.7
102.0
40.1
39.9
39.7
39.5
39.3
39.1
38.9
190
180
170
160
150
140
130
120
110
100
90
80
70
60
50
40
30
20
10
ppm

sery381B

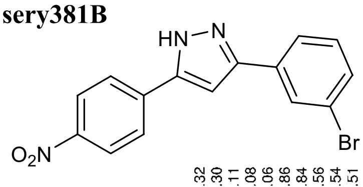
HN–N
Br
O2N

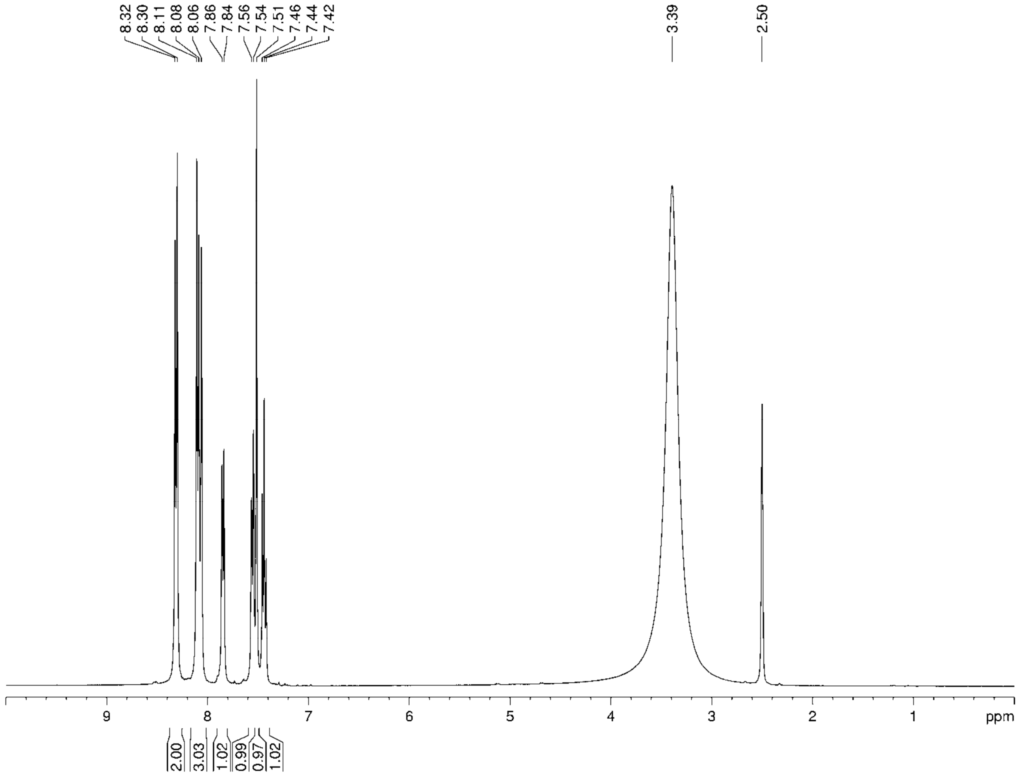
8.32 8.30 8.11 8.08 8.06 7.86 7.84 7.56 7.54 7.51 7.46 7.44 7.42
3.39
2.50
9 8 7 6 5 4 3 2 1 ppm
2.00 3.03 1.02 0.99 0.97 1.02

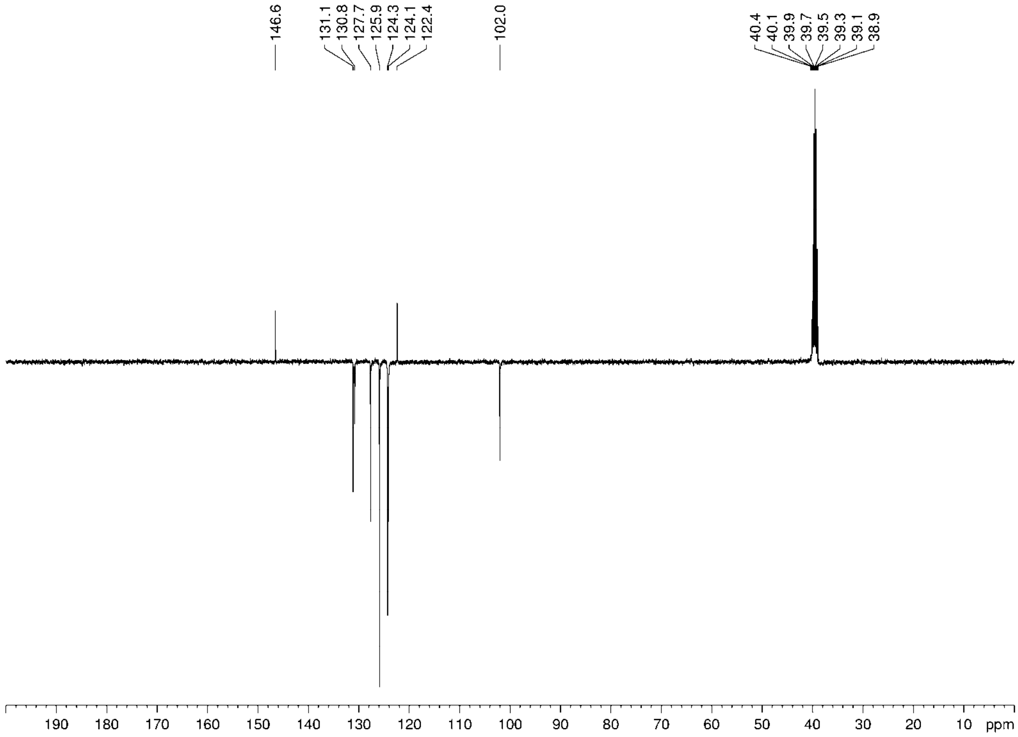
146.6
131.1 130.8 127.7 125.9 124.3 124.1 122.4
102.0
40.4 40.1 39.9 39.7 39.5 39.3 39.1 38.9
190 180 170 160 150 140 130 120 110 100 90 80 70 60 50 40 30 20 10 ppm

sery383

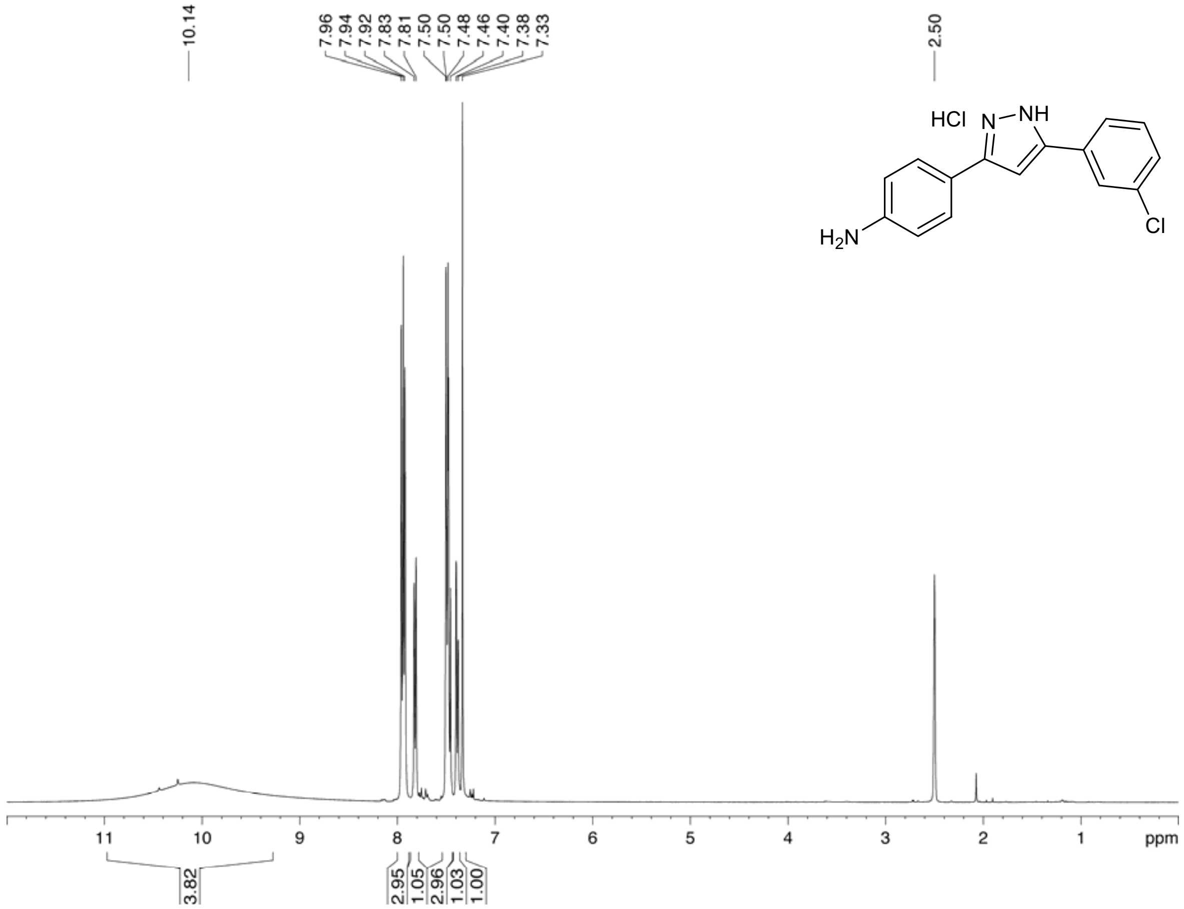
10.14
7.96
7.94
7.92
7.83
7.81
7.50
7.50
7.48
7.46
7.40
7.38
7.33
2.50
HCl
N
NH
H_2N
Cl
11
10
9
8
7
6
5
4
3
2
1
ppm
3.82
2.95
1.05
2.96
1.03
1.00

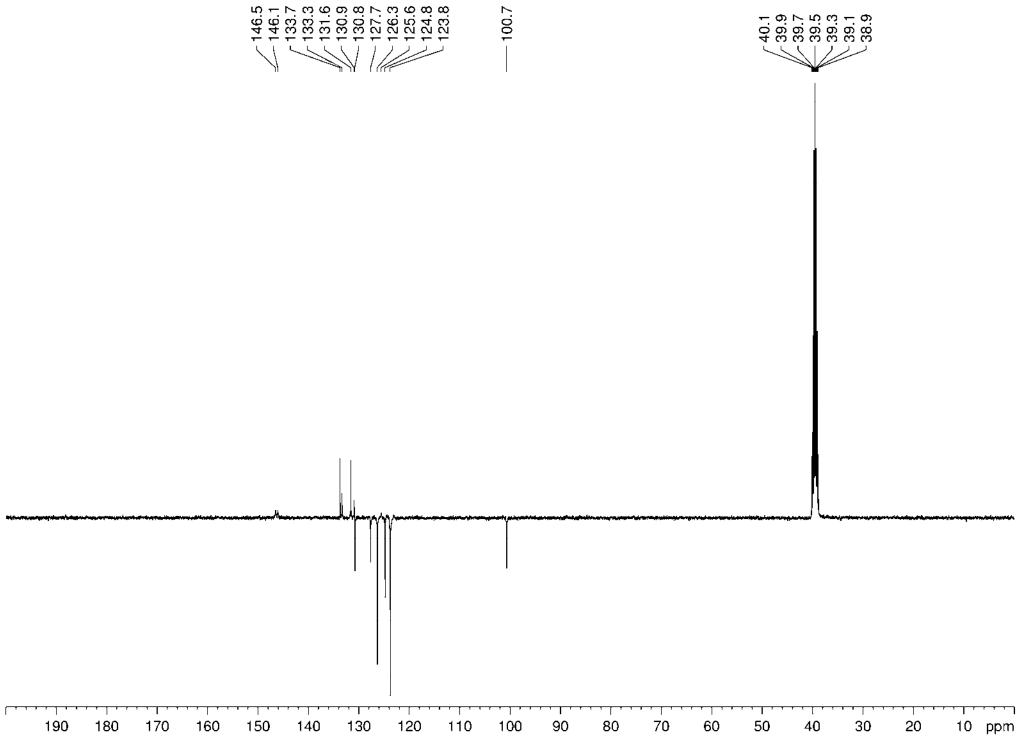
146.5
146.1
133.7
133.3
131.6
130.9
130.8
127.7
126.3
125.6
124.8
123.8
100.7
40.1
39.9
39.7
39.5
39.3
39.1
38.9
190
180
170
160
150
140
130
120
110
100
90
80
70
60
50
40
30
20
10
ppm

sery384

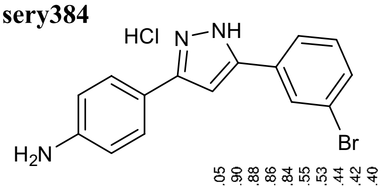
HCl
N–NH
H2N
Br

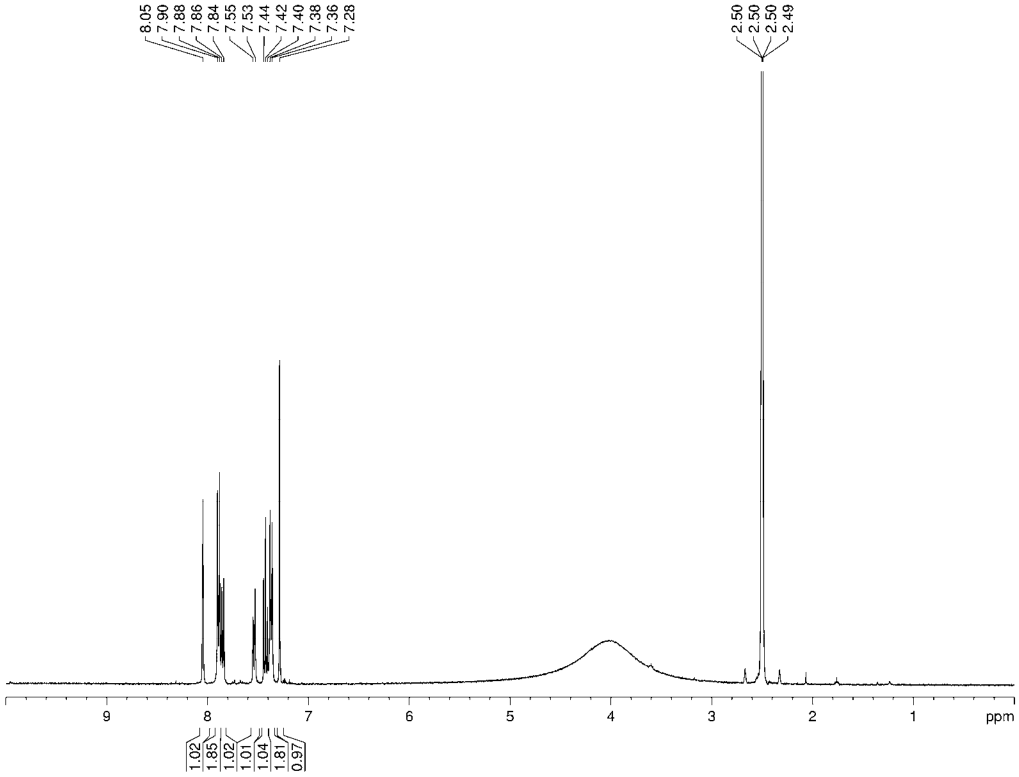
8.05 7.90 7.88 7.86 7.84 7.55 7.53 7.44 7.42 7.40 7.38 7.36 7.28
2.50 2.50 2.50 2.49
1.02 1.85 1.02 1.01 1.04 1.81 0.97
9 8 7 6 5 4 3 2 1 ppm

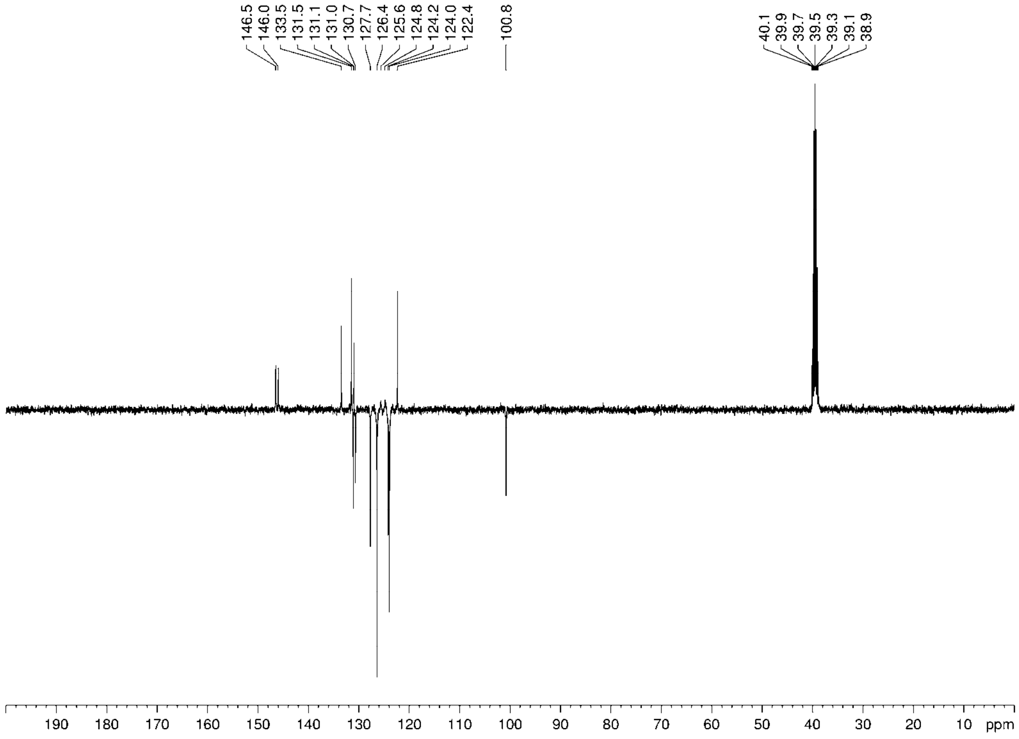
146.5 146.0 133.5 131.5 131.1 131.0 130.7 127.7 126.4 125.6 124.8 124.2 124.0 122.4
100.8
40.1 39.9 39.7 39.5 39.3 39.1 38.9
190 180 170 160 150 140 130 120 110 100 90 80 70 60 50 40 30 20 10 ppm

sery388A

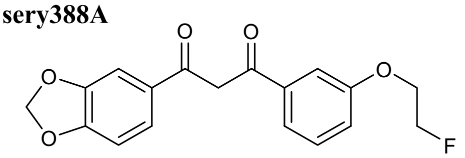
O
O
O
O
O
F

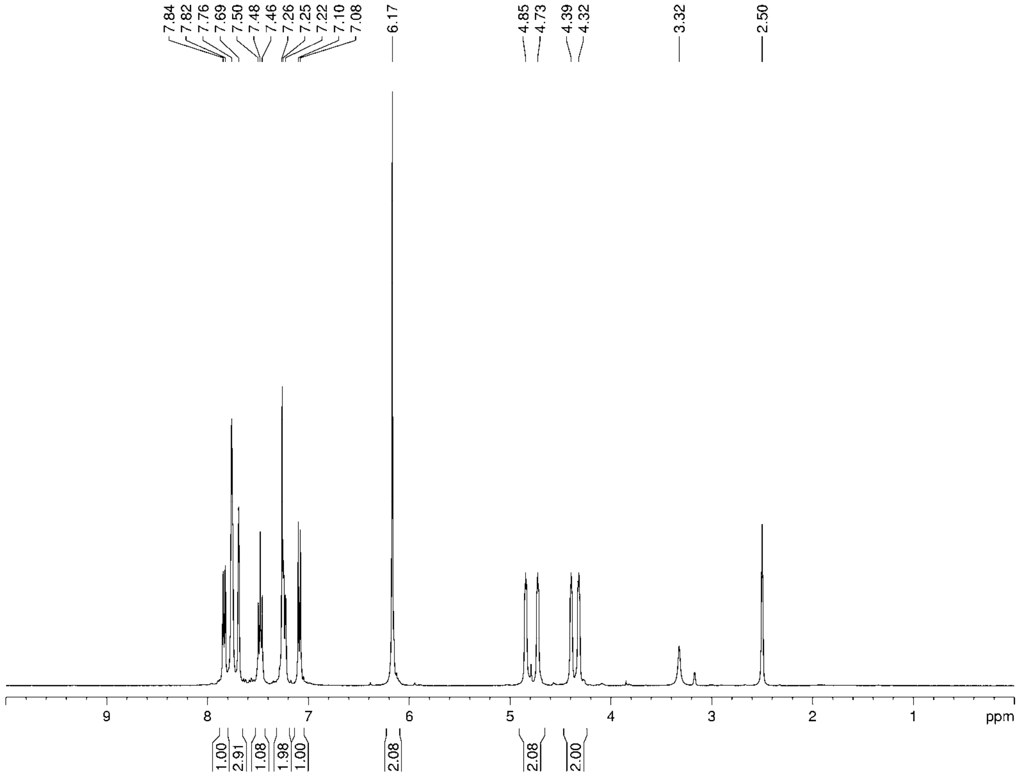
7.84
7.82
7.76
7.69
7.50
7.48
7.46
7.26
7.25
7.22
7.10
7.08
6.17
4.85
4.73
4.39
4.32
3.32
2.50
9
8
7
6
5
4
3
2
1
ppm
1.00
2.91
1.08
1.98
1.00
2.08
2.08
2.00

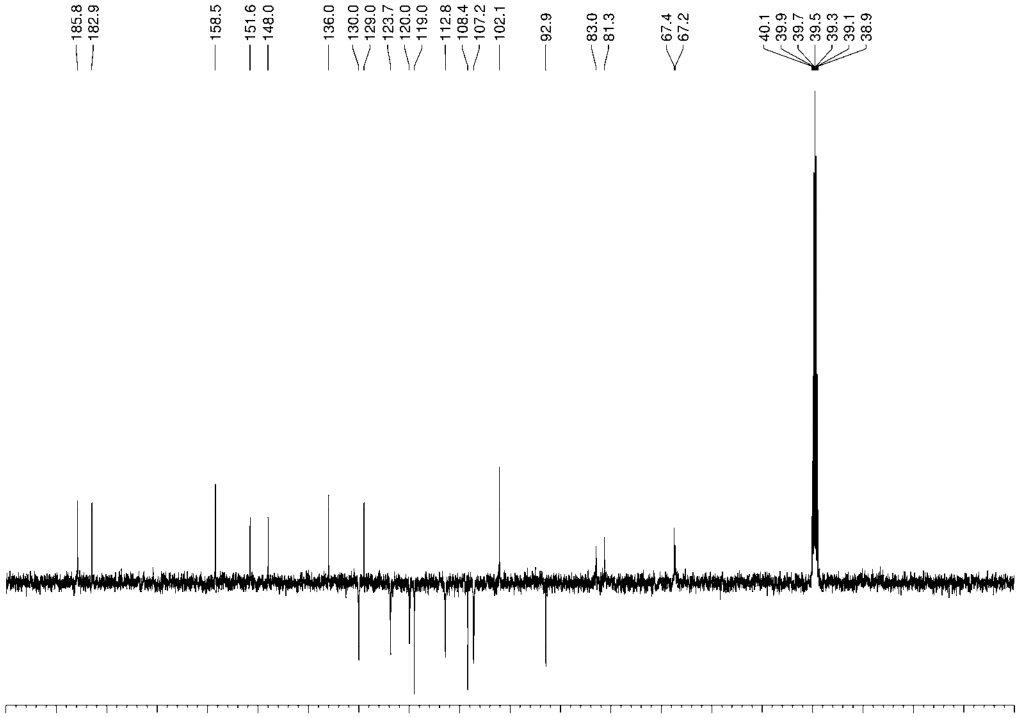
185.8
182.9
158.5
151.6
148.0
136.0
130.0
129.0
123.7
120.0
119.0
112.8
108.4
107.2
102.1
92.9
83.0
81.3
67.4
67.2
40.1
39.9
39.7
39.5
39.3
39.1
38.9
190
180
170
160
150
140
130
120
110
100
90
80
70
60
50
40
30
20
10
ppm

sery392A

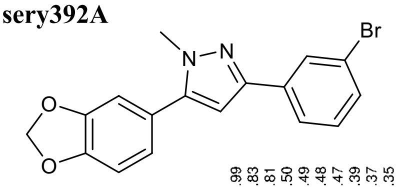

Br
N
N
O
O

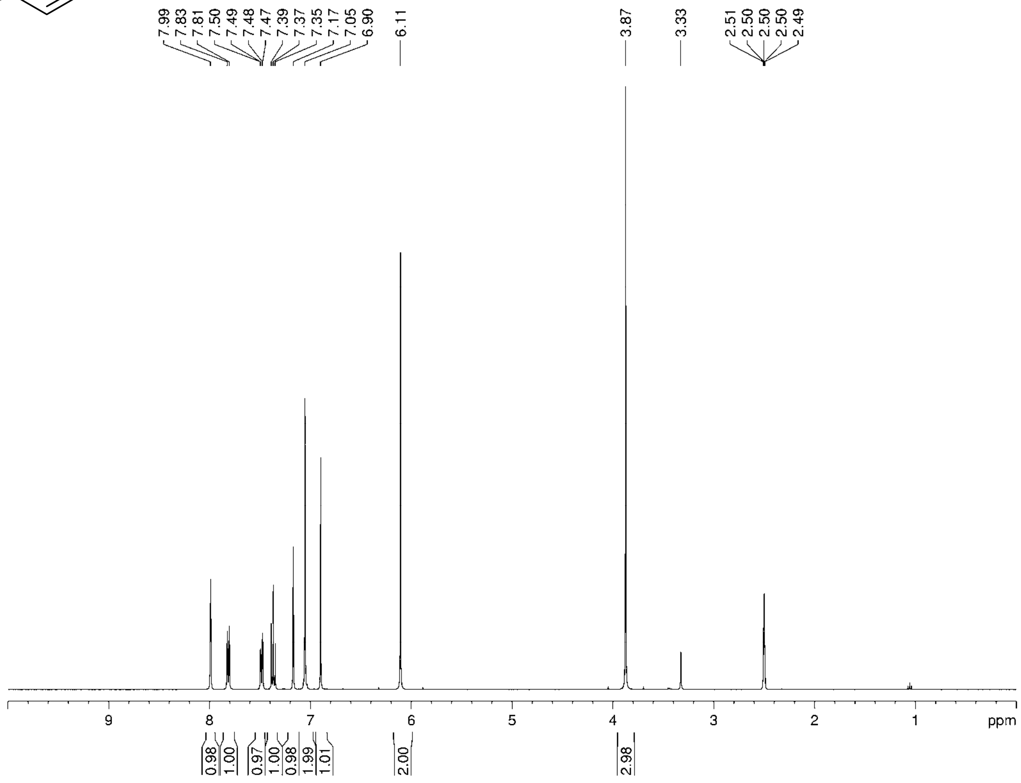

7.99
7.83
7.81
7.50
7.49
7.48
7.47
7.39
7.37
7.35
7.17
7.05
6.90
6.11
3.87
3.33
2.51
2.50
2.50
2.50
2.49
9
8
7
6
5
4
3
2
1
ppm
0.98
1.00
0.97
1.00
0.98
1.99
1.01
2.00
2.98

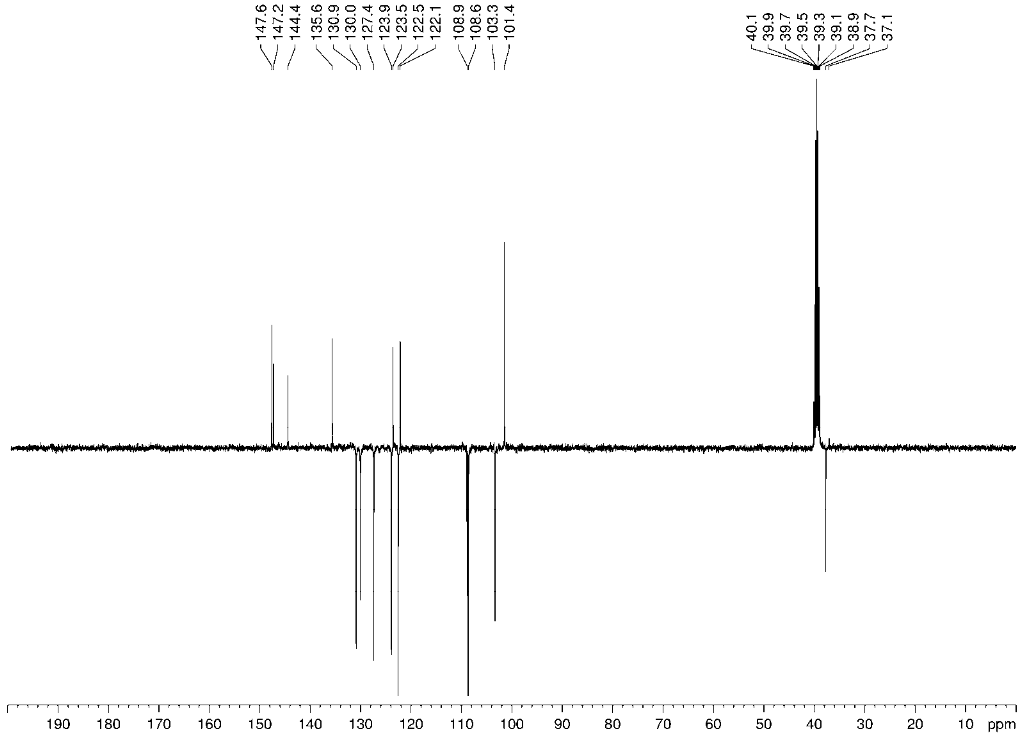

147.6
147.2
144.4
135.6
130.9
130.0
127.4
123.9
123.5
122.5
122.1
108.9
108.6
103.3
101.4
40.1
39.9
39.7
39.5
39.3
39.1
38.9
37.7
37.1
190
180
170
160
150
140
130
120
110
100
90
80
70
60
50
40
30
20
10
ppm

sery392B

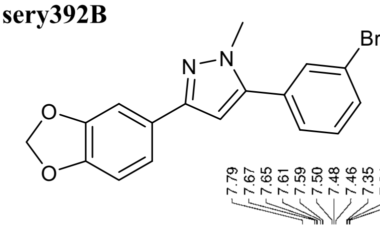

Br
N
N
O
O

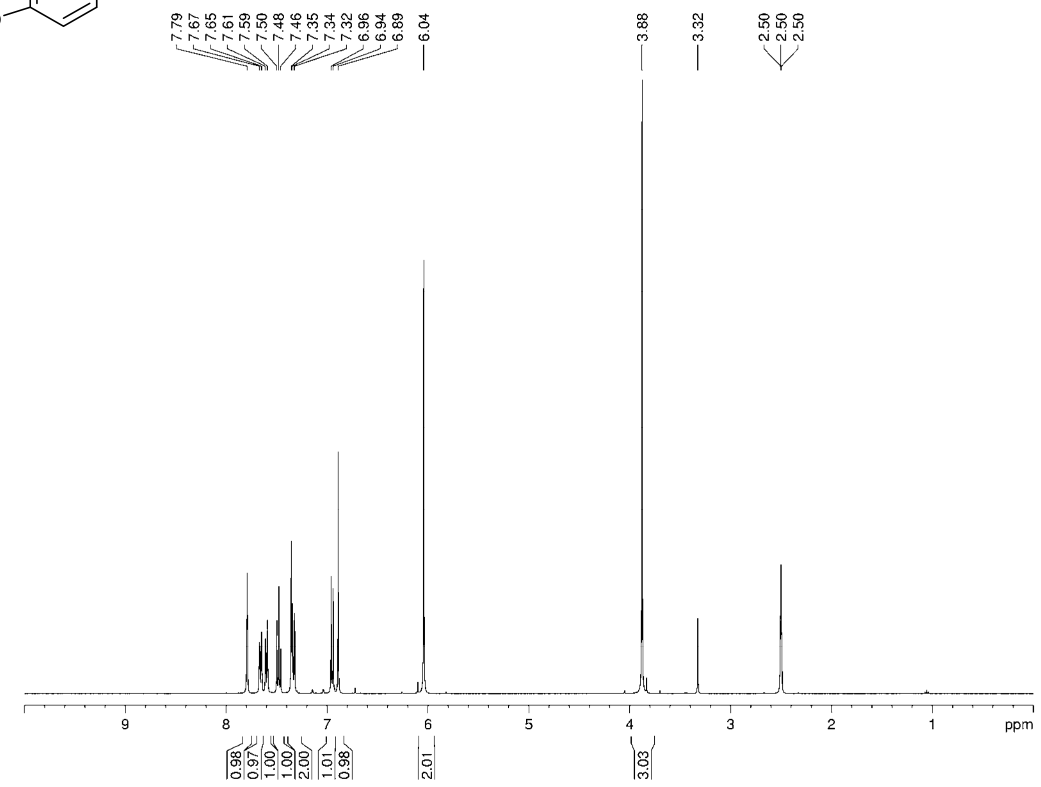

7.79
7.67
7.65
7.61
7.59
7.50
7.48
7.46
7.35
7.34
7.32
6.96
6.94
6.89
6.04
3.88
3.32
2.50
2.50
2.50
9
8
7
6
5
4
3
2
1
ppm
0.98
0.97
1.00
1.00
2.00
1.01
0.98
2.01
3.03

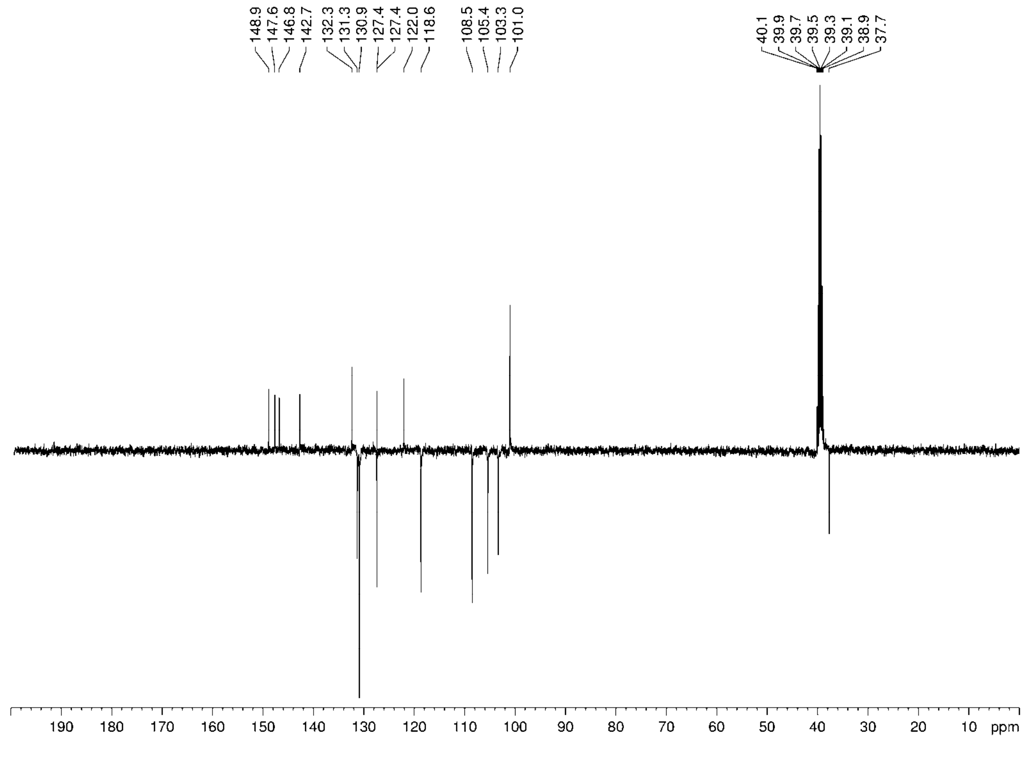

148.9
147.6
146.8
142.7
132.3
131.3
130.9
127.4
127.4
122.0
118.6
108.5
105.4
103.3
101.0
40.1
39.9
39.7
39.5
39.3
39.1
38.9
37.7
190
180
170
160
150
140
130
120
110
100
90
80
70
60
50
40
30
20
10
ppm

sery401A

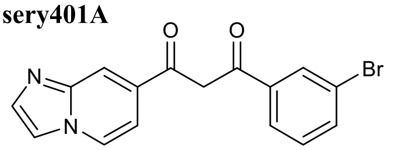

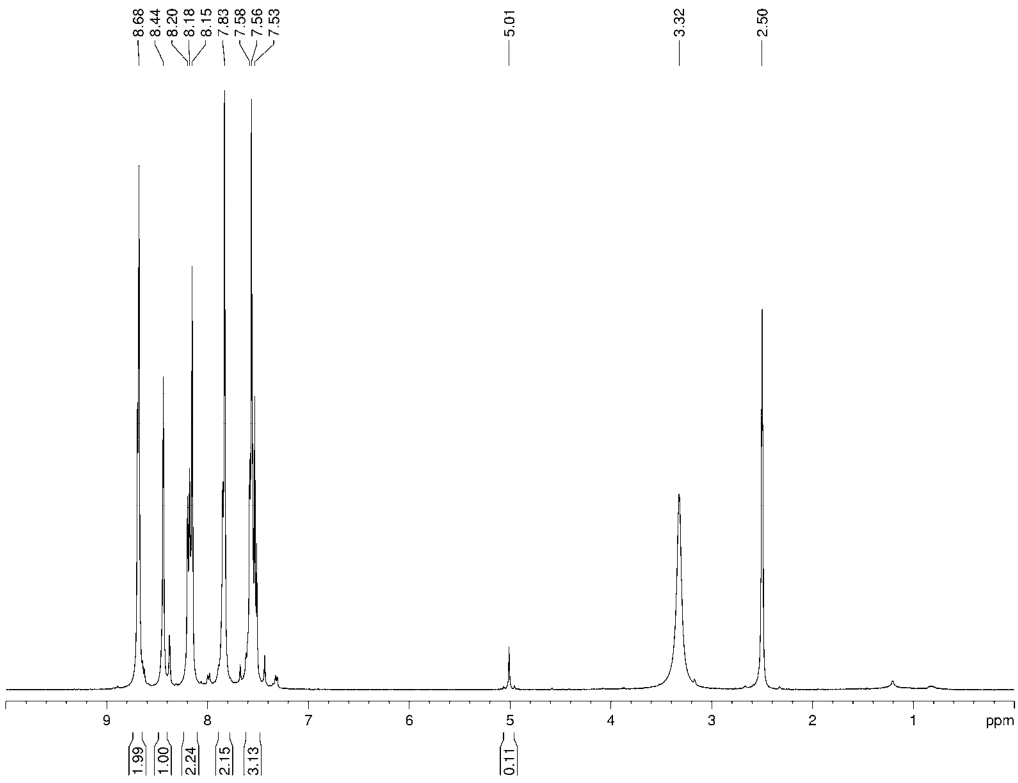

8.68
8.44
8.20
8.18
8.15
7.83
7.58
7.56
7.53
5.01
3.32
2.50
9
8
7
6
5
4
3
2
1
ppm
1.99
1.00
2.24
2.15
3.13
0.11

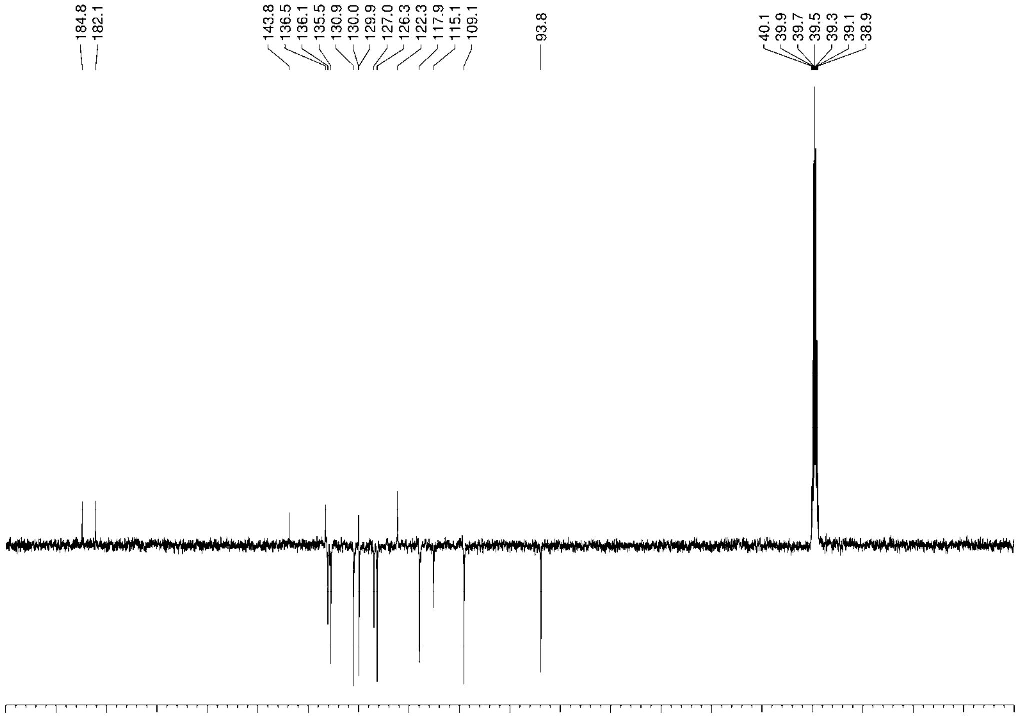

184.8
182.1
143.8
136.5
136.1
135.5
130.9
130.0
129.9
127.0
126.3
122.3
117.9
115.1
109.1
93.8
40.1
39.9
39.7
39.5
39.3
39.1
38.9
190
180
170
160
150
140
130
120
110
100
90
80
70
60
50
40
30
20
10
ppm

sery401B

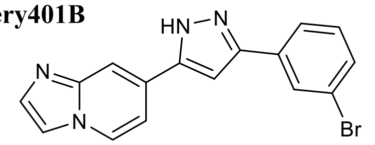
HN–N
N
N
Br

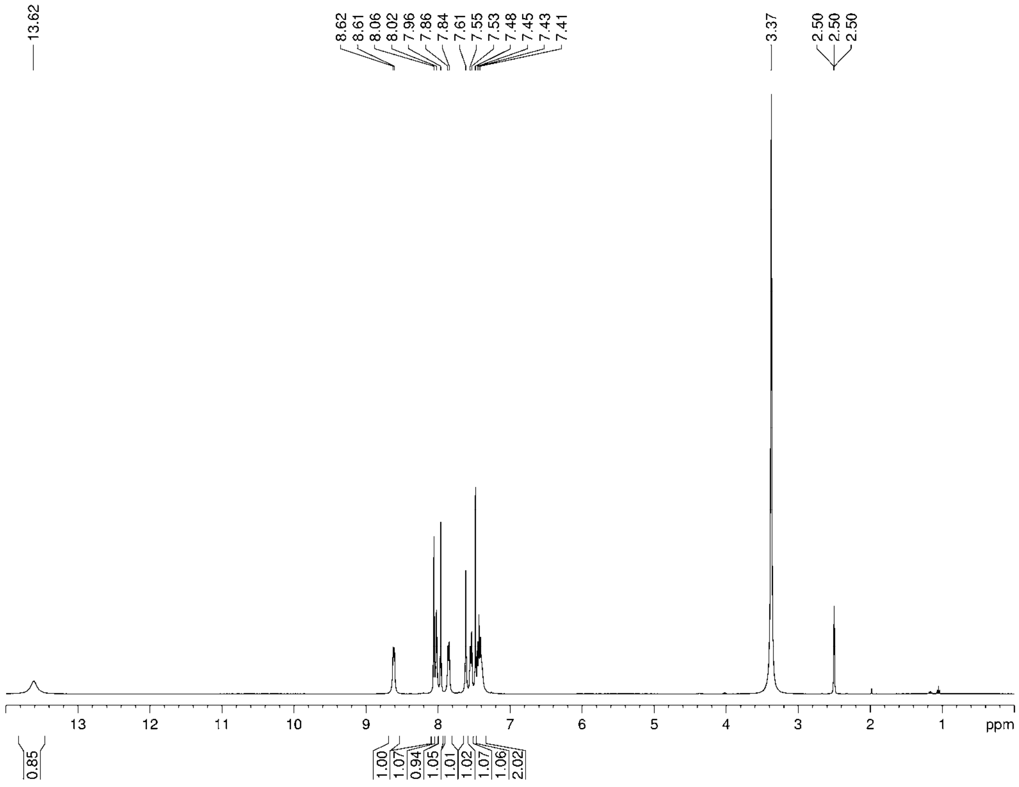
13.62
8.62 8.61 8.06 8.02 7.96 7.86 7.84 7.61 7.55 7.53 7.48 7.45 7.43 7.41
3.37
2.50 2.50 2.50
13 12 11 10 9 8 7 6 5 4 3 2 1 ppm
0.85
1.00 1.07 0.94 1.05 1.01 1.02 1.07 1.06 2.02

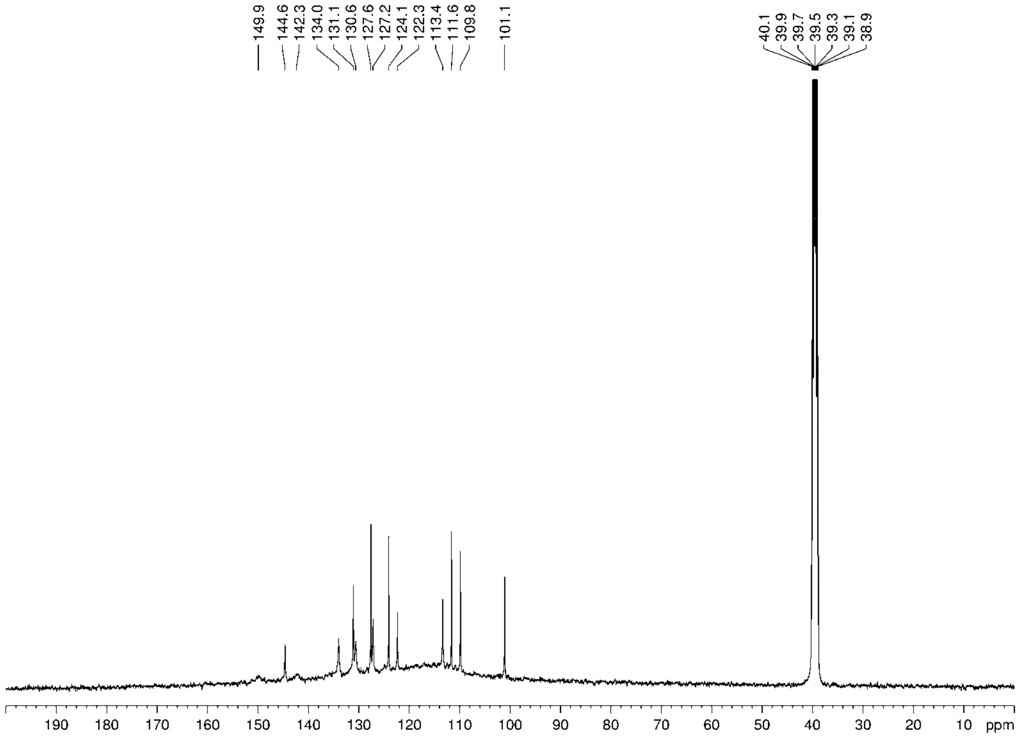
149.9 144.6 142.3 134.0 131.1 130.6 127.6 127.2 124.1 122.3 113.4 111.6 109.8 101.1
40.1 39.9 39.7 39.5 39.3 39.1 38.9
190 180 170 160 150 140 130 120 110 100 90 80 70 60 50 40 30 20 10 ppm

sery407

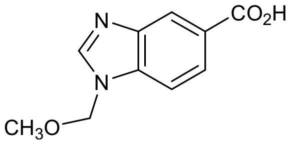
N
CO2H
N
CH3O

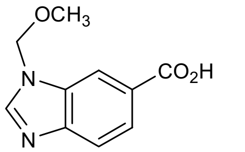
OCH3
N
CO2H
N

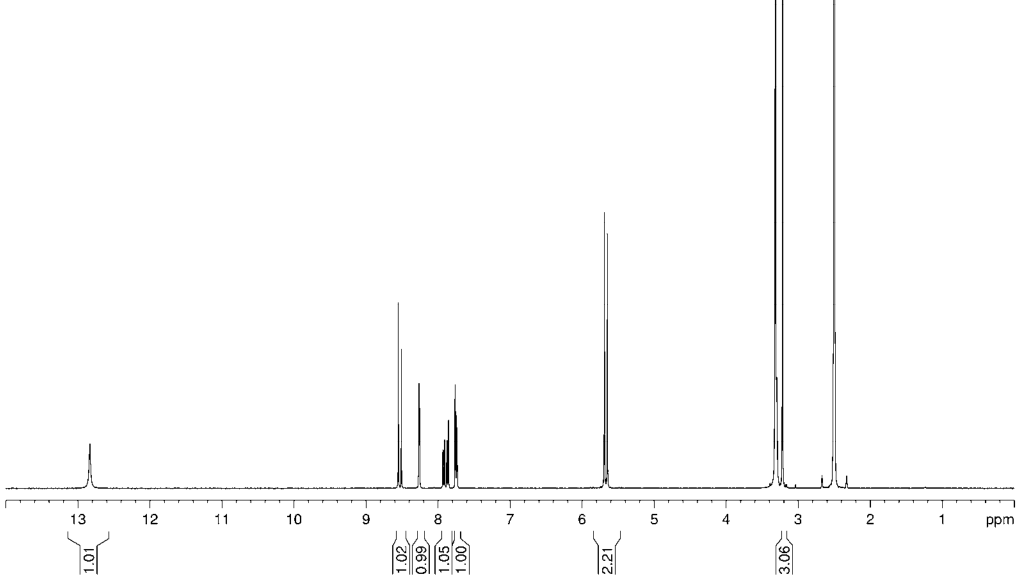
8.55
8.51
8.27
8.26
7.93
7.93
7.91
7.91
7.88
7.88
7.86
7.85
7.76
7.76
7.74
7.73
5.69
5.65
3.32
3.22
3.22
2.50
2.50
2.50
13
12
11
10
9
8
7
6
5
4
3
2
1
ppm
1.01
1.02
0.99
1.05
1.00
2.21
3.06

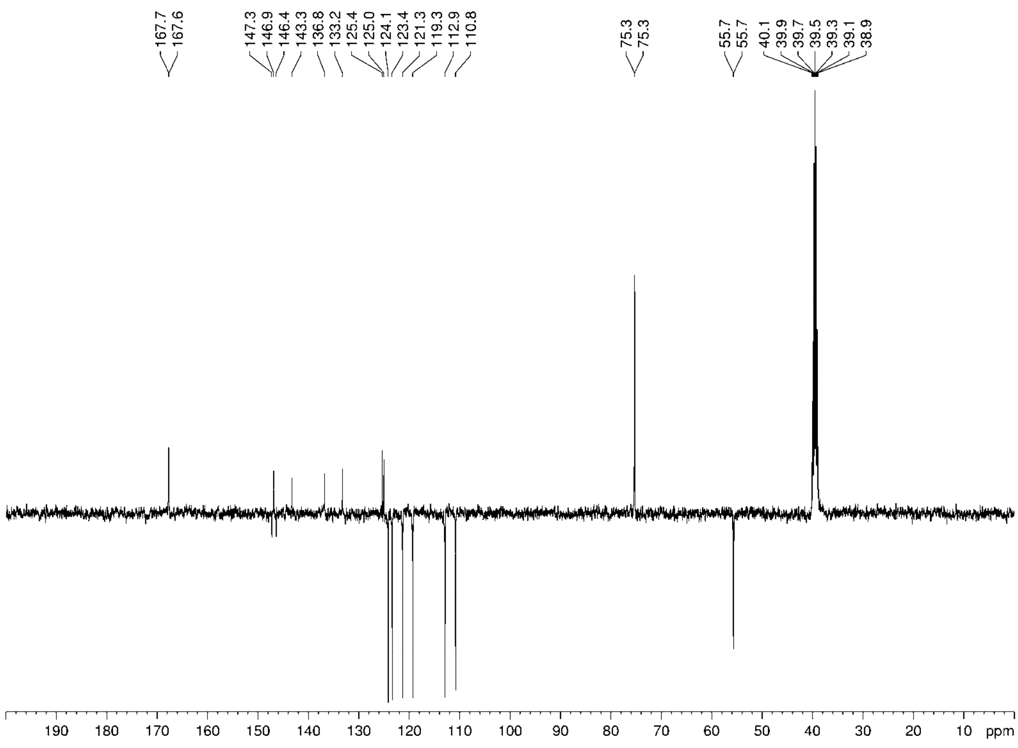
167.7
167.6
147.3
146.9
146.4
143.3
136.8
133.2
125.4
125.0
124.1
123.4
121.3
119.3
112.9
110.8
75.3
75.3
55.7
55.7
40.1
39.9
39.7
39.5
39.3
39.1
38.9
190
180
170
160
150
140
130
120
110
100
90
80
70
60
50
40
30
20
10
ppm

sery415

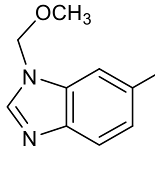
OCH3
N
N
N–NH
Br

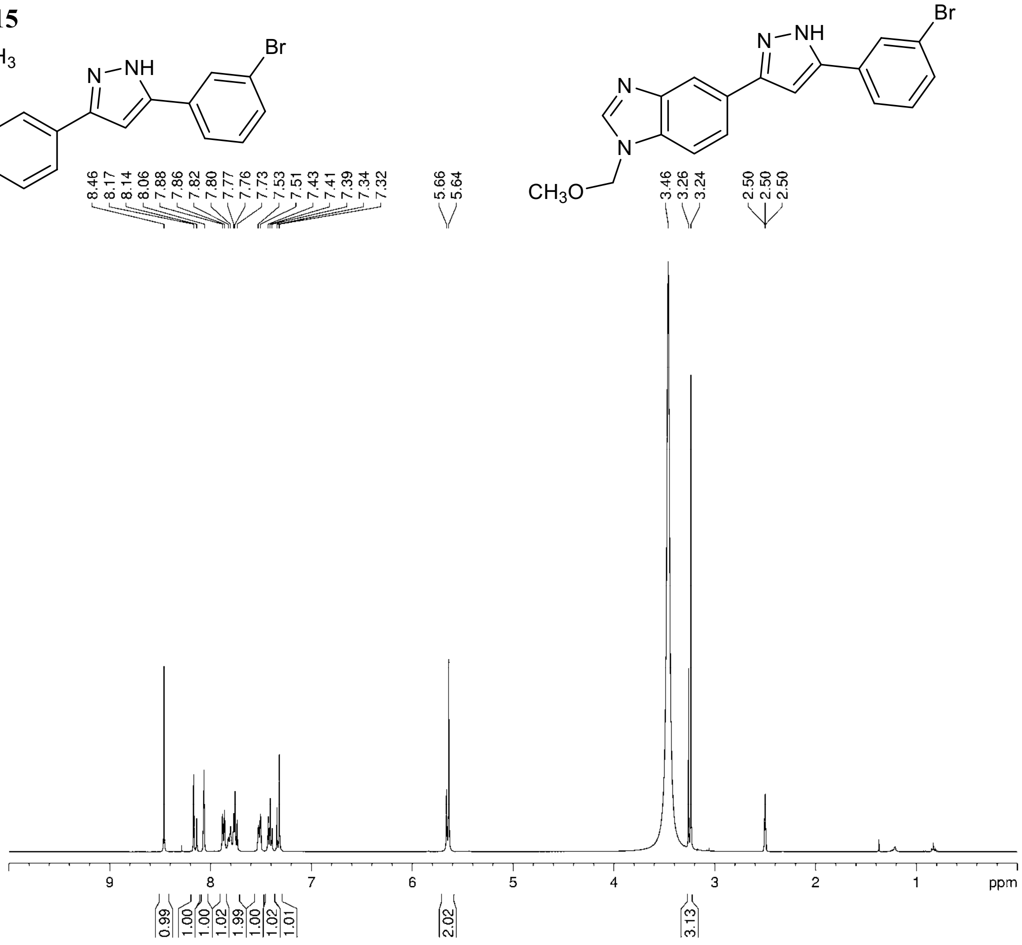
8.46 8.17 8.14 8.06 7.88 7.86 7.82 7.80 7.77 7.76 7.73 7.53 7.51 7.43 7.41 7.39 7.34 7.32
5.66 5.64
3.46 3.26 3.24
2.50 2.50 2.50
CH3O
N–NH
Br
9 8 7 6 5 4 3 2 1 ppm
0.99 1.00 1.00 1.02 1.99 1.00 1.02 1.01
2.02
3.13

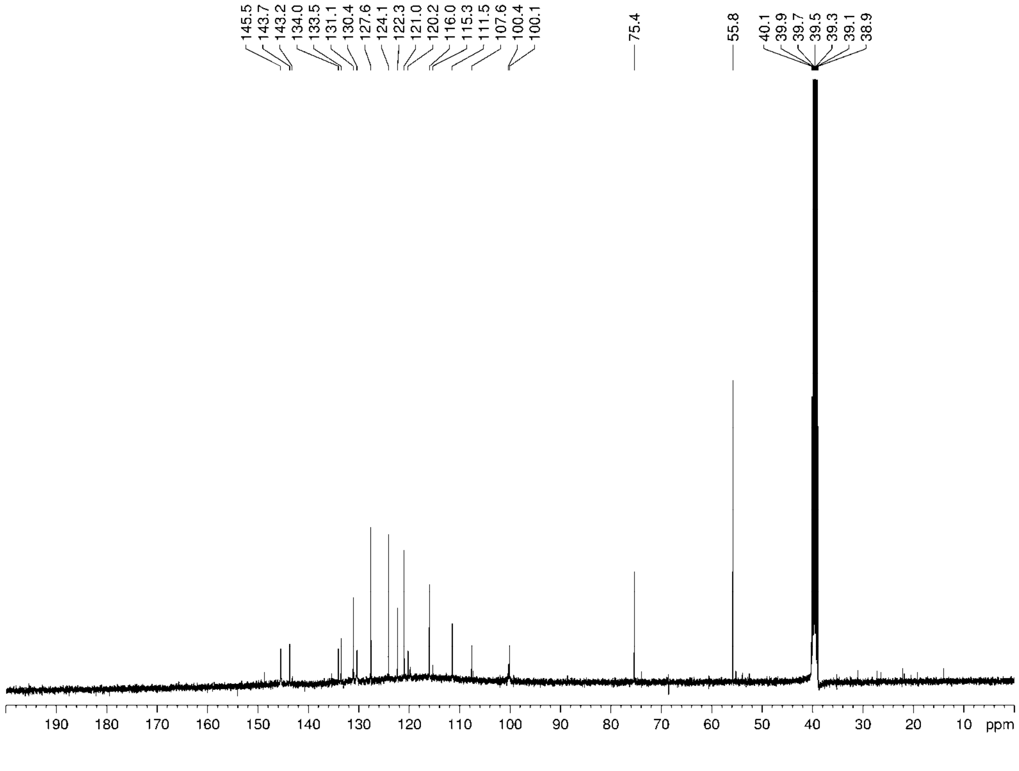
145.5 143.7 143.2 134.0 133.5 131.1 130.4 127.6 124.1 122.3 121.0 120.2 116.0 115.3 111.5 107.6 100.4 100.1
75.4
55.8
40.1 39.9 39.7 39.5 39.3 39.1 38.9
190 180 170 160 150 140 130 120 110 100 90 80 70 60 50 40 30 20 10 ppm

sery417

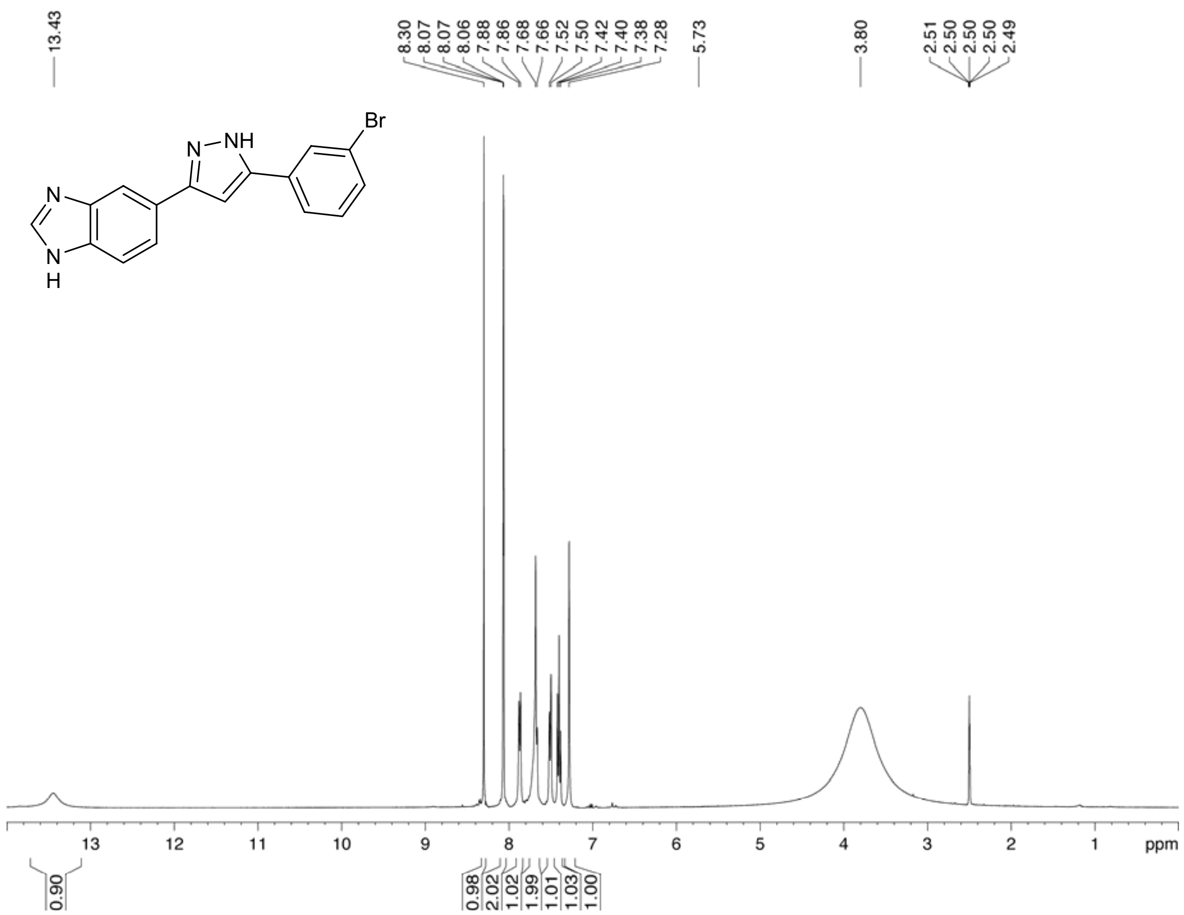
13.43
8.30
8.07
8.07
8.06
7.88
7.86
7.68
7.66
7.52
7.50
7.42
7.40
7.38
7.28
5.73
3.80
2.51
2.50
2.50
2.50
2.49
Br
N–NH
N
N
H
13 12 11 10 9 8 7 6 5 4 3 2 1 ppm
0.90
0.98
2.02
1.02
1.99
1.01
1.03
1.00

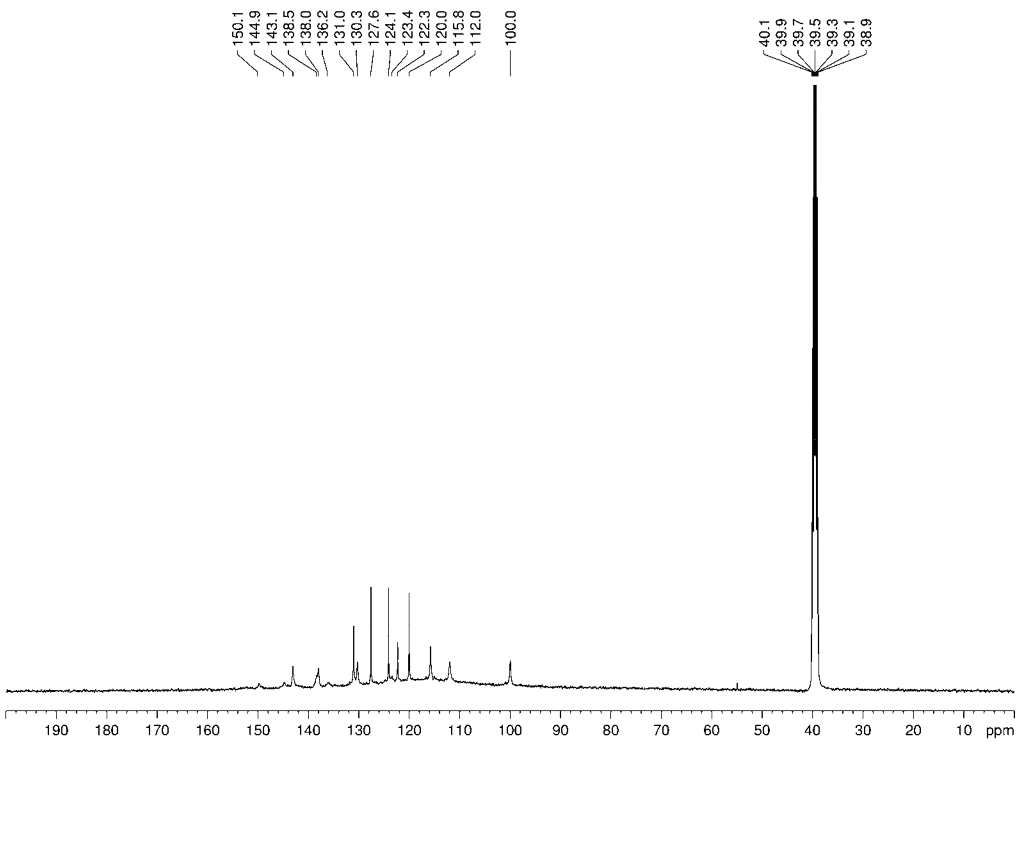
150.1
144.9
143.1
138.5
138.0
136.2
131.0
130.3
127.6
124.1
123.4
122.3
120.0
115.8
112.0
100.0
40.1
39.9
39.7
39.5
39.3
39.1
38.9
190 180 170 160 150 140 130 120 110 100 90 80 70 60 50 40 30 20 10 ppm

sery455A

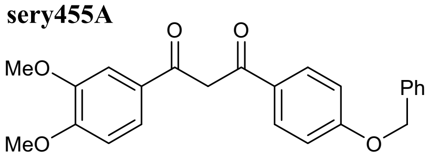
MeO
MeO
O
O
Ph
O

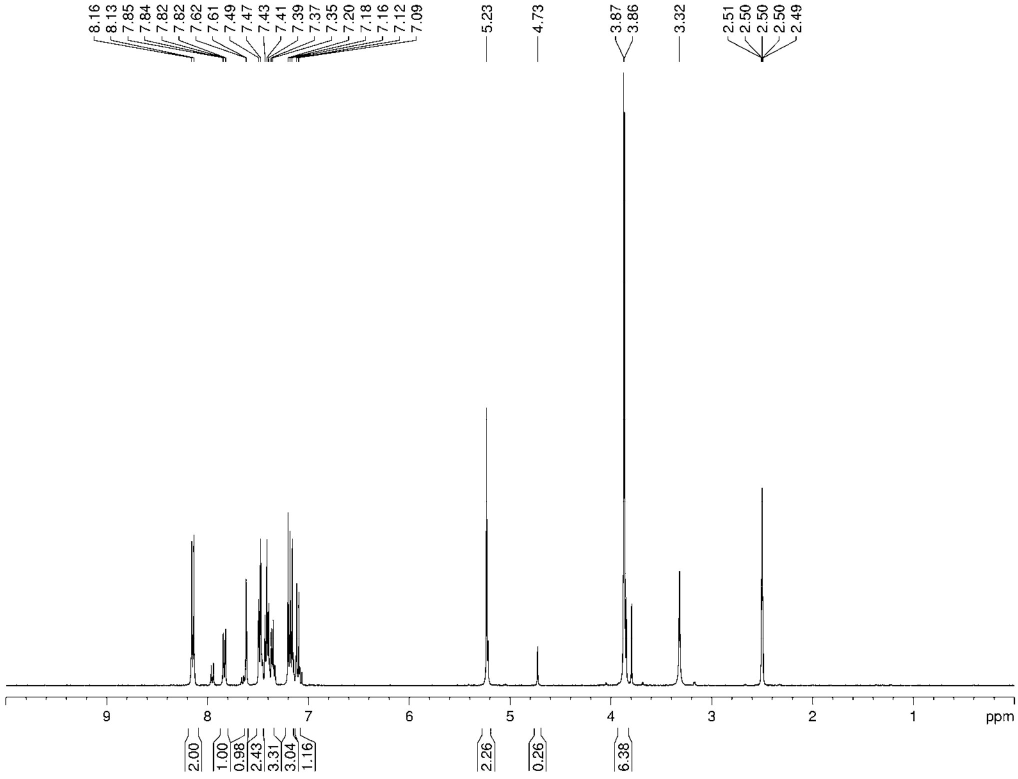
8.16
8.13
7.85
7.84
7.82
7.82
7.62
7.61
7.49
7.47
7.43
7.41
7.39
7.37
7.35
7.20
7.18
7.16
7.12
7.09
5.23
4.73
3.87
3.86
3.32
2.51
2.50
2.50
2.50
2.49
9
8
7
6
5
4
3
2
1
ppm
2.00
1.00
0.98
2.43
3.31
3.04
1.16
2.26
0.26
6.38

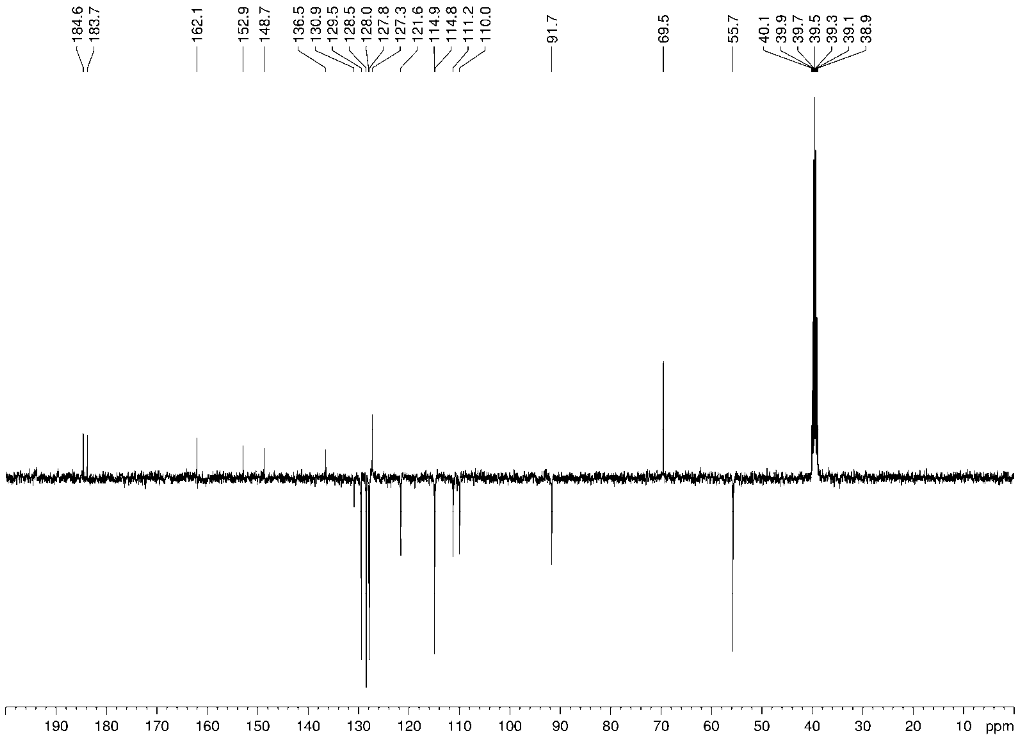
184.6
183.7
162.1
152.9
148.7
136.5
130.9
129.5
128.5
128.0
127.8
127.3
121.6
114.9
114.8
111.2
110.0
91.7
69.5
55.7
40.1
39.9
39.7
39.5
39.3
39.1
38.9
190
180
170
160
150
140
130
120
110
100
90
80
70
60
50
40
30
20
10
ppm

sery455B

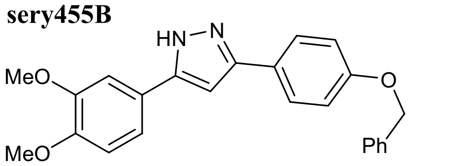
HN–N
MeO
MeO
O
Ph

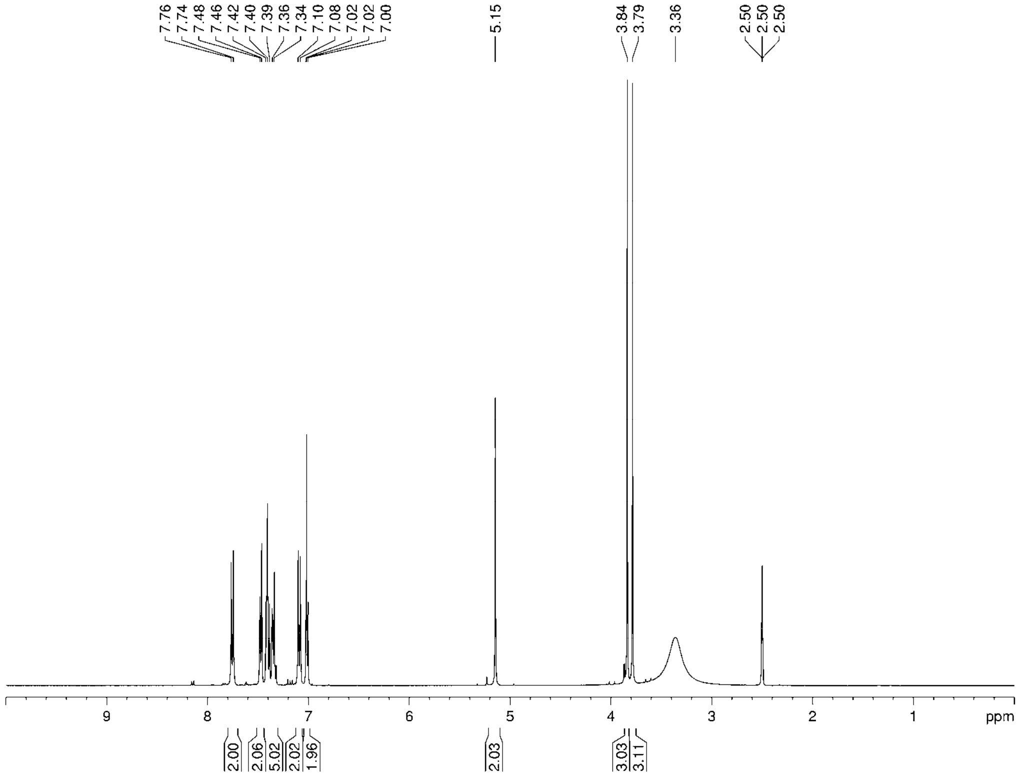
7.76
7.74
7.48
7.46
7.42
7.40
7.39
7.36
7.34
7.10
7.08
7.02
7.02
7.00
5.15
3.84
3.79
3.36
2.50
2.50
2.50
9
8
7
6
5
4
3
2
1
ppm
2.00
2.06
5.02
2.02
1.96
2.03
3.03
3.11

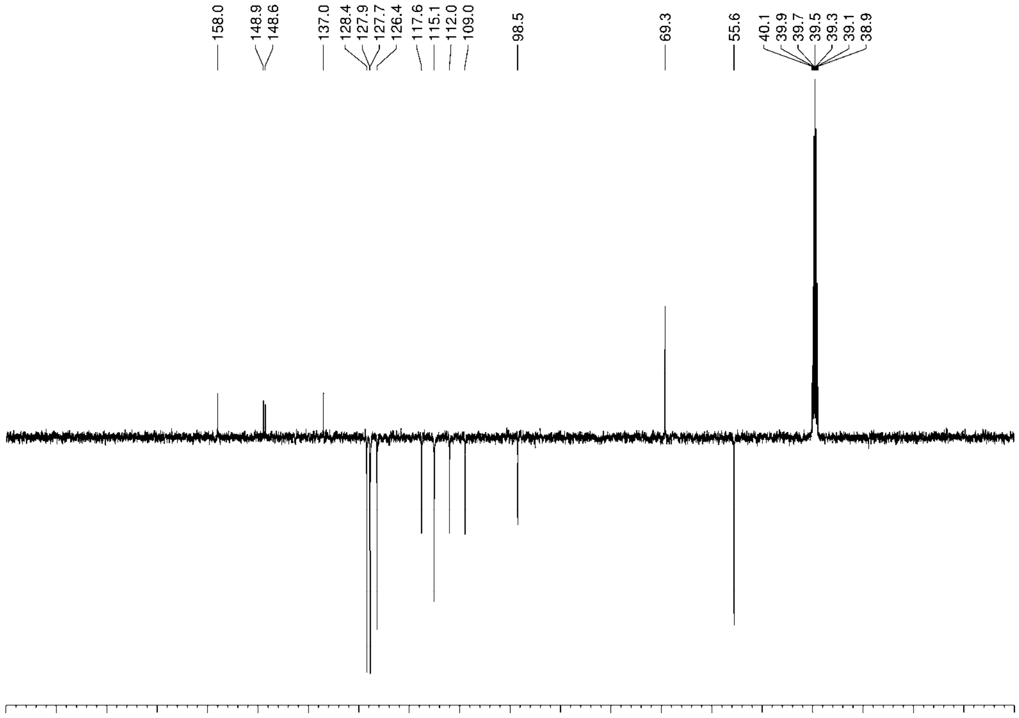
158.0
148.9
148.6
137.0
128.4
127.9
127.7
126.4
117.6
115.1
112.0
109.0
98.5
69.3
55.6
40.1
39.9
39.7
39.5
39.3
39.1
38.9
190
180
170
160
150
140
130
120
110
100
90
80
70
60
50
40
30
20
10
ppm

sery456A

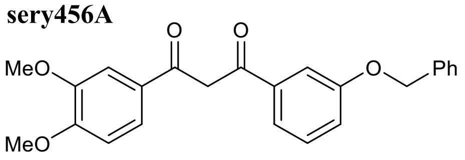
MeO
MeO
O
O
O
Ph

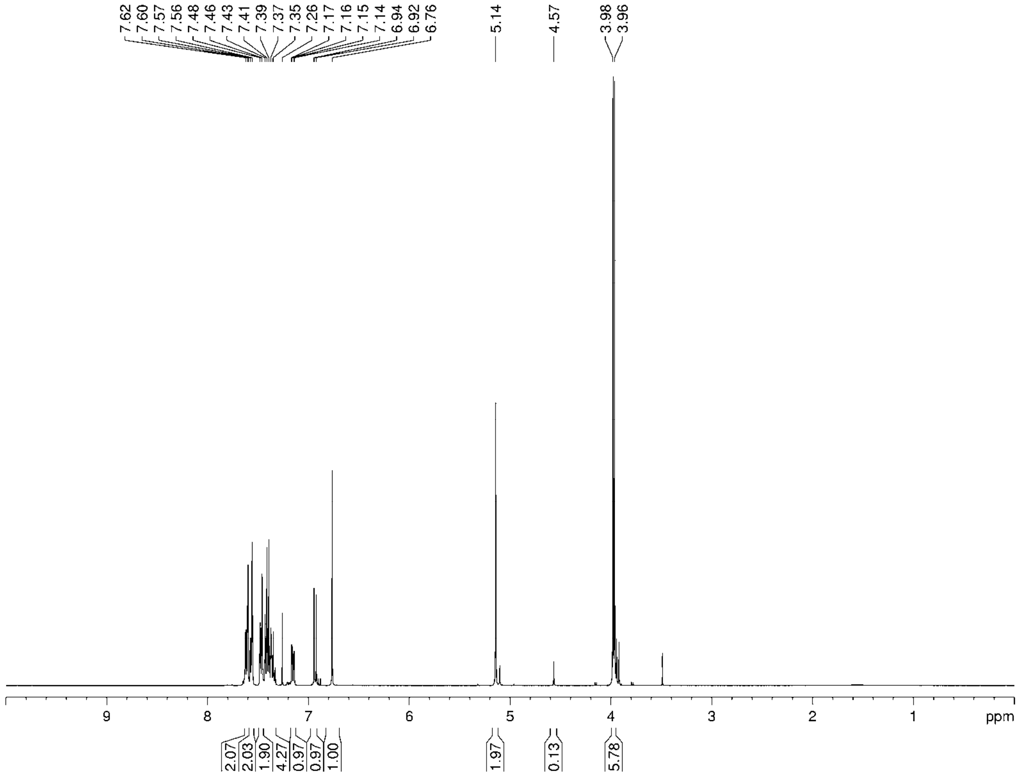
7.62
7.60
7.57
7.56
7.48
7.46
7.43
7.41
7.39
7.37
7.35
7.26
7.17
7.16
7.15
7.14
6.94
6.92
6.76
5.14
4.57
3.98
3.96
9
8
7
6
5
4
3
2
1
ppm
2.07
2.03
1.90
4.27
0.97
0.97
1.00
1.97
0.13
5.78

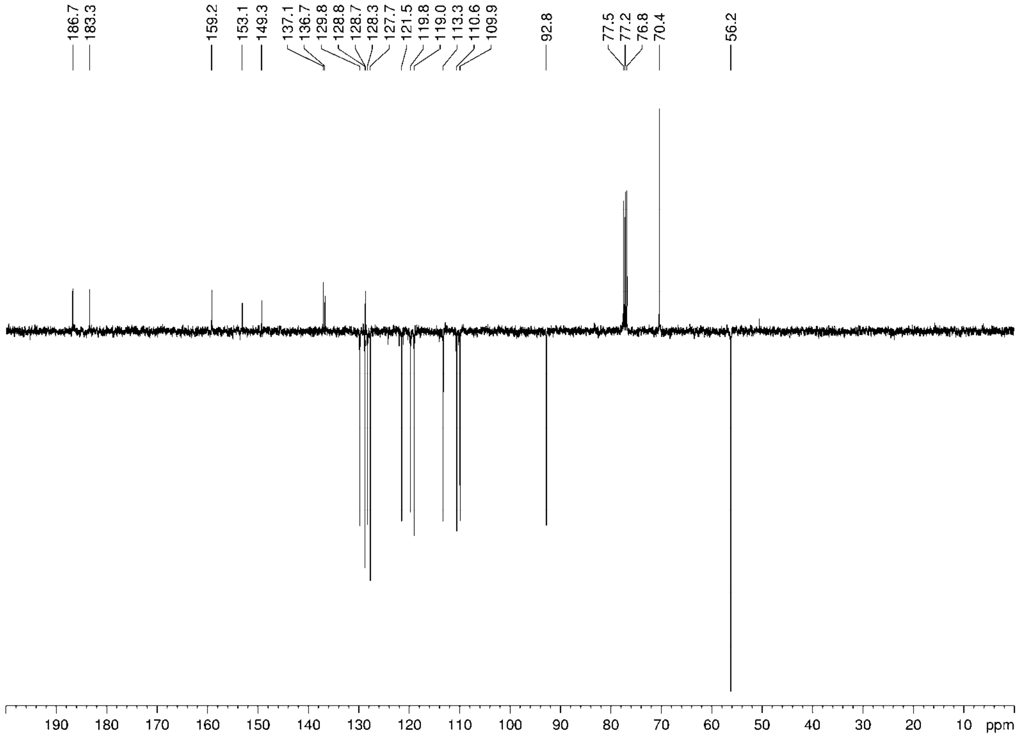
186.7
183.3
159.2
153.1
149.3
137.1
136.7
129.8
128.8
128.7
128.3
127.7
121.5
119.8
119.0
113.3
110.6
109.9
92.8
77.5
77.2
76.8
70.4
56.2
190
180
170
160
150
140
130
120
110
100
90
80
70
60
50
40
30
20
10
ppm

sery456B

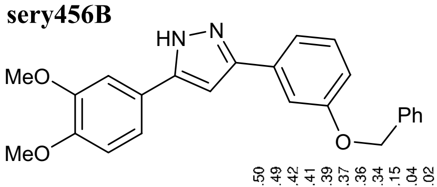
HN–N
MeO
MeO
O
Ph

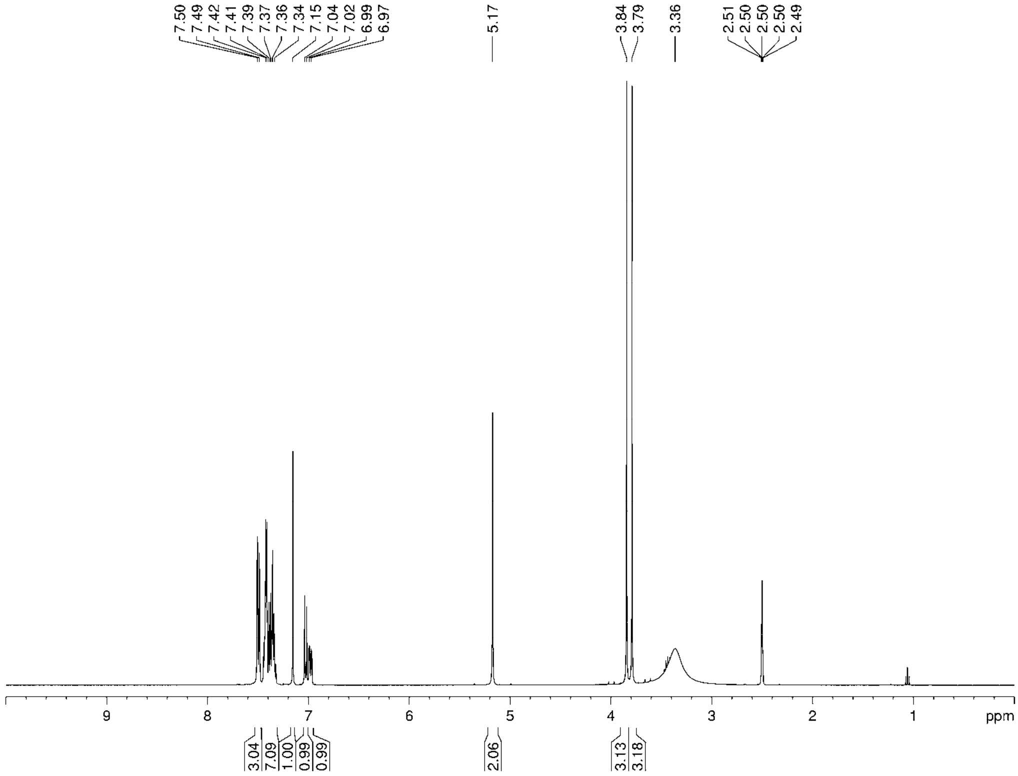
7.50
7.49
7.42
7.41
7.39
7.37
7.36
7.34
7.15
7.04
7.02
6.99
6.97
5.17
3.84
3.79
3.36
2.51
2.50
2.50
2.50
2.49
9
8
7
6
5
4
3
2
1
ppm
3.04
7.09
1.00
0.99
0.99
2.06
3.13
3.18

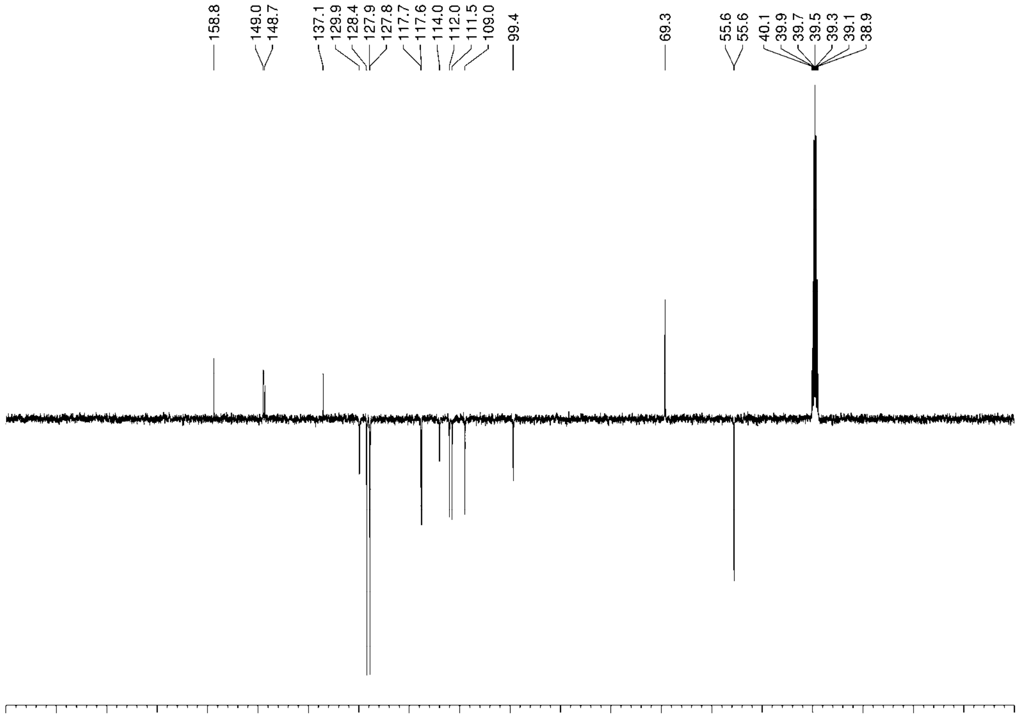
158.8
149.0
148.7
137.1
129.9
128.4
127.9
127.8
117.7
117.6
114.0
112.0
111.5
109.0
99.4
69.3
55.6
55.6
40.1
39.9
39.7
39.5
39.3
39.1
38.9
190
180
170
160
150
140
130
120
110
100
90
80
70
60
50
40
30
20
10
ppm
